# Multisite test–retest reliability and compatibility of brain metrics derived from FreeSurfer versions 7.1, 6.0, and 5.3

**DOI:** 10.1002/hbm.26147

**Published:** 2022-11-27

**Authors:** Elizabeth Haddad, Fabrizio Pizzagalli, Alyssa H. Zhu, Ravi R. Bhatt, Tasfiya Islam, Iyad Ba Gari, Daniel Dixon, Sophia I. Thomopoulos, Paul M. Thompson, Neda Jahanshad

**Affiliations:** ^1^ Imaging Genetics Center, Mark and Mary Stevens Neuroimaging and Informatics Institute, Keck School of Medicine University of Southern California Marina del Rey California USA; ^2^ Department of Neurosciences University of Turin Turin Italy

**Keywords:** MRI, neuroimaging, reliability, surface area, thickness, version compatibility

## Abstract

Automatic neuroimaging processing tools provide convenient and systematic methods for extracting features from brain magnetic resonance imaging scans. One tool, FreeSurfer, provides an easy‐to‐use pipeline to extract cortical and subcortical morphometric measures. There have been over 25 stable releases of FreeSurfer, with different versions used across published works. The reliability and compatibility of regional morphometric metrics derived from the most recent version releases have yet to be empirically assessed. Here, we used test–retest data from three public data sets to determine within‐version reliability and between‐version compatibility across 42 regional outputs from FreeSurfer versions 7.1, 6.0, and 5.3. Cortical thickness from v7.1 was less compatible with that of older versions, particularly along the cingulate gyrus, where the lowest version compatibility was observed (intraclass correlation coefficient 0.37–0.61). Surface area of the temporal pole, frontal pole, and medial orbitofrontal cortex, also showed low to moderate version compatibility. We confirm low compatibility between v6.0 and v5.3 of pallidum and putamen volumes, while those from v7.1 were compatible with v6.0. Replication in an independent sample showed largely similar results for measures of surface area and subcortical volumes, but had lower overall regional thickness reliability and compatibility. Batch effect correction may adjust for some inter‐version effects when most sites are run with one version, but results vary when more sites are run with different versions. Age associations in a quality controlled independent sample (*N* = 106) revealed version differences in results of downstream statistical analysis. We provide a reference to highlight the regional metrics that may yield recent version‐related inconsistencies in published findings. An interactive viewer is provided at http://data.brainescience.org/Freesurfer_Reliability/.

## Graphical abstract

We use test–retest data from three public data sets to determine within‐version reliability and between‐version compatibility across 42 regional outputs from FreeSurfer version 7.1, 6.0, and 5.3. Overall, we find generally high within‐version reliability across most versions, however, considerable differences are observed when analyzing between‐version compatibility for regional cortical thickness, surface area, and subcortical volumes.

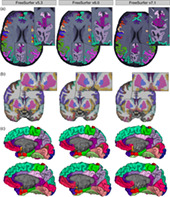

## 1 INTRODUCTION

The reproducibility of research findings in the biological sciences has recently come to light as a major problem, particularly for the neuroimaging‐heavy fields of psychological and neurological‐sciences (Boekel et al., 2015; Bowring et al., 2019; Button et al., 2013; Hodge et al., 2020; Poldrack et al., 2020). Studies on major depressive disorder (MDD), for example, have pointed out inconsistencies in results as well as difficulties in drawing comparisons due to analytical and study design variability (Beijers et al., 2019; Dichter et al., 2015; Fonseka et al., 2018; Kang & Cho, 2020; Müller et al., 2017; Stuhrmann et al., 2011). In one study, using a more heterogeneous sample and rigorous statistical testing, Dinga et al. (2019) were unable to replicate the statistical significance used to define MDD biotypes previously found in the literature. Inconsistent results investigating neuroimaging traits and diseases have also been found in studies of insomnia (Spiegelhalder et al., 2015) and mild traumatic brain injury (mTBI). A meta‐analysis of 14 reports of working memory in mTBI showed mixed findings of functional magnetic resonance imaging (MRI) hyperactivity, hypoactivity, and some studies even report both hyper and hypo activity (Bryer et al., 2013). Neuroimaging offers mechanistic insights into the variability that leads to risk for brain dysfunction, yet these findings must be replicable in order to extend the use of MRI‐derived biomarkers to a clinical setting.

It is important to understand how and why these discrepancies occur, so that we can better understand why certain findings are, or are not reproducible. For example, studies may be underpowered, or the variable of interest might have different effects across populations. Experimental results can also be affected by methodological factors such as the type of data collection (Han et al., 2006; Jovicich et al., 2009; Yan et al., 2020), data processing and analysis (Bennett & Miller, 2013; Botvinik‐Nezer et al., 2020; Carp, 2012; Lindquist, 2020), tool version and selection (Bigler et al., 2020; Dickie et al., 2017; Gronenschild et al., 2012; Meijerman et al., 2018; Perlaki et al., 2017; Tustison et al., 2014; Zavaliangos‐Petropulu et al., 2022), and even operating system environments (Glatard et al., 2015). The presence of pathological tissue has also been reported to cause systematic errors in segmentation output (Dadar et al., 2021). If sample population and methodology differ, it can be difficult to tease apart the main source of the discrepant findings.

Recent efforts in the neuroimaging community have heightened awareness and partially addressed concerns surrounding reproducibility. Guides and tools for enhancing reproducibility have been published in an effort to promote *Open Science*. Open science aims to provide transparency into research studies to better understand the data collected, the code implemented and software used, the analysis performed, and the full scope of results, including null findings (Gorgolewski et al., 2015; Gorgolewski & Poldrack, 2016; Kennedy et al., 2019; Nichols et al., 2017; Poldrack & Gorgolewski, 2017; Vicente‐Saez & Martinez‐Fuentes, 2018; Zuo et al., 2014). These efforts often include detailed documentation and containerization of analytical software to ensure consistency of software version, and even operating system to the extent possible should the study be replicated. Other efforts such as the Consortium for Reliability and Reproducibility (CoRR) emphasize reliability and reproducibility in neuroimaging. This is demonstrated by their open‐source test–retest data sets which help facilitate these reliability and reproducibility assessments in both structural and functional MRI (Zuo et al., 2014). Compared to sample size, these metrics are often overlooked, but it is important to note that reliability is a key determinant of statistical power (Zuo et al., 2019). Large consortia, such as the Enhancing NeuroImaging Genetics through Meta‐Analysis (ENIGMA) Consortium, have also addressed issues of low power and varying data processing pipelines by conducting large‐scale harmonized meta‐ and mega‐analyses across international data sets (Thompson et al., 2020). Analytical protocols are proposed and approved by the community in advance; they are then distributed and made readily available. These protocols also include data quality control (QC) guidelines to improve analytic consistency across heterogeneous data sets and populations.

Large, publicly available and densely phenotyped data sets that use these protocols have recently become a powerful resource that has advanced the field of neuroscience (Horien et al., 2021). Studies like the Alzheimer's Disease Neuroimaging Initiative (ADNI) and the UK Biobank collect data from 1000 to 10,000 of individuals (Littlejohns et al., 2020; Weiner et al., 2015) with some collecting longitudinal data that spans well over a decade (Weiner et al., 2017). Automatic segmentation tools are widely used on such data sets and have allowed for tens to hundreds of thousands of scans to be conveniently processed, thus enabling neuroimaging traits to be used in a wide range of clinical and epidemiological studies. However, these tools do not come without challenges and limitations.

Data processed from updated versions of these softwares are continuously released (http://adni.loni.usc.edu/2021/) and this leaves researchers questioning which version is most reliable or whether data and results from work that used prior versions are compatible with those of later releases. If the detected effects depend on the software version used, then that variability could threaten the reproducibility of published research and compromise clinical translation. However, these version updates are often needed to keep up with the many advancements made in the neuroimaging field. For example, version updates may include added options or tools to work with higher resolution images, or more computational efficient image processing pipelines (e.g., the use of GPUs for processing). As newer software releases are made available, we often lack information on whether new results will be consistent with prior findings, and the overall impact of a software upgrade. To understand sources of study variability, it is important to understand how version upgrades may impact outcome measures.

One such automatic feature extraction and quantification tool that is widely used in neuroimaging is FreeSurfer (Fischl, 2012). FreeSurfer is a structural MRI processing suite that allows researchers to obtain brain parcellations and metrics from just a single T1‐weighted image. Running the software involves just a one command, but the process itself is quite extensive—where the single image undergoes over 30 stepwise processing stages (https://surfer.nmr.mgh.harvard.edu/fswiki/recon-all). Notably, more than 60 research papers have been published detailing FreeSurfer's algorithms and workflows (https://www.zotero.org/freesurfer/collections/F5C8FNX8). The overall processing steps include: image preprocessing, brain extraction, gray and white matter segmentation, reconstruction of the white matter and pial surfaces, labeling of cortical and subcortical regions, and a spherical nonlinear registration of the cortical surface using a stereotaxic atlas, allowing for a more accurate alignment of gyral and sulcal landmarks. Users can then extract features, such as cortical thickness (defined as the distance between the white matter and pial surfaces), surface area (or the area of all the triangles on the mesh representing the white matter surface), and cortical and subcortical volumes, measured in cubic millimeters (Fischl, 2012).

A PubMed search of “freesurfer,” in the year 2020 alone, results in a total of 344 publications, indicating its wide use as a neuroimaging resource (https://pubmed.ncbi.nlm.nih.gov/?term=%28freesurfer%29&filter=years.2020-2020). It has been a popular tool for over 20 years throughout which over 25 different stable releases have been disseminated (https://surfer.nmr.mgh.harvard.edu/fswiki/PreviousReleaseNotes). Version release updates have included, for example, improvements in accuracy of the cortical labels or a change/addition in a preprocessing step such as denoising or bias field correction (https://surfer.nmr.mgh.harvard.edu/fswiki/ReleaseNotes). These version changes may affect certain extracted measures. Gronenschild et al. (2012) compared volumes and cortical thickness measures across FreeSurfer v4.3.1, v4.5.0, and v5.0.0 and found many measurements differed significantly. After the release of the next version, v5.3, Dickie et al. (2017) performed correlation analysis between cortical thickness measures output from FreeSurfer v5.1 and v5.3, and found high compatibility between the two versions. Such work helped inform protocols for consortia such as ENIGMA, where groups that had run FreeSurfer versions older than v5.0, were asked to rerun their processing pipeline, whereas both v5.1 and v5.3 were used for analyses within certain working groups. A more recent study, Bigler et al. (2020), compared FreeSurfer v5.3 and v6.0 across a select set of volumes, finding low compatibility between versions for the volume of the globus pallidus.

The latest stable release, v7.1, has yet to be thoroughly assessed for intraversion reliability and between‐version compatibility. Here, we assessed the reliability and compatibility of the last three stable FreeSurfer version releases—v5.3 (2013), v6.0 (2017), and v7.1 (2020)—across three publicly available test–retest data sets. We set out to determine the (1) between‐version compatibility and (2) within‐version reliability, for cortical thickness, surface area, and subcortical volumes. We also perform a replication analysis using an independent data set and test how batch correction using a mixture of versions affects age associations in these test–retest data sets. To further test how these version differences may influence population‐level findings, we ran all three FreeSurfer versions on a subset of cross‐sectional data from the UK Biobank, a cohort of middle‐aged to older adults. We visually quality controlled and computed Dice overlap scores between each pair of versions for all regional outputs. Finally, we determined the linear effect of age for each region and metric of interest, to understand the stability of this effect across software versions.

## 2 METHODS

### 2.1 Data sets

Test–retest data sets from the Human Connectome Project (HCP) (Van Essen et al., 2013), Kennedy Krieger Institute (KKI) (Landman et al., 2010), and Open Access Series of Imaging Studies (OASIS‐1) (Marcus et al., 2007) were used to assess reliability within and between FreeSurfer versions. We limited the analysis to 76 healthy individuals with T1‐weighted brain MRI scans aged 19–61. KKI includes test‐retest data from 21 healthy volunteers with no history of neurological conditions; a test‐retest subset of 35 healthy young adults was provided by HCP, and OASIS‐1 includes 20 nondemented subjects imaged twice. The maximum interscan interval of 11 months in the HCP data set is longer than OASIS and KKI, yet we do not suspect considerable changes in brain structure between sessions given that HCP is comprised of generally healthy young adults between the ages of 22 and 35 years (Van Essen et al., 2013). See Table 1 for more details.

**TABLE 1:** Cohort demographics and scan parameters for test–retest data sets analyzed. HCP is a family‐based data set including up to four individuals per family, so we limited our ICC investigations to one randomly chosen individual per family. *indicates the maximum duration between any two consecutive scans; the maximum duration between the baseline scan and the final retest is 40 days.

Cohort	Age range; mean (SD)	No. subjects (%F)	Maximum interscan interval in days (mean)	Manufacturer/field strength	Voxel size (mm)^3^
HCP	22–35; 30.7 (2.97)	35 (44%)	330 (144)	Siemens 3 T	(0.7 × 0.7 × 0.7)
KKI	22–61; 31.8 (9.47)	21 (48%)	14	Philips 3 T	(1 × 1 × 1.2)
OASIS	19–34; 23.4 (4.03)	20 (60%)	90 (20.6)	Siemens 1.5 T	(1.0 × 1.0 × 1.25)
HNU (replication)	20–30; 24.4 (2.41)	30 (50%)	10 (3.7)*	GE 3 T	(1.0 × 1.0 × 1.0)

Abbreviations: HCP, Human Connectome Project; HNU, Hangzhou Normal University; ICC, intraclass correlation coefficient; KKI, Kennedy Krieger Institute; OASIS, Open Access Series of Imaging Studies.

A subset of 106 neurologically normal individuals was selected at random from the UK Biobank (Miller et al., 2016) to test age association outcome differences between versions. This included 56 females with a mean age and standard deviation of 62.3 (7.2) years and 50 males with a mean age and standard deviation of 61.2 (7.7) years. The age ranged from 46 to 78 years of age. In this case, being neurologically normal was defined based on the following exclusion criteria: cancers of the nervous system, diseases of the nervous system, aortic valve diseases, head injuries, and schizophrenia/bipolar disorders; as a large number of individuals had either anxiety or depressive episodes, the entire mental disorders category was not excluded. While the UK Biobank has over 40,000 individual scans, we selected a relatively small subset, with a sample size more in line with most single‐site current neuroimaging studies.

### 2.2 FreeSurfer regions and metrics of interest

All scans were run through the same *recon‐all* pipeline provided by FreeSurfer for stable v5.3, v6.0, and v7.1 releases on the USC Mark and Mary Stevens Neuroimaging and Informatics Institute's high performance computing cluster using a Linux‐centos6 operating system, ensuring the same OS and environment. For runtimes, please see supplementary Table S1. Cortical parcellations were computed based on the Desikan‐Killiany (DK) atlas (Desikan et al., 2006), where 34 distinct regions on each cortical hemisphere are labeled according to the gyral patterns. For each cortical region, FreeSurfer outputs the average cortical thickness, surface area, and volume. We focus our analyses on cortical thickness and surface area, as these are largely independent measures (Winkler et al., 2010) and volume is a composite of the two. We also extract and evaluate the FreeSurfer derived measures of total intracranial volume (ICV) and volumes of eight subcortical regions: the nucleus accumbens, amygdala, caudate, hippocampus, lateral ventricle, pallidum, putamen, and the thalamus. These metrics are all ones that have been repeatedly used throughout multinational ENIGMA projects, and are therefore of particular interest to many collaborative investigators invested in reproducible findings. For all of our intraclass correlation coefficient (ICC) analysis here, we report left and right measures, as well as average cortical thickness, total surface area, and average subcortical volumes. We also include hemisphere and whole brain cortical thickness and surface area. Euler values from the test–retest data sets for the final surfaces, as well as the surfaces before topological defect correction, are made available in the supplementary materials (Table S2).

### 2.3 Statistics and quality control

ICCs were calculated using the *psych* library in R (https://CRAN.R-project.org/package=psych). The following three compatibility comparisons were evaluated: v7.1 versus v6.0, v7.1 versus v5.3, and v6.0 versus v5.3. Only the first time points from the test–retest data were selected for these comparisons. ICC2 was used to compute between‐version compatibility measures to account for any systematic errors using the following formula:

$$\mathrm{ICC}2=\frac{\mathrm{BMS}-\mathrm{EMS}}{\mathrm{BMS}+\left(k-1\right)\mathrm{EMS}+k\left(\mathrm{JMS}-\mathrm{EMS}\right)/n^{\prime }}$$

where *BMS* is the between‐targets mean square, *EMS* is the residual mean square, *k* is the number of judges, *JMS* is the between‐judges mean square, and *n*′ is the number of targets (in our context, the judges would correspond to different software versions used to compute the measures).

Within‐version reliability measures were performed on within‐subject test–retest data for FreeSurfer versions v7.1, v6.0, and v5.3. ICC3 was used to measure within‐version reliability using the following formula:

$$\mathrm{ICC}3=\frac{\mathrm{BMS}-\mathrm{EMS}}{\mathrm{BMS}+\left(k-1\right)\mathrm{EMS}}$$

where *BMS* is the between‐targets mean square, *EMS* is the residual mean square, and *k* is the number of judges. ICCs were computed for each site and a weighted average was also computed, where the reported ICC2 and ICC3 measures represent a weighted average to account for the number of participants in each data set. ICC interpretation was based on Koo and Li (2016): ICCs < 0.50 are considered poor; between 0.50 and 0.75 are moderate, between 0.75 and 0.90 denote good agreement; and values greater than 0.90 indicate excellent reliability.

To test if FreeSurfer version affects population level findings in studies of modest sample size, age associations were performed in a cross‐sectional subset of the UK Biobank using linear regressions. Sex was used as a covariate; ICV was added as a covariate for subcortical volumes. In that same subset, detailed QC was performed using the ENIGMA QC protocol (http://enigma.ini.usc.edu/protocols/imaging-protocols/) to test differences in regional fail rates across the versions. Then, 54 subjects were assigned to rater #1 and 52 to rater #2. Each rater QC'ed the same subset across all three versions. Rater #3 then reviewed all QC fails for consistency. All subcortical QC was performed by rater #3 where a fail constitutes any notable overestimation or underestimation of volume for any structure. Age associations were also performed in this QC'ed subset, where subjects were excluded if the QC of any ROI was inconsistent across versions. If subjects had consistent regional fails, they were kept in the analysis, but those regions were excluded. While many studies of such sample size may perform manual segmentation corrections, there is no way to ensure consistent manual editing across the outputs of all software versions. We therefore opted to exclude QC fails to ensure our reported differences were due to changes in software version.

For each set of regressions within a version, statistical significance was determined after controlling the false discovery rate (FDR) at *q* < 0.05 across 234 measures, which included all bilateral, unilateral, and full brain measures. FDR (Benjamini & Hochberg, 1995) corrected *p*‐values and z‐statistics were plotted on brain surfaces for comparison. All values, including uncorrected *p*‐values, are tabulated on our web‐viewer. Dice coefficients (Dice, 1945) were also calculated in the UK Biobank subset to assess the extent of spatial overlap of ROIs across versions, for all regions in the DK atlas.

### 2.4 Replication analysis

To ensure replicability of our results, we calculated reliability and compatibility measures on the Hangzhou Normal University (HNU) cohort. This data set is a valuable resource to assess reliability with its 10 test–retest design (Zuo et al., 2014): 30 participants were scanned 10 times, all within 40 days of their baseline scan with a mean of 3.7 days between two consecutive scans (see Table 1 for more details). Upon visual inspection of the FreeSurfer outputs, we excluded three subjects (subject IDs: 25434, 25440, 25438) due to an error in the brain extraction that cut off a superior segment of the brain in at least one of that subject's sessions. Out of 300 scans, we note that this was observed three times in v5.3, two times in v6.0, and four times in v7.1. Radar plots of ICCs are available in the supplementary materials (Figures S7 and S8).

### 2.5 ComBat analysis

To test the effects of batch correction, we performed an additional set of age associations on all the test–retest data sets—harmonizing for site using ComBat (Fortin et al., 2018). We limited our analysis to the first timepoint with a max age of 35 years old as the KKI data set only had a few participants with ages beyond this point. Here, we used all subjects from HNU given that errors in baseline scans were not observed. We compare harmonized v7.1 results to a mixture of v7.1 and other versions, where we change the version of one (75% v7.1) or two data sets (50% v7.1) to v5.3 or v6.0. Differences in bilateral significant z‐statistics before and after FDR correction are available in the supplementary materials (Figures S11–S14).

## 3 RESULTS

The full set of our reliability, compatibility, and association results are available through an interactive 3D brain viewer here: http://data.brainescience.org/ Freesurfer_Reliability /. Cohort specific ICCs and associated statistics are also available in the supplementary material (Figures S1–S8, Tables S3–S6).

### 3.1 Between version compatibility

**FIGURE 1:**
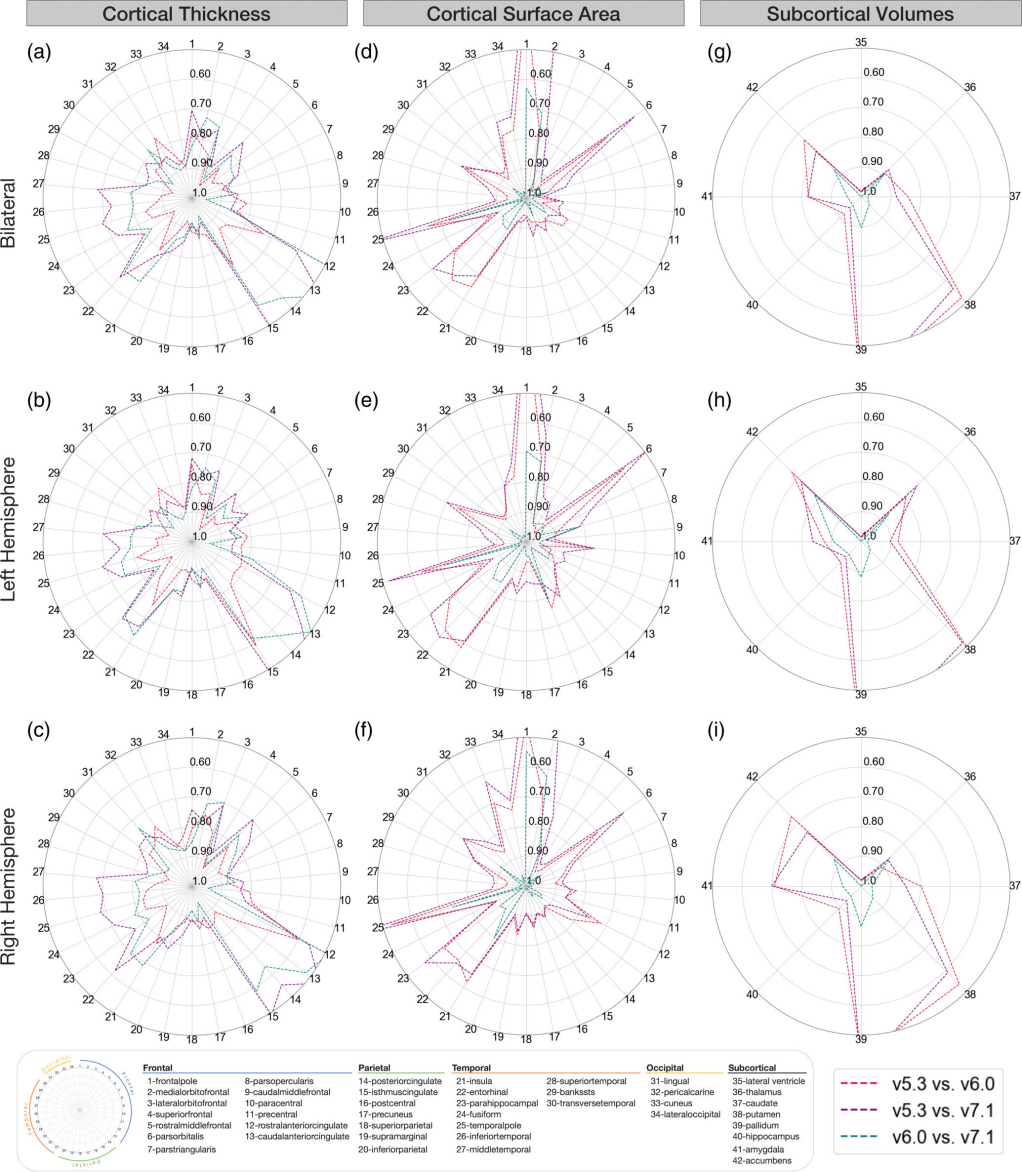
Regional interversion agreement (compatibility; estimated by ICC2). Bilateral, left, and right ICC2 values comparing cortical thickness (a–c), cortical surface area (d–f), and subcortical volumes (g–i) between versions. Outer concentric circles represent lower ICC2 values, truncated at 0.50, while the center represents ICC2 = 1. Regions with the lowest compatibility differ for cortical thickness and surface area. These compatibility estimates shown are a sample‐size weighted average of results in each of Human Connectome Project (HCP), Kennedy Krieger Institute (KKI), and Open Access Series of Imaging Studies (OASIS) data sets.

**FIGURE 2:**
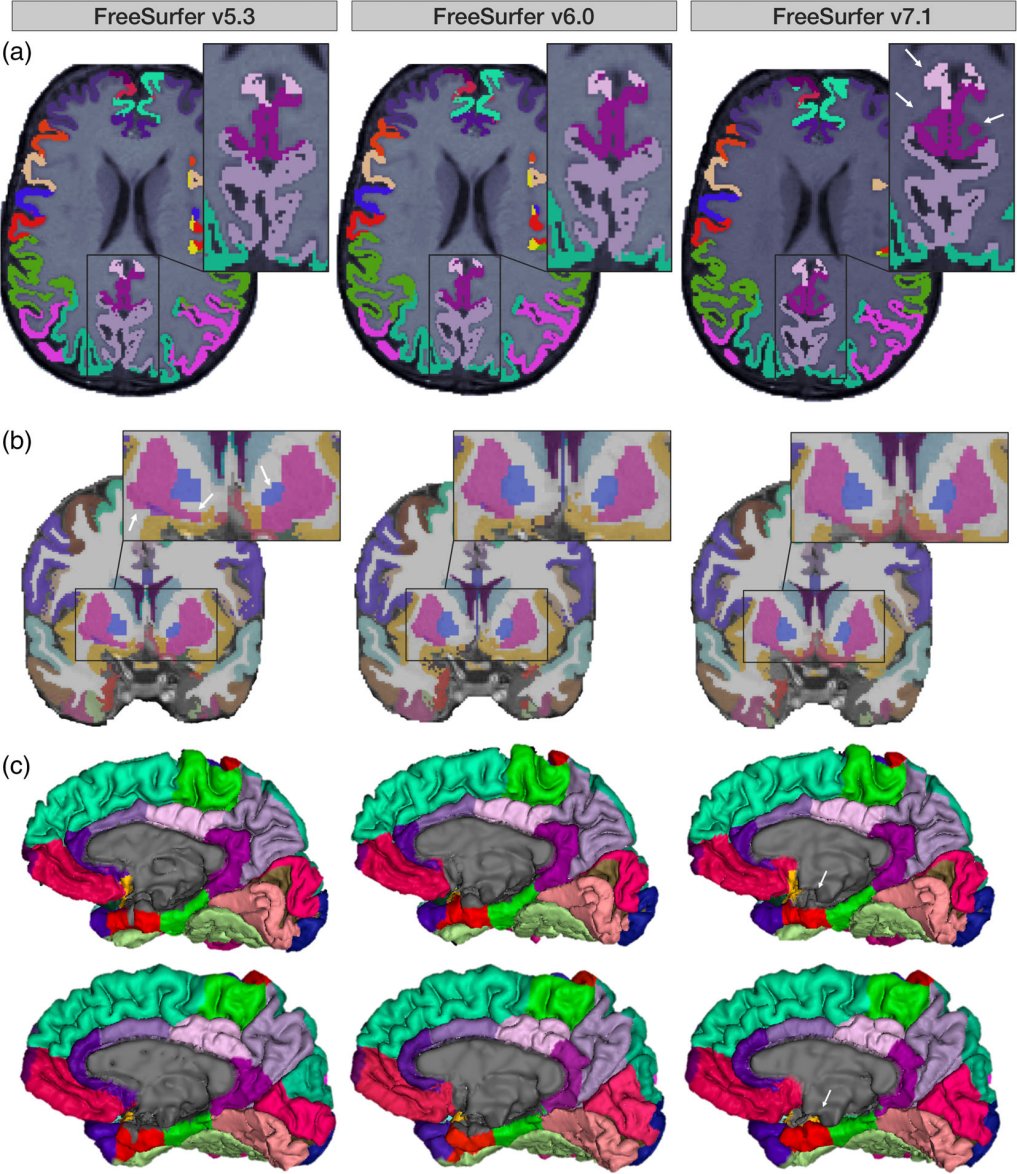
(a) Axial slices from the same UK Biobank participant across versions. Arrows indicate posterior and isthmus cingulate differences in v7.1 versus v5.3 and v6.0. (b) Coronal slices from the same subject across versions. Arrows demonstrate v5.3 volume differences in the putamen and pallidum versus v6.0 and v7.1. (c) Medial surface representations of two UK Biobank participants across versions. Arrows highlight differences in the medial wall pinning, particularly in the entorhinal cortex, in v7.1 compared to the two prior releases.

Version compatibility results between FreeSurfer v5.3, v6.0, and v7.1 for all cortical and subcortical metrics are shown in Figure 1. Overall, the version compatibility across all versions for average cortical thickness was good to excellent (ICC_v7.1:v6.0_ = 0.81; ICC_v7.1:v5.3_ = 0.85; ICC_v6.0:v5.3_ = 0.91). Similarly, left and right hemispheric thicknesses were good for v7.1 comparisons (left: ICC_v7.1:v6.0_ = 0.80, ICC_v7.1:v5.3_ = 0.86; right: ICC_v7.1:v6.0_ = 0.81, ICC_v7.1:v5.3_ = 0.83), and excellent when comparing v6.0 to v5.3 (left: ICC_v6.0:v5.3_ = 0.91; right: ICC_v6.0:v5.3_ = 0.90). Furthermore, version compatibility was excellent for v7.1 versus v6.0 in several bilateral regional parcellations including the paracentral, postcentral, superior frontal, transverse temporal, and superior parietal cortices (ICC_v7.1:v6.0_ > 0.90). The postcentral (ICC_v7.1:v5.3_ = 0.91) and superior parietal (ICC_v7.1:v5.3_ = 0.91) gyri also showed excellent compatibility between v7.1 and v5.3. Additionally, v6.0 was highly compatible with v5.3 in the superior frontal, superior temporal, parahippocampal, supramarginal, *pars orbitalis*, and the banks of the superior temporal sulcus (ICC_v6.0:v5.3_ ≥ 0.90). Several bilateral regions showed poor compatibility between v7.1 and older versions, however. In particular, the lowest ICCs were found for the isthmus (ICC_v7.1:v5.3_ = 0.37; ICC_v7.1:v6.0_ = 0.58), posterior (ICC_v7.1:v5.3_ = 0.41; ICC_v7.1:v6.0_ = 0.55), caudal anterior (ICC_v7.1:v5.3_ = 0.46; ICC_v7.1:v6.0_ = 0.45), and rostral anterior (ICC_v7.1:v5.3_ = 0.61; ICC_v7.1:v6.0_ = 0.50) subregions of the cingulate gyrus. An example subject with notable differences in cingulate segmentations is displayed in Figure 2a. Other regions that showed moderate agreement with v7.1 and either v6.0 or v5.3 included the entorhinal (ICC_v7.1:v5.3_ = 0.64; ICC_v7.1:v6.0_ = 0.67), middle temporal (ICC_v7.1:v5.3_ = 0.68), and insular (ICC_v7.1:v6.0_ = 0.67) cortices, as well as the temporal (ICC_v7.1:v5.3_ = 0.69) and frontal poles (ICC_v7.1:v5.3_ = 0.70; Figure 1a).

Total surface area showed excellent compatibility across all three versions (ICC_v7.1:v6.0_ = 0.99; ICC_v7.1:v5.3_ = 0.96; ICC_v6.0:v5.3_ = 0.99). Left and right hemispheric surface area compatibility between versions were also excellent across all comparisons (ICCs > 0.96). Overall, the two most compatible versions were v7.1 versus v6.0, where, notably, 29/34 bilateral regions had ICCs > 0.90. Several regions also showed excellent compatibility (ICC > 0.90) across all three version comparisons: these included the caudal middle frontal, the inferior parietal, postcentral, posterior cingulate, rostral middle frontal, superior parietal, and the supramarginal gyri. However, we did find surface area compatibility discrepancies not only in regions mostly distinct from cortical thickness, but also between the pairs of versions being compared as well. The lowest bilateral regional surface area compatibility ICCs were observed in frontal and temporal areas when comparing newer versions to v5.3, where v7.1 showed lower compatibility to v5.3 than to v6.0. Frontal regions included the medial orbitofrontal cortex (ICC_v7.1:v5.3_ = 0.51; ICC_v6.0:v5.3_ = 0.76), *pars orbitalis* (ICC_v7.1:v5.3_ = 0.54; ICC_v6.0:v5.3_ = 0.66), and the frontal poles which were not compatible between either v7.1 (ICC_v7.1:v5.3_ = 0.19) or v6.0 (ICC_v6.0:v5.3_ = 0.32). However, compatibility between v7.1 and v6.0 was moderate for the medial orbitofrontal cortex (ICC_v7.1:v6.0_ = 0.71), excellent for the *pars orbitalis* (ICC_v7.1:v6.0_ = 0.94), and moderate for the frontal pole (ICC_v7.1:v6.0_ = 0.63). Temporal regions that followed similar trends included the parahippocampal gyrus (ICC_v7.1:v5.3_ = 0.61; ICC_v6.0:v5.3_ = 0.70) and the temporal poles (ICC_v7.1:v5.3_ = 0.43; ICC_v6.0:v5.3_ = 0.66). In contrast, v7.1 has excellent compatibility with v6.0 for the parahippocampal gyrus (ICC_v7.1:v6.0_ = 0.90) and moderate compatibility for the temporal pole (ICC_v7.1:v6.0_ = 0.73; Figure 1d).

ICV was highly compatible across all versions (ICCs > 0.97). All bilateral subcortical volumes showed good to excellent compatibility when comparing v7.1 to v6.0 (ICCs > 0.87). Good to excellent compatibility was also found comparing v5.3 to the newer versions in the lateral ventricle, hippocampus, thalamus, caudate, and amygdala (ICCs > 0.82). Compatibility issues arose when comparing v7.1 and v6.0 against v5.3. Poor to moderate regional compatibility was found in the pallidum (ICC_v7.1:v5.3_ = 0.34; ICC_v6.0:v5.3_ = 0.36), putamen (ICC_v7.1:v5.3_ = 0.56; ICC_v6.0:v5.3_ = 0.52; Figure 2b), and to a lesser extent, the nucleus accumbens (ICC_v7.1:v5.3_ = 0.78; ICC_v6.0:v5.3_ = 0.73; Figure 1g).

Replication analysis using the HNU data set showed the compatibility of surface area and subcortical volumes to be largely in line with our main analysis (Figure S7d,g). Whereas in our main analysis, we show the most discrepancy between cortical thickness measures from v7.1 and the previous versions, mostly in the cingulate regions, our replication analysis showed lower compatibility more broadly between cortical thickness from v5.3 and those from newer versions (Figure S7a).

### 3.2 Within‐version reliability

**FIGURE 3:**
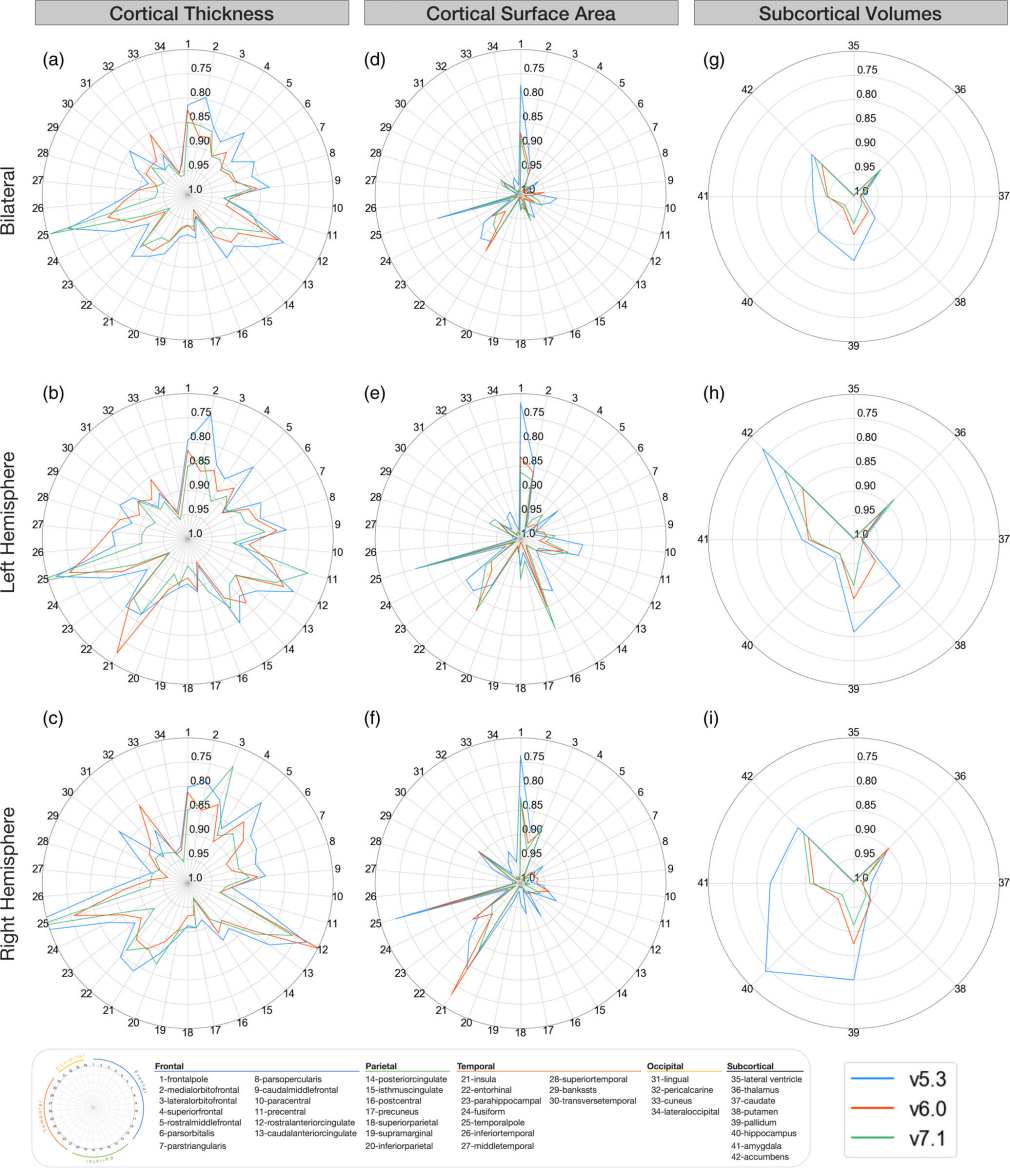
Regional intraversion agreement (reliability; estimated by ICC3). Bilateral, left, and right ICC3 values comparing cortical thickness (a–c), cortical surface area (d–f), and subcortical volumes (g–i) between versions. Outer concentric circles represent smaller ICC3 values, truncated at 0.70, while the center represents ICC3 = 1. Regions with the lowest reliability differ for cortical thickness and surface area. These reliability estimates shown are a sample‐size weighted average of results in each of Human Connectome Project (HCP), Kennedy Krieger Institute (KKI), and Open Access Series of Imaging Studies (OASIS) data sets.

The meta‐analyzed scan‐rescan reliability for all cortical and subcortical metrics within each of FreeSurfer v5.3, v6.0, and v7.1 are shown in Figure 3. All versions showed high reliability for average bilateral, left hemispheric, and right hemispheric cortical thickness (ICC > 0.90). Regional bilateral metrics with the lowest thickness ICCs—but still considered moderate to good—included the temporal pole (ICC_v7.1_ = 0.71; ICC_v6.0_ = 0.83; ICC_v5.3_ = 0.74), rostral anterior cingulate (ICC_v7.1_ = 0.83; ICC_v6.0_ = 0.79; ICC_v5.3_ = 0.78), and the medial orbitofrontal cortex (ICC_v7.1_ = 0.85; ICC_v6.0_ = 0.88; ICC_v5.3_ = 0.80; Figure 3a). Total bilateral, left hemispheric, and right hemispheric surface area reliability was also high (ICC = 0.99) for all three FreeSurfer versions. The regions with the lowest surface area ICCs were all still highly reliable, but included the frontal poles (ICC_v7.1_ = 0.88; ICC_v6.0_ = 0.87; ICC_v5.3_ = 0.77), insula (ICC_v7.1_ = 0.91; ICC_v6.0_ = 0.86; ICC_v5.3_ = 0.89), and entorhinal cortex (ICC_v7.1_ = 0.92; ICC_v6.0_ = 0.95; ICC_v5.3_ = 0.88) (Figure 3d). Regional bilateral subcortical volumes were all reliable for each of the three versions (ICC > 0.86; Figure 3g). ICV reliability was also very high (ICC > 0.97) for all versions.

Replication analysis using the HNU data set showed the reliability of surface area and most subcortical volumes to be largely in line with our main analysis—similar to compatibility (Figure S8d,g). The reliability of the accumbens, amygdala, and hippocampus in v5.3 showed the most discrepancy from the main analysis with lower ICCs in the replication data set. Cortical thickness reliability showed more widespread and lower reliability overall, across all versions. Regions that were classified as having moderate to poor reliability in HNU included the temporal pole, insula, entorhinal, inferior temporal, rostral anterior cingulate, medial orbitofrontal, and lateral orbitofrontal cortices (Figure S8a).

### 3.3 ComBat analysis

When comparing multisite age association results of harmonized v7.1 measures to a combination of either harmonized v7.1 and v6.0 or harmonized v7.1 and v5.3 measures, we find results to be similar, but not identical. When we harmonize a single cohort's v6.0 or v5.3 measures in combination with v7.1, we find that all measures that were considered statistically significant after FDR correction in the harmonized v7.1 analysis were also significant in the analyses for which we swapped out a single cohort's measures to be from v6.0 or v5.3 (Figures S11 and S12). The only exception was the thickness of the banks of the superior temporal sulcus, which was not significant using HCP's v6.0 values in the harmonization, but was when using all harmonized v7.1 and any of the KKI, OASIS, or HNU v6.0 swaps in combination with v7.1. We find some regions were not significant in the harmonized v7.1 alone analysis, but were significant when using a single cohort's v5.3 or v6.0 results. For example, the thickness of the precuneus was not significantly associated with age when using only v7.1 harmonized measures, but was when swapping HCP and OASIS measures for those of v5.3 and for swapping OASIS and HNU measures for those of v6.0. When we harmonize the measures of half the cohorts with v7.1 and half the cohorts with v5.3, we no longer find the same age associations as in v7.1 for the banks of the superior temporal sulcus thickness and the posterior cingulate surface area. When we harmonize with half the cohorts run through v6.0, again the banks of the superior temporal sulcus banks did not always show a significant association. Similar to the single cohort swaps, we see regions that are significantly associated with age in the mixtures but not in the v7.1 only analysis. In addition to the precuneus surface area in both v5.3 and v6.0, we also see associations with the pars opercularis and transverse temporal surface areas in combinations that included either v5.3 or v6.0, and postcentral surface area associations for combinations with v6.0. All results, including uncorrected associations are available in the supplementary materials (Figures S11–S14).

### 3.4 Quality control and population‐level analysis

**FIGURE 4:**
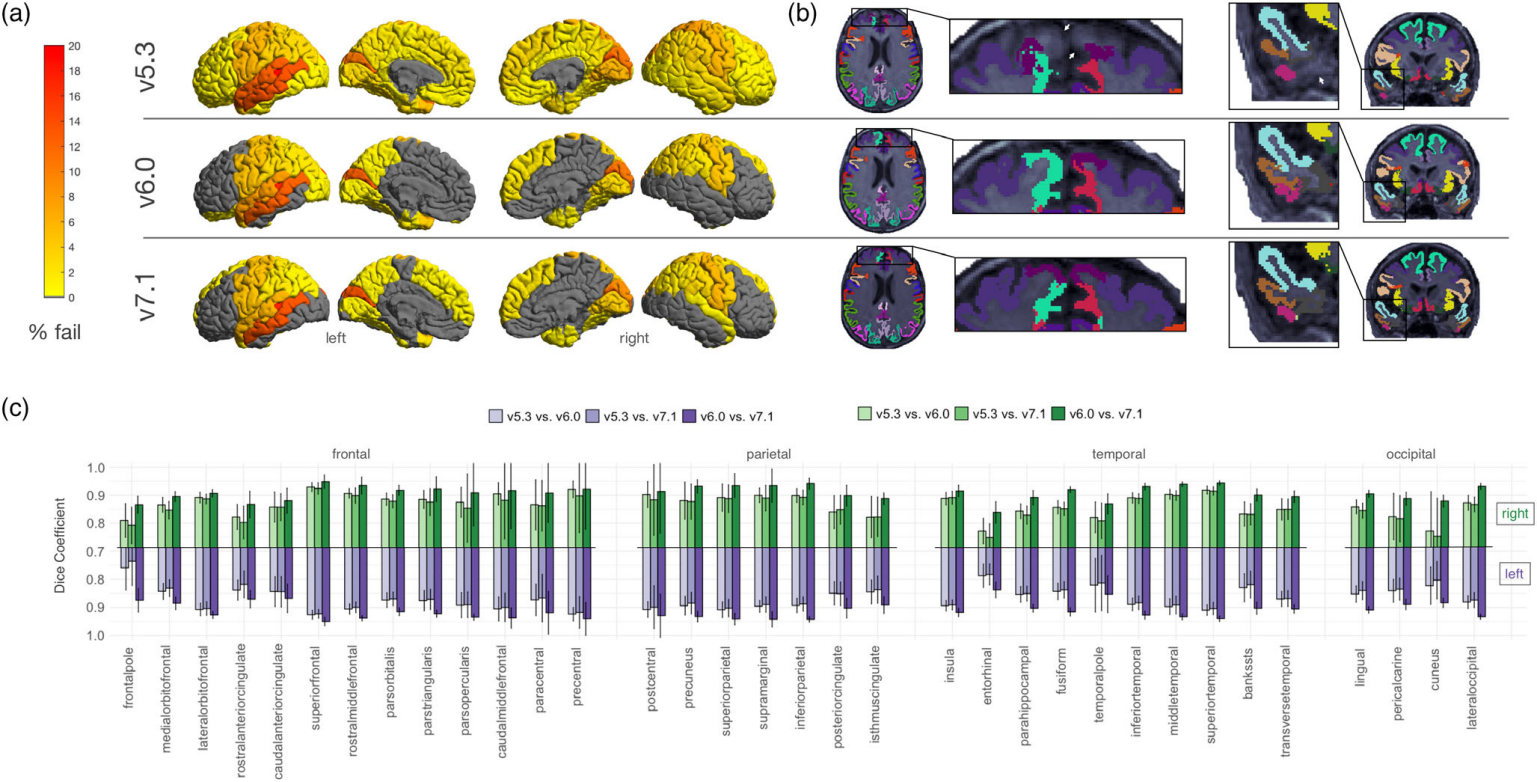
Cortical quality control results. Results based on 106 neurologically healthy UK Biobank participants. (a) Manual cortical quality control results (percentage fail) based on the Enhancing NeuroImaging Genetics through Meta‐Analysis (ENIGMA) quality control (QC) protocol across versions. Gray regions indicate no failures. Note more widespread failures particularly in the temporal and frontal regions due to a single subject (representative failure case) in (b). We also note generally higher rates of failure in the left temporal lobes across all versions. (c) Dice scores across left and right hemisphere Desikan‐Killiany atlas labels. We note the lowest overlap in the cuneus, entorhinal, pericalcarine, cingulate cortices, and temporal and frontal poles, particularly when comparing v5.3 to the newer versions.

Figure 4 highlights regional cortical quality issues noted in the subset of UK Biobank participant scans across each of the evaluated FreeSurfer versions. The region that showed the greatest difference in failure rate was the left superior temporal gyrus—where v7.1 performed the best (5.7% fails) followed by v6.0 (7.5% fails), and v5.3 performed the worst (12.3% fails; Figure 4a). In one subject with poor image quality, a general underestimation occurred throughout the brain in v5.3 but not in v6.0 and v7.1 (see Figure 4b). Other regions that failed at a relatively similar rate across all three versions included the left banks of the superior temporal sulcus (v7.1 = 17%; v6.0 = 17%; v5.3 = 18.9%), the left (v7.1 = 14.2%; v6.0 = 13.2%; v5.3 = 12.3%), and right (v7.1 = 11.3%; v6.0 = 12.3%; v5.3 = 13.2%) pericalcarine, the left middle temporal (v7.1 = 13.2%; v6.0 = 12.3%; v5.3 = 13.2%), the left cuneus (v7.1 = 13.2%; v6.0 = 12.3%; v5.3 = 10.4%), and the right cuneus (v7.1 = 8.5%; v6.0 = 10.4%; v5.3 = 10.4%).

The highest overlap was between v7.1 and v6.0, where most regions had a Dice coefficient of 0.90 or greater. The lowest overlap occurred when comparing v5.3 to both v7.1 and v6.0, particularly in the frontal pole (left: DC_v7.1:v5.3_ = 0.74, DC_v6.0:v5.3_ = 0.76; right: DC_v7.1:v5.3_ = 0.79, DC_v6.0:v5.3_ = 0.81), entorhinal (left: DC_v7.1:v5.3_ = 0.78, DC_v6.0:v5.3_ = 0.79; right: DC_v7.1:v5.3_ = 0.75, DC_v6.0:v5.3_ = 0.77), and right cuneus (DC_v7.1:v5.3_ = 0.75, DC_v6.0:v5.3_ = 0.77). Other regions with lower Dice coefficients were the cingulate regions, temporal pole, pericalcarine, and the banks of the superior temporal sulcus (Figure 4c).

**FIGURE 5:**
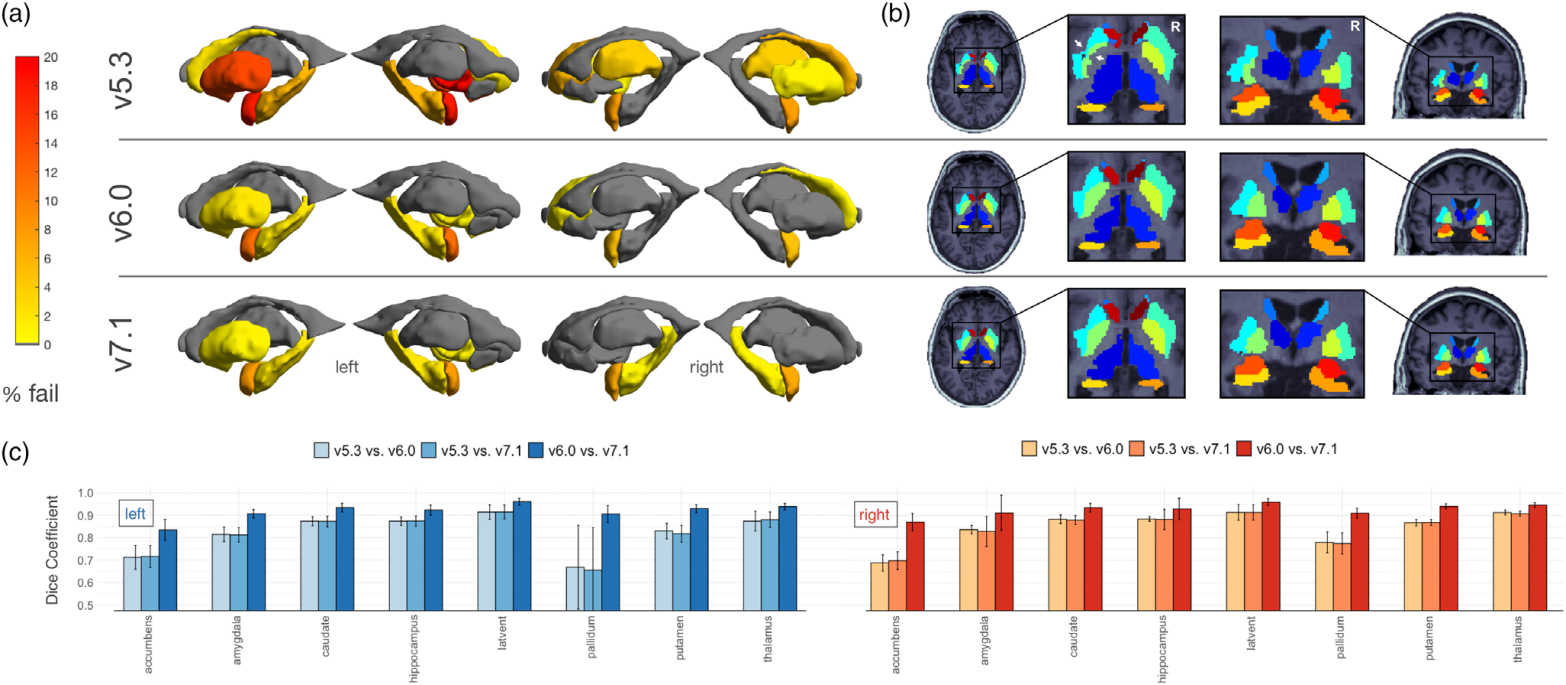
Subcortical quality control results. Results based on 106 neurologically healthy UK Biobank participants. (a) Manual subcortical quality control results (percentage fail) across versions. Gray regions indicate no failures. Note generally higher fail rates in the left hemisphere and when comparing v5.3 to the newer versions. (b) Example subcortical outputs. Arrows indicate the left putamen (cyan) and pallidum (light green) mis‐segmentation in v5.3. (c) Dice scores across left and right hemisphere subcortical regions. Note the lowest overlap when comparing v5.3 to v6.0 and v7.1.

Figure 5 highlights the subcortical quality issues noted across each of the evaluated FreeSurfer versions. The most regional failures were detected in v5.3. Failures occurred more often in the left hemisphere (Figure 5a). The most notable differences in failure rates were for the left pallidum (v7.1 = 0.9%; v6.0 = 0.9%; v5.3 = 18.9%), left amygdala (v7.1 = 7.5%; v6.0 = 11.3%; v5.3 = 17.9%), and left putamen (v7.1 = 0.9%; v6.0 = 1.9%; v5.3 = 14.2%). Example outputs may be viewed in Figure 5b. The regions with the lowest overlap were in the left and right pallidum (left: DC_v7.1:v5.3_ = 0.66, DC_v6.0:v5.3_ = 0.67; right: DC_v7.1:v5.3_ = 0.78, DC_v6.0:v5.3_ = 0.78) as well as the left and right nucleus accumbens (left: DC_v7.1:v5.3_ = 0.72, DC_v6.0:v5.3_ = 0.71; right: DC_v7.1:v5.3_ = 0.70, DC_v6.0:v5.3_ = 0.69) when comparing v5.3 to both newer versions. Notably, the segmentation of the left putamen often appeared larger and the left pallidum smaller in v5.3 compared to the newer versions (Figure 5b).

**FIGURE 6:**
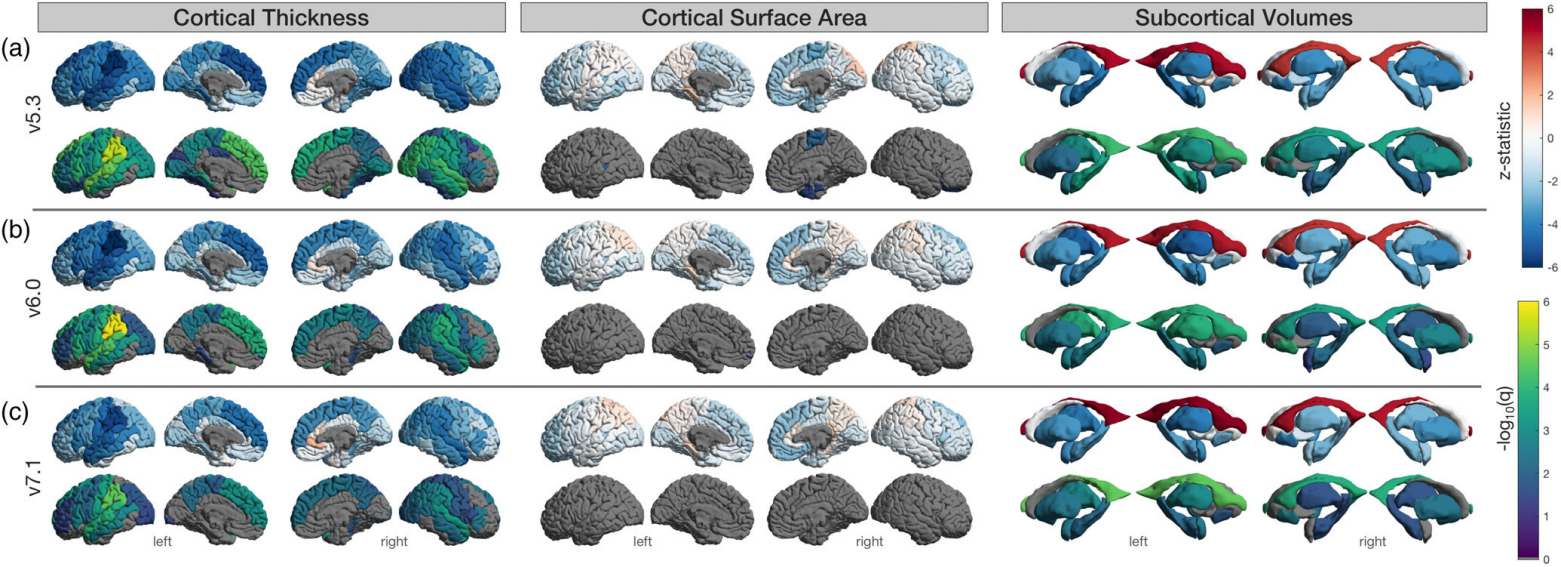
Regional age associations in all subjects. Results based on 106 neurologically healthy UK Biobank participants. (a) FreeSurfer v5.3, (b) v6.0, and (c) v7.1. Top row indicates the *z*‐statistic and bottom indicates −log_10_(*q* < 0.05) for left and right cortical thickness, surface area, and subcortical volumes. We note that v5.3 generally has the largest absolute *z*‐statistics, particularly for cortical thickness, and the largest number of statistically significant regions.

**FIGURE 7:**
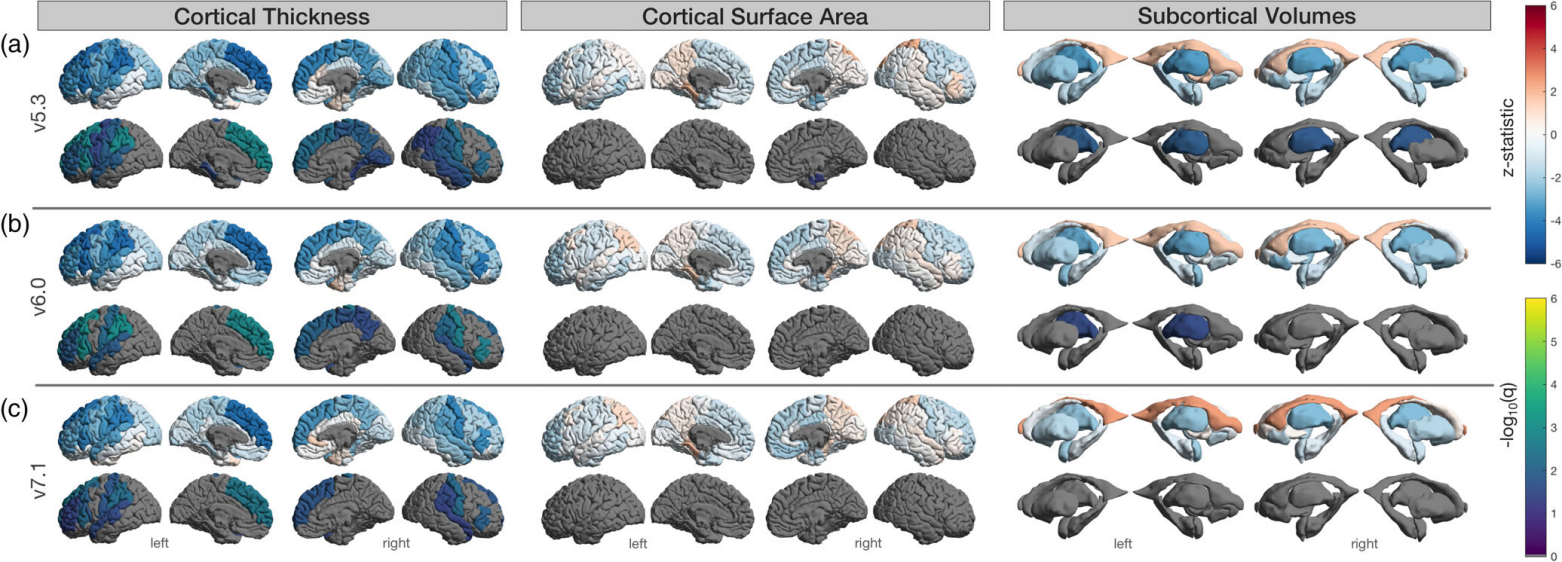
Regional age associations in subjects with no segmentation quality issues. Results based on *n* = 69 (cortical) and 61 (subcortical) of the 106 neurologically healthy UK Biobank participants. (a) FreeSurfer v5.3, (b) v6.0, and (c) v7.1. Top row indicates the *z*‐statistic and bottom indicates −log_10_(*q* < 0.05) for left and right surface area, thickness, and subcortical volumes. Several regions found to be significant in the full sample of *n* = 106 did not survive false discovery rate (FDR) correction here.

Age associations are shown in Figures 6 and 7. Maps of the z‐statistic differences are also made available in the supplementary materials (Figures S9 and S10). In the full set (106 UK Biobank scans) age associations for cortical thickness (Figure 6), v7.1 had 29 regions that survived FDR correction, less than both v6.0 with 32 and v5.3 with 43; all these regions showed lower thickness with age other than the right rostral anterior cingulate, which showed a positive association with age across all versions. The strongest associations were in the left supramarginal (*z*_v7.1_ = −5.55, *q*_v7.1_ = 3 × 10^−5^; *z*_v6.0_ = −6.24, *q*_v6.0_ = 1 × 10^−6^; *z*_v5.3_ = −5.90, *q*_v5.3_ = 4 × 10^−6^) and left superior temporal gyrus (*z*_v7.1_ = −4.88, *q*_v7.1_ = 1 × 10^−4^; *z*_v6.0_ = −5.21, *q*_v6.0_ = 4 × 10^−5^; *z*_v5.3_ = −5.33, *q*_v5.3_ = 2 × 10^−5^) for all three versions. All regions that were significant in v7.1 and v6.0 were also significant in v5.3, except for the left frontal pole in v6.0 (*z*_v7.1_ = −2.19, *q*_v7.1_ = 9 × 10^−2^; *z*_v6.0_ = −2.55, *q*_v6.0_ = 4 × 10^−2^; *z*_v5.3_ = −1.83, *q*_v5.3_ = 1 × 10^−1^). Generally, v5.3 had the largest absolute *z*‐statistics compared to v7.1 and v6.0. For the surface area age associations, no regions survived FDR correction in v7.1, whereas in v6.0, the left frontal pole survived correction, and in v5.3, the right paracentral, left banks of the superior temporal sulcus, right entorhinal, right lateral orbitofrontal, and right temporal pole were considered significantly associated with age after correction. For subcortical volumes, all regions were significantly associated with age, except for the left and right caudate and pallidum for all three versions and the right amygdala for v7.1 (*q*_v7.1_ = 0.08).

A total of 69 subjects remained for regression analysis in the cortical QC'ed subset (37F, mean age: 61.1 ± 7.11). In this subset, cortical thickness was associated with age in 13 regions for v7.1, 16 for v6.0, and 22 for v5.3 (Figure 7). As with the full set above, all regions that survived FDR correction in v7.1 also survived in v6.0 and v5.3 and all regions that survived in v6.0 were also significant in v5.3. Cortical thickness regions that had a considerable proportion of fails and no longer reached the significance threshold in the QC'ed subset included the left banks of the superior temporal sulcus, left middle temporal, right precentral, and the right superior parietal gyrus. The left lingual, left cuneus, right pericalcarine, and the right banks of the superior temporal sulcus were all regions that had considerable quality issues and for which cortical thickness associations met FDR significance criteria for v5.3 in the full subset, yet these thickness associations were no longer significant in the QC'ed subset. The only surviving region in the QC'ed subset for surface area was the right entorhinal cortex in v5.3, although it is worth noting this region was not heavily QC'ed. The external surface in this area was apparently different in v7.1 compared to the previous versions (Figure 2c) and the rate at which this occurred would have resulted in the majority of participants being considered as a “fail” in the older versions.

A total of 61 subjects (35F, mean age: 62.6 ± 6.8 years) were found to have no quality issues in the subcortical segmentations across any versions. Age associations with these subjects indicated that only the thalamic volumes were significantly associated with age in v5.3 (both right and left) and v6.0 (left only).

## 4 DISCUSSION

Our work has four main findings that may help explain how a FreeSurfer version upgrade can impact results:

1. The compatibility between v7.1 and the previous version, v6.0, was largely good to excellent for measures of cortical surface area and subcortical volume, with the exception of the medial orbitofrontal cortex and the frontal/temporal poles. Similar trends were observed in our replication analysis. Most compatibility issues arose in regional cortical thickness estimates, where moderate or even poor compatibility was seen in the thickness estimates of the cingulate gyrus (rostral anterior, caudal anterior, posterior, and isthmus), entorhinal, insula, and orbitofrontal regions (medial and lateral). In our replication analysis, mostly moderate compatibility was found in these regions. The exceptions were the entorhinal cortical thickness with poor compatibility and the thickness of the isthmus cingulate with good compatibility.
2. There were substantial compatibility issues between v7.1 and v5.3, in cortical regional thickness, area, and subcortical volumes. Thickness measures with low compatibility between v7.1 and v5.3 were the same as those between v7.1 and v6.0. However, the replication data set showed more similarities between the comparisons evaluating v5.3 against the newer versions. Regions with cortical surface area and subcortical volume compatibility issues between v7.1 and v5.3 were the same as the regions that were less compatible between v5.3 and v6.0, suggesting these area and volume differences were introduced with v6.0, not v7.1, which was in line with our replication analysis.
3. The test–retest reliability for all v7.1 metrics evaluated here was good to excellent in our main analysis, except for the thickness of the temporal pole. Replication analysis showed similar trends for surface area and subcortical volumes, but cortical thickness intraversion reliability was lower overall across all versions.
4. Age associations revealed generally smaller absolute *z*‐statistics in v7.1 compared to earlier releases, where v5.3 had the largest absolute *z*‐statistics overall. Quality issues were more prevalent in v5.3, particularly in the left superior temporal gyrus, pallidum, and putamen. Age associations did not meet the statistical significance threshold in many of the heavily quality controlled regions.

The regions in which v7.1 had the lowest compatibility with the previous versions were along the caudal‐rostral axis of cingulate cortex. The subdivisions of the cingulate cortex play distinct roles in large‐scale brain networks including the visceromotor, ventral salience, dorsal executive/salience, and default mode networks (Touroutoglou & Dickerson, 2019). Alterations in the subregions of the cingulate cortex have been demonstrated throughout the lifespan and in association with different neuropsychiatric disorders. For example, compared to controls, developmental delays in adolescents with attention deficit hyperactivity disorder are seen most prominently in the thickness of the prefrontal regions including the cingulate cortices (Vogt, 2019). In posttraumatic stress disorder (PTSD) studies, the anterior midcingulate, and in some cases the posterior cingulate, show, on average, lower thickness in individuals with PTSD compared to healthy controls (Hinojosa et al., 2019). Subregions of the cingulate cortex have also been associated with age related cognitive performance. In “SuperAgers,” or adults over the age of 80 years, whose episodic memory is resistant to age‐related decline, a preservation of the anterior cingulate thickness is observed (de Godoy et al., 2021; Gefen et al., 2015; Harrison et al., 2012; Harrison et al., 2018; Sun et al., 2016). Many of these studies were performed using versions of FreeSurfer that precede v7.1, so possible replication issues in future studies may be partially explained by the version incompatibility described in this work. Although we tested within a very narrow age range, and more extensive evaluation may be needed, we find that batch correction methods may adjust for these effects in the case where the large majority of the cohorts are run through the same version of FreeSurfer. By simulating a multi‐cohort analysis, where all but one of the cohorts have run v7.1 and one site has been run on a version that precedes v7.1, we find similar cortical thickness and surface area cingulate associations after multiple comparisons correction. However, prior to multiple comparisons correction, differences exist across the cohorts for both v6.0 and v5.3—suggesting that mixing versions could possibly result in false positives. Furthermore, as we also tested different age associations after ComBat harmonization across iterations with two cohorts run with v7.1, and two cohorts with other versions, we notice that as more cohorts are run with different FreeSurfer versions, ComBat harmonization is less effective and some regional variability in results, possibly false positives, may be introduced by using a mixture of versions. This analysis is limited in the number of combinations that were tested here, and additional extensive evaluation may be needed on a wider age‐range with more cohorts run across multiple versions.

Other regions with lower thickness compatibility with v7.1 included the medial and lateral orbitofrontal, entorhinal, and insular cortices. Inferior frontal regions such as the medial and lateral orbitofrontal cortices are often susceptible to signal loss and bias field inhomogeneities. v7.1 uses an updated bias field and denoising method that could affect the gray/white matter contrast in these areas. Temporal regions, such as the entorhinal and insular cortex, which were less compatible with v7.1, could be due to an algorithmic update that pins the pial surface in the medial wall to the white matter surface. This prevents a premature cutoff through the hippocampus and amygdala, which may affect surrounding regions in earlier versions. Notably, visual inspection of the external surface of the entorhinal cortex revealed an improvement of the entorhinal pinning to the medial wall in v7.1—as opposed to prior versions (Figure 2c). This issue was extremely prevalent, and considering these subjects as “QC‐fails” would have resulted in the majority of subjects failing; therefore, subject scans affected by this cutoff in v5.3 and v6.0 remained included in our “error‐free” subset. Downstream effects of this may be demonstrated in our age associations within the full *n* = 106 sample. Here, the left insular thickness showed significant age effects in v5.3 and v6.0, as well as the thickness of the right entorhinal cortex in v5.3, but neither showed associations with age in v7.1. The entorhinal cortex plays an important role in mediating information transfer between the hippocampus and the rest of the brain (Coutureau & Di Scala, 2009; Garcia & Buffalo, 2020). Measurements of its thickness are widely assessed in Alzheimer's disease, as it is one of the first regions to be impacted by the disease process (Braak & Braak, 1991) and researchers have found associations between its thickness and markers of amyloid and tau (Thaker et al., 2017). Entorhinal thickness is often a feature of interest in models that are designed to predict progressive cognitive decline due to its early vulnerability and role in the prodromal stages of Alzheimer's disease. Although v7.1 may have a more anatomically accurate segmentation, we advise caution when comparing the performance of predictive models that use earlier releases of FreeSurfer for deriving this metric.

Compatibility issues between v7.1 and older versions were less frequent with surface area and did not occur in the same regions as cortical thickness. This could be due to the relative independence of these measures: surface area is calculated as the area of all the triangles on the white matter surface, and the large area covered by many regions makes them more robust to slight variation in vertex counts. On the other hand, cortical thickness is measured as the distance between the vertices of the white matter and pial triangulated surfaces, and is often between 2 and 4 mm thick, a span of only two to four voxels; slight variability in partial voluming may have a more dramatic effect on cortical thickness. Yet, as the thickness is averaged in the entire area, a slight variation in the number of vertices on the surface will have little effect on the averaged cortical thickness estimates. The independence of these measures has also been established in relation to their genetic associations (Grasby et al., 2020; Winkler et al., 2010) overall suggesting that our results are not unexpected. Measures of v7.1 surface area that had poor compatibility with v5.3 (and moderate with v6.0) included the frontal and temporal poles. The release of v7.1 included a remeshing of the white matter surface to improve its triangle quality—potentially impacting the most rounded points of the frontal and temporal lobes. We find that v7.1 had the lowest fail rate in the temporal pole compared to v5.3 and v6.0 suggesting an improvement in the parcellation.

Subcortical volumes are also another set of metrics derived from FreeSurfer that are of major interest to neuroimaging researchers (Ohi et al., 2020; Satizabal et al., 2019). Efforts to provide references of normative subcortical volume changes that occur as a result of aging have been put forth (Bethlehem et al., 2022; Coupé et al., 2017; Dima et al., 2022; Miletić et al., 2022; Narvacan et al., 2017; Potvin et al., 2016). For example, Potvin et al., 2016 pooled data from 21 research groups (*n* = 2790) and segmented subcortical volumes using FreeSurfer v5.3 to provide norms of volumetric estimate changes during healthy aging. Although this study, along with many others, provides a valuable resource to researchers, we advise caution with the newer versions when referencing normative data derived from v5.3, particularly in the lentiform nucleus. The lentiform nucleus (i.e., the putamen and globus pallidus combined) has often been found to be difficult to segment due to the high white matter content in the pallidum—making it more difficult to distinguish gray‐white matter contrast (Bigler et al., 2020; Makowski et al., 2018; Ochs et al., 2015; Visser et al., 2016). We find poor compatibility in the pallidum and moderate in the putamen when comparing v7.1 and v5.3. Visual QC of these regions revealed a higher failure rate and lower Dice overlap in v5.3 compared to v7.1, particularly in the left hemisphere. However, we find the compatibility between v7.1 and v6.0 to be excellent and the Dice overlap was greater than 90% in the lentiform nucleus. This suggests that changes made in the release of v6.0 contributed to v5.3 discrepancies. For example, the putamen does not extend so far laterally in the two newer versions—a known issue noted in the release notes of v6.0.

The main goal of our work was to evaluate FreeSurfer's latest stable release, v7.1, yet it is also worth noting how v6.0 differs from v5.3. While compatibility was generally good for cortical thickness, regional surface area estimates were more moderately compatible, with the frontal pole even showing poor compatibility, similar to v7.1 compared to v5.3. Temporal lobe regions showing moderate compatibility in surface area between v6.0 and v5.3 included the entorhinal, insula, parahippocampal, and temporal pole. Updates that accompanied the release of v6.0 that may contribute to these compatibility discrepancies include improved accuracy of the cortical labels and an updated template (*fsaverage*) that “fixes” the peri/entorhinal labels. As previously mentioned, v6.0 compatibility with v5.3 was poorest in the pallidum and putamen. Our results coincide with Bigler et al. (2020) where the lowest agreement was also found in the pallidum and putamen when comparing v5.3 to v6.0.

Overall, we note consistencies across sites. For example, HCP, KKI, and OASIS all showed the lowest compatibility in the cingulate regions when comparing v7.1 to the previous versions, and overall lower compatibility in thickness measures compared to surface area. However, some site differences were observed. For example, more widespread lower compatibility in cortical thickness in temporal and frontal regions was seen for HCP compared to KKI and OASIS, which both have larger and anisotropic voxels. The opposite occurred for compatibility of surface area between v5.3 and the later versions where OASIS and KKI showed more widespread compatibility issues compared to HCP, particularly in the temporal regions. HCP also showed the highest overall reliability for cortical thickness and surface area compared to KKI and OASIS with more widespread and lower reliability, likely due to HCP's more advanced acquisition protocol. KKI had the lowest compatibility for the accumbens, particularly when comparing v5.3 to the newer versions, where compatibility was poor/moderate as opposed to moderate/good for OASIS and HCP, both of which are from Siemens scanners, compared to Philips for KKI. Reliability across subcortical regions and cohorts showed generally consistent good to excellent reliability, although v5.3 was most variable for the hippocampus and accumbens.

To assess if our main analysis generalizes to other data sets, we performed a replication analysis for reliability and compatibility using the HNU cohort—a data set composed of 30 participants with a 10 test–retest design within 40 days of the initial baseline scan. While the results for reliability and compatibility of surface area and subcortical volumes were largely in line with our main analysis, we observed distinct trends in the replication data set for cortical thickness. For example, whereas our main analysis showed generally good to excellent reliability of all measures for cortical thickness, reliability assessed in the HNU data set showed lower, more widespread differences across the versions, where some regions even had poor reliability. Between version analysis in the replication data set did not show the same distinctly lower compatibility between v7.1 and the earlier versions for the cingulate regions. Instead, the replication analysis showed more widespread discrepancy between v5.3 and the later versions. Differences in compatibility could be attributed to a smaller sample size (*n* = 27), as we only performed this analysis using the first time point to replicate the methods in our main data sets. (Individual data set variation can be observed in the supplementary materials, Figures S1–S8, Tables S3–S6.) However, despite the smaller sample size, the study design lends itself to more stable within‐version reliability measures, due to the increased number of repeated measures. This suggests that there may be another source of variation that is not accounted for, such as voxel thickness or scanner manufacturer, particularly impacting cortical thickness. KKI and OASIS have anisotropic voxel sizes, with thickness being 1.2–1.25 mm, while HNU has isotropic 1 mm voxels. Also, while HCP, and OASIS were scanned on Siemens and KKI on Philips, HNU used a GE scanner. Interscanner variability of local thickness between Siemens and GE scanners, for example, was found to be on average 0.15 mm in Han et al. (2006), and differences in volumetric measures between all three different platforms was observed in Jovicich et al. (2009).

One limitation of our study was that there was no available higher‐resolution or postmortem ground truth data to know which FreeSurfer version most represents true anatomical structure. However, given that many of these measures have been widely studied regarding their relationship with age, even in the absence of postmortem or higher resolution data (Fischl, 2012; Frangou et al., 2022; Salat et al., 2004), we instead assess age associations to gauge the downstream consequences of version differences. Version‐related differences in FreeSurfer metrics between cases and controls have been assessed in Filip et al., 2022. In their work, Filip and colleagues assess group differences between nine preselected cortical and subcortical volumes of patients with type 1 diabetes and those of controls across the latest FreeSurfer versions. They found the statistical significance between groups was dependent on version; notably, analyses run using v7.1 metrics did not replicate the results of older versions. Our compatibility findings highlight specifically the regions for which effects differ. Our work also highlights the dampened effects that might be expected with v7.1, suggesting larger sample sizes might be needed to find similar effects, than what might be expected from power calculations using v5.3 results.

We also performed QC of regional parcellations to rule out any spurious associations with gross mis‐segmentations. One example worth noting is that v7.1 and v6.0 may be better able to handle images with lower quality and/or motion as evidenced by one subject in our UK Biobank subset that failed in v5.3 but not in the newer versions (Figure 4b). This could be due to the improved error handling of the Talairach registration: if one registration fails, v7.1 and v6.0 would try an older atlas. Another example involved the left middle temporal gyrus, which is often susceptible to underestimations due to the spillage/overestimation of the banks of the superior temporal sulcus into that gyrus. This occurred at approximately the same rate across versions. When associating the thickness of both the left banks of the superior temporal sulcus and the middle temporal gyrus with age before QC, all versions reveal significant associations for both regions. After removing subjects encountering this issue, although the direction of the effects stayed the same, neither region was associated with age in any of the versions. While this may be due to a reduced sample size and study power, it is also possible that findings in these regions may not represent true anatomical structure, and may instead be due to common segmentation errors. It is also worth noting that our results are solely based on the DK atlas (Desikan et al., 2006) and translation to other atlases may not apply. We chose the DK atlas as it consists of a set of coarse regions defined by anatomical landmarks that can be reasonably quality controlled. Most other atlases, while possibly more precise, define finer parcellations based on cortical function, connectivity, topography, myelin, or a combination thereof (Glasser et al., 2016; Schaefer et al., 2018). Visual QC by region may not be readily possible when cortical parcellations are finer and there are over 100 regions in each hemisphere, so version performance of segmentation accuracy may be more difficult to compare. Our data sets were exclusively from adults without major neurological abnormalities, so our findings may not necessarily generalize to cohorts of young children, adolescents, or individuals with significant brain abnormalities. Finally, we recognize that repeatability is an important metric, and differences in repeatability may be explained, in part, by differences in operating systems used. While Tustison et al. (2014) found good repeatability for FreeSurfer v5.3, users of multiple workstations should exercise caution when pooling data run on various machines, as differences in floating point precision may affect reproducibility of these measures (Glatard et al., 2015). Containerization packages, such as Docker or Singularity (Matelsky et al., 2018), help mitigate differences in environment along with differences in version, which are quantified in this manuscript.

Overall, we find generally high within‐version reliability across most versions and data sets, and many advantages to using FreeSurfer v7.1 over older versions for adult neuroimaging studies. However, considerable differences are observed when analyzing between‐version compatibility for regional cortical thickness, surface area, and subcortical volumes. It is important to consider these compatibility differences when pooling data or statistical inferences across software versions, and when comparing findings across published works, especially for those regions with lower compatibility. Understanding these differences may help researchers to make informed decisions on study design and provide insight into reproducibility issues.

## CONFLICT OF INTEREST

NJ and PMT received grant support from Biogen, Inc., for research unrelated to this manuscript.

## Supporting information


**APPENDIX S1** Supplementary Information

## Data Availability

All data used in this study are in the public domain (https://www.humanconnectome.org/study/hcp‐young‐adult, https://www.nitrc.org/projects/multimodal, https://www.oasis-brains.org/#data, http://fcon_1000.projects.nitrc.org/indi/CoRR/html/download.html, https://www.ukbiobank.ac.uk/). The data that support the findings of this study are openly available at http://data.brainescience.org/Freesurfer_Reliability/.

## References

[hbm26147-bib-0001] Beijers, L. , Wardenaar, K. J. , van Loo, H. M. , & Schoevers, R. A. (2019). Data‐driven biological subtypes of depression: Systematic review of biological approaches to depression subtyping. Molecular Psychiatry, 24(6), 888–900. 30824865 10.1038/s41380-019-0385-5

[hbm26147-bib-0002] Benjamini, Y. , & Hochberg, Y. (1995). Controlling the false discovery rate: A practical and powerful approach to multiple testing. Journal of the Royal Statistical Society, 57(1), 289–300.

[hbm26147-bib-0003] Bennett, C. M. , & Miller, M. B. (2013). fMRI reliability: Influences of task and experimental design. Cognitive, Affective, & Behavioral Neuroscience, 13(4), 690–702. 10.3758/s13415-013-0195-1 23934630

[hbm26147-bib-0004] Bethlehem, R. A. I. , Seidlitz, J. , White, S. R. , Vogel, J. W. , Anderson, K. M. , Adamson, C. , Adler, S. , Alexopoulos, G. S. , Anagnostou, E. , Areces‐Gonzalez, A. , Astle, D. E. , Auyeung, B. , Ayub, M. , Bae, J. , Ball, G. , Baron‐Cohen, S. , Beare, R. , Bedford, S. A. , Benegal, V. , … Alexander‐Bloch, A. F. (2022). Brain charts for the human lifespan. Nature, 604, 525–533. 10.1038/s41586-022-04554-y 35388223 PMC9021021

[hbm26147-bib-0005] Bigler, E. D. , Skiles, M. , Wade, B. S. C. , Abildskov, T. J. , Tustison, N. J. , Scheibel, R. S. , Newsome, M. R. , Mayer, A. R. , Stone, J. R. , Taylor, B. A. , Tate, D. F. , Walker, W. C. , Levin, H. S. , & Wilde, E. A. (2020). FreeSurfer 5.3 versus 6.0: Are volumes comparable? A chronic effects of neurotrauma consortium study. Brain Imaging and Behavior, 14(5), 1318–1327. 30511116 10.1007/s11682-018-9994-x

[hbm26147-bib-0006] Boekel, W. , Wagenmakers, E.‐J. , Belay, L. , Verhagen, J. , Brown, S. , & Forstmann, B. U. (2015). A purely confirmatory replication study of structural brain‐behavior correlations. Cortex, 66, 115–133. 25684445 10.1016/j.cortex.2014.11.019

[hbm26147-bib-0007] Botvinik‐Nezer, R. , Holzmeister, F. , Camerer, C. F. , Dreber, A. , Huber, J. , Johannesson, M. , Kirchler, M. , Iwanir, R. , Mumford, J. A. , Adcock, R. A. , Avesani, P. , Baczkowski, B. M. , Bajracharya, A. , Bakst, L. , Ball, S. , Barilari, M. , Bault, N. , Beaton, D. , Beitner, J. , … Schonberg, T. (2020). Variability in the analysis of a single neuroimaging dataset by many teams. Nature, 582(7810), 84–88. 32483374 10.1038/s41586-020-2314-9 PMC7771346

[hbm26147-bib-0008] Bowring, A. , Maumet, C. , & Nichols, T. E. (2019). Exploring the impact of analysis software on task fMRI results. Human Brain Mapping, 40(11), 3362–3384. 31050106 10.1002/hbm.24603 PMC6618324

[hbm26147-bib-0009] Braak, H. , & Braak, E. (1991). Neuropathological stageing of Alzheimer‐related changes. Acta Neuropathologica, 82(4), 239–259. 1759558 10.1007/BF00308809

[hbm26147-bib-0010] Bryer, E. J. , Medaglia, J. D. , Rostami, S. , & Hillary, F. G. (2013). Neural recruitment after mild traumatic brain injury is task dependent: A meta‐analysis. Journal of the International Neuropsychological Society, 19(7), 751–762. 23656706 10.1017/S1355617713000490

[hbm26147-bib-0011] Button, K. S. , Ioannidis, J. P. A. , Mokrysz, C. , Nosek, B. A. , Flint, J. , Robinson, E. S. J. , & Munafò, M. R. (2013). Power failure: Why small sample size undermines the reliability of neuroscience. Nature Reviews. Neuroscience, 14(5), 365–376. 23571845 10.1038/nrn3475

[hbm26147-bib-0012] Carp, J. (2012). The secret lives of experiments: Methods reporting in the fMRI literature. NeuroImage, 63(1), 289–300. 22796459 10.1016/j.neuroimage.2012.07.004

[hbm26147-bib-0013] Coupé, P. , Catheline, G. , Lanuza, E. , Manjón, J. V. , & Alzheimer's Disease Neuroimaging Initiative . (2017). Towards a unified analysis of brain maturation and aging across the entire lifespan: A MRI analysis. Human Brain Mapping, 38(11), 5501–5518. 28737295 10.1002/hbm.23743 PMC6866824

[hbm26147-bib-0014] Coutureau, E. , & Di Scala, G. (2009). Entorhinal cortex and cognition. Progress in Neuro‐Psychopharmacology & Biological Psychiatry, 33(5), 753–761. 19376185 10.1016/j.pnpbp.2009.03.038

[hbm26147-bib-0015] Dadar, M. , Potvin, O. , Camicioli, R. , Duchesne, S. , & Alzheimer's Disease Neuroimaging Initiative . (2021). Beware of white matter hyperintensities causing systematic errors in FreeSurfer gray matter segmentations! Human Brain Mapping, 42(9), 2734–2745. 33783933 10.1002/hbm.25398 PMC8127151

[hbm26147-bib-0016] de Godoy, L. L. , Alves, C. A. P. F. , Saavedra, J. S. M. , Studart‐Neto, A. , Nitrini, R. , da Costa Leite, C. , & Bisdas, S. (2021). Understanding brain resilience in superagers: A systematic review. Neuroradiology, 63(5), 663–683. 32995945 10.1007/s00234-020-02562-1

[hbm26147-bib-0017] Desikan, R. S. , Ségonne, F. , Fischl, B. , Quinn, B. T. , Dickerson, B. C. , Blacker, D. , Buckner, R. L. , Dale, A. M. , Maguire, R. P. , Hyman, B. T. , Albert, M. S. , & Killiany, R. J. (2006). An automated labeling system for subdividing the human cerebral cortex on MRI scans into gyral based regions of interest. NeuroImage, 31(3), 968–980. 16530430 10.1016/j.neuroimage.2006.01.021

[hbm26147-bib-0018] Dice, L. R. (1945). Measures of the amount of ecologic association between species. Ecology, 26(3), 297–302.

[hbm26147-bib-0019] Dichter, G. S. , Gibbs, D. , & Smoski, M. J. (2015). A systematic review of relations between resting‐state functional‐MRI and treatment response in major depressive disorder. Journal of Affective Disorders, 172, 8–17. 25451389 10.1016/j.jad.2014.09.028 PMC4375066

[hbm26147-bib-0020] Dickie, E. , Hodge, S. , Craddock, R. , Poline, J.‐B. , & Kennedy, D. (2017). Tools matter: Comparison of two surface analysis tools applied to the ABIDE dataset. Research Ideas and Outcomes, 3, e13726. 10.3897/rio.3.e13726

[hbm26147-bib-0021] Dima, D. , Modabbernia, A. , Papachristou, E. , Doucet, G. E. , Agartz, I. , Aghajani, M. , Akudjedu, T. N. , Albajes‐Eizagirre, A. , Alnaes, D. , Alpert, K. I. , Andersson, M. , Andreasen, N. C. , Andreassen, O. A. , Asherson, P. , Banaschewski, T. , Bargallo, N. , Baumeister, S. , Baur‐Streubel, R. , Bertolino, A. , … Karolinska Schizophrenia Project (KaSP) . (2022). Subcortical volumes across the lifespan: Data from 18,605 healthy individuals aged 3‐90 years. Human Brain Mapping, 43(1), 452–469. 33570244 10.1002/hbm.25320 PMC8675429

[hbm26147-bib-0022] Dinga, R. , Schmaal, L. , Penninx, B. W. J. H. , van Tol, M. J. , Veltman, D. J. , van Velzen, L. , Mennes, M. , van der Wee, N. J. A. , & Marquand, A. F. (2019). Evaluating the evidence for biotypes of depression: Methodological replication and extension of. NeuroImage: Clinical, 22, 101796. 30935858 10.1016/j.nicl.2019.101796 PMC6543446

[hbm26147-bib-0023] Filip, P. , Bednarik, P. , Eberly, L. E. , Moheet, A. , Svatkova, A. , Grohn, H. , Kumar, A. F. , Seaquist, E. R. , & Mangia, S. (2022). Different FreeSurfer versions might generate different statistical outcomes in case‐control comparison studies. Neuroradiology, 64(4), 765–773. 34988592 10.1007/s00234-021-02862-0 PMC8916973

[hbm26147-bib-0024] Fischl, B. (2012). FreeSurfer. NeuroImage, 62(2), 774–781. 22248573 10.1016/j.neuroimage.2012.01.021 PMC3685476

[hbm26147-bib-0025] Fonseka, T. M. , MacQueen, G. M. , & Kennedy, S. H. (2018). Neuroimaging biomarkers as predictors of treatment outcome in major depressive disorder. Journal of Affective Disorders, 233, 21–35. 29150145 10.1016/j.jad.2017.10.049

[hbm26147-bib-0026] Fortin, J.‐P. , Cullen, N. , Sheline, Y. I. , Taylor, W. D. , Aselcioglu, I. , Cook, P. A. , Adams, P. , Cooper, C. , Fava, M. , McGrath, P. J. , McInnis, M. , Phillips, M. L. , Trivedi, M. H. , Weissman, M. M. , & Shinohara, R. T. (2018). Harmonization of cortical thickness measurements across scanners and sites. NeuroImage, 167, 104–120. 29155184 10.1016/j.neuroimage.2017.11.024 PMC5845848

[hbm26147-bib-0027] Frangou, S. , Modabbernia, A. , Williams, S. C. R. , Papachristou, E. , Doucet, G. E. , Agartz, I. , Aghajani, M. , Akudjedu, T. N. , Albajes‐Eizagirre, A. , Alnaes, D. , Alpert, K. I. , Andersson, M. , Andreasen, N. C. , Andreassen, O. A. , Asherson, P. , Banaschewski, T. , Bargallo, N. , Baumeister, S. , Baur‐Streubel, R. , … Dima, D. (2022). Cortical thickness across the lifespan: Data from 17,075 healthy individuals aged 3‐90 years. Human Brain Mapping, 43(1), 431–451. 33595143 10.1002/hbm.25364 PMC8675431

[hbm26147-bib-0028] Garcia, A. D. , & Buffalo, E. A. (2020). Anatomy and function of the primate entorhinal cortex. Annual Review of Vision Science, 6, 411–432. 10.1146/annurev-vision-030320-041115 PMC12889097 32580662

[hbm26147-bib-0029] Gefen, T. , Peterson, M. , Papastefan, S. T. , Martersteck, A. , Whitney, K. , Rademaker, A. , Bigio, E. H. , Weintraub, S. , Rogalski, E. , Mesulam, M.‐M. , & Geula, C. (2015). Morphometric and histologic substrates of cingulate integrity in elders with exceptional memory capacity. The Journal of Neuroscience, 35(4), 1781–1791. 25632151 10.1523/JNEUROSCI.2998-14.2015 PMC4308613

[hbm26147-bib-0030] Glasser, M. F. , Coalson, T. S. , Robinson, E. C. , Hacker, C. D. , Harwell, J. , Yacoub, E. , Ugurbil, K. , Andersson, J. , Beckmann, C. F. , Jenkinson, M. , Smith, S. M. , & Van Essen, D. C. (2016). A multi‐modal parcellation of human cerebral cortex. Nature, 536(7615), 171–178. 27437579 10.1038/nature18933 PMC4990127

[hbm26147-bib-0031] Glatard, T. , Lewis, L. B. , Ferreira da Silva, R. , Adalat, R. , Beck, N. , Lepage, C. , Rioux, P. , Rousseau, M.‐E. , Sherif, T. , Deelman, E. , Khalili‐Mahani, N. , & Evans, A. C. (2015). Reproducibility of neuroimaging analyses across operating systems. Frontiers in Neuroinformatics, 9, 12. 25964757 10.3389/fninf.2015.00012 PMC4408913

[hbm26147-bib-0032] Gorgolewski, K. J. , & Poldrack, R. A. (2016). A practical guide for improving transparency and reproducibility in neuroimaging research. PLoS Biology, 14(7), e1002506. 27389358 10.1371/journal.pbio.1002506 PMC4936733

[hbm26147-bib-0033] Gorgolewski, K. J. , Varoquaux, G. , Rivera, G. , Schwarz, Y. , Ghosh, S. S. , Maumet, C. , Sochat, V. V. , Nichols, T. E. , Poldrack, R. A. , Poline, J.‐B. , Yarkoni, T. , & Margulies, D. S. (2015). NeuroVault.org: A web‐based repository for collecting and sharing unthresholded statistical maps of the human brain. Frontiers in Neuroinformatics, 9, 8. 25914639 10.3389/fninf.2015.00008 PMC4392315

[hbm26147-bib-0034] Grasby, K. L. , Jahanshad, N. , Painter, J. N. , Colodro‐Conde, L. , Bralten, J. , Hibar, D. P. , Lind, P. A. , Pizzagalli, F. , Ching, C. R. K. , McMahon, M. A. B. , Shatokhina, N. , Zsembik, L. C. P. , Thomopoulos, S. I. , Zhu, A. H. , Strike, L. T. , Agartz, I. , Alhusaini, S. , Almeida, M. A. A. , Alnæs, D. , … Enhancing NeuroImaging Genetics through Meta‐Analysis Consortium (ENIGMA)—Genetics working group . (2020). The genetic architecture of the human cerebral cortex. Science, 367(6484), eaay6690. 10.1126/science.aay6690 PMC7295264 32193296

[hbm26147-bib-0035] Gronenschild, E. H. B. M. , Habets, P. , Jacobs, H. I. L. , Mengelers, R. , Rozendaal, N. , van Os, J. , & Marcelis, M. (2012). The effects of FreeSurfer version, workstation type, and Macintosh operating system version on anatomical volume and cortical thickness measurements. PLoS One, 7(6), e38234. 22675527 10.1371/journal.pone.0038234 PMC3365894

[hbm26147-bib-0036] Han, X. , Jovicich, J. , Salat, D. , van der Kouwe, A. , Quinn, B. , Czanner, S. , Busa, E. , Pacheco, J. , Albert, M. , Killiany, R. , Maguire, P. , Rosas, D. , Makris, N. , Dale, A. , Dickerson, B. , & Fischl, B. (2006). Reliability of MRI‐derived measurements of human cerebral cortical thickness: The effects of field strength, scanner upgrade and manufacturer. NeuroImage, 32(1), 180–194. 16651008 10.1016/j.neuroimage.2006.02.051

[hbm26147-bib-0037] Harrison, T. M. , Maass, A. , Baker, S. L. , & Jagust, W. J. (2018). Brain morphology, cognition, and β‐amyloid in older adults with superior memory performance. Neurobiology of Aging, 67, 162–170. 29665578 10.1016/j.neurobiolaging.2018.03.024 PMC5955827

[hbm26147-bib-0038] Harrison, T. M. , Weintraub, S. , Mesulam, M.‐M. , & Rogalski, E. (2012). Superior memory and higher cortical volumes in unusually successful cognitive aging. Journal of the International Neuropsychological Society, 18(6), 1081–1085. 23158231 10.1017/S1355617712000847 PMC3547607

[hbm26147-bib-0039] Hinojosa, C. A. , Kaur, N. , VanElzakker, M. B. , & Shin, L. M. (2019). Cingulate subregions in posttraumatic stress disorder, chronic stress, and treatment. Handbook of Clinical Neurology, 166, 355–370. 31731922 10.1016/B978-0-444-64196-0.00020-0

[hbm26147-bib-0040] Hodge, S. M. , Haselgrove, C. , Honor, L. , Kennedy, D. N. , & Frazier, J. A. (2020). An assessment of the autism neuroimaging literature for the prospects of re‐executability. F1000Research, 9, 1031. 33796274 10.12688/f1000research.25306.1 PMC7968525

[hbm26147-bib-0041] Horien, C. , Noble, S. , Greene, A. S. , Lee, K. , Barron, D. S. , Gao, S. , O'Connor, D. , Salehi, M. , Dadashkarimi, J. , Shen, X. , Lake, E. M. R. , Constable, R. T. , & Scheinost, D. (2021). A hitchhiker's guide to working with large, open‐source neuroimaging datasets. Nature Human Behaviour, 5(2), 185–193. 10.1038/s41562-020-01005-4 PMC7992920 33288916

[hbm26147-bib-0042] Jovicich, J. , Czanner, S. , Han, X. , Salat, D. , van der Kouwe, A. , Quinn, B. , Pacheco, J. , Albert, M. , Killiany, R. , Blacker, D. , Maguire, P. , Rosas, D. , Makris, N. , Gollub, R. , Dale, A. , Dickerson, B. C. , & Fischl, B. (2009). MRI‐derived measurements of human subcortical, ventricular and intracranial brain volumes: Reliability effects of scan sessions, acquisition sequences, data analyses, scanner upgrade, scanner vendors and field strengths. NeuroImage, 46(1), 177–192. 19233293 10.1016/j.neuroimage.2009.02.010 PMC2866077

[hbm26147-bib-0043] Kang, S.‐G. , & Cho, S.‐E. (2020). Neuroimaging biomarkers for predicting treatment response and recurrence of major depressive disorder. International Journal of Molecular Sciences, 21(6), 2148. 10.3390/ijms21062148 PMC7139562 32245086

[hbm26147-bib-0044] Kennedy, D. N. , Abraham, S. A. , Bates, J. F. , Crowley, A. , Ghosh, S. , Gillespie, T. , Goncalves, M. , Grethe, J. S. , Halchenko, Y. O. , Hanke, M. , Haselgrove, C. , Hodge, S. M. , Jarecka, D. , Kaczmarzyk, J. , Keator, D. B. , Meyer, K. , Martone, M. E. , Padhy, S. , Poline, J.‐B. , … Travers, M. (2019). Everything matters: The ReproNim perspective on reproducible neuroimaging. Frontiers in Neuroinformatics, 13, 1. 30792636 10.3389/fninf.2019.00001 PMC6374302

[hbm26147-bib-0045] Koo, T. K. , & Li, M. Y. (2016). A guideline of selecting and reporting intraclass correlation coefficients for reliability research. Journal of Chiropractic Medicine, 15(2), 155–163. 27330520 10.1016/j.jcm.2016.02.012 PMC4913118

[hbm26147-bib-0046] Landman, B. A. , Huang, A. J. , Gifford, A. , Vikram, D. S. , Lim, I. A. L. , Farrell, J. A. D. , Bogovic, J. A. , Hua, J. , Chen, M. , Jarso, S. , Smith, S. A. , Joel, S. , Mori, S. , Pekar, J. J. , Barker, P. B. , Prince, J. L. , & van Zijl, P. C. M. (2010). Multi‐parametric neuroimaging reproducibility: A 3‐T resource study. NeuroImage, 54(4), 2854–2866. 21094686 10.1016/j.neuroimage.2010.11.047 PMC3020263

[hbm26147-bib-0047] Lindquist, M. (2020). Neuroimaging results altered by varying analysis pipelines. Nature, 582(7810), 36–37. 32433631 10.1038/d41586-020-01282-z

[hbm26147-bib-0048] Littlejohns, T. J. , Holliday, J. , Gibson, L. M. , Garratt, S. , Oesingmann, N. , Alfaro‐Almagro, F. , Bell, J. D. , Boultwood, C. , Collins, R. , Conroy, M. C. , Crabtree, N. , Doherty, N. , Frangi, A. F. , Harvey, N. C. , Leeson, P. , Miller, K. L. , Neubauer, S. , Petersen, S. E. , Sellors, J. , … Allen, N. E. (2020). The UKUK Biobank imaging enhancement of 100,000 participants: Rationale, data collection, management and future directions. Nature Communications, 11(1), 2624. 10.1038/s41467-020-15948-9 PMC7250878 32457287

[hbm26147-bib-0049] Makowski, C. , Béland, S. , Kostopoulos, P. , Bhagwat, N. , Devenyi, G. A. , Malla, A. K. , Joober, R. , Lepage, M. , & Chakravarty, M. M. (2018). Evaluating accuracy of striatal, pallidal, and thalamic segmentation methods: Comparing automated approaches to manual delineation. NeuroImage, 170, 182–198. 28259781 10.1016/j.neuroimage.2017.02.069

[hbm26147-bib-0050] Marcus, D. S. , Wang, T. H. , Parker, J. , Csernansky, J. G. , Morris, J. C. , & Buckner, R. L. (2007). Open access series of imaging studies (OASIS): Cross‐sectional MRI data in young, middle aged, nondemented, and demented older adults. Journal of Cognitive Neuroscience, 19(9), 1498–1507. 17714011 10.1162/jocn.2007.19.9.1498

[hbm26147-bib-0051] Matelsky, J. , Kiar, G. , Johnson, E. , Rivera, C. , Toma, M. , & Gray‐Roncal, W. (2018). Container‐based clinical solutions for portable and reproducible image analysis. Journal of Digital Imaging, 31(3), 315–320. 29740715 10.1007/s10278-018-0089-4 PMC5959838

[hbm26147-bib-0052] Meijerman, A. , Amiri, H. , Steenwijk, M. D. , Jonker, M. A. , van Schijndel, R. A. , Cover, K. S. , Vrenken, H. , & Alzheimer's Disease Neuroimaging Initiative . (2018). Reproducibility of deep gray matter atrophy rate measurement in a large multicenter dataset. AJNR. American Journal of Neuroradiology, 39(1), 46–53. 29191870 10.3174/ajnr.A5459 PMC7410697

[hbm26147-bib-0053] Miletić, S. , Bazin, P.‐L. , Isherwood, S. J. S. , Keuken, M. C. , Alkemade, A. , & Forstmann, B. U. (2022). Charting human subcortical maturation across the adult lifespan with in vivo 7 T MRI. NeuroImage, 249, 118872. 34999202 10.1016/j.neuroimage.2022.118872

[hbm26147-bib-0054] Miller, K. L. , Alfaro‐Almagro, F. , Bangerter, N. K. , Thomas, D. L. , Yacoub, E. , Xu, J. , Bartsch, A. J. , Jbabdi, S. , Sotiropoulos, S. N. , Andersson, J. L. R. , Griffanti, L. , Douaud, G. , Okell, T. W. , Weale, P. , Dragonu, I. , Garratt, S. , Hudson, S. , Collins, R. , Jenkinson, M. , … Smith, S. M. (2016). Multimodal population brain imaging in the UK Biobank prospective epidemiological study. Nature Neuroscience, 19(11), 1523–1536. 27643430 10.1038/nn.4393 PMC5086094

[hbm26147-bib-0055] Müller, V. I. , Cieslik, E. C. , Serbanescu, I. , Laird, A. R. , Fox, P. T. , & Eickhoff, S. B. (2017). Altered brain activity in unipolar depression revisited: Meta‐analyses of neuroimaging studies. JAMA Psychiatry, 74(1), 47–55. 27829086 10.1001/jamapsychiatry.2016.2783 PMC5293141

[hbm26147-bib-0056] Narvacan, K. , Treit, S. , Camicioli, R. , Martin, W. , & Beaulieu, C. (2017). Evolution of deep gray matter volume across the human lifespan. Human Brain Mapping, 38(8), 3771–3790. 28548250 10.1002/hbm.23604 PMC6867004

[hbm26147-bib-0057] Nichols, T. E. , Das, S. , Eickhoff, S. B. , Evans, A. C. , Glatard, T. , Hanke, M. , Kriegeskorte, N. , Milham, M. P. , Poldrack, R. A. , Poline, J.‐B. , Proal, E. , Thirion, B. , Van Essen, D. C. , White, T. , & Yeo, B. T. T. (2017). Best practices in data analysis and sharing in neuroimaging using MRI. Nature Neuroscience, 20(3), 299–303. 28230846 10.1038/nn.4500 PMC5685169

[hbm26147-bib-0058] Ochs, A. L. , Ross, D. E. , Zannoni, M. D. , Abildskov, T. J. , Bigler, E. D. , & Alzheimer's Disease Neuroimaging Initiative . (2015). Comparison of automated brain volume measures obtained with NeuroQuant and FreeSurfer. Journal of Neuroimaging, 25(5), 721–727. 25727700 10.1111/jon.12229

[hbm26147-bib-0059] Ohi, K. , Shimada, T. , Kataoka, Y. , Yasuyama, T. , Kawasaki, Y. , Shioiri, T. , & Thompson, P. M. (2020). Genetic correlations between subcortical brain volumes and psychiatric disorders. The British Journal of Psychiatry: the Journal of Mental Science, 216(5), 280–283. 32000869 10.1192/bjp.2019.277

[hbm26147-bib-0060] Perlaki, G. , Horvath, R. , Nagy, S. A. , Bogner, P. , Doczi, T. , Janszky, J. , & Orsi, G. (2017). Comparison of accuracy between FSL's FIRST and Freesurfer for caudate nucleus and putamen segmentation. Scientific Reports, 7(1), 2418. 28546533 10.1038/s41598-017-02584-5 PMC5445091

[hbm26147-bib-0061] Poldrack, R. A. , & Gorgolewski, K. J. (2017). OpenfMRI: Open sharing of task fMRI data. NeuroImage, 144(Pt B), 259–261. 26048618 10.1016/j.neuroimage.2015.05.073 PMC4669234

[hbm26147-bib-0062] Poldrack, R. A. , Whitaker, K. , & Kennedy, D. (2020). Introduction to the special issue on reproducibility in neuroimaging. NeuroImage, 218, 116357. 31733374 10.1016/j.neuroimage.2019.116357

[hbm26147-bib-0063] Potvin, O. , Mouiha, A. , Dieumegarde, L. , Duchesne, S. , & Alzheimer's Disease Neuroimaging Initiative . (2016). Normative data for subcortical regional volumes over the lifetime of the adult human brain. NeuroImage, 137, 9–20. 27165761 10.1016/j.neuroimage.2016.05.016

[hbm26147-bib-0064] Salat, D. H. , Buckner, R. L. , Snyder, A. Z. , Greve, D. N. , Desikan, R. S. R. , Busa, E. , Morris, J. C. , Dale, A. M. , & Fischl, B. (2004). Thinning of the cerebral cortex in aging. Cerebral Cortex, 14(7), 721–730. 15054051 10.1093/cercor/bhh032

[hbm26147-bib-0065] Satizabal, C. L. , Adams, H. H. H. , Hibar, D. P. , White, C. C. , Knol, M. J. , Stein, J. L. , Scholz, M. , Sargurupremraj, M. , Jahanshad, N. , Roshchupkin, G. V. , Smith, A. V. , Bis, J. C. , Jian, X. , Luciano, M. , Hofer, E. , Teumer, A. , van der Lee, S. J. , Yang, J. , Yanek, L. R. , … Ikram, M. A. (2019). Genetic architecture of subcortical brain structures in 38,851 individuals. Nature Genetics, 51(11), 1624–1636. 31636452 10.1038/s41588-019-0511-y PMC7055269

[hbm26147-bib-0066] Schaefer, A. , Kong, R. , Gordon, E. M. , Laumann, T. O. , Zuo, X.‐N. , Holmes, A. J. , Eickhoff, S. B. , & Yeo, B. T. T. (2018). Local‐global parcellation of the human cerebral cortex from intrinsic functional connectivity MRI. Cerebral Cortex, 28(9), 3095–3114. 28981612 10.1093/cercor/bhx179 PMC6095216

[hbm26147-bib-0067] Spiegelhalder, K. , Regen, W. , Baglioni, C. , Nissen, C. , Riemann, D. , & Kyle, S. D. (2015). Neuroimaging insights into insomnia. Current Neurology and Neuroscience Reports, 15(3), 9. 25687698 10.1007/s11910-015-0527-3

[hbm26147-bib-0068] Stuhrmann, A. , Suslow, T. , & Dannlowski, U. (2011). Facial emotion processing in major depression: A systematic review of neuroimaging findings. Biology of Mood & Anxiety Disorders, 1(1), 10. 22738433 10.1186/2045-5380-1-10 PMC3384264

[hbm26147-bib-0069] Sun, F. W. , Stepanovic, M. R. , Andreano, J. , Barrett, L. F. , Touroutoglou, A. , & Dickerson, B. C. (2016). Youthful brains in older adults: Preserved neuroanatomy in the default mode and salience networks contributes to youthful memory in superaging. The Journal of Neuroscience, 36(37), 9659–9668. 27629716 10.1523/JNEUROSCI.1492-16.2016 PMC5039247

[hbm26147-bib-0070] Thaker, A. A. , Weinberg, B. D. , Dillon, W. P. , Hess, C. P. , Cabral, H. J. , Fleischman, D. A. , Leurgans, S. E. , Bennett, D. A. , Hyman, B. T. , Albert, M. S. , Killiany, R. J. , Fischl, B. , Dale, A. M. , & Desikan, R. S. (2017). Entorhinal cortex: Antemortem cortical thickness and postmortem neurofibrillary tangles and amyloid pathology. AJNR. American Journal of Neuroradiology, 38(5), 961–965. 28279988 10.3174/ajnr.A5133 PMC5433913

[hbm26147-bib-0071] Thompson, P. M. , Jahanshad, N. , Ching, C. R. K. , Salminen, L. E. , Thomopoulos, S. I. , Bright, J. , Baune, B. T. , Bertolín, S. , Bralten, J. , Bruin, W. B. , Bülow, R. , Chen, J. , Chye, Y. , Dannlowski, U. , de Kovel, C. G. F. , Donohoe, G. , Eyler, L. T. , Faraone, S. V. , Favre, P. , … ENIGMA Consortium . (2020). ENIGMA and global neuroscience: A decade of large‐scale studies of the brain in health and disease across more than 40 countries. Translational Psychiatry, 10(1), 100. 32198361 10.1038/s41398-020-0705-1 PMC7083923

[hbm26147-bib-0072] Touroutoglou, A. , & Dickerson, B. C. (2019). Cingulate‐centered large‐scale networks: Normal functions, aging, and neurodegenerative disease. Handbook of Clinical Neurology, 166, 113–127. 31731908 10.1016/B978-0-444-64196-0.00008-X

[hbm26147-bib-0073] Tustison, N. J. , Cook, P. A. , Klein, A. , Song, G. , Das, S. R. , Duda, J. T. , Kandel, B. M. , van Strien, N. , Stone, J. R. , Gee, J. C. , & Avants, B. B. (2014). Large‐scale evaluation of ANTs and FreeSurfer cortical thickness measurements. NeuroImage, 99, 166–179. 24879923 10.1016/j.neuroimage.2014.05.044

[hbm26147-bib-0074] Van Essen, D. C. , Smith, S. M. , Barch, D. M. , Behrens, T. E. J. , Yacoub, E. , Ugurbil, K. , & WU‐Minn HCP Consortium . (2013). The WU‐Minn Human Connectome Project: An overview. NeuroImage, 80, 62–79. 23684880 10.1016/j.neuroimage.2013.05.041 PMC3724347

[hbm26147-bib-0075] Vicente‐Saez, R. , & Martinez‐Fuentes, C. (2018). Open Science now: A systematic literature review for an integrated definition. Journal of Business Research, 88, 428–436.

[hbm26147-bib-0076] Visser, E. , Keuken, M. C. , Douaud, G. , Gaura, V. , Bachoud‐Levi, A.‐C. , Remy, P. , Forstmann, B. U. , & Jenkinson, M. (2016). Automatic segmentation of the striatum and globus pallidus using MIST: Multimodal image segmentation tool. NeuroImage, 125, 479–497. 26477650 10.1016/j.neuroimage.2015.10.013 PMC4692519

[hbm26147-bib-0077] Vogt, B. A. (2019). Cingulate impairments in ADHD: Comorbidities, connections, and treatment. Handbook of Clinical Neurology, 166, 297–314. 31731917 10.1016/B978-0-444-64196-0.00016-9

[hbm26147-bib-0078] Weiner, M. W. , Veitch, D. P. , Aisen, P. S. , Beckett, L. A. , Cairns, N. J. , Cedarbaum, J. , Donohue, M. C. , Green, R. C. , Harvey, D. , Jack, C. R., Jr. , Jagust, W. , Morris, J. C. , Petersen, R. C. , Saykin, A. J. , Shaw, L. , Thompson, P. M. , Toga, A. W. , Trojanowski, J. Q. , & Alzheimer's Disease Neuroimaging Initiative . (2015). Impact of the Alzheimer's Disease Neuroimaging Initiative, 2004 to 2014. Alzheimer's & Dementia, 11(7), 865–884. 10.1016/j.jalz.2015.04.005 PMC4659407 26194320

[hbm26147-bib-0079] Weiner, M. W. , Veitch, D. P. , Aisen, P. S. , Beckett, L. A. , Cairns, N. J. , Green, R. C. , Harvey, D. , Jack, C. R., Jr. , Jagust, W. , Morris, J. C. , Petersen, R. C. , Salazar, J. , Saykin, A. J. , Shaw, L. M. , Toga, A. W. , Trojanowski, J. Q. , & Alzheimer's Disease Neuroimaging Initiative . (2017). The Alzheimer's Disease Neuroimaging Initiative 3: Continued innovation for clinical trial improvement. Alzheimer's & Dementia, 13(5), 561–571. 10.1016/j.jalz.2016.10.006 PMC5536850 27931796

[hbm26147-bib-0080] Winkler, A. M. , Kochunov, P. , Blangero, J. , Almasy, L. , Zilles, K. , Fox, P. T. , Duggirala, R. , & Glahn, D. C. (2010). Cortical thickness or grey matter volume? The importance of selecting the phenotype for imaging genetics studies. NeuroImage, 53(3), 1135–1146. 20006715 10.1016/j.neuroimage.2009.12.028 PMC2891595

[hbm26147-bib-0081] Yan, S. , Qian, T. , Maréchal, B. , Kober, T. , Zhang, X. , Zhu, J. , Lei, J. , Li, M. , & Jin, Z. (2020). Test‐retest variability of brain morphometry analysis: An investigation of sequence and coil effects. Annals of Translational Medicine, 8(1), 12. 32055603 10.21037/atm.2019.11.149 PMC6995743

[hbm26147-bib-0082] Zavaliangos‐Petropulu, A. , Tubi, M. A. , Haddad, E. , Zhu, A. , Braskie, M. N. , Jahanshad, N. , Thompson, P. M. , & Liew, S.‐L. (2022). Testing a convolutional neural network‐based hippocampal segmentation method in a stroke population. Human Brain Mapping, 43(1), 234–243. 33067842 10.1002/hbm.25210 PMC8675423

[hbm26147-bib-0083] Zuo, X.‐N. , Anderson, J. S. , Bellec, P. , Birn, R. M. , Biswal, B. B. , Blautzik, J. , Breitner, J. C. S. , Buckner, R. L. , Calhoun, V. D. , Castellanos, F. X. , Chen, A. , Chen, B. , Chen, J. , Chen, X. , Colcombe, S. J. , Courtney, W. , Craddock, R. C. , Di Martino, A. , Dong, H.‐M. , … Milham, M. P. (2014). An open science resource for establishing reliability and reproducibility in functional connectomics. Scientific Data, 1, 140049. 25977800 10.1038/sdata.2014.49 PMC4421932

[hbm26147-bib-0084] Zuo, X.‐N. , Xu, T. , & Milham, M. P. (2019). Harnessing reliability for neuroscience research. Nature Human Behaviour, 3(8), 768–771. 10.1038/s41562-019-0655-x 31253883

