# Supplementary material for: Multisite test–retest reliability and compatibility of brain metrics derived from FreeSurfer versions 7.1, 6.0, and 5.3
Source: Hum Brain Mapp. 2022 Nov 27;44(4):1515–32. doi: 10.1002/hbm.26147 (PMC9921222; doi:10.1002/hbm.26147)
Supplement: Supplementary file 1 — APPENDIX S1 Supplementary Information [file HBM-44-1515-s001.pdf]

Supplementary Materials

Figures S1-S8: site specific ICC radar plots ………….………..……….……..…. pages 2-9
Figures S9-S10: z-statistic difference maps ……………………………….. page 10
Figure S11-S14: ComBat harmonization ……………………………………. pages 11-14
Table 1. runtimes ……………………………………………………………… pages 15
Table 2: Euler numbers ……………………………………………….………. pages 16
Table 3: HCP specific ICCs ………………………………………….…..…….. pages 17-49
Table 4: KKI specific ICCs ……………………………………….…….….…… pages 50-82
Table 5: OASIS specific ICCs ………………..………………….…….….…… pages 83-115
Table 6: HNU specific ICCs ………………..…………………..……….……. pages 116-148

Haddad, E., Pizzagalli, F., Zhu, A. H., Bhatt, R. R., Islam, T., Gari, I. B., ... & Jahanshad, N. (2022). Multisite Test-Retest Reliability and Compatibility of Brain Metrics derived from FreeSurfer Versions 7.1, 6.0, and 5.3. Human Brain Mapping

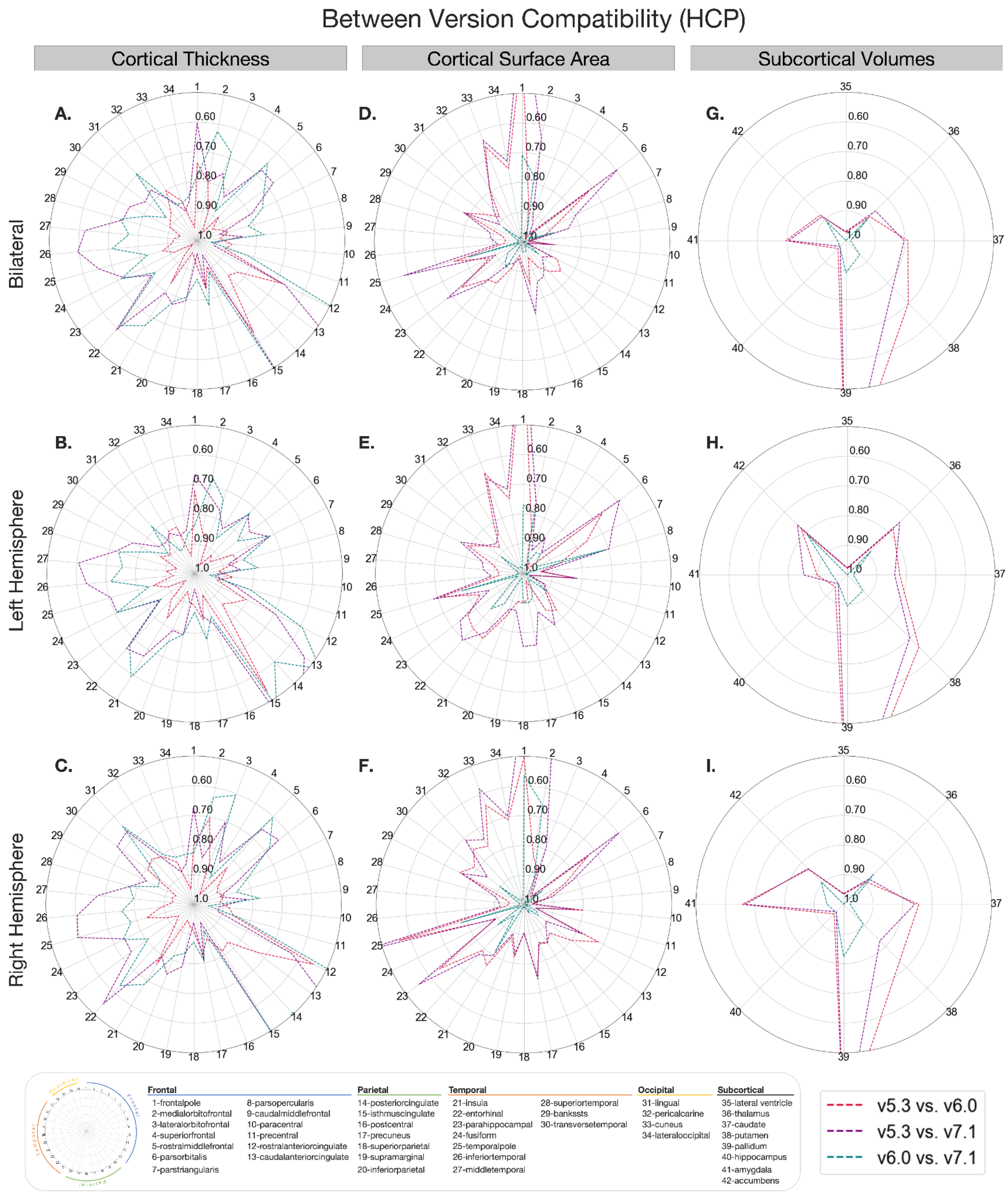

Figure S1. Regional inter-version agreement (compatibility) for the HCP dataset. Bilateral, left, and right ICC2 values comparing cortical thickness (**A, B, C**), cortical surface area (**D, E, F**), and subcortical volumes (**G, H, I**) between versions. Outer concentric circles represent lower ICC2 values, truncated at 0.50, while the center represents ICC2=1. Regions with the lowest compatibility differ for cortical thickness and surface area.

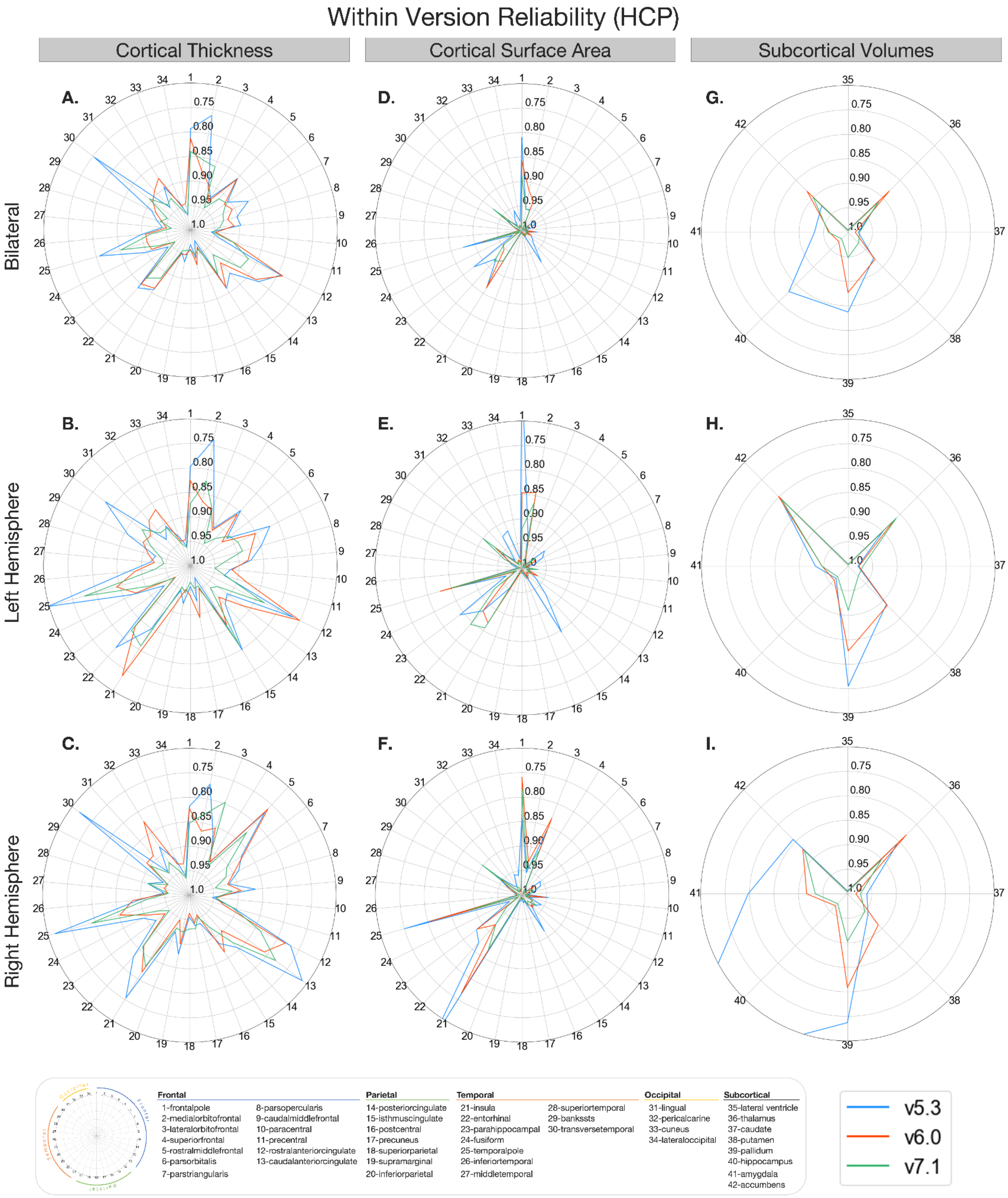

Figure S2. Regional intra-version agreement (reliability) for the HCP dataset. Bilateral, left, and right ICC3 values comparing cortical thickness (**A, B, C**), cortical surface area (**D, E, F**), and subcortical volumes (**G, H, I**) between versions. Outer concentric circles represent smaller ICC3 values, truncated at 0.70. Regions with the lowest reliability differ for cortical thickness and surface area.

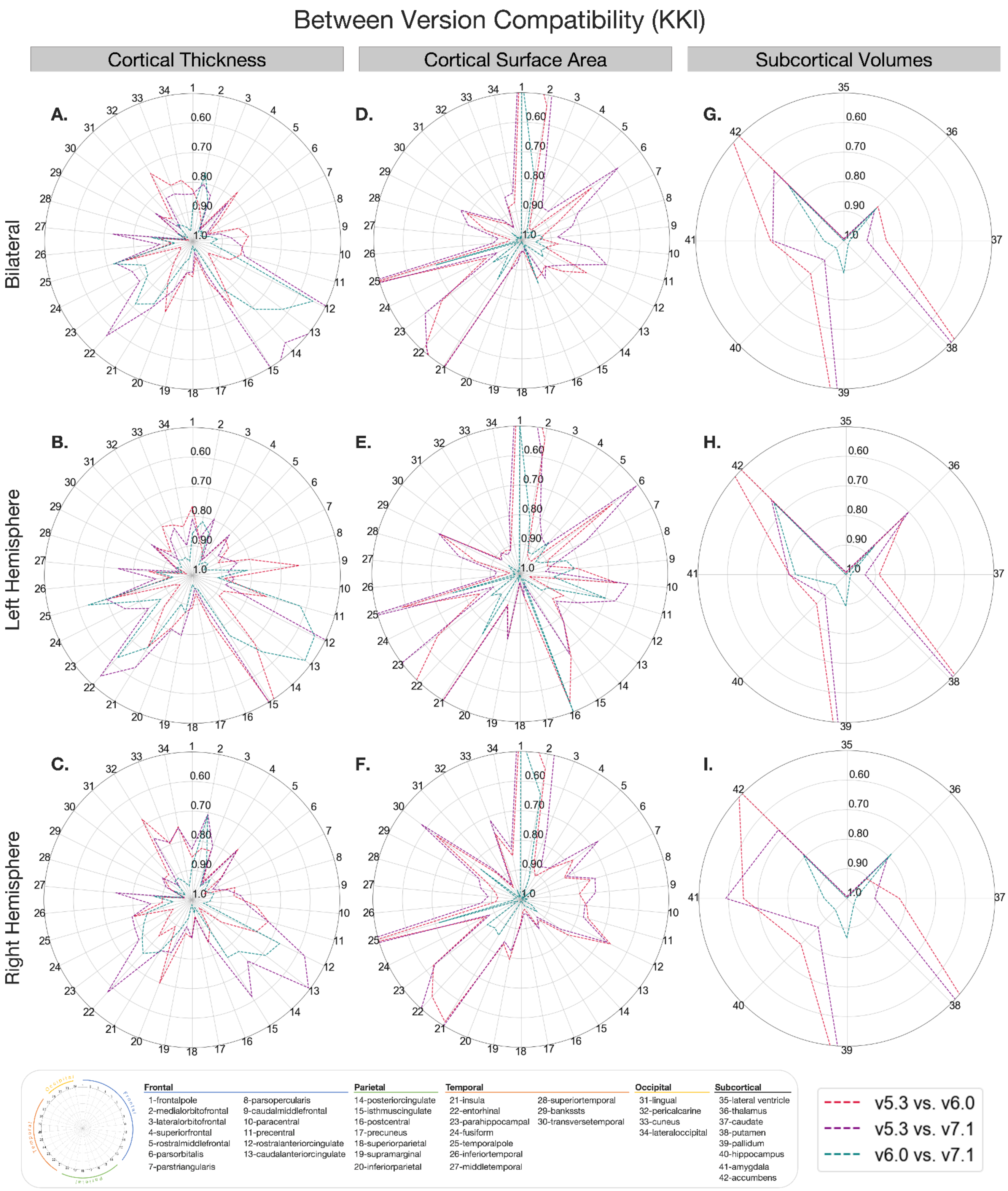

Figure S3. Regional inter-version agreement (compatibility) for the KKI dataset. Bilateral, left, and right ICC2 values comparing cortical thickness (**A, B, C**), cortical surface area (**D, E, F**), and subcortical volumes (**G, H, I**) between versions. Outer concentric circles represent lower ICC2 values, truncated at 0.50, while the center represents ICC2=1. Regions with the lowest compatibility differ for cortical thickness and surface area.

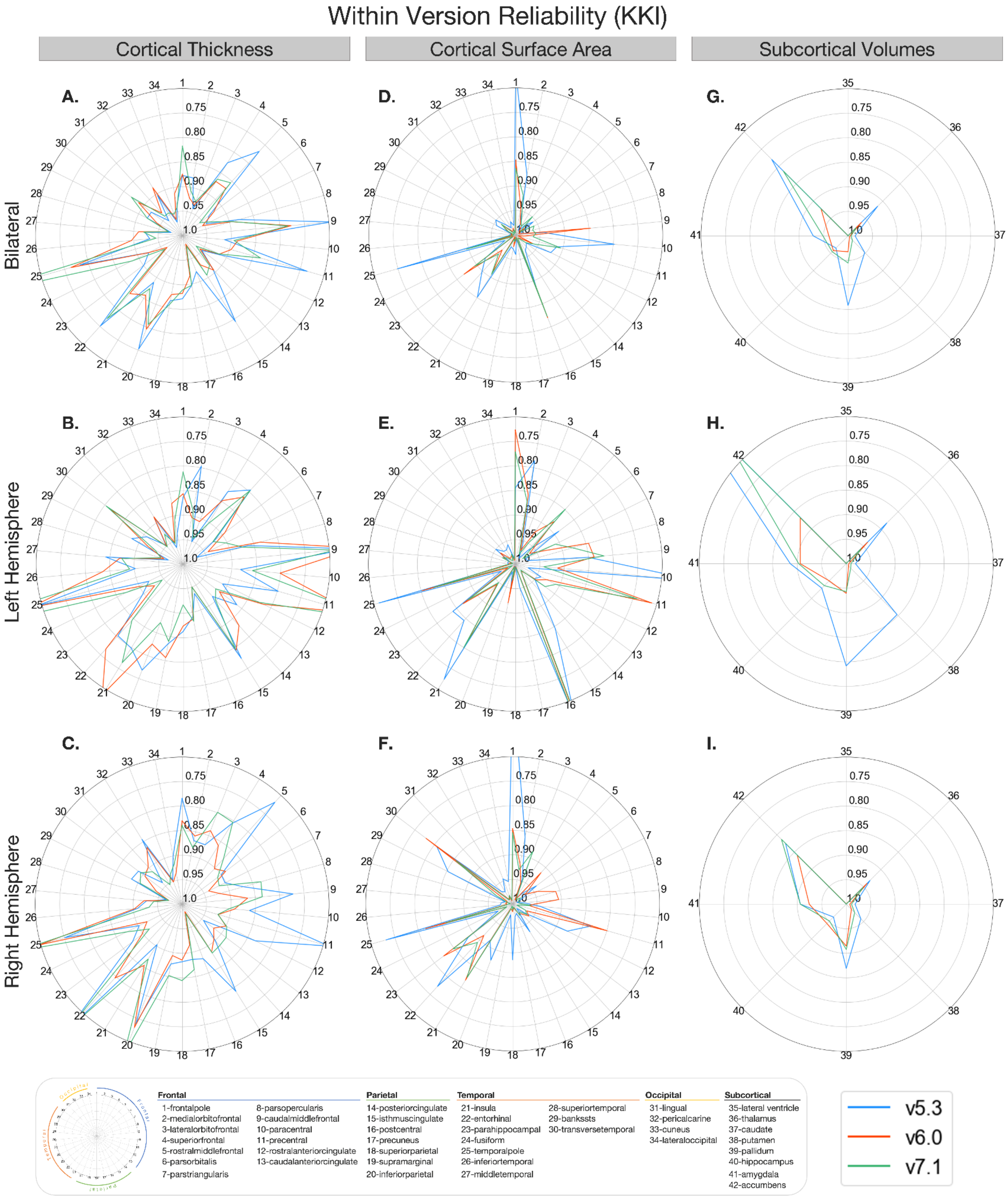

Figure S4. Regional intra-version agreement (reliability) for the KKI dataset. Bilateral, left, and right ICC3 values comparing cortical thickness (**A, B, C**), cortical surface area (**D, E, F**), and subcortical volumes (**G, H, I**) between versions. Outer concentric circles represent smaller ICC3 values, truncated at 0.70. Regions with the lowest reliability differ for cortical thickness and surface area.

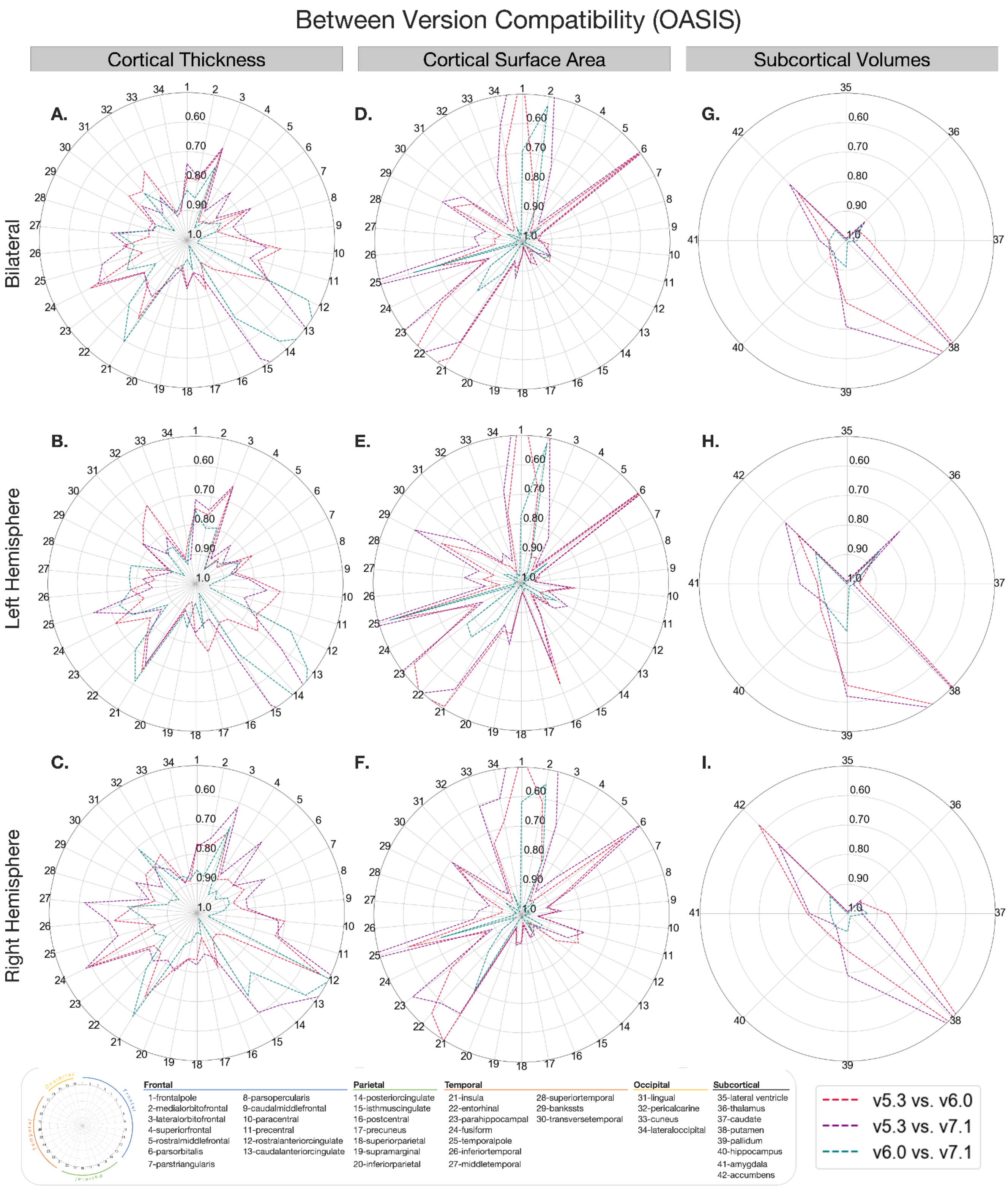

Figure S5. Regional inter-version agreement (compatibility) for the OASIS dataset. Bilateral, left, and right ICC2 values comparing cortical thickness (**A, B, C**), cortical surface area (**D, E, F**), and subcortical volumes (**G, H, I**) between versions. Outer concentric circles represent lower ICC2 values, truncated at 0.50, while the center represents ICC2=1. Regions with the lowest compatibility differ for cortical thickness and surface area.

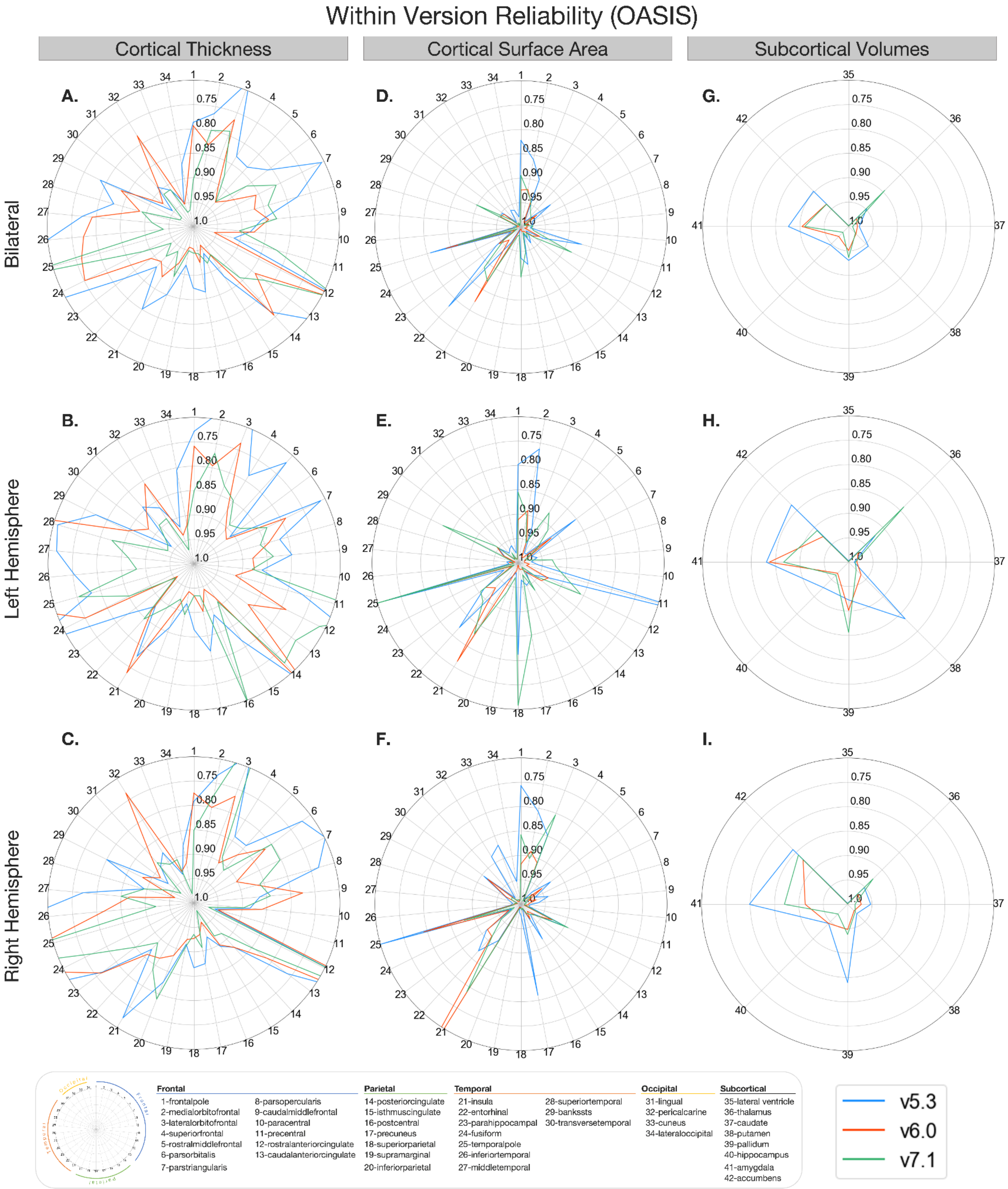

Figure S6. Regional intra-version agreement (reliability) for the OASIS dataset. Bilateral, left, and right ICC3 values comparing cortical thickness (**A, B, C**), cortical surface area (**D, E, F**), and subcortical volumes (**G, H, I**) between versions. Outer concentric circles represent smaller ICC3 values, truncated at 0.70. Regions with the lowest reliability differ for cortical thickness and surface area.

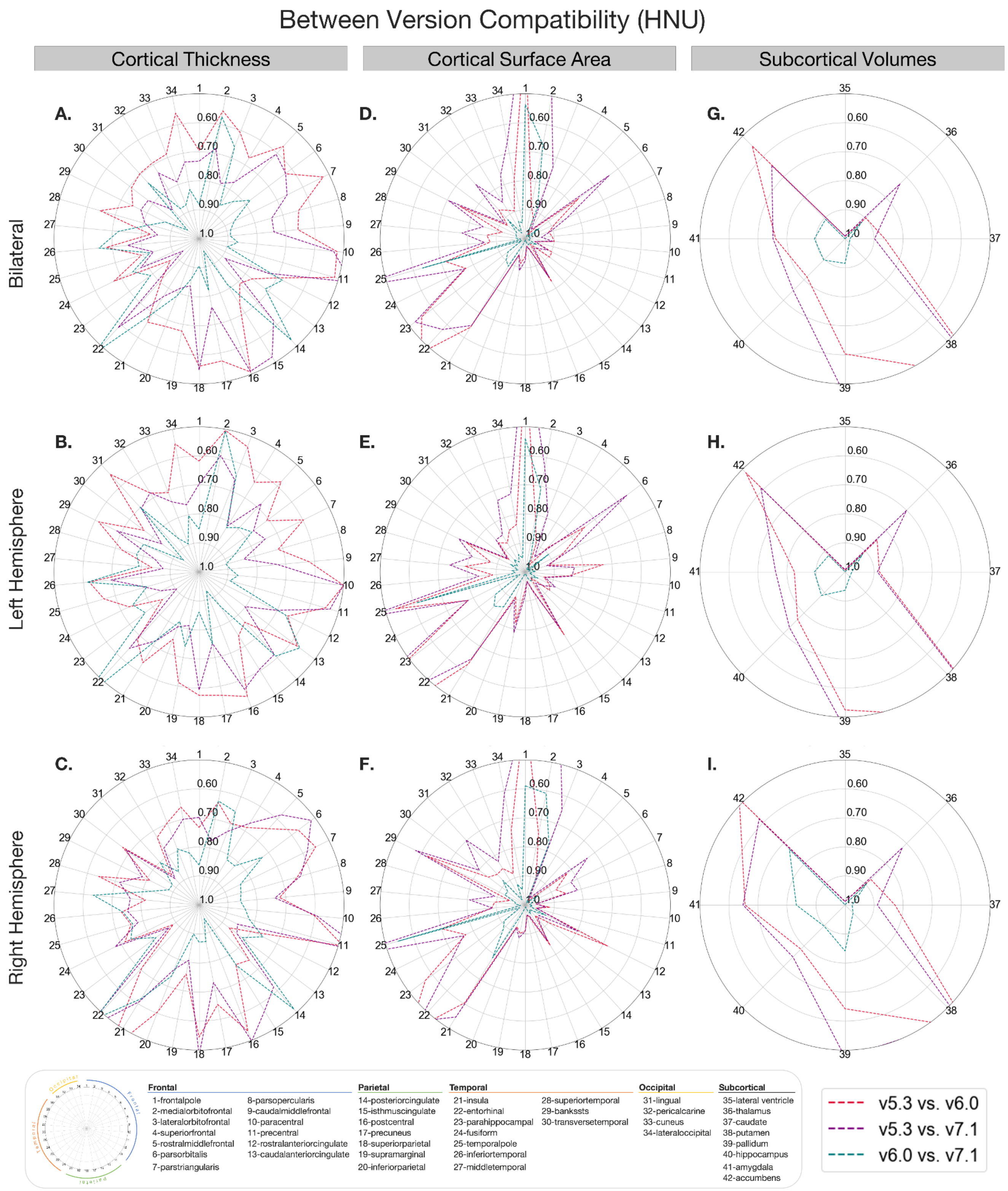

Figure S7. Regional inter-version agreement (compatibility) for the HNU dataset. Bilateral, left, and right ICC2 values comparing cortical thickness (**A, B, C**), cortical surface area (**D, E, F**), and subcortical volumes (**G, H, I**) between versions. Outer concentric circles represent lower ICC2 values, truncated at 0.50, while the center represents ICC2=1. Regions with the lowest compatibility differ for cortical thickness and surface area.

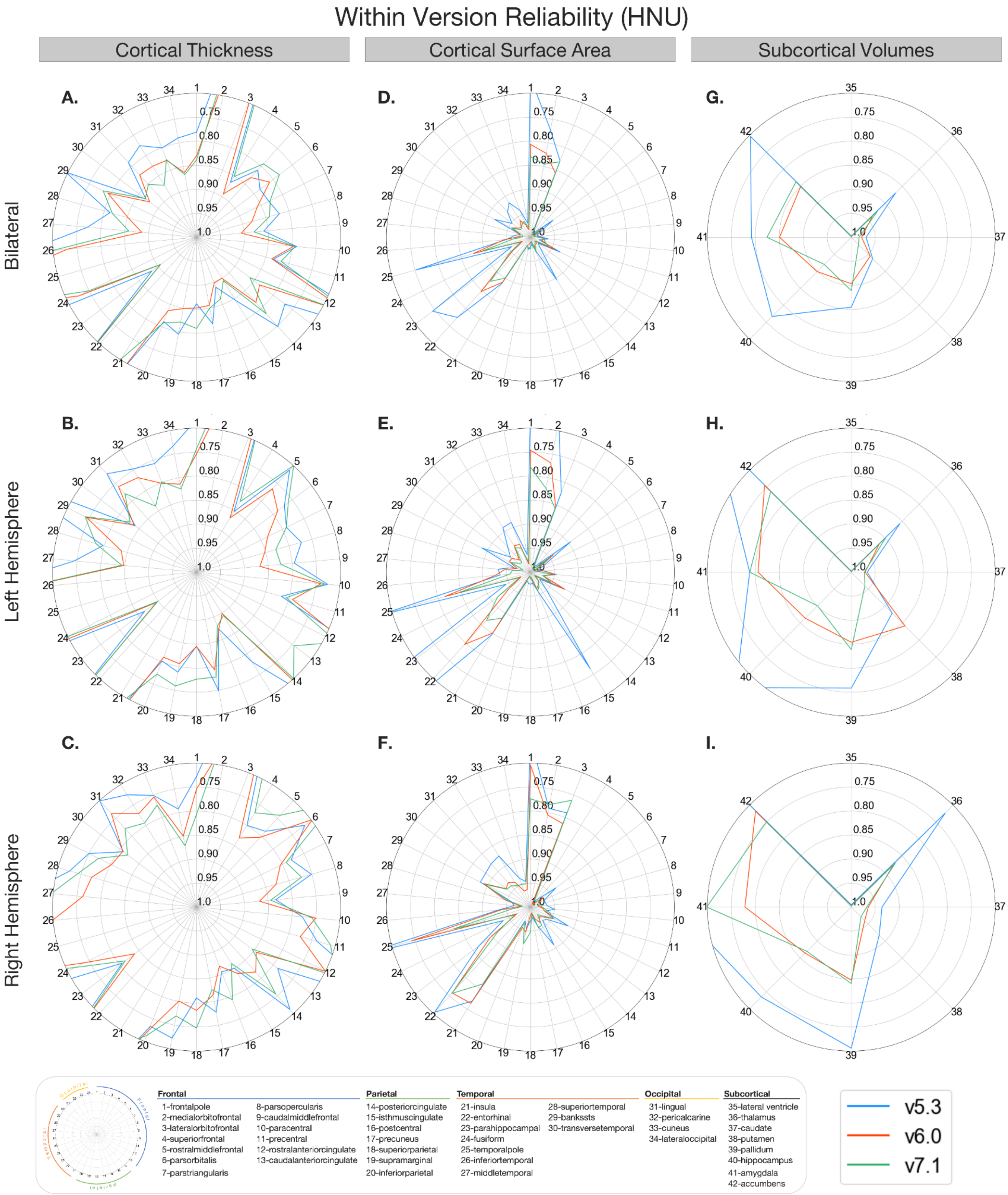

Figure S8. Regional reliability ICC3 measures for the HNU dataset using a 10 test-retest design. Bilateral, left, and right ICC3 values comparing cortical thickness (**A, B, C**), cortical surface area (**D, E, F**), and subcortical volumes (**G, H, I**) between versions. Outer concentric circles represent smaller ICC3 values, truncated at 0.70. Regions with the lowest reliability differ for cortical thickness and surface area.

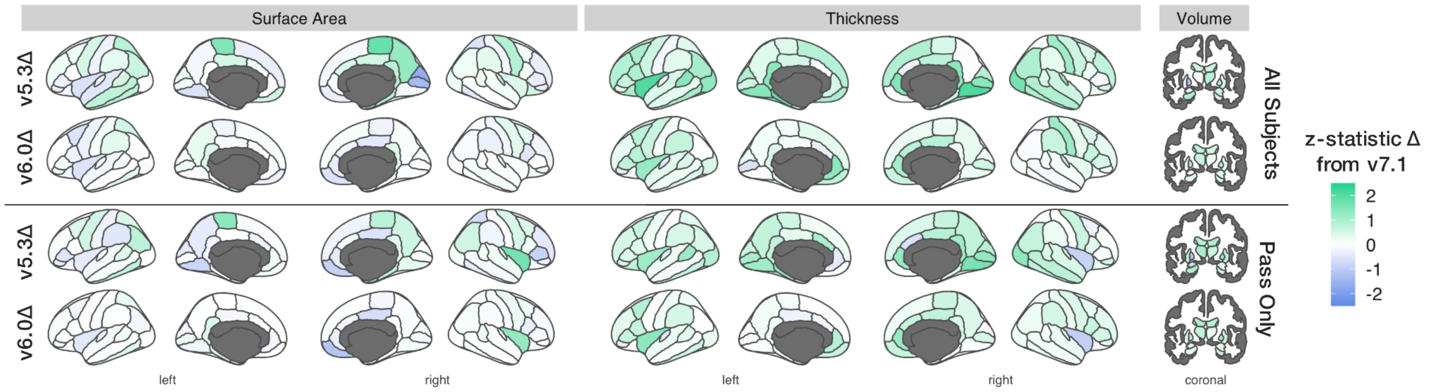

Figure S9. Difference in z-statistics (Δ) comparing v7.1 to the two previous versions.

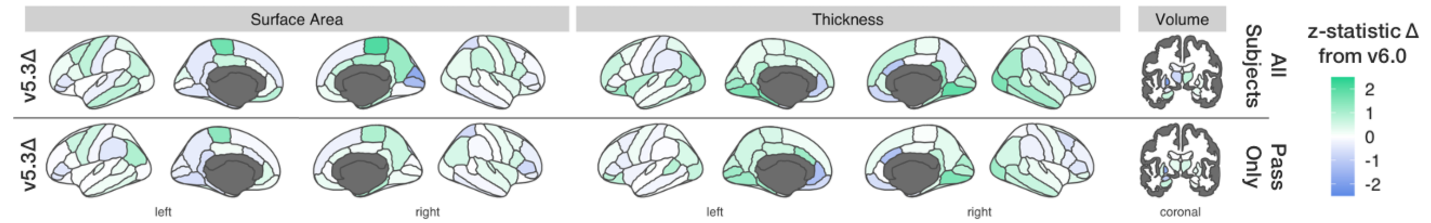

Figure S10. Difference in z-statistics (Δ) comparing v6.0 to v5.3.

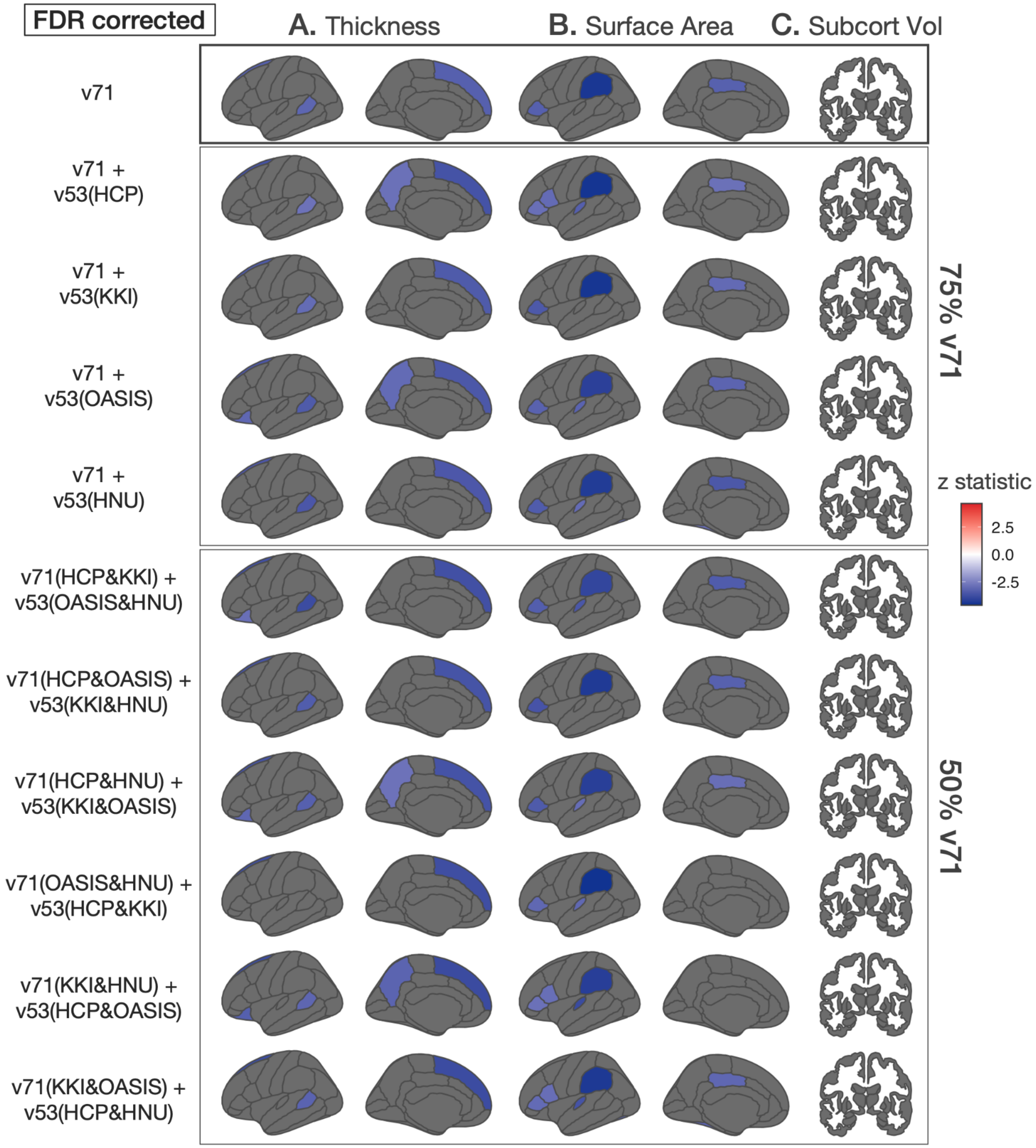

Figure S11. Harmonized v7.1 vs. harmonized v7.1 with v5.3 cohort mixtures. FDR-corrected significant z-statistics for **A.** thickness, **B.** surface area, and **C.** subcortical volume associations with age. Top box indicates the reference association results (all cohorts run through v7.1 before harmonization), middle box indicates associations after a single cohort was run through v5.3 (75% v7.1) before harmonization, and bottom box indicates association results after harmonizing two cohorts run with v5.3, and the other two with v7.1 (50% v7.1). The accumbens association with age was only significant in the v71(KKI&OASIS)+v53(HCP&HNU) comparison (z = -2.87; q = 0.04); not pictured.

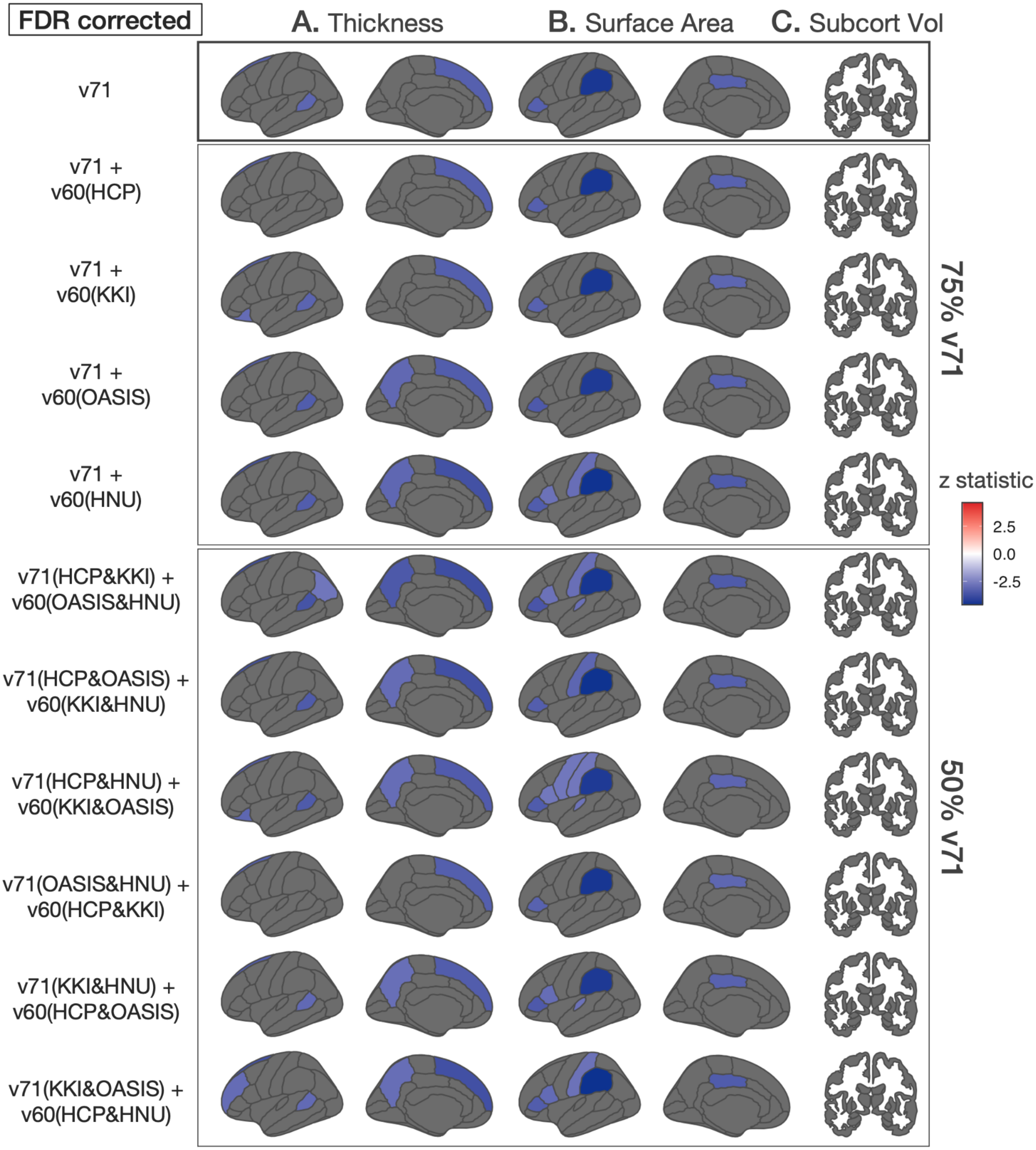

Figure S12. Harmonized v7.1 vs. harmonized v7.1 with v6.0 cohort mixtures. FDR-corrected significant z-statistics for **A.** thickness, **B.** surface area, and **C.** subcortical volume associations with age. Top box indicates the reference association results (all cohorts run through v7.1 before harmonization), middle box indicates associations after a single cohort was run through v6.0 (75% v7.1) before harmonization, and bottom box indicates association results after harmonizing two cohorts run with v6.0, and the other two with v7.1 (50% v7.1).

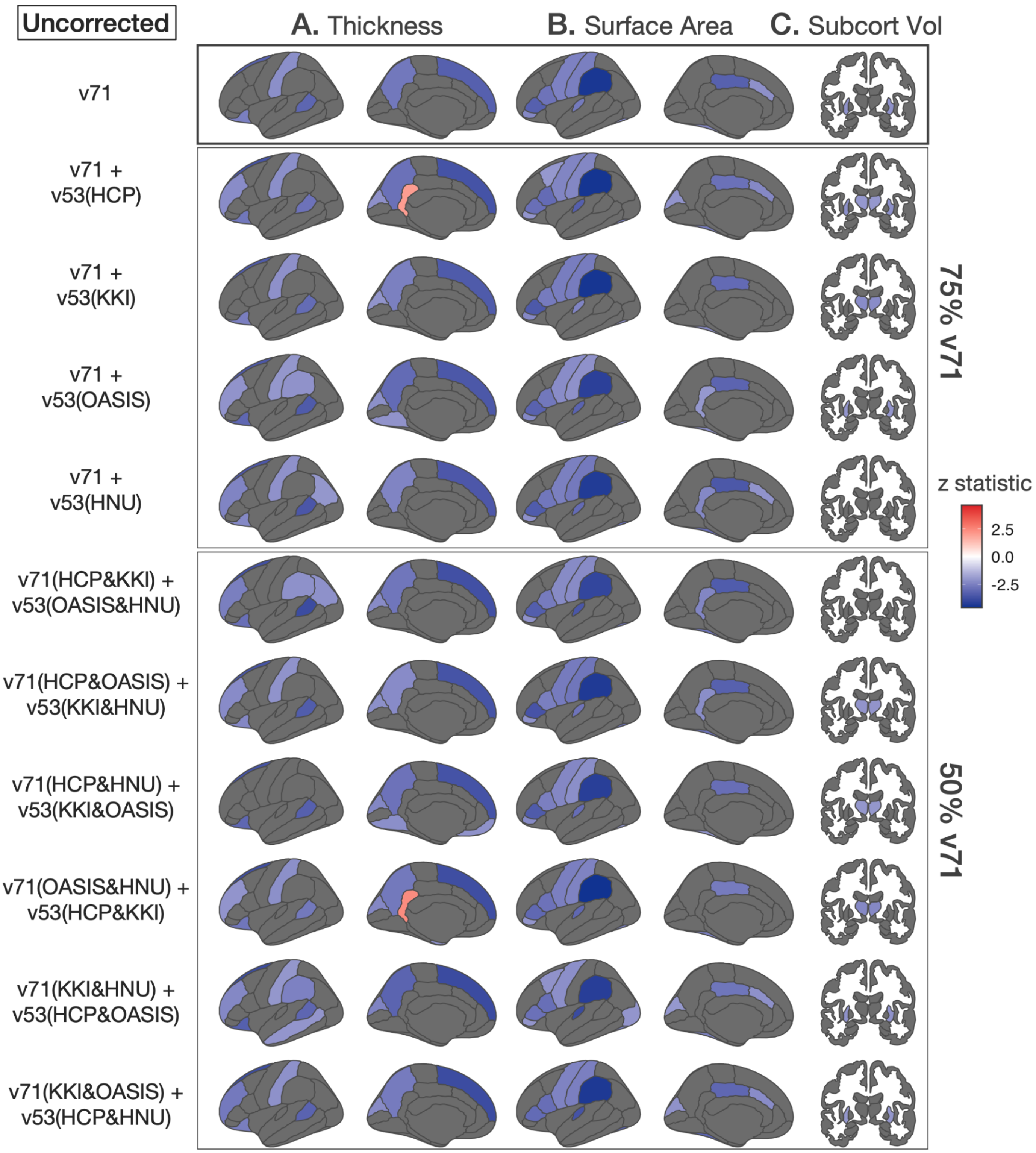

Figure S13. Harmonized v7.1 vs. harmonized v7.1 with v5.3 cohort mixtures. Uncorrected significant z-statistics for **A.** thickness, **B.** surface area, and **C.** subcortical volume associations with age. Top box indicates the reference association results (all cohorts run through v7.1 before harmonization), middle box indicates associations after a single cohort was run through v5.3 (75% v7.1) before harmonization, and bottom box indicates association results after harmonizing two cohorts run with v5.3, and the other two with v7.1 (50% v7.1). Significant associations between the accumbens volume and age were found in the v71+v53(HCP) comparison (z = -2.71; p = 0.008) and the v71(KKI&OASIS)+v53(HCP&HNU) comparison (z = -2.87; p = 0.005); not pictured.

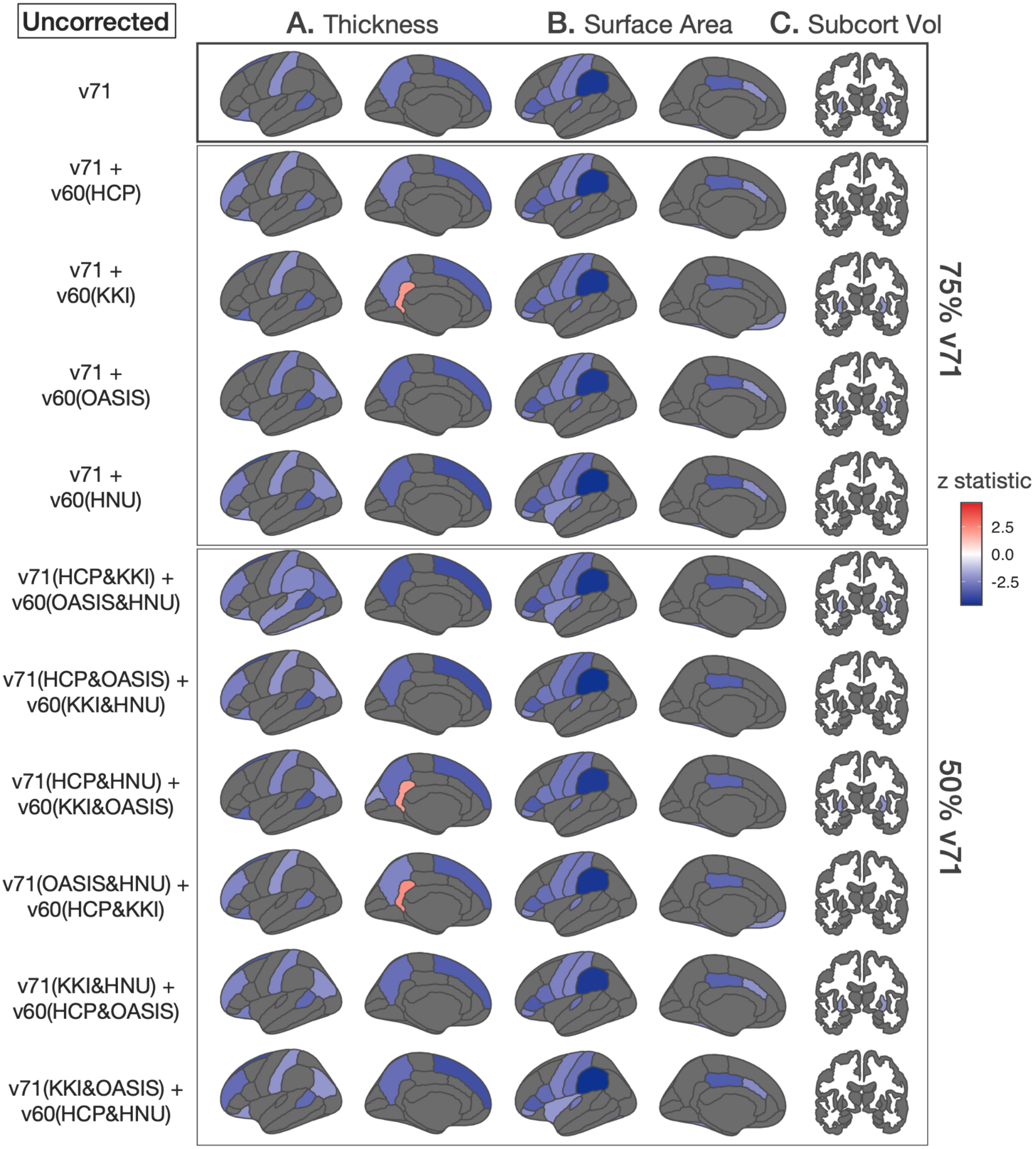

Figure S14. Harmonized v7.1 vs. harmonized v7.1 with v6.0 cohort mixtures. Uncorrected significant z-statistics for **A.** thickness, **B.** surface area, and **C.** subcortical volume associations with age. Top box indicates the reference association results (all cohorts run through v7.1 before harmonization), middle box indicates associations after a single cohort was run through v6.0 (75% v7.1) before harmonization, and bottom box indicates association results after harmonizing two cohorts run with v6.0, and the other two with v7.1 (50% v7.1). Significant associations between the accumbens volume and age were found for the v71+v60(HCP) comparison (z = -2.10; p = 0.04) and the v71(KKI&OASIS)+v60(HCP&HNU) comparison (z = -2.22; p = 0.03); not pictured.

Table S1. Runtimes for test-retest datasets across all three versions.

Cohorts		v5.3 runtime (hours)	v6.0 runtime (hours)	v7.1 runtime (hours)
HCP	Avg	6.64	8.90	6.27
	Min	5.44	6.57	5.46
	Max	14.20	11.56	7.59
KKI	Avg	7.80	9.80	6.67
	Min	6.51	7.77	5.75
	Max	9.61	11.50	7.53
OASIS	Avg	3.93	9.41	6.50
	Min	3.19	7.49	5.65
	Max	6.46	11.11	7.81

Table S2. Mean Euler numbers extracted from the nofix surface and the final surface. Parenthesis indicate the min, and max values.

Characteristic	HCP			KKI			OASIS		
	v5.3	v6.0	v7.1	v5.3	v6.0	v7.1	v5.3	v6.0	v7.1
lh.orig.nofix holes	20 (7,46)	17 (4,38)	8 (2,18)	21 (7,51)	19 (4,46)	11 (3,33)	21 (5,39)	20 (7,47)	10 (1,23)
rh.orig.nofix holes	22 (7,50)	17 (5,33)	9 (1,20)	23 (10,51)	21 (9,42)	10 (2,21)	18 (5,40)	16 (3,36)	8 (1,20)
total orig.nofix holes	42 (19,83)	34 (9,60)	17 (5,33)	44 (21,102)	40 (13,87)	20 (5,54)	40 (17,76)	36 (10,83)	18 (6,38)
lh.orig.nofix euler	-38 (-90,-12)	-31 (-74,-6)	-15 (-34,-2)	-40 (-100,-12)	-37 (-90,-6)	-19 (-64,-4)	-40 (-76,-8)	-38 (-92,-12)	-17 (-44,0)
rh.orig.nofix euler	-41 (-98,-12)	-32 (-64,-8)	-15 (-38,0)	-43 (-100,-18)	-39 (-82,-16)	-17 (-40,-2)	-35 (-78,-8)	-29 (-70,-4)	-14 (-38,0)
total orig.nofix euler	-79 (-162,-34)	-63 (-116,-14)	-30 (-62,-6)	-84 (-200,-38)	-76 (-170,-22)	-36 (-104,-6)	-75 (-148,-30)	-67 (-162,-16)	-32 (-72,-8)
lh.orig holes	0 (0,0)	0 (0,0)	0 (0,0)	0 (0,0)	0 (0,0)	0 (0,0)	0 (0,0)	0 (0,0)	0 (0,0)
rh.orig holes	0 (0,0)	0 (0,0)	0 (0,0)	0 (0,0)	0 (0,0)	0 (0,0)	0 (0,0)	0 (0,0)	0 (0,0)
total orig holes	0 (0,0)	0 (0,0)	0 (0,0)	0 (0,0)	0 (0,0)	0 (0,0)	0 (0,0)	0 (0,0)	0 (0,0)
lh.orig euler	2 (2,2)	2 (2,2)	2 (2,2)	2 (2,2)	2 (2,2)	2 (2,2)	2 (2,2)	2 (2,2)	2 (2,2)
rh.orig euler	2 (2,2)	2 (2,2)	2 (2,2)	2 (2,2)	2 (2,2)	2 (2,2)	2 (2,2)	2 (2,2)	2 (2,2)

Table S3. HCP specific ICCs and associated statistics.

ROI	Type	ICC	F	df1	df2	p	lower bound	upper bound	comparison	dataset
AVG_Accumbens	ICC3	0.9238	25.23	34	34	4.92E-16	0.869	0.956	v5.3	HCP
AVG_Accumbens	ICC2	0.8780	17.33	34	34	1.69E-13	0.781	0.931	v5.3 vs. v6.0	HCP
AVG_Accumbens	ICC2	0.8864	18.36	34	34	6.96E-14	0.799	0.936	v5.3 vs. v7.1	HCP
AVG_Accumbens	ICC3	0.8815	15.88	34	34	6.35E-13	0.799	0.931	v6.0	HCP
AVG_Accumbens	ICC2	0.9051	19.60	34	34	2.54E-14	0.838	0.945	v6.0 vs. v7.1	HCP
AVG_Accumbens	ICC3	0.8995	18.91	34	34	4.42E-14	0.829	0.942	v7.1	HCP
AVG_Amygdala	ICC3	0.9313	28.13	34	34	8.82E-17	0.881	0.961	v5.3	HCP
AVG_Amygdala	ICC2	0.7964	34.27	34	34	3.73E-18	0.033	0.932	v5.3 vs. v6.0	HCP
AVG_Amygdala	ICC2	0.8052	34.15	34	34	3.96E-18	0.049	0.934	v5.3 vs. v7.1	HCP
AVG_Amygdala	ICC3	0.9621	51.78	34	34	4.57E-21	0.934	0.978	v6.0	HCP
AVG_Amygdala	ICC2	0.9716	69.21	34	34	3.84E-23	0.950	0.984	v6.0 vs. v7.1	HCP
AVG_Amygdala	ICC3	0.9601	49.07	34	34	1.10E-20	0.930	0.977	v7.1	HCP
AVG_bankssts_thickavg	ICC3	0.9110	21.48	34	34	6.14E-15	0.848	0.949	v5.3	HCP
AVG_bankssts_thickavg	ICC2	0.8970	19.04	34	34	3.99E-14	0.825	0.941	v5.3 vs. v6.0	HCP
AVG_bankssts_thickavg	ICC2	0.7439	19.14	34	34	3.68E-14	0.059	0.904	v5.3 vs. v7.1	HCP
AVG_bankssts_thickavg	ICC3	0.9208	24.25	34	34	9.21E-16	0.864	0.955	v6.0	HCP
AVG_bankssts_thickavg	ICC2	0.8134	40.52	34	34	2.50E-19	0.036	0.939	v6.0 vs. v7.1	HCP
AVG_bankssts_thickavg	ICC3	0.9474	37.06	34	34	1.06E-18	0.909	0.970	v7.1	HCP
AVG_caudalanteriorcingulate_thickavg	ICC3	0.8251	10.44	34	34	3.01E-10	0.710	0.897	v5.3	HCP
AVG_caudalanteriorcingulate_thickavg	ICC2	0.8243	10.76	34	34	1.95E-10	0.709	0.897	v5.3 vs. v6.0	HCP
AVG_caudalanteriorcingulate_thickavg	ICC2	0.4188	7.88	34	34	1.45E-08	-0.063	0.720	v5.3 vs. v7.1	HCP
AVG_caudalanteriorcingulate_thickavg	ICC3	0.8324	10.94	34	34	1.54E-10	0.721	0.902	v6.0	HCP
AVG_caudalanteriorcingulate_thickavg	ICC2	0.3526	11.39	34	34	8.63E-11	-0.041	0.678	v6.0 vs. v7.1	HCP
AVG_caudalanteriorcingulate_thickavg	ICC3	0.8727	14.71	34	34	2.00E-12	0.785	0.926	v7.1	HCP
AVG_caudalmiddlefrontal_thickavg	ICC3	0.8967	18.35	34	34	6.99E-14	0.824	0.940	v5.3	HCP
AVG_caudalmiddlefrontal_thickavg	ICC2	0.9228	32.83	34	34	7.45E-18	0.822	0.962	v5.3 vs. v6.0	HCP
AVG_caudalmiddlefrontal_thickavg	ICC2	0.8456	25.84	34	34	3.38E-16	0.346	0.941	v5.3 vs. v7.1	HCP
AVG_caudalmiddlefrontal_thickavg	ICC3	0.9106	21.37	34	34	6.64E-15	0.847	0.949	v6.0	HCP
AVG_caudalmiddlefrontal_thickavg	ICC2	0.7716	39.54	34	34	3.71E-19	-0.006	0.925	v6.0 vs. v7.1	HCP
AVG_caudalmiddlefrontal_thickavg	ICC3	0.9396	32.09	34	34	1.07E-17	0.895	0.965	v7.1	HCP
AVG_Caudate	ICC3	0.9792	95.09	34	34	1.96E-25	0.963	0.988	v5.3	HCP
AVG_Caudate	ICC2	0.7902	75.91	34	34	8.30E-24	-0.006	0.936	v5.3 vs. v6.0	HCP
AVG_Caudate	ICC2	0.8029	52.63	34	34	3.49E-21	0.003	0.938	v5.3 vs. v7.1	HCP
AVG_Caudate	ICC3	0.9852	134.17	34	34	6.21E-28	0.974	0.992	v6.0	HCP
AVG_Caudate	ICC2	0.9846	146.05	34	34	1.50E-28	0.971	0.992	v6.0 vs. v7.1	HCP
AVG_Caudate	ICC3	0.9764	83.90	34	34	1.58E-24	0.959	0.987	v7.1	HCP
AVG_cuneus_thickavg	ICC3	0.9420	33.51	34	34	5.36E-18	0.900	0.967	v5.3	HCP
AVG_cuneus_thickavg	ICC2	0.8560	23.83	34	34	1.21E-15	0.479	0.941	v5.3 vs. v6.0	HCP
AVG_cuneus_thickavg	ICC2	0.8926	17.47	34	34	1.48E-13	0.818	0.938	v5.3 vs. v7.1	HCP
AVG_cuneus_thickavg	ICC3	0.9469	36.65	34	34	1.27E-18	0.908	0.970	v6.0	HCP

AVG_cuneus_thickavg	ICC2	0.8641	43.51	34	34	7.85E-20	0.176	0.954	v6.0 vs. v7.1	HCP
AVG_cuneus_thickavg	ICC3	0.9577	46.31	34	34	2.84E-20	0.926	0.976	v7.1	HCP
AVG_entorhinal_thickavg	ICC3	0.8403	11.52	34	34	7.30E-11	0.733	0.907	v5.3	HCP
AVG_entorhinal_thickavg	ICC2	0.9385	32.33	34	34	9.53E-18	0.894	0.965	v5.3 vs. v6.0	HCP
AVG_entorhinal_thickavg	ICC2	0.5946	16.12	34	34	5.05E-13	-0.045	0.841	v5.3 vs. v7.1	HCP
AVG_entorhinal_thickavg	ICC3	0.8510	12.42	34	34	2.44E-11	0.750	0.913	v6.0	HCP
AVG_entorhinal_thickavg	ICC2	0.6140	23.80	34	34	1.24E-15	-0.038	0.857	v6.0 vs. v7.1	HCP
AVG_entorhinal_thickavg	ICC3	0.8672	14.06	34	34	3.93E-12	0.776	0.923	v7.1	HCP
AVG_frontalpole_thickavg	ICC3	0.7934	8.68	34	34	3.94E-09	0.661	0.878	v5.3	HCP
AVG_frontalpole_thickavg	ICC2	0.7378	8.25	34	34	7.83E-09	0.515	0.855	v5.3 vs. v6.0	HCP
AVG_frontalpole_thickavg	ICC2	0.6025	9.52	34	34	1.10E-09	0.005	0.827	v5.3 vs. v7.1	HCP
AVG_frontalpole_thickavg	ICC3	0.8132	9.71	34	34	8.37E-10	0.691	0.890	v6.0	HCP
AVG_frontalpole_thickavg	ICC2	0.7795	14.69	34	34	2.05E-12	0.330	0.905	v6.0 vs. v7.1	HCP
AVG_frontalpole_thickavg	ICC3	0.8397	11.48	34	34	7.72E-11	0.732	0.906	v7.1	HCP
AVG_fusiform_thickavg	ICC3	0.8970	18.41	34	34	6.68E-14	0.824	0.941	v5.3	HCP
AVG_fusiform_thickavg	ICC2	0.9132	21.68	34	34	5.32E-15	0.852	0.950	v5.3 vs. v6.0	HCP
AVG_fusiform_thickavg	ICC2	0.7225	22.15	34	34	3.82E-15	-0.002	0.900	v5.3 vs. v7.1	HCP
AVG_fusiform_thickavg	ICC3	0.9113	21.55	34	34	5.85E-15	0.848	0.949	v6.0	HCP
AVG_fusiform_thickavg	ICC2	0.7125	60.81	34	34	3.25E-22	-0.016	0.906	v6.0 vs. v7.1	HCP
AVG_fusiform_thickavg	ICC3	0.9288	27.10	34	34	1.59E-16	0.877	0.959	v7.1	HCP
AVG_Hippocampus	ICC3	0.8290	10.69	34	34	2.13E-10	0.716	0.900	v5.3	HCP
AVG_Hippocampus	ICC2	0.9623	88.79	34	34	6.14E-25	0.849	0.985	v5.3 vs. v6.0	HCP
AVG_Hippocampus	ICC2	0.9729	74.16	34	34	1.22E-23	0.953	0.985	v5.3 vs. v7.1	HCP
AVG_Hippocampus	ICC3	0.9731	73.43	34	34	1.44E-23	0.953	0.985	v6.0	HCP
AVG_Hippocampus	ICC2	0.9713	95.15	34	34	1.94E-25	0.924	0.986	v6.0 vs. v7.1	HCP
AVG_Hippocampus	ICC3	0.9814	106.72	34	34	2.86E-26	0.967	0.989	v7.1	HCP
AVG_inferiorparietal_thickavg	ICC3	0.9507	39.57	34	34	3.66E-19	0.914	0.972	v5.3	HCP
AVG_inferiorparietal_thickavg	ICC2	0.9581	45.57	34	34	3.68E-20	0.927	0.976	v5.3 vs. v6.0	HCP
AVG_inferiorparietal_thickavg	ICC2	0.7704	27.09	34	34	1.60E-16	0.029	0.920	v5.3 vs. v7.1	HCP
AVG_inferiorparietal_thickavg	ICC3	0.9475	37.07	34	34	1.05E-18	0.909	0.970	v6.0	HCP
AVG_inferiorparietal_thickavg	ICC2	0.7816	75.87	34	34	8.38E-24	-0.007	0.933	v6.0 vs. v7.1	HCP
AVG_inferiorparietal_thickavg	ICC3	0.9547	43.13	34	34	9.04E-20	0.921	0.974	v7.1	HCP
AVG_inferiortemporal_thickavg	ICC3	0.8988	18.76	34	34	5.01E-14	0.827	0.942	v5.3	HCP
AVG_inferiortemporal_thickavg	ICC2	0.9156	35.36	34	34	2.26E-18	0.740	0.963	v5.3 vs. v6.0	HCP
AVG_inferiortemporal_thickavg	ICC2	0.5947	24.44	34	34	8.15E-16	-0.037	0.848	v5.3 vs. v7.1	HCP
AVG_inferiortemporal_thickavg	ICC3	0.9080	20.75	34	34	1.06E-14	0.843	0.947	v6.0	HCP
AVG_inferiortemporal_thickavg	ICC2	0.7107	41.36	34	34	1.79E-19	-0.023	0.903	v6.0 vs. v7.1	HCP
AVG_inferiortemporal_thickavg	ICC3	0.9202	24.07	34	34	1.03E-15	0.863	0.954	v7.1	HCP
AVG_insula_thickavg	ICC3	0.8566	12.95	34	34	1.33E-11	0.759	0.916	v5.3	HCP
AVG_insula_thickavg	ICC2	0.8325	12.11	34	34	3.56E-11	0.711	0.904	v5.3 vs. v6.0	HCP
AVG_insula_thickavg	ICC2	0.7952	16.65	34	34	3.08E-13	0.329	0.914	v5.3 vs. v7.1	HCP
AVG_insula_thickavg	ICC3	0.8602	13.31	34	34	8.90E-12	0.765	0.919	v6.0	HCP
AVG_insula_thickavg	ICC2	0.6638	10.09	34	34	4.84E-10	0.104	0.852	v6.0 vs. v7.1	HCP
AVG_insula_thickavg	ICC3	0.8847	16.34	34	34	4.11E-13	0.804	0.933	v7.1	HCP

AVG_isthmuscingulate_thickavg	ICC3	0.8600	13.29	34	34	9.09E-12	0.765	0.919	v5.3	HCP
AVG_isthmuscingulate_thickavg	ICC2	0.6365	7.49	34	34	2.87E-08	0.186	0.823	v5.3 vs. v6.0	HCP
AVG_isthmuscingulate_thickavg	ICC2	0.2349	7.71	34	34	1.95E-08	-0.038	0.546	v5.3 vs. v7.1	HCP
AVG_isthmuscingulate_thickavg	ICC3	0.8625	13.55	34	34	6.81E-12	0.769	0.920	v6.0	HCP
AVG_isthmuscingulate_thickavg	ICC2	0.3705	12.14	34	34	3.41E-11	-0.041	0.694	v6.0 vs. v7.1	HCP
AVG_isthmuscingulate_thickavg	ICC3	0.8945	17.95	34	34	9.82E-14	0.820	0.939	v7.1	HCP
AVG_lateraloccipital_thickavg	ICC3	0.9694	64.30	34	34	1.29E-22	0.946	0.983	v5.3	HCP
AVG_lateraloccipital_thickavg	ICC2	0.9553	46.51	34	34	2.64E-20	0.921	0.975	v5.3 vs. v6.0	HCP
AVG_lateraloccipital_thickavg	ICC2	0.8591	30.42	34	34	2.53E-17	0.336	0.948	v5.3 vs. v7.1	HCP
AVG_lateraloccipital_thickavg	ICC3	0.9452	35.49	34	34	2.12E-18	0.905	0.969	v6.0	HCP
AVG_lateraloccipital_thickavg	ICC2	0.8471	103.38	34	34	4.87E-26	0.014	0.955	v6.0 vs. v7.1	HCP
AVG_lateraloccipital_thickavg	ICC3	0.9674	60.37	34	34	3.67E-22	0.943	0.981	v7.1	HCP
AVG_lateralorbitofrontal_thickavg	ICC3	0.8960	18.24	34	34	7.70E-14	0.823	0.940	v5.3	HCP
AVG_lateralorbitofrontal_thickavg	ICC2	0.8975	25.08	34	34	5.41E-16	0.758	0.949	v5.3 vs. v6.0	HCP
AVG_lateralorbitofrontal_thickavg	ICC2	0.7570	15.10	34	34	1.36E-12	0.204	0.900	v5.3 vs. v7.1	HCP
AVG_lateralorbitofrontal_thickavg	ICC3	0.8992	18.85	34	34	4.66E-14	0.828	0.942	v6.0	HCP
AVG_lateralorbitofrontal_thickavg	ICC2	0.6804	31.21	34	34	1.68E-17	-0.030	0.889	v6.0 vs. v7.1	HCP
AVG_lateralorbitofrontal_thickavg	ICC3	0.8616	13.45	34	34	7.59E-12	0.767	0.919	v7.1	HCP
AVG_LateralVentricle	ICC3	0.9951	409.70	34	34	4.18E-36	0.991	0.997	v5.3	HCP
AVG_LateralVentricle	ICC2	0.9722	653.89	34	34	1.52E-39	0.254	0.993	v5.3 vs. v6.0	HCP
AVG_LateralVentricle	ICC2	0.9691	482.71	34	34	2.60E-37	0.270	0.992	v5.3 vs. v7.1	HCP
AVG_LateralVentricle	ICC3	0.9959	485.34	34	34	2.37E-37	0.993	0.998	v6.0	HCP
AVG_LateralVentricle	ICC2	0.9996	5694.19	34	34	1.67E-55	0.999	1.000	v6.0 vs. v7.1	HCP
AVG_LateralVentricle	ICC3	0.9964	552.86	34	34	2.61E-38	0.994	0.998	v7.1	HCP
AVG_lingual_thickavg	ICC3	0.9380	31.23	34	34	1.66E-17	0.893	0.965	v5.3	HCP
AVG_lingual_thickavg	ICC2	0.8688	19.85	34	34	2.09E-14	0.685	0.936	v5.3 vs. v6.0	HCP
AVG_lingual_thickavg	ICC2	0.7674	14.78	34	34	1.86E-12	0.265	0.902	v5.3 vs. v7.1	HCP
AVG_lingual_thickavg	ICC3	0.8910	17.35	34	34	1.66E-13	0.815	0.937	v6.0	HCP
AVG_lingual_thickavg	ICC2	0.6925	51.27	34	34	5.37E-21	-0.019	0.898	v6.0 vs. v7.1	HCP
AVG_lingual_thickavg	ICC3	0.9307	27.84	34	34	1.04E-16	0.880	0.960	v7.1	HCP
AVG_medialorbitofrontal_thickavg	ICC3	0.7621	7.41	34	34	3.34E-08	0.614	0.858	v5.3	HCP
AVG_medialorbitofrontal_thickavg	ICC2	0.7982	13.89	34	34	4.72E-12	0.475	0.906	v5.3 vs. v6.0	HCP
AVG_medialorbitofrontal_thickavg	ICC2	0.7975	13.37	34	34	8.26E-12	0.497	0.903	v5.3 vs. v7.1	HCP
AVG_medialorbitofrontal_thickavg	ICC3	0.8765	15.20	34	34	1.23E-12	0.791	0.928	v6.0	HCP
AVG_medialorbitofrontal_thickavg	ICC2	0.6251	16.78	34	34	2.76E-13	-0.040	0.856	v6.0 vs. v7.1	HCP
AVG_medialorbitofrontal_thickavg	ICC3	0.8553	12.82	34	34	1.54E-11	0.757	0.916	v7.1	HCP
AVG_middletemporal_thickavg	ICC3	0.9424	33.71	34	34	4.87E-18	0.900	0.967	v5.3	HCP
AVG_middletemporal_thickavg	ICC2	0.9215	59.67	34	34	4.44E-22	0.496	0.972	v5.3 vs. v6.0	HCP
AVG_middletemporal_thickavg	ICC2	0.6198	41.24	34	34	1.87E-19	-0.023	0.865	v5.3 vs. v7.1	HCP
AVG_middletemporal_thickavg	ICC3	0.9505	39.37	34	34	3.97E-19	0.914	0.972	v6.0	HCP
AVG_middletemporal_thickavg	ICC2	0.7677	100.18	34	34	8.22E-26	-0.005	0.929	v6.0 vs. v7.1	HCP
AVG_middletemporal_thickavg	ICC3	0.9610	50.32	34	34	7.29E-21	0.932	0.978	v7.1	HCP
AVG_Pallidum	ICC3	0.8373	11.29	34	34	9.72E-11	0.729	0.905	v5.3	HCP
AVG_Pallidum	ICC2	0.1837	4.89	34	34	5.83E-06	-0.045	0.465	v5.3 vs. v6.0	HCP

AVG_Pallidum	ICC2	0.1801	5.98	34	34	5.20E-07	-0.036	0.463	v5.3 vs. v7.1	HCP
AVG_Pallidum	ICC3	0.8772	15.28	34	34	1.13E-12	0.792	0.929	v6.0	HCP
AVG_Pallidum	ICC2	0.8918	19.51	34	34	2.73E-14	0.807	0.939	v6.0 vs. v7.1	HCP
AVG_Pallidum	ICC3	0.9480	37.46	34	34	8.91E-19	0.910	0.970	v7.1	HCP
AVG_paracentral_thickavg	ICC3	0.9573	45.84	34	34	3.35E-20	0.926	0.976	v5.3	HCP
AVG_paracentral_thickavg	ICC2	0.8812	21.85	34	34	4.73E-15	0.716	0.942	v5.3 vs. v6.0	HCP
AVG_paracentral_thickavg	ICC2	0.9392	32.00	34	34	1.12E-17	0.895	0.965	v5.3 vs. v7.1	HCP
AVG_paracentral_thickavg	ICC3	0.9484	37.76	34	34	7.81E-19	0.910	0.971	v6.0	HCP
AVG_paracentral_thickavg	ICC2	0.9538	77.08	34	34	6.44E-24	0.792	0.982	v6.0 vs. v7.1	HCP
AVG_paracentral_thickavg	ICC3	0.9537	42.15	34	34	1.31E-19	0.919	0.974	v7.1	HCP
AVG_parahippocampal_thickavg	ICC3	0.9431	34.18	34	34	3.91E-18	0.901	0.968	v5.3	HCP
AVG_parahippocampal_thickavg	ICC2	0.9816	104.63	34	34	3.98E-26	0.968	0.990	v5.3 vs. v6.0	HCP
AVG_parahippocampal_thickavg	ICC2	0.8069	35.49	34	34	2.13E-18	0.046	0.935	v5.3 vs. v7.1	HCP
AVG_parahippocampal_thickavg	ICC3	0.9603	49.32	34	34	1.01E-20	0.931	0.977	v6.0	HCP
AVG_parahippocampal_thickavg	ICC2	0.8229	87.13	34	34	8.42E-25	0.004	0.947	v6.0 vs. v7.1	HCP
AVG_parahippocampal_thickavg	ICC3	0.9597	48.67	34	34	1.26E-20	0.930	0.977	v7.1	HCP
AVG_parsopercularis_thickavg	ICC3	0.9016	19.32	34	34	3.17E-14	0.832	0.943	v5.3	HCP
AVG_parsopercularis_thickavg	ICC2	0.8910	37.77	34	34	7.78E-19	0.462	0.959	v5.3 vs. v6.0	HCP
AVG_parsopercularis_thickavg	ICC2	0.9210	28.69	34	34	6.42E-17	0.847	0.957	v5.3 vs. v7.1	HCP
AVG_parsopercularis_thickavg	ICC3	0.9210	24.33	34	34	8.75E-16	0.864	0.955	v6.0	HCP
AVG_parsopercularis_thickavg	ICC2	0.8197	35.36	34	34	2.26E-18	0.076	0.939	v6.0 vs. v7.1	HCP
AVG_parsopercularis_thickavg	ICC3	0.9299	27.53	34	34	1.24E-16	0.879	0.960	v7.1	HCP
AVG_parsorbitalis_thickavg	ICC3	0.9058	20.22	34	34	1.57E-14	0.839	0.946	v5.3	HCP
AVG_parsorbitalis_thickavg	ICC2	0.9356	29.72	34	34	3.66E-17	0.889	0.963	v5.3 vs. v6.0	HCP
AVG_parsorbitalis_thickavg	ICC2	0.6764	14.32	34	34	3.00E-12	0.013	0.872	v5.3 vs. v7.1	HCP
AVG_parsorbitalis_thickavg	ICC3	0.9133	22.08	34	34	4.00E-15	0.851	0.950	v6.0	HCP
AVG_parsorbitalis_thickavg	ICC2	0.7154	27.13	34	34	1.57E-16	-0.021	0.901	v6.0 vs. v7.1	HCP
AVG_parsorbitalis_thickavg	ICC3	0.9085	20.86	34	34	9.67E-15	0.843	0.947	v7.1	HCP
AVG_parstriangularis_thickavg	ICC3	0.8680	14.15	34	34	3.57E-12	0.777	0.923	v5.3	HCP
AVG_parstriangularis_thickavg	ICC2	0.9156	27.62	34	34	1.18E-16	0.830	0.955	v5.3 vs. v6.0	HCP
AVG_parstriangularis_thickavg	ICC2	0.7929	17.09	34	34	2.08E-13	0.299	0.915	v5.3 vs. v7.1	HCP
AVG_parstriangularis_thickavg	ICC3	0.9047	20.00	34	34	1.87E-14	0.837	0.945	v6.0	HCP
AVG_parstriangularis_thickavg	ICC2	0.7525	33.87	34	34	4.51E-18	-0.010	0.917	v6.0 vs. v7.1	HCP
AVG_parstriangularis_thickavg	ICC3	0.9259	25.98	34	34	3.11E-16	0.872	0.957	v7.1	HCP
AVG_pericalcarine_thickavg	ICC3	0.8964	18.31	34	34	7.24E-14	0.824	0.940	v5.3	HCP
AVG_pericalcarine_thickavg	ICC2	0.8006	14.45	34	34	2.62E-12	0.461	0.908	v5.3 vs. v6.0	HCP
AVG_pericalcarine_thickavg	ICC2	0.8446	11.56	34	34	6.93E-11	0.740	0.909	v5.3 vs. v7.1	HCP
AVG_pericalcarine_thickavg	ICC3	0.8758	15.11	34	34	1.34E-12	0.790	0.928	v6.0	HCP
AVG_pericalcarine_thickavg	ICC2	0.8555	25.64	34	34	3.83E-16	0.424	0.943	v6.0 vs. v7.1	HCP
AVG_pericalcarine_thickavg	ICC3	0.9238	25.26	34	34	4.83E-16	0.869	0.956	v7.1	HCP
AVG_postcentral_thickavg	ICC3	0.9773	87.22	34	34	8.27E-25	0.960	0.987	v5.3	HCP
AVG_postcentral_thickavg	ICC2	0.9066	56.77	34	34	1.01E-21	0.357	0.968	v5.3 vs. v6.0	HCP
AVG_postcentral_thickavg	ICC2	0.9492	37.78	34	34	7.74E-19	0.912	0.971	v5.3 vs. v7.1	HCP
AVG_postcentral_thickavg	ICC3	0.9629	52.90	34	34	3.21E-21	0.935	0.979	v6.0	HCP

AVG_postcentral_thickavg	ICC2	0.8879	30.33	34	34	2.66E-17	0.581	0.954	v6.0 vs. v7.1	HCP
AVG_postcentral_thickavg	ICC3	0.9587	47.42	34	34	1.92E-20	0.928	0.976	v7.1	HCP
AVG_posteriorcingulate_thickavg	ICC3	0.8809	15.79	34	34	6.90E-13	0.798	0.931	v5.3	HCP
AVG_posteriorcingulate_thickavg	ICC2	0.8433	14.70	34	34	2.02E-12	0.687	0.917	v5.3 vs. v6.0	HCP
AVG_posteriorcingulate_thickavg	ICC2	0.3597	15.12	34	34	1.33E-12	-0.032	0.688	v5.3 vs. v7.1	HCP
AVG_posteriorcingulate_thickavg	ICC3	0.9094	21.08	34	34	8.25E-15	0.845	0.948	v6.0	HCP
AVG_posteriorcingulate_thickavg	ICC2	0.4682	20.35	34	34	1.43E-14	-0.033	0.774	v6.0 vs. v7.1	HCP
AVG_posteriorcingulate_thickavg	ICC3	0.9324	28.58	34	34	6.84E-17	0.883	0.961	v7.1	HCP
AVG_precentral_thickavg	ICC3	0.9377	31.11	34	34	1.77E-17	0.892	0.964	v5.3	HCP
AVG_precentral_thickavg	ICC2	0.9010	57.12	34	34	9.12E-22	0.300	0.967	v5.3 vs. v6.0	HCP
AVG_precentral_thickavg	ICC2	0.9395	38.14	34	34	6.64E-19	0.880	0.968	v5.3 vs. v7.1	HCP
AVG_precentral_thickavg	ICC3	0.9080	20.75	34	34	1.05E-14	0.843	0.947	v6.0	HCP
AVG_precentral_thickavg	ICC2	0.8251	37.67	34	34	8.12E-19	0.074	0.942	v6.0 vs. v7.1	HCP
AVG_precentral_thickavg	ICC3	0.9091	21.00	34	34	8.75E-15	0.844	0.948	v7.1	HCP
AVG_precuneus_thickavg	ICC3	0.9415	33.21	34	34	6.20E-18	0.899	0.967	v5.3	HCP
AVG_precuneus_thickavg	ICC2	0.8346	14.12	34	34	3.68E-12	0.664	0.913	v5.3 vs. v6.0	HCP
AVG_precuneus_thickavg	ICC2	0.8387	15.43	34	34	9.83E-13	0.641	0.919	v5.3 vs. v7.1	HCP
AVG_precuneus_thickavg	ICC3	0.9275	26.60	34	34	2.13E-16	0.875	0.958	v6.0	HCP
AVG_precuneus_thickavg	ICC2	0.7785	51.49	34	34	5.00E-21	-0.009	0.929	v6.0 vs. v7.1	HCP
AVG_precuneus_thickavg	ICC3	0.9491	38.29	34	34	6.23E-19	0.912	0.971	v7.1	HCP
AVG_Putamen	ICC3	0.9260	26.03	34	34	3.02E-16	0.873	0.958	v5.3	HCP
AVG_Putamen	ICC2	0.7012	18.76	34	34	5.01E-14	-0.003	0.889	v5.3 vs. v6.0	HCP
AVG_Putamen	ICC2	0.7762	22.32	34	34	3.39E-15	0.091	0.918	v5.3 vs. v7.1	HCP
AVG_Putamen	ICC3	0.9221	24.67	34	34	7.01E-16	0.866	0.955	v6.0	HCP
AVG_Putamen	ICC2	0.9335	34.66	34	34	3.11E-18	0.868	0.964	v6.0 vs. v7.1	HCP
AVG_Putamen	ICC3	0.9695	64.67	34	34	1.18E-22	0.947	0.983	v7.1	HCP
AVG_rostralanteriorcingulate_thickavg	ICC3	0.7937	8.69	34	34	3.85E-09	0.661	0.878	v5.3	HCP
AVG_rostralanteriorcingulate_thickavg	ICC2	0.6772	8.43	34	34	5.90E-09	0.253	0.844	v5.3 vs. v6.0	HCP
AVG_rostralanteriorcingulate_thickavg	ICC2	0.6685	11.70	34	34	5.86E-11	0.055	0.861	v5.3 vs. v7.1	HCP
AVG_rostralanteriorcingulate_thickavg	ICC3	0.7899	8.52	34	34	5.07E-09	0.656	0.876	v6.0	HCP
AVG_rostralanteriorcingulate_thickavg	ICC2	0.4465	13.12	34	34	1.09E-11	-0.047	0.753	v6.0 vs. v7.1	HCP
AVG_rostralanteriorcingulate_thickavg	ICC3	0.8678	14.13	34	34	3.66E-12	0.777	0.923	v7.1	HCP
AVG_rostralmiddlefrontal_thickavg	ICC3	0.8575	13.03	34	34	1.21E-11	0.761	0.917	v5.3	HCP
AVG_rostralmiddlefrontal_thickavg	ICC2	0.8903	19.60	34	34	2.55E-14	0.800	0.939	v5.3 vs. v6.0	HCP
AVG_rostralmiddlefrontal_thickavg	ICC2	0.6766	14.63	34	34	2.17E-12	0.008	0.873	v5.3 vs. v7.1	HCP
AVG_rostralmiddlefrontal_thickavg	ICC3	0.8597	13.26	34	34	9.40E-12	0.764	0.918	v6.0	HCP
AVG_rostralmiddlefrontal_thickavg	ICC2	0.6456	32.23	34	34	1.00E-17	-0.030	0.874	v6.0 vs. v7.1	HCP
AVG_rostralmiddlefrontal_thickavg	ICC3	0.9139	22.22	34	34	3.63E-15	0.852	0.950	v7.1	HCP
AVG_superiorfrontal_thickavg	ICC3	0.9232	25.06	34	34	5.49E-16	0.868	0.956	v5.3	HCP
AVG_superiorfrontal_thickavg	ICC2	0.9691	74.54	34	34	1.12E-23	0.939	0.983	v5.3 vs. v6.0	HCP
AVG_superiorfrontal_thickavg	ICC2	0.8267	39.91	34	34	3.19E-19	0.066	0.943	v5.3 vs. v7.1	HCP
AVG_superiorfrontal_thickavg	ICC3	0.9310	27.98	34	34	9.60E-17	0.881	0.960	v6.0	HCP
AVG_superiorfrontal_thickavg	ICC2	0.8834	77.89	34	34	5.42E-24	0.090	0.964	v6.0 vs. v7.1	HCP
AVG_superiorfrontal_thickavg	ICC3	0.9473	36.92	34	34	1.12E-18	0.908	0.970	v7.1	HCP

AVG_superiorparietal_thickavg	ICC3	0.9704	66.59	34	34	7.26E-23	0.948	0.983	v5.3	HCP
AVG_superiorparietal_thickavg	ICC2	0.9133	70.52	34	34	2.82E-23	0.303	0.972	v5.3 vs. v6.0	HCP
AVG_superiorparietal_thickavg	ICC2	0.9572	55.29	34	34	1.56E-21	0.912	0.978	v5.3 vs. v7.1	HCP
AVG_superiorparietal_thickavg	ICC3	0.9606	49.76	34	34	8.75E-21	0.931	0.978	v6.0	HCP
AVG_superiorparietal_thickavg	ICC2	0.8713	89.82	34	34	5.07E-25	0.044	0.962	v6.0 vs. v7.1	HCP
AVG_superiorparietal_thickavg	ICC3	0.9582	46.82	34	34	2.37E-20	0.927	0.976	v7.1	HCP
AVG_superiortemporal_thickavg	ICC3	0.9283	26.91	34	34	1.78E-16	0.876	0.959	v5.3	HCP
AVG_superiortemporal_thickavg	ICC2	0.9417	32.92	34	34	7.13E-18	0.899	0.967	v5.3 vs. v6.0	HCP
AVG_superiortemporal_thickavg	ICC2	0.7397	29.31	34	34	4.57E-17	-0.011	0.910	v5.3 vs. v7.1	HCP
AVG_superiortemporal_thickavg	ICC3	0.9399	32.27	34	34	9.83E-18	0.896	0.966	v6.0	HCP
AVG_superiortemporal_thickavg	ICC2	0.7307	72.56	34	34	1.76E-23	-0.012	0.914	v6.0 vs. v7.1	HCP
AVG_superiortemporal_thickavg	ICC3	0.9354	29.98	34	34	3.19E-17	0.888	0.963	v7.1	HCP
AVG_supramarginal_thickavg	ICC3	0.9339	29.27	34	34	4.67E-17	0.886	0.962	v5.3	HCP
AVG_supramarginal_thickavg	ICC2	0.9439	33.94	34	34	4.36E-18	0.903	0.968	v5.3 vs. v6.0	HCP
AVG_supramarginal_thickavg	ICC2	0.7948	25.82	34	34	3.43E-16	0.096	0.927	v5.3 vs. v7.1	HCP
AVG_supramarginal_thickavg	ICC3	0.9485	37.84	34	34	7.56E-19	0.911	0.971	v6.0	HCP
AVG_supramarginal_thickavg	ICC2	0.8073	61.98	34	34	2.38E-22	0.001	0.940	v6.0 vs. v7.1	HCP
AVG_supramarginal_thickavg	ICC3	0.9573	45.81	34	34	3.39E-20	0.926	0.976	v7.1	HCP
AVG_temporalpole_thickavg	ICC3	0.8067	9.35	34	34	1.41E-09	0.681	0.886	v5.3	HCP
AVG_temporalpole_thickavg	ICC2	0.8787	22.88	34	34	2.29E-15	0.676	0.943	v5.3 vs. v6.0	HCP
AVG_temporalpole_thickavg	ICC2	0.6544	15.43	34	34	9.81E-13	-0.021	0.866	v5.3 vs. v7.1	HCP
AVG_temporalpole_thickavg	ICC3	0.9078	20.70	34	34	1.09E-14	0.842	0.947	v6.0	HCP
AVG_temporalpole_thickavg	ICC2	0.8254	30.06	34	34	3.05E-17	0.148	0.938	v6.0 vs. v7.1	HCP
AVG_temporalpole_thickavg	ICC3	0.8522	12.53	34	34	2.15E-11	0.752	0.914	v7.1	HCP
AVG_Thalamus	ICC3	0.9109	21.44	34	34	6.32E-15	0.847	0.949	v5.3	HCP
AVG_Thalamus	ICC2	0.8853	23.23	34	34	1.81E-15	0.714	0.945	v5.3 vs. v6.0	HCP
AVG_Thalamus	ICC2	0.8581	16.87	34	34	2.54E-13	0.701	0.926	v5.3 vs. v7.1	HCP
AVG_Thalamus	ICC3	0.8809	15.80	34	34	6.87E-13	0.798	0.931	v6.0	HCP
AVG_Thalamus	ICC2	0.8925	17.17	34	34	1.94E-13	0.817	0.938	v6.0 vs. v7.1	HCP
AVG_Thalamus	ICC3	0.9130	21.98	34	34	4.28E-15	0.851	0.950	v7.1	HCP
AVG_Thickness	ICC3	0.9475	37.11	34	34	1.04E-18	0.909	0.970	v5.3	HCP
AVG_Thickness	ICC2	0.9580	62.54	34	34	2.05E-22	0.896	0.980	v5.3 vs. v6.0	HCP
AVG_Thickness	ICC2	0.7758	39.99	34	34	3.09E-19	-0.003	0.927	v5.3 vs. v7.1	HCP
AVG_Thickness	ICC3	0.9464	36.29	34	34	1.48E-18	0.907	0.969	v6.0	HCP
AVG_Thickness	ICC2	0.7089	90.94	34	34	4.13E-25	-0.009	0.907	v6.0 vs. v7.1	HCP
AVG_Thickness	ICC3	0.9498	38.86	34	34	4.92E-19	0.913	0.971	v7.1	HCP
AVG_transversetemporal_thickavg	ICC3	0.7531	7.10	34	34	5.81E-08	0.601	0.853	v5.3	HCP
AVG_transversetemporal_thickavg	ICC2	0.8865	17.35	34	34	1.66E-13	0.807	0.934	v5.3 vs. v6.0	HCP
AVG_transversetemporal_thickavg	ICC2	0.7809	9.01	34	34	2.35E-09	0.630	0.873	v5.3 vs. v7.1	HCP
AVG_transversetemporal_thickavg	ICC3	0.9035	19.74	34	34	2.29E-14	0.835	0.944	v6.0	HCP
AVG_transversetemporal_thickavg	ICC2	0.8692	28.30	34	34	7.97E-17	0.465	0.948	v6.0 vs. v7.1	HCP
AVG_transversetemporal_thickavg	ICC3	0.8946	17.97	34	34	9.69E-14	0.820	0.939	v7.1	HCP
ICV	ICC3	0.9969	644.77	34	34	1.93E-39	0.995	0.998	v5.3	HCP
ICV	ICC2	1.0000	1.56E+05	34	34	6.14E-80	1.000	1.000	v5.3 vs. v6.0	HCP

ICV	ICC2	0.9569	53.62	34	34	2.58E-21	0.915	0.977	v5.3 vs. v7.1	HCP
ICV	ICC3	0.9963	532.38	34	34	4.95E-38	0.993	0.998	v6.0	HCP
ICV	ICC2	0.9577	54.67	34	34	1.87E-21	0.916	0.977	v6.0 vs. v7.1	HCP
ICV	ICC3	0.9524	41.03	34	34	2.03E-19	0.917	0.973	v7.1	HCP
LH_Accumbens	ICC3	0.8025	9.13	34	34	1.97E-09	0.675	0.884	v5.3	HCP
LH_Accumbens	ICC2	0.7795	8.89	34	34	2.85E-09	0.630	0.871	v5.3 vs. v6.0	HCP
LH_Accumbens	ICC2	0.7612	9.06	34	34	2.20E-09	0.559	0.868	v5.3 vs. v7.1	HCP
LH_Accumbens	ICC3	0.7983	8.91	34	34	2.74E-09	0.668	0.881	v6.0	HCP
LH_Accumbens	ICC2	0.8086	9.37	34	34	1.38E-09	0.685	0.887	v6.0 vs. v7.1	HCP
LH_Accumbens	ICC3	0.8078	9.40	34	34	1.30E-09	0.683	0.887	v7.1	HCP
LH_Amygdala	ICC3	0.9330	28.84	34	34	5.93E-17	0.884	0.962	v5.3	HCP
LH_Amygdala	ICC2	0.8981	38.15	34	34	6.61E-19	0.526	0.961	v5.3 vs. v6.0	HCP
LH_Amygdala	ICC2	0.8528	17.88	34	34	1.04E-13	0.642	0.929	v5.3 vs. v7.1	HCP
LH_Amygdala	ICC3	0.9466	36.45	34	34	1.38E-18	0.907	0.970	v6.0	HCP
LH_Amygdala	ICC2	0.9493	37.49	34	34	8.79E-19	0.912	0.971	v6.0 vs. v7.1	HCP
LH_Amygdala	ICC3	0.9464	36.29	34	34	1.48E-18	0.907	0.969	v7.1	HCP
LH_bankssts_surfavg	ICC3	0.9695	64.55	34	34	1.21E-22	0.947	0.983	v5.3	HCP
LH_bankssts_surfavg	ICC2	0.7913	9.98	34	34	5.70E-10	0.631	0.882	v5.3 vs. v6.0	HCP
LH_bankssts_surfavg	ICC2	0.7646	8.19	34	34	8.67E-09	0.610	0.862	v5.3 vs. v7.1	HCP
LH_bankssts_surfavg	ICC3	0.9729	72.82	34	34	1.65E-23	0.952	0.985	v6.0	HCP
LH_bankssts_surfavg	ICC2	0.9630	52.02	34	34	4.23E-21	0.935	0.979	v6.0 vs. v7.1	HCP
LH_bankssts_surfavg	ICC3	0.9603	49.34	34	34	1.01E-20	0.931	0.977	v7.1	HCP
LH_bankssts_thickavg	ICC3	0.8585	13.14	34	34	1.07E-11	0.762	0.918	v5.3	HCP
LH_bankssts_thickavg	ICC2	0.8711	14.85	34	34	1.75E-12	0.783	0.925	v5.3 vs. v6.0	HCP
LH_bankssts_thickavg	ICC2	0.7669	17.17	34	34	1.94E-13	0.173	0.908	v5.3 vs. v7.1	HCP
LH_bankssts_thickavg	ICC3	0.8922	17.56	34	34	1.38E-13	0.817	0.938	v6.0	HCP
LH_bankssts_thickavg	ICC2	0.8359	35.78	34	34	1.87E-18	0.124	0.944	v6.0 vs. v7.1	HCP
LH_bankssts_thickavg	ICC3	0.9101	21.25	34	34	7.27E-15	0.846	0.948	v7.1	HCP
LH_caudalanteriorcingulate_surfavg	ICC3	0.9636	54.02	34	34	2.28E-21	0.936	0.979	v5.3	HCP
LH_caudalanteriorcingulate_surfavg	ICC2	0.8955	25.77	34	34	3.52E-16	0.733	0.950	v5.3 vs. v6.0	HCP
LH_caudalanteriorcingulate_surfavg	ICC2	0.9100	20.76	34	34	1.05E-14	0.846	0.948	v5.3 vs. v7.1	HCP
LH_caudalanteriorcingulate_surfavg	ICC3	0.9847	129.54	34	34	1.12E-27	0.973	0.991	v6.0	HCP
LH_caudalanteriorcingulate_surfavg	ICC2	0.9004	23.85	34	34	1.20E-15	0.793	0.948	v6.0 vs. v7.1	HCP
LH_caudalanteriorcingulate_surfavg	ICC3	0.9876	159.75	34	34	3.32E-29	0.978	0.993	v7.1	HCP
LH_caudalanteriorcingulate_thickavg	ICC3	0.9057	20.21	34	34	1.59E-14	0.839	0.946	v5.3	HCP
LH_caudalanteriorcingulate_thickavg	ICC2	0.8382	11.61	34	34	6.50E-11	0.731	0.905	v5.3 vs. v6.0	HCP
LH_caudalanteriorcingulate_thickavg	ICC2	0.5351	6.70	34	34	1.23E-07	0.008	0.777	v5.3 vs. v7.1	HCP
LH_caudalanteriorcingulate_thickavg	ICC3	0.8644	13.74	34	34	5.51E-12	0.772	0.921	v6.0	HCP
LH_caudalanteriorcingulate_thickavg	ICC2	0.4678	11.02	34	34	1.39E-10	-0.056	0.763	v6.0 vs. v7.1	HCP
LH_caudalanteriorcingulate_thickavg	ICC3	0.9303	27.71	34	34	1.12E-16	0.880	0.960	v7.1	HCP
LH_caudalmiddlefrontal_surfavg	ICC3	0.9924	261.49	34	34	8.26E-33	0.987	0.996	v5.3	HCP
LH_caudalmiddlefrontal_surfavg	ICC2	0.9409	34.02	34	34	4.20E-18	0.897	0.966	v5.3 vs. v6.0	HCP
LH_caudalmiddlefrontal_surfavg	ICC2	0.9355	30.96	34	34	1.91E-17	0.888	0.963	v5.3 vs. v7.1	HCP
LH_caudalmiddlefrontal_surfavg	ICC3	0.9862	143.86	34	34	1.93E-28	0.976	0.992	v6.0	HCP

LH_caudalmiddlefrontal_surfavg	ICC2	0.9888	172.59	34	34	9.06E-30	0.980	0.994	v6.0 vs. v7.1	HCP
LH_caudalmiddlefrontal_surfavg	ICC3	0.9929	281.22	34	34	2.42E-33	0.987	0.996	v7.1	HCP
LH_caudalmiddlefrontal_thickavg	ICC3	0.8757	15.10	34	34	1.36E-12	0.790	0.928	v5.3	HCP
LH_caudalmiddlefrontal_thickavg	ICC2	0.9189	27.57	34	34	1.21E-16	0.845	0.956	v5.3 vs. v6.0	HCP
LH_caudalmiddlefrontal_thickavg	ICC2	0.8456	22.23	34	34	3.61E-15	0.450	0.936	v5.3 vs. v7.1	HCP
LH_caudalmiddlefrontal_thickavg	ICC3	0.8816	15.90	34	34	6.24E-13	0.799	0.931	v6.0	HCP
LH_caudalmiddlefrontal_thickavg	ICC2	0.7844	28.15	34	34	8.72E-17	0.047	0.925	v6.0 vs. v7.1	HCP
LH_caudalmiddlefrontal_thickavg	ICC3	0.9325	28.64	34	34	6.60E-17	0.883	0.961	v7.1	HCP
LH_Caudate	ICC3	0.9802	100.02	34	34	8.44E-26	0.965	0.989	v5.3	HCP
LH_Caudate	ICC2	0.8224	67.66	34	34	5.58E-23	0.008	0.946	v5.3 vs. v6.0	HCP
LH_Caudate	ICC2	0.8394	52.79	34	34	3.32E-21	0.045	0.949	v5.3 vs. v7.1	HCP
LH_Caudate	ICC3	0.9782	90.81	34	34	4.23E-25	0.962	0.988	v6.0	HCP
LH_Caudate	ICC2	0.9792	105.43	34	34	3.51E-26	0.961	0.989	v6.0 vs. v7.1	HCP
LH_Caudate	ICC3	0.9701	65.79	34	34	8.87E-23	0.948	0.983	v7.1	HCP
LH_cuneus_surfavg	ICC3	0.9189	23.65	34	34	1.36E-15	0.861	0.953	v5.3	HCP
LH_cuneus_surfavg	ICC2	0.6370	5.74	34	34	8.56E-07	0.359	0.795	v5.3 vs. v6.0	HCP
LH_cuneus_surfavg	ICC2	0.6582	7.48	34	34	2.92E-08	0.262	0.828	v5.3 vs. v7.1	HCP
LH_cuneus_surfavg	ICC3	0.9822	111.13	34	34	1.45E-26	0.969	0.990	v6.0	HCP
LH_cuneus_surfavg	ICC2	0.9763	101.99	34	34	6.10E-26	0.950	0.988	v6.0 vs. v7.1	HCP
LH_cuneus_surfavg	ICC3	0.9839	123.14	34	34	2.61E-27	0.972	0.991	v7.1	HCP
LH_cuneus_thickavg	ICC3	0.9359	30.19	34	34	2.86E-17	0.889	0.963	v5.3	HCP
LH_cuneus_thickavg	ICC2	0.8312	17.72	34	34	1.20E-13	0.509	0.925	v5.3 vs. v6.0	HCP
LH_cuneus_thickavg	ICC2	0.8628	13.30	34	34	8.93E-12	0.769	0.920	v5.3 vs. v7.1	HCP
LH_cuneus_thickavg	ICC3	0.9461	36.08	34	34	1.63E-18	0.906	0.969	v6.0	HCP
LH_cuneus_thickavg	ICC2	0.9010	43.73	34	34	7.21E-20	0.465	0.964	v6.0 vs. v7.1	HCP
LH_cuneus_thickavg	ICC3	0.9516	40.35	34	34	2.67E-19	0.916	0.972	v7.1	HCP
LH_entorhinal_surfavg	ICC3	0.8816	15.89	34	34	6.25E-13	0.799	0.931	v5.3	HCP
LH_entorhinal_surfavg	ICC2	0.7476	7.93	34	34	1.35E-08	0.570	0.854	v5.3 vs. v6.0	HCP
LH_entorhinal_surfavg	ICC2	0.6946	5.44	34	34	1.67E-06	0.515	0.816	v5.3 vs. v7.1	HCP
LH_entorhinal_surfavg	ICC3	0.8818	15.92	34	34	6.12E-13	0.800	0.932	v6.0	HCP
LH_entorhinal_surfavg	ICC2	0.8380	14.54	34	34	2.38E-12	0.667	0.915	v6.0 vs. v7.1	HCP
LH_entorhinal_surfavg	ICC3	0.8442	11.84	34	34	4.94E-11	0.740	0.909	v7.1	HCP
LH_entorhinal_thickavg	ICC3	0.7745	7.87	34	34	1.49E-08	0.632	0.866	v5.3	HCP
LH_entorhinal_thickavg	ICC2	0.8830	15.67	34	34	7.77E-13	0.801	0.932	v5.3 vs. v6.0	HCP
LH_entorhinal_thickavg	ICC2	0.6578	9.82	34	34	7.07E-10	0.101	0.849	v5.3 vs. v7.1	HCP
LH_entorhinal_thickavg	ICC3	0.8329	10.97	34	34	1.48E-10	0.722	0.902	v6.0	HCP
LH_entorhinal_thickavg	ICC2	0.6749	15.76	34	34	7.14E-13	-0.006	0.874	v6.0 vs. v7.1	HCP
LH_entorhinal_thickavg	ICC3	0.8222	10.25	34	34	3.90E-10	0.705	0.896	v7.1	HCP
LH_frontalpole_surfavg	ICC3	0.5983	3.98	34	34	5.82E-05	0.384	0.752	v5.3	HCP
LH_frontalpole_surfavg	ICC2	0.1703	3.00	34	34	9.48E-04	-0.062	0.429	v5.3 vs. v6.0	HCP
LH_frontalpole_surfavg	ICC2	0.1152	2.89	34	34	1.31E-03	-0.047	0.334	v5.3 vs. v7.1	HCP
LH_frontalpole_surfavg	ICC3	0.8467	12.05	34	34	3.82E-11	0.744	0.911	v6.0	HCP
LH_frontalpole_surfavg	ICC2	0.7689	14.83	34	34	1.77E-12	0.269	0.903	v6.0 vs. v7.1	HCP
LH_frontalpole_surfavg	ICC3	0.9203	24.10	34	34	1.01E-15	0.863	0.954	v7.1	HCP

LH_frontalpole_thickavg	ICC3	0.7974	8.87	34	34	2.92E-09	0.667	0.880	v5.3	HCP
LH_frontalpole_thickavg	ICC2	0.7199	7.60	34	34	2.37E-08	0.492	0.844	v5.3 vs. v6.0	HCP
LH_frontalpole_thickavg	ICC2	0.6665	11.11	34	34	1.23E-10	0.070	0.858	v5.3 vs. v7.1	HCP
LH_frontalpole_thickavg	ICC3	0.8258	10.48	34	34	2.83E-10	0.711	0.898	v6.0	HCP
LH_frontalpole_thickavg	ICC2	0.8365	17.62	34	34	1.31E-13	0.547	0.925	v6.0 vs. v7.1	HCP
LH_frontalpole_thickavg	ICC3	0.8731	14.76	34	34	1.91E-12	0.786	0.926	v7.1	HCP
LH_fusiform_surfavg	ICC3	0.9639	54.41	34	34	2.03E-21	0.937	0.979	v5.3	HCP
LH_fusiform_surfavg	ICC2	0.8378	16.52	34	34	3.48E-13	0.597	0.922	v5.3 vs. v6.0	HCP
LH_fusiform_surfavg	ICC2	0.8712	17.53	34	34	1.41E-13	0.750	0.930	v5.3 vs. v7.1	HCP
LH_fusiform_surfavg	ICC3	0.9829	116.05	34	34	7.05E-27	0.970	0.990	v6.0	HCP
LH_fusiform_surfavg	ICC2	0.9807	153.62	34	34	6.41E-29	0.939	0.991	v6.0 vs. v7.1	HCP
LH_fusiform_surfavg	ICC3	0.9882	168.43	34	34	1.37E-29	0.979	0.993	v7.1	HCP
LH_fusiform_thickavg	ICC3	0.8694	14.32	34	34	3.00E-12	0.780	0.924	v5.3	HCP
LH_fusiform_thickavg	ICC2	0.8987	18.55	34	34	5.93E-14	0.828	0.941	v5.3 vs. v6.0	HCP
LH_fusiform_thickavg	ICC2	0.7071	13.44	34	34	7.69E-12	0.093	0.880	v5.3 vs. v7.1	HCP
LH_fusiform_thickavg	ICC3	0.8759	15.11	34	34	1.34E-12	0.790	0.928	v6.0	HCP
LH_fusiform_thickavg	ICC2	0.7054	32.11	34	34	1.06E-17	-0.026	0.899	v6.0 vs. v7.1	HCP
LH_fusiform_thickavg	ICC3	0.9081	20.76	34	34	1.05E-14	0.843	0.947	v7.1	HCP
LH_Hippocampus	ICC3	0.9648	55.82	34	34	1.33E-21	0.938	0.980	v5.3	HCP
LH_Hippocampus	ICC2	0.9427	52.09	34	34	4.14E-21	0.821	0.975	v5.3 vs. v6.0	HCP
LH_Hippocampus	ICC2	0.9573	48.28	34	34	1.44E-20	0.925	0.976	v5.3 vs. v7.1	HCP
LH_Hippocampus	ICC3	0.9597	48.57	34	34	1.30E-20	0.930	0.977	v6.0	HCP
LH_Hippocampus	ICC2	0.9578	56.57	34	34	1.07E-21	0.912	0.978	v6.0 vs. v7.1	HCP
LH_Hippocampus	ICC3	0.9694	64.45	34	34	1.25E-22	0.946	0.983	v7.1	HCP
LH_inferiorparietal_surfavg	ICC3	0.9907	214.81	34	34	2.28E-31	0.984	0.995	v5.3	HCP
LH_inferiorparietal_surfavg	ICC2	0.8476	17.46	34	34	1.51E-13	0.624	0.927	v5.3 vs. v6.0	HCP
LH_inferiorparietal_surfavg	ICC2	0.8713	19.77	34	34	2.23E-14	0.703	0.936	v5.3 vs. v7.1	HCP
LH_inferiorparietal_surfavg	ICC3	0.9865	147.40	34	34	1.28E-28	0.976	0.992	v6.0	HCP
LH_inferiorparietal_surfavg	ICC2	0.9817	111.25	34	34	1.43E-26	0.968	0.990	v6.0 vs. v7.1	HCP
LH_inferiorparietal_surfavg	ICC3	0.9955	443.65	34	34	1.09E-36	0.992	0.997	v7.1	HCP
LH_inferiorparietal_thickavg	ICC3	0.9467	36.55	34	34	1.32E-18	0.908	0.970	v5.3	HCP
LH_inferiorparietal_thickavg	ICC2	0.9363	33.42	34	34	5.60E-18	0.885	0.964	v5.3 vs. v6.0	HCP
LH_inferiorparietal_thickavg	ICC2	0.7952	23.33	34	34	1.69E-15	0.136	0.925	v5.3 vs. v7.1	HCP
LH_inferiorparietal_thickavg	ICC3	0.9157	22.72	34	34	2.55E-15	0.855	0.952	v6.0	HCP
LH_inferiorparietal_thickavg	ICC2	0.7541	52.73	34	34	3.39E-21	-0.015	0.921	v6.0 vs. v7.1	HCP
LH_inferiorparietal_thickavg	ICC3	0.9539	42.35	34	34	1.22E-19	0.920	0.974	v7.1	HCP
LH_inferiortemporal_surfavg	ICC3	0.9877	161.42	34	34	2.79E-29	0.978	0.993	v5.3	HCP
LH_inferiortemporal_surfavg	ICC2	0.9172	29.74	34	34	3.63E-17	0.818	0.958	v5.3 vs. v6.0	HCP
LH_inferiortemporal_surfavg	ICC2	0.8923	32.64	34	34	8.16E-18	0.574	0.957	v5.3 vs. v7.1	HCP
LH_inferiortemporal_surfavg	ICC3	0.9915	234.69	34	34	5.12E-32	0.985	0.995	v6.0	HCP
LH_inferiortemporal_surfavg	ICC2	0.9859	303.68	34	34	6.61E-34	0.900	0.995	v6.0 vs. v7.1	HCP
LH_inferiortemporal_surfavg	ICC3	0.9935	304.96	34	34	6.15E-34	0.988	0.996	v7.1	HCP
LH_inferiortemporal_thickavg	ICC3	0.8718	14.60	34	34	2.23E-12	0.784	0.926	v5.3	HCP
LH_inferiortemporal_thickavg	ICC2	0.9281	33.04	34	34	6.71E-18	0.851	0.962	v5.3 vs. v6.0	HCP

LH_inferiortemporal_thickavg	ICC2	0.6330	20.90	34	34	9.37E-15	-0.039	0.863	v5.3 vs. v7.1	HCP
LH_inferiortemporal_thickavg	ICC3	0.8879	16.84	34	34	2.61E-13	0.810	0.935	v6.0	HCP
LH_inferiortemporal_thickavg	ICC2	0.7153	34.96	34	34	2.71E-18	-0.024	0.904	v6.0 vs. v7.1	HCP
LH_inferiortemporal_thickavg	ICC3	0.9277	26.68	34	34	2.04E-16	0.875	0.959	v7.1	HCP
LH_insula_surfavg	ICC3	0.9505	39.40	34	34	3.93E-19	0.914	0.972	v5.3	HCP
LH_insula_surfavg	ICC2	0.7425	16.60	34	34	3.24E-13	0.105	0.899	v5.3 vs. v6.0	HCP
LH_insula_surfavg	ICC2	0.7688	20.08	34	34	1.74E-14	0.108	0.913	v5.3 vs. v7.1	HCP
LH_insula_surfavg	ICC3	0.8680	14.15	34	34	3.56E-12	0.777	0.923	v6.0	HCP
LH_insula_surfavg	ICC2	0.8804	15.40	34	34	1.01E-12	0.798	0.931	v6.0 vs. v7.1	HCP
LH_insula_surfavg	ICC3	0.8562	12.91	34	34	1.38E-11	0.759	0.916	v7.1	HCP
LH_insula_thickavg	ICC3	0.8490	12.24	34	34	3.02E-11	0.747	0.912	v5.3	HCP
LH_insula_thickavg	ICC2	0.8242	10.52	34	34	2.69E-10	0.710	0.897	v5.3 vs. v6.0	HCP
LH_insula_thickavg	ICC2	0.6739	10.59	34	34	2.44E-10	0.109	0.858	v5.3 vs. v7.1	HCP
LH_insula_thickavg	ICC3	0.7360	6.58	34	34	1.56E-07	0.575	0.842	v6.0	HCP
LH_insula_thickavg	ICC2	0.5911	8.39	34	34	6.27E-09	0.024	0.815	v6.0 vs. v7.1	HCP
LH_insula_thickavg	ICC3	0.8077	9.40	34	34	1.31E-09	0.683	0.887	v7.1	HCP
LH_isthmuscingulate_surfavg	ICC3	0.8458	11.97	34	34	4.20E-11	0.742	0.910	v5.3	HCP
LH_isthmuscingulate_surfavg	ICC2	0.8532	12.45	34	34	2.35E-11	0.754	0.914	v5.3 vs. v6.0	HCP
LH_isthmuscingulate_surfavg	ICC2	0.7952	8.57	34	34	4.72E-09	0.663	0.879	v5.3 vs. v7.1	HCP
LH_isthmuscingulate_surfavg	ICC3	0.9744	76.99	34	34	6.58E-24	0.955	0.985	v6.0	HCP
LH_isthmuscingulate_surfavg	ICC2	0.9485	37.09	34	34	1.04E-18	0.911	0.971	v6.0 vs. v7.1	HCP
LH_isthmuscingulate_surfavg	ICC3	0.9779	89.45	34	34	5.43E-25	0.961	0.987	v7.1	HCP
LH_isthmuscingulate_thickavg	ICC3	0.7978	8.89	34	34	2.84E-09	0.668	0.881	v5.3	HCP
LH_isthmuscingulate_thickavg	ICC2	0.5328	6.60	34	34	1.49E-07	0.009	0.775	v5.3 vs. v6.0	HCP
LH_isthmuscingulate_thickavg	ICC2	0.2615	6.17	34	34	3.53E-07	-0.052	0.573	v5.3 vs. v7.1	HCP
LH_isthmuscingulate_thickavg	ICC3	0.8591	13.19	34	34	1.01E-11	0.763	0.918	v6.0	HCP
LH_isthmuscingulate_thickavg	ICC2	0.4735	6.40	34	34	2.21E-07	-0.043	0.744	v6.0 vs. v7.1	HCP
LH_isthmuscingulate_thickavg	ICC3	0.8090	9.47	34	34	1.18E-09	0.685	0.888	v7.1	HCP
LH_lateraloccipital_surfavg	ICC3	0.9818	108.76	34	34	2.09E-26	0.968	0.990	v5.3	HCP
LH_lateraloccipital_surfavg	ICC2	0.7443	13.98	34	34	4.26E-12	0.197	0.893	v5.3 vs. v6.0	HCP
LH_lateraloccipital_surfavg	ICC2	0.7116	13.20	34	34	9.98E-12	0.113	0.881	v5.3 vs. v7.1	HCP
LH_lateraloccipital_surfavg	ICC3	0.9883	169.31	34	34	1.25E-29	0.979	0.993	v6.0	HCP
LH_lateraloccipital_surfavg	ICC2	0.9860	155.40	34	34	5.29E-29	0.974	0.992	v6.0 vs. v7.1	HCP
LH_lateraloccipital_surfavg	ICC3	0.9943	349.67	34	34	6.09E-35	0.990	0.997	v7.1	HCP
LH_lateraloccipital_thickavg	ICC3	0.9589	47.72	34	34	1.74E-20	0.928	0.977	v5.3	HCP
LH_lateraloccipital_thickavg	ICC2	0.9399	31.67	34	34	1.33E-17	0.896	0.966	v5.3 vs. v6.0	HCP
LH_lateraloccipital_thickavg	ICC2	0.8509	25.37	34	34	4.53E-16	0.397	0.941	v5.3 vs. v7.1	HCP
LH_lateraloccipital_thickavg	ICC3	0.9460	36.01	34	34	1.68E-18	0.906	0.969	v6.0	HCP
LH_lateraloccipital_thickavg	ICC2	0.8663	67.13	34	34	6.36E-23	0.068	0.959	v6.0 vs. v7.1	HCP
LH_lateraloccipital_thickavg	ICC3	0.9617	51.16	34	34	5.56E-21	0.933	0.978	v7.1	HCP
LH_lateralorbitofrontal_surfavg	ICC3	0.9800	99.01	34	34	1.00E-25	0.965	0.989	v5.3	HCP
LH_lateralorbitofrontal_surfavg	ICC2	0.9646	62.02	34	34	2.35E-22	0.934	0.980	v5.3 vs. v6.0	HCP
LH_lateralorbitofrontal_surfavg	ICC2	0.9184	50.53	34	34	6.83E-21	0.565	0.970	v5.3 vs. v7.1	HCP
LH_lateralorbitofrontal_surfavg	ICC3	0.9786	92.40	34	34	3.17E-25	0.962	0.988	v6.0	HCP

LH_lateralorbitofrontal_surfavg	ICC2	0.9603	112.10	34	34	1.26E-26	0.725	0.986	v6.0 vs. v7.1	HCP
LH_lateralorbitofrontal_surfavg	ICC3	0.9829	115.87	34	34	7.24E-27	0.970	0.990	v7.1	HCP
LH_lateralorbitofrontal_thickavg	ICC3	0.8666	13.99	34	34	4.22E-12	0.775	0.922	v5.3	HCP
LH_lateralorbitofrontal_thickavg	ICC2	0.8748	16.84	34	34	2.61E-13	0.776	0.929	v5.3 vs. v6.0	HCP
LH_lateralorbitofrontal_thickavg	ICC2	0.7684	13.52	34	34	7.00E-12	0.330	0.898	v5.3 vs. v7.1	HCP
LH_lateralorbitofrontal_thickavg	ICC3	0.8789	15.51	34	34	9.06E-13	0.795	0.930	v6.0	HCP
LH_lateralorbitofrontal_thickavg	ICC2	0.7315	30.51	34	34	2.41E-17	-0.017	0.908	v6.0 vs. v7.1	HCP
LH_lateralorbitofrontal_thickavg	ICC3	0.8763	15.16	34	34	1.27E-12	0.791	0.928	v7.1	HCP
LH_LateralVentricle	ICC3	0.9959	489.13	34	34	2.08E-37	0.993	0.998	v5.3	HCP
LH_LateralVentricle	ICC2	0.9785	540.29	34	34	3.86E-38	0.441	0.994	v5.3 vs. v6.0	HCP
LH_LateralVentricle	ICC2	0.9755	446.94	34	34	9.58E-37	0.426	0.993	v5.3 vs. v7.1	HCP
LH_LateralVentricle	ICC3	0.9963	538.86	34	34	4.04E-38	0.993	0.998	v6.0	HCP
LH_LateralVentricle	ICC2	0.9993	3316.57	34	34	1.63E-51	0.999	1.000	v6.0 vs. v7.1	HCP
LH_LateralVentricle	ICC3	0.9967	602.66	34	34	6.06E-39	0.994	0.998	v7.1	HCP
LH_lingual_surfavg	ICC3	0.9794	95.95	34	34	1.69E-25	0.964	0.988	v5.3	HCP
LH_lingual_surfavg	ICC2	0.8818	15.53	34	34	8.89E-13	0.800	0.932	v5.3 vs. v6.0	HCP
LH_lingual_surfavg	ICC2	0.8647	14.91	34	34	1.64E-12	0.767	0.922	v5.3 vs. v7.1	HCP
LH_lingual_surfavg	ICC3	0.9872	155.41	34	34	5.28E-29	0.977	0.993	v6.0	HCP
LH_lingual_surfavg	ICC2	0.9783	166.01	34	34	1.74E-29	0.896	0.991	v6.0 vs. v7.1	HCP
LH_lingual_surfavg	ICC3	0.9910	222.22	34	34	1.29E-31	0.984	0.995	v7.1	HCP
LH_lingual_thickavg	ICC3	0.9271	26.43	34	34	2.37E-16	0.874	0.958	v5.3	HCP
LH_lingual_thickavg	ICC2	0.8732	24.38	34	34	8.46E-16	0.598	0.945	v5.3 vs. v6.0	HCP
LH_lingual_thickavg	ICC2	0.8566	18.73	34	34	5.11E-14	0.640	0.931	v5.3 vs. v7.1	HCP
LH_lingual_thickavg	ICC3	0.8748	14.97	34	34	1.54E-12	0.788	0.927	v6.0	HCP
LH_lingual_thickavg	ICC2	0.7832	45.32	34	34	4.03E-20	-0.004	0.930	v6.0 vs. v7.1	HCP
LH_lingual_thickavg	ICC3	0.9093	21.06	34	34	8.34E-15	0.845	0.948	v7.1	HCP
LH_medialorbitofrontal_surfavg	ICC3	0.9322	28.52	34	34	7.07E-17	0.883	0.961	v5.3	HCP
LH_medialorbitofrontal_surfavg	ICC2	0.8467	12.07	34	34	3.71E-11	0.745	0.910	v5.3 vs. v6.0	HCP
LH_medialorbitofrontal_surfavg	ICC2	0.7615	10.25	34	34	3.88E-10	0.488	0.878	v5.3 vs. v7.1	HCP
LH_medialorbitofrontal_surfavg	ICC3	0.8434	11.77	34	34	5.36E-11	0.738	0.908	v6.0	HCP
LH_medialorbitofrontal_surfavg	ICC2	0.7930	10.61	34	34	2.39E-10	0.611	0.886	v6.0 vs. v7.1	HCP
LH_medialorbitofrontal_surfavg	ICC3	0.8648	13.79	34	34	5.25E-12	0.772	0.921	v7.1	HCP
LH_medialorbitofrontal_thickavg	ICC3	0.7372	6.61	34	34	1.46E-07	0.577	0.843	v5.3	HCP
LH_medialorbitofrontal_thickavg	ICC2	0.8578	13.52	34	34	7.07E-12	0.762	0.917	v5.3 vs. v6.0	HCP
LH_medialorbitofrontal_thickavg	ICC2	0.7289	12.49	34	34	2.27E-11	0.203	0.884	v5.3 vs. v7.1	HCP
LH_medialorbitofrontal_thickavg	ICC3	0.8655	13.87	34	34	4.83E-12	0.773	0.922	v6.0	HCP
LH_medialorbitofrontal_thickavg	ICC2	0.6694	15.99	34	34	5.73E-13	-0.013	0.873	v6.0 vs. v7.1	HCP
LH_medialorbitofrontal_thickavg	ICC3	0.8234	10.33	34	34	3.50E-10	0.707	0.896	v7.1	HCP
LH_middletemporal_surfavg	ICC3	0.9888	177.20	34	34	5.82E-30	0.980	0.994	v5.3	HCP
LH_middletemporal_surfavg	ICC2	0.9150	40.86	34	34	2.18E-19	0.658	0.966	v5.3 vs. v6.0	HCP
LH_middletemporal_surfavg	ICC2	0.8647	36.22	34	34	1.53E-18	0.269	0.952	v5.3 vs. v7.1	HCP
LH_middletemporal_surfavg	ICC3	0.9923	257.81	34	34	1.05E-32	0.986	0.996	v6.0	HCP
LH_middletemporal_surfavg	ICC2	0.9812	221.99	34	34	1.31E-31	0.876	0.993	v6.0 vs. v7.1	HCP
LH_middletemporal_surfavg	ICC3	0.9952	419.43	34	34	2.81E-36	0.992	0.997	v7.1	HCP

LH_middletemporal_thickavg	ICC3	0.9181	23.41	34	34	1.60E-15	0.859	0.953	v5.3	HCP
LH_middletemporal_thickavg	ICC2	0.9031	39.56	34	34	3.68E-19	0.554	0.963	v5.3 vs. v6.0	HCP
LH_middletemporal_thickavg	ICC2	0.6058	29.55	34	34	4.02E-17	-0.031	0.855	v5.3 vs. v7.1	HCP
LH_middletemporal_thickavg	ICC3	0.9262	26.11	34	34	2.86E-16	0.873	0.958	v6.0	HCP
LH_middletemporal_thickavg	ICC2	0.7551	65.33	34	34	9.96E-23	-0.012	0.923	v6.0 vs. v7.1	HCP
LH_middletemporal_thickavg	ICC3	0.9459	35.95	34	34	1.73E-18	0.906	0.969	v7.1	HCP
LH_Pallidum	ICC3	0.7548	7.16	34	34	5.22E-08	0.603	0.854	v5.3	HCP
LH_Pallidum	ICC2	0.1522	3.69	34	34	1.29E-04	-0.049	0.408	v5.3 vs. v6.0	HCP
LH_Pallidum	ICC2	0.1607	4.06	34	34	4.73E-05	-0.047	0.424	v5.3 vs. v7.1	HCP
LH_Pallidum	ICC3	0.8271	10.57	34	34	2.53E-10	0.713	0.899	v6.0	HCP
LH_Pallidum	ICC2	0.8954	17.71	34	34	1.21E-13	0.822	0.940	v6.0 vs. v7.1	HCP
LH_Pallidum	ICC3	0.9096	21.13	34	34	7.92E-15	0.845	0.948	v7.1	HCP
LH_paracentral_surfavg	ICC3	0.9700	65.64	34	34	9.21E-23	0.947	0.983	v5.3	HCP
LH_paracentral_surfavg	ICC2	0.8239	10.84	34	34	1.76E-10	0.707	0.897	v5.3 vs. v6.0	HCP
LH_paracentral_surfavg	ICC2	0.8171	9.91	34	34	6.26E-10	0.699	0.892	v5.3 vs. v7.1	HCP
LH_paracentral_surfavg	ICC3	0.9817	108.24	34	34	2.26E-26	0.968	0.990	v6.0	HCP
LH_paracentral_surfavg	ICC2	0.9809	115.46	34	34	7.68E-27	0.964	0.989	v6.0 vs. v7.1	HCP
LH_paracentral_surfavg	ICC3	0.9666	58.89	34	34	5.51E-22	0.942	0.981	v7.1	HCP
LH_paracentral_thickavg	ICC3	0.9176	23.27	34	34	1.75E-15	0.858	0.953	v5.3	HCP
LH_paracentral_thickavg	ICC2	0.8706	20.65	34	34	1.14E-14	0.676	0.938	v5.3 vs. v6.0	HCP
LH_paracentral_thickavg	ICC2	0.9294	28.92	34	34	5.67E-17	0.877	0.960	v5.3 vs. v7.1	HCP
LH_paracentral_thickavg	ICC3	0.9145	22.39	34	34	3.22E-15	0.853	0.951	v6.0	HCP
LH_paracentral_thickavg	ICC2	0.9499	55.47	34	34	1.47E-21	0.863	0.977	v6.0 vs. v7.1	HCP
LH_paracentral_thickavg	ICC3	0.9495	38.61	34	34	5.46E-19	0.912	0.971	v7.1	HCP
LH_parahippocampal_surfavg	ICC3	0.8415	11.62	34	34	6.47E-11	0.735	0.907	v5.3	HCP
LH_parahippocampal_surfavg	ICC2	0.7660	7.97	34	34	1.25E-08	0.619	0.861	v5.3 vs. v6.0	HCP
LH_parahippocampal_surfavg	ICC2	0.7333	7.85	34	34	1.53E-08	0.525	0.850	v5.3 vs. v7.1	HCP
LH_parahippocampal_surfavg	ICC3	0.9622	51.88	34	34	4.43E-21	0.934	0.978	v6.0	HCP
LH_parahippocampal_surfavg	ICC2	0.9562	59.00	34	34	5.35E-22	0.896	0.978	v6.0 vs. v7.1	HCP
LH_parahippocampal_surfavg	ICC3	0.9309	27.93	34	34	9.87E-17	0.881	0.960	v7.1	HCP
LH_parahippocampal_thickavg	ICC3	0.9334	29.04	34	34	5.29E-17	0.885	0.962	v5.3	HCP
LH_parahippocampal_thickavg	ICC2	0.9628	52.61	34	34	3.52E-21	0.935	0.979	v5.3 vs. v6.0	HCP
LH_parahippocampal_thickavg	ICC2	0.8518	33.49	34	34	5.42E-18	0.230	0.947	v5.3 vs. v7.1	HCP
LH_parahippocampal_thickavg	ICC3	0.9620	51.70	34	34	4.68E-21	0.934	0.978	v6.0	HCP
LH_parahippocampal_thickavg	ICC2	0.8577	74.28	34	34	1.19E-23	0.039	0.957	v6.0 vs. v7.1	HCP
LH_parahippocampal_thickavg	ICC3	0.9594	48.29	34	34	1.43E-20	0.929	0.977	v7.1	HCP
LH_parsopercularis_surfavg	ICC3	0.9740	75.96	34	34	8.21E-24	0.954	0.985	v5.3	HCP
LH_parsopercularis_surfavg	ICC2	0.8692	23.50	34	34	1.51E-15	0.591	0.943	v5.3 vs. v6.0	HCP
LH_parsopercularis_surfavg	ICC2	0.6975	5.73	34	34	8.76E-07	0.523	0.817	v5.3 vs. v7.1	HCP
LH_parsopercularis_surfavg	ICC3	0.9913	228.08	34	34	8.29E-32	0.985	0.995	v6.0	HCP
LH_parsopercularis_surfavg	ICC2	0.7094	5.89	34	34	6.29E-07	0.539	0.824	v6.0 vs. v7.1	HCP
LH_parsopercularis_surfavg	ICC3	0.9963	541.13	34	34	3.76E-38	0.993	0.998	v7.1	HCP
LH_parsopercularis_thickavg	ICC3	0.8492	12.27	34	34	2.94E-11	0.748	0.912	v5.3	HCP
LH_parsopercularis_thickavg	ICC2	0.8501	19.94	34	34	1.95E-14	0.557	0.933	v5.3 vs. v6.0	HCP

LH_parsopercularis_thickavg	ICC2	0.8813	16.39	34	34	3.93E-13	0.799	0.931	v5.3 vs. v7.1	HCP
LH_parsopercularis_thickavg	ICC3	0.8707	14.47	34	34	2.56E-12	0.782	0.925	v6.0	HCP
LH_parsopercularis_thickavg	ICC2	0.8295	27.57	34	34	1.21E-16	0.208	0.938	v6.0 vs. v7.1	HCP
LH_parsopercularis_thickavg	ICC3	0.8889	17.01	34	34	2.24E-13	0.811	0.936	v7.1	HCP
LH_parsorbitalis_surfavg	ICC3	0.9423	33.64	34	34	5.03E-18	0.900	0.967	v5.3	HCP
LH_parsorbitalis_surfavg	ICC2	0.6648	16.03	34	34	5.51E-13	-0.017	0.871	v5.3 vs. v6.0	HCP
LH_parsorbitalis_surfavg	ICC2	0.5906	16.01	34	34	5.60E-13	-0.046	0.839	v5.3 vs. v7.1	HCP
LH_parsorbitalis_surfavg	ICC3	0.9719	70.14	34	34	3.08E-23	0.951	0.984	v6.0	HCP
LH_parsorbitalis_surfavg	ICC2	0.9464	60.82	34	34	3.24E-22	0.800	0.978	v6.0 vs. v7.1	HCP
LH_parsorbitalis_surfavg	ICC3	0.9655	56.96	34	34	9.55E-22	0.940	0.980	v7.1	HCP
LH_parsorbitalis_thickavg	ICC3	0.9067	20.44	34	34	1.33E-14	0.840	0.946	v5.3	HCP
LH_parsorbitalis_thickavg	ICC2	0.9344	28.89	34	34	5.74E-17	0.887	0.962	v5.3 vs. v6.0	HCP
LH_parsorbitalis_thickavg	ICC2	0.7674	15.27	34	34	1.15E-12	0.244	0.903	v5.3 vs. v7.1	HCP
LH_parsorbitalis_thickavg	ICC3	0.9188	23.65	34	34	1.37E-15	0.861	0.953	v6.0	HCP
LH_parsorbitalis_thickavg	ICC2	0.8182	36.75	34	34	1.21E-18	0.063	0.939	v6.0 vs. v7.1	HCP
LH_parsorbitalis_thickavg	ICC3	0.8994	18.88	34	34	4.54E-14	0.828	0.942	v7.1	HCP
LH_parstriangularis_surfavg	ICC3	0.9528	41.36	34	34	1.79E-19	0.918	0.973	v5.3	HCP
LH_parstriangularis_surfavg	ICC2	0.7617	8.62	34	34	4.34E-09	0.584	0.864	v5.3 vs. v6.0	HCP
LH_parstriangularis_surfavg	ICC2	0.6631	6.07	34	34	4.36E-07	0.415	0.807	v5.3 vs. v7.1	HCP
LH_parstriangularis_surfavg	ICC3	0.9695	64.65	34	34	1.18E-22	0.947	0.983	v6.0	HCP
LH_parstriangularis_surfavg	ICC2	0.9492	43.82	34	34	6.98E-20	0.904	0.972	v6.0 vs. v7.1	HCP
LH_parstriangularis_surfavg	ICC3	0.9875	159.08	34	34	3.57E-29	0.978	0.993	v7.1	HCP
LH_parstriangularis_thickavg	ICC3	0.8185	10.02	34	34	5.37E-10	0.699	0.893	v5.3	HCP
LH_parstriangularis_thickavg	ICC2	0.8559	14.12	34	34	3.68E-12	0.750	0.917	v5.3 vs. v6.0	HCP
LH_parstriangularis_thickavg	ICC2	0.7192	8.86	34	34	2.98E-09	0.392	0.858	v5.3 vs. v7.1	HCP
LH_parstriangularis_thickavg	ICC3	0.8514	12.46	34	34	2.33E-11	0.751	0.913	v6.0	HCP
LH_parstriangularis_thickavg	ICC2	0.7147	16.24	34	34	4.50E-13	0.045	0.890	v6.0 vs. v7.1	HCP
LH_parstriangularis_thickavg	ICC3	0.8912	17.38	34	34	1.61E-13	0.815	0.937	v7.1	HCP
LH_pericalcarine_surfavg	ICC3	0.9249	25.63	34	34	3.84E-16	0.871	0.957	v5.3	HCP
LH_pericalcarine_surfavg	ICC2	0.8145	10.49	34	34	2.79E-10	0.689	0.892	v5.3 vs. v6.0	HCP
LH_pericalcarine_surfavg	ICC2	0.8397	13.41	34	34	7.91E-12	0.707	0.911	v5.3 vs. v7.1	HCP
LH_pericalcarine_surfavg	ICC3	0.9848	130.85	34	34	9.45E-28	0.973	0.991	v6.0	HCP
LH_pericalcarine_surfavg	ICC2	0.9778	91.97	34	34	3.42E-25	0.961	0.987	v6.0 vs. v7.1	HCP
LH_pericalcarine_surfavg	ICC3	0.9869	151.21	34	34	8.36E-29	0.977	0.993	v7.1	HCP
LH_pericalcarine_thickavg	ICC3	0.9046	19.96	34	34	1.92E-14	0.837	0.945	v5.3	HCP
LH_pericalcarine_thickavg	ICC2	0.8320	13.86	34	34	4.86E-12	0.660	0.911	v5.3 vs. v6.0	HCP
LH_pericalcarine_thickavg	ICC2	0.8401	11.26	34	34	1.01E-10	0.733	0.907	v5.3 vs. v7.1	HCP
LH_pericalcarine_thickavg	ICC3	0.8650	13.82	34	34	5.08E-12	0.773	0.922	v6.0	HCP
LH_pericalcarine_thickavg	ICC2	0.9110	30.80	34	34	2.07E-17	0.765	0.958	v6.0 vs. v7.1	HCP
LH_pericalcarine_thickavg	ICC3	0.9088	20.92	34	34	9.27E-15	0.844	0.947	v7.1	HCP
LH_postcentral_surfavg	ICC3	0.9903	204.35	34	34	5.28E-31	0.983	0.994	v5.3	HCP
LH_postcentral_surfavg	ICC2	0.9737	98.04	34	34	1.18E-25	0.937	0.987	v5.3 vs. v6.0	HCP
LH_postcentral_surfavg	ICC2	0.8618	13.66	34	34	6.01E-12	0.769	0.919	v5.3 vs. v7.1	HCP
LH_postcentral_surfavg	ICC3	0.9939	325.53	34	34	2.04E-34	0.989	0.997	v6.0	HCP

LH_postcentral_surfavg	ICC2	0.9117	21.06	34	34	8.36E-15	0.849	0.949	v6.0 vs. v7.1	HCP
LH_postcentral_surfavg	ICC3	0.9778	88.99	34	34	5.92E-25	0.961	0.987	v7.1	HCP
LH_postcentral_thickavg	ICC3	0.9748	78.39	34	34	4.88E-24	0.956	0.986	v5.3	HCP
LH_postcentral_thickavg	ICC2	0.9237	51.66	34	34	4.74E-21	0.615	0.971	v5.3 vs. v6.0	HCP
LH_postcentral_thickavg	ICC2	0.8205	9.96	34	34	5.86E-10	0.703	0.895	v5.3 vs. v7.1	HCP
LH_postcentral_thickavg	ICC3	0.9506	39.45	34	34	3.85E-19	0.914	0.972	v6.0	HCP
LH_postcentral_thickavg	ICC2	0.8454	13.51	34	34	7.12E-12	0.726	0.913	v6.0 vs. v7.1	HCP
LH_postcentral_thickavg	ICC3	0.9560	44.44	34	34	5.55E-20	0.923	0.975	v7.1	HCP
LH_posteriorcingulate_surfavg	ICC3	0.9286	27.01	34	34	1.67E-16	0.877	0.959	v5.3	HCP
LH_posteriorcingulate_surfavg	ICC2	0.8323	10.69	34	34	2.15E-10	0.721	0.902	v5.3 vs. v6.0	HCP
LH_posteriorcingulate_surfavg	ICC2	0.8623	13.21	34	34	9.94E-12	0.768	0.920	v5.3 vs. v7.1	HCP
LH_posteriorcingulate_surfavg	ICC3	0.9877	160.95	34	34	2.93E-29	0.978	0.993	v6.0	HCP
LH_posteriorcingulate_surfavg	ICC2	0.9779	99.24	34	34	9.63E-26	0.959	0.988	v6.0 vs. v7.1	HCP
LH_posteriorcingulate_surfavg	ICC3	0.9807	102.51	34	34	5.60E-26	0.966	0.989	v7.1	HCP
LH_posteriorcingulate_thickavg	ICC3	0.8941	17.88	34	34	1.04E-13	0.820	0.939	v5.3	HCP
LH_posteriorcingulate_thickavg	ICC2	0.8619	17.48	34	34	1.47E-13	0.706	0.929	v5.3 vs. v6.0	HCP
LH_posteriorcingulate_thickavg	ICC2	0.4565	12.52	34	34	2.18E-11	-0.050	0.759	v5.3 vs. v7.1	HCP
LH_posteriorcingulate_thickavg	ICC3	0.9138	22.21	34	34	3.66E-15	0.852	0.950	v6.0	HCP
LH_posteriorcingulate_thickavg	ICC2	0.5980	26.27	34	34	2.61E-16	-0.035	0.850	v6.0 vs. v7.1	HCP
LH_posteriorcingulate_thickavg	ICC3	0.9394	32.02	34	34	1.11E-17	0.895	0.965	v7.1	HCP
LH_precentral_surfavg	ICC3	0.9892	184.87	34	34	2.85E-30	0.981	0.994	v5.3	HCP
LH_precentral_surfavg	ICC2	0.9760	110.90	34	34	1.51E-26	0.939	0.988	v5.3 vs. v6.0	HCP
LH_precentral_surfavg	ICC2	0.9771	98.51	34	34	1.09E-25	0.956	0.988	v5.3 vs. v7.1	HCP
LH_precentral_surfavg	ICC3	0.9866	148.04	34	34	1.19E-28	0.976	0.992	v6.0	HCP
LH_precentral_surfavg	ICC2	0.9943	379.92	34	34	1.50E-35	0.989	0.997	v6.0 vs. v7.1	HCP
LH_precentral_surfavg	ICC3	0.9906	210.76	34	34	3.14E-31	0.983	0.995	v7.1	HCP
LH_precentral_thickavg	ICC3	0.9276	26.61	34	34	2.12E-16	0.875	0.958	v5.3	HCP
LH_precentral_thickavg	ICC2	0.9213	61.36	34	34	2.81E-22	0.475	0.973	v5.3 vs. v6.0	HCP
LH_precentral_thickavg	ICC2	0.8623	14.52	34	34	2.42E-12	0.765	0.921	v5.3 vs. v7.1	HCP
LH_precentral_thickavg	ICC3	0.8816	15.89	34	34	6.31E-13	0.799	0.931	v6.0	HCP
LH_precentral_thickavg	ICC2	0.8042	17.60	34	34	1.33E-13	0.343	0.918	v6.0 vs. v7.1	HCP
LH_precentral_thickavg	ICC3	0.9243	25.42	34	34	4.37E-16	0.870	0.957	v7.1	HCP
LH_precuneus_surfavg	ICC3	0.9887	176.68	34	34	6.11E-30	0.980	0.994	v5.3	HCP
LH_precuneus_surfavg	ICC2	0.9119	22.00	34	34	4.23E-15	0.850	0.949	v5.3 vs. v6.0	HCP
LH_precuneus_surfavg	ICC2	0.7545	7.20	34	34	4.82E-08	0.605	0.853	v5.3 vs. v7.1	HCP
LH_precuneus_surfavg	ICC3	0.9955	441.05	34	34	1.20E-36	0.992	0.997	v6.0	HCP
LH_precuneus_surfavg	ICC2	0.8993	18.58	34	34	5.79E-14	0.829	0.942	v6.0 vs. v7.1	HCP
LH_precuneus_surfavg	ICC3	0.9895	189.74	34	34	1.84E-30	0.981	0.994	v7.1	HCP
LH_precuneus_thickavg	ICC3	0.9273	26.49	34	34	2.28E-16	0.875	0.958	v5.3	HCP
LH_precuneus_thickavg	ICC2	0.8427	16.57	34	34	3.34E-13	0.624	0.923	v5.3 vs. v6.0	HCP
LH_precuneus_thickavg	ICC2	0.8524	15.90	34	34	6.25E-13	0.698	0.922	v5.3 vs. v7.1	HCP
LH_precuneus_thickavg	ICC3	0.8926	17.62	34	34	1.31E-13	0.817	0.938	v6.0	HCP
LH_precuneus_thickavg	ICC2	0.7747	40.88	34	34	2.16E-19	-0.005	0.926	v6.0 vs. v7.1	HCP
LH_precuneus_thickavg	ICC3	0.9531	41.61	34	34	1.62E-19	0.918	0.973	v7.1	HCP

LH_Putamen	ICC3	0.8899	17.16	34	34	1.96E-13	0.813	0.936	v5.3	HCP
LH_Putamen	ICC2	0.6571	14.84	34	34	1.75E-12	-0.015	0.866	v5.3 vs. v6.0	HCP
LH_Putamen	ICC2	0.7007	13.58	34	34	6.60E-12	0.073	0.879	v5.3 vs. v7.1	HCP
LH_Putamen	ICC3	0.8870	16.70	34	34	2.97E-13	0.808	0.935	v6.0	HCP
LH_Putamen	ICC2	0.9250	29.08	34	34	5.19E-17	0.861	0.958	v6.0 vs. v7.1	HCP
LH_Putamen	ICC3	0.9712	68.42	34	34	4.64E-23	0.950	0.984	v7.1	HCP
LH_rostralanteriorcingulate_surfavg	ICC3	0.9791	94.59	34	34	2.14E-25	0.963	0.988	v5.3	HCP
LH_rostralanteriorcingulate_surfavg	ICC2	0.9252	34.71	34	34	3.04E-18	0.821	0.963	v5.3 vs. v6.0	HCP
LH_rostralanteriorcingulate_surfavg	ICC2	0.9155	23.63	34	34	1.39E-15	0.855	0.952	v5.3 vs. v7.1	HCP
LH_rostralanteriorcingulate_surfavg	ICC3	0.9637	54.16	34	34	2.18E-21	0.937	0.979	v6.0	HCP
LH_rostralanteriorcingulate_surfavg	ICC2	0.9519	42.89	34	34	9.92E-20	0.915	0.973	v6.0 vs. v7.1	HCP
LH_rostralanteriorcingulate_surfavg	ICC3	0.9800	99.12	34	34	9.82E-26	0.965	0.989	v7.1	HCP
LH_rostralanteriorcingulate_thickavg	ICC3	0.7654	7.53	34	34	2.70E-08	0.619	0.860	v5.3	HCP
LH_rostralanteriorcingulate_thickavg	ICC2	0.8046	10.05	34	34	5.17E-10	0.671	0.886	v5.3 vs. v6.0	HCP
LH_rostralanteriorcingulate_thickavg	ICC2	0.6934	11.78	34	34	5.26E-11	0.113	0.870	v5.3 vs. v7.1	HCP
LH_rostralanteriorcingulate_thickavg	ICC3	0.7507	7.02	34	34	6.71E-08	0.597	0.851	v6.0	HCP
LH_rostralanteriorcingulate_thickavg	ICC2	0.5928	11.20	34	34	1.10E-10	-0.032	0.830	v6.0 vs. v7.1	HCP
LH_rostralanteriorcingulate_thickavg	ICC3	0.8311	10.84	34	34	1.75E-10	0.719	0.901	v7.1	HCP
LH_rostralmiddlefrontal_surfavg	ICC3	0.9930	283.54	34	34	2.11E-33	0.988	0.996	v5.3	HCP
LH_rostralmiddlefrontal_surfavg	ICC2	0.9825	110.84	34	34	1.52E-26	0.969	0.990	v5.3 vs. v6.0	HCP
LH_rostralmiddlefrontal_surfavg	ICC2	0.9078	21.80	34	34	4.88E-15	0.841	0.947	v5.3 vs. v7.1	HCP
LH_rostralmiddlefrontal_surfavg	ICC3	0.9928	277.01	34	34	3.12E-33	0.987	0.996	v6.0	HCP
LH_rostralmiddlefrontal_surfavg	ICC2	0.9129	23.56	34	34	1.45E-15	0.848	0.950	v6.0 vs. v7.1	HCP
LH_rostralmiddlefrontal_surfavg	ICC3	0.9967	605.99	34	34	5.52E-39	0.994	0.998	v7.1	HCP
LH_rostralmiddlefrontal_thickavg	ICC3	0.8472	12.09	34	34	3.65E-11	0.744	0.911	v5.3	HCP
LH_rostralmiddlefrontal_thickavg	ICC2	0.9141	22.19	34	34	3.70E-15	0.853	0.950	v5.3 vs. v6.0	HCP
LH_rostralmiddlefrontal_thickavg	ICC2	0.7312	18.16	34	34	8.24E-14	0.047	0.898	v5.3 vs. v7.1	HCP
LH_rostralmiddlefrontal_thickavg	ICC3	0.8569	12.98	34	34	1.28E-11	0.760	0.917	v6.0	HCP
LH_rostralmiddlefrontal_thickavg	ICC2	0.7237	28.56	34	34	6.92E-17	-0.019	0.904	v6.0 vs. v7.1	HCP
LH_rostralmiddlefrontal_thickavg	ICC3	0.9242	25.39	34	34	4.47E-16	0.870	0.957	v7.1	HCP
LH_superiorfrontal_surfavg	ICC3	0.9943	348.69	34	34	6.39E-35	0.990	0.997	v5.3	HCP
LH_superiorfrontal_surfavg	ICC2	0.9574	58.13	34	34	6.83E-22	0.906	0.978	v5.3 vs. v6.0	HCP
LH_superiorfrontal_surfavg	ICC2	0.8573	18.36	34	34	6.96E-14	0.655	0.931	v5.3 vs. v7.1	HCP
LH_superiorfrontal_surfavg	ICC3	0.9945	359.72	34	34	3.77E-35	0.990	0.997	v6.0	HCP
LH_superiorfrontal_surfavg	ICC2	0.9173	26.83	34	34	1.86E-16	0.844	0.955	v6.0 vs. v7.1	HCP
LH_superiorfrontal_surfavg	ICC3	0.9966	594.28	34	34	7.69E-39	0.994	0.998	v7.1	HCP
LH_superiorfrontal_thickavg	ICC3	0.9099	21.20	34	34	7.54E-15	0.846	0.948	v5.3	HCP
LH_superiorfrontal_thickavg	ICC2	0.9649	60.46	34	34	3.58E-22	0.937	0.980	v5.3 vs. v6.0	HCP
LH_superiorfrontal_thickavg	ICC2	0.8218	40.33	34	34	2.69E-19	0.052	0.941	v5.3 vs. v7.1	HCP
LH_superiorfrontal_thickavg	ICC3	0.9149	22.50	34	34	2.99E-15	0.854	0.951	v6.0	HCP
LH_superiorfrontal_thickavg	ICC2	0.8602	57.33	34	34	8.59E-22	0.079	0.956	v6.0 vs. v7.1	HCP
LH_superiorfrontal_thickavg	ICC3	0.9461	36.09	34	34	1.62E-18	0.906	0.969	v7.1	HCP
LH_superiorparietal_surfavg	ICC3	0.9886	174.84	34	34	7.29E-30	0.980	0.994	v5.3	HCP
LH_superiorparietal_surfavg	ICC2	0.9003	26.88	34	34	1.81E-16	0.748	0.952	v5.3 vs. v6.0	HCP

LH_superiorparietal_surfavg	ICC2	0.7535	7.81	34	34	1.66E-08	0.592	0.855	v5.3 vs. v7.1	HCP
LH_superiorparietal_surfavg	ICC3	0.9933	296.63	34	34	9.83E-34	0.988	0.996	v6.0	HCP
LH_superiorparietal_surfavg	ICC2	0.9010	18.68	34	34	5.33E-14	0.831	0.943	v6.0 vs. v7.1	HCP
LH_superiorparietal_surfavg	ICC3	0.9896	190.64	34	34	1.70E-30	0.982	0.994	v7.1	HCP
LH_superiorparietal_thickavg	ICC3	0.9580	46.60	34	34	2.57E-20	0.927	0.976	v5.3	HCP
LH_superiorparietal_thickavg	ICC2	0.8906	41.09	34	34	1.99E-19	0.403	0.960	v5.3 vs. v6.0	HCP
LH_superiorparietal_thickavg	ICC2	0.9481	41.41	34	34	1.75E-19	0.906	0.971	v5.3 vs. v7.1	HCP
LH_superiorparietal_thickavg	ICC3	0.9388	31.67	34	34	1.33E-17	0.894	0.965	v6.0	HCP
LH_superiorparietal_thickavg	ICC2	0.8705	85.22	34	34	1.22E-24	0.047	0.961	v6.0 vs. v7.1	HCP
LH_superiorparietal_thickavg	ICC3	0.9670	59.65	34	34	4.46E-22	0.942	0.981	v7.1	HCP
LH_superiortemporal_surfavg	ICC3	0.9803	100.44	34	34	7.87E-26	0.965	0.989	v5.3	HCP
LH_superiortemporal_surfavg	ICC2	0.8939	37.69	34	34	8.07E-19	0.492	0.960	v5.3 vs. v6.0	HCP
LH_superiortemporal_surfavg	ICC2	0.8563	46.41	34	34	2.74E-20	0.118	0.953	v5.3 vs. v7.1	HCP
LH_superiortemporal_surfavg	ICC3	0.9906	211.68	34	34	2.91E-31	0.983	0.995	v6.0	HCP
LH_superiortemporal_surfavg	ICC2	0.9832	242.37	34	34	2.97E-32	0.895	0.994	v6.0 vs. v7.1	HCP
LH_superiortemporal_surfavg	ICC3	0.9901	201.23	34	34	6.84E-31	0.983	0.994	v7.1	HCP
LH_superiortemporal_thickavg	ICC3	0.9263	26.13	34	34	2.83E-16	0.873	0.958	v5.3	HCP
LH_superiortemporal_thickavg	ICC2	0.9382	30.51	34	34	2.41E-17	0.893	0.965	v5.3 vs. v6.0	HCP
LH_superiortemporal_thickavg	ICC2	0.7230	26.05	34	34	2.98E-16	-0.015	0.903	v5.3 vs. v7.1	HCP
LH_superiortemporal_thickavg	ICC3	0.9105	21.36	34	34	6.72E-15	0.847	0.949	v6.0	HCP
LH_superiortemporal_thickavg	ICC2	0.7327	46.62	34	34	2.55E-20	-0.019	0.913	v6.0 vs. v7.1	HCP
LH_superiortemporal_thickavg	ICC3	0.9379	31.21	34	34	1.68E-17	0.893	0.964	v7.1	HCP
LH_supramarginal_surfavg	ICC3	0.9944	356.94	34	34	4.30E-35	0.990	0.997	v5.3	HCP
LH_supramarginal_surfavg	ICC2	0.9040	25.58	34	34	3.96E-16	0.790	0.951	v5.3 vs. v6.0	HCP
LH_supramarginal_surfavg	ICC2	0.8723	17.51	34	34	1.44E-13	0.756	0.931	v5.3 vs. v7.1	HCP
LH_supramarginal_surfavg	ICC3	0.9967	614.09	34	34	4.41E-39	0.994	0.998	v6.0	HCP
LH_supramarginal_surfavg	ICC2	0.9826	110.87	34	34	1.51E-26	0.969	0.990	v6.0 vs. v7.1	HCP
LH_supramarginal_surfavg	ICC3	0.9967	598.79	34	34	6.76E-39	0.994	0.998	v7.1	HCP
LH_supramarginal_thickavg	ICC3	0.9218	24.56	34	34	7.52E-16	0.865	0.955	v5.3	HCP
LH_supramarginal_thickavg	ICC2	0.9042	20.77	34	34	1.03E-14	0.836	0.945	v5.3 vs. v6.0	HCP
LH_supramarginal_thickavg	ICC2	0.7904	16.62	34	34	3.17E-13	0.304	0.913	v5.3 vs. v7.1	HCP
LH_supramarginal_thickavg	ICC3	0.9514	40.14	34	34	2.90E-19	0.915	0.972	v6.0	HCP
LH_supramarginal_thickavg	ICC2	0.7917	39.96	34	34	3.13E-19	0.009	0.932	v6.0 vs. v7.1	HCP
LH_supramarginal_thickavg	ICC3	0.9441	34.75	34	34	2.99E-18	0.903	0.968	v7.1	HCP
LH_SurfArea	ICC3	0.9984	1250.12	34	34	2.56E-44	0.997	0.999	v5.3	HCP
LH_SurfArea	ICC2	0.9943	1331.75	34	34	8.74E-45	0.869	0.998	v5.3 vs. v6.0	HCP
LH_SurfArea	ICC2	0.9797	1075.59	34	34	3.28E-43	0.295	0.995	v5.3 vs. v7.1	HCP
LH_SurfArea	ICC3	0.9982	1088.05	34	34	2.70E-43	0.997	0.999	v6.0	HCP
LH_SurfArea	ICC2	0.9942	2822.78	34	34	2.52E-50	0.677	0.998	v6.0 vs. v7.1	HCP
LH_SurfArea	ICC3	0.9985	1318.29	34	34	1.04E-44	0.997	0.999	v7.1	HCP
LH_temporalpole_surfavg	ICC3	0.8467	12.05	34	34	3.81E-11	0.744	0.911	v5.3	HCP
LH_temporalpole_surfavg	ICC2	0.7107	5.88	34	34	6.44E-07	0.540	0.825	v5.3 vs. v6.0	HCP
LH_temporalpole_surfavg	ICC2	0.6852	7.10	34	34	5.86E-08	0.400	0.829	v5.3 vs. v7.1	HCP
LH_temporalpole_surfavg	ICC3	0.8260	10.50	34	34	2.78E-10	0.711	0.898	v6.0	HCP

LH_temporalpole_surfavg	ICC2	0.7848	11.51	34	34	7.42E-11	0.528	0.891	v6.0 vs. v7.1	HCP
LH_temporalpole_surfavg	ICC3	0.8497	12.31	34	34	2.79E-11	0.748	0.912	v7.1	HCP
LH_temporalpole_thickavg	ICC3	0.6975	5.61	34	34	1.14E-06	0.520	0.817	v5.3	HCP
LH_temporalpole_thickavg	ICC2	0.8544	13.56	34	34	6.71E-12	0.753	0.916	v5.3 vs. v6.0	HCP
LH_temporalpole_thickavg	ICC2	0.7053	9.29	34	34	1.54E-09	0.299	0.859	v5.3 vs. v7.1	HCP
LH_temporalpole_thickavg	ICC3	0.8435	11.78	34	34	5.27E-11	0.739	0.909	v6.0	HCP
LH_temporalpole_thickavg	ICC2	0.8389	18.17	34	34	8.13E-14	0.542	0.927	v6.0 vs. v7.1	HCP
LH_temporalpole_thickavg	ICC3	0.7749	7.88	34	34	1.45E-08	0.633	0.866	v7.1	HCP
LH_Thalamus	ICC3	0.9007	19.13	34	34	3.69E-14	0.830	0.943	v5.3	HCP
LH_Thalamus	ICC2	0.7799	14.94	34	34	1.60E-12	0.321	0.906	v5.3 vs. v6.0	HCP
LH_Thalamus	ICC2	0.7488	11.62	34	34	6.49E-11	0.339	0.885	v5.3 vs. v7.1	HCP
LH_Thalamus	ICC3	0.8684	14.20	34	34	3.41E-12	0.778	0.924	v6.0	HCP
LH_Thalamus	ICC2	0.8858	16.13	34	34	5.02E-13	0.806	0.934	v6.0 vs. v7.1	HCP
LH_Thalamus	ICC3	0.8611	13.40	34	34	8.05E-12	0.766	0.919	v7.1	HCP
LH_Thickness	ICC3	0.9411	32.95	34	34	7.02E-18	0.898	0.966	v5.3	HCP
LH_Thickness	ICC2	0.9561	58.15	34	34	6.79E-22	0.898	0.978	v5.3 vs. v6.0	HCP
LH_Thickness	ICC2	0.7893	39.27	34	34	4.15E-19	0.008	0.931	v5.3 vs. v7.1	HCP
LH_Thickness	ICC3	0.9279	26.74	34	34	1.96E-16	0.876	0.959	v6.0	HCP
LH_Thickness	ICC2	0.7212	77.19	34	34	6.30E-24	-0.011	0.911	v6.0 vs. v7.1	HCP
LH_Thickness	ICC3	0.9480	37.43	34	34	9.00E-19	0.910	0.970	v7.1	HCP
LH_transversetemporal_surfavg	ICC3	0.9494	38.56	34	34	5.58E-19	0.912	0.971	v5.3	HCP
LH_transversetemporal_surfavg	ICC2	0.8469	12.81	34	34	1.56E-11	0.742	0.911	v5.3 vs. v6.0	HCP
LH_transversetemporal_surfavg	ICC2	0.8442	11.74	34	34	5.57E-11	0.741	0.909	v5.3 vs. v7.1	HCP
LH_transversetemporal_surfavg	ICC3	0.9318	28.34	34	34	7.83E-17	0.882	0.961	v6.0	HCP
LH_transversetemporal_surfavg	ICC2	0.9061	33.82	34	34	4.63E-18	0.679	0.960	v6.0 vs. v7.1	HCP
LH_transversetemporal_surfavg	ICC3	0.9006	19.13	34	34	3.70E-14	0.830	0.943	v7.1	HCP
LH_transversetemporal_thickavg	ICC3	0.7826	8.20	34	34	8.55E-09	0.645	0.871	v5.3	HCP
LH_transversetemporal_thickavg	ICC2	0.8974	18.17	34	34	8.16E-14	0.825	0.941	v5.3 vs. v6.0	HCP
LH_transversetemporal_thickavg	ICC2	0.8485	14.09	34	34	3.80E-12	0.726	0.915	v5.3 vs. v7.1	HCP
LH_transversetemporal_thickavg	ICC3	0.8981	18.62	34	34	5.59E-14	0.826	0.941	v6.0	HCP
LH_transversetemporal_thickavg	ICC2	0.8882	23.83	34	34	1.21E-15	0.721	0.946	v6.0 vs. v7.1	HCP
LH_transversetemporal_thickavg	ICC3	0.8766	15.20	34	34	1.22E-12	0.791	0.928	v7.1	HCP
RH_Accumbens	ICC3	0.8429	11.73	34	34	5.64E-11	0.737	0.908	v5.3	HCP
RH_Accumbens	ICC2	0.8328	11.09	34	34	1.26E-10	0.723	0.902	v5.3 vs. v6.0	HCP
RH_Accumbens	ICC2	0.8295	10.46	34	34	2.93E-10	0.716	0.900	v5.3 vs. v7.1	HCP
RH_Accumbens	ICC3	0.8713	14.54	34	34	2.39E-12	0.783	0.925	v6.0	HCP
RH_Accumbens	ICC2	0.8927	18.51	34	34	6.13E-14	0.817	0.938	v6.0 vs. v7.1	HCP
RH_Accumbens	ICC3	0.8760	15.12	34	34	1.32E-12	0.790	0.928	v7.1	HCP
RH_Amygdala	ICC3	0.7972	8.86	34	34	2.98E-09	0.667	0.880	v5.3	HCP
RH_Amygdala	ICC2	0.6565	17.31	34	34	1.71E-13	-0.029	0.869	v5.3 vs. v6.0	HCP
RH_Amygdala	ICC2	0.6662	18.84	34	34	4.69E-14	-0.029	0.875	v5.3 vs. v7.1	HCP
RH_Amygdala	ICC3	0.9168	23.04	34	34	2.06E-15	0.857	0.952	v6.0	HCP
RH_Amygdala	ICC2	0.9438	34.65	34	34	3.13E-18	0.903	0.968	v6.0 vs. v7.1	HCP
RH_Amygdala	ICC3	0.9344	29.49	34	34	4.15E-17	0.887	0.962	v7.1	HCP

RH_bankssts_surfavg	ICC3	0.9703	66.28	34	34	7.85E-23	0.948	0.983	v5.3	HCP
RH_bankssts_surfavg	ICC2	0.7571	10.70	34	34	2.12E-10	0.438	0.881	v5.3 vs. v6.0	HCP
RH_bankssts_surfavg	ICC2	0.7652	9.62	34	34	9.51E-10	0.544	0.874	v5.3 vs. v7.1	HCP
RH_bankssts_surfavg	ICC3	0.9724	71.54	34	34	2.22E-23	0.952	0.984	v6.0	HCP
RH_bankssts_surfavg	ICC2	0.9689	67.39	34	34	5.97E-23	0.945	0.982	v6.0 vs. v7.1	HCP
RH_bankssts_surfavg	ICC3	0.9843	126.18	34	34	1.74E-27	0.972	0.991	v7.1	HCP
RH_bankssts_thickavg	ICC3	0.9406	32.68	34	34	8.00E-18	0.897	0.966	v5.3	HCP
RH_bankssts_thickavg	ICC2	0.9329	29.06	34	34	5.25E-17	0.885	0.961	v5.3 vs. v6.0	HCP
RH_bankssts_thickavg	ICC2	0.8222	24.26	34	34	9.15E-16	0.240	0.933	v5.3 vs. v7.1	HCP
RH_bankssts_thickavg	ICC3	0.9506	39.46	34	34	3.83E-19	0.914	0.972	v6.0	HCP
RH_bankssts_thickavg	ICC2	0.8664	41.55	34	34	1.66E-19	0.209	0.954	v6.0 vs. v7.1	HCP
RH_bankssts_thickavg	ICC3	0.9513	40.10	34	34	2.96E-19	0.915	0.972	v7.1	HCP
RH_caudalanteriorcingulate_surfavg	ICC3	0.9664	58.57	34	34	6.04E-22	0.941	0.981	v5.3	HCP
RH_caudalanteriorcingulate_surfavg	ICC2	0.8011	31.84	34	34	1.22E-17	0.055	0.932	v5.3 vs. v6.0	HCP
RH_caudalanteriorcingulate_surfavg	ICC2	0.9083	36.22	34	34	1.53E-18	0.661	0.962	v5.3 vs. v7.1	HCP
RH_caudalanteriorcingulate_surfavg	ICC3	0.9775	87.83	34	34	7.37E-25	0.960	0.987	v6.0	HCP
RH_caudalanteriorcingulate_surfavg	ICC2	0.9399	96.99	34	34	1.41E-25	0.451	0.980	v6.0 vs. v7.1	HCP
RH_caudalanteriorcingulate_surfavg	ICC3	0.9639	54.44	34	34	2.01E-21	0.937	0.979	v7.1	HCP
RH_caudalanteriorcingulate_thickavg	ICC3	0.7110	5.92	34	34	5.87E-07	0.539	0.826	v5.3	HCP
RH_caudalanteriorcingulate_thickavg	ICC2	0.7331	6.45	34	34	2.01E-07	0.573	0.840	v5.3 vs. v6.0	HCP
RH_caudalanteriorcingulate_thickavg	ICC2	0.4013	6.09	34	34	4.11E-07	-0.065	0.697	v5.3 vs. v7.1	HCP
RH_caudalanteriorcingulate_thickavg	ICC3	0.8082	9.43	34	34	1.25E-09	0.684	0.887	v6.0	HCP
RH_caudalanteriorcingulate_thickavg	ICC2	0.4085	7.33	34	34	3.86E-08	-0.064	0.710	v6.0 vs. v7.1	HCP
RH_caudalanteriorcingulate_thickavg	ICC3	0.7790	8.05	34	34	1.09E-08	0.639	0.869	v7.1	HCP
RH_caudalmiddlefrontal_surfavg	ICC3	0.9881	167.66	34	34	1.47E-29	0.979	0.993	v5.3	HCP
RH_caudalmiddlefrontal_surfavg	ICC2	0.9741	92.19	34	34	3.29E-25	0.946	0.986	v5.3 vs. v6.0	HCP
RH_caudalmiddlefrontal_surfavg	ICC2	0.9679	62.92	34	34	1.85E-22	0.944	0.982	v5.3 vs. v7.1	HCP
RH_caudalmiddlefrontal_surfavg	ICC3	0.9936	312.82	34	34	4.00E-34	0.989	0.996	v6.0	HCP
RH_caudalmiddlefrontal_surfavg	ICC2	0.9921	274.76	34	34	3.58E-33	0.986	0.996	v6.0 vs. v7.1	HCP
RH_caudalmiddlefrontal_surfavg	ICC3	0.9942	343.94	34	34	8.06E-35	0.990	0.997	v7.1	HCP
RH_caudalmiddlefrontal_thickavg	ICC3	0.8646	13.77	34	34	5.38E-12	0.772	0.921	v5.3	HCP
RH_caudalmiddlefrontal_thickavg	ICC2	0.9099	27.84	34	34	1.04E-16	0.796	0.955	v5.3 vs. v6.0	HCP
RH_caudalmiddlefrontal_thickavg	ICC2	0.8187	16.33	34	34	4.14E-13	0.485	0.918	v5.3 vs. v7.1	HCP
RH_caudalmiddlefrontal_thickavg	ICC3	0.8937	17.82	34	34	1.10E-13	0.819	0.939	v6.0	HCP
RH_caudalmiddlefrontal_thickavg	ICC2	0.7752	37.99	34	34	7.09E-19	-0.001	0.926	v6.0 vs. v7.1	HCP
RH_caudalmiddlefrontal_thickavg	ICC3	0.9117	21.64	34	34	5.47E-15	0.849	0.949	v7.1	HCP
RH_Caudate	ICC3	0.9602	49.25	34	34	1.04E-20	0.931	0.977	v5.3	HCP
RH_Caudate	ICC2	0.7455	36.64	34	34	1.27E-18	-0.016	0.915	v5.3 vs. v6.0	HCP
RH_Caudate	ICC2	0.7597	33.38	34	34	5.70E-18	-0.005	0.919	v5.3 vs. v7.1	HCP
RH_Caudate	ICC3	0.9828	115.26	34	34	7.91E-27	0.970	0.990	v6.0	HCP
RH_Caudate	ICC2	0.9783	96.03	34	34	1.67E-25	0.961	0.988	v6.0 vs. v7.1	HCP
RH_Caudate	ICC3	0.9733	73.85	34	34	1.31E-23	0.953	0.985	v7.1	HCP
RH_cuneus_surfavg	ICC3	0.9552	43.61	34	34	7.54E-20	0.922	0.974	v5.3	HCP
RH_cuneus_surfavg	ICC2	0.6406	6.96	34	34	7.55E-08	0.247	0.817	v5.3 vs. v6.0	HCP

RH_cuneus_surfavg	ICC2	0.5806	6.78	34	34	1.05E-07	0.086	0.796	v5.3 vs. v7.1	HCP
RH_cuneus_surfavg	ICC3	0.9874	158.06	34	34	3.97E-29	0.978	0.993	v6.0	HCP
RH_cuneus_surfavg	ICC2	0.9787	176.22	34	34	6.39E-30	0.889	0.992	v6.0 vs. v7.1	HCP
RH_cuneus_surfavg	ICC3	0.9834	119.76	34	34	4.17E-27	0.971	0.991	v7.1	HCP
RH_cuneus_thickavg	ICC3	0.9218	24.56	34	34	7.52E-16	0.865	0.955	v5.3	HCP
RH_cuneus_thickavg	ICC2	0.8491	17.36	34	34	1.64E-13	0.636	0.927	v5.3 vs. v6.0	HCP
RH_cuneus_thickavg	ICC2	0.8816	17.59	34	34	1.34E-13	0.791	0.933	v5.3 vs. v7.1	HCP
RH_cuneus_thickavg	ICC3	0.9314	28.17	34	34	8.60E-17	0.882	0.961	v6.0	HCP
RH_cuneus_thickavg	ICC2	0.8299	30.62	34	34	2.28E-17	0.160	0.940	v6.0 vs. v7.1	HCP
RH_cuneus_thickavg	ICC3	0.9483	37.66	34	34	8.16E-19	0.910	0.970	v7.1	HCP
RH_entorhinal_surfavg	ICC3	0.8685	14.21	34	34	3.35E-12	0.778	0.924	v5.3	HCP
RH_entorhinal_surfavg	ICC2	0.7926	11.01	34	34	1.40E-10	0.591	0.889	v5.3 vs. v6.0	HCP
RH_entorhinal_surfavg	ICC2	0.7730	8.92	34	34	2.71E-09	0.609	0.869	v5.3 vs. v7.1	HCP
RH_entorhinal_surfavg	ICC3	0.9179	23.36	34	34	1.65E-15	0.859	0.953	v6.0	HCP
RH_entorhinal_surfavg	ICC2	0.9510	40.69	34	34	2.33E-19	0.915	0.972	v6.0 vs. v7.1	HCP
RH_entorhinal_surfavg	ICC3	0.9209	24.29	34	34	8.98E-16	0.864	0.955	v7.1	HCP
RH_entorhinal_thickavg	ICC3	0.8303	10.79	34	34	1.88E-10	0.718	0.901	v5.3	HCP
RH_entorhinal_thickavg	ICC2	0.9082	22.66	34	34	2.67E-15	0.838	0.948	v5.3 vs. v6.0	HCP
RH_entorhinal_thickavg	ICC2	0.5461	12.79	34	34	1.58E-11	-0.052	0.812	v5.3 vs. v7.1	HCP
RH_entorhinal_thickavg	ICC3	0.8623	13.53	34	34	6.97E-12	0.768	0.920	v6.0	HCP
RH_entorhinal_thickavg	ICC2	0.6293	27.19	34	34	1.51E-16	-0.034	0.865	v6.0 vs. v7.1	HCP
RH_entorhinal_thickavg	ICC3	0.8587	13.15	34	34	1.05E-11	0.763	0.918	v7.1	HCP
RH_frontalpole_surfavg	ICC3	0.8359	11.19	34	34	1.11E-10	0.727	0.904	v5.3	HCP
RH_frontalpole_surfavg	ICC2	0.5029	7.34	34	34	3.75E-08	-0.042	0.767	v5.3 vs. v6.0	HCP
RH_frontalpole_surfavg	ICC2	0.2986	7.02	34	34	6.68E-08	-0.053	0.616	v5.3 vs. v7.1	HCP
RH_frontalpole_surfavg	ICC3	0.7594	7.31	34	34	3.94E-08	0.610	0.857	v6.0	HCP
RH_frontalpole_surfavg	ICC2	0.5636	5.95	34	34	5.53E-07	0.111	0.778	v6.0 vs. v7.1	HCP
RH_frontalpole_surfavg	ICC3	0.7833	8.23	34	34	8.16E-09	0.646	0.872	v7.1	HCP
RH_frontalpole_thickavg	ICC3	0.8185	10.02	34	34	5.38E-10	0.699	0.893	v5.3	HCP
RH_frontalpole_thickavg	ICC2	0.8008	9.58	34	34	1.01E-09	0.670	0.883	v5.3 vs. v6.0	HCP
RH_frontalpole_thickavg	ICC2	0.6731	7.70	34	34	1.99E-08	0.298	0.835	v5.3 vs. v7.1	HCP
RH_frontalpole_thickavg	ICC3	0.8227	10.28	34	34	3.73E-10	0.706	0.896	v6.0	HCP
RH_frontalpole_thickavg	ICC2	0.8271	17.89	34	34	1.03E-13	0.475	0.924	v6.0 vs. v7.1	HCP
RH_frontalpole_thickavg	ICC3	0.8525	12.56	34	34	2.08E-11	0.753	0.914	v7.1	HCP
RH_fusiform_surfavg	ICC3	0.9717	69.60	34	34	3.50E-23	0.950	0.984	v5.3	HCP
RH_fusiform_surfavg	ICC2	0.8903	32.35	34	34	9.45E-18	0.561	0.956	v5.3 vs. v6.0	HCP
RH_fusiform_surfavg	ICC2	0.8947	26.31	34	34	2.54E-16	0.719	0.951	v5.3 vs. v7.1	HCP
RH_fusiform_surfavg	ICC3	0.9915	235.53	34	34	4.82E-32	0.985	0.995	v6.0	HCP
RH_fusiform_surfavg	ICC2	0.9885	212.23	34	34	2.79E-31	0.975	0.994	v6.0 vs. v7.1	HCP
RH_fusiform_surfavg	ICC3	0.9914	230.89	34	34	6.74E-32	0.985	0.995	v7.1	HCP
RH_fusiform_thickavg	ICC3	0.8950	18.04	34	34	9.09E-14	0.821	0.939	v5.3	HCP
RH_fusiform_thickavg	ICC2	0.8997	18.48	34	34	6.32E-14	0.829	0.942	v5.3 vs. v6.0	HCP
RH_fusiform_thickavg	ICC2	0.7391	25.03	34	34	5.60E-16	0.001	0.908	v5.3 vs. v7.1	HCP
RH_fusiform_thickavg	ICC3	0.9105	21.34	34	34	6.79E-15	0.847	0.948	v6.0	HCP

RH_fusiform_thickavg	ICC2	0.7341	44.69	34	34	5.07E-20	-0.019	0.913	v6.0 vs. v7.1	HCP
RH_fusiform_thickavg	ICC3	0.9288	27.08	34	34	1.61E-16	0.877	0.959	v7.1	HCP
RH_Hippocampus	ICC3	0.4952	2.96	34	34	1.07E-03	0.251	0.680	v5.3	HCP
RH_Hippocampus	ICC2	0.9547	54.81	34	34	1.80E-21	0.899	0.977	v5.3 vs. v6.0	HCP
RH_Hippocampus	ICC2	0.9650	54.90	34	34	1.75E-21	0.939	0.980	v5.3 vs. v7.1	HCP
RH_Hippocampus	ICC3	0.9656	57.10	34	34	9.16E-22	0.940	0.980	v6.0	HCP
RH_Hippocampus	ICC2	0.9633	66.08	34	34	8.24E-23	0.922	0.981	v6.0 vs. v7.1	HCP
RH_Hippocampus	ICC3	0.9719	70.05	34	34	3.14E-23	0.951	0.984	v7.1	HCP
RH_inferiorparietal_surfavg	ICC3	0.9882	169.19	34	34	1.27E-29	0.979	0.993	v5.3	HCP
RH_inferiorparietal_surfavg	ICC2	0.8839	20.00	34	34	1.86E-14	0.767	0.938	v5.3 vs. v6.0	HCP
RH_inferiorparietal_surfavg	ICC2	0.8957	18.93	34	34	4.34E-14	0.822	0.940	v5.3 vs. v7.1	HCP
RH_inferiorparietal_surfavg	ICC3	0.9957	465.07	34	34	4.89E-37	0.992	0.998	v6.0	HCP
RH_inferiorparietal_surfavg	ICC2	0.9903	383.89	34	34	1.26E-35	0.950	0.996	v6.0 vs. v7.1	HCP
RH_inferiorparietal_surfavg	ICC3	0.9960	498.97	34	34	1.49E-37	0.993	0.998	v7.1	HCP
RH_inferiorparietal_thickavg	ICC3	0.9206	24.18	34	34	9.66E-16	0.863	0.954	v5.3	HCP
RH_inferiorparietal_thickavg	ICC2	0.9427	35.27	34	34	2.35E-18	0.900	0.967	v5.3 vs. v6.0	HCP
RH_inferiorparietal_thickavg	ICC2	0.7481	22.77	34	34	2.48E-15	0.025	0.909	v5.3 vs. v7.1	HCP
RH_inferiorparietal_thickavg	ICC3	0.9467	36.54	34	34	1.33E-18	0.907	0.970	v6.0	HCP
RH_inferiorparietal_thickavg	ICC2	0.8136	52.30	34	34	3.87E-21	0.012	0.941	v6.0 vs. v7.1	HCP
RH_inferiorparietal_thickavg	ICC3	0.9283	26.88	34	34	1.81E-16	0.876	0.959	v7.1	HCP
RH_inferiortemporal_surfavg	ICC3	0.9899	196.38	34	34	1.03E-30	0.982	0.994	v5.3	HCP
RH_inferiortemporal_surfavg	ICC2	0.9359	56.39	34	34	1.13E-21	0.713	0.975	v5.3 vs. v6.0	HCP
RH_inferiortemporal_surfavg	ICC2	0.9182	57.19	34	34	8.93E-22	0.483	0.971	v5.3 vs. v7.1	HCP
RH_inferiortemporal_surfavg	ICC3	0.9854	135.60	34	34	5.20E-28	0.974	0.992	v6.0	HCP
RH_inferiortemporal_surfavg	ICC2	0.9859	157.72	34	34	4.12E-29	0.974	0.992	v6.0 vs. v7.1	HCP
RH_inferiortemporal_surfavg	ICC3	0.9854	135.68	34	34	5.15E-28	0.974	0.992	v7.1	HCP
RH_inferiortemporal_thickavg	ICC3	0.8965	18.32	34	34	7.17E-14	0.824	0.940	v5.3	HCP
RH_inferiortemporal_thickavg	ICC2	0.8879	25.23	34	34	4.93E-16	0.690	0.948	v5.3 vs. v6.0	HCP
RH_inferiortemporal_thickavg	ICC2	0.6050	16.90	34	34	2.47E-13	-0.044	0.847	v5.3 vs. v7.1	HCP
RH_inferiortemporal_thickavg	ICC3	0.8837	16.19	34	34	4.73E-13	0.803	0.933	v6.0	HCP
RH_inferiortemporal_thickavg	ICC2	0.7566	32.22	34	34	1.01E-17	-0.005	0.918	v6.0 vs. v7.1	HCP
RH_inferiortemporal_thickavg	ICC3	0.9141	22.28	34	34	3.48E-15	0.853	0.951	v7.1	HCP
RH_insula_surfavg	ICC3	0.6465	4.66	34	34	1.02E-05	0.449	0.784	v5.3	HCP
RH_insula_surfavg	ICC2	0.7585	7.11	34	34	5.70E-08	0.608	0.856	v5.3 vs. v6.0	HCP
RH_insula_surfavg	ICC2	0.7979	10.13	34	34	4.60E-10	0.648	0.884	v5.3 vs. v7.1	HCP
RH_insula_surfavg	ICC3	0.7642	7.48	34	34	2.91E-08	0.617	0.860	v6.0	HCP
RH_insula_surfavg	ICC2	0.8154	11.09	34	34	1.26E-10	0.679	0.894	v6.0 vs. v7.1	HCP
RH_insula_surfavg	ICC3	0.8694	14.32	34	34	3.00E-12	0.780	0.924	v7.1	HCP
RH_insula_thickavg	ICC3	0.7533	7.11	34	34	5.74E-08	0.601	0.853	v5.3	HCP
RH_insula_thickavg	ICC2	0.7624	8.32	34	34	6.98E-09	0.599	0.862	v5.3 vs. v6.0	HCP
RH_insula_thickavg	ICC2	0.7950	10.18	34	34	4.30E-10	0.636	0.884	v5.3 vs. v7.1	HCP
RH_insula_thickavg	ICC3	0.8159	9.86	34	34	6.69E-10	0.695	0.892	v6.0	HCP
RH_insula_thickavg	ICC2	0.6832	7.77	34	34	1.76E-08	0.331	0.838	v6.0 vs. v7.1	HCP
RH_insula_thickavg	ICC3	0.8297	10.74	34	34	1.99E-10	0.717	0.900	v7.1	HCP

RH_isthmuscingulate_surfavg	ICC3	0.9134	22.09	34	34	3.96E-15	0.851	0.950	v5.3	HCP
RH_isthmuscingulate_surfavg	ICC2	0.8491	14.90	34	34	1.65E-12	0.709	0.918	v5.3 vs. v6.0	HCP
RH_isthmuscingulate_surfavg	ICC2	0.8501	12.41	34	34	2.49E-11	0.750	0.912	v5.3 vs. v7.1	HCP
RH_isthmuscingulate_surfavg	ICC3	0.9788	93.19	34	34	2.75E-25	0.963	0.988	v6.0	HCP
RH_isthmuscingulate_surfavg	ICC2	0.9661	87.11	34	34	8.45E-25	0.895	0.985	v6.0 vs. v7.1	HCP
RH_isthmuscingulate_surfavg	ICC3	0.9766	84.55	34	34	1.39E-24	0.959	0.987	v7.1	HCP
RH_isthmuscingulate_thickavg	ICC3	0.8842	16.28	34	34	4.37E-13	0.804	0.933	v5.3	HCP
RH_isthmuscingulate_thickavg	ICC2	0.8144	9.96	34	34	5.81E-10	0.695	0.891	v5.3 vs. v6.0	HCP
RH_isthmuscingulate_thickavg	ICC2	0.3545	7.77	34	34	1.78E-08	-0.058	0.671	v5.3 vs. v7.1	HCP
RH_isthmuscingulate_thickavg	ICC3	0.8640	13.71	34	34	5.73E-12	0.771	0.921	v6.0	HCP
RH_isthmuscingulate_thickavg	ICC2	0.4274	18.38	34	34	6.86E-14	-0.033	0.745	v6.0 vs. v7.1	HCP
RH_isthmuscingulate_thickavg	ICC3	0.9248	25.58	34	34	3.95E-16	0.870	0.957	v7.1	HCP
RH_lateraloccipital_surfavg	ICC3	0.9589	47.61	34	34	1.80E-20	0.928	0.977	v5.3	HCP
RH_lateraloccipital_surfavg	ICC2	0.7089	8.79	34	34	3.31E-09	0.353	0.855	v5.3 vs. v6.0	HCP
RH_lateraloccipital_surfavg	ICC2	0.6967	9.84	34	34	6.88E-10	0.226	0.861	v5.3 vs. v7.1	HCP
RH_lateraloccipital_surfavg	ICC3	0.9933	295.42	34	34	1.05E-33	0.988	0.996	v6.0	HCP
RH_lateraloccipital_surfavg	ICC2	0.9832	136.60	34	34	4.60E-28	0.967	0.991	v6.0 vs. v7.1	HCP
RH_lateraloccipital_surfavg	ICC3	0.9910	221.42	34	34	1.37E-31	0.984	0.995	v7.1	HCP
RH_lateraloccipital_thickavg	ICC3	0.9602	49.29	34	34	1.02E-20	0.931	0.977	v5.3	HCP
RH_lateraloccipital_thickavg	ICC2	0.9481	44.14	34	34	6.20E-20	0.898	0.972	v5.3 vs. v6.0	HCP
RH_lateraloccipital_thickavg	ICC2	0.8615	29.16	34	34	4.97E-17	0.382	0.947	v5.3 vs. v7.1	HCP
RH_lateraloccipital_thickavg	ICC3	0.9348	29.66	34	34	3.79E-17	0.887	0.963	v6.0	HCP
RH_lateraloccipital_thickavg	ICC2	0.8295	89.63	34	34	5.25E-25	0.006	0.949	v6.0 vs. v7.1	HCP
RH_lateraloccipital_thickavg	ICC3	0.9647	55.61	34	34	1.42E-21	0.938	0.980	v7.1	HCP
RH_lateralorbitofrontal_surfavg	ICC3	0.8552	12.81	34	34	1.55E-11	0.757	0.916	v5.3	HCP
RH_lateralorbitofrontal_surfavg	ICC2	0.8779	15.20	34	34	1.22E-12	0.794	0.929	v5.3 vs. v6.0	HCP
RH_lateralorbitofrontal_surfavg	ICC2	0.7900	10.85	34	34	1.72E-10	0.587	0.888	v5.3 vs. v7.1	HCP
RH_lateralorbitofrontal_surfavg	ICC3	0.8316	10.88	34	34	1.67E-10	0.720	0.901	v6.0	HCP
RH_lateralorbitofrontal_surfavg	ICC2	0.9066	30.10	34	34	3.00E-17	0.742	0.957	v6.0 vs. v7.1	HCP
RH_lateralorbitofrontal_surfavg	ICC3	0.9022	19.46	34	34	2.85E-14	0.833	0.944	v7.1	HCP
RH_lateralorbitofrontal_thickavg	ICC3	0.8663	13.96	34	34	4.39E-12	0.775	0.922	v5.3	HCP
RH_lateralorbitofrontal_thickavg	ICC2	0.8505	15.86	34	34	6.48E-13	0.690	0.922	v5.3 vs. v6.0	HCP
RH_lateralorbitofrontal_thickavg	ICC2	0.7029	9.74	34	34	7.96E-10	0.257	0.862	v5.3 vs. v7.1	HCP
RH_lateralorbitofrontal_thickavg	ICC3	0.8530	12.60	34	34	1.98E-11	0.753	0.914	v6.0	HCP
RH_lateralorbitofrontal_thickavg	ICC2	0.6055	12.64	34	34	1.90E-11	-0.035	0.839	v6.0 vs. v7.1	HCP
RH_lateralorbitofrontal_thickavg	ICC3	0.7962	8.82	34	34	3.19E-09	0.665	0.880	v7.1	HCP
RH_LateralVentricle	ICC3	0.9941	335.36	34	34	1.24E-34	0.989	0.997	v5.3	HCP
RH_LateralVentricle	ICC2	0.9656	435.79	34	34	1.47E-36	0.244	0.991	v5.3 vs. v6.0	HCP
RH_LateralVentricle	ICC2	0.9634	349.75	34	34	6.07E-35	0.267	0.990	v5.3 vs. v7.1	HCP
RH_LateralVentricle	ICC3	0.9952	417.76	34	34	3.01E-36	0.992	0.997	v6.0	HCP
RH_LateralVentricle	ICC2	0.9993	2986.76	34	34	9.64E-51	0.999	1.000	v6.0 vs. v7.1	HCP
RH_LateralVentricle	ICC3	0.9959	482.75	34	34	2.60E-37	0.993	0.998	v7.1	HCP
RH_lingual_surfavg	ICC3	0.9739	75.74	34	34	8.63E-24	0.954	0.985	v5.3	HCP
RH_lingual_surfavg	ICC2	0.7463	8.08	34	34	1.05E-08	0.558	0.855	v5.3 vs. v6.0	HCP

RH_lingual_surfavg	ICC2	0.7064	8.56	34	34	4.73E-09	0.360	0.852	v5.3 vs. v7.1	HCP
RH_lingual_surfavg	ICC3	0.9898	195.93	34	34	1.07E-30	0.982	0.994	v6.0	HCP
RH_lingual_surfavg	ICC2	0.9781	376.77	34	34	1.72E-35	0.573	0.994	v6.0 vs. v7.1	HCP
RH_lingual_surfavg	ICC3	0.9912	226.46	34	34	9.35E-32	0.984	0.995	v7.1	HCP
RH_lingual_thickavg	ICC3	0.9347	29.61	34	34	3.89E-17	0.887	0.963	v5.3	HCP
RH_lingual_thickavg	ICC2	0.7916	9.26	34	34	1.61E-09	0.653	0.878	v5.3 vs. v6.0	HCP
RH_lingual_thickavg	ICC2	0.6660	9.08	34	34	2.12E-09	0.166	0.846	v5.3 vs. v7.1	HCP
RH_lingual_thickavg	ICC3	0.8906	17.28	34	34	1.76E-13	0.814	0.937	v6.0	HCP
RH_lingual_thickavg	ICC2	0.6437	38.75	34	34	5.15E-19	-0.025	0.875	v6.0 vs. v7.1	HCP
RH_lingual_thickavg	ICC3	0.9384	31.48	34	34	1.46E-17	0.893	0.965	v7.1	HCP
RH_medialorbitofrontal_surfavg	ICC3	0.9535	42.02	34	34	1.38E-19	0.919	0.973	v5.3	HCP
RH_medialorbitofrontal_surfavg	ICC2	0.8286	16.70	34	34	2.97E-13	0.532	0.921	v5.3 vs. v6.0	HCP
RH_medialorbitofrontal_surfavg	ICC2	0.4974	13.91	34	34	4.63E-12	-0.050	0.786	v5.3 vs. v7.1	HCP
RH_medialorbitofrontal_surfavg	ICC3	0.9309	27.96	34	34	9.67E-17	0.881	0.960	v6.0	HCP
RH_medialorbitofrontal_surfavg	ICC2	0.6586	16.46	34	34	3.67E-13	-0.024	0.869	v6.0 vs. v7.1	HCP
RH_medialorbitofrontal_surfavg	ICC3	0.9470	36.74	34	34	1.22E-18	0.908	0.970	v7.1	HCP
RH_medialorbitofrontal_thickavg	ICC3	0.7694	7.67	34	34	2.08E-08	0.625	0.863	v5.3	HCP
RH_medialorbitofrontal_thickavg	ICC2	0.7039	10.65	34	34	2.25E-10	0.203	0.868	v5.3 vs. v6.0	HCP
RH_medialorbitofrontal_thickavg	ICC2	0.8402	11.76	34	34	5.41E-11	0.734	0.906	v5.3 vs. v7.1	HCP
RH_medialorbitofrontal_thickavg	ICC3	0.8668	14.02	34	34	4.10E-12	0.776	0.923	v6.0	HCP
RH_medialorbitofrontal_thickavg	ICC2	0.6332	12.01	34	34	4.01E-11	-0.006	0.849	v6.0 vs. v7.1	HCP
RH_medialorbitofrontal_thickavg	ICC3	0.8315	10.87	34	34	1.69E-10	0.720	0.901	v7.1	HCP
RH_middletemporal_surfavg	ICC3	0.9886	174.84	34	34	7.29E-30	0.980	0.994	v5.3	HCP
RH_middletemporal_surfavg	ICC2	0.9516	94.77	34	34	2.08E-25	0.659	0.983	v5.3 vs. v6.0	HCP
RH_middletemporal_surfavg	ICC2	0.9149	87.87	34	34	7.31E-25	0.219	0.974	v5.3 vs. v7.1	HCP
RH_middletemporal_surfavg	ICC3	0.9886	175.05	34	34	7.14E-30	0.980	0.994	v6.0	HCP
RH_middletemporal_surfavg	ICC2	0.9835	250.04	34	34	1.76E-32	0.892	0.994	v6.0 vs. v7.1	HCP
RH_middletemporal_surfavg	ICC3	0.9944	353.38	34	34	5.10E-35	0.990	0.997	v7.1	HCP
RH_middletemporal_thickavg	ICC3	0.9476	37.18	34	34	1.00E-18	0.909	0.970	v5.3	HCP
RH_middletemporal_thickavg	ICC2	0.9295	48.88	34	34	1.17E-20	0.714	0.971	v5.3 vs. v6.0	HCP
RH_middletemporal_thickavg	ICC2	0.6747	31.96	34	34	1.15E-17	-0.030	0.887	v5.3 vs. v7.1	HCP
RH_middletemporal_thickavg	ICC3	0.9556	44.08	34	34	6.33E-20	0.923	0.975	v6.0	HCP
RH_middletemporal_thickavg	ICC2	0.7962	50.82	34	34	6.21E-21	0.000	0.935	v6.0 vs. v7.1	HCP
RH_middletemporal_thickavg	ICC3	0.9571	45.64	34	34	3.59E-20	0.925	0.976	v7.1	HCP
RH_Pallidum	ICC3	0.7372	6.61	34	34	1.47E-07	0.577	0.843	v5.3	HCP
RH_Pallidum	ICC2	0.2449	5.66	34	34	1.02E-06	-0.053	0.551	v5.3 vs. v6.0	HCP
RH_Pallidum	ICC2	0.2115	7.53	34	34	2.66E-08	-0.034	0.514	v5.3 vs. v7.1	HCP
RH_Pallidum	ICC3	0.8087	9.46	34	34	1.21E-09	0.684	0.887	v6.0	HCP
RH_Pallidum	ICC2	0.8233	13.73	34	34	5.63E-12	0.622	0.909	v6.0 vs. v7.1	HCP
RH_Pallidum	ICC3	0.9029	19.59	34	34	2.56E-14	0.834	0.944	v7.1	HCP
RH_paracentral_surfavg	ICC3	0.9454	35.60	34	34	2.03E-18	0.905	0.969	v5.3	HCP
RH_paracentral_surfavg	ICC2	0.7983	8.71	34	34	3.74E-09	0.668	0.881	v5.3 vs. v6.0	HCP
RH_paracentral_surfavg	ICC2	0.8310	11.08	34	34	1.28E-10	0.720	0.901	v5.3 vs. v7.1	HCP
RH_paracentral_surfavg	ICC3	0.9520	40.69	34	34	2.33E-19	0.917	0.973	v6.0	HCP

RH_paracentral_surfavg	ICC2	0.9785	117.04	34	34	6.12E-27	0.951	0.989	v6.0 vs. v7.1	HCP
RH_paracentral_surfavg	ICC3	0.9833	118.90	34	34	4.70E-27	0.971	0.991	v7.1	HCP
RH_paracentral_thickavg	ICC3	0.9526	41.19	34	34	1.91E-19	0.918	0.973	v5.3	HCP
RH_paracentral_thickavg	ICC2	0.8791	18.60	34	34	5.70E-14	0.767	0.934	v5.3 vs. v6.0	HCP
RH_paracentral_thickavg	ICC2	0.9140	21.66	34	34	5.41E-15	0.852	0.951	v5.3 vs. v7.1	HCP
RH_paracentral_thickavg	ICC3	0.9453	35.58	34	34	2.04E-18	0.905	0.969	v6.0	HCP
RH_paracentral_thickavg	ICC2	0.9499	67.70	34	34	5.53E-23	0.798	0.980	v6.0 vs. v7.1	HCP
RH_paracentral_thickavg	ICC3	0.9463	36.21	34	34	1.54E-18	0.907	0.969	v7.1	HCP
RH_parahippocampal_surfavg	ICC3	0.8835	16.16	34	34	4.86E-13	0.802	0.933	v5.3	HCP
RH_parahippocampal_surfavg	ICC2	0.6421	7.25	34	34	4.40E-08	0.225	0.821	v5.3 vs. v6.0	HCP
RH_parahippocampal_surfavg	ICC2	0.5555	6.39	34	34	2.27E-07	0.059	0.781	v5.3 vs. v7.1	HCP
RH_parahippocampal_surfavg	ICC3	0.8859	16.53	34	34	3.45E-13	0.806	0.934	v6.0	HCP
RH_parahippocampal_surfavg	ICC2	0.8896	22.44	34	34	3.10E-15	0.755	0.944	v6.0 vs. v7.1	HCP
RH_parahippocampal_surfavg	ICC3	0.9614	50.87	34	34	6.11E-21	0.933	0.978	v7.1	HCP
RH_parahippocampal_thickavg	ICC3	0.9146	22.43	34	34	3.12E-15	0.854	0.951	v5.3	HCP
RH_parahippocampal_thickavg	ICC2	0.9363	30.73	34	34	2.15E-17	0.890	0.963	v5.3 vs. v6.0	HCP
RH_parahippocampal_thickavg	ICC2	0.7361	17.96	34	34	9.72E-14	0.060	0.900	v5.3 vs. v7.1	HCP
RH_parahippocampal_thickavg	ICC3	0.9377	31.11	34	34	1.76E-17	0.892	0.964	v6.0	HCP
RH_parahippocampal_thickavg	ICC2	0.8076	69.36	34	34	3.70E-23	0.000	0.941	v6.0 vs. v7.1	HCP
RH_parahippocampal_thickavg	ICC3	0.9484	37.75	34	34	7.85E-19	0.910	0.971	v7.1	HCP
RH_parsopercularis_surfavg	ICC3	0.9751	79.20	34	34	4.11E-24	0.956	0.986	v5.3	HCP
RH_parsopercularis_surfavg	ICC2	0.9239	29.29	34	34	4.61E-17	0.856	0.958	v5.3 vs. v6.0	HCP
RH_parsopercularis_surfavg	ICC2	0.9009	23.90	34	34	1.15E-15	0.795	0.948	v5.3 vs. v7.1	HCP
RH_parsopercularis_surfavg	ICC3	0.9907	214.78	34	34	2.28E-31	0.984	0.995	v6.0	HCP
RH_parsopercularis_surfavg	ICC2	0.9866	163.43	34	34	2.27E-29	0.975	0.993	v6.0 vs. v7.1	HCP
RH_parsopercularis_surfavg	ICC3	0.9760	82.21	34	34	2.21E-24	0.958	0.986	v7.1	HCP
RH_parsopercularis_thickavg	ICC3	0.9158	22.76	34	34	2.49E-15	0.856	0.952	v5.3	HCP
RH_parsopercularis_thickavg	ICC2	0.8930	26.15	34	34	2.80E-16	0.709	0.950	v5.3 vs. v6.0	HCP
RH_parsopercularis_thickavg	ICC2	0.9075	23.67	34	34	1.34E-15	0.828	0.949	v5.3 vs. v7.1	HCP
RH_parsopercularis_thickavg	ICC3	0.9187	23.59	34	34	1.42E-15	0.860	0.953	v6.0	HCP
RH_parsopercularis_thickavg	ICC2	0.8484	35.20	34	34	2.42E-18	0.186	0.947	v6.0 vs. v7.1	HCP
RH_parsopercularis_thickavg	ICC3	0.9448	35.26	34	34	2.36E-18	0.904	0.968	v7.1	HCP
RH_parsorbitalis_surfavg	ICC3	0.9536	42.14	34	34	1.32E-19	0.919	0.974	v5.3	HCP
RH_parsorbitalis_surfavg	ICC2	0.7299	20.24	34	34	1.54E-14	0.020	0.901	v5.3 vs. v6.0	HCP
RH_parsorbitalis_surfavg	ICC2	0.5988	17.83	34	34	1.09E-13	-0.044	0.845	v5.3 vs. v7.1	HCP
RH_parsorbitalis_surfavg	ICC3	0.9688	63.20	34	34	1.72E-22	0.945	0.982	v6.0	HCP
RH_parsorbitalis_surfavg	ICC2	0.9231	56.08	34	34	1.23E-21	0.556	0.972	v6.0 vs. v7.1	HCP
RH_parsorbitalis_surfavg	ICC3	0.9852	133.78	34	34	6.52E-28	0.974	0.992	v7.1	HCP
RH_parsorbitalis_thickavg	ICC3	0.8661	13.94	34	34	4.47E-12	0.774	0.922	v5.3	HCP
RH_parsorbitalis_thickavg	ICC2	0.8964	18.02	34	34	9.23E-14	0.824	0.940	v5.3 vs. v6.0	HCP
RH_parsorbitalis_thickavg	ICC2	0.6394	12.09	34	34	3.65E-11	-0.001	0.852	v5.3 vs. v7.1	HCP
RH_parsorbitalis_thickavg	ICC3	0.8607	13.36	34	34	8.39E-12	0.766	0.919	v6.0	HCP
RH_parsorbitalis_thickavg	ICC2	0.6764	17.31	34	34	1.72E-13	-0.016	0.877	v6.0 vs. v7.1	HCP
RH_parsorbitalis_thickavg	ICC3	0.9041	19.85	34	34	2.10E-14	0.836	0.945	v7.1	HCP

RH_parstriangularis_surfavg	ICC3	0.9583	46.93	34	34	2.29E-20	0.927	0.976	v5.3	HCP
RH_parstriangularis_surfavg	ICC2	0.8494	13.29	34	34	9.01E-12	0.742	0.913	v5.3 vs. v6.0	HCP
RH_parstriangularis_surfavg	ICC2	0.8181	12.40	34	34	2.50E-11	0.647	0.902	v5.3 vs. v7.1	HCP
RH_parstriangularis_surfavg	ICC3	0.9817	108.24	34	34	2.26E-26	0.968	0.990	v6.0	HCP
RH_parstriangularis_surfavg	ICC2	0.9836	195.71	34	34	1.09E-30	0.939	0.993	v6.0 vs. v7.1	HCP
RH_parstriangularis_surfavg	ICC3	0.9750	78.97	34	34	4.31E-24	0.956	0.986	v7.1	HCP
RH_parstriangularis_thickavg	ICC3	0.8875	16.77	34	34	2.77E-13	0.809	0.935	v5.3	HCP
RH_parstriangularis_thickavg	ICC2	0.9111	24.19	34	34	9.56E-16	0.838	0.950	v5.3 vs. v6.0	HCP
RH_parstriangularis_thickavg	ICC2	0.8421	22.61	34	34	2.75E-15	0.413	0.936	v5.3 vs. v7.1	HCP
RH_parstriangularis_thickavg	ICC3	0.8991	18.83	34	34	4.73E-14	0.828	0.942	v6.0	HCP
RH_parstriangularis_thickavg	ICC2	0.7928	35.00	34	34	2.66E-18	0.024	0.931	v6.0 vs. v7.1	HCP
RH_parstriangularis_thickavg	ICC3	0.9163	22.90	34	34	2.27E-15	0.856	0.952	v7.1	HCP
RH_pericalcarine_surfavg	ICC3	0.9797	97.74	34	34	1.24E-25	0.964	0.989	v5.3	HCP
RH_pericalcarine_surfavg	ICC2	0.7681	9.38	34	34	1.35E-09	0.569	0.872	v5.3 vs. v6.0	HCP
RH_pericalcarine_surfavg	ICC2	0.7426	8.47	34	34	5.51E-09	0.520	0.858	v5.3 vs. v7.1	HCP
RH_pericalcarine_surfavg	ICC3	0.9919	247.03	34	34	2.16E-32	0.986	0.995	v6.0	HCP
RH_pericalcarine_surfavg	ICC2	0.9837	125.12	34	34	2.00E-27	0.971	0.991	v6.0 vs. v7.1	HCP
RH_pericalcarine_surfavg	ICC3	0.9799	98.55	34	34	1.08E-25	0.965	0.989	v7.1	HCP
RH_pericalcarine_thickavg	ICC3	0.8837	16.19	34	34	4.72E-13	0.803	0.933	v5.3	HCP
RH_pericalcarine_thickavg	ICC2	0.8121	16.98	34	34	2.29E-13	0.416	0.919	v5.3 vs. v6.0	HCP
RH_pericalcarine_thickavg	ICC2	0.8307	10.58	34	34	2.47E-10	0.718	0.901	v5.3 vs. v7.1	HCP
RH_pericalcarine_thickavg	ICC3	0.8231	10.30	34	34	3.61E-10	0.706	0.896	v6.0	HCP
RH_pericalcarine_thickavg	ICC2	0.8021	16.58	34	34	3.29E-13	0.372	0.915	v6.0 vs. v7.1	HCP
RH_pericalcarine_thickavg	ICC3	0.9208	24.25	34	34	9.18E-16	0.864	0.955	v7.1	HCP
RH_postcentral_surfavg	ICC3	0.9880	165.33	34	34	1.87E-29	0.979	0.993	v5.3	HCP
RH_postcentral_surfavg	ICC2	0.8579	13.45	34	34	7.61E-12	0.762	0.917	v5.3 vs. v6.0	HCP
RH_postcentral_surfavg	ICC2	0.8630	13.35	34	34	8.45E-12	0.770	0.920	v5.3 vs. v7.1	HCP
RH_postcentral_surfavg	ICC3	0.9928	278.36	34	34	2.88E-33	0.987	0.996	v6.0	HCP
RH_postcentral_surfavg	ICC2	0.9897	288.44	34	34	1.58E-33	0.967	0.995	v6.0 vs. v7.1	HCP
RH_postcentral_surfavg	ICC3	0.9928	275.15	34	34	3.50E-33	0.987	0.996	v7.1	HCP
RH_postcentral_thickavg	ICC3	0.9385	31.51	34	34	1.44E-17	0.894	0.965	v5.3	HCP
RH_postcentral_thickavg	ICC2	0.8760	28.96	34	34	5.54E-17	0.507	0.950	v5.3 vs. v6.0	HCP
RH_postcentral_thickavg	ICC2	0.9363	29.57	34	34	3.98E-17	0.890	0.964	v5.3 vs. v7.1	HCP
RH_postcentral_thickavg	ICC3	0.9574	45.99	34	34	3.17E-20	0.926	0.976	v6.0	HCP
RH_postcentral_thickavg	ICC2	0.9041	68.15	34	34	4.96E-23	0.239	0.969	v6.0 vs. v7.1	HCP
RH_postcentral_thickavg	ICC3	0.9526	41.20	34	34	1.90E-19	0.918	0.973	v7.1	HCP
RH_posteriorcingulate_surfavg	ICC3	0.9722	70.95	34	34	2.55E-23	0.951	0.984	v5.3	HCP
RH_posteriorcingulate_surfavg	ICC2	0.8717	16.28	34	34	4.34E-13	0.773	0.927	v5.3 vs. v6.0	HCP
RH_posteriorcingulate_surfavg	ICC2	0.8880	17.28	34	34	1.77E-13	0.810	0.935	v5.3 vs. v7.1	HCP
RH_posteriorcingulate_surfavg	ICC3	0.9664	58.61	34	34	5.97E-22	0.941	0.981	v6.0	HCP
RH_posteriorcingulate_surfavg	ICC2	0.9802	115.54	34	34	7.60E-27	0.962	0.989	v6.0 vs. v7.1	HCP
RH_posteriorcingulate_surfavg	ICC3	0.9838	122.76	34	34	2.75E-27	0.972	0.991	v7.1	HCP
RH_posteriorcingulate_thickavg	ICC3	0.8564	12.92	34	34	1.37E-11	0.759	0.916	v5.3	HCP
RH_posteriorcingulate_thickavg	ICC2	0.8279	11.06	34	34	1.31E-10	0.714	0.899	v5.3 vs. v6.0	HCP

RH_posteriorcingulate_thickavg	ICC2	0.4367	13.21	34	34	9.94E-12	-0.046	0.747	v5.3 vs. v7.1	HCP
RH_posteriorcingulate_thickavg	ICC3	0.9028	19.58	34	34	2.59E-14	0.834	0.944	v6.0	HCP
RH_posteriorcingulate_thickavg	ICC2	0.5065	11.18	34	34	1.13E-10	-0.056	0.786	v6.0 vs. v7.1	HCP
RH_posteriorcingulate_thickavg	ICC3	0.8704	14.44	34	34	2.65E-12	0.781	0.925	v7.1	HCP
RH_precentral_surfavg	ICC3	0.9849	131.13	34	34	9.13E-28	0.973	0.991	v5.3	HCP
RH_precentral_surfavg	ICC2	0.9592	54.29	34	34	2.10E-21	0.924	0.978	v5.3 vs. v6.0	HCP
RH_precentral_surfavg	ICC2	0.9586	50.68	34	34	6.50E-21	0.926	0.977	v5.3 vs. v7.1	HCP
RH_precentral_surfavg	ICC3	0.9905	210.61	34	34	3.18E-31	0.983	0.995	v6.0	HCP
RH_precentral_surfavg	ICC2	0.9956	459.64	34	34	5.96E-37	0.992	0.998	v6.0 vs. v7.1	HCP
RH_precentral_surfavg	ICC3	0.9930	283.69	34	34	2.09E-33	0.988	0.996	v7.1	HCP
RH_precentral_thickavg	ICC3	0.9064	20.37	34	34	1.40E-14	0.840	0.946	v5.3	HCP
RH_precentral_thickavg	ICC2	0.8588	26.94	34	34	1.74E-16	0.415	0.945	v5.3 vs. v6.0	HCP
RH_precentral_thickavg	ICC2	0.9150	23.39	34	34	1.62E-15	0.854	0.951	v5.3 vs. v7.1	HCP
RH_precentral_thickavg	ICC3	0.9259	26.00	34	34	3.07E-16	0.872	0.958	v6.0	HCP
RH_precentral_thickavg	ICC2	0.8436	57.86	34	34	7.37E-22	0.041	0.951	v6.0 vs. v7.1	HCP
RH_precentral_thickavg	ICC3	0.9086	20.88	34	34	9.55E-15	0.844	0.947	v7.1	HCP
RH_precuneus_surfavg	ICC3	0.9837	121.76	34	34	3.16E-27	0.971	0.991	v5.3	HCP
RH_precuneus_surfavg	ICC2	0.7551	7.19	34	34	4.95E-08	0.605	0.854	v5.3 vs. v6.0	HCP
RH_precuneus_surfavg	ICC2	0.7448	6.72	34	34	1.19E-07	0.588	0.848	v5.3 vs. v7.1	HCP
RH_precuneus_surfavg	ICC3	0.9911	224.70	34	34	1.07E-31	0.984	0.995	v6.0	HCP
RH_precuneus_surfavg	ICC2	0.9941	499.94	34	34	1.44E-37	0.982	0.997	v6.0 vs. v7.1	HCP
RH_precuneus_surfavg	ICC3	0.9931	288.74	34	34	1.55E-33	0.988	0.996	v7.1	HCP
RH_precuneus_thickavg	ICC3	0.9371	30.82	34	34	2.05E-17	0.891	0.964	v5.3	HCP
RH_precuneus_thickavg	ICC2	0.8299	12.08	34	34	3.68E-11	0.703	0.903	v5.3 vs. v6.0	HCP
RH_precuneus_thickavg	ICC2	0.8054	11.78	34	34	5.31E-11	0.614	0.896	v5.3 vs. v7.1	HCP
RH_precuneus_thickavg	ICC3	0.9425	33.76	34	34	4.76E-18	0.900	0.967	v6.0	HCP
RH_precuneus_thickavg	ICC2	0.8068	50.25	34	34	7.46E-21	0.008	0.939	v6.0 vs. v7.1	HCP
RH_precuneus_thickavg	ICC3	0.9308	27.91	34	34	9.99E-17	0.881	0.960	v7.1	HCP
RH_Putamen	ICC3	0.9406	32.65	34	34	8.16E-18	0.897	0.966	v5.3	HCP
RH_Putamen	ICC2	0.7315	16.79	34	34	2.72E-13	0.071	0.896	v5.3 vs. v6.0	HCP
RH_Putamen	ICC2	0.8267	25.23	34	34	4.92E-16	0.241	0.935	v5.3 vs. v7.1	HCP
RH_Putamen	ICC3	0.9108	21.42	34	34	6.41E-15	0.847	0.949	v6.0	HCP
RH_Putamen	ICC2	0.9064	23.08	34	34	1.99E-15	0.829	0.948	v6.0 vs. v7.1	HCP
RH_Putamen	ICC3	0.9495	38.60	34	34	5.47E-19	0.912	0.971	v7.1	HCP
RH_rostralanteriorcingulate_surfavg	ICC3	0.9561	44.59	34	34	5.25E-20	0.924	0.975	v5.3	HCP
RH_rostralanteriorcingulate_surfavg	ICC2	0.7183	15.73	34	34	7.32E-13	0.062	0.890	v5.3 vs. v6.0	HCP
RH_rostralanteriorcingulate_surfavg	ICC2	0.7792	13.81	34	34	5.13E-12	0.373	0.902	v5.3 vs. v7.1	HCP
RH_rostralanteriorcingulate_surfavg	ICC3	0.9767	85.02	34	34	1.26E-24	0.959	0.987	v6.0	HCP
RH_rostralanteriorcingulate_surfavg	ICC2	0.9526	64.62	34	34	1.19E-22	0.843	0.979	v6.0 vs. v7.1	HCP
RH_rostralanteriorcingulate_surfavg	ICC3	0.9796	97.14	34	34	1.37E-25	0.964	0.988	v7.1	HCP
RH_rostralanteriorcingulate_thickavg	ICC3	0.7688	7.65	34	34	2.17E-08	0.624	0.863	v5.3	HCP
RH_rostralanteriorcingulate_thickavg	ICC2	0.5467	6.93	34	34	7.94E-08	0.014	0.784	v5.3 vs. v6.0	HCP
RH_rostralanteriorcingulate_thickavg	ICC2	0.6295	7.03	34	34	6.59E-08	0.202	0.815	v5.3 vs. v7.1	HCP
RH_rostralanteriorcingulate_thickavg	ICC3	0.7811	8.14	34	34	9.49E-09	0.642	0.870	v6.0	HCP

RH_rostralanteriorcingulate_thickavg	ICC2	0.3778	11.45	34	34	7.93E-11	-0.044	0.699	v6.0 vs. v7.1	HCP
RH_rostralanteriorcingulate_thickavg	ICC3	0.8384	11.37	34	34	8.79E-11	0.730	0.905	v7.1	HCP
RH_rostralmiddlefrontal_surfavg	ICC3	0.9931	290.17	34	34	1.43E-33	0.988	0.996	v5.3	HCP
RH_rostralmiddlefrontal_surfavg	ICC2	0.9664	59.17	34	34	5.10E-22	0.941	0.981	v5.3 vs. v6.0	HCP
RH_rostralmiddlefrontal_surfavg	ICC2	0.9612	55.00	34	34	1.70E-21	0.930	0.978	v5.3 vs. v7.1	HCP
RH_rostralmiddlefrontal_surfavg	ICC3	0.9956	456.33	34	34	6.74E-37	0.992	0.998	v6.0	HCP
RH_rostralmiddlefrontal_surfavg	ICC2	0.9861	503.66	34	34	1.27E-37	0.754	0.996	v6.0 vs. v7.1	HCP
RH_rostralmiddlefrontal_surfavg	ICC3	0.9927	271.19	34	34	4.47E-33	0.987	0.996	v7.1	HCP
RH_rostralmiddlefrontal_thickavg	ICC3	0.7709	7.73	34	34	1.90E-08	0.627	0.864	v5.3	HCP
RH_rostralmiddlefrontal_thickavg	ICC2	0.8318	13.53	34	34	6.93E-12	0.670	0.910	v5.3 vs. v6.0	HCP
RH_rostralmiddlefrontal_thickavg	ICC2	0.6573	10.09	34	34	4.88E-10	0.088	0.850	v5.3 vs. v7.1	HCP
RH_rostralmiddlefrontal_thickavg	ICC3	0.7616	7.39	34	34	3.45E-08	0.613	0.858	v6.0	HCP
RH_rostralmiddlefrontal_thickavg	ICC2	0.6036	23.81	34	34	1.23E-15	-0.038	0.852	v6.0 vs. v7.1	HCP
RH_rostralmiddlefrontal_thickavg	ICC3	0.8260	10.49	34	34	2.78E-10	0.711	0.898	v7.1	HCP
RH_superiorfrontal_surfavg	ICC3	0.9931	287.18	34	34	1.70E-33	0.988	0.996	v5.3	HCP
RH_superiorfrontal_surfavg	ICC2	0.9609	62.49	34	34	2.07E-22	0.915	0.980	v5.3 vs. v6.0	HCP
RH_superiorfrontal_surfavg	ICC2	0.9424	44.21	34	34	6.05E-20	0.866	0.971	v5.3 vs. v7.1	HCP
RH_superiorfrontal_surfavg	ICC3	0.9962	522.22	34	34	6.87E-38	0.993	0.998	v6.0	HCP
RH_superiorfrontal_surfavg	ICC2	0.9958	602.29	34	34	6.13E-39	0.991	0.998	v6.0 vs. v7.1	HCP
RH_superiorfrontal_surfavg	ICC3	0.9959	491.03	34	34	1.95E-37	0.993	0.998	v7.1	HCP
RH_superiorfrontal_thickavg	ICC3	0.9206	24.18	34	34	9.62E-16	0.863	0.954	v5.3	HCP
RH_superiorfrontal_thickavg	ICC2	0.9610	58.59	34	34	6.00E-22	0.924	0.979	v5.3 vs. v6.0	HCP
RH_superiorfrontal_thickavg	ICC2	0.8336	30.76	34	34	2.12E-17	0.175	0.941	v5.3 vs. v7.1	HCP
RH_superiorfrontal_thickavg	ICC3	0.9203	24.10	34	34	1.02E-15	0.863	0.954	v6.0	HCP
RH_superiorfrontal_thickavg	ICC2	0.8951	51.19	34	34	5.51E-21	0.309	0.964	v6.0 vs. v7.1	HCP
RH_superiorfrontal_thickavg	ICC3	0.9315	28.18	34	34	8.57E-17	0.882	0.961	v7.1	HCP
RH_superiorparietal_surfavg	ICC3	0.9914	232.03	34	34	6.21E-32	0.985	0.995	v5.3	HCP
RH_superiorparietal_surfavg	ICC2	0.9045	24.51	34	34	7.76E-16	0.806	0.950	v5.3 vs. v6.0	HCP
RH_superiorparietal_surfavg	ICC2	0.9049	22.80	34	34	2.42E-15	0.825	0.947	v5.3 vs. v7.1	HCP
RH_superiorparietal_surfavg	ICC3	0.9951	408.38	34	34	4.41E-36	0.991	0.997	v6.0	HCP
RH_superiorparietal_surfavg	ICC2	0.9887	178.49	34	34	5.15E-30	0.980	0.994	v6.0 vs. v7.1	HCP
RH_superiorparietal_surfavg	ICC3	0.9917	238.81	34	34	3.82E-32	0.985	0.995	v7.1	HCP
RH_superiorparietal_thickavg	ICC3	0.9561	44.61	34	34	5.22E-20	0.924	0.975	v5.3	HCP
RH_superiorparietal_thickavg	ICC2	0.9105	43.59	34	34	7.62E-20	0.569	0.966	v5.3 vs. v6.0	HCP
RH_superiorparietal_thickavg	ICC2	0.9389	36.55	34	34	1.32E-18	0.884	0.967	v5.3 vs. v7.1	HCP
RH_superiorparietal_thickavg	ICC3	0.9636	53.91	34	34	2.36E-21	0.936	0.979	v6.0	HCP
RH_superiorparietal_thickavg	ICC2	0.8730	59.11	34	34	5.19E-22	0.115	0.960	v6.0 vs. v7.1	HCP
RH_superiorparietal_thickavg	ICC3	0.9314	28.15	34	34	8.69E-17	0.882	0.961	v7.1	HCP
RH_superiortemporal_surfavg	ICC3	0.9845	127.71	34	34	1.42E-27	0.973	0.991	v5.3	HCP
RH_superiortemporal_surfavg	ICC2	0.9348	62.72	34	34	1.95E-22	0.641	0.976	v5.3 vs. v6.0	HCP
RH_superiortemporal_surfavg	ICC2	0.8985	62.12	34	34	2.29E-22	0.238	0.967	v5.3 vs. v7.1	HCP
RH_superiortemporal_surfavg	ICC3	0.9879	163.62	34	34	2.22E-29	0.979	0.993	v6.0	HCP
RH_superiortemporal_surfavg	ICC2	0.9843	264.18	34	34	6.95E-33	0.896	0.994	v6.0 vs. v7.1	HCP
RH_superiortemporal_surfavg	ICC3	0.9915	234.93	34	34	5.03E-32	0.985	0.995	v7.1	HCP

RH_superiortemporal_thickavg	ICC3	0.9116	21.62	34	34	5.54E-15	0.849	0.949	v5.3	HCP
RH_superiortemporal_thickavg	ICC2	0.9237	25.51	34	34	4.14E-16	0.869	0.956	v5.3 vs. v6.0	HCP
RH_superiortemporal_thickavg	ICC2	0.7772	20.71	34	34	1.08E-14	0.123	0.916	v5.3 vs. v7.1	HCP
RH_superiortemporal_thickavg	ICC3	0.9466	36.45	34	34	1.38E-18	0.907	0.970	v6.0	HCP
RH_superiortemporal_thickavg	ICC2	0.7743	56.57	34	34	1.07E-21	-0.010	0.929	v6.0 vs. v7.1	HCP
RH_superiortemporal_thickavg	ICC3	0.9126	21.88	34	34	4.62E-15	0.850	0.950	v7.1	HCP
RH_supramarginal_surfavg	ICC3	0.9877	161.51	34	34	2.76E-29	0.978	0.993	v5.3	HCP
RH_supramarginal_surfavg	ICC2	0.8200	10.30	34	34	3.65E-10	0.703	0.894	v5.3 vs. v6.0	HCP
RH_supramarginal_surfavg	ICC2	0.8300	10.66	34	34	2.23E-10	0.718	0.900	v5.3 vs. v7.1	HCP
RH_supramarginal_surfavg	ICC3	0.9917	239.06	34	34	3.75E-32	0.985	0.995	v6.0	HCP
RH_supramarginal_surfavg	ICC2	0.9937	433.58	34	34	1.60E-36	0.983	0.997	v6.0 vs. v7.1	HCP
RH_supramarginal_surfavg	ICC3	0.9964	559.91	34	34	2.11E-38	0.994	0.998	v7.1	HCP
RH_supramarginal_thickavg	ICC3	0.8780	15.39	34	34	1.02E-12	0.794	0.929	v5.3	HCP
RH_supramarginal_thickavg	ICC2	0.9275	26.55	34	34	2.20E-16	0.876	0.958	v5.3 vs. v6.0	HCP
RH_supramarginal_thickavg	ICC2	0.7870	20.37	34	34	1.41E-14	0.167	0.919	v5.3 vs. v7.1	HCP
RH_supramarginal_thickavg	ICC3	0.8987	18.74	34	34	5.09E-14	0.827	0.942	v6.0	HCP
RH_supramarginal_thickavg	ICC2	0.8321	40.62	34	34	2.40E-19	0.076	0.945	v6.0 vs. v7.1	HCP
RH_supramarginal_thickavg	ICC3	0.9239	25.27	34	34	4.81E-16	0.869	0.956	v7.1	HCP
RH_SurfArea	ICC3	0.9976	829.55	34	34	2.69E-41	0.996	0.999	v5.3	HCP
RH_SurfArea	ICC2	0.9980	2091.51	34	34	4.10E-48	0.985	0.999	v5.3 vs. v6.0	HCP
RH_SurfArea	ICC2	0.9848	1563.47	34	34	5.74E-46	0.351	0.996	v5.3 vs. v7.1	HCP
RH_SurfArea	ICC3	0.9973	730.77	34	34	2.31E-40	0.995	0.998	v6.0	HCP
RH_SurfArea	ICC2	0.9922	4077.54	34	34	4.86E-53	0.478	0.998	v6.0 vs. v7.1	HCP
RH_SurfArea	ICC3	0.9983	1163.06	34	34	8.71E-44	0.997	0.999	v7.1	HCP
RH_temporalpole_surfavg	ICC3	0.7496	6.99	34	34	7.16E-08	0.595	0.851	v5.3	HCP
RH_temporalpole_surfavg	ICC2	0.5611	6.90	34	34	8.49E-08	0.039	0.790	v5.3 vs. v6.0	HCP
RH_temporalpole_surfavg	ICC2	0.4094	5.36	34	34	1.97E-06	-0.057	0.694	v5.3 vs. v7.1	HCP
RH_temporalpole_surfavg	ICC3	0.8030	9.15	34	34	1.90E-09	0.676	0.884	v6.0	HCP
RH_temporalpole_surfavg	ICC2	0.7795	14.17	34	34	3.51E-12	0.356	0.903	v6.0 vs. v7.1	HCP
RH_temporalpole_surfavg	ICC3	0.8875	16.77	34	34	2.77E-13	0.809	0.935	v7.1	HCP
RH_temporalpole_thickavg	ICC3	0.7141	5.99	34	34	5.03E-07	0.544	0.828	v5.3	HCP
RH_temporalpole_thickavg	ICC2	0.8354	17.37	34	34	1.62E-13	0.549	0.924	v5.3 vs. v6.0	HCP
RH_temporalpole_thickavg	ICC2	0.5923	13.12	34	34	1.09E-11	-0.043	0.835	v5.3 vs. v7.1	HCP
RH_temporalpole_thickavg	ICC3	0.8524	12.55	34	34	2.11E-11	0.753	0.914	v6.0	HCP
RH_temporalpole_thickavg	ICC2	0.7798	17.95	34	34	9.82E-14	0.203	0.913	v6.0 vs. v7.1	HCP
RH_temporalpole_thickavg	ICC3	0.7920	8.62	34	34	4.37E-09	0.659	0.877	v7.1	HCP
RH_Thalamus	ICC3	0.8562	12.91	34	34	1.39E-11	0.759	0.916	v5.3	HCP
RH_Thalamus	ICC2	0.8925	17.17	34	34	1.94E-13	0.817	0.938	v5.3 vs. v6.0	HCP
RH_Thalamus	ICC2	0.8791	15.14	34	34	1.30E-12	0.795	0.930	v5.3 vs. v7.1	HCP
RH_Thalamus	ICC3	0.8301	10.77	34	34	1.92E-10	0.717	0.900	v6.0	HCP
RH_Thalamus	ICC2	0.8570	12.65	34	34	1.88E-11	0.759	0.917	v6.0 vs. v7.1	HCP
RH_Thalamus	ICC3	0.9021	19.43	34	34	2.91E-14	0.833	0.944	v7.1	HCP
RH_Thickness	ICC3	0.9401	32.39	34	34	9.24E-18	0.896	0.966	v5.3	HCP
RH_Thickness	ICC2	0.9507	50.16	34	34	7.69E-21	0.891	0.975	v5.3 vs. v6.0	HCP

RH_Thickness	ICC2	0.7617	35.18	34	34	2.46E-18	-0.007	0.920	v5.3 vs. v7.1	HCP
RH_Thickness	ICC3	0.9459	35.97	34	34	1.71E-18	0.906	0.969	v6.0	HCP
RH_Thickness	ICC2	0.6961	67.81	34	34	5.38E-23	-0.014	0.900	v6.0 vs. v7.1	HCP
RH_Thickness	ICC3	0.9401	32.39	34	34	9.27E-18	0.896	0.966	v7.1	HCP
RH_transversetemporal_surfavg	ICC3	0.9347	29.61	34	34	3.89E-17	0.887	0.963	v5.3	HCP
RH_transversetemporal_surfavg	ICC2	0.7902	10.14	34	34	4.55E-10	0.620	0.883	v5.3 vs. v6.0	HCP
RH_transversetemporal_surfavg	ICC2	0.8446	12.37	34	34	2.60E-11	0.740	0.909	v5.3 vs. v7.1	HCP
RH_transversetemporal_surfavg	ICC3	0.9454	35.66	34	34	1.97E-18	0.905	0.969	v6.0	HCP
RH_transversetemporal_surfavg	ICC2	0.8976	20.79	34	34	1.02E-14	0.815	0.943	v6.0 vs. v7.1	HCP
RH_transversetemporal_surfavg	ICC3	0.8971	18.44	34	34	6.50E-14	0.825	0.941	v7.1	HCP
RH_transversetemporal_thickavg	ICC3	0.7185	6.10	34	34	4.02E-07	0.550	0.831	v5.3	HCP
RH_transversetemporal_thickavg	ICC2	0.8057	9.73	34	34	8.07E-10	0.679	0.886	v5.3 vs. v6.0	HCP
RH_transversetemporal_thickavg	ICC2	0.6760	5.30	34	34	2.26E-06	0.493	0.803	v5.3 vs. v7.1	HCP
RH_transversetemporal_thickavg	ICC3	0.8909	17.33	34	34	1.68E-13	0.814	0.937	v6.0	HCP
RH_transversetemporal_thickavg	ICC2	0.8558	25.12	34	34	5.29E-16	0.441	0.942	v6.0 vs. v7.1	HCP
RH_transversetemporal_thickavg	ICC3	0.9005	19.11	34	34	3.77E-14	0.830	0.943	v7.1	HCP
SUM_bankssts_surfavg	ICC3	0.9787	92.97	34	34	2.86E-25	0.963	0.988	v5.3	HCP
SUM_bankssts_surfavg	ICC2	0.7835	11.84	34	34	4.93E-11	0.503	0.893	v5.3 vs. v6.0	HCP
SUM_bankssts_surfavg	ICC2	0.7791	10.12	34	34	4.68E-10	0.576	0.880	v5.3 vs. v7.1	HCP
SUM_bankssts_surfavg	ICC3	0.9786	92.67	34	34	3.01E-25	0.962	0.988	v6.0	HCP
SUM_bankssts_surfavg	ICC2	0.9760	85.42	34	34	1.17E-24	0.958	0.986	v6.0 vs. v7.1	HCP
SUM_bankssts_surfavg	ICC3	0.9816	107.64	34	34	2.48E-26	0.968	0.990	v7.1	HCP
SUM_caudalanteriorcingulate_surfavg	ICC3	0.9729	72.82	34	34	1.65E-23	0.952	0.985	v5.3	HCP
SUM_caudalanteriorcingulate_surfavg	ICC2	0.8363	34.93	34	34	2.75E-18	0.134	0.944	v5.3 vs. v6.0	HCP
SUM_caudalanteriorcingulate_surfavg	ICC2	0.9148	27.56	34	34	1.22E-16	0.826	0.955	v5.3 vs. v7.1	HCP
SUM_caudalanteriorcingulate_surfavg	ICC3	0.9909	218.17	34	34	1.75E-31	0.984	0.995	v6.0	HCP
SUM_caudalanteriorcingulate_surfavg	ICC2	0.9312	75.87	34	34	8.38E-24	0.472	0.977	v6.0 vs. v7.1	HCP
SUM_caudalanteriorcingulate_surfavg	ICC3	0.9886	173.78	34	34	8.07E-30	0.980	0.994	v7.1	HCP
SUM_caudalmiddlefrontal_surfavg	ICC3	0.9933	298.51	34	34	8.83E-34	0.988	0.996	v5.3	HCP
SUM_caudalmiddlefrontal_surfavg	ICC2	0.9801	97.70	34	34	1.25E-25	0.965	0.989	v5.3 vs. v6.0	HCP
SUM_caudalmiddlefrontal_surfavg	ICC2	0.9729	70.79	34	34	2.64E-23	0.952	0.985	v5.3 vs. v7.1	HCP
SUM_caudalmiddlefrontal_surfavg	ICC3	0.9938	322.56	34	34	2.38E-34	0.989	0.997	v6.0	HCP
SUM_caudalmiddlefrontal_surfavg	ICC2	0.9947	391.27	34	34	9.11E-36	0.991	0.997	v6.0 vs. v7.1	HCP
SUM_caudalmiddlefrontal_surfavg	ICC3	0.9950	399.52	34	34	6.40E-36	0.991	0.997	v7.1	HCP
SUM_cuneus_surfavg	ICC3	0.9562	44.63	34	34	5.18E-20	0.924	0.975	v5.3	HCP
SUM_cuneus_surfavg	ICC2	0.6562	7.13	34	34	5.55E-08	0.287	0.823	v5.3 vs. v6.0	HCP
SUM_cuneus_surfavg	ICC2	0.6334	8.27	34	34	7.62E-09	0.120	0.829	v5.3 vs. v7.1	HCP
SUM_cuneus_surfavg	ICC3	0.9889	178.54	34	34	5.12E-30	0.980	0.994	v6.0	HCP
SUM_cuneus_surfavg	ICC2	0.9824	202.73	34	34	6.03E-31	0.918	0.993	v6.0 vs. v7.1	HCP
SUM_cuneus_surfavg	ICC3	0.9874	158.20	34	34	3.91E-29	0.978	0.993	v7.1	HCP
SUM_entorhinal_surfavg	ICC3	0.9222	24.72	34	34	6.81E-16	0.866	0.955	v5.3	HCP
SUM_entorhinal_surfavg	ICC2	0.8273	14.89	34	34	1.67E-12	0.597	0.915	v5.3 vs. v6.0	HCP
SUM_entorhinal_surfavg	ICC2	0.8145	10.35	34	34	3.39E-10	0.692	0.891	v5.3 vs. v7.1	HCP
SUM_entorhinal_surfavg	ICC3	0.9453	35.58	34	34	2.04E-18	0.905	0.969	v6.0	HCP

SUM_entorhinal_surfavg	ICC2	0.9159	28.81	34	34	6.00E-17	0.820	0.957	v6.0 vs. v7.1	HCP
SUM_entorhinal_surfavg	ICC3	0.9154	22.65	34	34	2.68E-15	0.855	0.951	v7.1	HCP
SUM_frontalpole_surfavg	ICC3	0.8103	9.54	34	34	1.06E-09	0.687	0.888	v5.3	HCP
SUM_frontalpole_surfavg	ICC2	0.3331	10.41	34	34	3.12E-10	-0.042	0.659	v5.3 vs. v6.0	HCP
SUM_frontalpole_surfavg	ICC2	0.1930	7.71	34	34	1.95E-08	-0.030	0.487	v5.3 vs. v7.1	HCP
SUM_frontalpole_surfavg	ICC3	0.8582	13.10	34	34	1.12E-11	0.762	0.917	v6.0	HCP
SUM_frontalpole_surfavg	ICC2	0.7072	16.36	34	34	4.03E-13	0.029	0.887	v6.0 vs. v7.1	HCP
SUM_frontalpole_surfavg	ICC3	0.8884	16.93	34	34	2.41E-13	0.810	0.935	v7.1	HCP
SUM_fusiform_surfavg	ICC3	0.9791	94.63	34	34	2.13E-25	0.963	0.988	v5.3	HCP
SUM_fusiform_surfavg	ICC2	0.8752	29.04	34	34	5.31E-17	0.498	0.950	v5.3 vs. v6.0	HCP
SUM_fusiform_surfavg	ICC2	0.8992	28.29	34	34	8.04E-17	0.716	0.954	v5.3 vs. v7.1	HCP
SUM_fusiform_surfavg	ICC3	0.9930	286.72	34	34	1.74E-33	0.988	0.996	v6.0	HCP
SUM_fusiform_surfavg	ICC2	0.9898	366.77	34	34	2.72E-35	0.946	0.996	v6.0 vs. v7.1	HCP
SUM_fusiform_surfavg	ICC3	0.9956	455.91	34	34	6.85E-37	0.992	0.998	v7.1	HCP
SUM_inferiorparietal_surfavg	ICC3	0.9943	351.73	34	34	5.52E-35	0.990	0.997	v5.3	HCP
SUM_inferiorparietal_surfavg	ICC2	0.9077	35.02	34	34	2.64E-18	0.675	0.961	v5.3 vs. v6.0	HCP
SUM_inferiorparietal_surfavg	ICC2	0.9219	31.95	34	34	1.15E-17	0.825	0.961	v5.3 vs. v7.1	HCP
SUM_inferiorparietal_surfavg	ICC3	0.9957	463.40	34	34	5.20E-37	0.992	0.998	v6.0	HCP
SUM_inferiorparietal_surfavg	ICC2	0.9897	264.65	34	34	6.75E-33	0.972	0.995	v6.0 vs. v7.1	HCP
SUM_inferiorparietal_surfavg	ICC3	0.9976	839.31	34	34	2.21E-41	0.996	0.999	v7.1	HCP
SUM_inferiortemporal_surfavg	ICC3	0.9919	247.30	34	34	2.12E-32	0.986	0.995	v5.3	HCP
SUM_inferiortemporal_surfavg	ICC2	0.9396	56.85	34	34	9.86E-22	0.753	0.976	v5.3 vs. v6.0	HCP
SUM_inferiortemporal_surfavg	ICC2	0.9182	69.91	34	34	3.25E-23	0.359	0.973	v5.3 vs. v7.1	HCP
SUM_inferiortemporal_surfavg	ICC3	0.9908	216.84	34	34	1.94E-31	0.984	0.995	v6.0	HCP
SUM_inferiortemporal_surfavg	ICC2	0.9876	257.37	34	34	1.08E-32	0.954	0.995	v6.0 vs. v7.1	HCP
SUM_inferiortemporal_surfavg	ICC3	0.9928	277.15	34	34	3.10E-33	0.987	0.996	v7.1	HCP
SUM_insula_surfavg	ICC3	0.8639	13.70	34	34	5.80E-12	0.771	0.921	v5.3	HCP
SUM_insula_surfavg	ICC2	0.8360	14.41	34	34	2.73E-12	0.661	0.914	v5.3 vs. v6.0	HCP
SUM_insula_surfavg	ICC2	0.9109	23.14	34	34	1.92E-15	0.844	0.949	v5.3 vs. v7.1	HCP
SUM_insula_surfavg	ICC3	0.8625	13.54	34	34	6.87E-12	0.769	0.920	v6.0	HCP
SUM_insula_surfavg	ICC2	0.8974	20.29	34	34	1.49E-14	0.819	0.942	v6.0 vs. v7.1	HCP
SUM_insula_surfavg	ICC3	0.9277	26.68	34	34	2.04E-16	0.875	0.959	v7.1	HCP
SUM_isthmuscingulate_surfavg	ICC3	0.9245	25.49	34	34	4.20E-16	0.870	0.957	v5.3	HCP
SUM_isthmuscingulate_surfavg	ICC2	0.9002	19.22	34	34	3.44E-14	0.831	0.942	v5.3 vs. v6.0	HCP
SUM_isthmuscingulate_surfavg	ICC2	0.8622	13.19	34	34	1.02E-11	0.768	0.920	v5.3 vs. v7.1	HCP
SUM_isthmuscingulate_surfavg	ICC3	0.9863	144.50	34	34	1.79E-28	0.976	0.992	v6.0	HCP
SUM_isthmuscingulate_surfavg	ICC2	0.9791	101.37	34	34	6.75E-26	0.963	0.988	v6.0 vs. v7.1	HCP
SUM_isthmuscingulate_surfavg	ICC3	0.9898	194.68	34	34	1.19E-30	0.982	0.994	v7.1	HCP
SUM_lateraloccipital_surfavg	ICC3	0.9806	101.94	34	34	6.15E-26	0.966	0.989	v5.3	HCP
SUM_lateraloccipital_surfavg	ICC2	0.7494	13.73	34	34	5.60E-12	0.229	0.894	v5.3 vs. v6.0	HCP
SUM_lateraloccipital_surfavg	ICC2	0.7243	14.50	34	34	2.48E-12	0.109	0.889	v5.3 vs. v7.1	HCP
SUM_lateraloccipital_surfavg	ICC3	0.9949	393.02	34	34	8.44E-36	0.991	0.997	v6.0	HCP
SUM_lateraloccipital_surfavg	ICC2	0.9873	185.41	34	34	2.72E-30	0.974	0.993	v6.0 vs. v7.1	HCP
SUM_lateraloccipital_surfavg	ICC3	0.9940	333.24	34	34	1.37E-34	0.989	0.997	v7.1	HCP

SUM_lateralorbitofrontal_surfavg	ICC3	0.9539	42.43	34	34	1.18E-19	0.920	0.974	v5.3	HCP
SUM_lateralorbitofrontal_surfavg	ICC2	0.9531	43.64	34	34	7.46E-20	0.918	0.973	v5.3 vs. v6.0	HCP
SUM_lateralorbitofrontal_surfavg	ICC2	0.8974	37.53	34	34	8.64E-19	0.530	0.960	v5.3 vs. v7.1	HCP
SUM_lateralorbitofrontal_surfavg	ICC3	0.9347	29.63	34	34	3.86E-17	0.887	0.963	v6.0	HCP
SUM_lateralorbitofrontal_surfavg	ICC2	0.9476	85.25	34	34	1.21E-24	0.652	0.981	v6.0 vs. v7.1	HCP
SUM_lateralorbitofrontal_surfavg	ICC3	0.9566	45.06	34	34	4.43E-20	0.924	0.975	v7.1	HCP
SUM_lingual_surfavg	ICC3	0.9860	142.28	34	34	2.32E-28	0.975	0.992	v5.3	HCP
SUM_lingual_surfavg	ICC2	0.8860	19.20	34	34	3.51E-14	0.788	0.937	v5.3 vs. v6.0	HCP
SUM_lingual_surfavg	ICC2	0.8564	22.43	34	34	3.13E-15	0.525	0.939	v5.3 vs. v7.1	HCP
SUM_lingual_surfavg	ICC3	0.9915	233.29	34	34	5.67E-32	0.985	0.995	v6.0	HCP
SUM_lingual_surfavg	ICC2	0.9804	393.52	34	34	8.26E-36	0.634	0.994	v6.0 vs. v7.1	HCP
SUM_lingual_surfavg	ICC3	0.9935	307.25	34	34	5.42E-34	0.989	0.996	v7.1	HCP
SUM_medialorbitofrontal_surfavg	ICC3	0.9555	43.93	34	34	6.71E-20	0.922	0.975	v5.3	HCP
SUM_medialorbitofrontal_surfavg	ICC2	0.8776	19.73	34	34	2.29E-14	0.739	0.937	v5.3 vs. v6.0	HCP
SUM_medialorbitofrontal_surfavg	ICC2	0.6450	18.52	34	34	6.10E-14	-0.036	0.866	v5.3 vs. v7.1	HCP
SUM_medialorbitofrontal_surfavg	ICC3	0.9184	23.51	34	34	1.50E-15	0.860	0.953	v6.0	HCP
SUM_medialorbitofrontal_surfavg	ICC2	0.7773	24.64	34	34	7.17E-16	0.064	0.920	v6.0 vs. v7.1	HCP
SUM_medialorbitofrontal_surfavg	ICC3	0.9543	42.78	34	34	1.03E-19	0.920	0.974	v7.1	HCP
SUM_middletemporal_surfavg	ICC3	0.9948	380.28	34	34	1.47E-35	0.991	0.997	v5.3	HCP
SUM_middletemporal_surfavg	ICC2	0.9429	98.14	34	34	1.16E-25	0.492	0.981	v5.3 vs. v6.0	HCP
SUM_middletemporal_surfavg	ICC2	0.8944	80.25	34	34	3.30E-24	0.125	0.968	v5.3 vs. v7.1	HCP
SUM_middletemporal_surfavg	ICC3	0.9938	319.54	34	34	2.80E-34	0.989	0.996	v6.0	HCP
SUM_middletemporal_surfavg	ICC2	0.9841	362.92	34	34	3.25E-35	0.799	0.995	v6.0 vs. v7.1	HCP
SUM_middletemporal_surfavg	ICC3	0.9970	655.76	34	34	1.45E-39	0.995	0.998	v7.1	HCP
SUM_paracentral_surfavg	ICC3	0.9690	63.45	34	34	1.61E-22	0.946	0.982	v5.3	HCP
SUM_paracentral_surfavg	ICC2	0.8881	16.64	34	34	3.12E-13	0.810	0.935	v5.3 vs. v6.0	HCP
SUM_paracentral_surfavg	ICC2	0.9029	19.15	34	34	3.63E-14	0.834	0.944	v5.3 vs. v7.1	HCP
SUM_paracentral_surfavg	ICC3	0.9734	74.15	34	34	1.22E-23	0.953	0.985	v6.0	HCP
SUM_paracentral_surfavg	ICC2	0.9881	261.84	34	34	8.08E-33	0.958	0.995	v6.0 vs. v7.1	HCP
SUM_paracentral_surfavg	ICC3	0.9865	147.41	34	34	1.28E-28	0.976	0.992	v7.1	HCP
SUM_parahippocampal_surfavg	ICC3	0.8775	15.33	34	34	1.08E-12	0.793	0.929	v5.3	HCP
SUM_parahippocampal_surfavg	ICC2	0.7444	9.75	34	34	7.90E-10	0.441	0.871	v5.3 vs. v6.0	HCP
SUM_parahippocampal_surfavg	ICC2	0.6832	9.93	34	34	6.08E-10	0.171	0.857	v5.3 vs. v7.1	HCP
SUM_parahippocampal_surfavg	ICC3	0.9525	41.13	34	34	1.96E-19	0.917	0.973	v6.0	HCP
SUM_parahippocampal_surfavg	ICC2	0.9404	51.17	34	34	5.55E-21	0.806	0.974	v6.0 vs. v7.1	HCP
SUM_parahippocampal_surfavg	ICC3	0.9561	44.55	34	34	5.33E-20	0.923	0.975	v7.1	HCP
SUM_parsopercularis_surfavg	ICC3	0.9814	106.72	34	34	2.86E-26	0.967	0.989	v5.3	HCP
SUM_parsopercularis_surfavg	ICC2	0.9200	44.10	34	34	6.30E-20	0.667	0.968	v5.3 vs. v6.0	HCP
SUM_parsopercularis_surfavg	ICC2	0.8380	12.75	34	34	1.67E-11	0.716	0.908	v5.3 vs. v7.1	HCP
SUM_parsopercularis_surfavg	ICC3	0.9952	412.94	34	34	3.66E-36	0.991	0.997	v6.0	HCP
SUM_parsopercularis_surfavg	ICC2	0.8905	17.02	34	34	2.22E-13	0.814	0.937	v6.0 vs. v7.1	HCP
SUM_parsopercularis_surfavg	ICC3	0.9909	218.28	34	34	1.74E-31	0.984	0.995	v7.1	HCP
SUM_parsorbitalis_surfavg	ICC3	0.9715	69.10	34	34	3.94E-23	0.950	0.984	v5.3	HCP
SUM_parsorbitalis_surfavg	ICC2	0.7168	37.96	34	34	7.19E-19	-0.023	0.905	v5.3 vs. v6.0	HCP

SUM_parsorbitalis_surfavg	ICC2	0.5967	32.49	34	34	8.80E-18	-0.028	0.852	v5.3 vs. v7.1	HCP
SUM_parsorbitalis_surfavg	ICC3	0.9816	107.72	34	34	2.45E-26	0.968	0.990	v6.0	HCP
SUM_parsorbitalis_surfavg	ICC2	0.9455	106.29	34	34	3.06E-26	0.487	0.982	v6.0 vs. v7.1	HCP
SUM_parsorbitalis_surfavg	ICC3	0.9826	113.87	34	34	9.69E-27	0.969	0.990	v7.1	HCP
SUM_parstriangularis_surfavg	ICC3	0.9695	64.55	34	34	1.21E-22	0.947	0.983	v5.3	HCP
SUM_parstriangularis_surfavg	ICC2	0.8738	19.93	34	34	1.97E-14	0.713	0.937	v5.3 vs. v6.0	HCP
SUM_parstriangularis_surfavg	ICC2	0.7967	13.43	34	34	7.72E-12	0.489	0.904	v5.3 vs. v7.1	HCP
SUM_parstriangularis_surfavg	ICC3	0.9863	145.05	34	34	1.68E-28	0.976	0.992	v6.0	HCP
SUM_parstriangularis_surfavg	ICC2	0.9746	113.61	34	34	1.01E-26	0.925	0.988	v6.0 vs. v7.1	HCP
SUM_parstriangularis_surfavg	ICC3	0.9898	196.02	34	34	1.06E-30	0.982	0.994	v7.1	HCP
SUM_pericalcarine_surfavg	ICC3	0.9732	73.49	34	34	1.42E-23	0.953	0.985	v5.3	HCP
SUM_pericalcarine_surfavg	ICC2	0.7895	9.95	34	34	5.88E-10	0.625	0.881	v5.3 vs. v6.0	HCP
SUM_pericalcarine_surfavg	ICC2	0.7934	10.88	34	34	1.66E-10	0.601	0.888	v5.3 vs. v7.1	HCP
SUM_pericalcarine_surfavg	ICC3	0.9912	227.32	34	34	8.77E-32	0.985	0.995	v6.0	HCP
SUM_pericalcarine_surfavg	ICC2	0.9900	219.21	34	34	1.62E-31	0.982	0.994	v6.0 vs. v7.1	HCP
SUM_pericalcarine_surfavg	ICC3	0.9905	209.43	34	34	3.49E-31	0.983	0.995	v7.1	HCP
SUM_postcentral_surfavg	ICC3	0.9929	280.01	34	34	2.60E-33	0.987	0.996	v5.3	HCP
SUM_postcentral_surfavg	ICC2	0.9441	39.63	34	34	3.57E-19	0.895	0.969	v5.3 vs. v6.0	HCP
SUM_postcentral_surfavg	ICC2	0.8566	12.93	34	34	1.35E-11	0.760	0.916	v5.3 vs. v7.1	HCP
SUM_postcentral_surfavg	ICC3	0.9961	513.48	34	34	9.14E-38	0.993	0.998	v6.0	HCP
SUM_postcentral_surfavg	ICC2	0.9648	55.54	34	34	1.45E-21	0.939	0.980	v6.0 vs. v7.1	HCP
SUM_postcentral_surfavg	ICC3	0.9881	167.47	34	34	1.50E-29	0.979	0.993	v7.1	HCP
SUM_posteriorcingulate_surfavg	ICC3	0.9649	56.05	34	34	1.24E-21	0.939	0.980	v5.3	HCP
SUM_posteriorcingulate_surfavg	ICC2	0.8652	14.21	34	34	3.34E-12	0.774	0.921	v5.3 vs. v6.0	HCP
SUM_posteriorcingulate_surfavg	ICC2	0.8918	17.14	34	34	1.99E-13	0.816	0.937	v5.3 vs. v7.1	HCP
SUM_posteriorcingulate_surfavg	ICC3	0.9915	235.39	34	34	4.87E-32	0.985	0.995	v6.0	HCP
SUM_posteriorcingulate_surfavg	ICC2	0.9867	200.84	34	34	7.07E-31	0.966	0.994	v6.0 vs. v7.1	HCP
SUM_posteriorcingulate_surfavg	ICC3	0.9879	164.56	34	34	2.02E-29	0.979	0.993	v7.1	HCP
SUM_precentral_surfavg	ICC3	0.9897	193.08	34	34	1.37E-30	0.982	0.994	v5.3	HCP
SUM_precentral_surfavg	ICC2	0.9835	117.10	34	34	6.06E-27	0.971	0.991	v5.3 vs. v6.0	HCP
SUM_precentral_surfavg	ICC2	0.9822	108.27	34	34	2.25E-26	0.969	0.990	v5.3 vs. v7.1	HCP
SUM_precentral_surfavg	ICC3	0.9912	225.95	34	34	9.71E-32	0.984	0.995	v6.0	HCP
SUM_precentral_surfavg	ICC2	0.9974	759.33	34	34	1.21E-40	0.995	0.999	v6.0 vs. v7.1	HCP
SUM_precentral_surfavg	ICC3	0.9925	266.02	34	34	6.18E-33	0.987	0.996	v7.1	HCP
SUM_precuneus_surfavg	ICC3	0.9923	257.14	34	34	1.10E-32	0.986	0.996	v5.3	HCP
SUM_precuneus_surfavg	ICC2	0.8538	12.81	34	34	1.55E-11	0.756	0.915	v5.3 vs. v6.0	HCP
SUM_precuneus_surfavg	ICC2	0.7536	7.08	34	34	6.04E-08	0.603	0.853	v5.3 vs. v7.1	HCP
SUM_precuneus_surfavg	ICC3	0.9967	610.29	34	34	4.90E-39	0.994	0.998	v6.0	HCP
SUM_precuneus_surfavg	ICC2	0.9664	56.91	34	34	9.69E-22	0.941	0.981	v6.0 vs. v7.1	HCP
SUM_precuneus_surfavg	ICC3	0.9939	328.30	34	34	1.77E-34	0.989	0.997	v7.1	HCP
SUM_rostralanteriorcingulate_surfavg	ICC3	0.9793	95.45	34	34	1.84E-25	0.964	0.988	v5.3	HCP
SUM_rostralanteriorcingulate_surfavg	ICC2	0.8675	48.50	34	34	1.33E-20	0.151	0.957	v5.3 vs. v6.0	HCP
SUM_rostralanteriorcingulate_surfavg	ICC2	0.8892	26.60	34	34	2.13E-16	0.671	0.950	v5.3 vs. v7.1	HCP
SUM_rostralanteriorcingulate_surfavg	ICC3	0.9780	90.10	34	34	4.81E-25	0.961	0.988	v6.0	HCP

SUM_rostralanteriorcingulate_surfavg	ICC2	0.9677	87.27	34	34	8.19E-25	0.909	0.985	v6.0 vs. v7.1	HCP
SUM_rostralanteriorcingulate_surfavg	ICC3	0.9861	142.83	34	34	2.18E-28	0.975	0.992	v7.1	HCP
SUM_rostralmiddlefrontal_surfavg	ICC3	0.9960	502.82	34	34	1.30E-37	0.993	0.998	v5.3	HCP
SUM_rostralmiddlefrontal_surfavg	ICC2	0.9828	115.88	34	34	7.23E-27	0.970	0.990	v5.3 vs. v6.0	HCP
SUM_rostralmiddlefrontal_surfavg	ICC2	0.9648	65.76	34	34	8.94E-23	0.930	0.981	v5.3 vs. v7.1	HCP
SUM_rostralmiddlefrontal_surfavg	ICC3	0.9970	664.98	34	34	1.14E-39	0.995	0.998	v6.0	HCP
SUM_rostralmiddlefrontal_surfavg	ICC2	0.9638	72.27	34	34	1.87E-23	0.912	0.982	v6.0 vs. v7.1	HCP
SUM_rostralmiddlefrontal_surfavg	ICC3	0.9974	767.59	34	34	1.00E-40	0.995	0.999	v7.1	HCP
SUM_superiorfrontal_surfavg	ICC3	0.9953	423.38	34	34	2.40E-36	0.992	0.997	v5.3	HCP
SUM_superiorfrontal_surfavg	ICC2	0.9663	80.48	34	34	3.15E-24	0.912	0.984	v5.3 vs. v6.0	HCP
SUM_superiorfrontal_surfavg	ICC2	0.9320	50.68	34	34	6.49E-21	0.723	0.972	v5.3 vs. v7.1	HCP
SUM_superiorfrontal_surfavg	ICC3	0.9965	569.29	34	34	1.59E-38	0.994	0.998	v6.0	HCP
SUM_superiorfrontal_surfavg	ICC2	0.9730	91.21	34	34	3.93E-25	0.941	0.986	v6.0 vs. v7.1	HCP
SUM_superiorfrontal_surfavg	ICC3	0.9976	833.15	34	34	2.50E-41	0.996	0.999	v7.1	HCP
SUM_superiorparietal_surfavg	ICC3	0.9940	329.98	34	34	1.62E-34	0.989	0.997	v5.3	HCP
SUM_superiorparietal_surfavg	ICC2	0.9242	39.63	34	34	3.57E-19	0.762	0.967	v5.3 vs. v6.0	HCP
SUM_superiorparietal_surfavg	ICC2	0.9063	26.89	34	34	1.79E-16	0.786	0.953	v5.3 vs. v7.1	HCP
SUM_superiorparietal_surfavg	ICC3	0.9963	540.74	34	34	3.81E-38	0.993	0.998	v6.0	HCP
SUM_superiorparietal_surfavg	ICC2	0.9665	57.27	34	34	8.73E-22	0.941	0.981	v6.0 vs. v7.1	HCP
SUM_superiorparietal_surfavg	ICC3	0.9929	279.70	34	34	2.65E-33	0.987	0.996	v7.1	HCP
SUM_superiortemporal_surfavg	ICC3	0.9893	186.42	34	34	2.48E-30	0.981	0.994	v5.3	HCP
SUM_superiortemporal_surfavg	ICC2	0.9309	106.80	34	34	2.83E-26	0.284	0.979	v5.3 vs. v6.0	HCP
SUM_superiortemporal_surfavg	ICC2	0.8858	116.78	34	34	6.35E-27	0.048	0.967	v5.3 vs. v7.1	HCP
SUM_superiortemporal_surfavg	ICC3	0.9909	217.74	34	34	1.81E-31	0.984	0.995	v6.0	HCP
SUM_superiortemporal_surfavg	ICC2	0.9867	512.34	34	34	9.49E-38	0.770	0.996	v6.0 vs. v7.1	HCP
SUM_superiortemporal_surfavg	ICC3	0.9950	398.35	34	34	6.72E-36	0.991	0.997	v7.1	HCP
SUM_supramarginal_surfavg	ICC3	0.9955	439.46	34	34	1.28E-36	0.992	0.997	v5.3	HCP
SUM_supramarginal_surfavg	ICC2	0.9532	43.03	34	34	9.38E-20	0.919	0.973	v5.3 vs. v6.0	HCP
SUM_supramarginal_surfavg	ICC2	0.9401	34.49	34	34	3.37E-18	0.895	0.966	v5.3 vs. v7.1	HCP
SUM_supramarginal_surfavg	ICC3	0.9967	613.53	34	34	4.48E-39	0.994	0.998	v6.0	HCP
SUM_supramarginal_surfavg	ICC2	0.9887	177.92	34	34	5.43E-30	0.980	0.994	v6.0 vs. v7.1	HCP
SUM_supramarginal_surfavg	ICC3	0.9977	861.52	34	34	1.42E-41	0.996	0.999	v7.1	HCP
SUM_SurfArea	ICC3	0.9984	1268.40	34	34	2.00E-44	0.997	0.999	v5.3	HCP
SUM_SurfArea	ICC2	0.9966	1981.66	34	34	1.02E-47	0.934	0.999	v5.3 vs. v6.0	HCP
SUM_SurfArea	ICC2	0.9825	1449.50	34	34	2.07E-45	0.305	0.995	v5.3 vs. v7.1	HCP
SUM_SurfArea	ICC3	0.9980	977.44	34	34	1.66E-42	0.996	0.999	v6.0	HCP
SUM_SurfArea	ICC2	0.9934	4313.97	34	34	1.87E-53	0.535	0.998	v6.0 vs. v7.1	HCP
SUM_SurfArea	ICC3	0.9985	1363.00	34	34	5.89E-45	0.997	0.999	v7.1	HCP
SUM_temporalpole_surfavg	ICC3	0.8755	15.06	34	34	1.41E-12	0.789	0.928	v5.3	HCP
SUM_temporalpole_surfavg	ICC2	0.7618	11.75	34	34	5.48E-11	0.396	0.888	v5.3 vs. v6.0	HCP
SUM_temporalpole_surfavg	ICC2	0.5848	9.14	34	34	1.95E-09	-0.007	0.817	v5.3 vs. v7.1	HCP
SUM_temporalpole_surfavg	ICC3	0.9157	22.72	34	34	2.55E-15	0.855	0.952	v6.0	HCP
SUM_temporalpole_surfavg	ICC2	0.8120	18.64	34	34	5.53E-14	0.349	0.922	v6.0 vs. v7.1	HCP
SUM_temporalpole_surfavg	ICC3	0.9217	24.55	34	34	7.57E-16	0.865	0.955	v7.1	HCP

SUM_transversetemporal_surfavg	ICC3	0.9632	53.37	34	34	2.78E-21	0.936	0.979	v5.3	HCP
SUM_transversetemporal_surfavg	ICC2	0.8481	14.34	34	34	2.94E-12	0.718	0.916	v5.3 vs. v6.0	HCP
SUM_transversetemporal_surfavg	ICC2	0.9042	19.37	34	34	3.06E-14	0.836	0.945	v5.3 vs. v7.1	HCP
SUM_transversetemporal_surfavg	ICC3	0.9452	35.51	34	34	2.10E-18	0.905	0.969	v6.0	HCP
SUM_transversetemporal_surfavg	ICC2	0.9206	39.69	34	34	3.49E-19	0.729	0.966	v6.0 vs. v7.1	HCP
SUM_transversetemporal_surfavg	ICC3	0.9263	26.13	34	34	2.83E-16	0.873	0.958	v7.1	HCP

Table S4. KKI specific ICCs and associated statistics.

ROI	Type	ICC	F	df1	df2	p	lower bound	upper bound	comparison	dataset
AVG_Accumbens	ICC3	0.780	8.08	20	20	9.44E-06	0.584	0.890	v5.3	KKI
AVG_Accumbens	ICC2	0.438	10.70	20	20	9.31E-07	-0.055	0.755	v5.3 vs. v6.0	KKI
AVG_Accumbens	ICC2	0.666	12.24	20	20	2.96E-07	0.025	0.872	v5.3 vs. v7.1	KKI
AVG_Accumbens	ICC3	0.922	24.64	20	20	5.44E-10	0.841	0.963	v6.0	KKI
AVG_Accumbens	ICC2	0.737	13.23	20	20	1.50E-07	0.178	0.897	v6.0 vs. v7.1	KKI
AVG_Accumbens	ICC3	0.813	9.68	20	20	2.15E-06	0.640	0.907	v7.1	KKI
AVG_Amygdala	ICC3	0.929	27.13	20	20	2.21E-10	0.855	0.966	v5.3	KKI
AVG_Amygdala	ICC2	0.753	14.05	20	20	8.87E-08	0.208	0.904	v5.3 vs. v6.0	KKI
AVG_Amygdala	ICC2	0.760	10.23	20	20	1.36E-06	0.441	0.891	v5.3 vs. v7.1	KKI
AVG_Amygdala	ICC3	0.961	50.03	20	20	6.56E-13	0.919	0.981	v6.0	KKI
AVG_Amygdala	ICC2	0.933	31.90	20	20	4.83E-11	0.859	0.968	v6.0 vs. v7.1	KKI
AVG_Amygdala	ICC3	0.949	38.00	20	20	9.19E-12	0.894	0.976	v7.1	KKI
AVG_bankssts_thickavg	ICC3	0.920	23.92	20	20	7.15E-10	0.837	0.961	v5.3	KKI
AVG_bankssts_thickavg	ICC2	0.950	47.01	20	20	1.20E-12	0.882	0.977	v5.3 vs. v6.0	KKI
AVG_bankssts_thickavg	ICC2	0.955	45.15	20	20	1.76E-12	0.907	0.978	v5.3 vs. v7.1	KKI
AVG_bankssts_thickavg	ICC3	0.941	33.10	20	20	3.40E-11	0.879	0.972	v6.0	KKI
AVG_bankssts_thickavg	ICC2	0.945	70.77	20	20	2.27E-14	0.694	0.981	v6.0 vs. v7.1	KKI
AVG_bankssts_thickavg	ICC3	0.920	23.85	20	20	7.35E-10	0.836	0.961	v7.1	KKI
AVG_caudalanteriorcingulate_thickavg	ICC3	0.950	39.29	20	20	6.67E-12	0.897	0.976	v5.3	KKI
AVG_caudalanteriorcingulate_thickavg	ICC2	0.935	45.62	20	20	1.59E-12	0.779	0.974	v5.3 vs. v6.0	KKI
AVG_caudalanteriorcingulate_thickavg	ICC2	0.490	12.51	20	20	2.45E-07	-0.054	0.791	v5.3 vs. v7.1	KKI
AVG_caudalanteriorcingulate_thickavg	ICC3	0.955	43.83	20	20	2.34E-12	0.908	0.979	v6.0	KKI
AVG_caudalanteriorcingulate_thickavg	ICC2	0.619	18.06	20	20	9.43E-09	-0.043	0.862	v6.0 vs. v7.1	KKI
AVG_caudalanteriorcingulate_thickavg	ICC3	0.965	56.05	20	20	2.19E-13	0.927	0.983	v7.1	KKI
AVG_caudalmiddlefrontal_thickavg	ICC3	0.694	5.54	20	20	1.70E-04	0.445	0.843	v5.3	KKI
AVG_caudalmiddlefrontal_thickavg	ICC2	0.811	17.19	20	20	1.47E-08	0.371	0.926	v5.3 vs. v6.0	KKI
AVG_caudalmiddlefrontal_thickavg	ICC2	0.869	28.25	20	20	1.52E-10	0.441	0.953	v5.3 vs. v7.1	KKI
AVG_caudalmiddlefrontal_thickavg	ICC3	0.777	7.98	20	20	1.04E-05	0.580	0.889	v6.0	KKI
AVG_caudalmiddlefrontal_thickavg	ICC2	0.915	22.06	20	20	1.51E-09	0.829	0.959	v6.0 vs. v7.1	KKI
AVG_caudalmiddlefrontal_thickavg	ICC3	0.784	8.25	20	20	7.98E-06	0.590	0.892	v7.1	KKI
AVG_Caudate	ICC3	0.986	138.85	20	20	3.04E-17	0.970	0.993	v5.3	KKI
AVG_Caudate	ICC2	0.856	293.89	20	20	1.81E-20	0.016	0.962	v5.3 vs. v6.0	KKI
AVG_Caudate	ICC2	0.922	215.12	20	20	4.00E-19	0.077	0.979	v5.3 vs. v7.1	KKI
AVG_Caudate	ICC3	0.996	487.36	20	20	1.18E-22	0.991	0.998	v6.0	KKI
AVG_Caudate	ICC2	0.980	551.88	20	20	3.41E-23	0.481	0.995	v6.0 vs. v7.1	KKI
AVG_Caudate	ICC3	0.994	326.03	20	20	6.44E-21	0.987	0.997	v7.1	KKI
AVG_cuneus_thickavg	ICC3	0.970	66.15	20	20	4.39E-14	0.938	0.986	v5.3	KKI
AVG_cuneus_thickavg	ICC2	0.797	81.43	20	20	5.77E-15	-0.004	0.941	v5.3 vs. v6.0	KKI
AVG_cuneus_thickavg	ICC2	0.828	27.41	20	20	2.01E-10	0.188	0.942	v5.3 vs. v7.1	KKI
AVG_cuneus_thickavg	ICC3	0.964	55.22	20	20	2.53E-13	0.926	0.983	v6.0	KKI
AVG_cuneus_thickavg	ICC2	0.950	65.39	20	20	4.91E-14	0.796	0.981	v6.0 vs. v7.1	KKI

AVG_cuneus_thickavg	ICC3	0.953	41.27	20	20	4.18E-12	0.902	0.977	v7.1	KKI
AVG_entorhinal_thickavg	ICC3	0.751	7.04	20	20	2.81E-05	0.536	0.875	v5.3	KKI
AVG_entorhinal_thickavg	ICC2	0.909	24.93	20	20	4.87E-10	0.796	0.958	v5.3 vs. v6.0	KKI
AVG_entorhinal_thickavg	ICC2	0.568	11.72	20	20	4.28E-07	-0.050	0.830	v5.3 vs. v7.1	KKI
AVG_entorhinal_thickavg	ICC3	0.840	11.53	20	20	4.91E-07	0.689	0.922	v6.0	KKI
AVG_entorhinal_thickavg	ICC2	0.713	19.58	20	20	4.52E-09	-0.002	0.900	v6.0 vs. v7.1	KKI
AVG_entorhinal_thickavg	ICC3	0.771	7.75	20	20	1.31E-05	0.570	0.885	v7.1	KKI
AVG_frontalpole_thickavg	ICC3	0.877	15.26	20	20	4.26E-08	0.756	0.940	v5.3	KKI
AVG_frontalpole_thickavg	ICC2	0.825	13.37	20	20	1.37E-07	0.606	0.920	v5.3 vs. v6.0	KKI
AVG_frontalpole_thickavg	ICC2	0.840	13.31	20	20	1.42E-07	0.673	0.923	v5.3 vs. v7.1	KKI
AVG_frontalpole_thickavg	ICC3	0.875	14.96	20	20	5.08E-08	0.751	0.939	v6.0	KKI
AVG_frontalpole_thickavg	ICC2	0.893	17.40	20	20	1.32E-08	0.788	0.948	v6.0 vs. v7.1	KKI
AVG_frontalpole_thickavg	ICC3	0.817	9.95	20	20	1.71E-06	0.648	0.910	v7.1	KKI
AVG_fusiform_thickavg	ICC3	0.923	25.05	20	20	4.66E-10	0.844	0.963	v5.3	KKI
AVG_fusiform_thickavg	ICC2	0.957	43.10	20	20	2.75E-12	0.910	0.979	v5.3 vs. v6.0	KKI
AVG_fusiform_thickavg	ICC2	0.772	16.24	20	20	2.45E-08	0.204	0.914	v5.3 vs. v7.1	KKI
AVG_fusiform_thickavg	ICC3	0.923	24.83	20	20	5.07E-10	0.842	0.963	v6.0	KKI
AVG_fusiform_thickavg	ICC2	0.820	42.64	20	20	3.05E-12	0.035	0.945	v6.0 vs. v7.1	KKI
AVG_fusiform_thickavg	ICC3	0.884	16.27	20	20	2.41E-08	0.769	0.944	v7.1	KKI
AVG_Hippocampus	ICC3	0.965	56.40	20	20	2.06E-13	0.927	0.983	v5.3	KKI
AVG_Hippocampus	ICC2	0.842	44.07	20	20	2.23E-12	0.077	0.951	v5.3 vs. v6.0	KKI
AVG_Hippocampus	ICC2	0.908	54.38	20	20	2.93E-13	0.383	0.971	v5.3 vs. v7.1	KKI
AVG_Hippocampus	ICC3	0.958	46.17	20	20	1.42E-12	0.912	0.980	v6.0	KKI
AVG_Hippocampus	ICC2	0.965	91.87	20	20	1.77E-15	0.860	0.987	v6.0 vs. v7.1	KKI
AVG_Hippocampus	ICC3	0.954	42.76	20	20	2.97E-12	0.905	0.978	v7.1	KKI
AVG_inferiorparietal_thickavg	ICC3	0.752	7.07	20	20	2.70E-05	0.538	0.875	v5.3	KKI
AVG_inferiorparietal_thickavg	ICC2	0.744	10.68	20	20	9.47E-07	0.340	0.890	v5.3 vs. v6.0	KKI
AVG_inferiorparietal_thickavg	ICC2	0.823	11.36	20	20	5.61E-07	0.653	0.913	v5.3 vs. v7.1	KKI
AVG_inferiorparietal_thickavg	ICC3	0.795	8.78	20	20	4.81E-06	0.610	0.898	v6.0	KKI
AVG_inferiorparietal_thickavg	ICC2	0.920	50.54	20	20	5.95E-13	0.560	0.972	v6.0 vs. v7.1	KKI
AVG_inferiorparietal_thickavg	ICC3	0.806	9.30	20	20	3.00E-06	0.628	0.904	v7.1	KKI
AVG_inferiortemporal_thickavg	ICC3	0.933	28.85	20	20	1.25E-10	0.863	0.968	v5.3	KKI
AVG_inferiortemporal_thickavg	ICC2	0.928	29.64	20	20	9.66E-11	0.850	0.966	v5.3 vs. v6.0	KKI
AVG_inferiortemporal_thickavg	ICC2	0.889	28.66	20	20	1.33E-10	0.604	0.957	v5.3 vs. v7.1	KKI
AVG_inferiortemporal_thickavg	ICC3	0.896	18.15	20	20	9.00E-09	0.790	0.949	v6.0	KKI
AVG_inferiortemporal_thickavg	ICC2	0.940	45.05	20	20	1.80E-12	0.828	0.975	v6.0 vs. v7.1	KKI
AVG_inferiortemporal_thickavg	ICC3	0.946	35.75	20	20	1.64E-11	0.888	0.974	v7.1	KKI
AVG_insula_thickavg	ICC3	0.888	16.91	20	20	1.70E-08	0.777	0.946	v5.3	KKI
AVG_insula_thickavg	ICC2	0.842	12.47	20	20	2.51E-07	0.691	0.922	v5.3 vs. v6.0	KKI
AVG_insula_thickavg	ICC2	0.735	16.80	20	20	1.81E-08	0.069	0.904	v5.3 vs. v7.1	KKI
AVG_insula_thickavg	ICC3	0.857	13.03	20	20	1.71E-07	0.720	0.930	v6.0	KKI
AVG_insula_thickavg	ICC2	0.752	10.07	20	20	1.55E-06	0.413	0.888	v6.0 vs. v7.1	KKI
AVG_insula_thickavg	ICC3	0.873	14.76	20	20	5.74E-08	0.748	0.938	v7.1	KKI
AVG_isthmuscingulate_thickavg	ICC3	0.794	8.72	20	20	5.11E-06	0.608	0.898	v5.3	KKI

AVG_isthmuscingulate_thickavg	ICC2	0.740	10.86	20	20	8.23E-07	0.313	0.890	v5.3 vs. v6.0	KKI
AVG_isthmuscingulate_thickavg	ICC2	0.463	5.44	20	20	1.93E-04	-0.035	0.745	v5.3 vs. v7.1	KKI
AVG_isthmuscingulate_thickavg	ICC3	0.930	27.60	20	20	1.89E-10	0.857	0.966	v6.0	KKI
AVG_isthmuscingulate_thickavg	ICC2	0.813	19.96	20	20	3.79E-09	0.284	0.931	v6.0 vs. v7.1	KKI
AVG_isthmuscingulate_thickavg	ICC3	0.905	20.16	20	20	3.46E-09	0.809	0.954	v7.1	KKI
AVG_lateraloccipital_thickavg	ICC3	0.938	31.41	20	20	5.59E-11	0.873	0.970	v5.3	KKI
AVG_lateraloccipital_thickavg	ICC2	0.790	46.89	20	20	1.23E-12	-0.003	0.936	v5.3 vs. v6.0	KKI
AVG_lateraloccipital_thickavg	ICC2	0.838	36.23	20	20	1.45E-11	0.119	0.948	v5.3 vs. v7.1	KKI
AVG_lateraloccipital_thickavg	ICC3	0.913	21.92	20	20	1.61E-09	0.823	0.958	v6.0	KKI
AVG_lateraloccipital_thickavg	ICC2	0.965	86.63	20	20	3.15E-15	0.870	0.986	v6.0 vs. v7.1	KKI
AVG_lateraloccipital_thickavg	ICC3	0.941	32.86	20	20	3.65E-11	0.879	0.972	v7.1	KKI
AVG_lateralorbitofrontal_thickavg	ICC3	0.941	32.93	20	20	3.58E-11	0.879	0.972	v5.3	KKI
AVG_lateralorbitofrontal_thickavg	ICC2	0.923	27.00	20	20	2.31E-10	0.842	0.963	v5.3 vs. v6.0	KKI
AVG_lateralorbitofrontal_thickavg	ICC2	0.834	25.10	20	20	4.57E-10	0.267	0.942	v5.3 vs. v7.1	KKI
AVG_lateralorbitofrontal_thickavg	ICC3	0.933	28.71	20	20	1.30E-10	0.862	0.968	v6.0	KKI
AVG_lateralorbitofrontal_thickavg	ICC2	0.859	21.53	20	20	1.89E-09	0.540	0.944	v6.0 vs. v7.1	KKI
AVG_lateralorbitofrontal_thickavg	ICC3	0.925	25.79	20	20	3.56E-10	0.848	0.964	v7.1	KKI
AVG_LateralVentricle	ICC3	1.000	4290.15	20	20	4.36E-32	0.999	1.000	v5.3	KKI
AVG_LateralVentricle	ICC2	0.995	5747.89	20	20	2.34E-33	0.595	0.999	v5.3 vs. v6.0	KKI
AVG_LateralVentricle	ICC2	0.993	5435.82	20	20	4.09E-33	0.480	0.998	v5.3 vs. v7.1	KKI
AVG_LateralVentricle	ICC3	1.000	5105.60	20	20	7.65E-33	0.999	1.000	v6.0	KKI
AVG_LateralVentricle	ICC2	1.000	11983.17	20	20	1.51E-36	0.998	1.000	v6.0 vs. v7.1	KKI
AVG_LateralVentricle	ICC3	1.000	7202.68	20	20	2.45E-34	0.999	1.000	v7.1	KKI
AVG_lingual_thickavg	ICC3	0.959	47.43	20	20	1.10E-12	0.914	0.980	v5.3	KKI
AVG_lingual_thickavg	ICC2	0.897	33.44	20	20	3.09E-11	0.574	0.962	v5.3 vs. v6.0	KKI
AVG_lingual_thickavg	ICC2	0.940	30.81	20	20	6.71E-11	0.876	0.971	v5.3 vs. v7.1	KKI
AVG_lingual_thickavg	ICC3	0.937	30.86	20	20	6.62E-11	0.871	0.970	v6.0	KKI
AVG_lingual_thickavg	ICC2	0.922	116.96	20	20	1.65E-16	0.170	0.978	v6.0 vs. v7.1	KKI
AVG_lingual_thickavg	ICC3	0.946	36.00	20	20	1.53E-11	0.889	0.974	v7.1	KKI
AVG_medialorbitofrontal_thickavg	ICC3	0.880	15.69	20	20	3.32E-08	0.762	0.942	v5.3	KKI
AVG_medialorbitofrontal_thickavg	ICC2	0.877	14.61	20	20	6.28E-08	0.755	0.940	v5.3 vs. v6.0	KKI
AVG_medialorbitofrontal_thickavg	ICC2	0.803	23.12	20	20	9.82E-10	0.156	0.932	v5.3 vs. v7.1	KKI
AVG_medialorbitofrontal_thickavg	ICC3	0.922	24.62	20	20	5.49E-10	0.841	0.962	v6.0	KKI
AVG_medialorbitofrontal_thickavg	ICC2	0.764	15.45	20	20	3.81E-08	0.200	0.911	v6.0 vs. v7.1	KKI
AVG_medialorbitofrontal_thickavg	ICC3	0.907	20.48	20	20	2.99E-09	0.812	0.955	v7.1	KKI
AVG_middletemporal_thickavg	ICC3	0.923	25.01	20	20	4.73E-10	0.843	0.963	v5.3	KKI
AVG_middletemporal_thickavg	ICC2	0.887	21.71	20	20	1.75E-09	0.723	0.950	v5.3 vs. v6.0	KKI
AVG_middletemporal_thickavg	ICC2	0.729	16.95	20	20	1.66E-08	0.051	0.902	v5.3 vs. v7.1	KKI
AVG_middletemporal_thickavg	ICC3	0.910	21.30	20	20	2.09E-09	0.819	0.957	v6.0	KKI
AVG_middletemporal_thickavg	ICC2	0.862	47.00	20	20	1.20E-12	0.128	0.958	v6.0 vs. v7.1	KKI
AVG_middletemporal_thickavg	ICC3	0.921	24.18	20	20	6.47E-10	0.839	0.962	v7.1	KKI
AVG_Pallidum	ICC3	0.858	13.11	20	20	1.63E-07	0.721	0.931	v5.3	KKI
AVG_Pallidum	ICC2	0.234	3.96	20	20	1.69E-03	-0.068	0.545	v5.3 vs. v6.0	KKI
AVG_Pallidum	ICC2	0.269	4.59	20	20	6.37E-04	-0.068	0.590	v5.3 vs. v7.1	KKI

AVG_Pallidum	ICC3	0.968	60.85	20	20	9.87E-14	0.933	0.985	v6.0	KKI
AVG_Pallidum	ICC2	0.891	16.63	20	20	1.98E-08	0.782	0.947	v6.0 vs. v7.1	KKI
AVG_Pallidum	ICC3	0.945	35.19	20	20	1.91E-11	0.886	0.974	v7.1	KKI
AVG_paracentral_thickavg	ICC3	0.913	22.10	20	20	1.49E-09	0.825	0.958	v5.3	KKI
AVG_paracentral_thickavg	ICC2	0.830	20.59	20	20	2.85E-09	0.369	0.936	v5.3 vs. v6.0	KKI
AVG_paracentral_thickavg	ICC2	0.835	16.95	20	20	1.67E-08	0.530	0.931	v5.3 vs. v7.1	KKI
AVG_paracentral_thickavg	ICC3	0.887	16.69	20	20	1.92E-08	0.774	0.945	v6.0	KKI
AVG_paracentral_thickavg	ICC2	0.939	31.51	20	20	5.42E-11	0.877	0.971	v6.0 vs. v7.1	KKI
AVG_paracentral_thickavg	ICC3	0.894	17.92	20	20	1.01E-08	0.788	0.949	v7.1	KKI
AVG_parahippocampal_thickavg	ICC3	0.902	19.43	20	20	4.85E-09	0.803	0.953	v5.3	KKI
AVG_parahippocampal_thickavg	ICC2	0.945	34.65	20	20	2.21E-11	0.888	0.974	v5.3 vs. v6.0	KKI
AVG_parahippocampal_thickavg	ICC2	0.776	23.29	20	20	9.18E-10	0.066	0.924	v5.3 vs. v7.1	KKI
AVG_parahippocampal_thickavg	ICC3	0.961	49.83	20	20	6.82E-13	0.918	0.981	v6.0	KKI
AVG_parahippocampal_thickavg	ICC2	0.810	38.98	20	20	7.21E-12	0.031	0.941	v6.0 vs. v7.1	KKI
AVG_parahippocampal_thickavg	ICC3	0.955	43.52	20	20	2.51E-12	0.907	0.979	v7.1	KKI
AVG_parsopercularis_thickavg	ICC3	0.879	15.59	20	20	3.53E-08	0.760	0.941	v5.3	KKI
AVG_parsopercularis_thickavg	ICC2	0.838	16.26	20	20	2.42E-08	0.575	0.930	v5.3 vs. v6.0	KKI
AVG_parsopercularis_thickavg	ICC2	0.906	21.99	20	20	1.56E-09	0.808	0.955	v5.3 vs. v7.1	KKI
AVG_parsopercularis_thickavg	ICC3	0.895	18.13	20	20	9.07E-09	0.790	0.949	v6.0	KKI
AVG_parsopercularis_thickavg	ICC2	0.940	45.58	20	20	1.61E-12	0.824	0.975	v6.0 vs. v7.1	KKI
AVG_parsopercularis_thickavg	ICC3	0.895	18.01	20	20	9.65E-09	0.789	0.949	v7.1	KKI
AVG_parsorbitalis_thickavg	ICC3	0.932	28.25	20	20	1.52E-10	0.860	0.967	v5.3	KKI
AVG_parsorbitalis_thickavg	ICC2	0.893	17.57	20	20	1.21E-08	0.788	0.948	v5.3 vs. v6.0	KKI
AVG_parsorbitalis_thickavg	ICC2	0.925	24.62	20	20	5.47E-10	0.847	0.964	v5.3 vs. v7.1	KKI
AVG_parsorbitalis_thickavg	ICC3	0.920	24.09	20	20	6.70E-10	0.838	0.962	v6.0	KKI
AVG_parsorbitalis_thickavg	ICC2	0.967	63.49	20	20	6.53E-14	0.932	0.984	v6.0 vs. v7.1	KKI
AVG_parsorbitalis_thickavg	ICC3	0.956	44.54	20	20	2.01E-12	0.909	0.979	v7.1	KKI
AVG_parstriangularis_thickavg	ICC3	0.946	35.71	20	20	1.66E-11	0.888	0.974	v5.3	KKI
AVG_parstriangularis_thickavg	ICC2	0.942	47.39	20	20	1.11E-12	0.831	0.976	v5.3 vs. v6.0	KKI
AVG_parstriangularis_thickavg	ICC2	0.949	45.26	20	20	1.72E-12	0.885	0.977	v5.3 vs. v7.1	KKI
AVG_parstriangularis_thickavg	ICC3	0.954	42.53	20	20	3.13E-12	0.905	0.978	v6.0	KKI
AVG_parstriangularis_thickavg	ICC2	0.964	54.61	20	20	2.81E-13	0.926	0.983	v6.0 vs. v7.1	KKI
AVG_parstriangularis_thickavg	ICC3	0.937	30.58	20	20	7.21E-11	0.870	0.970	v7.1	KKI
AVG_pericalcarine_thickavg	ICC3	0.903	19.61	20	20	4.46E-09	0.805	0.953	v5.3	KKI
AVG_pericalcarine_thickavg	ICC2	0.729	18.99	20	20	5.98E-09	0.025	0.905	v5.3 vs. v6.0	KKI
AVG_pericalcarine_thickavg	ICC2	0.829	21.73	20	20	1.74E-09	0.325	0.937	v5.3 vs. v7.1	KKI
AVG_pericalcarine_thickavg	ICC3	0.885	16.35	20	20	2.31E-08	0.770	0.944	v6.0	KKI
AVG_pericalcarine_thickavg	ICC2	0.938	49.33	20	20	7.51E-13	0.780	0.976	v6.0 vs. v7.1	KKI
AVG_pericalcarine_thickavg	ICC3	0.946	35.70	20	20	1.66E-11	0.888	0.974	v7.1	KKI
AVG_postcentral_thickavg	ICC3	0.922	24.50	20	20	5.73E-10	0.840	0.962	v5.3	KKI
AVG_postcentral_thickavg	ICC2	0.850	61.30	20	20	9.18E-14	0.042	0.956	v5.3 vs. v6.0	KKI
AVG_postcentral_thickavg	ICC2	0.895	92.17	20	20	1.72E-15	0.094	0.970	v5.3 vs. v7.1	KKI
AVG_postcentral_thickavg	ICC3	0.982	109.06	20	20	3.29E-16	0.962	0.991	v6.0	KKI
AVG_postcentral_thickavg	ICC2	0.975	90.83	20	20	1.98E-15	0.942	0.988	v6.0 vs. v7.1	KKI

AVG_postcentral_thickavg	ICC3	0.978	88.25	20	20	2.63E-15	0.953	0.989	v7.1	KKI
AVG_posteriorcingulate_thickavg	ICC3	0.908	20.63	20	20	2.80E-09	0.813	0.955	v5.3	KKI
AVG_posteriorcingulate_thickavg	ICC2	0.894	37.94	20	20	9.31E-12	0.470	0.963	v5.3 vs. v6.0	KKI
AVG_posteriorcingulate_thickavg	ICC2	0.536	16.44	20	20	2.19E-08	-0.047	0.821	v5.3 vs. v7.1	KKI
AVG_posteriorcingulate_thickavg	ICC3	0.905	20.09	20	20	3.57E-09	0.809	0.954	v6.0	KKI
AVG_posteriorcingulate_thickavg	ICC2	0.689	23.48	20	20	8.51E-10	-0.029	0.895	v6.0 vs. v7.1	KKI
AVG_posteriorcingulate_thickavg	ICC3	0.918	23.38	20	20	8.83E-10	0.833	0.961	v7.1	KKI
AVG_precentral_thickavg	ICC3	0.735	6.56	20	20	4.80E-05	0.511	0.866	v5.3	KKI
AVG_precentral_thickavg	ICC2	0.831	36.07	20	20	1.51E-11	0.096	0.946	v5.3 vs. v6.0	KKI
AVG_precentral_thickavg	ICC2	0.817	11.16	20	20	6.51E-07	0.639	0.911	v5.3 vs. v7.1	KKI
AVG_precentral_thickavg	ICC3	0.897	18.37	20	20	8.06E-09	0.793	0.950	v6.0	KKI
AVG_precentral_thickavg	ICC2	0.887	20.24	20	20	3.34E-09	0.749	0.948	v6.0 vs. v7.1	KKI
AVG_precentral_thickavg	ICC3	0.834	11.08	20	20	6.91E-07	0.678	0.918	v7.1	KKI
AVG_precuneus_thickavg	ICC3	0.897	18.47	20	20	7.66E-09	0.794	0.950	v5.3	KKI
AVG_precuneus_thickavg	ICC2	0.944	72.04	20	20	1.91E-14	0.675	0.981	v5.3 vs. v6.0	KKI
AVG_precuneus_thickavg	ICC2	0.962	53.21	20	20	3.62E-13	0.921	0.982	v5.3 vs. v7.1	KKI
AVG_precuneus_thickavg	ICC3	0.917	23.14	20	20	9.72E-10	0.832	0.960	v6.0	KKI
AVG_precuneus_thickavg	ICC2	0.965	104.35	20	20	5.07E-16	0.819	0.988	v6.0 vs. v7.1	KKI
AVG_precuneus_thickavg	ICC3	0.897	18.49	20	20	7.59E-09	0.794	0.950	v7.1	KKI
AVG_Putamen	ICC3	0.952	40.71	20	20	4.76E-12	0.901	0.977	v5.3	KKI
AVG_Putamen	ICC2	0.342	16.69	20	20	1.91E-08	-0.027	0.686	v5.3 vs. v6.0	KKI
AVG_Putamen	ICC2	0.351	14.56	20	20	6.45E-08	-0.032	0.693	v5.3 vs. v7.1	KKI
AVG_Putamen	ICC3	0.996	475.84	20	20	1.49E-22	0.991	0.998	v6.0	KKI
AVG_Putamen	ICC2	0.980	120.47	20	20	1.23E-16	0.949	0.991	v6.0 vs. v7.1	KKI
AVG_Putamen	ICC3	0.987	158.04	20	20	8.48E-18	0.973	0.994	v7.1	KKI
AVG_rostralanteriorcingulate_thickavg	ICC3	0.911	21.49	20	20	1.92E-09	0.820	0.957	v5.3	KKI
AVG_rostralanteriorcingulate_thickavg	ICC2	0.861	14.07	20	20	8.75E-08	0.728	0.932	v5.3 vs. v6.0	KKI
AVG_rostralanteriorcingulate_thickavg	ICC2	0.445	7.98	20	20	1.04E-05	-0.065	0.752	v5.3 vs. v7.1	KKI
AVG_rostralanteriorcingulate_thickavg	ICC3	0.916	22.82	20	20	1.11E-09	0.830	0.960	v6.0	KKI
AVG_rostralanteriorcingulate_thickavg	ICC2	0.547	18.57	20	20	7.32E-09	-0.043	0.828	v6.0 vs. v7.1	KKI
AVG_rostralanteriorcingulate_thickavg	ICC3	0.917	23.10	20	20	9.88E-10	0.832	0.960	v7.1	KKI
AVG_rostralmiddlefrontal_thickavg	ICC3	0.767	7.60	20	20	1.54E-05	0.563	0.883	v5.3	KKI
AVG_rostralmiddlefrontal_thickavg	ICC2	0.774	21.80	20	20	1.69E-09	0.082	0.922	v5.3 vs. v6.0	KKI
AVG_rostralmiddlefrontal_thickavg	ICC2	0.826	30.11	20	20	8.32E-11	0.136	0.943	v5.3 vs. v7.1	KKI
AVG_rostralmiddlefrontal_thickavg	ICC3	0.869	14.28	20	20	7.69E-08	0.741	0.936	v6.0	KKI
AVG_rostralmiddlefrontal_thickavg	ICC2	0.951	41.78	20	20	3.71E-12	0.900	0.977	v6.0 vs. v7.1	KKI
AVG_rostralmiddlefrontal_thickavg	ICC3	0.854	12.70	20	20	2.15E-07	0.713	0.928	v7.1	KKI
AVG_superiorfrontal_thickavg	ICC3	0.825	10.43	20	20	1.15E-06	0.662	0.914	v5.3	KKI
AVG_superiorfrontal_thickavg	ICC2	0.945	35.67	20	20	1.68E-11	0.888	0.974	v5.3 vs. v6.0	KKI
AVG_superiorfrontal_thickavg	ICC2	0.955	42.05	20	20	3.49E-12	0.907	0.979	v5.3 vs. v7.1	KKI
AVG_superiorfrontal_thickavg	ICC3	0.870	14.38	20	20	7.22E-08	0.743	0.937	v6.0	KKI
AVG_superiorfrontal_thickavg	ICC2	0.964	63.93	20	20	6.11E-14	0.919	0.984	v6.0 vs. v7.1	KKI
AVG_superiorfrontal_thickavg	ICC3	0.865	13.77	20	20	1.06E-07	0.733	0.934	v7.1	KKI
AVG_superiorparietal_thickavg	ICC3	0.872	14.57	20	20	6.43E-08	0.745	0.937	v5.3	KKI

AVG_superiorparietal_thickavg	ICC2	0.881	42.57	20	20	3.10E-12	0.277	0.961	v5.3 vs. v6.0	KKI
AVG_superiorparietal_thickavg	ICC2	0.896	47.66	20	20	1.05E-12	0.341	0.966	v5.3 vs. v7.1	KKI
AVG_superiorparietal_thickavg	ICC3	0.882	16.02	20	20	2.77E-08	0.766	0.943	v6.0	KKI
AVG_superiorparietal_thickavg	ICC2	0.985	129.86	20	20	5.89E-17	0.968	0.993	v6.0 vs. v7.1	KKI
AVG_superiorparietal_thickavg	ICC3	0.889	17.08	20	20	1.56E-08	0.779	0.946	v7.1	KKI
AVG_superiortemporal_thickavg	ICC3	0.976	81.41	20	20	5.79E-15	0.949	0.989	v5.3	KKI
AVG_superiortemporal_thickavg	ICC2	0.986	138.53	20	20	3.11E-17	0.971	0.994	v5.3 vs. v6.0	KKI
AVG_superiortemporal_thickavg	ICC2	0.973	125.03	20	20	8.56E-17	0.885	0.990	v5.3 vs. v7.1	KKI
AVG_superiortemporal_thickavg	ICC3	0.970	64.93	20	20	5.25E-14	0.937	0.986	v6.0	KKI
AVG_superiortemporal_thickavg	ICC2	0.976	144.74	20	20	2.02E-17	0.892	0.991	v6.0 vs. v7.1	KKI
AVG_superiortemporal_thickavg	ICC3	0.971	67.48	20	20	3.61E-14	0.939	0.986	v7.1	KKI
AVG_supramarginal_thickavg	ICC3	0.865	13.83	20	20	1.01E-07	0.734	0.934	v5.3	KKI
AVG_supramarginal_thickavg	ICC2	0.892	22.02	20	20	1.54E-09	0.746	0.951	v5.3 vs. v6.0	KKI
AVG_supramarginal_thickavg	ICC2	0.892	17.07	20	20	1.57E-08	0.785	0.948	v5.3 vs. v7.1	KKI
AVG_supramarginal_thickavg	ICC3	0.878	15.39	20	20	3.96E-08	0.757	0.941	v6.0	KKI
AVG_supramarginal_thickavg	ICC2	0.941	46.77	20	20	1.26E-12	0.827	0.976	v6.0 vs. v7.1	KKI
AVG_supramarginal_thickavg	ICC3	0.858	13.07	20	20	1.67E-07	0.720	0.930	v7.1	KKI
AVG_temporalpole_thickavg	ICC3	0.782	8.19	20	20	8.44E-06	0.588	0.891	v5.3	KKI
AVG_temporalpole_thickavg	ICC2	0.766	9.73	20	20	2.06E-06	0.496	0.890	v5.3 vs. v6.0	KKI
AVG_temporalpole_thickavg	ICC2	0.730	17.80	20	20	1.07E-08	0.040	0.904	v5.3 vs. v7.1	KKI
AVG_temporalpole_thickavg	ICC3	0.763	7.45	20	20	1.80E-05	0.556	0.881	v6.0	KKI
AVG_temporalpole_thickavg	ICC2	0.722	7.80	20	20	1.25E-05	0.436	0.865	v6.0 vs. v7.1	KKI
AVG_temporalpole_thickavg	ICC3	0.543	3.38	20	20	4.48E-03	0.228	0.755	v7.1	KKI
AVG_Thalamus	ICC3	0.915	22.40	20	20	1.32E-09	0.827	0.959	v5.3	KKI
AVG_Thalamus	ICC2	0.836	16.30	20	20	2.37E-08	0.562	0.930	v5.3 vs. v6.0	KKI
AVG_Thalamus	ICC2	0.852	12.32	20	20	2.79E-07	0.712	0.927	v5.3 vs. v7.1	KKI
AVG_Thalamus	ICC3	0.961	50.68	20	20	5.80E-13	0.920	0.982	v6.0	KKI
AVG_Thalamus	ICC2	0.844	26.67	20	20	2.60E-10	0.295	0.945	v6.0 vs. v7.1	KKI
AVG_Thalamus	ICC3	0.976	84.06	20	20	4.23E-15	0.951	0.989	v7.1	KKI
AVG_Thickness	ICC3	0.928	26.84	20	20	2.45E-10	0.853	0.966	v5.3	KKI
AVG_Thickness	ICC2	0.925	63.45	20	20	6.58E-14	0.479	0.976	v5.3 vs. v6.0	KKI
AVG_Thickness	ICC2	0.971	65.67	20	20	4.70E-14	0.939	0.986	v5.3 vs. v7.1	KKI
AVG_Thickness	ICC3	0.942	33.42	20	20	3.11E-11	0.880	0.972	v6.0	KKI
AVG_Thickness	ICC2	0.939	95.57	20	20	1.20E-15	0.434	0.982	v6.0 vs. v7.1	KKI
AVG_Thickness	ICC3	0.934	29.33	20	20	1.07E-10	0.865	0.968	v7.1	KKI
AVG_transversetemporal_thickavg	ICC3	0.920	24.07	20	20	6.76E-10	0.838	0.962	v5.3	KKI
AVG_transversetemporal_thickavg	ICC2	0.863	56.09	20	20	2.17E-13	0.085	0.959	v5.3 vs. v6.0	KKI
AVG_transversetemporal_thickavg	ICC2	0.841	40.85	20	20	4.60E-12	0.094	0.951	v5.3 vs. v7.1	KKI
AVG_transversetemporal_thickavg	ICC3	0.887	16.77	20	20	1.83E-08	0.775	0.945	v6.0	KKI
AVG_transversetemporal_thickavg	ICC2	0.979	88.70	20	20	2.50E-15	0.955	0.990	v6.0 vs. v7.1	KKI
AVG_transversetemporal_thickavg	ICC3	0.869	14.32	20	20	7.48E-08	0.742	0.936	v7.1	KKI
ICV	ICC3	0.997	657.47	20	20	5.95E-24	0.994	0.999	v5.3	KKI
ICV	ICC2	1.000	4.03E+11	20	20	8.09E-112	1.000	1.000	v5.3 vs. v6.0	KKI
ICV	ICC2	0.985	149.16	20	20	1.50E-17	0.968	0.993	v5.3 vs. v7.1	KKI

ICV	ICC3	0.997	657.45	20	20	5.96E-24	0.994	0.999	v6.0	KKI
ICV	ICC2	0.985	149.16	20	20	1.50E-17	0.968	0.993	v6.0 vs. v7.1	KKI
ICV	ICC3	0.994	322.54	20	20	7.17E-21	0.987	0.997	v7.1	KKI
LH_Accumbens	ICC3	0.524	3.20	20	20	6.13E-03	0.203	0.744	v5.3	KKI
LH_Accumbens	ICC2	0.395	5.70	20	20	1.37E-04	-0.069	0.706	v5.3 vs. v6.0	KKI
LH_Accumbens	ICC2	0.645	8.53	20	20	6.06E-06	0.114	0.849	v5.3 vs. v7.1	KKI
LH_Accumbens	ICC3	0.867	14.08	20	20	8.68E-08	0.738	0.935	v6.0	KKI
LH_Accumbens	ICC2	0.657	9.68	20	20	2.15E-06	0.088	0.859	v6.0 vs. v7.1	KKI
LH_Accumbens	ICC3	0.675	5.15	20	20	2.85E-04	0.416	0.833	v7.1	KKI
LH_Amygdala	ICC3	0.886	16.53	20	20	2.08E-08	0.772	0.945	v5.3	KKI
LH_Amygdala	ICC2	0.810	13.18	20	20	1.55E-07	0.538	0.915	v5.3 vs. v6.0	KKI
LH_Amygdala	ICC2	0.808	9.01	20	20	3.91E-06	0.630	0.905	v5.3 vs. v7.1	KKI
LH_Amygdala	ICC3	0.906	20.33	20	20	3.20E-09	0.811	0.955	v6.0	KKI
LH_Amygdala	ICC2	0.830	15.92	20	20	2.92E-08	0.536	0.928	v6.0 vs. v7.1	KKI
LH_Amygdala	ICC3	0.903	19.65	20	20	4.38E-09	0.805	0.953	v7.1	KKI
LH_bankssts_surfavg	ICC3	0.985	135.35	20	20	3.92E-17	0.969	0.993	v5.3	KKI
LH_bankssts_surfavg	ICC2	0.701	6.79	20	20	3.70E-05	0.430	0.851	v5.3 vs. v6.0	KKI
LH_bankssts_surfavg	ICC2	0.694	6.98	20	20	2.98E-05	0.393	0.850	v5.3 vs. v7.1	KKI
LH_bankssts_surfavg	ICC3	0.978	90.54	20	20	2.04E-15	0.954	0.990	v6.0	KKI
LH_bankssts_surfavg	ICC2	0.968	62.18	20	20	8.00E-14	0.934	0.985	v6.0 vs. v7.1	KKI
LH_bankssts_surfavg	ICC3	0.970	66.29	20	20	4.29E-14	0.938	0.986	v7.1	KKI
LH_bankssts_thickavg	ICC3	0.883	16.09	20	20	2.66E-08	0.767	0.943	v5.3	KKI
LH_bankssts_thickavg	ICC2	0.935	36.73	20	20	1.27E-11	0.845	0.971	v5.3 vs. v6.0	KKI
LH_bankssts_thickavg	ICC2	0.929	26.26	20	20	3.01E-10	0.856	0.966	v5.3 vs. v7.1	KKI
LH_bankssts_thickavg	ICC3	0.921	24.42	20	20	5.90E-10	0.840	0.962	v6.0	KKI
LH_bankssts_thickavg	ICC2	0.939	49.31	20	20	7.55E-13	0.790	0.976	v6.0 vs. v7.1	KKI
LH_bankssts_thickavg	ICC3	0.936	30.43	20	20	7.55E-11	0.869	0.970	v7.1	KKI
LH_caudalanteriorcingulate_surfavg	ICC3	0.951	39.79	20	20	5.91E-12	0.899	0.977	v5.3	KKI
LH_caudalanteriorcingulate_surfavg	ICC2	0.861	16.69	20	20	1.92E-08	0.687	0.936	v5.3 vs. v6.0	KKI
LH_caudalanteriorcingulate_surfavg	ICC2	0.846	11.55	20	20	4.84E-07	0.699	0.925	v5.3 vs. v7.1	KKI
LH_caudalanteriorcingulate_surfavg	ICC3	0.946	36.28	20	20	1.43E-11	0.889	0.974	v6.0	KKI
LH_caudalanteriorcingulate_surfavg	ICC2	0.908	44.48	20	20	2.04E-12	0.511	0.968	v6.0 vs. v7.1	KKI
LH_caudalanteriorcingulate_surfavg	ICC3	0.924	25.30	20	20	4.25E-10	0.845	0.963	v7.1	KKI
LH_caudalanteriorcingulate_thickavg	ICC3	0.862	13.53	20	20	1.23E-07	0.729	0.933	v5.3	KKI
LH_caudalanteriorcingulate_thickavg	ICC2	0.886	17.51	20	20	1.25E-08	0.774	0.945	v5.3 vs. v6.0	KKI
LH_caudalanteriorcingulate_thickavg	ICC2	0.457	10.96	20	20	7.57E-07	-0.056	0.768	v5.3 vs. v7.1	KKI
LH_caudalanteriorcingulate_thickavg	ICC3	0.907	20.54	20	20	2.92E-09	0.813	0.955	v6.0	KKI
LH_caudalanteriorcingulate_thickavg	ICC2	0.523	9.14	20	20	3.46E-06	-0.054	0.801	v6.0 vs. v7.1	KKI
LH_caudalanteriorcingulate_thickavg	ICC3	0.916	22.95	20	20	1.05E-09	0.831	0.960	v7.1	KKI
LH_caudalmiddlefrontal_surfavg	ICC3	0.850	12.34	20	20	2.74E-07	0.706	0.927	v5.3	KKI
LH_caudalmiddlefrontal_surfavg	ICC2	0.804	10.58	20	20	1.02E-06	0.609	0.904	v5.3 vs. v6.0	KKI
LH_caudalmiddlefrontal_surfavg	ICC2	0.913	23.26	20	20	9.29E-10	0.823	0.958	v5.3 vs. v7.1	KKI
LH_caudalmiddlefrontal_surfavg	ICC3	0.839	11.39	20	20	5.47E-07	0.686	0.921	v6.0	KKI
LH_caudalmiddlefrontal_surfavg	ICC2	0.814	9.93	20	20	1.74E-06	0.646	0.907	v6.0 vs. v7.1	KKI

LH_caudalmiddlefrontal_surfavg	ICC3	0.818	10.00	20	20	1.64E-06	0.650	0.910	v7.1	KKI
LH_caudalmiddlefrontal_thickavg	ICC3	0.659	4.86	20	20	4.29E-04	0.392	0.823	v5.3	KKI
LH_caudalmiddlefrontal_thickavg	ICC2	0.637	5.23	20	20	2.55E-04	0.349	0.813	v5.3 vs. v6.0	KKI
LH_caudalmiddlefrontal_thickavg	ICC2	0.879	20.63	20	20	2.80E-09	0.696	0.946	v5.3 vs. v7.1	KKI
LH_caudalmiddlefrontal_thickavg	ICC3	0.644	4.61	20	20	6.15E-04	0.369	0.815	v6.0	KKI
LH_caudalmiddlefrontal_thickavg	ICC2	0.814	9.62	20	20	2.27E-06	0.645	0.907	v6.0 vs. v7.1	KKI
LH_caudalmiddlefrontal_thickavg	ICC3	0.688	5.40	20	20	2.03E-04	0.436	0.840	v7.1	KKI
LH_Caudate	ICC3	0.981	104.63	20	20	4.94E-16	0.960	0.991	v5.3	KKI
LH_Caudate	ICC2	0.887	179.87	20	20	2.36E-18	0.032	0.970	v5.3 vs. v6.0	KKI
LH_Caudate	ICC2	0.935	176.21	20	20	2.89E-18	0.157	0.983	v5.3 vs. v7.1	KKI
LH_Caudate	ICC3	0.995	371.77	20	20	1.74E-21	0.989	0.997	v6.0	KKI
LH_Caudate	ICC2	0.983	276.77	20	20	3.28E-20	0.859	0.995	v6.0 vs. v7.1	KKI
LH_Caudate	ICC3	0.990	205.99	20	20	6.15E-19	0.980	0.995	v7.1	KKI
LH_cuneus_surfavg	ICC3	0.957	45.94	20	20	1.49E-12	0.912	0.980	v5.3	KKI
LH_cuneus_surfavg	ICC2	0.913	31.04	20	20	6.25E-11	0.756	0.963	v5.3 vs. v6.0	KKI
LH_cuneus_surfavg	ICC2	0.914	32.66	20	20	3.87E-11	0.736	0.965	v5.3 vs. v7.1	KKI
LH_cuneus_surfavg	ICC3	0.981	103.11	20	20	5.71E-16	0.960	0.991	v6.0	KKI
LH_cuneus_surfavg	ICC2	0.986	136.16	20	20	3.69E-17	0.971	0.993	v6.0 vs. v7.1	KKI
LH_cuneus_surfavg	ICC3	0.978	90.43	20	20	2.07E-15	0.954	0.990	v7.1	KKI
LH_cuneus_thickavg	ICC3	0.964	53.97	20	20	3.15E-13	0.924	0.983	v5.3	KKI
LH_cuneus_thickavg	ICC2	0.817	51.37	20	20	5.09E-13	0.014	0.945	v5.3 vs. v6.0	KKI
LH_cuneus_thickavg	ICC2	0.829	18.74	20	20	6.73E-09	0.424	0.933	v5.3 vs. v7.1	KKI
LH_cuneus_thickavg	ICC3	0.953	41.59	20	20	3.87E-12	0.903	0.978	v6.0	KKI
LH_cuneus_thickavg	ICC2	0.935	42.94	20	20	2.85E-12	0.799	0.973	v6.0 vs. v7.1	KKI
LH_cuneus_thickavg	ICC3	0.955	43.11	20	20	2.75E-12	0.906	0.978	v7.1	KKI
LH_entorhinal_surfavg	ICC3	0.845	11.86	20	20	3.86E-07	0.696	0.924	v5.3	KKI
LH_entorhinal_surfavg	ICC2	0.447	4.49	20	20	7.35E-04	-0.009	0.722	v5.3 vs. v6.0	KKI
LH_entorhinal_surfavg	ICC2	0.425	3.02	20	20	8.64E-03	0.085	0.677	v5.3 vs. v7.1	KKI
LH_entorhinal_surfavg	ICC3	0.944	34.98	20	20	2.02E-11	0.886	0.973	v6.0	KKI
LH_entorhinal_surfavg	ICC2	0.909	28.36	20	20	1.46E-10	0.760	0.961	v6.0 vs. v7.1	KKI
LH_entorhinal_surfavg	ICC3	0.949	38.45	20	20	8.21E-12	0.895	0.976	v7.1	KKI
LH_entorhinal_thickavg	ICC3	0.803	9.13	20	20	3.49E-06	0.623	0.902	v5.3	KKI
LH_entorhinal_thickavg	ICC2	0.860	13.10	20	20	1.63E-07	0.727	0.931	v5.3 vs. v6.0	KKI
LH_entorhinal_thickavg	ICC2	0.540	7.97	20	20	1.05E-05	-0.034	0.803	v5.3 vs. v7.1	KKI
LH_entorhinal_thickavg	ICC3	0.766	7.54	20	20	1.64E-05	0.560	0.882	v6.0	KKI
LH_entorhinal_thickavg	ICC2	0.624	22.02	20	20	1.54E-09	-0.040	0.867	v6.0 vs. v7.1	KKI
LH_entorhinal_thickavg	ICC3	0.845	11.91	20	20	3.72E-07	0.697	0.924	v7.1	KKI
LH_frontalpole_surfavg	ICC3	0.844	11.85	20	20	3.88E-07	0.696	0.924	v5.3	KKI
LH_frontalpole_surfavg	ICC2	0.150	2.81	20	20	1.28E-02	-0.061	0.417	v5.3 vs. v6.0	KKI
LH_frontalpole_surfavg	ICC2	0.122	4.12	20	20	1.30E-03	-0.036	0.371	v5.3 vs. v7.1	KKI
LH_frontalpole_surfavg	ICC3	0.727	6.32	20	20	6.41E-05	0.497	0.861	v6.0	KKI
LH_frontalpole_surfavg	ICC2	0.496	6.06	20	20	8.74E-05	-0.026	0.768	v6.0 vs. v7.1	KKI
LH_frontalpole_surfavg	ICC3	0.771	7.75	20	20	1.31E-05	0.570	0.886	v7.1	KKI
LH_frontalpole_thickavg	ICC3	0.863	13.65	20	20	1.14E-07	0.731	0.933	v5.3	KKI

LH_frontalpole_thickavg	ICC2	0.767	8.21	20	20	8.26E-06	0.562	0.883	v5.3 vs. v6.0	KKI
LH_frontalpole_thickavg	ICC2	0.809	9.27	20	20	3.07E-06	0.635	0.905	v5.3 vs. v7.1	KKI
LH_frontalpole_thickavg	ICC3	0.857	12.96	20	20	1.80E-07	0.718	0.930	v6.0	KKI
LH_frontalpole_thickavg	ICC2	0.858	13.68	20	20	1.12E-07	0.722	0.930	v6.0 vs. v7.1	KKI
LH_frontalpole_thickavg	ICC3	0.812	9.66	20	20	2.19E-06	0.640	0.907	v7.1	KKI
LH_fusiform_surfavg	ICC3	0.968	61.82	20	20	8.47E-14	0.934	0.985	v5.3	KKI
LH_fusiform_surfavg	ICC2	0.849	21.81	20	20	1.68E-09	0.464	0.942	v5.3 vs. v6.0	KKI
LH_fusiform_surfavg	ICC2	0.901	26.78	20	20	2.50E-10	0.727	0.958	v5.3 vs. v7.1	KKI
LH_fusiform_surfavg	ICC3	0.983	119.32	20	20	1.36E-16	0.965	0.992	v6.0	KKI
LH_fusiform_surfavg	ICC2	0.979	154.69	20	20	1.05E-17	0.913	0.992	v6.0 vs. v7.1	KKI
LH_fusiform_surfavg	ICC3	0.993	274.45	20	20	3.57E-20	0.985	0.997	v7.1	KKI
LH_fusiform_thickavg	ICC3	0.922	24.60	20	20	5.51E-10	0.841	0.962	v5.3	KKI
LH_fusiform_thickavg	ICC2	0.928	25.49	20	20	3.97E-10	0.852	0.965	v5.3 vs. v6.0	KKI
LH_fusiform_thickavg	ICC2	0.747	15.37	20	20	4.00E-08	0.138	0.906	v5.3 vs. v7.1	KKI
LH_fusiform_thickavg	ICC3	0.897	18.40	20	20	7.96E-09	0.793	0.950	v6.0	KKI
LH_fusiform_thickavg	ICC2	0.805	55.97	20	20	2.22E-13	0.001	0.942	v6.0 vs. v7.1	KKI
LH_fusiform_thickavg	ICC3	0.871	14.51	20	20	6.68E-08	0.745	0.937	v7.1	KKI
LH_Hippocampus	ICC3	0.930	27.38	20	20	2.03E-10	0.856	0.966	v5.3	KKI
LH_Hippocampus	ICC2	0.860	26.34	20	20	2.93E-10	0.418	0.949	v5.3 vs. v6.0	KKI
LH_Hippocampus	ICC2	0.900	22.42	20	20	1.30E-09	0.781	0.953	v5.3 vs. v7.1	KKI
LH_Hippocampus	ICC3	0.951	39.63	20	20	6.14E-12	0.898	0.977	v6.0	KKI
LH_Hippocampus	ICC2	0.950	68.94	20	20	2.93E-14	0.777	0.982	v6.0 vs. v7.1	KKI
LH_Hippocampus	ICC3	0.937	30.66	20	20	7.02E-11	0.870	0.970	v7.1	KKI
LH_inferiorparietal_surfavg	ICC3	0.983	119.91	20	20	1.29E-16	0.965	0.992	v5.3	KKI
LH_inferiorparietal_surfavg	ICC2	0.889	35.44	20	20	1.78E-11	0.460	0.961	v5.3 vs. v6.0	KKI
LH_inferiorparietal_surfavg	ICC2	0.883	30.97	20	20	6.38E-11	0.502	0.957	v5.3 vs. v7.1	KKI
LH_inferiorparietal_surfavg	ICC3	0.995	434.23	20	20	3.72E-22	0.990	0.998	v6.0	KKI
LH_inferiorparietal_surfavg	ICC2	0.995	352.74	20	20	2.94E-21	0.989	0.997	v6.0 vs. v7.1	KKI
LH_inferiorparietal_surfavg	ICC3	0.993	296.66	20	20	1.65E-20	0.986	0.997	v7.1	KKI
LH_inferiorparietal_thickavg	ICC3	0.768	7.64	20	20	1.48E-05	0.565	0.884	v5.3	KKI
LH_inferiorparietal_thickavg	ICC2	0.813	13.29	20	20	1.44E-07	0.549	0.917	v5.3 vs. v6.0	KKI
LH_inferiorparietal_thickavg	ICC2	0.805	9.13	20	20	3.48E-06	0.630	0.903	v5.3 vs. v7.1	KKI
LH_inferiorparietal_thickavg	ICC3	0.825	10.44	20	20	1.14E-06	0.662	0.914	v6.0	KKI
LH_inferiorparietal_thickavg	ICC2	0.896	25.21	20	20	4.40E-10	0.720	0.955	v6.0 vs. v7.1	KKI
LH_inferiorparietal_thickavg	ICC3	0.872	14.66	20	20	6.08E-08	0.747	0.938	v7.1	KKI
LH_inferiortemporal_surfavg	ICC3	0.984	126.97	20	20	7.35E-17	0.967	0.993	v5.3	KKI
LH_inferiortemporal_surfavg	ICC2	0.942	44.45	20	20	2.05E-12	0.845	0.975	v5.3 vs. v6.0	KKI
LH_inferiortemporal_surfavg	ICC2	0.914	36.44	20	20	1.37E-11	0.686	0.967	v5.3 vs. v7.1	KKI
LH_inferiortemporal_surfavg	ICC3	0.986	142.95	20	20	2.28E-17	0.971	0.993	v6.0	KKI
LH_inferiortemporal_surfavg	ICC2	0.986	217.13	20	20	3.65E-19	0.953	0.995	v6.0 vs. v7.1	KKI
LH_inferiortemporal_surfavg	ICC3	0.980	99.70	20	20	7.94E-16	0.958	0.991	v7.1	KKI
LH_inferiortemporal_thickavg	ICC3	0.902	19.44	20	20	4.81E-09	0.803	0.953	v5.3	KKI
LH_inferiortemporal_thickavg	ICC2	0.923	29.32	20	20	1.07E-10	0.828	0.964	v5.3 vs. v6.0	KKI
LH_inferiortemporal_thickavg	ICC2	0.876	26.75	20	20	2.53E-10	0.535	0.953	v5.3 vs. v7.1	KKI

LH_inferiortemporal_thickavg	ICC3	0.836	11.16	20	20	6.49E-07	0.680	0.919	v6.0	KKI
LH_inferiortemporal_thickavg	ICC2	0.934	38.68	20	20	7.74E-12	0.830	0.971	v6.0 vs. v7.1	KKI
LH_inferiortemporal_thickavg	ICC3	0.890	17.24	20	20	1.43E-08	0.781	0.947	v7.1	KKI
LH_insula_surfavg	ICC3	0.724	6.24	20	20	6.99E-05	0.492	0.860	v5.3	KKI
LH_insula_surfavg	ICC2	0.386	6.59	20	20	4.63E-05	-0.068	0.706	v5.3 vs. v6.0	KKI
LH_insula_surfavg	ICC2	0.370	7.39	20	20	1.92E-05	-0.063	0.697	v5.3 vs. v7.1	KKI
LH_insula_surfavg	ICC3	0.815	9.79	20	20	1.97E-06	0.643	0.908	v6.0	KKI
LH_insula_surfavg	ICC2	0.759	7.05	20	20	2.75E-05	0.548	0.879	v6.0 vs. v7.1	KKI
LH_insula_surfavg	ICC3	0.800	8.98	20	20	3.99E-06	0.618	0.900	v7.1	KKI
LH_insula_thickavg	ICC3	0.799	8.94	20	20	4.14E-06	0.616	0.900	v5.3	KKI
LH_insula_thickavg	ICC2	0.717	7.15	20	20	2.47E-05	0.458	0.859	v5.3 vs. v6.0	KKI
LH_insula_thickavg	ICC2	0.634	7.87	20	20	1.16E-05	0.124	0.840	v5.3 vs. v7.1	KKI
LH_insula_thickavg	ICC3	0.687	5.39	20	20	2.06E-04	0.435	0.839	v6.0	KKI
LH_insula_thickavg	ICC2	0.713	6.37	20	20	6.00E-05	0.480	0.853	v6.0 vs. v7.1	KKI
LH_insula_thickavg	ICC3	0.764	7.47	20	20	1.76E-05	0.557	0.881	v7.1	KKI
LH_isthmuscingulate_surfavg	ICC3	0.845	11.91	20	20	3.73E-07	0.697	0.924	v5.3	KKI
LH_isthmuscingulate_surfavg	ICC2	0.671	4.92	20	20	3.96E-04	0.408	0.831	v5.3 vs. v6.0	KKI
LH_isthmuscingulate_surfavg	ICC2	0.681	5.23	20	20	2.56E-04	0.429	0.835	v5.3 vs. v7.1	KKI
LH_isthmuscingulate_surfavg	ICC3	0.973	72.10	20	20	1.89E-14	0.943	0.987	v6.0	KKI
LH_isthmuscingulate_surfavg	ICC2	0.980	117.66	20	20	1.56E-16	0.954	0.991	v6.0 vs. v7.1	KKI
LH_isthmuscingulate_surfavg	ICC3	0.970	64.92	20	20	5.26E-14	0.937	0.986	v7.1	KKI
LH_isthmuscingulate_thickavg	ICC3	0.774	7.85	20	20	1.19E-05	0.574	0.887	v5.3	KKI
LH_isthmuscingulate_thickavg	ICC2	0.436	3.59	20	20	3.13E-03	0.048	0.698	v5.3 vs. v6.0	KKI
LH_isthmuscingulate_thickavg	ICC2	0.312	2.79	20	20	1.32E-02	-0.039	0.600	v5.3 vs. v7.1	KKI
LH_isthmuscingulate_thickavg	ICC3	0.794	8.71	20	20	5.12E-06	0.608	0.897	v6.0	KKI
LH_isthmuscingulate_thickavg	ICC2	0.761	9.02	20	20	3.84E-06	0.514	0.885	v6.0 vs. v7.1	KKI
LH_isthmuscingulate_thickavg	ICC3	0.797	8.83	20	20	4.58E-06	0.612	0.899	v7.1	KKI
LH_lateraloccipital_surfavg	ICC3	0.989	176.52	20	20	2.84E-18	0.976	0.995	v5.3	KKI
LH_lateraloccipital_surfavg	ICC2	0.849	23.61	20	20	8.07E-10	0.406	0.944	v5.3 vs. v6.0	KKI
LH_lateraloccipital_surfavg	ICC2	0.805	19.56	20	20	4.56E-09	0.257	0.929	v5.3 vs. v7.1	KKI
LH_lateraloccipital_surfavg	ICC3	0.992	257.35	20	20	6.76E-20	0.984	0.996	v6.0	KKI
LH_lateraloccipital_surfavg	ICC2	0.991	342.86	20	20	3.90E-21	0.965	0.997	v6.0 vs. v7.1	KKI
LH_lateraloccipital_surfavg	ICC3	0.990	197.10	20	20	9.52E-19	0.979	0.995	v7.1	KKI
LH_lateraloccipital_thickavg	ICC3	0.935	29.98	20	20	8.67E-11	0.868	0.969	v5.3	KKI
LH_lateraloccipital_thickavg	ICC2	0.832	35.68	20	20	1.67E-11	0.101	0.947	v5.3 vs. v6.0	KKI
LH_lateraloccipital_thickavg	ICC2	0.896	30.51	20	20	7.35E-11	0.628	0.960	v5.3 vs. v7.1	KKI
LH_lateraloccipital_thickavg	ICC3	0.874	14.90	20	20	5.28E-08	0.750	0.939	v6.0	KKI
LH_lateraloccipital_thickavg	ICC2	0.951	93.78	20	20	1.45E-15	0.644	0.984	v6.0 vs. v7.1	KKI
LH_lateraloccipital_thickavg	ICC3	0.908	20.73	20	20	2.68E-09	0.814	0.956	v7.1	KKI
LH_lateralorbitofrontal_surfavg	ICC3	0.961	49.79	20	20	6.88E-13	0.918	0.981	v5.3	KKI
LH_lateralorbitofrontal_surfavg	ICC2	0.929	59.60	20	20	1.21E-13	0.568	0.976	v5.3 vs. v6.0	KKI
LH_lateralorbitofrontal_surfavg	ICC2	0.810	69.80	20	20	2.60E-14	-0.001	0.945	v5.3 vs. v7.1	KKI
LH_lateralorbitofrontal_surfavg	ICC3	0.970	66.55	20	20	4.13E-14	0.938	0.986	v6.0	KKI
LH_lateralorbitofrontal_surfavg	ICC2	0.922	93.11	20	20	1.55E-15	0.243	0.977	v6.0 vs. v7.1	KKI

LH_lateralorbitofrontal_surfavg	ICC3	0.978	90.90	20	20	1.97E-15	0.954	0.990	v7.1	KKI
LH_lateralorbitofrontal_thickavg	ICC3	0.951	39.84	20	20	5.85E-12	0.899	0.977	v5.3	KKI
LH_lateralorbitofrontal_thickavg	ICC2	0.908	22.58	20	20	1.22E-09	0.811	0.956	v5.3 vs. v6.0	KKI
LH_lateralorbitofrontal_thickavg	ICC2	0.795	18.00	20	20	9.72E-09	0.253	0.924	v5.3 vs. v7.1	KKI
LH_lateralorbitofrontal_thickavg	ICC3	0.907	20.51	20	20	2.96E-09	0.812	0.955	v6.0	KKI
LH_lateralorbitofrontal_thickavg	ICC2	0.846	19.57	20	20	4.54E-09	0.510	0.938	v6.0 vs. v7.1	KKI
LH_lateralorbitofrontal_thickavg	ICC3	0.944	34.72	20	20	2.17E-11	0.885	0.973	v7.1	KKI
LH_LateralVentricle	ICC3	0.999	2616.87	20	20	6.09E-30	0.998	1.000	v5.3	KKI
LH_LateralVentricle	ICC2	0.994	2482.70	20	20	1.03E-29	0.670	0.998	v5.3 vs. v6.0	KKI
LH_LateralVentricle	ICC2	0.991	2339.77	20	20	1.86E-29	0.536	0.998	v5.3 vs. v7.1	KKI
LH_LateralVentricle	ICC3	1.000	4159.40	20	20	5.93E-32	0.999	1.000	v6.0	KKI
LH_LateralVentricle	ICC2	1.000	16464.35	20	20	6.30E-38	0.997	1.000	v6.0 vs. v7.1	KKI
LH_LateralVentricle	ICC3	1.000	4988.49	20	20	9.64E-33	0.999	1.000	v7.1	KKI
LH_lingual_surfavg	ICC3	0.965	55.69	20	20	2.33E-13	0.927	0.983	v5.3	KKI
LH_lingual_surfavg	ICC2	0.910	20.56	20	20	2.89E-09	0.819	0.957	v5.3 vs. v6.0	KKI
LH_lingual_surfavg	ICC2	0.904	20.66	20	20	2.76E-09	0.808	0.953	v5.3 vs. v7.1	KKI
LH_lingual_surfavg	ICC3	0.985	128.94	20	20	6.32E-17	0.968	0.993	v6.0	KKI
LH_lingual_surfavg	ICC2	0.989	227.15	20	20	2.33E-19	0.970	0.995	v6.0 vs. v7.1	KKI
LH_lingual_surfavg	ICC3	0.989	182.75	20	20	2.01E-18	0.977	0.995	v7.1	KKI
LH_lingual_thickavg	ICC3	0.948	37.54	20	20	1.03E-11	0.893	0.975	v5.3	KKI
LH_lingual_thickavg	ICC2	0.893	27.29	20	20	2.09E-10	0.657	0.957	v5.3 vs. v6.0	KKI
LH_lingual_thickavg	ICC2	0.926	24.97	20	20	4.80E-10	0.850	0.965	v5.3 vs. v7.1	KKI
LH_lingual_thickavg	ICC3	0.950	39.21	20	20	6.81E-12	0.897	0.976	v6.0	KKI
LH_lingual_thickavg	ICC2	0.930	90.13	20	20	2.14E-15	0.342	0.979	v6.0 vs. v7.1	KKI
LH_lingual_thickavg	ICC3	0.952	40.80	20	20	4.66E-12	0.901	0.977	v7.1	KKI
LH_medialorbitofrontal_surfavg	ICC3	0.786	8.32	20	20	7.41E-06	0.593	0.893	v5.3	KKI
LH_medialorbitofrontal_surfavg	ICC2	0.539	5.82	20	20	1.19E-04	0.045	0.785	v5.3 vs. v6.0	KKI
LH_medialorbitofrontal_surfavg	ICC2	0.593	13.46	20	20	1.29E-07	-0.046	0.845	v5.3 vs. v7.1	KKI
LH_medialorbitofrontal_surfavg	ICC3	0.857	12.97	20	20	1.78E-07	0.719	0.930	v6.0	KKI
LH_medialorbitofrontal_surfavg	ICC2	0.814	9.88	20	20	1.81E-06	0.646	0.907	v6.0 vs. v7.1	KKI
LH_medialorbitofrontal_surfavg	ICC3	0.870	14.35	20	20	7.36E-08	0.742	0.936	v7.1	KKI
LH_medialorbitofrontal_thickavg	ICC3	0.797	8.85	20	20	4.53E-06	0.613	0.899	v5.3	KKI
LH_medialorbitofrontal_thickavg	ICC2	0.873	14.06	20	20	8.82E-08	0.747	0.938	v5.3 vs. v6.0	KKI
LH_medialorbitofrontal_thickavg	ICC2	0.874	30.47	20	20	7.44E-11	0.427	0.955	v5.3 vs. v7.1	KKI
LH_medialorbitofrontal_thickavg	ICC3	0.903	19.71	20	20	4.25E-09	0.805	0.953	v6.0	KKI
LH_medialorbitofrontal_thickavg	ICC2	0.815	15.13	20	20	4.60E-08	0.478	0.922	v6.0 vs. v7.1	KKI
LH_medialorbitofrontal_thickavg	ICC3	0.889	17.04	20	20	1.59E-08	0.778	0.946	v7.1	KKI
LH_middletemporal_surfavg	ICC3	0.985	129.97	20	20	5.84E-17	0.968	0.993	v5.3	KKI
LH_middletemporal_surfavg	ICC2	0.965	68.28	20	20	3.22E-14	0.917	0.984	v5.3 vs. v6.0	KKI
LH_middletemporal_surfavg	ICC2	0.921	47.19	20	20	1.15E-12	0.618	0.972	v5.3 vs. v7.1	KKI
LH_middletemporal_surfavg	ICC3	0.987	148.00	20	20	1.62E-17	0.972	0.994	v6.0	KKI
LH_middletemporal_surfavg	ICC2	0.977	239.65	20	20	1.37E-19	0.733	0.993	v6.0 vs. v7.1	KKI
LH_middletemporal_surfavg	ICC3	0.993	279.90	20	20	2.93E-20	0.985	0.997	v7.1	KKI
LH_middletemporal_thickavg	ICC3	0.842	11.62	20	20	4.61E-07	0.691	0.922	v5.3	KKI

LH_middletemporal_thickavg	ICC2	0.854	13.65	20	20	1.14E-07	0.712	0.928	v5.3 vs. v6.0	KKI
LH_middletemporal_thickavg	ICC2	0.748	13.08	20	20	1.65E-07	0.227	0.900	v5.3 vs. v7.1	KKI
LH_middletemporal_thickavg	ICC3	0.870	14.40	20	20	7.12E-08	0.743	0.937	v6.0	KKI
LH_middletemporal_thickavg	ICC2	0.877	38.28	20	20	8.55E-12	0.307	0.959	v6.0 vs. v7.1	KKI
LH_middletemporal_thickavg	ICC3	0.874	14.92	20	20	5.22E-08	0.751	0.939	v7.1	KKI
LH_Pallidum	ICC3	0.792	8.61	20	20	5.66E-06	0.604	0.896	v5.3	KKI
LH_Pallidum	ICC2	0.192	2.98	20	20	9.17E-03	-0.070	0.481	v5.3 vs. v6.0	KKI
LH_Pallidum	ICC2	0.240	3.75	20	20	2.36E-03	-0.071	0.551	v5.3 vs. v7.1	KKI
LH_Pallidum	ICC3	0.940	32.16	20	20	4.48E-11	0.876	0.971	v6.0	KKI
LH_Pallidum	ICC2	0.893	16.96	20	20	1.66E-08	0.785	0.948	v6.0 vs. v7.1	KKI
LH_Pallidum	ICC3	0.943	34.12	20	20	2.56E-11	0.883	0.973	v7.1	KKI
LH_paracentral_surfavg	ICC3	0.623	4.30	20	20	9.84E-04	0.339	0.803	v5.3	KKI
LH_paracentral_surfavg	ICC2	0.671	4.95	20	20	3.80E-04	0.410	0.831	v5.3 vs. v6.0	KKI
LH_paracentral_surfavg	ICC2	0.634	4.61	20	20	6.21E-04	0.364	0.807	v5.3 vs. v7.1	KKI
LH_paracentral_surfavg	ICC3	0.913	22.07	20	20	1.51E-09	0.824	0.958	v6.0	KKI
LH_paracentral_surfavg	ICC2	0.885	18.34	20	20	8.18E-09	0.764	0.945	v6.0 vs. v7.1	KKI
LH_paracentral_surfavg	ICC3	0.942	33.28	20	20	3.23E-11	0.880	0.972	v7.1	KKI
LH_paracentral_thickavg	ICC3	0.882	15.98	20	20	2.82E-08	0.765	0.943	v5.3	KKI
LH_paracentral_thickavg	ICC2	0.892	26.05	20	20	3.24E-10	0.674	0.955	v5.3 vs. v6.0	KKI
LH_paracentral_thickavg	ICC2	0.858	14.13	20	20	8.44E-08	0.720	0.931	v5.3 vs. v7.1	KKI
LH_paracentral_thickavg	ICC3	0.801	9.06	20	20	3.71E-06	0.620	0.901	v6.0	KKI
LH_paracentral_thickavg	ICC2	0.914	22.80	20	20	1.12E-09	0.827	0.958	v6.0 vs. v7.1	KKI
LH_paracentral_thickavg	ICC3	0.858	13.06	20	20	1.68E-07	0.720	0.930	v7.1	KKI
LH_parahippocampal_surfavg	ICC3	0.840	11.52	20	20	4.98E-07	0.689	0.921	v5.3	KKI
LH_parahippocampal_surfavg	ICC2	0.652	5.64	20	20	1.49E-04	0.361	0.823	v5.3 vs. v6.0	KKI
LH_parahippocampal_surfavg	ICC2	0.504	3.18	20	20	6.46E-03	0.192	0.728	v5.3 vs. v7.1	KKI
LH_parahippocampal_surfavg	ICC3	0.866	13.96	20	20	9.39E-08	0.736	0.935	v6.0	KKI
LH_parahippocampal_surfavg	ICC2	0.879	15.12	20	20	4.63E-08	0.761	0.941	v6.0 vs. v7.1	KKI
LH_parahippocampal_surfavg	ICC3	0.879	15.58	20	20	3.54E-08	0.760	0.941	v7.1	KKI
LH_parahippocampal_thickavg	ICC3	0.896	18.18	20	20	8.88E-09	0.791	0.950	v5.3	KKI
LH_parahippocampal_thickavg	ICC2	0.947	37.11	20	20	1.15E-11	0.892	0.975	v5.3 vs. v6.0	KKI
LH_parahippocampal_thickavg	ICC2	0.815	24.25	20	20	6.30E-10	0.184	0.936	v5.3 vs. v7.1	KKI
LH_parahippocampal_thickavg	ICC3	0.927	26.43	20	20	2.83E-10	0.851	0.965	v6.0	KKI
LH_parahippocampal_thickavg	ICC2	0.866	48.42	20	20	8.99E-13	0.138	0.959	v6.0 vs. v7.1	KKI
LH_parahippocampal_thickavg	ICC3	0.946	36.29	20	20	1.42E-11	0.889	0.974	v7.1	KKI
LH_parsopercularis_surfavg	ICC3	0.973	73.70	20	20	1.53E-14	0.944	0.987	v5.3	KKI
LH_parsopercularis_surfavg	ICC2	0.903	33.56	20	20	2.99E-11	0.635	0.963	v5.3 vs. v6.0	KKI
LH_parsopercularis_surfavg	ICC2	0.882	16.64	20	20	1.96E-08	0.766	0.942	v5.3 vs. v7.1	KKI
LH_parsopercularis_surfavg	ICC3	0.845	11.87	20	20	3.84E-07	0.696	0.924	v6.0	KKI
LH_parsopercularis_surfavg	ICC2	0.837	11.39	20	20	5.47E-07	0.687	0.919	v6.0 vs. v7.1	KKI
LH_parsopercularis_surfavg	ICC3	0.878	15.46	20	20	3.81E-08	0.758	0.941	v7.1	KKI
LH_parsopercularis_thickavg	ICC3	0.862	13.46	20	20	1.29E-07	0.727	0.932	v5.3	KKI
LH_parsopercularis_thickavg	ICC2	0.801	10.35	20	20	1.23E-06	0.607	0.903	v5.3 vs. v6.0	KKI
LH_parsopercularis_thickavg	ICC2	0.842	11.70	20	20	4.35E-07	0.696	0.922	v5.3 vs. v7.1	KKI

LH_parsopercularis_thickavg	ICC3	0.836	11.21	20	20	6.26E-07	0.681	0.919	v6.0	KKI
LH_parsopercularis_thickavg	ICC2	0.933	34.03	20	20	2.62E-11	0.850	0.969	v6.0 vs. v7.1	KKI
LH_parsopercularis_thickavg	ICC3	0.873	14.76	20	20	5.74E-08	0.748	0.938	v7.1	KKI
LH_parsorbitalis_surfavg	ICC3	0.889	17.03	20	20	1.60E-08	0.778	0.946	v5.3	KKI
LH_parsorbitalis_surfavg	ICC2	0.610	14.22	20	20	7.95E-08	-0.043	0.854	v5.3 vs. v6.0	KKI
LH_parsorbitalis_surfavg	ICC2	0.508	13.50	20	20	1.26E-07	-0.052	0.802	v5.3 vs. v7.1	KKI
LH_parsorbitalis_surfavg	ICC3	0.964	54.06	20	20	3.10E-13	0.924	0.983	v6.0	KKI
LH_parsorbitalis_surfavg	ICC2	0.932	58.07	20	20	1.55E-13	0.633	0.977	v6.0 vs. v7.1	KKI
LH_parsorbitalis_surfavg	ICC3	0.981	104.98	20	20	4.78E-16	0.960	0.991	v7.1	KKI
LH_parsorbitalis_thickavg	ICC3	0.918	23.53	20	20	8.35E-10	0.834	0.961	v5.3	KKI
LH_parsorbitalis_thickavg	ICC2	0.848	11.62	20	20	4.61E-07	0.701	0.925	v5.3 vs. v6.0	KKI
LH_parsorbitalis_thickavg	ICC2	0.890	17.57	20	20	1.21E-08	0.783	0.947	v5.3 vs. v7.1	KKI
LH_parsorbitalis_thickavg	ICC3	0.893	17.66	20	20	1.15E-08	0.785	0.948	v6.0	KKI
LH_parsorbitalis_thickavg	ICC2	0.934	31.81	20	20	4.96E-11	0.864	0.969	v6.0 vs. v7.1	KKI
LH_parsorbitalis_thickavg	ICC3	0.924	25.23	20	20	4.37E-10	0.845	0.963	v7.1	KKI
LH_parstriangularis_surfavg	ICC3	0.977	84.63	20	20	3.96E-15	0.951	0.989	v5.3	KKI
LH_parstriangularis_surfavg	ICC2	0.892	25.73	20	20	3.63E-10	0.683	0.955	v5.3 vs. v6.0	KKI
LH_parstriangularis_surfavg	ICC2	0.750	10.34	20	20	1.24E-06	0.389	0.889	v5.3 vs. v7.1	KKI
LH_parstriangularis_surfavg	ICC3	0.941	32.86	20	20	3.65E-11	0.879	0.972	v6.0	KKI
LH_parstriangularis_surfavg	ICC2	0.915	26.71	20	20	2.57E-10	0.811	0.961	v6.0 vs. v7.1	KKI
LH_parstriangularis_surfavg	ICC3	0.909	20.93	20	20	2.45E-09	0.816	0.956	v7.1	KKI
LH_parstriangularis_thickavg	ICC3	0.968	62.02	20	20	8.21E-14	0.934	0.985	v5.3	KKI
LH_parstriangularis_thickavg	ICC2	0.903	24.83	20	20	5.06E-10	0.766	0.956	v5.3 vs. v6.0	KKI
LH_parstriangularis_thickavg	ICC2	0.873	15.11	20	20	4.65E-08	0.751	0.938	v5.3 vs. v7.1	KKI
LH_parstriangularis_thickavg	ICC3	0.943	34.01	20	20	2.64E-11	0.882	0.973	v6.0	KKI
LH_parstriangularis_thickavg	ICC2	0.957	49.97	20	20	6.64E-13	0.909	0.980	v6.0 vs. v7.1	KKI
LH_parstriangularis_thickavg	ICC3	0.903	19.58	20	20	4.52E-09	0.804	0.953	v7.1	KKI
LH_pericalcarine_surfavg	ICC3	0.966	57.72	20	20	1.65E-13	0.929	0.984	v5.3	KKI
LH_pericalcarine_surfavg	ICC2	0.908	28.78	20	20	1.27E-10	0.745	0.961	v5.3 vs. v6.0	KKI
LH_pericalcarine_surfavg	ICC2	0.924	36.94	20	20	1.20E-11	0.768	0.969	v5.3 vs. v7.1	KKI
LH_pericalcarine_surfavg	ICC3	0.983	116.96	20	20	1.65E-16	0.964	0.992	v6.0	KKI
LH_pericalcarine_surfavg	ICC2	0.992	247.53	20	20	9.94E-20	0.984	0.996	v6.0 vs. v7.1	KKI
LH_pericalcarine_surfavg	ICC3	0.991	221.02	20	20	3.06E-19	0.981	0.996	v7.1	KKI
LH_pericalcarine_thickavg	ICC3	0.887	16.72	20	20	1.88E-08	0.775	0.945	v5.3	KKI
LH_pericalcarine_thickavg	ICC2	0.806	21.25	20	20	2.13E-09	0.213	0.931	v5.3 vs. v6.0	KKI
LH_pericalcarine_thickavg	ICC2	0.858	17.28	20	20	1.40E-08	0.658	0.937	v5.3 vs. v7.1	KKI
LH_pericalcarine_thickavg	ICC3	0.886	16.62	20	20	1.99E-08	0.773	0.945	v6.0	KKI
LH_pericalcarine_thickavg	ICC2	0.938	46.69	20	20	1.28E-12	0.802	0.975	v6.0 vs. v7.1	KKI
LH_pericalcarine_thickavg	ICC3	0.928	26.81	20	20	2.48E-10	0.853	0.965	v7.1	KKI
LH_postcentral_surfavg	ICC3	0.663	4.93	20	20	3.87E-04	0.398	0.826	v5.3	KKI
LH_postcentral_surfavg	ICC2	0.532	3.24	20	20	5.75E-03	0.215	0.748	v5.3 vs. v6.0	KKI
LH_postcentral_surfavg	ICC2	0.963	51.05	20	20	5.41E-13	0.923	0.982	v5.3 vs. v7.1	KKI
LH_postcentral_surfavg	ICC3	0.483	2.87	20	20	1.13E-02	0.149	0.718	v6.0	KKI
LH_postcentral_surfavg	ICC2	0.424	2.45	20	20	2.59E-02	0.076	0.680	v6.0 vs. v7.1	KKI

LH_postcentral_surfavg	ICC3	0.347	2.06	20	20	5.67E-02	-0.015	0.628	v7.1	KKI
LH_postcentral_thickavg	ICC3	0.936	30.16	20	20	8.21E-11	0.868	0.969	v5.3	KKI
LH_postcentral_thickavg	ICC2	0.835	22.96	20	20	1.04E-09	0.331	0.940	v5.3 vs. v6.0	KKI
LH_postcentral_thickavg	ICC2	0.898	35.32	20	20	1.84E-11	0.549	0.963	v5.3 vs. v7.1	KKI
LH_postcentral_thickavg	ICC3	0.951	39.43	20	20	6.45E-12	0.898	0.976	v6.0	KKI
LH_postcentral_thickavg	ICC2	0.950	43.37	20	20	2.59E-12	0.893	0.976	v6.0 vs. v7.1	KKI
LH_postcentral_thickavg	ICC3	0.950	39.13	20	20	6.94E-12	0.897	0.976	v7.1	KKI
LH_posteriorcingulate_surfavg	ICC3	0.926	25.90	20	20	3.41E-10	0.848	0.964	v5.3	KKI
LH_posteriorcingulate_surfavg	ICC2	0.780	7.80	20	20	1.25E-05	0.583	0.890	v5.3 vs. v6.0	KKI
LH_posteriorcingulate_surfavg	ICC2	0.780	8.01	20	20	1.01E-05	0.588	0.890	v5.3 vs. v7.1	KKI
LH_posteriorcingulate_surfavg	ICC3	0.981	106.55	20	20	4.13E-16	0.961	0.991	v6.0	KKI
LH_posteriorcingulate_surfavg	ICC2	0.970	135.58	20	20	3.85E-17	0.813	0.990	v6.0 vs. v7.1	KKI
LH_posteriorcingulate_surfavg	ICC3	0.984	123.16	20	20	9.93E-17	0.966	0.992	v7.1	KKI
LH_posteriorcingulate_thickavg	ICC3	0.911	21.49	20	20	1.93E-09	0.820	0.957	v5.3	KKI
LH_posteriorcingulate_thickavg	ICC2	0.686	10.05	20	20	1.57E-06	0.149	0.870	v5.3 vs. v6.0	KKI
LH_posteriorcingulate_thickavg	ICC2	0.401	9.80	20	20	1.94E-06	-0.055	0.727	v5.3 vs. v7.1	KKI
LH_posteriorcingulate_thickavg	ICC3	0.831	10.80	20	20	8.56E-07	0.671	0.916	v6.0	KKI
LH_posteriorcingulate_thickavg	ICC2	0.651	17.96	20	20	9.87E-09	-0.036	0.876	v6.0 vs. v7.1	KKI
LH_posteriorcingulate_thickavg	ICC3	0.902	19.40	20	20	4.92E-09	0.803	0.953	v7.1	KKI
LH_precentral_surfavg	ICC3	0.973	72.52	20	20	1.79E-14	0.943	0.987	v5.3	KKI
LH_precentral_surfavg	ICC2	0.967	57.15	20	20	1.81E-13	0.931	0.984	v5.3 vs. v6.0	KKI
LH_precentral_surfavg	ICC2	0.665	4.95	20	20	3.77E-04	0.406	0.826	v5.3 vs. v7.1	KKI
LH_precentral_surfavg	ICC3	0.710	5.90	20	20	1.07E-04	0.471	0.852	v6.0	KKI
LH_precentral_surfavg	ICC2	0.696	5.68	20	20	1.41E-04	0.456	0.844	v6.0 vs. v7.1	KKI
LH_precentral_surfavg	ICC3	0.741	6.73	20	20	3.96E-05	0.520	0.869	v7.1	KKI
LH_precentral_thickavg	ICC3	0.798	8.91	20	20	4.27E-06	0.615	0.900	v5.3	KKI
LH_precentral_thickavg	ICC2	0.740	13.81	20	20	1.03E-07	0.167	0.900	v5.3 vs. v6.0	KKI
LH_precentral_thickavg	ICC2	0.883	17.20	20	20	1.46E-08	0.768	0.943	v5.3 vs. v7.1	KKI
LH_precentral_thickavg	ICC3	0.636	4.50	20	20	7.29E-04	0.358	0.811	v6.0	KKI
LH_precentral_thickavg	ICC2	0.621	4.71	20	20	5.33E-04	0.340	0.801	v6.0 vs. v7.1	KKI
LH_precentral_thickavg	ICC3	0.580	3.76	20	20	2.35E-03	0.278	0.777	v7.1	KKI
LH_precuneus_surfavg	ICC3	0.952	40.41	20	20	5.11E-12	0.900	0.977	v5.3	KKI
LH_precuneus_surfavg	ICC2	0.924	24.64	20	20	5.44E-10	0.847	0.964	v5.3 vs. v6.0	KKI
LH_precuneus_surfavg	ICC2	0.922	24.16	20	20	6.52E-10	0.842	0.962	v5.3 vs. v7.1	KKI
LH_precuneus_surfavg	ICC3	0.994	360.53	20	20	2.37E-21	0.988	0.997	v6.0	KKI
LH_precuneus_surfavg	ICC2	0.995	374.74	20	20	1.61E-21	0.989	0.997	v6.0 vs. v7.1	KKI
LH_precuneus_surfavg	ICC3	0.993	282.78	20	20	2.65E-20	0.985	0.997	v7.1	KKI
LH_precuneus_thickavg	ICC3	0.891	17.36	20	20	1.35E-08	0.782	0.947	v5.3	KKI
LH_precuneus_thickavg	ICC2	0.937	43.24	20	20	2.67E-12	0.814	0.974	v5.3 vs. v6.0	KKI
LH_precuneus_thickavg	ICC2	0.957	43.68	20	20	2.42E-12	0.911	0.979	v5.3 vs. v7.1	KKI
LH_precuneus_thickavg	ICC3	0.887	16.62	20	20	1.99E-08	0.773	0.945	v6.0	KKI
LH_precuneus_thickavg	ICC2	0.955	120.66	20	20	1.22E-16	0.567	0.986	v6.0 vs. v7.1	KKI
LH_precuneus_thickavg	ICC3	0.882	15.94	20	20	2.90E-08	0.765	0.943	v7.1	KKI
LH_Putamen	ICC3	0.853	12.63	20	20	2.25E-07	0.712	0.928	v5.3	KKI

LH_Putamen	ICC2	0.347	9.14	20	20	3.46E-06	-0.050	0.683	v5.3 vs. v6.0	KKI
LH_Putamen	ICC2	0.349	8.13	20	20	8.99E-06	-0.056	0.682	v5.3 vs. v7.1	KKI
LH_Putamen	ICC3	0.993	293.74	20	20	1.82E-20	0.986	0.997	v6.0	KKI
LH_Putamen	ICC2	0.981	124.33	20	20	9.05E-17	0.955	0.991	v6.0 vs. v7.1	KKI
LH_Putamen	ICC3	0.987	152.27	20	20	1.22E-17	0.972	0.994	v7.1	KKI
LH_rostralanteriorcingulate_surfavg	ICC3	0.942	33.34	20	20	3.18E-11	0.880	0.972	v5.3	KKI
LH_rostralanteriorcingulate_surfavg	ICC2	0.834	11.86	20	20	3.86E-07	0.677	0.918	v5.3 vs. v6.0	KKI
LH_rostralanteriorcingulate_surfavg	ICC2	0.781	8.24	20	20	8.03E-06	0.591	0.890	v5.3 vs. v7.1	KKI
LH_rostralanteriorcingulate_surfavg	ICC3	0.929	27.18	20	20	2.18E-10	0.855	0.966	v6.0	KKI
LH_rostralanteriorcingulate_surfavg	ICC2	0.893	37.90	20	20	9.41E-12	0.458	0.963	v6.0 vs. v7.1	KKI
LH_rostralanteriorcingulate_surfavg	ICC3	0.908	20.65	20	20	2.77E-09	0.813	0.955	v7.1	KKI
LH_rostralanteriorcingulate_thickavg	ICC3	0.910	21.22	20	20	2.16E-09	0.818	0.957	v5.3	KKI
LH_rostralanteriorcingulate_thickavg	ICC2	0.880	23.99	20	20	6.96E-10	0.634	0.951	v5.3 vs. v6.0	KKI
LH_rostralanteriorcingulate_thickavg	ICC2	0.380	7.08	20	20	2.67E-05	-0.066	0.704	v5.3 vs. v7.1	KKI
LH_rostralanteriorcingulate_thickavg	ICC3	0.816	9.89	20	20	1.80E-06	0.646	0.909	v6.0	KKI
LH_rostralanteriorcingulate_thickavg	ICC2	0.539	11.98	20	20	3.55E-07	-0.055	0.816	v6.0 vs. v7.1	KKI
LH_rostralanteriorcingulate_thickavg	ICC3	0.825	10.42	20	20	1.16E-06	0.661	0.914	v7.1	KKI
LH_rostralmiddlefrontal_surfavg	ICC3	0.963	52.48	20	20	4.14E-13	0.922	0.982	v5.3	KKI
LH_rostralmiddlefrontal_surfavg	ICC2	0.942	31.74	20	20	5.07E-11	0.880	0.972	v5.3 vs. v6.0	KKI
LH_rostralmiddlefrontal_surfavg	ICC2	0.870	14.19	20	20	8.12E-08	0.744	0.936	v5.3 vs. v7.1	KKI
LH_rostralmiddlefrontal_surfavg	ICC3	0.882	16.02	20	20	2.77E-08	0.766	0.943	v6.0	KKI
LH_rostralmiddlefrontal_surfavg	ICC2	0.856	12.77	20	20	2.05E-07	0.720	0.929	v6.0 vs. v7.1	KKI
LH_rostralmiddlefrontal_surfavg	ICC3	0.848	12.20	20	20	3.04E-07	0.703	0.926	v7.1	KKI
LH_rostralmiddlefrontal_thickavg	ICC3	0.795	8.78	20	20	4.82E-06	0.610	0.898	v5.3	KKI
LH_rostralmiddlefrontal_thickavg	ICC2	0.833	19.53	20	20	4.62E-09	0.426	0.935	v5.3 vs. v6.0	KKI
LH_rostralmiddlefrontal_thickavg	ICC2	0.815	15.14	20	20	4.58E-08	0.479	0.922	v5.3 vs. v7.1	KKI
LH_rostralmiddlefrontal_thickavg	ICC3	0.815	9.80	20	20	1.95E-06	0.644	0.908	v6.0	KKI
LH_rostralmiddlefrontal_thickavg	ICC2	0.939	30.64	20	20	7.08E-11	0.875	0.971	v6.0 vs. v7.1	KKI
LH_rostralmiddlefrontal_thickavg	ICC3	0.797	8.84	20	20	4.54E-06	0.613	0.899	v7.1	KKI
LH_superiorfrontal_surfavg	ICC3	0.986	142.02	20	20	2.44E-17	0.971	0.993	v5.3	KKI
LH_superiorfrontal_surfavg	ICC2	0.942	68.36	20	20	3.18E-14	0.667	0.980	v5.3 vs. v6.0	KKI
LH_superiorfrontal_surfavg	ICC2	0.858	39.90	20	20	5.76E-12	0.169	0.955	v5.3 vs. v7.1	KKI
LH_superiorfrontal_surfavg	ICC3	0.922	24.73	20	20	5.25E-10	0.842	0.963	v6.0	KKI
LH_superiorfrontal_surfavg	ICC2	0.922	32.00	20	20	4.70E-11	0.801	0.966	v6.0 vs. v7.1	KKI
LH_superiorfrontal_surfavg	ICC3	0.949	38.08	20	20	8.99E-12	0.894	0.976	v7.1	KKI
LH_superiorfrontal_thickavg	ICC3	0.826	10.49	20	20	1.10E-06	0.663	0.914	v5.3	KKI
LH_superiorfrontal_thickavg	ICC2	0.939	31.99	20	20	4.71E-11	0.876	0.971	v5.3 vs. v6.0	KKI
LH_superiorfrontal_thickavg	ICC2	0.939	31.71	20	20	5.11E-11	0.875	0.970	v5.3 vs. v7.1	KKI
LH_superiorfrontal_thickavg	ICC3	0.848	12.13	20	20	3.19E-07	0.702	0.925	v6.0	KKI
LH_superiorfrontal_thickavg	ICC2	0.958	61.94	20	20	8.30E-14	0.885	0.982	v6.0 vs. v7.1	KKI
LH_superiorfrontal_thickavg	ICC3	0.903	19.72	20	20	4.23E-09	0.806	0.953	v7.1	KKI
LH_superiorparietal_surfavg	ICC3	0.996	472.47	20	20	1.60E-22	0.991	0.998	v5.3	KKI
LH_superiorparietal_surfavg	ICC2	0.968	68.16	20	20	3.28E-14	0.930	0.985	v5.3 vs. v6.0	KKI
LH_superiorparietal_surfavg	ICC2	0.972	81.21	20	20	5.93E-15	0.938	0.987	v5.3 vs. v7.1	KKI

LH_superiorparietal_surfavg	ICC3	0.990	203.47	20	20	6.95E-19	0.979	0.995	v6.0	KKI
LH_superiorparietal_surfavg	ICC2	0.997	632.59	20	20	8.75E-24	0.994	0.999	v6.0 vs. v7.1	KKI
LH_superiorparietal_surfavg	ICC3	0.993	289.90	20	20	2.07E-20	0.985	0.997	v7.1	KKI
LH_superiorparietal_thickavg	ICC3	0.863	13.55	20	20	1.21E-07	0.729	0.933	v5.3	KKI
LH_superiorparietal_thickavg	ICC2	0.890	41.31	20	20	4.13E-12	0.377	0.963	v5.3 vs. v6.0	KKI
LH_superiorparietal_thickavg	ICC2	0.916	43.56	20	20	2.48E-12	0.606	0.970	v5.3 vs. v7.1	KKI
LH_superiorparietal_thickavg	ICC3	0.874	14.83	20	20	5.48E-08	0.749	0.938	v6.0	KKI
LH_superiorparietal_thickavg	ICC2	0.969	70.25	20	20	2.44E-14	0.933	0.985	v6.0 vs. v7.1	KKI
LH_superiorparietal_thickavg	ICC3	0.917	23.08	20	20	9.96E-10	0.831	0.960	v7.1	KKI
LH_superiortemporal_surfavg	ICC3	0.984	127.13	20	20	7.26E-17	0.967	0.993	v5.3	KKI
LH_superiortemporal_surfavg	ICC2	0.845	55.79	20	20	2.29E-13	0.044	0.954	v5.3 vs. v6.0	KKI
LH_superiortemporal_surfavg	ICC2	0.788	63.65	20	20	6.38E-14	-0.007	0.937	v5.3 vs. v7.1	KKI
LH_superiortemporal_surfavg	ICC3	0.992	252.48	20	20	8.17E-20	0.983	0.996	v6.0	KKI
LH_superiortemporal_surfavg	ICC2	0.981	206.07	20	20	6.13E-19	0.888	0.994	v6.0 vs. v7.1	KKI
LH_superiortemporal_surfavg	ICC3	0.994	356.54	20	20	2.64E-21	0.988	0.997	v7.1	KKI
LH_superiortemporal_thickavg	ICC3	0.969	62.73	20	20	7.35E-14	0.934	0.985	v5.3	KKI
LH_superiortemporal_thickavg	ICC2	0.979	97.50	20	20	9.88E-16	0.957	0.990	v5.3 vs. v6.0	KKI
LH_superiortemporal_thickavg	ICC2	0.953	73.15	20	20	1.65E-14	0.789	0.983	v5.3 vs. v7.1	KKI
LH_superiortemporal_thickavg	ICC3	0.959	48.18	20	20	9.44E-13	0.916	0.981	v6.0	KKI
LH_superiortemporal_thickavg	ICC2	0.965	84.03	20	20	4.24E-15	0.884	0.986	v6.0 vs. v7.1	KKI
LH_superiortemporal_thickavg	ICC3	0.953	41.49	20	20	3.97E-12	0.903	0.978	v7.1	KKI
LH_supramarginal_surfavg	ICC3	0.947	36.50	20	20	1.35E-11	0.890	0.975	v5.3	KKI
LH_supramarginal_surfavg	ICC2	0.776	16.25	20	20	2.43E-08	0.225	0.916	v5.3 vs. v6.0	KKI
LH_supramarginal_surfavg	ICC2	0.776	19.44	20	20	4.83E-09	0.131	0.920	v5.3 vs. v7.1	KKI
LH_supramarginal_surfavg	ICC3	0.920	23.94	20	20	7.10E-10	0.837	0.961	v6.0	KKI
LH_supramarginal_surfavg	ICC2	0.973	69.66	20	20	2.65E-14	0.943	0.987	v6.0 vs. v7.1	KKI
LH_supramarginal_surfavg	ICC3	0.967	59.60	20	20	1.21E-13	0.931	0.984	v7.1	KKI
LH_supramarginal_thickavg	ICC3	0.835	11.13	20	20	6.65E-07	0.680	0.919	v5.3	KKI
LH_supramarginal_thickavg	ICC2	0.873	17.25	20	20	1.42E-08	0.730	0.940	v5.3 vs. v6.0	KKI
LH_supramarginal_thickavg	ICC2	0.793	8.33	20	20	7.35E-06	0.605	0.897	v5.3 vs. v7.1	KKI
LH_supramarginal_thickavg	ICC3	0.806	9.32	20	20	2.95E-06	0.629	0.904	v6.0	KKI
LH_supramarginal_thickavg	ICC2	0.872	17.06	20	20	1.57E-08	0.731	0.940	v6.0 vs. v7.1	KKI
LH_supramarginal_thickavg	ICC3	0.840	11.47	20	20	5.15E-07	0.687	0.921	v7.1	KKI
LH_SurfArea	ICC3	0.995	428.27	20	20	4.27E-22	0.990	0.998	v5.3	KKI
LH_SurfArea	ICC2	0.981	1190.32	20	20	1.59E-26	0.296	0.995	v5.3 vs. v6.0	KKI
LH_SurfArea	ICC2	0.954	541.96	20	20	4.09E-23	0.123	0.989	v5.3 vs. v7.1	KKI
LH_SurfArea	ICC3	0.996	485.35	20	20	1.23E-22	0.991	0.998	v6.0	KKI
LH_SurfArea	ICC2	0.993	1571.64	20	20	9.93E-28	0.720	0.998	v6.0 vs. v7.1	KKI
LH_SurfArea	ICC3	0.997	660.51	20	20	5.69E-24	0.994	0.999	v7.1	KKI
LH_temporalpole_surfavg	ICC3	0.710	5.91	20	20	1.06E-04	0.471	0.852	v5.3	KKI
LH_temporalpole_surfavg	ICC2	0.587	4.99	20	20	3.58E-04	0.235	0.790	v5.3 vs. v6.0	KKI
LH_temporalpole_surfavg	ICC2	0.418	3.75	20	20	2.40E-03	0.005	0.692	v5.3 vs. v7.1	KKI
LH_temporalpole_surfavg	ICC3	0.802	9.09	20	20	3.62E-06	0.621	0.902	v6.0	KKI
LH_temporalpole_surfavg	ICC2	0.765	11.75	20	20	4.20E-07	0.375	0.900	v6.0 vs. v7.1	KKI

LH_temporalpole_surfavg	ICC3	0.827	10.54	20	20	1.05E-06	0.665	0.915	v7.1	KKI
LH_temporalpole_thickavg	ICC3	0.690	5.46	20	20	1.89E-04	0.440	0.841	v5.3	KKI
LH_temporalpole_thickavg	ICC2	0.724	6.35	20	20	6.12E-05	0.498	0.859	v5.3 vs. v6.0	KKI
LH_temporalpole_thickavg	ICC2	0.701	10.82	20	20	8.49E-07	0.161	0.878	v5.3 vs. v7.1	KKI
LH_temporalpole_thickavg	ICC3	0.675	5.16	20	20	2.82E-04	0.417	0.833	v6.0	KKI
LH_temporalpole_thickavg	ICC2	0.635	6.19	20	20	7.44E-05	0.260	0.824	v6.0 vs. v7.1	KKI
LH_temporalpole_thickavg	ICC3	0.433	2.53	20	20	2.21E-02	0.087	0.686	v7.1	KKI
LH_Thalamus	ICC3	0.883	16.04	20	20	2.73E-08	0.766	0.943	v5.3	KKI
LH_Thalamus	ICC2	0.725	11.03	20	20	7.20E-07	0.238	0.886	v5.3 vs. v6.0	KKI
LH_Thalamus	ICC2	0.701	6.21	20	20	7.28E-05	0.458	0.847	v5.3 vs. v7.1	KKI
LH_Thalamus	ICC3	0.938	31.35	20	20	5.70E-11	0.873	0.970	v6.0	KKI
LH_Thalamus	ICC2	0.865	18.17	20	20	8.90E-09	0.673	0.940	v6.0 vs. v7.1	KKI
LH_Thalamus	ICC3	0.956	44.77	20	20	1.91E-12	0.909	0.979	v7.1	KKI
LH_Thickness	ICC3	0.925	25.73	20	20	3.64E-10	0.847	0.964	v5.3	KKI
LH_Thickness	ICC2	0.930	45.91	20	20	1.50E-12	0.734	0.973	v5.3 vs. v6.0	KKI
LH_Thickness	ICC2	0.946	35.51	20	20	1.75E-11	0.889	0.974	v5.3 vs. v7.1	KKI
LH_Thickness	ICC3	0.913	21.90	20	20	1.62E-09	0.823	0.958	v6.0	KKI
LH_Thickness	ICC2	0.923	76.70	20	20	1.04E-14	0.343	0.977	v6.0 vs. v7.1	KKI
LH_Thickness	ICC3	0.926	26.04	20	20	3.25E-10	0.849	0.964	v7.1	KKI
LH_transversetemporal_surfavg	ICC3	0.950	38.96	20	20	7.24E-12	0.897	0.976	v5.3	KKI
LH_transversetemporal_surfavg	ICC2	0.914	37.87	20	20	9.49E-12	0.667	0.967	v5.3 vs. v6.0	KKI
LH_transversetemporal_surfavg	ICC2	0.924	27.75	20	20	1.80E-10	0.843	0.964	v5.3 vs. v7.1	KKI
LH_transversetemporal_surfavg	ICC3	0.962	51.51	20	20	4.95E-13	0.921	0.982	v6.0	KKI
LH_transversetemporal_surfavg	ICC2	0.953	54.99	20	20	2.63E-13	0.875	0.980	v6.0 vs. v7.1	KKI
LH_transversetemporal_surfavg	ICC3	0.979	95.83	20	20	1.17E-15	0.957	0.990	v7.1	KKI
LH_transversetemporal_thickavg	ICC3	0.907	20.61	20	20	2.83E-09	0.813	0.955	v5.3	KKI
LH_transversetemporal_thickavg	ICC2	0.847	32.34	20	20	4.25E-11	0.207	0.949	v5.3 vs. v6.0	KKI
LH_transversetemporal_thickavg	ICC2	0.827	30.37	20	20	7.69E-11	0.137	0.943	v5.3 vs. v7.1	KKI
LH_transversetemporal_thickavg	ICC3	0.816	9.87	20	20	1.84E-06	0.646	0.909	v6.0	KKI
LH_transversetemporal_thickavg	ICC2	0.954	40.73	20	20	4.73E-12	0.905	0.978	v6.0 vs. v7.1	KKI
LH_transversetemporal_thickavg	ICC3	0.803	9.16	20	20	3.40E-06	0.624	0.902	v7.1	KKI
RH_Accumbens	ICC3	0.825	10.41	20	20	1.17E-06	0.661	0.913	v5.3	KKI
RH_Accumbens	ICC2	0.484	11.48	20	20	5.12E-07	-0.056	0.785	v5.3 vs. v6.0	KKI
RH_Accumbens	ICC2	0.673	10.67	20	20	9.52E-07	0.084	0.869	v5.3 vs. v7.1	KKI
RH_Accumbens	ICC3	0.859	13.14	20	20	1.59E-07	0.722	0.931	v6.0	KKI
RH_Accumbens	ICC2	0.792	12.57	20	20	2.34E-07	0.473	0.909	v6.0 vs. v7.1	KKI
RH_Accumbens	ICC3	0.813	9.69	20	20	2.13E-06	0.640	0.907	v7.1	KKI
RH_Amygdala	ICC3	0.906	20.36	20	20	3.17E-09	0.811	0.955	v5.3	KKI
RH_Amygdala	ICC2	0.651	9.37	20	20	2.81E-06	0.085	0.855	v5.3 vs. v6.0	KKI
RH_Amygdala	ICC2	0.591	7.87	20	20	1.17E-05	0.030	0.825	v5.3 vs. v7.1	KKI
RH_Amygdala	ICC3	0.924	25.45	20	20	4.02E-10	0.846	0.964	v6.0	KKI
RH_Amygdala	ICC2	0.923	25.51	20	20	3.94E-10	0.846	0.963	v6.0 vs. v7.1	KKI
RH_Amygdala	ICC3	0.906	20.34	20	20	3.18E-09	0.811	0.955	v7.1	KKI
RH_bankssts_surfavg	ICC3	0.853	12.58	20	20	2.33E-07	0.711	0.928	v5.3	KKI

RH_bankssts_surfavg	ICC2	0.873	36.67	20	20	1.29E-11	0.306	0.958	v5.3 vs. v6.0	KKI
RH_bankssts_surfavg	ICC2	0.841	28.19	20	20	1.55E-10	0.245	0.946	v5.3 vs. v7.1	KKI
RH_bankssts_surfavg	ICC3	0.985	135.28	20	20	3.94E-17	0.969	0.993	v6.0	KKI
RH_bankssts_surfavg	ICC2	0.980	95.97	20	20	1.16E-15	0.958	0.990	v6.0 vs. v7.1	KKI
RH_bankssts_surfavg	ICC3	0.963	53.52	20	20	3.42E-13	0.924	0.983	v7.1	KKI
RH_bankssts_thickavg	ICC3	0.885	16.36	20	20	2.30E-08	0.770	0.944	v5.3	KKI
RH_bankssts_thickavg	ICC2	0.918	23.30	20	20	9.14E-10	0.836	0.961	v5.3 vs. v6.0	KKI
RH_bankssts_thickavg	ICC2	0.903	20.07	20	20	3.61E-09	0.806	0.953	v5.3 vs. v7.1	KKI
RH_bankssts_thickavg	ICC3	0.942	33.26	20	20	3.26E-11	0.880	0.972	v6.0	KKI
RH_bankssts_thickavg	ICC2	0.934	39.82	20	20	5.88E-12	0.820	0.972	v6.0 vs. v7.1	KKI
RH_bankssts_thickavg	ICC3	0.905	19.97	20	20	3.78E-09	0.808	0.954	v7.1	KKI
RH_caudalanteriorcingulate_surfavg	ICC3	0.969	62.73	20	20	7.35E-14	0.934	0.985	v5.3	KKI
RH_caudalanteriorcingulate_surfavg	ICC2	0.801	18.15	20	20	9.01E-09	0.282	0.925	v5.3 vs. v6.0	KKI
RH_caudalanteriorcingulate_surfavg	ICC2	0.834	12.36	20	20	2.71E-07	0.671	0.919	v5.3 vs. v7.1	KKI
RH_caudalanteriorcingulate_surfavg	ICC3	0.959	47.51	20	20	1.08E-12	0.914	0.980	v6.0	KKI
RH_caudalanteriorcingulate_surfavg	ICC2	0.931	60.67	20	20	1.02E-13	0.595	0.977	v6.0 vs. v7.1	KKI
RH_caudalanteriorcingulate_surfavg	ICC3	0.964	54.95	20	20	2.65E-13	0.926	0.983	v7.1	KKI
RH_caudalanteriorcingulate_thickavg	ICC3	0.945	35.48	20	20	1.76E-11	0.887	0.974	v5.3	KKI
RH_caudalanteriorcingulate_thickavg	ICC2	0.875	19.25	20	20	5.28E-09	0.706	0.944	v5.3 vs. v6.0	KKI
RH_caudalanteriorcingulate_thickavg	ICC2	0.506	7.22	20	20	2.30E-05	-0.044	0.782	v5.3 vs. v7.1	KKI
RH_caudalanteriorcingulate_thickavg	ICC3	0.919	23.78	20	20	7.55E-10	0.836	0.961	v6.0	KKI
RH_caudalanteriorcingulate_thickavg	ICC2	0.688	14.74	20	20	5.80E-08	0.015	0.885	v6.0 vs. v7.1	KKI
RH_caudalanteriorcingulate_thickavg	ICC3	0.887	16.76	20	20	1.85E-08	0.775	0.945	v7.1	KKI
RH_caudalmiddlefrontal_surfavg	ICC3	0.977	85.43	20	20	3.61E-15	0.951	0.989	v5.3	KKI
RH_caudalmiddlefrontal_surfavg	ICC2	0.801	8.77	20	20	4.87E-06	0.620	0.901	v5.3 vs. v6.0	KKI
RH_caudalmiddlefrontal_surfavg	ICC2	0.746	6.70	20	20	4.09E-05	0.529	0.872	v5.3 vs. v7.1	KKI
RH_caudalmiddlefrontal_surfavg	ICC3	0.906	20.20	20	20	3.40E-09	0.810	0.954	v6.0	KKI
RH_caudalmiddlefrontal_surfavg	ICC2	0.976	80.15	20	20	6.74E-15	0.950	0.989	v6.0 vs. v7.1	KKI
RH_caudalmiddlefrontal_surfavg	ICC3	0.987	152.48	20	20	1.21E-17	0.973	0.994	v7.1	KKI
RH_caudalmiddlefrontal_thickavg	ICC3	0.772	7.79	20	20	1.26E-05	0.572	0.886	v5.3	KKI
RH_caudalmiddlefrontal_thickavg	ICC2	0.857	24.46	20	20	5.82E-10	0.443	0.947	v5.3 vs. v6.0	KKI
RH_caudalmiddlefrontal_thickavg	ICC2	0.835	18.02	20	20	9.59E-09	0.493	0.933	v5.3 vs. v7.1	KKI
RH_caudalmiddlefrontal_thickavg	ICC3	0.866	13.89	20	20	9.79E-08	0.735	0.934	v6.0	KKI
RH_caudalmiddlefrontal_thickavg	ICC2	0.933	27.52	20	20	1.94E-10	0.862	0.968	v6.0 vs. v7.1	KKI
RH_caudalmiddlefrontal_thickavg	ICC3	0.836	11.17	20	20	6.45E-07	0.680	0.919	v7.1	KKI
RH_Caudate	ICC3	0.982	110.30	20	20	2.94E-16	0.962	0.991	v5.3	KKI
RH_Caudate	ICC2	0.823	274.64	20	20	3.54E-20	0.010	0.952	v5.3 vs. v6.0	KKI
RH_Caudate	ICC2	0.906	183.74	20	20	1.91E-18	0.053	0.975	v5.3 vs. v7.1	KKI
RH_Caudate	ICC3	0.993	276.45	20	20	3.32E-20	0.985	0.997	v6.0	KKI
RH_Caudate	ICC2	0.970	292.06	20	20	1.92E-20	0.444	0.992	v6.0 vs. v7.1	KKI
RH_Caudate	ICC3	0.989	186.03	20	20	1.69E-18	0.977	0.995	v7.1	KKI
RH_cuneus_surfavg	ICC3	0.946	35.71	20	20	1.66E-11	0.888	0.974	v5.3	KKI
RH_cuneus_surfavg	ICC2	0.763	9.74	20	20	2.05E-06	0.482	0.889	v5.3 vs. v6.0	KKI
RH_cuneus_surfavg	ICC2	0.713	9.81	20	20	1.93E-06	0.264	0.877	v5.3 vs. v7.1	KKI

RH_cuneus_surfavg	ICC3	0.982	108.17	20	20	3.56E-16	0.961	0.991	v6.0	KKI
RH_cuneus_surfavg	ICC2	0.966	80.64	20	20	6.35E-15	0.899	0.986	v6.0 vs. v7.1	KKI
RH_cuneus_surfavg	ICC3	0.982	108.40	20	20	3.49E-16	0.962	0.991	v7.1	KKI
RH_cuneus_thickavg	ICC3	0.947	36.83	20	20	1.24E-11	0.891	0.975	v5.3	KKI
RH_cuneus_thickavg	ICC2	0.796	71.36	20	20	2.09E-14	-0.005	0.940	v5.3 vs. v6.0	KKI
RH_cuneus_thickavg	ICC2	0.792	18.41	20	20	7.92E-09	0.225	0.923	v5.3 vs. v7.1	KKI
RH_cuneus_thickavg	ICC3	0.951	39.91	20	20	5.75E-12	0.899	0.977	v6.0	KKI
RH_cuneus_thickavg	ICC2	0.935	36.96	20	20	1.20E-11	0.847	0.971	v6.0 vs. v7.1	KKI
RH_cuneus_thickavg	ICC3	0.934	29.41	20	20	1.04E-10	0.865	0.968	v7.1	KKI
RH_entorhinal_surfavg	ICC3	0.772	7.78	20	20	1.27E-05	0.571	0.886	v5.3	KKI
RH_entorhinal_surfavg	ICC2	0.555	15.37	20	20	4.00E-08	-0.050	0.830	v5.3 vs. v6.0	KKI
RH_entorhinal_surfavg	ICC2	0.500	10.37	20	20	1.21E-06	-0.059	0.792	v5.3 vs. v7.1	KKI
RH_entorhinal_surfavg	ICC3	0.913	21.93	20	20	1.60E-09	0.823	0.958	v6.0	KKI
RH_entorhinal_surfavg	ICC2	0.870	14.30	20	20	7.57E-08	0.745	0.936	v6.0 vs. v7.1	KKI
RH_entorhinal_surfavg	ICC3	0.893	17.76	20	20	1.09E-08	0.786	0.948	v7.1	KKI
RH_entorhinal_thickavg	ICC3	0.659	4.87	20	20	4.21E-04	0.393	0.824	v5.3	KKI
RH_entorhinal_thickavg	ICC2	0.835	12.54	20	20	2.39E-07	0.668	0.920	v5.3 vs. v6.0	KKI
RH_entorhinal_thickavg	ICC2	0.578	7.41	20	20	1.88E-05	0.027	0.816	v5.3 vs. v7.1	KKI
RH_entorhinal_thickavg	ICC3	0.797	8.83	20	20	4.59E-06	0.612	0.899	v6.0	KKI
RH_entorhinal_thickavg	ICC2	0.755	11.55	20	20	4.85E-07	0.341	0.896	v6.0 vs. v7.1	KKI
RH_entorhinal_thickavg	ICC3	0.661	4.89	20	20	4.08E-04	0.395	0.825	v7.1	KKI
RH_frontalpole_surfavg	ICC3	0.551	3.45	20	20	3.94E-03	0.238	0.760	v5.3	KKI
RH_frontalpole_surfavg	ICC2	0.341	2.76	20	20	1.40E-02	-0.008	0.619	v5.3 vs. v6.0	KKI
RH_frontalpole_surfavg	ICC2	0.208	3.54	20	20	3.38E-03	-0.067	0.510	v5.3 vs. v7.1	KKI
RH_frontalpole_surfavg	ICC3	0.847	12.05	20	20	3.37E-07	0.700	0.925	v6.0	KKI
RH_frontalpole_surfavg	ICC2	0.445	6.45	20	20	5.45E-05	-0.062	0.744	v6.0 vs. v7.1	KKI
RH_frontalpole_surfavg	ICC3	0.855	12.81	20	20	1.99E-07	0.716	0.929	v7.1	KKI
RH_frontalpole_thickavg	ICC3	0.784	8.26	20	20	7.85E-06	0.591	0.892	v5.3	KKI
RH_frontalpole_thickavg	ICC2	0.856	16.63	20	20	1.98E-08	0.660	0.935	v5.3 vs. v6.0	KKI
RH_frontalpole_thickavg	ICC2	0.829	14.83	20	20	5.51E-08	0.574	0.925	v5.3 vs. v7.1	KKI
RH_frontalpole_thickavg	ICC3	0.830	10.77	20	20	8.81E-07	0.670	0.916	v6.0	KKI
RH_frontalpole_thickavg	ICC2	0.890	16.90	20	20	1.71E-08	0.782	0.947	v6.0 vs. v7.1	KKI
RH_frontalpole_thickavg	ICC3	0.840	11.46	20	20	5.18E-07	0.687	0.921	v7.1	KKI
RH_fusiform_surfavg	ICC3	0.967	59.49	20	20	1.23E-13	0.931	0.984	v5.3	KKI
RH_fusiform_surfavg	ICC2	0.868	23.62	20	20	8.05E-10	0.549	0.948	v5.3 vs. v6.0	KKI
RH_fusiform_surfavg	ICC2	0.883	23.52	20	20	8.36E-10	0.661	0.951	v5.3 vs. v7.1	KKI
RH_fusiform_surfavg	ICC3	0.987	147.97	20	20	1.62E-17	0.972	0.994	v6.0	KKI
RH_fusiform_surfavg	ICC2	0.989	191.05	20	20	1.30E-18	0.976	0.995	v6.0 vs. v7.1	KKI
RH_fusiform_surfavg	ICC3	0.987	152.10	20	20	1.24E-17	0.972	0.994	v7.1	KKI
RH_fusiform_thickavg	ICC3	0.902	19.48	20	20	4.73E-09	0.803	0.953	v5.3	KKI
RH_fusiform_thickavg	ICC2	0.929	26.20	20	20	3.07E-10	0.856	0.966	v5.3 vs. v6.0	KKI
RH_fusiform_thickavg	ICC2	0.785	13.51	20	20	1.25E-07	0.386	0.911	v5.3 vs. v7.1	KKI
RH_fusiform_thickavg	ICC3	0.920	24.03	20	20	6.86E-10	0.838	0.962	v6.0	KKI
RH_fusiform_thickavg	ICC2	0.819	19.73	20	20	4.20E-09	0.326	0.932	v6.0 vs. v7.1	KKI

RH_fusiform_thickavg	ICC3	0.873	14.80	20	20	5.61E-08	0.749	0.938	v7.1	KKI
RH_Hippocampus	ICC3	0.963	53.19	20	20	3.63E-13	0.923	0.982	v5.3	KKI
RH_Hippocampus	ICC2	0.780	28.56	20	20	1.37E-10	0.031	0.929	v5.3 vs. v6.0	KKI
RH_Hippocampus	ICC2	0.863	63.16	20	20	6.88E-14	0.063	0.960	v5.3 vs. v7.1	KKI
RH_Hippocampus	ICC3	0.944	34.88	20	20	2.07E-11	0.885	0.973	v6.0	KKI
RH_Hippocampus	ICC2	0.945	38.70	20	20	7.72E-12	0.885	0.974	v6.0 vs. v7.1	KKI
RH_Hippocampus	ICC3	0.952	40.28	20	20	5.26E-12	0.900	0.977	v7.1	KKI
RH_inferiorparietal_surfavg	ICC3	0.877	15.27	20	20	4.23E-08	0.756	0.940	v5.3	KKI
RH_inferiorparietal_surfavg	ICC2	0.839	15.46	20	20	3.79E-08	0.606	0.928	v5.3 vs. v6.0	KKI
RH_inferiorparietal_surfavg	ICC2	0.848	12.75	20	20	2.06E-07	0.704	0.925	v5.3 vs. v7.1	KKI
RH_inferiorparietal_surfavg	ICC3	0.990	190.62	20	20	1.33E-18	0.978	0.995	v6.0	KKI
RH_inferiorparietal_surfavg	ICC2	0.974	109.52	20	20	3.15E-16	0.920	0.990	v6.0 vs. v7.1	KKI
RH_inferiorparietal_surfavg	ICC3	0.985	136.08	20	20	3.71E-17	0.969	0.993	v7.1	KKI
RH_inferiorparietal_thickavg	ICC3	0.739	6.67	20	20	4.21E-05	0.517	0.868	v5.3	KKI
RH_inferiorparietal_thickavg	ICC2	0.698	8.60	20	20	5.72E-06	0.285	0.865	v5.3 vs. v6.0	KKI
RH_inferiorparietal_thickavg	ICC2	0.809	11.75	20	20	4.20E-07	0.592	0.910	v5.3 vs. v7.1	KKI
RH_inferiorparietal_thickavg	ICC3	0.730	6.42	20	20	5.67E-05	0.503	0.863	v6.0	KKI
RH_inferiorparietal_thickavg	ICC2	0.892	24.16	20	20	6.53E-10	0.709	0.953	v6.0 vs. v7.1	KKI
RH_inferiorparietal_thickavg	ICC3	0.671	5.07	20	20	3.17E-04	0.410	0.830	v7.1	KKI
RH_inferiortemporal_surfavg	ICC3	0.975	78.61	20	20	8.15E-15	0.947	0.988	v5.3	KKI
RH_inferiortemporal_surfavg	ICC2	0.915	56.94	20	20	1.88E-13	0.428	0.973	v5.3 vs. v6.0	KKI
RH_inferiortemporal_surfavg	ICC2	0.866	63.62	20	20	6.41E-14	0.070	0.961	v5.3 vs. v7.1	KKI
RH_inferiortemporal_surfavg	ICC3	0.988	169.28	20	20	4.30E-18	0.975	0.994	v6.0	KKI
RH_inferiortemporal_surfavg	ICC2	0.977	132.20	20	20	4.94E-17	0.912	0.991	v6.0 vs. v7.1	KKI
RH_inferiortemporal_surfavg	ICC3	0.991	228.77	20	20	2.17E-19	0.982	0.996	v7.1	KKI
RH_inferiortemporal_thickavg	ICC3	0.909	20.92	20	20	2.46E-09	0.816	0.956	v5.3	KKI
RH_inferiortemporal_thickavg	ICC2	0.900	18.83	20	20	6.44E-09	0.800	0.951	v5.3 vs. v6.0	KKI
RH_inferiortemporal_thickavg	ICC2	0.860	16.14	20	20	2.59E-08	0.696	0.935	v5.3 vs. v7.1	KKI
RH_inferiortemporal_thickavg	ICC3	0.903	19.62	20	20	4.43E-09	0.805	0.953	v6.0	KKI
RH_inferiortemporal_thickavg	ICC2	0.936	38.06	20	20	9.05E-12	0.846	0.972	v6.0 vs. v7.1	KKI
RH_inferiortemporal_thickavg	ICC3	0.932	28.48	20	20	1.41E-10	0.861	0.967	v7.1	KKI
RH_insula_surfavg	ICC3	0.913	21.90	20	20	1.62E-09	0.823	0.958	v5.3	KKI
RH_insula_surfavg	ICC2	0.513	8.88	20	20	4.38E-06	-0.056	0.794	v5.3 vs. v6.0	KKI
RH_insula_surfavg	ICC2	0.453	6.73	20	20	3.95E-05	-0.062	0.750	v5.3 vs. v7.1	KKI
RH_insula_surfavg	ICC3	0.817	9.93	20	20	1.74E-06	0.648	0.910	v6.0	KKI
RH_insula_surfavg	ICC2	0.858	13.32	20	20	1.41E-07	0.724	0.930	v6.0 vs. v7.1	KKI
RH_insula_surfavg	ICC3	0.823	10.32	20	20	1.26E-06	0.659	0.913	v7.1	KKI
RH_insula_thickavg	ICC3	0.903	19.71	20	20	4.26E-09	0.805	0.953	v5.3	KKI
RH_insula_thickavg	ICC2	0.894	17.08	20	20	1.55E-08	0.787	0.949	v5.3 vs. v6.0	KKI
RH_insula_thickavg	ICC2	0.798	18.57	20	20	7.30E-09	0.250	0.925	v5.3 vs. v7.1	KKI
RH_insula_thickavg	ICC3	0.853	12.65	20	20	2.22E-07	0.712	0.928	v6.0	KKI
RH_insula_thickavg	ICC2	0.792	19.48	20	20	4.72E-09	0.193	0.925	v6.0 vs. v7.1	KKI
RH_insula_thickavg	ICC3	0.869	14.23	20	20	7.92E-08	0.740	0.936	v7.1	KKI
RH_isthmuscingulate_surfavg	ICC3	0.933	28.86	20	20	1.24E-10	0.863	0.968	v5.3	KKI

RH_isthmuscingulate_surfavg	ICC2	0.899	18.22	20	20	8.71E-09	0.798	0.951	v5.3 vs. v6.0	KKI
RH_isthmuscingulate_surfavg	ICC2	0.884	17.17	20	20	1.49E-08	0.769	0.943	v5.3 vs. v7.1	KKI
RH_isthmuscingulate_surfavg	ICC3	0.977	84.85	20	20	3.86E-15	0.951	0.989	v6.0	KKI
RH_isthmuscingulate_surfavg	ICC2	0.972	90.11	20	20	2.14E-15	0.930	0.988	v6.0 vs. v7.1	KKI
RH_isthmuscingulate_surfavg	ICC3	0.972	70.16	20	20	2.47E-14	0.941	0.987	v7.1	KKI
RH_isthmuscingulate_thickavg	ICC3	0.791	8.57	20	20	5.84E-06	0.603	0.896	v5.3	KKI
RH_isthmuscingulate_thickavg	ICC2	0.885	16.54	20	20	2.08E-08	0.774	0.944	v5.3 vs. v6.0	KKI
RH_isthmuscingulate_thickavg	ICC2	0.614	6.91	20	20	3.23E-05	0.137	0.825	v5.3 vs. v7.1	KKI
RH_isthmuscingulate_thickavg	ICC3	0.886	16.47	20	20	2.16E-08	0.772	0.944	v6.0	KKI
RH_isthmuscingulate_thickavg	ICC2	0.771	13.54	20	20	1.23E-07	0.314	0.908	v6.0 vs. v7.1	KKI
RH_isthmuscingulate_thickavg	ICC3	0.883	16.10	20	20	2.64E-08	0.767	0.943	v7.1	KKI
RH_lateraloccipital_surfavg	ICC3	0.954	42.33	20	20	3.27E-12	0.904	0.978	v5.3	KKI
RH_lateraloccipital_surfavg	ICC2	0.849	53.32	20	20	3.55E-13	0.059	0.955	v5.3 vs. v6.0	KKI
RH_lateraloccipital_surfavg	ICC2	0.827	49.42	20	20	7.39E-13	0.029	0.948	v5.3 vs. v7.1	KKI
RH_lateraloccipital_surfavg	ICC3	0.994	343.74	20	20	3.80E-21	0.988	0.997	v6.0	KKI
RH_lateraloccipital_surfavg	ICC2	0.992	286.10	20	20	2.36E-20	0.981	0.996	v6.0 vs. v7.1	KKI
RH_lateraloccipital_surfavg	ICC3	0.992	257.13	20	20	6.81E-20	0.984	0.996	v7.1	KKI
RH_lateraloccipital_thickavg	ICC3	0.900	19.06	20	20	5.76E-09	0.799	0.952	v5.3	KKI
RH_lateraloccipital_thickavg	ICC2	0.749	31.28	20	20	5.82E-11	-0.012	0.919	v5.3 vs. v6.0	KKI
RH_lateraloccipital_thickavg	ICC2	0.753	23.70	20	20	7.80E-10	0.019	0.917	v5.3 vs. v7.1	KKI
RH_lateraloccipital_thickavg	ICC3	0.934	29.43	20	20	1.03E-10	0.865	0.969	v6.0	KKI
RH_lateraloccipital_thickavg	ICC2	0.953	41.71	20	20	3.77E-12	0.903	0.977	v6.0 vs. v7.1	KKI
RH_lateraloccipital_thickavg	ICC3	0.915	22.49	20	20	1.27E-09	0.827	0.959	v7.1	KKI
RH_lateralorbitofrontal_surfavg	ICC3	0.947	36.55	20	20	1.33E-11	0.890	0.975	v5.3	KKI
RH_lateralorbitofrontal_surfavg	ICC2	0.878	64.60	20	20	5.52E-14	0.105	0.964	v5.3 vs. v6.0	KKI
RH_lateralorbitofrontal_surfavg	ICC2	0.758	24.85	20	20	5.02E-10	0.019	0.919	v5.3 vs. v7.1	KKI
RH_lateralorbitofrontal_surfavg	ICC3	0.954	42.66	20	20	3.04E-12	0.905	0.978	v6.0	KKI
RH_lateralorbitofrontal_surfavg	ICC2	0.923	30.63	20	20	7.09E-11	0.820	0.965	v6.0 vs. v7.1	KKI
RH_lateralorbitofrontal_surfavg	ICC3	0.886	16.47	20	20	2.16E-08	0.772	0.944	v7.1	KKI
RH_lateralorbitofrontal_thickavg	ICC3	0.870	14.38	20	20	7.21E-08	0.743	0.937	v5.3	KKI
RH_lateralorbitofrontal_thickavg	ICC2	0.823	10.06	20	20	1.56E-06	0.660	0.912	v5.3 vs. v6.0	KKI
RH_lateralorbitofrontal_thickavg	ICC2	0.805	12.01	20	20	3.46E-07	0.564	0.910	v5.3 vs. v7.1	KKI
RH_lateralorbitofrontal_thickavg	ICC3	0.839	11.44	20	20	5.26E-07	0.687	0.921	v6.0	KKI
RH_lateralorbitofrontal_thickavg	ICC2	0.862	17.45	20	20	1.28E-08	0.674	0.938	v6.0 vs. v7.1	KKI
RH_lateralorbitofrontal_thickavg	ICC3	0.799	8.95	20	20	4.11E-06	0.616	0.900	v7.1	KKI
RH_LateralVentricle	ICC3	1.000	4340.66	20	20	3.87E-32	0.999	1.000	v5.3	KKI
RH_LateralVentricle	ICC2	0.995	1692.71	20	20	4.73E-28	0.860	0.999	v5.3 vs. v6.0	KKI
RH_LateralVentricle	ICC2	0.994	1967.91	20	20	1.05E-28	0.730	0.998	v5.3 vs. v7.1	KKI
RH_LateralVentricle	ICC3	0.999	2808.31	20	20	3.01E-30	0.998	1.000	v6.0	KKI
RH_LateralVentricle	ICC2	1.000	6103.36	20	20	1.28E-33	0.999	1.000	v6.0 vs. v7.1	KKI
RH_LateralVentricle	ICC3	1.000	7004.17	20	20	3.24E-34	0.999	1.000	v7.1	KKI
RH_lingual_surfavg	ICC3	0.967	58.76	20	20	1.38E-13	0.930	0.984	v5.3	KKI
RH_lingual_surfavg	ICC2	0.881	18.75	20	20	6.69E-09	0.743	0.944	v5.3 vs. v6.0	KKI
RH_lingual_surfavg	ICC2	0.860	18.93	20	20	6.13E-09	0.626	0.940	v5.3 vs. v7.1	KKI

RH_lingual_surfavg	ICC3	0.986	137.01	20	20	3.47E-17	0.969	0.993	v6.0	KKI
RH_lingual_surfavg	ICC2	0.990	287.72	20	20	2.23E-20	0.966	0.996	v6.0 vs. v7.1	KKI
RH_lingual_surfavg	ICC3	0.989	183.96	20	20	1.89E-18	0.977	0.995	v7.1	KKI
RH_lingual_thickavg	ICC3	0.941	32.67	20	20	3.85E-11	0.878	0.972	v5.3	KKI
RH_lingual_thickavg	ICC2	0.885	26.35	20	20	2.91E-10	0.613	0.954	v5.3 vs. v6.0	KKI
RH_lingual_thickavg	ICC2	0.936	28.76	20	20	1.28E-10	0.868	0.969	v5.3 vs. v7.1	KKI
RH_lingual_thickavg	ICC3	0.897	18.48	20	20	7.62E-09	0.794	0.950	v6.0	KKI
RH_lingual_thickavg	ICC2	0.916	79.71	20	20	7.11E-15	0.253	0.975	v6.0 vs. v7.1	KKI
RH_lingual_thickavg	ICC3	0.910	21.30	20	20	2.09E-09	0.819	0.957	v7.1	KKI
RH_medialorbitofrontal_surfavg	ICC3	0.862	13.45	20	20	1.30E-07	0.727	0.932	v5.3	KKI
RH_medialorbitofrontal_surfavg	ICC2	0.552	10.36	20	20	1.22E-06	-0.050	0.819	v5.3 vs. v6.0	KKI
RH_medialorbitofrontal_surfavg	ICC2	0.262	5.84	20	20	1.15E-04	-0.056	0.589	v5.3 vs. v7.1	KKI
RH_medialorbitofrontal_surfavg	ICC3	0.918	23.29	20	20	9.18E-10	0.833	0.960	v6.0	KKI
RH_medialorbitofrontal_surfavg	ICC2	0.639	15.64	20	20	3.42E-08	-0.035	0.868	v6.0 vs. v7.1	KKI
RH_medialorbitofrontal_surfavg	ICC3	0.929	27.28	20	20	2.10E-10	0.856	0.966	v7.1	KKI
RH_medialorbitofrontal_thickavg	ICC3	0.868	14.18	20	20	8.15E-08	0.739	0.936	v5.3	KKI
RH_medialorbitofrontal_thickavg	ICC2	0.823	9.85	20	20	1.86E-06	0.656	0.913	v5.3 vs. v6.0	KKI
RH_medialorbitofrontal_thickavg	ICC2	0.706	10.50	20	20	1.09E-06	0.195	0.878	v5.3 vs. v7.1	KKI
RH_medialorbitofrontal_thickavg	ICC3	0.859	13.14	20	20	1.59E-07	0.722	0.931	v6.0	KKI
RH_medialorbitofrontal_thickavg	ICC2	0.709	12.05	20	20	3.38E-07	0.130	0.885	v6.0 vs. v7.1	KKI
RH_medialorbitofrontal_thickavg	ICC3	0.882	15.99	20	20	2.81E-08	0.766	0.943	v7.1	KKI
RH_middletemporal_surfavg	ICC3	0.977	84.70	20	20	3.93E-15	0.951	0.989	v5.3	KKI
RH_middletemporal_surfavg	ICC2	0.922	44.69	20	20	1.95E-12	0.658	0.971	v5.3 vs. v6.0	KKI
RH_middletemporal_surfavg	ICC2	0.889	66.19	20	20	4.36E-14	0.145	0.967	v5.3 vs. v7.1	KKI
RH_middletemporal_surfavg	ICC3	0.985	136.80	20	20	3.53E-17	0.969	0.993	v6.0	KKI
RH_middletemporal_surfavg	ICC2	0.977	163.64	20	20	6.00E-18	0.862	0.992	v6.0 vs. v7.1	KKI
RH_middletemporal_surfavg	ICC3	0.995	402.52	20	20	7.91E-22	0.990	0.998	v7.1	KKI
RH_middletemporal_thickavg	ICC3	0.927	26.40	20	20	2.86E-10	0.851	0.965	v5.3	KKI
RH_middletemporal_thickavg	ICC2	0.900	26.89	20	20	2.41E-10	0.717	0.958	v5.3 vs. v6.0	KKI
RH_middletemporal_thickavg	ICC2	0.742	18.66	20	20	6.99E-09	0.053	0.909	v5.3 vs. v7.1	KKI
RH_middletemporal_thickavg	ICC3	0.922	24.62	20	20	5.47E-10	0.841	0.962	v6.0	KKI
RH_middletemporal_thickavg	ICC2	0.872	37.55	20	20	1.03E-11	0.284	0.958	v6.0 vs. v7.1	KKI
RH_middletemporal_thickavg	ICC3	0.942	33.29	20	20	3.23E-11	0.880	0.972	v7.1	KKI
RH_Pallidum	ICC3	0.869	14.29	20	20	7.62E-08	0.741	0.936	v5.3	KKI
RH_Pallidum	ICC2	0.306	4.69	20	20	5.51E-04	-0.072	0.627	v5.3 vs. v6.0	KKI
RH_Pallidum	ICC2	0.307	4.42	20	20	8.14E-04	-0.073	0.625	v5.3 vs. v7.1	KKI
RH_Pallidum	ICC3	0.915	22.44	20	20	1.29E-09	0.827	0.959	v6.0	KKI
RH_Pallidum	ICC2	0.865	13.56	20	20	1.21E-07	0.736	0.934	v6.0 vs. v7.1	KKI
RH_Pallidum	ICC3	0.908	20.69	20	20	2.72E-09	0.814	0.956	v7.1	KKI
RH_paracentral_surfavg	ICC3	0.943	34.38	20	20	2.38E-11	0.884	0.973	v5.3	KKI
RH_paracentral_surfavg	ICC2	0.777	8.01	20	20	1.01E-05	0.583	0.888	v5.3 vs. v6.0	KKI
RH_paracentral_surfavg	ICC2	0.783	8.51	20	20	6.19E-06	0.593	0.891	v5.3 vs. v7.1	KKI
RH_paracentral_surfavg	ICC3	0.968	61.10	20	20	9.48E-14	0.933	0.985	v6.0	KKI
RH_paracentral_surfavg	ICC2	0.984	122.50	20	20	1.05E-16	0.966	0.992	v6.0 vs. v7.1	KKI

RH_paracentral_surfavg	ICC3	0.962	51.97	20	20	4.54E-13	0.921	0.982	v7.1	KKI
RH_paracentral_thickavg	ICC3	0.862	13.46	20	20	1.29E-07	0.727	0.932	v5.3	KKI
RH_paracentral_thickavg	ICC2	0.782	15.68	20	20	3.35E-08	0.276	0.916	v5.3 vs. v6.0	KKI
RH_paracentral_thickavg	ICC2	0.810	19.13	20	20	5.57E-09	0.296	0.929	v5.3 vs. v7.1	KKI
RH_paracentral_thickavg	ICC3	0.892	17.60	20	20	1.19E-08	0.785	0.948	v6.0	KKI
RH_paracentral_thickavg	ICC2	0.939	30.52	20	20	7.33E-11	0.875	0.971	v6.0 vs. v7.1	KKI
RH_paracentral_thickavg	ICC3	0.903	19.56	20	20	4.56E-09	0.804	0.953	v7.1	KKI
RH_parahippocampal_surfavg	ICC3	0.886	16.52	20	20	2.10E-08	0.772	0.945	v5.3	KKI
RH_parahippocampal_surfavg	ICC2	0.630	6.67	20	20	4.22E-05	0.201	0.828	v5.3 vs. v6.0	KKI
RH_parahippocampal_surfavg	ICC2	0.636	7.44	20	20	1.82E-05	0.160	0.837	v5.3 vs. v7.1	KKI
RH_parahippocampal_surfavg	ICC3	0.824	10.33	20	20	1.25E-06	0.659	0.913	v6.0	KKI
RH_parahippocampal_surfavg	ICC2	0.858	12.89	20	20	1.88E-07	0.723	0.930	v6.0 vs. v7.1	KKI
RH_parahippocampal_surfavg	ICC3	0.847	12.10	20	20	3.25E-07	0.701	0.925	v7.1	KKI
RH_parahippocampal_thickavg	ICC3	0.873	14.79	20	20	5.63E-08	0.749	0.938	v5.3	KKI
RH_parahippocampal_thickavg	ICC2	0.899	17.94	20	20	9.98E-09	0.796	0.951	v5.3 vs. v6.0	KKI
RH_parahippocampal_thickavg	ICC2	0.776	16.17	20	20	2.54E-08	0.226	0.915	v5.3 vs. v7.1	KKI
RH_parahippocampal_thickavg	ICC3	0.897	18.42	20	20	7.88E-09	0.793	0.950	v6.0	KKI
RH_parahippocampal_thickavg	ICC2	0.790	20.86	20	20	2.53E-09	0.151	0.926	v6.0 vs. v7.1	KKI
RH_parahippocampal_thickavg	ICC3	0.916	22.89	20	20	1.08E-09	0.830	0.960	v7.1	KKI
RH_parsopercularis_surfavg	ICC3	0.991	213.25	20	20	4.36E-19	0.980	0.996	v5.3	KKI
RH_parsopercularis_surfavg	ICC2	0.756	7.00	20	20	2.91E-05	0.545	0.877	v5.3 vs. v6.0	KKI
RH_parsopercularis_surfavg	ICC2	0.739	6.48	20	20	5.29E-05	0.517	0.868	v5.3 vs. v7.1	KKI
RH_parsopercularis_surfavg	ICC3	0.909	20.86	20	20	2.53E-09	0.815	0.956	v6.0	KKI
RH_parsopercularis_surfavg	ICC2	0.990	181.45	20	20	2.16E-18	0.978	0.995	v6.0 vs. v7.1	KKI
RH_parsopercularis_surfavg	ICC3	0.986	146.79	20	20	1.76E-17	0.971	0.994	v7.1	KKI
RH_parsopercularis_thickavg	ICC3	0.870	14.35	20	20	7.35E-08	0.742	0.936	v5.3	KKI
RH_parsopercularis_thickavg	ICC2	0.849	17.97	20	20	9.85E-09	0.582	0.936	v5.3 vs. v6.0	KKI
RH_parsopercularis_thickavg	ICC2	0.876	15.99	20	20	2.81E-08	0.756	0.940	v5.3 vs. v7.1	KKI
RH_parsopercularis_thickavg	ICC3	0.920	24.04	20	20	6.84E-10	0.838	0.962	v6.0	KKI
RH_parsopercularis_thickavg	ICC2	0.923	31.14	20	20	6.08E-11	0.816	0.965	v6.0 vs. v7.1	KKI
RH_parsopercularis_thickavg	ICC3	0.833	10.94	20	20	7.68E-07	0.675	0.918	v7.1	KKI
RH_parsorbitalis_surfavg	ICC3	0.939	31.88	20	20	4.87E-11	0.875	0.971	v5.3	KKI
RH_parsorbitalis_surfavg	ICC2	0.779	32.14	20	20	4.51E-11	0.012	0.929	v5.3 vs. v6.0	KKI
RH_parsorbitalis_surfavg	ICC2	0.673	25.74	20	20	3.62E-10	-0.034	0.890	v5.3 vs. v7.1	KKI
RH_parsorbitalis_surfavg	ICC3	0.968	61.07	20	20	9.54E-14	0.933	0.985	v6.0	KKI
RH_parsorbitalis_surfavg	ICC2	0.950	72.69	20	20	1.75E-14	0.757	0.982	v6.0 vs. v7.1	KKI
RH_parsorbitalis_surfavg	ICC3	0.980	97.02	20	20	1.04E-15	0.957	0.990	v7.1	KKI
RH_parsorbitalis_thickavg	ICC3	0.900	18.96	20	20	6.05E-09	0.799	0.952	v5.3	KKI
RH_parsorbitalis_thickavg	ICC2	0.877	16.51	20	20	2.12E-08	0.753	0.940	v5.3 vs. v6.0	KKI
RH_parsorbitalis_thickavg	ICC2	0.872	15.48	20	20	3.75E-08	0.747	0.937	v5.3 vs. v7.1	KKI
RH_parsorbitalis_thickavg	ICC3	0.889	16.97	20	20	1.65E-08	0.778	0.946	v6.0	KKI
RH_parsorbitalis_thickavg	ICC2	0.944	33.41	20	20	3.12E-11	0.885	0.973	v6.0 vs. v7.1	KKI
RH_parsorbitalis_thickavg	ICC3	0.909	20.97	20	20	2.42E-09	0.816	0.956	v7.1	KKI
RH_parstriangularis_surfavg	ICC3	0.983	118.44	20	20	1.46E-16	0.965	0.992	v5.3	KKI

RH_parstriangularis_surfavg	ICC2	0.836	12.80	20	20	2.01E-07	0.666	0.921	v5.3 vs. v6.0	KKI
RH_parstriangularis_surfavg	ICC2	0.827	13.29	20	20	1.44E-07	0.615	0.920	v5.3 vs. v7.1	KKI
RH_parstriangularis_surfavg	ICC3	0.943	34.14	20	20	2.54E-11	0.883	0.973	v6.0	KKI
RH_parstriangularis_surfavg	ICC2	0.993	349.09	20	20	3.26E-21	0.983	0.997	v6.0 vs. v7.1	KKI
RH_parstriangularis_surfavg	ICC3	0.987	151.51	20	20	1.29E-17	0.972	0.994	v7.1	KKI
RH_parstriangularis_thickavg	ICC3	0.902	19.41	20	20	4.89E-09	0.803	0.953	v5.3	KKI
RH_parstriangularis_thickavg	ICC2	0.954	48.15	20	20	9.49E-13	0.898	0.978	v5.3 vs. v6.0	KKI
RH_parstriangularis_thickavg	ICC2	0.921	25.27	20	20	4.30E-10	0.841	0.962	v5.3 vs. v7.1	KKI
RH_parstriangularis_thickavg	ICC3	0.939	31.98	20	20	4.71E-11	0.875	0.971	v6.0	KKI
RH_parstriangularis_thickavg	ICC2	0.941	31.48	20	20	5.48E-11	0.879	0.972	v6.0 vs. v7.1	KKI
RH_parstriangularis_thickavg	ICC3	0.916	22.68	20	20	1.17E-09	0.829	0.959	v7.1	KKI
RH_pericalcarine_surfavg	ICC3	0.971	68.12	20	20	3.29E-14	0.940	0.986	v5.3	KKI
RH_pericalcarine_surfavg	ICC2	0.953	44.98	20	20	1.83E-12	0.900	0.978	v5.3 vs. v6.0	KKI
RH_pericalcarine_surfavg	ICC2	0.942	35.62	20	20	1.70E-11	0.881	0.972	v5.3 vs. v7.1	KKI
RH_pericalcarine_surfavg	ICC3	0.990	194.74	20	20	1.07E-18	0.978	0.995	v6.0	KKI
RH_pericalcarine_surfavg	ICC2	0.992	238.37	20	20	1.45E-19	0.983	0.996	v6.0 vs. v7.1	KKI
RH_pericalcarine_surfavg	ICC3	0.987	151.20	20	20	1.31E-17	0.972	0.994	v7.1	KKI
RH_pericalcarine_thickavg	ICC3	0.844	11.86	20	20	3.87E-07	0.696	0.924	v5.3	KKI
RH_pericalcarine_thickavg	ICC2	0.679	15.47	20	20	3.79E-08	-0.005	0.883	v5.3 vs. v6.0	KKI
RH_pericalcarine_thickavg	ICC2	0.756	13.95	20	20	9.43E-08	0.223	0.905	v5.3 vs. v7.1	KKI
RH_pericalcarine_thickavg	ICC3	0.863	13.65	20	20	1.14E-07	0.731	0.933	v6.0	KKI
RH_pericalcarine_thickavg	ICC2	0.913	25.84	20	20	3.49E-10	0.811	0.960	v6.0 vs. v7.1	KKI
RH_pericalcarine_thickavg	ICC3	0.953	41.54	20	20	3.92E-12	0.903	0.978	v7.1	KKI
RH_postcentral_surfavg	ICC3	0.979	93.68	20	20	1.46E-15	0.956	0.990	v5.3	KKI
RH_postcentral_surfavg	ICC2	0.964	52.11	20	20	4.43E-13	0.925	0.983	v5.3 vs. v6.0	KKI
RH_postcentral_surfavg	ICC2	0.948	36.10	20	20	1.50E-11	0.893	0.975	v5.3 vs. v7.1	KKI
RH_postcentral_surfavg	ICC3	0.989	181.11	20	20	2.20E-18	0.977	0.995	v6.0	KKI
RH_postcentral_surfavg	ICC2	0.991	278.88	20	20	3.04E-20	0.979	0.996	v6.0 vs. v7.1	KKI
RH_postcentral_surfavg	ICC3	0.992	246.34	20	20	1.04E-19	0.983	0.996	v7.1	KKI
RH_postcentral_thickavg	ICC3	0.881	15.80	20	20	3.13E-08	0.763	0.942	v5.3	KKI
RH_postcentral_thickavg	ICC2	0.845	105.67	20	20	4.49E-16	0.012	0.957	v5.3 vs. v6.0	KKI
RH_postcentral_thickavg	ICC2	0.856	56.21	20	20	2.13E-13	0.066	0.957	v5.3 vs. v7.1	KKI
RH_postcentral_thickavg	ICC3	0.984	122.44	20	20	1.05E-16	0.966	0.992	v6.0	KKI
RH_postcentral_thickavg	ICC2	0.972	74.65	20	20	1.35E-14	0.942	0.987	v6.0 vs. v7.1	KKI
RH_postcentral_thickavg	ICC3	0.980	96.68	20	20	1.07E-15	0.957	0.990	v7.1	KKI
RH_posteriorcingulate_surfavg	ICC3	0.987	155.55	20	20	9.91E-18	0.973	0.994	v5.3	KKI
RH_posteriorcingulate_surfavg	ICC2	0.905	19.27	20	20	5.22E-09	0.809	0.954	v5.3 vs. v6.0	KKI
RH_posteriorcingulate_surfavg	ICC2	0.920	22.80	20	20	1.11E-09	0.836	0.961	v5.3 vs. v7.1	KKI
RH_posteriorcingulate_surfavg	ICC3	0.972	70.06	20	20	2.51E-14	0.941	0.987	v6.0	KKI
RH_posteriorcingulate_surfavg	ICC2	0.965	55.80	20	20	2.28E-13	0.928	0.983	v6.0 vs. v7.1	KKI
RH_posteriorcingulate_surfavg	ICC3	0.978	89.64	20	20	2.25E-15	0.954	0.990	v7.1	KKI
RH_posteriorcingulate_thickavg	ICC3	0.899	18.82	20	20	6.48E-09	0.797	0.951	v5.3	KKI
RH_posteriorcingulate_thickavg	ICC2	0.964	56.43	20	20	2.05E-13	0.925	0.983	v5.3 vs. v6.0	KKI
RH_posteriorcingulate_thickavg	ICC2	0.720	17.63	20	20	1.17E-08	0.026	0.901	v5.3 vs. v7.1	KKI

RH_posteriorcingulate_thickavg	ICC3	0.904	19.74	20	20	4.20E-09	0.806	0.953	v6.0	KKI
RH_posteriorcingulate_thickavg	ICC2	0.764	21.15	20	20	2.23E-09	0.066	0.919	v6.0 vs. v7.1	KKI
RH_posteriorcingulate_thickavg	ICC3	0.886	16.55	20	20	2.07E-08	0.773	0.945	v7.1	KKI
RH_precentral_surfavg	ICC3	0.834	11.06	20	20	7.02E-07	0.678	0.918	v5.3	KKI
RH_precentral_surfavg	ICC2	0.764	7.45	20	20	1.79E-05	0.561	0.881	v5.3 vs. v6.0	KKI
RH_precentral_surfavg	ICC2	0.780	8.33	20	20	7.36E-06	0.589	0.889	v5.3 vs. v7.1	KKI
RH_precentral_surfavg	ICC3	0.799	8.96	20	20	4.06E-06	0.617	0.900	v6.0	KKI
RH_precentral_surfavg	ICC2	0.995	485.70	20	20	1.22E-22	0.988	0.998	v6.0 vs. v7.1	KKI
RH_precentral_surfavg	ICC3	0.991	216.02	20	20	3.84E-19	0.981	0.996	v7.1	KKI
RH_precentral_thickavg	ICC3	0.688	5.41	20	20	2.01E-04	0.436	0.840	v5.3	KKI
RH_precentral_thickavg	ICC2	0.731	8.04	20	20	9.81E-06	0.456	0.870	v5.3 vs. v6.0	KKI
RH_precentral_thickavg	ICC2	0.732	7.35	20	20	2.00E-05	0.494	0.865	v5.3 vs. v7.1	KKI
RH_precentral_thickavg	ICC3	0.923	25.09	20	20	4.60E-10	0.844	0.963	v6.0	KKI
RH_precentral_thickavg	ICC2	0.959	50.27	20	20	6.27E-13	0.915	0.981	v6.0 vs. v7.1	KKI
RH_precentral_thickavg	ICC3	0.924	25.35	20	20	4.18E-10	0.845	0.964	v7.1	KKI
RH_precuneus_surfavg	ICC3	0.973	74.35	20	20	1.40E-14	0.944	0.987	v5.3	KKI
RH_precuneus_surfavg	ICC2	0.960	48.20	20	20	9.39E-13	0.918	0.981	v5.3 vs. v6.0	KKI
RH_precuneus_surfavg	ICC2	0.949	38.90	20	20	7.34E-12	0.897	0.976	v5.3 vs. v7.1	KKI
RH_precuneus_surfavg	ICC3	0.991	213.47	20	20	4.32E-19	0.980	0.996	v6.0	KKI
RH_precuneus_surfavg	ICC2	0.996	511.48	20	20	7.27E-23	0.991	0.998	v6.0 vs. v7.1	KKI
RH_precuneus_surfavg	ICC3	0.993	280.17	20	20	2.91E-20	0.985	0.997	v7.1	KKI
RH_precuneus_thickavg	ICC3	0.886	16.48	20	20	2.15E-08	0.772	0.944	v5.3	KKI
RH_precuneus_thickavg	ICC2	0.943	89.95	20	20	2.18E-15	0.534	0.982	v5.3 vs. v6.0	KKI
RH_precuneus_thickavg	ICC2	0.938	40.32	20	20	5.21E-12	0.841	0.973	v5.3 vs. v7.1	KKI
RH_precuneus_thickavg	ICC3	0.915	22.66	20	20	1.18E-09	0.829	0.959	v6.0	KKI
RH_precuneus_thickavg	ICC2	0.954	45.54	20	20	1.62E-12	0.903	0.978	v6.0 vs. v7.1	KKI
RH_precuneus_thickavg	ICC3	0.865	13.77	20	20	1.05E-07	0.733	0.934	v7.1	KKI
RH_Putamen	ICC3	0.958	46.51	20	20	1.33E-12	0.913	0.980	v5.3	KKI
RH_Putamen	ICC2	0.328	17.97	20	20	9.85E-09	-0.023	0.674	v5.3 vs. v6.0	KKI
RH_Putamen	ICC2	0.346	17.37	20	20	1.34E-08	-0.026	0.690	v5.3 vs. v7.1	KKI
RH_Putamen	ICC3	0.985	134.08	20	20	4.30E-17	0.969	0.993	v6.0	KKI
RH_Putamen	ICC2	0.967	69.64	20	20	2.66E-14	0.927	0.985	v6.0 vs. v7.1	KKI
RH_Putamen	ICC3	0.976	81.40	20	20	5.79E-15	0.949	0.988	v7.1	KKI
RH_rostralanteriorcingulate_surfavg	ICC3	0.875	15.01	20	20	4.95E-08	0.752	0.939	v5.3	KKI
RH_rostralanteriorcingulate_surfavg	ICC2	0.662	11.99	20	20	3.53E-07	0.023	0.869	v5.3 vs. v6.0	KKI
RH_rostralanteriorcingulate_surfavg	ICC2	0.674	9.34	20	20	2.90E-06	0.153	0.863	v5.3 vs. v7.1	KKI
RH_rostralanteriorcingulate_surfavg	ICC3	0.968	61.10	20	20	9.48E-14	0.933	0.985	v6.0	KKI
RH_rostralanteriorcingulate_surfavg	ICC2	0.960	64.48	20	20	5.62E-14	0.895	0.983	v6.0 vs. v7.1	KKI
RH_rostralanteriorcingulate_surfavg	ICC3	0.970	66.60	20	20	4.11E-14	0.938	0.986	v7.1	KKI
RH_rostralanteriorcingulate_thickavg	ICC3	0.833	10.94	20	20	7.69E-07	0.675	0.918	v5.3	KKI
RH_rostralanteriorcingulate_thickavg	ICC2	0.757	6.95	20	20	3.09E-05	0.544	0.878	v5.3 vs. v6.0	KKI
RH_rostralanteriorcingulate_thickavg	ICC2	0.574	7.63	20	20	1.49E-05	0.013	0.816	v5.3 vs. v7.1	KKI
RH_rostralanteriorcingulate_thickavg	ICC3	0.900	19.01	20	20	5.92E-09	0.799	0.952	v6.0	KKI
RH_rostralanteriorcingulate_thickavg	ICC2	0.669	23.66	20	20	7.93E-10	-0.035	0.887	v6.0 vs. v7.1	KKI

RH_rostralanteriorcingulate_thickavg	ICC3	0.898	18.65	20	20	7.05E-09	0.795	0.951	v7.1	KKI
RH_rostralmiddlefrontal_surfavg	ICC3	0.971	68.35	20	20	3.19E-14	0.940	0.986	v5.3	KKI
RH_rostralmiddlefrontal_surfavg	ICC2	0.860	14.76	20	20	5.75E-08	0.718	0.932	v5.3 vs. v6.0	KKI
RH_rostralmiddlefrontal_surfavg	ICC2	0.857	14.43	20	20	6.99E-08	0.711	0.931	v5.3 vs. v7.1	KKI
RH_rostralmiddlefrontal_surfavg	ICC3	0.914	22.27	20	20	1.39E-09	0.826	0.959	v6.0	KKI
RH_rostralmiddlefrontal_surfavg	ICC2	0.995	416.34	20	20	5.65E-22	0.990	0.998	v6.0 vs. v7.1	KKI
RH_rostralmiddlefrontal_surfavg	ICC3	0.988	170.50	20	20	4.00E-18	0.975	0.994	v7.1	KKI
RH_rostralmiddlefrontal_thickavg	ICC3	0.719	6.11	20	20	8.23E-05	0.484	0.857	v5.3	KKI
RH_rostralmiddlefrontal_thickavg	ICC2	0.768	15.96	20	20	2.87E-08	0.200	0.913	v5.3 vs. v6.0	KKI
RH_rostralmiddlefrontal_thickavg	ICC2	0.775	12.65	20	20	2.21E-07	0.380	0.906	v5.3 vs. v7.1	KKI
RH_rostralmiddlefrontal_thickavg	ICC3	0.902	19.45	20	20	4.81E-09	0.803	0.953	v6.0	KKI
RH_rostralmiddlefrontal_thickavg	ICC2	0.908	21.85	20	20	1.65E-09	0.816	0.956	v6.0 vs. v7.1	KKI
RH_rostralmiddlefrontal_thickavg	ICC3	0.874	14.92	20	20	5.21E-08	0.751	0.939	v7.1	KKI
RH_superiorfrontal_surfavg	ICC3	0.985	135.39	20	20	3.90E-17	0.969	0.993	v5.3	KKI
RH_superiorfrontal_surfavg	ICC2	0.907	27.80	20	20	1.77E-10	0.756	0.960	v5.3 vs. v6.0	KKI
RH_superiorfrontal_surfavg	ICC2	0.870	21.05	20	20	2.33E-09	0.629	0.945	v5.3 vs. v7.1	KKI
RH_superiorfrontal_surfavg	ICC3	0.991	214.49	20	20	4.12E-19	0.980	0.996	v6.0	KKI
RH_superiorfrontal_surfavg	ICC2	0.986	172.46	20	20	3.57E-18	0.967	0.994	v6.0 vs. v7.1	KKI
RH_superiorfrontal_surfavg	ICC3	0.985	131.01	20	20	5.40E-17	0.968	0.993	v7.1	KKI
RH_superiorfrontal_thickavg	ICC3	0.806	9.32	20	20	2.95E-06	0.629	0.904	v5.3	KKI
RH_superiorfrontal_thickavg	ICC2	0.928	26.50	20	20	2.76E-10	0.855	0.965	v5.3 vs. v6.0	KKI
RH_superiorfrontal_thickavg	ICC2	0.941	31.63	20	20	5.24E-11	0.879	0.972	v5.3 vs. v7.1	KKI
RH_superiorfrontal_thickavg	ICC3	0.859	13.23	20	20	1.50E-07	0.723	0.931	v6.0	KKI
RH_superiorfrontal_thickavg	ICC2	0.946	35.56	20	20	1.73E-11	0.889	0.974	v6.0 vs. v7.1	KKI
RH_superiorfrontal_thickavg	ICC3	0.803	9.18	20	20	3.35E-06	0.624	0.902	v7.1	KKI
RH_superiorparietal_surfavg	ICC3	0.886	16.53	20	20	2.09E-08	0.772	0.945	v5.3	KKI
RH_superiorparietal_surfavg	ICC2	0.921	23.70	20	20	7.80E-10	0.841	0.962	v5.3 vs. v6.0	KKI
RH_superiorparietal_surfavg	ICC2	0.913	21.27	20	20	2.12E-09	0.825	0.958	v5.3 vs. v7.1	KKI
RH_superiorparietal_surfavg	ICC3	0.984	124.45	20	20	8.96E-17	0.966	0.992	v6.0	KKI
RH_superiorparietal_surfavg	ICC2	0.995	415.32	20	20	5.79E-22	0.990	0.998	v6.0 vs. v7.1	KKI
RH_superiorparietal_surfavg	ICC3	0.987	154.98	20	20	1.03E-17	0.973	0.994	v7.1	KKI
RH_superiorparietal_thickavg	ICC3	0.883	16.06	20	20	2.70E-08	0.766	0.943	v5.3	KKI
RH_superiorparietal_thickavg	ICC2	0.871	34.78	20	20	2.13E-11	0.322	0.957	v5.3 vs. v6.0	KKI
RH_superiorparietal_thickavg	ICC2	0.870	38.13	20	20	8.88E-12	0.263	0.958	v5.3 vs. v7.1	KKI
RH_superiorparietal_thickavg	ICC3	0.886	16.57	20	20	2.04E-08	0.773	0.945	v6.0	KKI
RH_superiorparietal_thickavg	ICC2	0.984	117.82	20	20	1.54E-16	0.966	0.992	v6.0 vs. v7.1	KKI
RH_superiorparietal_thickavg	ICC3	0.844	11.85	20	20	3.91E-07	0.696	0.924	v7.1	KKI
RH_superiortemporal_surfavg	ICC3	0.984	125.14	20	20	8.49E-17	0.967	0.993	v5.3	KKI
RH_superiortemporal_surfavg	ICC2	0.910	83.29	20	20	4.63E-15	0.191	0.974	v5.3 vs. v6.0	KKI
RH_superiortemporal_surfavg	ICC2	0.859	68.37	20	20	3.18E-14	0.046	0.959	v5.3 vs. v7.1	KKI
RH_superiortemporal_surfavg	ICC3	0.989	184.60	20	20	1.82E-18	0.977	0.995	v6.0	KKI
RH_superiortemporal_surfavg	ICC2	0.986	317.84	20	20	8.29E-21	0.880	0.995	v6.0 vs. v7.1	KKI
RH_superiortemporal_surfavg	ICC3	0.988	166.53	20	20	5.05E-18	0.975	0.994	v7.1	KKI
RH_superiortemporal_thickavg	ICC3	0.966	58.38	20	20	1.48E-13	0.930	0.984	v5.3	KKI

RH_superiortemporal_thickavg	ICC2	0.976	80.78	20	20	6.24E-15	0.950	0.989	v5.3 vs. v6.0	KKI
RH_superiortemporal_thickavg	ICC2	0.974	82.84	20	20	4.88E-15	0.943	0.988	v5.3 vs. v7.1	KKI
RH_superiortemporal_thickavg	ICC3	0.972	70.01	20	20	2.53E-14	0.941	0.987	v6.0	KKI
RH_superiortemporal_thickavg	ICC2	0.971	86.71	20	20	3.12E-15	0.922	0.987	v6.0 vs. v7.1	KKI
RH_superiortemporal_thickavg	ICC3	0.972	69.19	20	20	2.83E-14	0.940	0.986	v7.1	KKI
RH_supramarginal_surfavg	ICC3	0.977	87.44	20	20	2.88E-15	0.953	0.989	v5.3	KKI
RH_supramarginal_surfavg	ICC2	0.793	10.25	20	20	1.34E-06	0.582	0.900	v5.3 vs. v6.0	KKI
RH_supramarginal_surfavg	ICC2	0.822	11.02	20	20	7.25E-07	0.656	0.912	v5.3 vs. v7.1	KKI
RH_supramarginal_surfavg	ICC3	0.988	167.69	20	20	4.72E-18	0.975	0.994	v6.0	KKI
RH_supramarginal_surfavg	ICC2	0.989	255.10	20	20	7.37E-20	0.967	0.996	v6.0 vs. v7.1	KKI
RH_supramarginal_surfavg	ICC3	0.996	479.07	20	20	1.40E-22	0.991	0.998	v7.1	KKI
RH_supramarginal_thickavg	ICC3	0.876	15.13	20	20	4.60E-08	0.754	0.940	v5.3	KKI
RH_supramarginal_thickavg	ICC2	0.885	18.70	20	20	6.86E-09	0.759	0.945	v5.3 vs. v6.0	KKI
RH_supramarginal_thickavg	ICC2	0.908	20.48	20	20	2.99E-09	0.816	0.955	v5.3 vs. v7.1	KKI
RH_supramarginal_thickavg	ICC3	0.894	17.79	20	20	1.08E-08	0.787	0.948	v6.0	KKI
RH_supramarginal_thickavg	ICC2	0.936	34.12	20	20	2.56E-11	0.864	0.970	v6.0 vs. v7.1	KKI
RH_supramarginal_thickavg	ICC3	0.852	12.54	20	20	2.40E-07	0.710	0.928	v7.1	KKI
RH_SurfArea	ICC3	0.997	584.54	20	20	1.92E-23	0.993	0.998	v5.3	KKI
RH_SurfArea	ICC2	0.983	984.58	20	20	1.06E-25	0.404	0.996	v5.3 vs. v6.0	KKI
RH_SurfArea	ICC2	0.954	772.72	20	20	1.19E-24	0.103	0.989	v5.3 vs. v7.1	KKI
RH_SurfArea	ICC3	0.996	519.42	20	20	6.24E-23	0.992	0.998	v6.0	KKI
RH_SurfArea	ICC2	0.990	1561.98	20	20	1.06E-27	0.564	0.998	v6.0 vs. v7.1	KKI
RH_SurfArea	ICC3	0.995	384.23	20	20	1.26E-21	0.989	0.998	v7.1	KKI
RH_temporalpole_surfavg	ICC3	0.732	6.46	20	20	5.44E-05	0.505	0.864	v5.3	KKI
RH_temporalpole_surfavg	ICC2	0.410	5.01	20	20	3.46E-04	-0.058	0.709	v5.3 vs. v6.0	KKI
RH_temporalpole_surfavg	ICC2	0.226	3.44	20	20	4.02E-03	-0.071	0.531	v5.3 vs. v7.1	KKI
RH_temporalpole_surfavg	ICC3	0.815	9.83	20	20	1.89E-06	0.645	0.909	v6.0	KKI
RH_temporalpole_surfavg	ICC2	0.713	12.15	20	20	3.13E-07	0.138	0.887	v6.0 vs. v7.1	KKI
RH_temporalpole_surfavg	ICC3	0.858	13.04	20	20	1.70E-07	0.720	0.930	v7.1	KKI
RH_temporalpole_thickavg	ICC3	0.751	7.03	20	20	2.81E-05	0.536	0.875	v5.3	KKI
RH_temporalpole_thickavg	ICC2	0.796	13.80	20	20	1.04E-07	0.434	0.914	v5.3 vs. v6.0	KKI
RH_temporalpole_thickavg	ICC2	0.790	24.26	20	20	6.28E-10	0.093	0.929	v5.3 vs. v7.1	KKI
RH_temporalpole_thickavg	ICC3	0.698	5.61	20	20	1.54E-04	0.451	0.845	v6.0	KKI
RH_temporalpole_thickavg	ICC2	0.844	12.50	20	20	2.45E-07	0.696	0.923	v6.0 vs. v7.1	KKI
RH_temporalpole_thickavg	ICC3	0.650	4.71	20	20	5.31E-04	0.378	0.818	v7.1	KKI
RH_Thalamus	ICC3	0.931	28.17	20	20	1.56E-10	0.860	0.967	v5.3	KKI
RH_Thalamus	ICC2	0.906	19.58	20	20	4.53E-09	0.811	0.955	v5.3 vs. v6.0	KKI
RH_Thalamus	ICC2	0.805	28.17	20	20	1.56E-10	0.089	0.936	v5.3 vs. v7.1	KKI
RH_Thalamus	ICC3	0.940	32.28	20	20	4.32E-11	0.877	0.971	v6.0	KKI
RH_Thalamus	ICC2	0.787	28.89	20	20	1.23E-10	0.039	0.931	v6.0 vs. v7.1	KKI
RH_Thalamus	ICC3	0.952	40.24	20	20	5.31E-12	0.900	0.977	v7.1	KKI
RH_Thickness	ICC3	0.908	20.79	20	20	2.61E-09	0.815	0.956	v5.3	KKI
RH_Thickness	ICC2	0.902	53.08	20	20	3.71E-13	0.330	0.969	v5.3 vs. v6.0	KKI
RH_Thickness	ICC2	0.958	58.45	20	20	1.46E-13	0.897	0.981	v5.3 vs. v7.1	KKI

RH_Thickness	ICC3	0.951	39.79	20	20	5.92E-12	0.899	0.977	v6.0	KKI
RH_Thickness	ICC2	0.936	53.23	20	20	3.60E-13	0.729	0.976	v6.0 vs. v7.1	KKI
RH_Thickness	ICC3	0.913	21.92	20	20	1.61E-09	0.823	0.958	v7.1	KKI
RH_transversetemporal_surfavg	ICC3	0.806	9.30	20	20	2.99E-06	0.628	0.904	v5.3	KKI
RH_transversetemporal_surfavg	ICC2	0.641	4.69	20	20	5.48E-04	0.374	0.812	v5.3 vs. v6.0	KKI
RH_transversetemporal_surfavg	ICC2	0.579	3.98	20	20	1.63E-03	0.289	0.775	v5.3 vs. v7.1	KKI
RH_transversetemporal_surfavg	ICC3	0.778	8.02	20	20	9.99E-06	0.581	0.889	v6.0	KKI
RH_transversetemporal_surfavg	ICC2	0.831	10.65	20	20	9.68E-07	0.674	0.916	v6.0 vs. v7.1	KKI
RH_transversetemporal_surfavg	ICC3	0.855	12.80	20	20	2.00E-07	0.715	0.929	v7.1	KKI
RH_transversetemporal_thickavg	ICC3	0.888	16.91	20	20	1.70E-08	0.777	0.946	v5.3	KKI
RH_transversetemporal_thickavg	ICC2	0.882	45.41	20	20	1.67E-12	0.255	0.962	v5.3 vs. v6.0	KKI
RH_transversetemporal_thickavg	ICC2	0.876	43.85	20	20	2.33E-12	0.228	0.961	v5.3 vs. v7.1	KKI
RH_transversetemporal_thickavg	ICC3	0.905	20.06	20	20	3.62E-09	0.808	0.954	v6.0	KKI
RH_transversetemporal_thickavg	ICC2	0.973	69.54	20	20	2.70E-14	0.943	0.987	v6.0 vs. v7.1	KKI
RH_transversetemporal_thickavg	ICC3	0.894	17.86	20	20	1.04E-08	0.787	0.949	v7.1	KKI
SUM_bankssts_surfavg	ICC3	0.956	44.76	20	20	1.91E-12	0.909	0.979	v5.3	KKI
SUM_bankssts_surfavg	ICC2	0.792	14.48	20	20	6.79E-08	0.379	0.915	v5.3 vs. v6.0	KKI
SUM_bankssts_surfavg	ICC2	0.770	14.31	20	20	7.51E-08	0.274	0.910	v5.3 vs. v7.1	KKI
SUM_bankssts_surfavg	ICC3	0.982	113.21	20	20	2.28E-16	0.963	0.992	v6.0	KKI
SUM_bankssts_surfavg	ICC2	0.989	205.20	20	20	6.39E-19	0.976	0.995	v6.0 vs. v7.1	KKI
SUM_bankssts_surfavg	ICC3	0.980	98.58	20	20	8.88E-16	0.958	0.990	v7.1	KKI
SUM_caudalanteriorcingulate_surfavg	ICC3	0.973	73.93	20	20	1.48E-14	0.944	0.987	v5.3	KKI
SUM_caudalanteriorcingulate_surfavg	ICC2	0.828	26.07	20	20	3.21E-10	0.211	0.941	v5.3 vs. v6.0	KKI
SUM_caudalanteriorcingulate_surfavg	ICC2	0.859	13.50	20	20	1.26E-07	0.726	0.931	v5.3 vs. v7.1	KKI
SUM_caudalanteriorcingulate_surfavg	ICC3	0.989	186.82	20	20	1.62E-18	0.978	0.995	v6.0	KKI
SUM_caudalanteriorcingulate_surfavg	ICC2	0.909	75.50	20	20	1.21E-14	0.223	0.973	v6.0 vs. v7.1	KKI
SUM_caudalanteriorcingulate_surfavg	ICC3	0.974	76.89	20	20	1.01E-14	0.946	0.988	v7.1	KKI
SUM_caudalmiddlefrontal_surfavg	ICC3	0.942	33.51	20	20	3.03E-11	0.881	0.972	v5.3	KKI
SUM_caudalmiddlefrontal_surfavg	ICC2	0.936	31.81	20	20	4.96E-11	0.870	0.969	v5.3 vs. v6.0	KKI
SUM_caudalmiddlefrontal_surfavg	ICC2	0.879	14.79	20	20	5.62E-08	0.758	0.941	v5.3 vs. v7.1	KKI
SUM_caudalmiddlefrontal_surfavg	ICC3	0.847	12.05	20	20	3.38E-07	0.700	0.925	v6.0	KKI
SUM_caudalmiddlefrontal_surfavg	ICC2	0.928	27.27	20	20	2.12E-10	0.854	0.965	v6.0 vs. v7.1	KKI
SUM_caudalmiddlefrontal_surfavg	ICC3	0.959	47.34	20	20	1.12E-12	0.914	0.980	v7.1	KKI
SUM_cuneus_surfavg	ICC3	0.971	67.59	20	20	3.55E-14	0.939	0.986	v5.3	KKI
SUM_cuneus_surfavg	ICC2	0.879	27.29	20	20	2.10E-10	0.549	0.954	v5.3 vs. v6.0	KKI
SUM_cuneus_surfavg	ICC2	0.843	24.12	20	20	6.63E-10	0.354	0.943	v5.3 vs. v7.1	KKI
SUM_cuneus_surfavg	ICC3	0.985	128.19	20	20	6.69E-17	0.967	0.993	v6.0	KKI
SUM_cuneus_surfavg	ICC2	0.983	148.08	20	20	1.61E-17	0.958	0.993	v6.0 vs. v7.1	KKI
SUM_cuneus_surfavg	ICC3	0.988	168.00	20	20	4.63E-18	0.975	0.994	v7.1	KKI
SUM_entorhinal_surfavg	ICC3	0.897	18.48	20	20	7.63E-09	0.794	0.950	v5.3	KKI
SUM_entorhinal_surfavg	ICC2	0.519	11.42	20	20	5.34E-07	-0.057	0.805	v5.3 vs. v6.0	KKI
SUM_entorhinal_surfavg	ICC2	0.523	7.18	20	20	2.41E-05	-0.032	0.790	v5.3 vs. v7.1	KKI
SUM_entorhinal_surfavg	ICC3	0.959	47.84	20	20	1.01E-12	0.915	0.981	v6.0	KKI
SUM_entorhinal_surfavg	ICC2	0.934	29.39	20	20	1.05E-10	0.866	0.968	v6.0 vs. v7.1	KKI

SUM_entorhinal_surfavg	ICC3	0.948	37.34	20	20	1.09E-11	0.892	0.975	v7.1	KKI
SUM_frontalpole_surfavg	ICC3	0.667	5.00	20	20	3.51E-04	0.404	0.828	v5.3	KKI
SUM_frontalpole_surfavg	ICC2	0.221	3.28	20	20	5.31E-03	-0.072	0.523	v5.3 vs. v6.0	KKI
SUM_frontalpole_surfavg	ICC2	0.152	5.43	20	20	1.96E-04	-0.033	0.431	v5.3 vs. v7.1	KKI
SUM_frontalpole_surfavg	ICC3	0.847	12.08	20	20	3.30E-07	0.701	0.925	v6.0	KKI
SUM_frontalpole_surfavg	ICC2	0.439	8.22	20	20	8.17E-06	-0.064	0.749	v6.0 vs. v7.1	KKI
SUM_frontalpole_surfavg	ICC3	0.864	13.76	20	20	1.06E-07	0.733	0.934	v7.1	KKI
SUM_fusiform_surfavg	ICC3	0.976	80.99	20	20	6.09E-15	0.949	0.988	v5.3	KKI
SUM_fusiform_surfavg	ICC2	0.866	27.14	20	20	2.21E-10	0.444	0.951	v5.3 vs. v6.0	KKI
SUM_fusiform_surfavg	ICC2	0.907	33.36	20	20	3.16E-11	0.669	0.964	v5.3 vs. v7.1	KKI
SUM_fusiform_surfavg	ICC3	0.995	395.25	20	20	9.48E-22	0.989	0.998	v6.0	KKI
SUM_fusiform_surfavg	ICC2	0.987	237.99	20	20	1.47E-19	0.956	0.995	v6.0 vs. v7.1	KKI
SUM_fusiform_surfavg	ICC3	0.993	284.35	20	20	2.51E-20	0.985	0.997	v7.1	KKI
SUM_inferiorparietal_surfavg	ICC3	0.974	76.55	20	20	1.06E-14	0.946	0.988	v5.3	KKI
SUM_inferiorparietal_surfavg	ICC2	0.889	34.77	20	20	2.14E-11	0.474	0.961	v5.3 vs. v6.0	KKI
SUM_inferiorparietal_surfavg	ICC2	0.891	24.62	20	20	5.48E-10	0.695	0.954	v5.3 vs. v7.1	KKI
SUM_inferiorparietal_surfavg	ICC3	0.996	557.76	20	20	3.07E-23	0.992	0.998	v6.0	KKI
SUM_inferiorparietal_surfavg	ICC2	0.991	316.85	20	20	8.55E-21	0.974	0.996	v6.0 vs. v7.1	KKI
SUM_inferiorparietal_surfavg	ICC3	0.991	230.68	20	20	2.00E-19	0.982	0.996	v7.1	KKI
SUM_inferiortemporal_surfavg	ICC3	0.985	133.29	20	20	4.56E-17	0.969	0.993	v5.3	KKI
SUM_inferiortemporal_surfavg	ICC2	0.949	115.95	20	20	1.80E-16	0.490	0.985	v5.3 vs. v6.0	KKI
SUM_inferiortemporal_surfavg	ICC2	0.903	67.48	20	20	3.61E-14	0.223	0.971	v5.3 vs. v7.1	KKI
SUM_inferiortemporal_surfavg	ICC3	0.994	336.80	20	20	4.66E-21	0.987	0.997	v6.0	KKI
SUM_inferiortemporal_surfavg	ICC2	0.986	280.39	20	20	2.88E-20	0.912	0.995	v6.0 vs. v7.1	KKI
SUM_inferiortemporal_surfavg	ICC3	0.989	175.15	20	20	3.07E-18	0.976	0.995	v7.1	KKI
SUM_insula_surfavg	ICC3	0.851	12.46	20	20	2.53E-07	0.709	0.927	v5.3	KKI
SUM_insula_surfavg	ICC2	0.447	10.81	20	20	8.55E-07	-0.056	0.761	v5.3 vs. v6.0	KKI
SUM_insula_surfavg	ICC2	0.416	8.95	20	20	4.13E-06	-0.060	0.736	v5.3 vs. v7.1	KKI
SUM_insula_surfavg	ICC3	0.907	20.61	20	20	2.83E-09	0.813	0.955	v6.0	KKI
SUM_insula_surfavg	ICC2	0.846	11.87	20	20	3.83E-07	0.702	0.924	v6.0 vs. v7.1	KKI
SUM_insula_surfavg	ICC3	0.906	20.29	20	20	3.26E-09	0.810	0.955	v7.1	KKI
SUM_isthmuscingulate_surfavg	ICC3	0.963	52.68	20	20	3.99E-13	0.922	0.982	v5.3	KKI
SUM_isthmuscingulate_surfavg	ICC2	0.870	13.97	20	20	9.34E-08	0.744	0.937	v5.3 vs. v6.0	KKI
SUM_isthmuscingulate_surfavg	ICC2	0.849	12.93	20	20	1.83E-07	0.707	0.926	v5.3 vs. v7.1	KKI
SUM_isthmuscingulate_surfavg	ICC3	0.992	250.77	20	20	8.74E-20	0.983	0.996	v6.0	KKI
SUM_isthmuscingulate_surfavg	ICC2	0.984	182.75	20	20	2.01E-18	0.942	0.994	v6.0 vs. v7.1	KKI
SUM_isthmuscingulate_surfavg	ICC3	0.992	237.92	20	20	1.47E-19	0.982	0.996	v7.1	KKI
SUM_lateraloccipital_surfavg	ICC3	0.980	98.95	20	20	8.55E-16	0.958	0.991	v5.3	KKI
SUM_lateraloccipital_surfavg	ICC2	0.870	84.40	20	20	4.07E-15	0.044	0.963	v5.3 vs. v6.0	KKI
SUM_lateraloccipital_surfavg	ICC2	0.836	68.19	20	20	3.26E-14	0.017	0.953	v5.3 vs. v7.1	KKI
SUM_lateraloccipital_surfavg	ICC3	0.995	440.05	20	20	3.26E-22	0.990	0.998	v6.0	KKI
SUM_lateraloccipital_surfavg	ICC2	0.994	640.66	20	20	7.71E-24	0.968	0.998	v6.0 vs. v7.1	KKI
SUM_lateraloccipital_surfavg	ICC3	0.996	480.91	20	20	1.34E-22	0.991	0.998	v7.1	KKI
SUM_lateralorbitofrontal_surfavg	ICC3	0.974	75.97	20	20	1.14E-14	0.946	0.988	v5.3	KKI

SUM_lateralorbitofrontal_surfavg	ICC2	0.911	110.35	20	20	2.93E-16	0.124	0.975	v5.3 vs. v6.0	KKI
SUM_lateralorbitofrontal_surfavg	ICC2	0.793	62.76	20	20	7.32E-14	-0.006	0.939	v5.3 vs. v7.1	KKI
SUM_lateralorbitofrontal_surfavg	ICC3	0.975	78.72	20	20	8.04E-15	0.947	0.988	v6.0	KKI
SUM_lateralorbitofrontal_surfavg	ICC2	0.941	74.99	20	20	1.29E-14	0.611	0.981	v6.0 vs. v7.1	KKI
SUM_lateralorbitofrontal_surfavg	ICC3	0.968	60.64	20	20	1.02E-13	0.932	0.985	v7.1	KKI
SUM_lingual_surfavg	ICC3	0.973	73.26	20	20	1.62E-14	0.944	0.987	v5.3	KKI
SUM_lingual_surfavg	ICC2	0.922	26.65	20	20	2.62E-10	0.841	0.963	v5.3 vs. v6.0	KKI
SUM_lingual_surfavg	ICC2	0.909	27.01	20	20	2.31E-10	0.775	0.960	v5.3 vs. v7.1	KKI
SUM_lingual_surfavg	ICC3	0.990	190.62	20	20	1.33E-18	0.978	0.995	v6.0	KKI
SUM_lingual_surfavg	ICC2	0.993	492.99	20	20	1.05E-22	0.960	0.997	v6.0 vs. v7.1	KKI
SUM_lingual_surfavg	ICC3	0.996	490.26	20	20	1.11E-22	0.991	0.998	v7.1	KKI
SUM_medialorbitofrontal_surfavg	ICC3	0.876	15.08	20	20	4.74E-08	0.753	0.939	v5.3	KKI
SUM_medialorbitofrontal_surfavg	ICC2	0.537	8.43	20	20	6.67E-06	-0.043	0.804	v5.3 vs. v6.0	KKI
SUM_medialorbitofrontal_surfavg	ICC2	0.395	10.40	20	20	1.18E-06	-0.051	0.724	v5.3 vs. v7.1	KKI
SUM_medialorbitofrontal_surfavg	ICC3	0.943	34.35	20	20	2.40E-11	0.884	0.973	v6.0	KKI
SUM_medialorbitofrontal_surfavg	ICC2	0.776	17.61	20	20	1.18E-08	0.178	0.918	v6.0 vs. v7.1	KKI
SUM_medialorbitofrontal_surfavg	ICC3	0.917	23.07	20	20	9.99E-10	0.831	0.960	v7.1	KKI
SUM_middletemporal_surfavg	ICC3	0.989	173.34	20	20	3.40E-18	0.976	0.995	v5.3	KKI
SUM_middletemporal_surfavg	ICC2	0.960	100.35	20	20	7.45E-16	0.750	0.986	v5.3 vs. v6.0	KKI
SUM_middletemporal_surfavg	ICC2	0.920	138.75	20	20	3.07E-17	0.122	0.978	v5.3 vs. v7.1	KKI
SUM_middletemporal_surfavg	ICC3	0.990	202.02	20	20	7.46E-19	0.979	0.995	v6.0	KKI
SUM_middletemporal_surfavg	ICC2	0.980	363.02	20	20	2.21E-21	0.649	0.994	v6.0 vs. v7.1	KKI
SUM_middletemporal_surfavg	ICC3	0.996	518.17	20	20	6.39E-23	0.992	0.998	v7.1	KKI
SUM_paracentral_surfavg	ICC3	0.798	8.88	20	20	4.38E-06	0.614	0.899	v5.3	KKI
SUM_paracentral_surfavg	ICC2	0.876	15.87	20	20	3.01E-08	0.755	0.939	v5.3 vs. v6.0	KKI
SUM_paracentral_surfavg	ICC2	0.769	8.58	20	20	5.82E-06	0.558	0.885	v5.3 vs. v7.1	KKI
SUM_paracentral_surfavg	ICC3	0.981	105.38	20	20	4.61E-16	0.960	0.991	v6.0	KKI
SUM_paracentral_surfavg	ICC2	0.953	48.42	20	20	8.99E-13	0.895	0.978	v6.0 vs. v7.1	KKI
SUM_paracentral_surfavg	ICC3	0.964	55.15	20	20	2.56E-13	0.926	0.983	v7.1	KKI
SUM_parahippocampal_surfavg	ICC3	0.922	24.65	20	20	5.43E-10	0.841	0.963	v5.3	KKI
SUM_parahippocampal_surfavg	ICC2	0.662	7.38	20	20	1.94E-05	0.247	0.845	v5.3 vs. v6.0	KKI
SUM_parahippocampal_surfavg	ICC2	0.592	5.19	20	20	2.71E-04	0.227	0.795	v5.3 vs. v7.1	KKI
SUM_parahippocampal_surfavg	ICC3	0.868	14.13	20	20	8.43E-08	0.739	0.936	v6.0	KKI
SUM_parahippocampal_surfavg	ICC2	0.878	14.66	20	20	6.07E-08	0.756	0.941	v6.0 vs. v7.1	KKI
SUM_parahippocampal_surfavg	ICC3	0.895	18.05	20	20	9.46E-09	0.789	0.949	v7.1	KKI
SUM_parsopercularis_surfavg	ICC3	0.989	175.72	20	20	2.97E-18	0.976	0.995	v5.3	KKI
SUM_parsopercularis_surfavg	ICC2	0.919	31.86	20	20	4.88E-11	0.783	0.965	v5.3 vs. v6.0	KKI
SUM_parsopercularis_surfavg	ICC2	0.822	10.22	20	20	1.37E-06	0.660	0.912	v5.3 vs. v7.1	KKI
SUM_parsopercularis_surfavg	ICC3	0.991	223.23	20	20	2.77E-19	0.981	0.996	v6.0	KKI
SUM_parsopercularis_surfavg	ICC2	0.935	29.90	20	20	8.90E-11	0.869	0.969	v6.0 vs. v7.1	KKI
SUM_parsopercularis_surfavg	ICC3	0.964	54.54	20	20	2.85E-13	0.925	0.983	v7.1	KKI
SUM_parsorbitalis_surfavg	ICC3	0.956	44.11	20	20	2.20E-12	0.908	0.979	v5.3	KKI
SUM_parsorbitalis_surfavg	ICC2	0.706	32.08	20	20	4.58E-11	-0.027	0.904	v5.3 vs. v6.0	KKI
SUM_parsorbitalis_surfavg	ICC2	0.593	25.40	20	20	4.10E-10	-0.036	0.855	v5.3 vs. v7.1	KKI

SUM_parsorbitalis_surfavg	ICC3	0.977	87.15	20	20	2.97E-15	0.952	0.989	v6.0	KKI
SUM_parsorbitalis_surfavg	ICC2	0.951	114.45	20	20	2.05E-16	0.529	0.985	v6.0 vs. v7.1	KKI
SUM_parsorbitalis_surfavg	ICC3	0.989	176.46	20	20	2.85E-18	0.976	0.995	v7.1	KKI
SUM_parstriangularis_surfavg	ICC3	0.987	158.54	20	20	8.21E-18	0.974	0.994	v5.3	KKI
SUM_parstriangularis_surfavg	ICC2	0.865	18.57	20	20	7.31E-09	0.665	0.940	v5.3 vs. v6.0	KKI
SUM_parstriangularis_surfavg	ICC2	0.787	12.34	20	20	2.75E-07	0.456	0.907	v5.3 vs. v7.1	KKI
SUM_parstriangularis_surfavg	ICC3	0.993	288.04	20	20	2.21E-20	0.985	0.997	v6.0	KKI
SUM_parstriangularis_surfavg	ICC2	0.980	153.46	20	20	1.13E-17	0.923	0.992	v6.0 vs. v7.1	KKI
SUM_parstriangularis_surfavg	ICC3	0.958	46.87	20	20	1.23E-12	0.913	0.980	v7.1	KKI
SUM_pericalcarine_surfavg	ICC3	0.982	112.16	20	20	2.49E-16	0.963	0.992	v5.3	KKI
SUM_pericalcarine_surfavg	ICC2	0.947	50.80	20	20	5.66E-13	0.847	0.978	v5.3 vs. v6.0	KKI
SUM_pericalcarine_surfavg	ICC2	0.950	53.29	20	20	3.57E-13	0.858	0.979	v5.3 vs. v7.1	KKI
SUM_pericalcarine_surfavg	ICC3	0.990	193.69	20	20	1.13E-18	0.978	0.995	v6.0	KKI
SUM_pericalcarine_surfavg	ICC2	0.994	318.55	20	20	8.11E-21	0.987	0.997	v6.0 vs. v7.1	KKI
SUM_pericalcarine_surfavg	ICC3	0.993	270.48	20	20	4.12E-20	0.984	0.997	v7.1	KKI
SUM_postcentral_surfavg	ICC3	0.879	15.52	20	20	3.67E-08	0.759	0.941	v5.3	KKI
SUM_postcentral_surfavg	ICC2	0.861	13.15	20	20	1.58E-07	0.729	0.932	v5.3 vs. v6.0	KKI
SUM_postcentral_surfavg	ICC2	0.972	67.85	20	20	3.43E-14	0.942	0.987	v5.3 vs. v7.1	KKI
SUM_postcentral_surfavg	ICC3	0.819	10.07	20	20	1.56E-06	0.651	0.911	v6.0	KKI
SUM_postcentral_surfavg	ICC2	0.843	11.43	20	20	5.29E-07	0.695	0.923	v6.0 vs. v7.1	KKI
SUM_postcentral_surfavg	ICC3	0.822	10.24	20	20	1.35E-06	0.656	0.912	v7.1	KKI
SUM_posteriorcingulate_surfavg	ICC3	0.968	61.62	20	20	8.73E-14	0.933	0.985	v5.3	KKI
SUM_posteriorcingulate_surfavg	ICC2	0.884	15.70	20	20	3.31E-08	0.769	0.944	v5.3 vs. v6.0	KKI
SUM_posteriorcingulate_surfavg	ICC2	0.880	15.35	20	20	4.04E-08	0.763	0.941	v5.3 vs. v7.1	KKI
SUM_posteriorcingulate_surfavg	ICC3	0.992	249.01	20	20	9.37E-20	0.983	0.996	v6.0	KKI
SUM_posteriorcingulate_surfavg	ICC2	0.977	139.52	20	20	2.90E-17	0.907	0.991	v6.0 vs. v7.1	KKI
SUM_posteriorcingulate_surfavg	ICC3	0.991	219.96	20	20	3.21E-19	0.981	0.996	v7.1	KKI
SUM_precentral_surfavg	ICC3	0.913	21.93	20	20	1.60E-09	0.823	0.958	v5.3	KKI
SUM_precentral_surfavg	ICC2	0.915	22.16	20	20	1.45E-09	0.830	0.959	v5.3 vs. v6.0	KKI
SUM_precentral_surfavg	ICC2	0.702	5.79	20	20	1.23E-04	0.465	0.847	v5.3 vs. v7.1	KKI
SUM_precentral_surfavg	ICC3	0.995	390.13	20	20	1.08E-21	0.989	0.998	v6.0	KKI
SUM_precentral_surfavg	ICC2	0.911	22.77	20	20	1.13E-09	0.821	0.957	v6.0 vs. v7.1	KKI
SUM_precentral_surfavg	ICC3	0.905	20.01	20	20	3.70E-09	0.808	0.954	v7.1	KKI
SUM_precuneus_surfavg	ICC3	0.991	232.03	20	20	1.89E-19	0.982	0.996	v5.3	KKI
SUM_precuneus_surfavg	ICC2	0.963	50.53	20	20	5.96E-13	0.923	0.982	v5.3 vs. v6.0	KKI
SUM_precuneus_surfavg	ICC2	0.959	45.87	20	20	1.52E-12	0.915	0.981	v5.3 vs. v7.1	KKI
SUM_precuneus_surfavg	ICC3	0.996	452.28	20	20	2.48E-22	0.991	0.998	v6.0	KKI
SUM_precuneus_surfavg	ICC2	0.997	570.11	20	20	2.47E-23	0.993	0.998	v6.0 vs. v7.1	KKI
SUM_precuneus_surfavg	ICC3	0.996	472.40	20	20	1.61E-22	0.991	0.998	v7.1	KKI
SUM_rostralanteriorcingulate_surfavg	ICC3	0.920	23.99	20	20	6.98E-10	0.837	0.962	v5.3	KKI
SUM_rostralanteriorcingulate_surfavg	ICC2	0.755	16.03	20	20	2.76E-08	0.147	0.909	v5.3 vs. v6.0	KKI
SUM_rostralanteriorcingulate_surfavg	ICC2	0.815	11.94	20	20	3.64E-07	0.609	0.912	v5.3 vs. v7.1	KKI
SUM_rostralanteriorcingulate_surfavg	ICC3	0.961	50.05	20	20	6.54E-13	0.919	0.981	v6.0	KKI
SUM_rostralanteriorcingulate_surfavg	ICC2	0.927	73.38	20	20	1.60E-14	0.417	0.977	v6.0 vs. v7.1	KKI

SUM_rostralanteriorcingulate_surfavg	ICC3	0.960	48.77	20	20	8.39E-13	0.917	0.981	v7.1	KKI
SUM_rostralmiddlefrontal_surfavg	ICC3	0.974	74.97	20	20	1.29E-14	0.945	0.988	v5.3	KKI
SUM_rostralmiddlefrontal_surfavg	ICC2	0.953	46.10	20	20	1.44E-12	0.898	0.978	v5.3 vs. v6.0	KKI
SUM_rostralmiddlefrontal_surfavg	ICC2	0.867	14.92	20	20	5.22E-08	0.737	0.935	v5.3 vs. v7.1	KKI
SUM_rostralmiddlefrontal_surfavg	ICC3	0.990	204.65	20	20	6.56E-19	0.979	0.995	v6.0	KKI
SUM_rostralmiddlefrontal_surfavg	ICC2	0.965	55.17	20	20	2.55E-13	0.927	0.983	v6.0 vs. v7.1	KKI
SUM_rostralmiddlefrontal_surfavg	ICC3	0.949	38.42	20	20	8.27E-12	0.895	0.976	v7.1	KKI
SUM_superiorfrontal_surfavg	ICC3	0.990	196.45	20	20	9.84E-19	0.979	0.995	v5.3	KKI
SUM_superiorfrontal_surfavg	ICC2	0.950	104.28	20	20	5.11E-16	0.566	0.985	v5.3 vs. v6.0	KKI
SUM_superiorfrontal_surfavg	ICC2	0.868	31.27	20	20	5.84E-11	0.364	0.954	v5.3 vs. v7.1	KKI
SUM_superiorfrontal_surfavg	ICC3	0.979	92.73	20	20	1.62E-15	0.955	0.990	v6.0	KKI
SUM_superiorfrontal_surfavg	ICC2	0.967	84.21	20	20	4.16E-15	0.895	0.986	v6.0 vs. v7.1	KKI
SUM_superiorfrontal_surfavg	ICC3	0.979	95.52	20	20	1.21E-15	0.956	0.990	v7.1	KKI
SUM_superiorparietal_surfavg	ICC3	0.960	49.59	20	20	7.14E-13	0.918	0.981	v5.3	KKI
SUM_superiorparietal_surfavg	ICC2	0.961	52.34	20	20	4.24E-13	0.921	0.982	v5.3 vs. v6.0	KKI
SUM_superiorparietal_surfavg	ICC2	0.966	58.92	20	20	1.35E-13	0.929	0.984	v5.3 vs. v7.1	KKI
SUM_superiorparietal_surfavg	ICC3	0.990	191.19	20	20	1.29E-18	0.978	0.995	v6.0	KKI
SUM_superiorparietal_surfavg	ICC2	0.998	944.15	20	20	1.61E-25	0.996	0.999	v6.0 vs. v7.1	KKI
SUM_superiorparietal_surfavg	ICC3	0.992	256.26	20	20	7.05E-20	0.984	0.996	v7.1	KKI
SUM_superiortemporal_surfavg	ICC3	0.991	211.40	20	20	4.76E-19	0.980	0.996	v5.3	KKI
SUM_superiortemporal_surfavg	ICC2	0.873	104.28	20	20	5.11E-16	0.035	0.965	v5.3 vs. v6.0	KKI
SUM_superiortemporal_surfavg	ICC2	0.813	117.35	20	20	1.60E-16	0.002	0.948	v5.3 vs. v7.1	KKI
SUM_superiortemporal_surfavg	ICC3	0.992	253.82	20	20	7.75E-20	0.983	0.996	v6.0	KKI
SUM_superiortemporal_surfavg	ICC2	0.985	371.44	20	20	1.76E-21	0.799	0.995	v6.0 vs. v7.1	KKI
SUM_superiortemporal_surfavg	ICC3	0.996	467.49	20	20	1.78E-22	0.991	0.998	v7.1	KKI
SUM_supramarginal_surfavg	ICC3	0.971	68.87	20	20	2.96E-14	0.940	0.986	v5.3	KKI
SUM_supramarginal_surfavg	ICC2	0.952	43.70	20	20	2.41E-12	0.900	0.977	v5.3 vs. v6.0	KKI
SUM_supramarginal_surfavg	ICC2	0.925	29.69	20	20	9.50E-11	0.835	0.965	v5.3 vs. v7.1	KKI
SUM_supramarginal_surfavg	ICC3	0.976	81.78	20	20	5.54E-15	0.949	0.989	v6.0	KKI
SUM_supramarginal_surfavg	ICC2	0.990	227.73	20	20	2.27E-19	0.976	0.995	v6.0 vs. v7.1	KKI
SUM_supramarginal_surfavg	ICC3	0.987	148.38	20	20	1.58E-17	0.972	0.994	v7.1	KKI
SUM_SurfArea	ICC3	0.996	537.49	20	20	4.44E-23	0.992	0.998	v5.3	KKI
SUM_SurfArea	ICC2	0.982	1380.71	20	20	3.62E-27	0.310	0.996	v5.3 vs. v6.0	KKI
SUM_SurfArea	ICC2	0.954	770.02	20	20	1.23E-24	0.105	0.989	v5.3 vs. v7.1	KKI
SUM_SurfArea	ICC3	0.996	531.89	20	20	4.93E-23	0.992	0.998	v6.0	KKI
SUM_SurfArea	ICC2	0.992	2632.76	20	20	5.73E-30	0.541	0.998	v6.0 vs. v7.1	KKI
SUM_SurfArea	ICC3	0.997	595.60	20	20	1.60E-23	0.993	0.998	v7.1	KKI
SUM_temporalpole_surfavg	ICC3	0.749	6.97	20	20	3.03E-05	0.533	0.873	v5.3	KKI
SUM_temporalpole_surfavg	ICC2	0.446	5.20	20	20	2.68E-04	-0.040	0.733	v5.3 vs. v6.0	KKI
SUM_temporalpole_surfavg	ICC2	0.287	4.73	20	20	5.15E-04	-0.070	0.609	v5.3 vs. v7.1	KKI
SUM_temporalpole_surfavg	ICC3	0.905	19.96	20	20	3.80E-09	0.808	0.954	v6.0	KKI
SUM_temporalpole_surfavg	ICC2	0.700	12.91	20	20	1.85E-07	0.077	0.885	v6.0 vs. v7.1	KKI
SUM_temporalpole_surfavg	ICC3	0.895	18.00	20	20	9.71E-09	0.789	0.949	v7.1	KKI
SUM_transversetemporal_surfavg	ICC3	0.949	38.27	20	20	8.59E-12	0.895	0.976	v5.3	KKI

SUM_transversetemporal_surfavg	ICC2	0.893	27.94	20	20	1.68E-10	0.648	0.957	v5.3 vs. v6.0	KKI
SUM_transversetemporal_surfavg	ICC2	0.876	18.20	20	20	8.77E-09	0.729	0.942	v5.3 vs. v7.1	KKI
SUM_transversetemporal_surfavg	ICC3	0.964	54.68	20	20	2.78E-13	0.925	0.983	v6.0	KKI
SUM_transversetemporal_surfavg	ICC2	0.959	50.20	20	20	6.35E-13	0.915	0.980	v6.0 vs. v7.1	KKI
SUM_transversetemporal_surfavg	ICC3	0.963	53.38	20	20	3.51E-13	0.923	0.983	v7.1	KKI

Table S5. OASIS specific ICCs and associated statistics.

ROI	Type	ICC	F	df1	df2	p	lower bound	upper boun	comparison	dataset
AVG_Accumbens	ICC3	0.898	18.56	19	19	1.71E-08	0.791	0.952	v5.3	OASIS
AVG_Accumbens	ICC2	0.770	14.61	19	19	1.32E-07	0.254	0.911	v5.3 vs. v6.0	OASIS
AVG_Accumbens	ICC2	0.730	14.18	19	19	1.69E-07	0.117	0.899	v5.3 vs. v7.1	OASIS
AVG_Accumbens	ICC3	0.935	29.87	19	19	2.60E-10	0.865	0.970	v6.0	OASIS
AVG_Accumbens	ICC2	0.950	41.25	19	19	1.41E-11	0.895	0.977	v6.0 vs. v7.1	OASIS
AVG_Accumbens	ICC3	0.936	30.06	19	19	2.46E-10	0.865	0.970	v7.1	OASIS
AVG_Amygdala	ICC3	0.876	15.14	19	19	9.72E-08	0.749	0.941	v5.3	OASIS
AVG_Amygdala	ICC2	0.941	40.23	19	19	1.77E-11	0.859	0.974	v5.3 vs. v6.0	OASIS
AVG_Amygdala	ICC2	0.911	20.46	19	19	7.36E-09	0.816	0.958	v5.3 vs. v7.1	OASIS
AVG_Amygdala	ICC3	0.904	19.84	19	19	9.58E-09	0.803	0.955	v6.0	OASIS
AVG_Amygdala	ICC2	0.943	37.45	19	19	3.39E-11	0.878	0.974	v6.0 vs. v7.1	OASIS
AVG_Amygdala	ICC3	0.910	21.12	19	19	5.57E-09	0.814	0.957	v7.1	OASIS
AVG_bankssts_thickavg	ICC3	0.787	8.38	19	19	1.17E-05	0.589	0.896	v5.3	OASIS
AVG_bankssts_thickavg	ICC2	0.870	16.57	19	19	4.53E-08	0.725	0.939	v5.3 vs. v6.0	OASIS
AVG_bankssts_thickavg	ICC2	0.879	14.83	19	19	1.16E-07	0.754	0.943	v5.3 vs. v7.1	OASIS
AVG_bankssts_thickavg	ICC3	0.835	11.10	19	19	1.27E-06	0.673	0.920	v6.0	OASIS
AVG_bankssts_thickavg	ICC2	0.888	21.53	19	19	4.70E-09	0.730	0.950	v6.0 vs. v7.1	OASIS
AVG_bankssts_thickavg	ICC3	0.970	65.16	19	19	2.13E-13	0.936	0.986	v7.1	OASIS
AVG_caudalanteriorcingulate_thickavg	ICC3	0.680	5.25	19	19	3.49E-04	0.415	0.838	v5.3	OASIS
AVG_caudalanteriorcingulate_thickavg	ICC2	0.817	9.49	19	19	4.43E-06	0.640	0.912	v5.3 vs. v6.0	OASIS
AVG_caudalanteriorcingulate_thickavg	ICC2	0.483	14.99	19	19	1.06E-07	-0.046	0.791	v5.3 vs. v7.1	OASIS
AVG_caudalanteriorcingulate_thickavg	ICC3	0.841	11.54	19	19	9.25E-07	0.684	0.923	v6.0	OASIS
AVG_caudalanteriorcingulate_thickavg	ICC2	0.454	7.12	19	19	4.01E-05	-0.064	0.755	v6.0 vs. v7.1	OASIS
AVG_caudalanteriorcingulate_thickavg	ICC3	0.817	9.94	19	19	3.07E-06	0.642	0.911	v7.1	OASIS
AVG_caudalmiddlefrontal_thickavg	ICC3	0.860	13.24	19	19	2.98E-07	0.719	0.933	v5.3	OASIS
AVG_caudalmiddlefrontal_thickavg	ICC2	0.851	33.76	19	19	8.66E-11	0.206	0.952	v5.3 vs. v6.0	OASIS
AVG_caudalmiddlefrontal_thickavg	ICC2	0.816	20.78	19	19	6.42E-09	0.275	0.934	v5.3 vs. v7.1	OASIS
AVG_caudalmiddlefrontal_thickavg	ICC3	0.844	11.86	19	19	7.40E-07	0.691	0.925	v6.0	OASIS
AVG_caudalmiddlefrontal_thickavg	ICC2	0.956	42.38	19	19	1.11E-11	0.907	0.979	v6.0 vs. v7.1	OASIS
AVG_caudalmiddlefrontal_thickavg	ICC3	0.828	10.61	19	19	1.82E-06	0.661	0.917	v7.1	OASIS
AVG_Caudate	ICC3	0.978	91.15	19	19	9.46E-15	0.954	0.990	v5.3	OASIS
AVG_Caudate	ICC2	0.921	96.18	19	19	5.74E-15	0.221	0.977	v5.3 vs. v6.0	OASIS
AVG_Caudate	ICC2	0.977	115.46	19	19	1.04E-15	0.932	0.990	v5.3 vs. v7.1	OASIS
AVG_Caudate	ICC3	0.982	109.90	19	19	1.65E-15	0.961	0.992	v6.0	OASIS
AVG_Caudate	ICC2	0.962	209.01	19	19	3.96E-18	0.425	0.990	v6.0 vs. v7.1	OASIS
AVG_Caudate	ICC3	0.992	250.80	19	19	7.11E-19	0.983	0.996	v7.1	OASIS
AVG_cuneus_thickavg	ICC3	0.947	36.59	19	19	4.19E-11	0.888	0.975	v5.3	OASIS
AVG_cuneus_thickavg	ICC2	0.896	54.58	19	19	1.09E-12	0.267	0.968	v5.3 vs. v6.0	OASIS
AVG_cuneus_thickavg	ICC2	0.914	22.30	19	19	3.47E-09	0.825	0.959	v5.3 vs. v7.1	OASIS
AVG_cuneus_thickavg	ICC3	0.951	39.67	19	19	2.01E-11	0.896	0.977	v6.0	OASIS
AVG_cuneus_thickavg	ICC2	0.901	37.78	19	19	3.14E-11	0.537	0.965	v6.0 vs. v7.1	OASIS

AVG_cuneus_thickavg	ICC3	0.969	63.49	19	19	2.71E-13	0.934	0.986	v7.1	OASIS
AVG_entorhinal_thickavg	ICC3	0.888	16.89	19	19	3.84E-08	0.772	0.947	v5.3	OASIS
AVG_entorhinal_thickavg	ICC2	0.804	13.66	19	19	2.31E-07	0.482	0.917	v5.3 vs. v6.0	OASIS
AVG_entorhinal_thickavg	ICC2	0.797	10.11	19	19	2.68E-06	0.594	0.902	v5.3 vs. v7.1	OASIS
AVG_entorhinal_thickavg	ICC3	0.903	19.59	19	19	1.07E-08	0.801	0.954	v6.0	OASIS
AVG_entorhinal_thickavg	ICC2	0.710	30.18	19	19	2.37E-10	-0.026	0.906	v6.0 vs. v7.1	OASIS
AVG_entorhinal_thickavg	ICC3	0.917	23.21	19	19	2.44E-09	0.829	0.961	v7.1	OASIS
AVG_frontalpole_thickavg	ICC3	0.786	8.36	19	19	1.18E-05	0.588	0.895	v5.3	OASIS
AVG_frontalpole_thickavg	ICC2	0.787	13.40	19	19	2.70E-07	0.399	0.912	v5.3 vs. v6.0	OASIS
AVG_frontalpole_thickavg	ICC2	0.741	8.79	19	19	8.04E-06	0.441	0.880	v5.3 vs. v7.1	OASIS
AVG_frontalpole_thickavg	ICC3	0.793	8.67	19	19	8.93E-06	0.600	0.899	v6.0	OASIS
AVG_frontalpole_thickavg	ICC2	0.831	10.40	19	19	2.14E-06	0.666	0.918	v6.0 vs. v7.1	OASIS
AVG_frontalpole_thickavg	ICC3	0.904	19.85	19	19	9.55E-09	0.803	0.955	v7.1	OASIS
AVG_fusiform_thickavg	ICC3	0.616	4.21	19	19	1.47E-03	0.320	0.802	v5.3	OASIS
AVG_fusiform_thickavg	ICC2	0.635	11.14	19	19	1.23E-06	0.001	0.858	v5.3 vs. v6.0	OASIS
AVG_fusiform_thickavg	ICC2	0.665	6.18	19	19	1.12E-04	0.353	0.836	v5.3 vs. v7.1	OASIS
AVG_fusiform_thickavg	ICC3	0.751	7.04	19	19	4.34E-05	0.529	0.877	v6.0	OASIS
AVG_fusiform_thickavg	ICC2	0.774	10.99	19	19	1.38E-06	0.459	0.900	v6.0 vs. v7.1	OASIS
AVG_fusiform_thickavg	ICC3	0.885	16.32	19	19	5.14E-08	0.766	0.945	v7.1	OASIS
AVG_Hippocampus	ICC3	0.947	36.54	19	19	4.24E-11	0.888	0.975	v5.3	OASIS
AVG_Hippocampus	ICC2	0.925	25.96	19	19	9.08E-10	0.845	0.964	v5.3 vs. v6.0	OASIS
AVG_Hippocampus	ICC2	0.944	34.14	19	19	7.83E-11	0.883	0.973	v5.3 vs. v7.1	OASIS
AVG_Hippocampus	ICC3	0.971	68.36	19	19	1.37E-13	0.939	0.987	v6.0	OASIS
AVG_Hippocampus	ICC2	0.934	35.41	19	19	5.64E-11	0.846	0.970	v6.0 vs. v7.1	OASIS
AVG_Hippocampus	ICC3	0.983	117.63	19	19	8.76E-16	0.964	0.992	v7.1	OASIS
AVG_inferiorparietal_thickavg	ICC3	0.851	12.40	19	19	5.12E-07	0.702	0.928	v5.3	OASIS
AVG_inferiorparietal_thickavg	ICC2	0.893	18.04	19	19	2.18E-08	0.784	0.949	v5.3 vs. v6.0	OASIS
AVG_inferiorparietal_thickavg	ICC2	0.860	13.32	19	19	2.83E-07	0.723	0.933	v5.3 vs. v7.1	OASIS
AVG_inferiorparietal_thickavg	ICC3	0.920	24.05	19	19	1.78E-09	0.835	0.962	v6.0	OASIS
AVG_inferiorparietal_thickavg	ICC2	0.895	24.56	19	19	1.48E-09	0.723	0.955	v6.0 vs. v7.1	OASIS
AVG_inferiorparietal_thickavg	ICC3	0.890	17.14	19	19	3.39E-08	0.775	0.948	v7.1	OASIS
AVG_inferiortemporal_thickavg	ICC3	0.699	5.64	19	19	2.12E-04	0.445	0.849	v5.3	OASIS
AVG_inferiortemporal_thickavg	ICC2	0.783	10.26	19	19	2.39E-06	0.538	0.899	v5.3 vs. v6.0	OASIS
AVG_inferiortemporal_thickavg	ICC2	0.797	8.69	19	19	8.80E-06	0.609	0.901	v5.3 vs. v7.1	OASIS
AVG_inferiortemporal_thickavg	ICC3	0.774	7.84	19	19	1.94E-05	0.567	0.889	v6.0	OASIS
AVG_inferiortemporal_thickavg	ICC2	0.821	11.68	19	19	8.41E-07	0.635	0.915	v6.0 vs. v7.1	OASIS
AVG_inferiortemporal_thickavg	ICC3	0.938	31.46	19	19	1.63E-10	0.871	0.971	v7.1	OASIS
AVG_insula_thickavg	ICC3	0.801	9.04	19	19	6.47E-06	0.613	0.903	v5.3	OASIS
AVG_insula_thickavg	ICC2	0.687	8.90	19	19	7.33E-06	0.220	0.866	v5.3 vs. v6.0	OASIS
AVG_insula_thickavg	ICC2	0.774	10.52	19	19	1.95E-06	0.485	0.898	v5.3 vs. v7.1	OASIS
AVG_insula_thickavg	ICC3	0.879	15.48	19	19	8.06E-08	0.754	0.942	v6.0	OASIS
AVG_insula_thickavg	ICC2	0.594	46.37	19	19	4.87E-12	-0.019	0.861	v6.0 vs. v7.1	OASIS
AVG_insula_thickavg	ICC3	0.940	32.37	19	19	1.27E-10	0.874	0.972	v7.1	OASIS
AVG_isthmuscingulate_thickavg	ICC3	0.879	15.58	19	19	7.63E-08	0.756	0.943	v5.3	OASIS

AVG_isthmuscingulate_thickavg	ICC2	0.912	45.86	19	19	5.39E-12	0.525	0.970	v5.3 vs. v6.0	OASIS
AVG_isthmuscingulate_thickavg	ICC2	0.528	22.71	19	19	2.95E-09	-0.035	0.822	v5.3 vs. v7.1	OASIS
AVG_isthmuscingulate_thickavg	ICC3	0.928	26.73	19	19	7.00E-10	0.850	0.966	v6.0	OASIS
AVG_isthmuscingulate_thickavg	ICC2	0.689	41.13	19	19	1.45E-11	-0.025	0.901	v6.0 vs. v7.1	OASIS
AVG_isthmuscingulate_thickavg	ICC3	0.932	28.53	19	19	3.92E-10	0.859	0.968	v7.1	OASIS
AVG_lateraloccipital_thickavg	ICC3	0.871	14.51	19	19	1.39E-07	0.740	0.938	v5.3	OASIS
AVG_lateraloccipital_thickavg	ICC2	0.891	22.37	19	19	3.37E-09	0.730	0.952	v5.3 vs. v6.0	OASIS
AVG_lateraloccipital_thickavg	ICC2	0.896	18.91	19	19	1.45E-08	0.789	0.950	v5.3 vs. v7.1	OASIS
AVG_lateraloccipital_thickavg	ICC3	0.925	25.83	19	19	9.50E-10	0.845	0.965	v6.0	OASIS
AVG_lateraloccipital_thickavg	ICC2	0.876	31.57	19	19	1.58E-10	0.424	0.957	v6.0 vs. v7.1	OASIS
AVG_lateraloccipital_thickavg	ICC3	0.965	56.75	19	19	7.63E-13	0.926	0.984	v7.1	OASIS
AVG_lateralorbitofrontal_thickavg	ICC3	0.680	5.26	19	19	3.45E-04	0.416	0.839	v5.3	OASIS
AVG_lateralorbitofrontal_thickavg	ICC2	0.676	9.38	19	19	4.84E-06	0.151	0.865	v5.3 vs. v6.0	OASIS
AVG_lateralorbitofrontal_thickavg	ICC2	0.665	5.00	19	19	4.83E-04	0.400	0.829	v5.3 vs. v7.1	OASIS
AVG_lateralorbitofrontal_thickavg	ICC3	0.765	7.51	19	19	2.67E-05	0.552	0.884	v6.0	OASIS
AVG_lateralorbitofrontal_thickavg	ICC2	0.734	11.24	19	19	1.15E-06	0.260	0.891	v6.0 vs. v7.1	OASIS
AVG_lateralorbitofrontal_thickavg	ICC3	0.791	8.55	19	19	9.96E-06	0.596	0.898	v7.1	OASIS
AVG_LateralVentricle	ICC3	0.998	1235.69	19	19	1.98E-25	0.996	0.999	v5.3	OASIS
AVG_LateralVentricle	ICC2	0.994	3419.08	19	19	1.26E-29	0.596	0.998	v5.3 vs. v6.0	OASIS
AVG_LateralVentricle	ICC2	0.993	3628.17	19	19	7.18E-30	0.518	0.998	v5.3 vs. v7.1	OASIS
AVG_LateralVentricle	ICC3	0.999	1761.69	19	19	6.84E-27	0.998	0.999	v6.0	OASIS
AVG_LateralVentricle	ICC2	1.000	6892.87	19	19	1.62E-32	0.999	1.000	v6.0 vs. v7.1	OASIS
AVG_LateralVentricle	ICC3	0.999	1801.73	19	19	5.52E-27	0.998	0.999	v7.1	OASIS
AVG_lingual_thickavg	ICC3	0.891	17.34	19	19	3.07E-08	0.778	0.948	v5.3	OASIS
AVG_lingual_thickavg	ICC2	0.777	23.95	19	19	1.85E-09	0.060	0.925	v5.3 vs. v6.0	OASIS
AVG_lingual_thickavg	ICC2	0.878	14.74	19	19	1.22E-07	0.753	0.942	v5.3 vs. v7.1	OASIS
AVG_lingual_thickavg	ICC3	0.895	18.03	19	19	2.19E-08	0.785	0.950	v6.0	OASIS
AVG_lingual_thickavg	ICC2	0.785	26.56	19	19	7.41E-10	0.053	0.930	v6.0 vs. v7.1	OASIS
AVG_lingual_thickavg	ICC3	0.911	21.35	19	19	5.07E-09	0.816	0.958	v7.1	OASIS
AVG_medialorbitofrontal_thickavg	ICC3	0.764	7.49	19	19	2.74E-05	0.551	0.884	v5.3	OASIS
AVG_medialorbitofrontal_thickavg	ICC2	0.794	14.66	19	19	1.27E-07	0.378	0.917	v5.3 vs. v6.0	OASIS
AVG_medialorbitofrontal_thickavg	ICC2	0.785	8.20	19	19	1.37E-05	0.590	0.894	v5.3 vs. v7.1	OASIS
AVG_medialorbitofrontal_thickavg	ICC3	0.829	10.73	19	19	1.67E-06	0.664	0.918	v6.0	OASIS
AVG_medialorbitofrontal_thickavg	ICC2	0.855	29.21	19	19	3.17E-10	0.312	0.950	v6.0 vs. v7.1	OASIS
AVG_medialorbitofrontal_thickavg	ICC3	0.799	8.96	19	19	6.93E-06	0.610	0.902	v7.1	OASIS
AVG_middletemporal_thickavg	ICC3	0.772	7.76	19	19	2.10E-05	0.563	0.888	v5.3	OASIS
AVG_middletemporal_thickavg	ICC2	0.872	15.89	19	19	6.47E-08	0.741	0.939	v5.3 vs. v6.0	OASIS
AVG_middletemporal_thickavg	ICC2	0.742	6.90	19	19	5.04E-05	0.520	0.871	v5.3 vs. v7.1	OASIS
AVG_middletemporal_thickavg	ICC3	0.792	8.60	19	19	9.58E-06	0.597	0.898	v6.0	OASIS
AVG_middletemporal_thickavg	ICC2	0.766	10.02	19	19	2.88E-06	0.474	0.893	v6.0 vs. v7.1	OASIS
AVG_middletemporal_thickavg	ICC3	0.915	22.58	19	19	3.11E-09	0.825	0.960	v7.1	OASIS
AVG_Pallidum	ICC3	0.930	27.77	19	19	4.99E-10	0.855	0.967	v5.3	OASIS
AVG_Pallidum	ICC2	0.790	10.25	19	19	2.40E-06	0.562	0.901	v5.3 vs. v6.0	OASIS
AVG_Pallidum	ICC2	0.709	9.96	19	19	3.02E-06	0.233	0.878	v5.3 vs. v7.1	OASIS

AVG_Pallidum	ICC3	0.951	39.65	19	19	2.02E-11	0.896	0.977	v6.0	OASIS
AVG_Pallidum	ICC2	0.911	37.93	19	19	3.03E-11	0.632	0.967	v6.0 vs. v7.1	OASIS
AVG_Pallidum	ICC3	0.936	30.49	19	19	2.16E-10	0.867	0.970	v7.1	OASIS
AVG_paracentral_thickavg	ICC3	0.882	15.97	19	19	6.19E-08	0.761	0.944	v5.3	OASIS
AVG_paracentral_thickavg	ICC2	0.681	21.88	19	19	4.09E-09	-0.031	0.891	v5.3 vs. v6.0	OASIS
AVG_paracentral_thickavg	ICC2	0.728	20.12	19	19	8.51E-09	0.012	0.907	v5.3 vs. v7.1	OASIS
AVG_paracentral_thickavg	ICC3	0.874	14.93	19	19	1.09E-07	0.746	0.940	v6.0	OASIS
AVG_paracentral_thickavg	ICC2	0.962	62.33	19	19	3.22E-13	0.907	0.983	v6.0 vs. v7.1	OASIS
AVG_paracentral_thickavg	ICC3	0.905	20.01	19	19	8.93E-09	0.804	0.955	v7.1	OASIS
AVG_parahippocampal_thickavg	ICC3	0.824	10.34	19	19	2.24E-06	0.653	0.915	v5.3	OASIS
AVG_parahippocampal_thickavg	ICC2	0.816	21.19	19	19	5.41E-09	0.262	0.934	v5.3 vs. v6.0	OASIS
AVG_parahippocampal_thickavg	ICC2	0.819	10.29	19	19	2.33E-06	0.650	0.912	v5.3 vs. v7.1	OASIS
AVG_parahippocampal_thickavg	ICC3	0.892	17.44	19	19	2.92E-08	0.779	0.948	v6.0	OASIS
AVG_parahippocampal_thickavg	ICC2	0.868	30.23	19	19	2.33E-10	0.382	0.954	v6.0 vs. v7.1	OASIS
AVG_parahippocampal_thickavg	ICC3	0.943	34.28	19	19	7.56E-11	0.881	0.973	v7.1	OASIS
AVG_parsopercularis_thickavg	ICC3	0.777	7.98	19	19	1.69E-05	0.573	0.891	v5.3	OASIS
AVG_parsopercularis_thickavg	ICC2	0.894	38.43	19	19	2.69E-11	0.454	0.964	v5.3 vs. v6.0	OASIS
AVG_parsopercularis_thickavg	ICC2	0.838	11.93	19	19	7.06E-07	0.681	0.921	v5.3 vs. v7.1	OASIS
AVG_parsopercularis_thickavg	ICC3	0.869	14.31	19	19	1.57E-07	0.737	0.938	v6.0	OASIS
AVG_parsopercularis_thickavg	ICC2	0.898	22.42	19	19	3.30E-09	0.768	0.954	v6.0 vs. v7.1	OASIS
AVG_parsopercularis_thickavg	ICC3	0.840	11.52	19	19	9.40E-07	0.683	0.923	v7.1	OASIS
AVG_parsorbitalis_thickavg	ICC3	0.807	9.39	19	19	4.82E-06	0.625	0.906	v5.3	OASIS
AVG_parsorbitalis_thickavg	ICC2	0.906	20.36	19	19	7.68E-09	0.810	0.955	v5.3 vs. v6.0	OASIS
AVG_parsorbitalis_thickavg	ICC2	0.878	14.69	19	19	1.25E-07	0.752	0.942	v5.3 vs. v7.1	OASIS
AVG_parsorbitalis_thickavg	ICC3	0.913	22.01	19	19	3.88E-09	0.821	0.959	v6.0	OASIS
AVG_parsorbitalis_thickavg	ICC2	0.909	21.95	19	19	3.98E-09	0.813	0.957	v6.0 vs. v7.1	OASIS
AVG_parsorbitalis_thickavg	ICC3	0.862	13.53	19	19	2.49E-07	0.724	0.934	v7.1	OASIS
AVG_parstriangularis_thickavg	ICC3	0.705	5.79	19	19	1.78E-04	0.455	0.852	v5.3	OASIS
AVG_parstriangularis_thickavg	ICC2	0.782	12.86	19	19	3.81E-07	0.401	0.909	v5.3 vs. v6.0	OASIS
AVG_parstriangularis_thickavg	ICC2	0.759	7.78	19	19	2.05E-05	0.545	0.880	v5.3 vs. v7.1	OASIS
AVG_parstriangularis_thickavg	ICC3	0.856	12.90	19	19	3.71E-07	0.712	0.931	v6.0	OASIS
AVG_parstriangularis_thickavg	ICC2	0.872	17.93	19	19	2.31E-08	0.708	0.942	v6.0 vs. v7.1	OASIS
AVG_parstriangularis_thickavg	ICC3	0.810	9.51	19	19	4.35E-06	0.629	0.908	v7.1	OASIS
AVG_pericalcarine_thickavg	ICC3	0.913	21.89	19	19	4.08E-09	0.820	0.959	v5.3	OASIS
AVG_pericalcarine_thickavg	ICC2	0.727	14.86	19	19	1.14E-07	0.088	0.899	v5.3 vs. v6.0	OASIS
AVG_pericalcarine_thickavg	ICC2	0.828	11.27	19	19	1.12E-06	0.663	0.916	v5.3 vs. v7.1	OASIS
AVG_pericalcarine_thickavg	ICC3	0.781	8.14	19	19	1.46E-05	0.579	0.893	v6.0	OASIS
AVG_pericalcarine_thickavg	ICC2	0.855	25.72	19	19	9.85E-10	0.387	0.948	v6.0 vs. v7.1	OASIS
AVG_pericalcarine_thickavg	ICC3	0.911	21.47	19	19	4.84E-09	0.817	0.958	v7.1	OASIS
AVG_postcentral_thickavg	ICC3	0.941	32.97	19	19	1.07E-10	0.877	0.972	v5.3	OASIS
AVG_postcentral_thickavg	ICC2	0.821	32.35	19	19	1.27E-10	0.095	0.943	v5.3 vs. v6.0	OASIS
AVG_postcentral_thickavg	ICC2	0.872	39.80	19	19	1.96E-11	0.247	0.959	v5.3 vs. v7.1	OASIS
AVG_postcentral_thickavg	ICC3	0.959	47.82	19	19	3.67E-12	0.913	0.981	v6.0	OASIS
AVG_postcentral_thickavg	ICC2	0.981	157.25	19	19	5.76E-17	0.937	0.993	v6.0 vs. v7.1	OASIS

AVG_postcentral_thickavg	ICC3	0.919	23.76	19	19	1.98E-09	0.833	0.962	v7.1	OASIS
AVG_posteriorcingulate_thickavg	ICC3	0.763	7.43	19	19	2.90E-05	0.548	0.883	v5.3	OASIS
AVG_posteriorcingulate_thickavg	ICC2	0.865	27.90	19	19	4.78E-10	0.416	0.952	v5.3 vs. v6.0	OASIS
AVG_posteriorcingulate_thickavg	ICC2	0.384	10.80	19	19	1.58E-06	-0.048	0.718	v5.3 vs. v7.1	OASIS
AVG_posteriorcingulate_thickavg	ICC3	0.754	7.12	19	19	3.99E-05	0.533	0.878	v6.0	OASIS
AVG_posteriorcingulate_thickavg	ICC2	0.536	21.13	19	19	5.55E-09	-0.038	0.826	v6.0 vs. v7.1	OASIS
AVG_posteriorcingulate_thickavg	ICC3	0.858	13.08	19	19	3.31E-07	0.716	0.932	v7.1	OASIS
AVG_precentral_thickavg	ICC3	0.913	21.95	19	19	3.97E-09	0.820	0.959	v5.3	OASIS
AVG_precentral_thickavg	ICC2	0.749	30.09	19	19	2.43E-10	-0.010	0.920	v5.3 vs. v6.0	OASIS
AVG_precentral_thickavg	ICC2	0.778	24.81	19	19	1.36E-09	0.054	0.926	v5.3 vs. v7.1	OASIS
AVG_precentral_thickavg	ICC3	0.955	43.41	19	19	8.89E-12	0.905	0.979	v6.0	OASIS
AVG_precentral_thickavg	ICC2	0.963	61.66	19	19	3.55E-13	0.916	0.983	v6.0 vs. v7.1	OASIS
AVG_precentral_thickavg	ICC3	0.822	10.22	19	19	2.46E-06	0.650	0.914	v7.1	OASIS
AVG_precuneus_thickavg	ICC3	0.863	13.64	19	19	2.33E-07	0.726	0.935	v5.3	OASIS
AVG_precuneus_thickavg	ICC2	0.884	37.65	19	19	3.23E-11	0.378	0.961	v5.3 vs. v6.0	OASIS
AVG_precuneus_thickavg	ICC2	0.888	16.94	19	19	3.74E-08	0.776	0.947	v5.3 vs. v7.1	OASIS
AVG_precuneus_thickavg	ICC3	0.925	25.54	19	19	1.05E-09	0.844	0.965	v6.0	OASIS
AVG_precuneus_thickavg	ICC2	0.899	31.43	19	19	1.65E-10	0.627	0.961	v6.0 vs. v7.1	OASIS
AVG_precuneus_thickavg	ICC3	0.944	34.40	19	19	7.32E-11	0.881	0.974	v7.1	OASIS
AVG_Putamen	ICC3	0.943	34.09	19	19	7.94E-11	0.880	0.973	v5.3	OASIS
AVG_Putamen	ICC2	0.399	23.04	19	19	2.60E-09	-0.023	0.738	v5.3 vs. v6.0	OASIS
AVG_Putamen	ICC2	0.411	21.86	19	19	4.12E-09	-0.026	0.747	v5.3 vs. v7.1	OASIS
AVG_Putamen	ICC3	0.979	93.48	19	19	7.48E-15	0.955	0.990	v6.0	OASIS
AVG_Putamen	ICC2	0.994	353.43	19	19	2.79E-20	0.986	0.997	v6.0 vs. v7.1	OASIS
AVG_Putamen	ICC3	0.981	101.87	19	19	3.36E-15	0.958	0.991	v7.1	OASIS
AVG_rostralanteriorcingulate_thickavg	ICC3	0.613	4.17	19	19	1.56E-03	0.316	0.801	v5.3	OASIS
AVG_rostralanteriorcingulate_thickavg	ICC2	0.694	8.18	19	19	1.40E-05	0.299	0.863	v5.3 vs. v6.0	OASIS
AVG_rostralanteriorcingulate_thickavg	ICC2	0.689	5.94	19	19	1.48E-04	0.431	0.843	v5.3 vs. v7.1	OASIS
AVG_rostralanteriorcingulate_thickavg	ICC3	0.658	4.85	19	19	5.91E-04	0.382	0.826	v6.0	OASIS
AVG_rostralanteriorcingulate_thickavg	ICC2	0.541	11.28	19	19	1.11E-06	-0.055	0.817	v6.0 vs. v7.1	OASIS
AVG_rostralanteriorcingulate_thickavg	ICC3	0.665	4.96	19	19	5.08E-04	0.392	0.830	v7.1	OASIS
AVG_rostralmiddlefrontal_thickavg	ICC3	0.835	11.15	19	19	1.23E-06	0.674	0.921	v5.3	OASIS
AVG_rostralmiddlefrontal_thickavg	ICC2	0.915	28.40	19	19	4.08E-10	0.791	0.962	v5.3 vs. v6.0	OASIS
AVG_rostralmiddlefrontal_thickavg	ICC2	0.781	8.54	19	19	1.01E-05	0.584	0.892	v5.3 vs. v7.1	OASIS
AVG_rostralmiddlefrontal_thickavg	ICC3	0.932	28.59	19	19	3.85E-10	0.859	0.968	v6.0	OASIS
AVG_rostralmiddlefrontal_thickavg	ICC2	0.920	23.03	19	19	2.61E-09	0.835	0.962	v6.0 vs. v7.1	OASIS
AVG_rostralmiddlefrontal_thickavg	ICC3	0.930	27.74	19	19	5.03E-10	0.855	0.967	v7.1	OASIS
AVG_superiorfrontal_thickavg	ICC3	0.831	10.84	19	19	1.54E-06	0.667	0.918	v5.3	OASIS
AVG_superiorfrontal_thickavg	ICC2	0.928	27.53	19	19	5.39E-10	0.852	0.966	v5.3 vs. v6.0	OASIS
AVG_superiorfrontal_thickavg	ICC2	0.885	16.38	19	19	4.99E-08	0.770	0.945	v5.3 vs. v7.1	OASIS
AVG_superiorfrontal_thickavg	ICC3	0.902	19.39	19	19	1.17E-08	0.799	0.954	v6.0	OASIS
AVG_superiorfrontal_thickavg	ICC2	0.951	38.18	19	19	2.85E-11	0.897	0.977	v6.0 vs. v7.1	OASIS
AVG_superiorfrontal_thickavg	ICC3	0.879	15.53	19	19	7.86E-08	0.755	0.942	v7.1	OASIS
AVG_superiorparietal_thickavg	ICC3	0.874	14.85	19	19	1.14E-07	0.745	0.940	v5.3	OASIS

AVG_superiorparietal_thickavg	ICC2	0.843	32.90	19	19	1.09E-10	0.175	0.949	v5.3 vs. v6.0	OASIS
AVG_superiorparietal_thickavg	ICC2	0.835	23.81	19	19	1.95E-09	0.305	0.942	v5.3 vs. v7.1	OASIS
AVG_superiorparietal_thickavg	ICC3	0.954	42.71	19	19	1.03E-11	0.903	0.979	v6.0	OASIS
AVG_superiorparietal_thickavg	ICC2	0.935	28.76	19	19	3.65E-10	0.865	0.970	v6.0 vs. v7.1	OASIS
AVG_superiorparietal_thickavg	ICC3	0.946	35.77	19	19	5.15E-11	0.886	0.975	v7.1	OASIS
AVG_superiortemporal_thickavg	ICC3	0.830	10.78	19	19	1.61E-06	0.665	0.918	v5.3	OASIS
AVG_superiortemporal_thickavg	ICC2	0.864	53.40	19	19	1.34E-12	0.098	0.959	v5.3 vs. v6.0	OASIS
AVG_superiortemporal_thickavg	ICC2	0.882	18.87	19	19	1.48E-08	0.742	0.946	v5.3 vs. v7.1	OASIS
AVG_superiortemporal_thickavg	ICC3	0.886	16.52	19	19	4.65E-08	0.768	0.946	v6.0	OASIS
AVG_superiortemporal_thickavg	ICC2	0.877	21.61	19	19	4.56E-09	0.660	0.948	v6.0 vs. v7.1	OASIS
AVG_superiortemporal_thickavg	ICC3	0.891	17.31	19	19	3.11E-08	0.777	0.948	v7.1	OASIS
AVG_supramarginal_thickavg	ICC3	0.919	23.80	19	19	1.95E-09	0.833	0.962	v5.3	OASIS
AVG_supramarginal_thickavg	ICC2	0.914	28.83	19	19	3.56E-10	0.781	0.963	v5.3 vs. v6.0	OASIS
AVG_supramarginal_thickavg	ICC2	0.900	18.57	19	19	1.70E-08	0.797	0.953	v5.3 vs. v7.1	OASIS
AVG_supramarginal_thickavg	ICC3	0.957	45.46	19	19	5.83E-12	0.909	0.980	v6.0	OASIS
AVG_supramarginal_thickavg	ICC2	0.902	34.69	19	19	6.78E-11	0.595	0.964	v6.0 vs. v7.1	OASIS
AVG_supramarginal_thickavg	ICC3	0.949	38.38	19	19	2.72E-11	0.893	0.976	v7.1	OASIS
AVG_temporalpole_thickavg	ICC3	0.593	3.91	19	19	2.30E-03	0.287	0.789	v5.3	OASIS
AVG_temporalpole_thickavg	ICC2	0.844	11.52	19	19	9.37E-07	0.692	0.925	v5.3 vs. v6.0	OASIS
AVG_temporalpole_thickavg	ICC2	0.702	7.78	19	19	2.05E-05	0.356	0.862	v5.3 vs. v7.1	OASIS
AVG_temporalpole_thickavg	ICC3	0.762	7.39	19	19	3.02E-05	0.546	0.883	v6.0	OASIS
AVG_temporalpole_thickavg	ICC2	0.800	15.52	19	19	7.87E-08	0.370	0.921	v6.0 vs. v7.1	OASIS
AVG_temporalpole_thickavg	ICC3	0.619	4.25	19	19	1.38E-03	0.324	0.804	v7.1	OASIS
AVG_Thalamus	ICC3	0.947	36.80	19	19	3.98E-11	0.889	0.975	v5.3	OASIS
AVG_Thalamus	ICC2	0.946	35.80	19	19	5.10E-11	0.888	0.975	v5.3 vs. v6.0	OASIS
AVG_Thalamus	ICC2	0.909	34.34	19	19	7.44E-11	0.671	0.965	v5.3 vs. v7.1	OASIS
AVG_Thalamus	ICC3	0.977	86.27	19	19	1.58E-14	0.951	0.989	v6.0	OASIS
AVG_Thalamus	ICC2	0.925	35.25	19	19	5.88E-11	0.788	0.968	v6.0 vs. v7.1	OASIS
AVG_Thalamus	ICC3	0.895	18.08	19	19	2.14E-08	0.786	0.950	v7.1	OASIS
AVG_Thickness	ICC3	0.839	11.43	19	19	9.99E-07	0.681	0.922	v5.3	OASIS
AVG_Thickness	ICC2	0.819	36.08	19	19	4.76E-11	0.062	0.943	v5.3 vs. v6.0	OASIS
AVG_Thickness	ICC2	0.870	15.58	19	19	7.62E-08	0.737	0.938	v5.3 vs. v7.1	OASIS
AVG_Thickness	ICC3	0.934	29.22	19	19	3.17E-10	0.862	0.969	v6.0	OASIS
AVG_Thickness	ICC2	0.857	26.39	19	19	7.84E-10	0.387	0.949	v6.0 vs. v7.1	OASIS
AVG_Thickness	ICC3	0.935	29.61	19	19	2.81E-10	0.864	0.969	v7.1	OASIS
AVG_transversetemporal_thickavg	ICC3	0.946	36.29	19	19	4.51E-11	0.887	0.975	v5.3	OASIS
AVG_transversetemporal_thickavg	ICC2	0.753	29.71	19	19	2.73E-10	-0.006	0.921	v5.3 vs. v6.0	OASIS
AVG_transversetemporal_thickavg	ICC2	0.808	26.74	19	19	6.98E-10	0.116	0.937	v5.3 vs. v7.1	OASIS
AVG_transversetemporal_thickavg	ICC3	0.922	24.67	19	19	1.42E-09	0.838	0.963	v6.0	OASIS
AVG_transversetemporal_thickavg	ICC2	0.949	48.05	19	19	3.51E-12	0.870	0.978	v6.0 vs. v7.1	OASIS
AVG_transversetemporal_thickavg	ICC3	0.970	65.96	19	19	1.90E-13	0.936	0.986	v7.1	OASIS
ICV	ICC3	0.998	844.21	19	19	7.33E-24	0.995	0.999	v5.3	OASIS
ICV	ICC2	0.999	2672.94	19	19	1.31E-28	0.998	1.000	v5.3 vs. v6.0	OASIS
ICV	ICC2	0.993	320.35	19	19	7.06E-20	0.982	0.997	v5.3 vs. v7.1	OASIS

ICV	ICC3	0.998	841.04	19	19	7.60E-24	0.995	0.999	v6.0	OASIS
ICV	ICC2	0.992	273.99	19	19	3.09E-19	0.983	0.996	v6.0 vs. v7.1	OASIS
ICV	ICC3	0.998	904.63	19	19	3.81E-24	0.995	0.999	v7.1	OASIS
LH_Accumbens	ICC3	0.834	11.04	19	19	1.32E-06	0.672	0.920	v5.3	OASIS
LH_Accumbens	ICC2	0.766	7.64	19	19	2.35E-05	0.560	0.884	v5.3 vs. v6.0	OASIS
LH_Accumbens	ICC2	0.706	7.97	19	19	1.71E-05	0.359	0.864	v5.3 vs. v7.1	OASIS
LH_Accumbens	ICC3	0.926	25.96	19	19	9.09E-10	0.846	0.965	v6.0	OASIS
LH_Accumbens	ICC2	0.850	15.20	19	19	9.41E-08	0.666	0.931	v6.0 vs. v7.1	OASIS
LH_Accumbens	ICC3	0.908	20.83	19	19	6.27E-09	0.811	0.957	v7.1	OASIS
LH_Amygdala	ICC3	0.831	10.84	19	19	1.54E-06	0.667	0.918	v5.3	OASIS
LH_Amygdala	ICC2	0.901	27.73	19	19	5.05E-10	0.712	0.959	v5.3 vs. v6.0	OASIS
LH_Amygdala	ICC2	0.842	11.47	19	19	9.71E-07	0.689	0.923	v5.3 vs. v7.1	OASIS
LH_Amygdala	ICC3	0.837	11.26	19	19	1.12E-06	0.677	0.921	v6.0	OASIS
LH_Amygdala	ICC2	0.912	23.45	19	19	2.23E-09	0.817	0.959	v6.0 vs. v7.1	OASIS
LH_Amygdala	ICC3	0.866	13.98	19	19	1.90E-07	0.731	0.936	v7.1	OASIS
LH_bankssts_surfavg	ICC3	0.969	63.16	19	19	2.84E-13	0.934	0.986	v5.3	OASIS
LH_bankssts_surfavg	ICC2	0.701	5.62	19	19	2.18E-04	0.451	0.849	v5.3 vs. v6.0	OASIS
LH_bankssts_surfavg	ICC2	0.598	3.87	19	19	2.48E-03	0.292	0.793	v5.3 vs. v7.1	OASIS
LH_bankssts_surfavg	ICC3	0.966	57.53	19	19	6.73E-13	0.927	0.984	v6.0	OASIS
LH_bankssts_surfavg	ICC2	0.936	29.50	19	19	2.91E-10	0.868	0.970	v6.0 vs. v7.1	OASIS
LH_bankssts_surfavg	ICC3	0.827	10.58	19	19	1.87E-06	0.660	0.916	v7.1	OASIS
LH_bankssts_thickavg	ICC3	0.775	7.88	19	19	1.86E-05	0.568	0.889	v5.3	OASIS
LH_bankssts_thickavg	ICC2	0.899	23.34	19	19	2.32E-09	0.764	0.955	v5.3 vs. v6.0	OASIS
LH_bankssts_thickavg	ICC2	0.912	21.49	19	19	4.79E-09	0.821	0.958	v5.3 vs. v7.1	OASIS
LH_bankssts_thickavg	ICC3	0.848	12.17	19	19	5.98E-07	0.698	0.927	v6.0	OASIS
LH_bankssts_thickavg	ICC2	0.890	27.57	19	19	5.32E-10	0.628	0.957	v6.0 vs. v7.1	OASIS
LH_bankssts_thickavg	ICC3	0.940	32.14	19	19	1.35E-10	0.874	0.972	v7.1	OASIS
LH_caudalanteriorcingulate_surfavg	ICC3	0.959	47.56	19	19	3.86E-12	0.913	0.981	v5.3	OASIS
LH_caudalanteriorcingulate_surfavg	ICC2	0.881	15.46	19	19	8.17E-08	0.761	0.943	v5.3 vs. v6.0	OASIS
LH_caudalanteriorcingulate_surfavg	ICC2	0.858	13.77	19	19	2.16E-07	0.718	0.932	v5.3 vs. v7.1	OASIS
LH_caudalanteriorcingulate_surfavg	ICC3	0.940	32.34	19	19	1.28E-10	0.874	0.972	v6.0	OASIS
LH_caudalanteriorcingulate_surfavg	ICC2	0.919	39.40	19	19	2.14E-11	0.692	0.969	v6.0 vs. v7.1	OASIS
LH_caudalanteriorcingulate_surfavg	ICC3	0.955	43.30	19	19	9.09E-12	0.905	0.979	v7.1	OASIS
LH_caudalanteriorcingulate_thickavg	ICC3	0.633	4.45	19	19	1.04E-03	0.345	0.812	v5.3	OASIS
LH_caudalanteriorcingulate_thickavg	ICC2	0.735	6.64	19	19	6.65E-05	0.509	0.868	v5.3 vs. v6.0	OASIS
LH_caudalanteriorcingulate_thickavg	ICC2	0.636	15.33	19	19	8.76E-08	-0.036	0.867	v5.3 vs. v7.1	OASIS
LH_caudalanteriorcingulate_thickavg	ICC3	0.861	13.40	19	19	2.71E-07	0.721	0.933	v6.0	OASIS
LH_caudalanteriorcingulate_thickavg	ICC2	0.525	7.51	19	19	2.67E-05	-0.038	0.796	v6.0 vs. v7.1	OASIS
LH_caudalanteriorcingulate_thickavg	ICC3	0.739	6.66	19	19	6.49E-05	0.509	0.871	v7.1	OASIS
LH_caudalmiddlefrontal_surfavg	ICC3	0.973	71.99	19	19	8.48E-14	0.942	0.987	v5.3	OASIS
LH_caudalmiddlefrontal_surfavg	ICC2	0.953	43.28	19	19	9.12E-12	0.901	0.978	v5.3 vs. v6.0	OASIS
LH_caudalmiddlefrontal_surfavg	ICC2	0.953	42.78	19	19	1.02E-11	0.903	0.978	v5.3 vs. v7.1	OASIS
LH_caudalmiddlefrontal_surfavg	ICC3	0.987	151.82	19	19	8.01E-17	0.972	0.994	v6.0	OASIS
LH_caudalmiddlefrontal_surfavg	ICC2	0.992	230.26	19	19	1.59E-18	0.982	0.996	v6.0 vs. v7.1	OASIS

LH_caudalmiddlefrontal_surfavg	ICC3	0.930	27.39	19	19	5.63E-10	0.853	0.967	v7.1	OASIS
LH_caudalmiddlefrontal_thickavg	ICC3	0.798	8.93	19	19	7.16E-06	0.609	0.902	v5.3	OASIS
LH_caudalmiddlefrontal_thickavg	ICC2	0.808	26.64	19	19	7.22E-10	0.114	0.936	v5.3 vs. v6.0	OASIS
LH_caudalmiddlefrontal_thickavg	ICC2	0.820	17.39	19	19	2.99E-08	0.416	0.930	v5.3 vs. v7.1	OASIS
LH_caudalmiddlefrontal_thickavg	ICC3	0.879	15.54	19	19	7.79E-08	0.755	0.942	v6.0	OASIS
LH_caudalmiddlefrontal_thickavg	ICC2	0.928	29.31	19	19	3.08E-10	0.848	0.966	v6.0 vs. v7.1	OASIS
LH_caudalmiddlefrontal_thickavg	ICC3	0.837	11.24	19	19	1.14E-06	0.677	0.921	v7.1	OASIS
LH_Caudate	ICC3	0.989	173.70	19	19	2.26E-17	0.975	0.995	v5.3	OASIS
LH_Caudate	ICC2	0.958	123.73	19	19	5.46E-16	0.615	0.987	v5.3 vs. v6.0	OASIS
LH_Caudate	ICC2	0.979	103.30	19	19	2.95E-15	0.954	0.990	v5.3 vs. v7.1	OASIS
LH_Caudate	ICC3	0.983	116.01	19	19	9.97E-16	0.963	0.992	v6.0	OASIS
LH_Caudate	ICC2	0.976	173.08	19	19	2.34E-17	0.832	0.992	v6.0 vs. v7.1	OASIS
LH_Caudate	ICC3	0.986	146.12	19	19	1.15E-16	0.971	0.994	v7.1	OASIS
LH_cuneus_surfavg	ICC3	0.962	51.22	19	19	1.96E-12	0.919	0.982	v5.3	OASIS
LH_cuneus_surfavg	ICC2	0.962	60.34	19	19	4.33E-13	0.910	0.983	v5.3 vs. v6.0	OASIS
LH_cuneus_surfavg	ICC2	0.920	67.72	19	19	1.49E-13	0.379	0.975	v5.3 vs. v7.1	OASIS
LH_cuneus_surfavg	ICC3	0.990	196.91	19	19	6.95E-18	0.978	0.995	v6.0	OASIS
LH_cuneus_surfavg	ICC2	0.963	115.87	19	19	1.01E-15	0.750	0.988	v6.0 vs. v7.1	OASIS
LH_cuneus_surfavg	ICC3	0.992	254.32	19	19	6.24E-19	0.983	0.996	v7.1	OASIS
LH_cuneus_thickavg	ICC3	0.919	23.69	19	19	2.04E-09	0.832	0.962	v5.3	OASIS
LH_cuneus_thickavg	ICC2	0.906	47.06	19	19	4.25E-12	0.445	0.969	v5.3 vs. v6.0	OASIS
LH_cuneus_thickavg	ICC2	0.917	23.31	19	19	2.35E-09	0.830	0.961	v5.3 vs. v7.1	OASIS
LH_cuneus_thickavg	ICC3	0.937	30.95	19	19	1.89E-10	0.869	0.971	v6.0	OASIS
LH_cuneus_thickavg	ICC2	0.930	48.99	19	19	2.94E-12	0.695	0.974	v6.0 vs. v7.1	OASIS
LH_cuneus_thickavg	ICC3	0.970	65.47	19	19	2.04E-13	0.936	0.986	v7.1	OASIS
LH_entorhinal_surfavg	ICC3	0.821	10.18	19	19	2.53E-06	0.649	0.913	v5.3	OASIS
LH_entorhinal_surfavg	ICC2	0.489	4.07	19	19	1.80E-03	0.091	0.737	v5.3 vs. v6.0	OASIS
LH_entorhinal_surfavg	ICC2	0.494	3.26	19	19	6.71E-03	0.171	0.725	v5.3 vs. v7.1	OASIS
LH_entorhinal_surfavg	ICC3	0.931	28.09	19	19	4.50E-10	0.857	0.968	v6.0	OASIS
LH_entorhinal_surfavg	ICC2	0.752	8.44	19	19	1.10E-05	0.501	0.881	v6.0 vs. v7.1	OASIS
LH_entorhinal_surfavg	ICC3	0.893	17.63	19	19	2.66E-08	0.781	0.949	v7.1	OASIS
LH_entorhinal_thickavg	ICC3	0.882	15.90	19	19	6.43E-08	0.760	0.944	v5.3	OASIS
LH_entorhinal_thickavg	ICC2	0.751	9.89	19	19	3.19E-06	0.418	0.889	v5.3 vs. v6.0	OASIS
LH_entorhinal_thickavg	ICC2	0.761	7.70	19	19	2.23E-05	0.550	0.881	v5.3 vs. v7.1	OASIS
LH_entorhinal_thickavg	ICC3	0.870	14.35	19	19	1.53E-07	0.737	0.938	v6.0	OASIS
LH_entorhinal_thickavg	ICC2	0.698	23.41	19	19	2.26E-09	-0.026	0.899	v6.0 vs. v7.1	OASIS
LH_entorhinal_thickavg	ICC3	0.846	12.03	19	19	6.59E-07	0.695	0.926	v7.1	OASIS
LH_frontalpole_surfavg	ICC3	0.798	8.91	19	19	7.27E-06	0.608	0.902	v5.3	OASIS
LH_frontalpole_surfavg	ICC2	0.368	5.97	19	19	1.42E-04	-0.070	0.690	v5.3 vs. v6.0	OASIS
LH_frontalpole_surfavg	ICC2	0.237	5.92	19	19	1.51E-04	-0.050	0.562	v5.3 vs. v7.1	OASIS
LH_frontalpole_surfavg	ICC3	0.911	21.52	19	19	4.73E-09	0.817	0.958	v6.0	OASIS
LH_frontalpole_surfavg	ICC2	0.773	32.22	19	19	1.32E-10	0.006	0.928	v6.0 vs. v7.1	OASIS
LH_frontalpole_surfavg	ICC3	0.855	12.83	19	19	3.86E-07	0.711	0.931	v7.1	OASIS
LH_frontalpole_thickavg	ICC3	0.728	6.36	19	19	9.09E-05	0.491	0.865	v5.3	OASIS

LH_frontalpole_thickavg	ICC2	0.745	10.71	19	19	1.69E-06	0.338	0.891	v5.3 vs. v6.0	OASIS
LH_frontalpole_thickavg	ICC2	0.714	8.88	19	19	7.42E-06	0.327	0.873	v5.3 vs. v7.1	OASIS
LH_frontalpole_thickavg	ICC3	0.759	7.30	19	19	3.30E-05	0.542	0.881	v6.0	OASIS
LH_frontalpole_thickavg	ICC2	0.752	6.76	19	19	5.87E-05	0.527	0.878	v6.0 vs. v7.1	OASIS
LH_frontalpole_thickavg	ICC3	0.850	12.32	19	19	5.41E-07	0.701	0.928	v7.1	OASIS
LH_fusiform_surfavg	ICC3	0.968	61.20	19	19	3.81E-13	0.932	0.985	v5.3	OASIS
LH_fusiform_surfavg	ICC2	0.830	20.96	19	19	5.96E-09	0.356	0.937	v5.3 vs. v6.0	OASIS
LH_fusiform_surfavg	ICC2	0.856	20.05	19	19	8.76E-09	0.560	0.942	v5.3 vs. v7.1	OASIS
LH_fusiform_surfavg	ICC3	0.986	138.31	19	19	1.92E-16	0.969	0.993	v6.0	OASIS
LH_fusiform_surfavg	ICC2	0.977	102.20	19	19	3.26E-15	0.944	0.990	v6.0 vs. v7.1	OASIS
LH_fusiform_surfavg	ICC3	0.992	234.81	19	19	1.32E-18	0.982	0.996	v7.1	OASIS
LH_fusiform_thickavg	ICC3	0.622	4.29	19	19	1.30E-03	0.329	0.806	v5.3	OASIS
LH_fusiform_thickavg	ICC2	0.697	12.07	19	19	6.40E-07	0.095	0.883	v5.3 vs. v6.0	OASIS
LH_fusiform_thickavg	ICC2	0.774	8.73	19	19	8.49E-06	0.563	0.890	v5.3 vs. v7.1	OASIS
LH_fusiform_thickavg	ICC3	0.750	7.00	19	19	4.51E-05	0.527	0.876	v6.0	OASIS
LH_fusiform_thickavg	ICC2	0.765	11.43	19	19	1.00E-06	0.389	0.900	v6.0 vs. v7.1	OASIS
LH_fusiform_thickavg	ICC3	0.852	12.47	19	19	4.89E-07	0.704	0.929	v7.1	OASIS
LH_Hippocampus	ICC3	0.926	26.19	19	19	8.38E-10	0.847	0.965	v5.3	OASIS
LH_Hippocampus	ICC2	0.876	18.19	19	19	2.04E-08	0.724	0.943	v5.3 vs. v6.0	OASIS
LH_Hippocampus	ICC2	0.902	18.95	19	19	1.43E-08	0.801	0.953	v5.3 vs. v7.1	OASIS
LH_Hippocampus	ICC3	0.968	61.40	19	19	3.69E-13	0.932	0.985	v6.0	OASIS
LH_Hippocampus	ICC2	0.899	32.11	19	19	1.36E-10	0.614	0.962	v6.0 vs. v7.1	OASIS
LH_Hippocampus	ICC3	0.963	52.97	19	19	1.44E-12	0.921	0.983	v7.1	OASIS
LH_inferiorparietal_surfavg	ICC3	0.972	70.52	19	19	1.03E-13	0.940	0.987	v5.3	OASIS
LH_inferiorparietal_surfavg	ICC2	0.824	15.61	19	19	7.51E-08	0.513	0.927	v5.3 vs. v6.0	OASIS
LH_inferiorparietal_surfavg	ICC2	0.849	17.71	19	19	2.56E-08	0.585	0.936	v5.3 vs. v7.1	OASIS
LH_inferiorparietal_surfavg	ICC3	0.973	73.80	19	19	6.74E-14	0.943	0.988	v6.0	OASIS
LH_inferiorparietal_surfavg	ICC2	0.990	217.46	19	19	2.73E-18	0.980	0.996	v6.0 vs. v7.1	OASIS
LH_inferiorparietal_surfavg	ICC3	0.936	30.24	19	19	2.33E-10	0.866	0.970	v7.1	OASIS
LH_inferiorparietal_thickavg	ICC3	0.860	13.29	19	19	2.89E-07	0.720	0.933	v5.3	OASIS
LH_inferiorparietal_thickavg	ICC2	0.924	25.04	19	19	1.25E-09	0.844	0.964	v5.3 vs. v6.0	OASIS
LH_inferiorparietal_thickavg	ICC2	0.905	21.82	19	19	4.19E-09	0.804	0.955	v5.3 vs. v7.1	OASIS
LH_inferiorparietal_thickavg	ICC3	0.900	19.10	19	19	1.34E-08	0.796	0.953	v6.0	OASIS
LH_inferiorparietal_thickavg	ICC2	0.889	21.72	19	19	4.36E-09	0.729	0.951	v6.0 vs. v7.1	OASIS
LH_inferiorparietal_thickavg	ICC3	0.922	24.67	19	19	1.42E-09	0.838	0.963	v7.1	OASIS
LH_inferiortemporal_surfavg	ICC3	0.981	106.71	19	19	2.18E-15	0.960	0.991	v5.3	OASIS
LH_inferiortemporal_surfavg	ICC2	0.928	46.76	19	19	4.50E-12	0.699	0.974	v5.3 vs. v6.0	OASIS
LH_inferiortemporal_surfavg	ICC2	0.880	35.62	19	19	5.34E-11	0.373	0.959	v5.3 vs. v7.1	OASIS
LH_inferiortemporal_surfavg	ICC3	0.991	220.33	19	19	2.41E-18	0.981	0.996	v6.0	OASIS
LH_inferiortemporal_surfavg	ICC2	0.983	216.43	19	19	2.85E-18	0.910	0.994	v6.0 vs. v7.1	OASIS
LH_inferiortemporal_surfavg	ICC3	0.990	206.03	19	19	4.54E-18	0.979	0.996	v7.1	OASIS
LH_inferiortemporal_thickavg	ICC3	0.753	7.10	19	19	4.05E-05	0.532	0.878	v5.3	OASIS
LH_inferiortemporal_thickavg	ICC2	0.780	9.00	19	19	6.69E-06	0.572	0.893	v5.3 vs. v6.0	OASIS
LH_inferiortemporal_thickavg	ICC2	0.853	12.26	19	19	5.65E-07	0.708	0.930	v5.3 vs. v7.1	OASIS

LH_inferiortemporal_thickavg	ICC3	0.643	4.60	19	19	8.31E-04	0.360	0.818	v6.0	OASIS
LH_inferiortemporal_thickavg	ICC2	0.780	10.21	19	19	2.48E-06	0.528	0.898	v6.0 vs. v7.1	OASIS
LH_inferiortemporal_thickavg	ICC3	0.881	15.84	19	19	6.62E-08	0.759	0.943	v7.1	OASIS
LH_insula_surfavg	ICC3	0.956	43.96	19	19	7.91E-12	0.906	0.979	v5.3	OASIS
LH_insula_surfavg	ICC2	0.508	19.97	19	19	9.09E-09	-0.038	0.809	v5.3 vs. v6.0	OASIS
LH_insula_surfavg	ICC2	0.577	22.28	19	19	3.49E-09	-0.039	0.847	v5.3 vs. v7.1	OASIS
LH_insula_surfavg	ICC3	0.763	7.45	19	19	2.85E-05	0.549	0.883	v6.0	OASIS
LH_insula_surfavg	ICC2	0.858	13.42	19	19	2.68E-07	0.719	0.931	v6.0 vs. v7.1	OASIS
LH_insula_surfavg	ICC3	0.829	10.72	19	19	1.68E-06	0.663	0.917	v7.1	OASIS
LH_insula_thickavg	ICC3	0.777	7.96	19	19	1.72E-05	0.572	0.891	v5.3	OASIS
LH_insula_thickavg	ICC2	0.655	6.03	19	19	1.34E-04	0.334	0.831	v5.3 vs. v6.0	OASIS
LH_insula_thickavg	ICC2	0.679	6.39	19	19	8.77E-05	0.379	0.842	v5.3 vs. v7.1	OASIS
LH_insula_thickavg	ICC3	0.737	6.60	19	19	6.92E-05	0.506	0.869	v6.0	OASIS
LH_insula_thickavg	ICC2	0.612	21.31	19	19	5.15E-09	-0.041	0.863	v6.0 vs. v7.1	OASIS
LH_insula_thickavg	ICC3	0.894	17.96	19	19	2.27E-08	0.785	0.950	v7.1	OASIS
LH_isthmuscingulate_surfavg	ICC3	0.955	43.88	19	19	8.06E-12	0.906	0.979	v5.3	OASIS
LH_isthmuscingulate_surfavg	ICC2	0.876	33.25	19	19	9.93E-11	0.389	0.958	v5.3 vs. v6.0	OASIS
LH_isthmuscingulate_surfavg	ICC2	0.873	25.36	19	19	1.12E-09	0.538	0.952	v5.3 vs. v7.1	OASIS
LH_isthmuscingulate_surfavg	ICC3	0.986	139.11	19	19	1.82E-16	0.969	0.993	v6.0	OASIS
LH_isthmuscingulate_surfavg	ICC2	0.978	88.30	19	19	1.27E-14	0.953	0.990	v6.0 vs. v7.1	OASIS
LH_isthmuscingulate_surfavg	ICC3	0.934	29.44	19	19	2.96E-10	0.863	0.969	v7.1	OASIS
LH_isthmuscingulate_thickavg	ICC3	0.815	9.82	19	19	3.37E-06	0.638	0.910	v5.3	OASIS
LH_isthmuscingulate_thickavg	ICC2	0.846	26.74	19	19	6.97E-10	0.301	0.946	v5.3 vs. v6.0	OASIS
LH_isthmuscingulate_thickavg	ICC2	0.518	18.69	19	19	1.61E-08	-0.041	0.814	v5.3 vs. v7.1	OASIS
LH_isthmuscingulate_thickavg	ICC3	0.918	23.38	19	19	2.28E-09	0.830	0.961	v6.0	OASIS
LH_isthmuscingulate_thickavg	ICC2	0.720	38.45	19	19	2.67E-11	-0.024	0.912	v6.0 vs. v7.1	OASIS
LH_isthmuscingulate_thickavg	ICC3	0.909	20.97	19	19	5.92E-09	0.813	0.957	v7.1	OASIS
LH_lateraloccipital_surfavg	ICC3	0.980	96.69	19	19	5.46E-15	0.956	0.991	v5.3	OASIS
LH_lateraloccipital_surfavg	ICC2	0.759	21.51	19	19	4.74E-09	0.049	0.918	v5.3 vs. v6.0	OASIS
LH_lateraloccipital_surfavg	ICC2	0.683	17.23	19	19	3.23E-08	-0.016	0.888	v5.3 vs. v7.1	OASIS
LH_lateraloccipital_surfavg	ICC3	0.993	270.20	19	19	3.52E-19	0.984	0.997	v6.0	OASIS
LH_lateraloccipital_surfavg	ICC2	0.978	177.21	19	19	1.87E-17	0.872	0.993	v6.0 vs. v7.1	OASIS
LH_lateraloccipital_surfavg	ICC3	0.992	241.20	19	19	1.03E-18	0.982	0.996	v7.1	OASIS
LH_lateraloccipital_thickavg	ICC3	0.805	9.26	19	19	5.39E-06	0.620	0.905	v5.3	OASIS
LH_lateraloccipital_thickavg	ICC2	0.858	20.26	19	19	7.99E-09	0.567	0.943	v5.3 vs. v6.0	OASIS
LH_lateraloccipital_thickavg	ICC2	0.915	21.92	19	19	4.02E-09	0.826	0.960	v5.3 vs. v7.1	OASIS
LH_lateraloccipital_thickavg	ICC3	0.919	23.63	19	19	2.08E-09	0.832	0.962	v6.0	OASIS
LH_lateraloccipital_thickavg	ICC2	0.872	32.44	19	19	1.24E-10	0.374	0.956	v6.0 vs. v7.1	OASIS
LH_lateraloccipital_thickavg	ICC3	0.924	25.31	19	19	1.14E-09	0.842	0.964	v7.1	OASIS
LH_lateralorbitofrontal_surfavg	ICC3	0.920	24.08	19	19	1.76E-09	0.835	0.962	v5.3	OASIS
LH_lateralorbitofrontal_surfavg	ICC2	0.907	44.68	19	19	6.82E-12	0.490	0.969	v5.3 vs. v6.0	OASIS
LH_lateralorbitofrontal_surfavg	ICC2	0.732	22.10	19	19	3.74E-09	0.002	0.910	v5.3 vs. v7.1	OASIS
LH_lateralorbitofrontal_surfavg	ICC3	0.951	39.70	19	19	2.00E-11	0.896	0.977	v6.0	OASIS
LH_lateralorbitofrontal_surfavg	ICC2	0.899	57.22	19	19	7.06E-13	0.266	0.969	v6.0 vs. v7.1	OASIS

LH_lateralorbitofrontal_surfavg	ICC3	0.956	44.44	19	19	7.18E-12	0.907	0.979	v7.1	OASIS
LH_lateralorbitofrontal_thickavg	ICC3	0.660	4.89	19	19	5.63E-04	0.385	0.827	v5.3	OASIS
LH_lateralorbitofrontal_thickavg	ICC2	0.656	12.35	19	19	5.32E-07	0.006	0.869	v5.3 vs. v6.0	OASIS
LH_lateralorbitofrontal_thickavg	ICC2	0.645	6.07	19	19	1.27E-04	0.299	0.828	v5.3 vs. v7.1	OASIS
LH_lateralorbitofrontal_thickavg	ICC3	0.735	6.54	19	19	7.46E-05	0.502	0.868	v6.0	OASIS
LH_lateralorbitofrontal_thickavg	ICC2	0.798	12.79	19	19	3.97E-07	0.485	0.913	v6.0 vs. v7.1	OASIS
LH_lateralorbitofrontal_thickavg	ICC3	0.829	10.67	19	19	1.74E-06	0.662	0.917	v7.1	OASIS
LH_LateralVentricle	ICC3	0.997	748.63	19	19	2.29E-23	0.994	0.999	v5.3	OASIS
LH_LateralVentricle	ICC2	0.993	2146.26	19	19	1.05E-27	0.638	0.998	v5.3 vs. v6.0	OASIS
LH_LateralVentricle	ICC2	0.991	2498.35	19	19	2.48E-28	0.510	0.998	v5.3 vs. v7.1	OASIS
LH_LateralVentricle	ICC3	0.998	1324.26	19	19	1.03E-25	0.997	0.999	v6.0	OASIS
LH_LateralVentricle	ICC2	0.999	3163.56	19	19	2.64E-29	0.999	1.000	v6.0 vs. v7.1	OASIS
LH_LateralVentricle	ICC3	0.999	1383.65	19	19	6.76E-26	0.997	0.999	v7.1	OASIS
LH_lingual_surfavg	ICC3	0.973	72.92	19	19	7.52E-14	0.942	0.987	v5.3	OASIS
LH_lingual_surfavg	ICC2	0.915	21.70	19	19	4.40E-09	0.825	0.960	v5.3 vs. v6.0	OASIS
LH_lingual_surfavg	ICC2	0.907	20.72	19	19	6.59E-09	0.812	0.956	v5.3 vs. v7.1	OASIS
LH_lingual_surfavg	ICC3	0.990	195.73	19	19	7.36E-18	0.978	0.995	v6.0	OASIS
LH_lingual_surfavg	ICC2	0.981	213.95	19	19	3.18E-18	0.867	0.994	v6.0 vs. v7.1	OASIS
LH_lingual_surfavg	ICC3	0.991	211.56	19	19	3.54E-18	0.980	0.996	v7.1	OASIS
LH_lingual_thickavg	ICC3	0.843	11.71	19	19	8.18E-07	0.688	0.924	v5.3	OASIS
LH_lingual_thickavg	ICC2	0.736	14.57	19	19	1.34E-07	0.125	0.902	v5.3 vs. v6.0	OASIS
LH_lingual_thickavg	ICC2	0.865	14.77	19	19	1.20E-07	0.728	0.935	v5.3 vs. v7.1	OASIS
LH_lingual_thickavg	ICC3	0.854	12.71	19	19	4.18E-07	0.709	0.930	v6.0	OASIS
LH_lingual_thickavg	ICC2	0.849	24.40	19	19	1.57E-09	0.384	0.945	v6.0 vs. v7.1	OASIS
LH_lingual_thickavg	ICC3	0.893	17.70	19	19	2.57E-08	0.782	0.949	v7.1	OASIS
LH_medialorbitofrontal_surfavg	ICC3	0.762	7.41	19	19	2.95E-05	0.547	0.883	v5.3	OASIS
LH_medialorbitofrontal_surfavg	ICC2	0.682	5.11	19	19	4.18E-04	0.416	0.840	v5.3 vs. v6.0	OASIS
LH_medialorbitofrontal_surfavg	ICC2	0.467	8.11	19	19	1.49E-05	-0.063	0.767	v5.3 vs. v7.1	OASIS
LH_medialorbitofrontal_surfavg	ICC3	0.891	17.28	19	19	3.16E-08	0.777	0.948	v6.0	OASIS
LH_medialorbitofrontal_surfavg	ICC2	0.519	12.31	19	19	5.44E-07	-0.055	0.808	v6.0 vs. v7.1	OASIS
LH_medialorbitofrontal_surfavg	ICC3	0.902	19.44	19	19	1.14E-08	0.799	0.954	v7.1	OASIS
LH_medialorbitofrontal_thickavg	ICC3	0.679	5.24	19	19	3.54E-04	0.414	0.838	v5.3	OASIS
LH_medialorbitofrontal_thickavg	ICC2	0.763	13.09	19	19	3.29E-07	0.292	0.905	v5.3 vs. v6.0	OASIS
LH_medialorbitofrontal_thickavg	ICC2	0.746	6.86	19	19	5.24E-05	0.526	0.874	v5.3 vs. v7.1	OASIS
LH_medialorbitofrontal_thickavg	ICC3	0.796	8.79	19	19	8.08E-06	0.604	0.900	v6.0	OASIS
LH_medialorbitofrontal_thickavg	ICC2	0.809	18.23	19	19	2.00E-08	0.318	0.928	v6.0 vs. v7.1	OASIS
LH_medialorbitofrontal_thickavg	ICC3	0.770	7.69	19	19	2.25E-05	0.560	0.887	v7.1	OASIS
LH_middletemporal_surfavg	ICC3	0.976	84.07	19	19	2.01E-14	0.950	0.989	v5.3	OASIS
LH_middletemporal_surfavg	ICC2	0.871	48.50	19	19	3.23E-12	0.156	0.960	v5.3 vs. v6.0	OASIS
LH_middletemporal_surfavg	ICC2	0.808	50.19	19	19	2.36E-12	0.007	0.943	v5.3 vs. v7.1	OASIS
LH_middletemporal_surfavg	ICC3	0.981	104.72	19	19	2.60E-15	0.959	0.991	v6.0	OASIS
LH_middletemporal_surfavg	ICC2	0.978	199.72	19	19	6.08E-18	0.836	0.993	v6.0 vs. v7.1	OASIS
LH_middletemporal_surfavg	ICC3	0.988	165.21	19	19	3.62E-17	0.974	0.994	v7.1	OASIS
LH_middletemporal_thickavg	ICC3	0.716	6.05	19	19	1.30E-04	0.472	0.858	v5.3	OASIS

LH_middletemporal_thickavg	ICC2	0.861	14.92	19	19	1.10E-07	0.717	0.934	v5.3 vs. v6.0	OASIS
LH_middletemporal_thickavg	ICC2	0.821	10.76	19	19	1.63E-06	0.651	0.913	v5.3 vs. v7.1	OASIS
LH_middletemporal_thickavg	ICC3	0.648	4.67	19	19	7.53E-04	0.366	0.820	v6.0	OASIS
LH_middletemporal_thickavg	ICC2	0.775	12.12	19	19	6.20E-07	0.405	0.905	v6.0 vs. v7.1	OASIS
LH_middletemporal_thickavg	ICC3	0.864	13.71	19	19	2.23E-07	0.727	0.935	v7.1	OASIS
LH_Pallidum	ICC3	0.923	24.87	19	19	1.33E-09	0.840	0.964	v5.3	OASIS
LH_Pallidum	ICC2	0.657	5.98	19	19	1.41E-04	0.342	0.831	v5.3 vs. v6.0	OASIS
LH_Pallidum	ICC2	0.620	7.69	19	19	2.23E-05	0.095	0.836	v5.3 vs. v7.1	OASIS
LH_Pallidum	ICC3	0.901	19.25	19	19	1.25E-08	0.798	0.953	v6.0	OASIS
LH_Pallidum	ICC2	0.839	16.19	19	19	5.51E-08	0.578	0.931	v6.0 vs. v7.1	OASIS
LH_Pallidum	ICC3	0.856	12.90	19	19	3.70E-07	0.712	0.931	v7.1	OASIS
LH_paracentral_surfavg	ICC3	0.958	46.65	19	19	4.60E-12	0.911	0.980	v5.3	OASIS
LH_paracentral_surfavg	ICC2	0.816	10.52	19	19	1.95E-06	0.642	0.911	v5.3 vs. v6.0	OASIS
LH_paracentral_surfavg	ICC2	0.831	10.83	19	19	1.55E-06	0.670	0.918	v5.3 vs. v7.1	OASIS
LH_paracentral_surfavg	ICC3	0.977	84.24	19	19	1.97E-14	0.950	0.989	v6.0	OASIS
LH_paracentral_surfavg	ICC2	0.984	141.57	19	19	1.54E-16	0.962	0.993	v6.0 vs. v7.1	OASIS
LH_paracentral_surfavg	ICC3	0.922	24.60	19	19	1.46E-09	0.838	0.963	v7.1	OASIS
LH_paracentral_thickavg	ICC3	0.860	13.28	19	19	2.91E-07	0.719	0.933	v5.3	OASIS
LH_paracentral_thickavg	ICC2	0.711	20.96	19	19	5.96E-09	-0.012	0.902	v5.3 vs. v6.0	OASIS
LH_paracentral_thickavg	ICC2	0.756	17.57	19	19	2.74E-08	0.107	0.913	v5.3 vs. v7.1	OASIS
LH_paracentral_thickavg	ICC3	0.876	15.10	19	19	9.95E-08	0.749	0.941	v6.0	OASIS
LH_paracentral_thickavg	ICC2	0.952	46.60	19	19	4.65E-12	0.890	0.978	v6.0 vs. v7.1	OASIS
LH_paracentral_thickavg	ICC3	0.880	15.70	19	19	7.16E-08	0.757	0.943	v7.1	OASIS
LH_parahippocampal_surfavg	ICC3	0.912	21.84	19	19	4.16E-09	0.819	0.959	v5.3	OASIS
LH_parahippocampal_surfavg	ICC2	0.548	4.57	19	19	8.71E-04	0.170	0.772	v5.3 vs. v6.0	OASIS
LH_parahippocampal_surfavg	ICC2	0.457	4.81	19	19	6.21E-04	-0.016	0.736	v5.3 vs. v7.1	OASIS
LH_parahippocampal_surfavg	ICC3	0.917	23.10	19	19	2.54E-09	0.828	0.961	v6.0	OASIS
LH_parahippocampal_surfavg	ICC2	0.772	9.74	19	19	3.59E-06	0.513	0.893	v6.0 vs. v7.1	OASIS
LH_parahippocampal_surfavg	ICC3	0.904	19.79	19	19	9.81E-09	0.803	0.954	v7.1	OASIS
LH_parahippocampal_thickavg	ICC3	0.829	10.71	19	19	1.69E-06	0.663	0.917	v5.3	OASIS
LH_parahippocampal_thickavg	ICC2	0.810	16.32	19	19	5.14E-08	0.395	0.925	v5.3 vs. v6.0	OASIS
LH_parahippocampal_thickavg	ICC2	0.856	12.96	19	19	3.57E-07	0.716	0.931	v5.3 vs. v7.1	OASIS
LH_parahippocampal_thickavg	ICC3	0.957	45.76	19	19	5.49E-12	0.910	0.980	v6.0	OASIS
LH_parahippocampal_thickavg	ICC2	0.882	33.16	19	19	1.02E-10	0.440	0.959	v6.0 vs. v7.1	OASIS
LH_parahippocampal_thickavg	ICC3	0.953	41.27	19	19	1.41E-11	0.900	0.978	v7.1	OASIS
LH_parsopercularis_surfavg	ICC3	0.959	48.25	19	19	3.38E-12	0.914	0.981	v5.3	OASIS
LH_parsopercularis_surfavg	ICC2	0.936	35.41	19	19	5.64E-11	0.855	0.971	v5.3 vs. v6.0	OASIS
LH_parsopercularis_surfavg	ICC2	0.947	38.73	19	19	2.51E-11	0.890	0.975	v5.3 vs. v7.1	OASIS
LH_parsopercularis_surfavg	ICC3	0.987	152.40	19	19	7.73E-17	0.972	0.994	v6.0	OASIS
LH_parsopercularis_surfavg	ICC2	0.988	193.74	19	19	8.10E-18	0.973	0.995	v6.0 vs. v7.1	OASIS
LH_parsopercularis_surfavg	ICC3	0.978	91.42	19	19	9.21E-15	0.954	0.990	v7.1	OASIS
LH_parsopercularis_thickavg	ICC3	0.817	9.95	19	19	3.05E-06	0.642	0.911	v5.3	OASIS
LH_parsopercularis_thickavg	ICC2	0.862	25.11	19	19	1.22E-09	0.461	0.949	v5.3 vs. v6.0	OASIS
LH_parsopercularis_thickavg	ICC2	0.876	16.22	19	19	5.42E-08	0.748	0.941	v5.3 vs. v7.1	OASIS

LH_parsopercularis_thickavg	ICC3	0.840	11.53	19	19	9.34E-07	0.683	0.923	v6.0	OASIS
LH_parsopercularis_thickavg	ICC2	0.857	15.66	19	19	7.31E-08	0.686	0.934	v6.0 vs. v7.1	OASIS
LH_parsopercularis_thickavg	ICC3	0.859	13.15	19	19	3.16E-07	0.717	0.932	v7.1	OASIS
LH_parsorbitalis_surfavg	ICC3	0.853	12.58	19	19	4.57E-07	0.706	0.929	v5.3	OASIS
LH_parsorbitalis_surfavg	ICC2	0.420	9.37	19	19	4.90E-06	-0.059	0.741	v5.3 vs. v6.0	OASIS
LH_parsorbitalis_surfavg	ICC2	0.329	10.62	19	19	1.82E-06	-0.041	0.671	v5.3 vs. v7.1	OASIS
LH_parsorbitalis_surfavg	ICC3	0.915	22.51	19	19	3.19E-09	0.824	0.960	v6.0	OASIS
LH_parsorbitalis_surfavg	ICC2	0.889	30.56	19	19	2.12E-10	0.557	0.959	v6.0 vs. v7.1	OASIS
LH_parsorbitalis_surfavg	ICC3	0.953	41.35	19	19	1.38E-11	0.900	0.978	v7.1	OASIS
LH_parsorbitalis_thickavg	ICC3	0.908	20.80	19	19	6.37E-09	0.811	0.957	v5.3	OASIS
LH_parsorbitalis_thickavg	ICC2	0.909	26.99	19	19	6.43E-10	0.776	0.960	v5.3 vs. v6.0	OASIS
LH_parsorbitalis_thickavg	ICC2	0.884	16.03	19	19	5.98E-08	0.767	0.945	v5.3 vs. v7.1	OASIS
LH_parsorbitalis_thickavg	ICC3	0.917	23.11	19	19	2.53E-09	0.828	0.961	v6.0	OASIS
LH_parsorbitalis_thickavg	ICC2	0.891	18.49	19	19	1.76E-08	0.778	0.948	v6.0 vs. v7.1	OASIS
LH_parsorbitalis_thickavg	ICC3	0.880	15.68	19	19	7.25E-08	0.757	0.943	v7.1	OASIS
LH_parstriangularis_surfavg	ICC3	0.969	63.32	19	19	2.78E-13	0.934	0.986	v5.3	OASIS
LH_parstriangularis_surfavg	ICC2	0.930	32.54	19	19	1.21E-10	0.842	0.968	v5.3 vs. v6.0	OASIS
LH_parstriangularis_surfavg	ICC2	0.927	43.19	19	19	9.29E-12	0.725	0.972	v5.3 vs. v7.1	OASIS
LH_parstriangularis_surfavg	ICC3	0.981	104.24	19	19	2.71E-15	0.959	0.991	v6.0	OASIS
LH_parstriangularis_surfavg	ICC2	0.976	96.44	19	19	5.60E-15	0.945	0.989	v6.0 vs. v7.1	OASIS
LH_parstriangularis_surfavg	ICC3	0.983	114.93	19	19	1.09E-15	0.963	0.992	v7.1	OASIS
LH_parstriangularis_thickavg	ICC3	0.709	5.88	19	19	1.59E-04	0.461	0.855	v5.3	OASIS
LH_parstriangularis_thickavg	ICC2	0.785	12.41	19	19	5.12E-07	0.437	0.908	v5.3 vs. v6.0	OASIS
LH_parstriangularis_thickavg	ICC2	0.826	10.49	19	19	2.00E-06	0.661	0.915	v5.3 vs. v7.1	OASIS
LH_parstriangularis_thickavg	ICC3	0.790	8.53	19	19	1.01E-05	0.595	0.897	v6.0	OASIS
LH_parstriangularis_thickavg	ICC2	0.841	16.70	19	19	4.24E-08	0.569	0.933	v6.0 vs. v7.1	OASIS
LH_parstriangularis_thickavg	ICC3	0.833	10.98	19	19	1.38E-06	0.670	0.919	v7.1	OASIS
LH_pericalcarine_surfavg	ICC3	0.967	59.40	19	19	5.01E-13	0.930	0.985	v5.3	OASIS
LH_pericalcarine_surfavg	ICC2	0.935	41.49	19	19	1.34E-11	0.808	0.973	v5.3 vs. v6.0	OASIS
LH_pericalcarine_surfavg	ICC2	0.883	26.02	19	19	8.90E-10	0.601	0.954	v5.3 vs. v7.1	OASIS
LH_pericalcarine_surfavg	ICC3	0.988	169.29	19	19	2.88E-17	0.975	0.995	v6.0	OASIS
LH_pericalcarine_surfavg	ICC2	0.973	95.97	19	19	5.86E-15	0.929	0.989	v6.0 vs. v7.1	OASIS
LH_pericalcarine_surfavg	ICC3	0.976	81.53	19	19	2.67E-14	0.948	0.989	v7.1	OASIS
LH_pericalcarine_thickavg	ICC3	0.865	13.84	19	19	2.07E-07	0.729	0.936	v5.3	OASIS
LH_pericalcarine_thickavg	ICC2	0.686	9.42	19	19	4.68E-06	0.183	0.868	v5.3 vs. v6.0	OASIS
LH_pericalcarine_thickavg	ICC2	0.813	9.83	19	19	3.36E-06	0.640	0.909	v5.3 vs. v7.1	OASIS
LH_pericalcarine_thickavg	ICC3	0.807	9.36	19	19	4.92E-06	0.624	0.906	v6.0	OASIS
LH_pericalcarine_thickavg	ICC2	0.853	27.28	19	19	5.84E-10	0.340	0.949	v6.0 vs. v7.1	OASIS
LH_pericalcarine_thickavg	ICC3	0.891	17.35	19	19	3.05E-08	0.778	0.948	v7.1	OASIS
LH_postcentral_surfavg	ICC3	0.951	39.94	19	19	1.89E-11	0.897	0.977	v5.3	OASIS
LH_postcentral_surfavg	ICC2	0.632	4.58	19	19	8.61E-04	0.353	0.810	v5.3 vs. v6.0	OASIS
LH_postcentral_surfavg	ICC2	0.706	5.69	19	19	2.01E-04	0.458	0.852	v5.3 vs. v7.1	OASIS
LH_postcentral_surfavg	ICC3	0.986	137.25	19	19	2.07E-16	0.969	0.993	v6.0	OASIS
LH_postcentral_surfavg	ICC2	0.970	99.41	19	19	4.22E-15	0.895	0.988	v6.0 vs. v7.1	OASIS

LH_postcentral_surfavg	ICC3	0.974	76.07	19	19	5.09E-14	0.945	0.988	v7.1	OASIS
LH_postcentral_thickavg	ICC3	0.888	16.82	19	19	3.98E-08	0.772	0.947	v5.3	OASIS
LH_postcentral_thickavg	ICC2	0.815	26.01	19	19	8.91E-10	0.150	0.938	v5.3 vs. v6.0	OASIS
LH_postcentral_thickavg	ICC2	0.873	25.55	19	19	1.04E-09	0.535	0.952	v5.3 vs. v7.1	OASIS
LH_postcentral_thickavg	ICC3	0.944	34.69	19	19	6.78E-11	0.882	0.974	v6.0	OASIS
LH_postcentral_thickavg	ICC2	0.966	103.96	19	19	2.78E-15	0.828	0.988	v6.0 vs. v7.1	OASIS
LH_postcentral_thickavg	ICC3	0.690	5.46	19	19	2.67E-04	0.431	0.844	v7.1	OASIS
LH_posteriorcingulate_surfavg	ICC3	0.976	83.09	19	19	2.24E-14	0.949	0.989	v5.3	OASIS
LH_posteriorcingulate_surfavg	ICC2	0.951	38.52	19	19	2.63E-11	0.897	0.977	v5.3 vs. v6.0	OASIS
LH_posteriorcingulate_surfavg	ICC2	0.939	40.01	19	19	1.87E-11	0.847	0.973	v5.3 vs. v7.1	OASIS
LH_posteriorcingulate_surfavg	ICC3	0.973	74.47	19	19	6.20E-14	0.943	0.988	v6.0	OASIS
LH_posteriorcingulate_surfavg	ICC2	0.974	120.39	19	19	7.05E-16	0.899	0.990	v6.0 vs. v7.1	OASIS
LH_posteriorcingulate_surfavg	ICC3	0.989	179.21	19	19	1.69E-17	0.976	0.995	v7.1	OASIS
LH_posteriorcingulate_thickavg	ICC3	0.676	5.16	19	19	3.89E-04	0.409	0.836	v5.3	OASIS
LH_posteriorcingulate_thickavg	ICC2	0.837	17.37	19	19	3.02E-08	0.525	0.933	v5.3 vs. v6.0	OASIS
LH_posteriorcingulate_thickavg	ICC2	0.304	7.80	19	19	2.01E-05	-0.050	0.642	v5.3 vs. v7.1	OASIS
LH_posteriorcingulate_thickavg	ICC3	0.649	4.70	19	19	7.26E-04	0.369	0.821	v6.0	OASIS
LH_posteriorcingulate_thickavg	ICC2	0.446	11.33	19	19	1.07E-06	-0.054	0.763	v6.0 vs. v7.1	OASIS
LH_posteriorcingulate_thickavg	ICC3	0.725	6.26	19	19	1.01E-04	0.486	0.863	v7.1	OASIS
LH_precentral_surfavg	ICC3	0.578	3.74	19	19	3.04E-03	0.266	0.780	v5.3	OASIS
LH_precentral_surfavg	ICC2	0.899	23.01	19	19	2.63E-09	0.769	0.955	v5.3 vs. v6.0	OASIS
LH_precentral_surfavg	ICC2	0.906	30.33	19	19	2.27E-10	0.708	0.962	v5.3 vs. v7.1	OASIS
LH_precentral_surfavg	ICC3	0.982	110.12	19	19	1.62E-15	0.961	0.992	v6.0	OASIS
LH_precentral_surfavg	ICC2	0.991	246.23	19	19	8.46E-19	0.979	0.996	v6.0 vs. v7.1	OASIS
LH_precentral_surfavg	ICC3	0.900	19.07	19	19	1.35E-08	0.796	0.953	v7.1	OASIS
LH_precentral_thickavg	ICC3	0.769	7.65	19	19	2.33E-05	0.558	0.886	v5.3	OASIS
LH_precentral_thickavg	ICC2	0.743	21.75	19	19	4.31E-09	0.018	0.913	v5.3 vs. v6.0	OASIS
LH_precentral_thickavg	ICC2	0.828	31.43	19	19	1.65E-10	0.129	0.944	v5.3 vs. v7.1	OASIS
LH_precentral_thickavg	ICC3	0.912	21.69	19	19	4.42E-09	0.818	0.958	v6.0	OASIS
LH_precentral_thickavg	ICC2	0.946	48.47	19	19	3.24E-12	0.852	0.977	v6.0 vs. v7.1	OASIS
LH_precentral_thickavg	ICC3	0.594	3.92	19	19	2.27E-03	0.288	0.790	v7.1	OASIS
LH_precuneus_surfavg	ICC3	0.964	55.11	19	19	9.98E-13	0.924	0.983	v5.3	OASIS
LH_precuneus_surfavg	ICC2	0.970	85.32	19	19	1.75E-14	0.922	0.987	v5.3 vs. v6.0	OASIS
LH_precuneus_surfavg	ICC2	0.965	87.74	19	19	1.35E-14	0.873	0.987	v5.3 vs. v7.1	OASIS
LH_precuneus_surfavg	ICC3	0.988	165.51	19	19	3.56E-17	0.974	0.994	v6.0	OASIS
LH_precuneus_surfavg	ICC2	0.993	320.44	19	19	7.04E-20	0.986	0.997	v6.0 vs. v7.1	OASIS
LH_precuneus_surfavg	ICC3	0.850	12.31	19	19	5.46E-07	0.700	0.928	v7.1	OASIS
LH_precuneus_thickavg	ICC3	0.818	9.98	19	19	2.96E-06	0.643	0.912	v5.3	OASIS
LH_precuneus_thickavg	ICC2	0.766	20.39	19	19	7.56E-09	0.080	0.919	v5.3 vs. v6.0	OASIS
LH_precuneus_thickavg	ICC2	0.861	15.96	19	19	6.21E-08	0.697	0.936	v5.3 vs. v7.1	OASIS
LH_precuneus_thickavg	ICC3	0.901	19.15	19	19	1.31E-08	0.797	0.953	v6.0	OASIS
LH_precuneus_thickavg	ICC2	0.847	22.15	19	19	3.67E-09	0.433	0.943	v6.0 vs. v7.1	OASIS
LH_precuneus_thickavg	ICC3	0.933	28.83	19	19	3.57E-10	0.860	0.969	v7.1	OASIS
LH_Putamen	ICC3	0.836	11.19	19	19	1.19E-06	0.675	0.921	v5.3	OASIS

LH_Putamen	ICC2	0.378	8.43	19	19	1.12E-05	-0.058	0.708	v5.3 vs. v6.0	OASIS
LH_Putamen	ICC2	0.378	7.91	19	19	1.82E-05	-0.061	0.707	v5.3 vs. v7.1	OASIS
LH_Putamen	ICC3	0.964	54.51	19	19	1.10E-12	0.923	0.983	v6.0	OASIS
LH_Putamen	ICC2	0.989	172.59	19	19	2.40E-17	0.976	0.995	v6.0 vs. v7.1	OASIS
LH_Putamen	ICC3	0.978	90.44	19	19	1.02E-14	0.953	0.990	v7.1	OASIS
LH_rostralanteriorcingulate_surfavg	ICC3	0.934	29.37	19	19	3.03E-10	0.862	0.969	v5.3	OASIS
LH_rostralanteriorcingulate_surfavg	ICC2	0.928	27.65	19	19	5.18E-10	0.851	0.966	v5.3 vs. v6.0	OASIS
LH_rostralanteriorcingulate_surfavg	ICC2	0.824	11.75	19	19	7.98E-07	0.644	0.916	v5.3 vs. v7.1	OASIS
LH_rostralanteriorcingulate_surfavg	ICC3	0.931	28.00	19	19	4.63E-10	0.856	0.968	v6.0	OASIS
LH_rostralanteriorcingulate_surfavg	ICC2	0.867	23.50	19	19	2.19E-09	0.543	0.949	v6.0 vs. v7.1	OASIS
LH_rostralanteriorcingulate_surfavg	ICC3	0.856	12.91	19	19	3.68E-07	0.712	0.931	v7.1	OASIS
LH_rostralanteriorcingulate_thickavg	ICC3	0.577	3.73	19	19	3.06E-03	0.265	0.780	v5.3	OASIS
LH_rostralanteriorcingulate_thickavg	ICC2	0.769	9.89	19	19	3.20E-06	0.494	0.893	v5.3 vs. v6.0	OASIS
LH_rostralanteriorcingulate_thickavg	ICC2	0.789	10.31	19	19	2.30E-06	0.557	0.901	v5.3 vs. v7.1	OASIS
LH_rostralanteriorcingulate_thickavg	ICC3	0.789	8.49	19	19	1.06E-05	0.593	0.897	v6.0	OASIS
LH_rostralanteriorcingulate_thickavg	ICC2	0.640	11.91	19	19	7.16E-07	-0.007	0.862	v6.0 vs. v7.1	OASIS
LH_rostralanteriorcingulate_thickavg	ICC3	0.712	5.94	19	19	1.47E-04	0.465	0.856	v7.1	OASIS
LH_rostralmiddlefrontal_surfavg	ICC3	0.975	80.48	19	19	3.01E-14	0.948	0.989	v5.3	OASIS
LH_rostralmiddlefrontal_surfavg	ICC2	0.946	34.20	19	19	7.70E-11	0.886	0.975	v5.3 vs. v6.0	OASIS
LH_rostralmiddlefrontal_surfavg	ICC2	0.927	27.43	19	19	5.57E-10	0.850	0.966	v5.3 vs. v7.1	OASIS
LH_rostralmiddlefrontal_surfavg	ICC3	0.985	130.94	19	19	3.21E-16	0.967	0.993	v6.0	OASIS
LH_rostralmiddlefrontal_surfavg	ICC2	0.986	183.53	19	19	1.35E-17	0.961	0.994	v6.0 vs. v7.1	OASIS
LH_rostralmiddlefrontal_surfavg	ICC3	0.909	21.05	19	19	5.73E-09	0.813	0.957	v7.1	OASIS
LH_rostralmiddlefrontal_thickavg	ICC3	0.719	6.12	19	19	1.19E-04	0.477	0.860	v5.3	OASIS
LH_rostralmiddlefrontal_thickavg	ICC2	0.885	21.03	19	19	5.78E-09	0.720	0.949	v5.3 vs. v6.0	OASIS
LH_rostralmiddlefrontal_thickavg	ICC2	0.824	10.93	19	19	1.44E-06	0.656	0.914	v5.3 vs. v7.1	OASIS
LH_rostralmiddlefrontal_thickavg	ICC3	0.904	19.86	19	19	9.51E-09	0.803	0.955	v6.0	OASIS
LH_rostralmiddlefrontal_thickavg	ICC2	0.895	17.90	19	19	2.34E-08	0.788	0.950	v6.0 vs. v7.1	OASIS
LH_rostralmiddlefrontal_thickavg	ICC3	0.892	17.57	19	19	2.74E-08	0.780	0.949	v7.1	OASIS
LH_superiorfrontal_surfavg	ICC3	0.976	80.68	19	19	2.94E-14	0.948	0.989	v5.3	OASIS
LH_superiorfrontal_surfavg	ICC2	0.899	38.64	19	19	2.56E-11	0.499	0.965	v5.3 vs. v6.0	OASIS
LH_superiorfrontal_surfavg	ICC2	0.823	45.46	19	19	5.83E-12	0.032	0.947	v5.3 vs. v7.1	OASIS
LH_superiorfrontal_surfavg	ICC3	0.985	131.89	19	19	3.00E-16	0.968	0.993	v6.0	OASIS
LH_superiorfrontal_surfavg	ICC2	0.958	112.43	19	19	1.34E-15	0.659	0.987	v6.0 vs. v7.1	OASIS
LH_superiorfrontal_surfavg	ICC3	0.879	15.56	19	19	7.73E-08	0.755	0.942	v7.1	OASIS
LH_superiorfrontal_thickavg	ICC3	0.787	8.40	19	19	1.14E-05	0.590	0.896	v5.3	OASIS
LH_superiorfrontal_thickavg	ICC2	0.942	39.42	19	19	2.14E-11	0.865	0.974	v5.3 vs. v6.0	OASIS
LH_superiorfrontal_thickavg	ICC2	0.901	18.80	19	19	1.53E-08	0.799	0.953	v5.3 vs. v7.1	OASIS
LH_superiorfrontal_thickavg	ICC3	0.870	14.39	19	19	1.49E-07	0.738	0.938	v6.0	OASIS
LH_superiorfrontal_thickavg	ICC2	0.944	35.26	19	19	5.86E-11	0.883	0.973	v6.0 vs. v7.1	OASIS
LH_superiorfrontal_thickavg	ICC3	0.848	12.13	19	19	6.17E-07	0.697	0.927	v7.1	OASIS
LH_superiorparietal_surfavg	ICC3	0.812	9.66	19	19	3.84E-06	0.633	0.909	v5.3	OASIS
LH_superiorparietal_surfavg	ICC2	0.950	37.44	19	19	3.40E-11	0.895	0.977	v5.3 vs. v6.0	OASIS
LH_superiorparietal_surfavg	ICC2	0.954	43.56	19	19	8.61E-12	0.904	0.978	v5.3 vs. v7.1	OASIS

LH_superiorparietal_surfavg	ICC3	0.996	503.72	19	19	9.77E-22	0.991	0.998	v6.0	OASIS
LH_superiorparietal_surfavg	ICC2	0.990	275.05	19	19	2.98E-19	0.967	0.996	v6.0 vs. v7.1	OASIS
LH_superiorparietal_surfavg	ICC3	0.708	5.85	19	19	1.65E-04	0.459	0.854	v7.1	OASIS
LH_superiorparietal_thickavg	ICC3	0.865	13.76	19	19	2.16E-07	0.728	0.935	v5.3	OASIS
LH_superiorparietal_thickavg	ICC2	0.827	29.81	19	19	2.65E-10	0.144	0.943	v5.3 vs. v6.0	OASIS
LH_superiorparietal_thickavg	ICC2	0.833	23.82	19	19	1.94E-09	0.293	0.941	v5.3 vs. v7.1	OASIS
LH_superiorparietal_thickavg	ICC3	0.934	29.40	19	19	3.00E-10	0.863	0.969	v6.0	OASIS
LH_superiorparietal_thickavg	ICC2	0.936	29.42	19	19	2.98E-10	0.867	0.970	v6.0 vs. v7.1	OASIS
LH_superiorparietal_thickavg	ICC3	0.935	29.58	19	19	2.84E-10	0.863	0.969	v7.1	OASIS
LH_superiortemporal_surfavg	ICC3	0.987	148.25	19	19	1.00E-16	0.971	0.994	v5.3	OASIS
LH_superiortemporal_surfavg	ICC2	0.905	59.70	19	19	4.78E-13	0.295	0.971	v5.3 vs. v6.0	OASIS
LH_superiortemporal_surfavg	ICC2	0.853	42.51	19	19	1.07E-11	0.121	0.954	v5.3 vs. v7.1	OASIS
LH_superiortemporal_surfavg	ICC3	0.993	301.56	19	19	1.25E-19	0.986	0.997	v6.0	OASIS
LH_superiortemporal_surfavg	ICC2	0.989	391.20	19	19	1.07E-20	0.920	0.996	v6.0 vs. v7.1	OASIS
LH_superiortemporal_surfavg	ICC3	0.988	159.05	19	19	5.18E-17	0.973	0.994	v7.1	OASIS
LH_superiortemporal_thickavg	ICC3	0.708	5.86	19	19	1.63E-04	0.460	0.854	v5.3	OASIS
LH_superiortemporal_thickavg	ICC2	0.826	28.11	19	19	4.48E-10	0.167	0.942	v5.3 vs. v6.0	OASIS
LH_superiortemporal_thickavg	ICC2	0.883	15.51	19	19	7.95E-08	0.763	0.945	v5.3 vs. v7.1	OASIS
LH_superiortemporal_thickavg	ICC3	0.648	4.69	19	19	7.40E-04	0.367	0.821	v6.0	OASIS
LH_superiortemporal_thickavg	ICC2	0.773	16.60	19	19	4.45E-08	0.196	0.916	v6.0 vs. v7.1	OASIS
LH_superiortemporal_thickavg	ICC3	0.824	10.38	19	19	2.17E-06	0.655	0.915	v7.1	OASIS
LH_supramarginal_surfavg	ICC3	0.980	99.95	19	19	4.01E-15	0.958	0.991	v5.3	OASIS
LH_supramarginal_surfavg	ICC2	0.827	24.77	19	19	1.37E-09	0.232	0.940	v5.3 vs. v6.0	OASIS
LH_supramarginal_surfavg	ICC2	0.788	21.27	19	19	5.23E-09	0.133	0.926	v5.3 vs. v7.1	OASIS
LH_supramarginal_surfavg	ICC3	0.984	122.68	19	19	5.91E-16	0.965	0.993	v6.0	OASIS
LH_supramarginal_surfavg	ICC2	0.992	392.51	19	19	1.03E-20	0.972	0.997	v6.0 vs. v7.1	OASIS
LH_supramarginal_surfavg	ICC3	0.970	64.98	19	19	2.19E-13	0.935	0.986	v7.1	OASIS
LH_supramarginal_thickavg	ICC3	0.917	23.05	19	19	2.59E-09	0.828	0.961	v5.3	OASIS
LH_supramarginal_thickavg	ICC2	0.938	55.97	19	19	8.66E-13	0.719	0.978	v5.3 vs. v6.0	OASIS
LH_supramarginal_thickavg	ICC2	0.877	16.23	19	19	5.40E-08	0.753	0.941	v5.3 vs. v7.1	OASIS
LH_supramarginal_thickavg	ICC3	0.942	33.44	19	19	9.43E-11	0.878	0.973	v6.0	OASIS
LH_supramarginal_thickavg	ICC2	0.848	25.01	19	19	1.26E-09	0.358	0.946	v6.0 vs. v7.1	OASIS
LH_supramarginal_thickavg	ICC3	0.894	17.81	19	19	2.44E-08	0.783	0.950	v7.1	OASIS
LH_SurfArea	ICC3	0.995	368.64	19	19	1.87E-20	0.988	0.998	v5.3	OASIS
LH_SurfArea	ICC2	0.975	872.29	19	19	5.38E-24	0.241	0.994	v5.3 vs. v6.0	OASIS
LH_SurfArea	ICC2	0.918	538.89	19	19	5.16E-22	0.046	0.979	v5.3 vs. v7.1	OASIS
LH_SurfArea	ICC3	0.996	485.00	19	19	1.40E-21	0.991	0.998	v6.0	OASIS
LH_SurfArea	ICC2	0.979	691.59	19	19	4.85E-23	0.365	0.995	v6.0 vs. v7.1	OASIS
LH_SurfArea	ICC3	0.993	288.00	19	19	1.93E-19	0.985	0.997	v7.1	OASIS
LH_temporalpole_surfavg	ICC3	0.716	6.05	19	19	1.30E-04	0.472	0.858	v5.3	OASIS
LH_temporalpole_surfavg	ICC2	0.555	3.47	19	19	4.71E-03	0.238	0.766	v5.3 vs. v6.0	OASIS
LH_temporalpole_surfavg	ICC2	0.339	2.62	19	19	2.10E-02	-0.004	0.619	v5.3 vs. v7.1	OASIS
LH_temporalpole_surfavg	ICC3	0.765	7.53	19	19	2.64E-05	0.553	0.885	v6.0	OASIS
LH_temporalpole_surfavg	ICC2	0.540	9.66	19	19	3.85E-06	-0.052	0.812	v6.0 vs. v7.1	OASIS

LH_temporalpole_surfavg	ICC3	0.678	5.21	19	19	3.66E-04	0.412	0.837	v7.1	OASIS
LH_temporalpole_thickavg	ICC3	0.781	8.12	19	19	1.48E-05	0.579	0.893	v5.3	OASIS
LH_temporalpole_thickavg	ICC2	0.820	9.71	19	19	3.69E-06	0.646	0.913	v5.3 vs. v6.0	OASIS
LH_temporalpole_thickavg	ICC2	0.642	5.74	19	19	1.88E-04	0.317	0.823	v5.3 vs. v7.1	OASIS
LH_temporalpole_thickavg	ICC3	0.651	4.73	19	19	6.98E-04	0.371	0.822	v6.0	OASIS
LH_temporalpole_thickavg	ICC2	0.732	9.71	19	19	3.68E-06	0.346	0.883	v6.0 vs. v7.1	OASIS
LH_temporalpole_thickavg	ICC3	0.754	7.12	19	19	3.98E-05	0.533	0.878	v7.1	OASIS
LH_Thalamus	ICC3	0.923	24.90	19	19	1.31E-09	0.840	0.964	v5.3	OASIS
LH_Thalamus	ICC2	0.865	17.90	19	19	2.33E-08	0.676	0.940	v5.3 vs. v6.0	OASIS
LH_Thalamus	ICC2	0.749	15.99	19	19	6.11E-08	0.122	0.908	v5.3 vs. v7.1	OASIS
LH_Thalamus	ICC3	0.972	69.52	19	19	1.17E-13	0.940	0.987	v6.0	OASIS
LH_Thalamus	ICC2	0.844	17.22	19	19	3.26E-08	0.570	0.934	v6.0 vs. v7.1	OASIS
LH_Thalamus	ICC3	0.840	11.48	19	19	9.67E-07	0.682	0.923	v7.1	OASIS
LH_Thickness	ICC3	0.798	8.91	19	19	7.28E-06	0.608	0.902	v5.3	OASIS
LH_Thickness	ICC2	0.790	41.37	19	19	1.38E-11	0.002	0.936	v5.3 vs. v6.0	OASIS
LH_Thickness	ICC2	0.895	19.62	19	19	1.06E-08	0.784	0.950	v5.3 vs. v7.1	OASIS
LH_Thickness	ICC3	0.872	14.57	19	19	1.35E-07	0.741	0.939	v6.0	OASIS
LH_Thickness	ICC2	0.813	22.59	19	19	3.09E-09	0.209	0.935	v6.0 vs. v7.1	OASIS
LH_Thickness	ICC3	0.895	18.08	19	19	2.14E-08	0.786	0.950	v7.1	OASIS
LH_transversetemporal_surfavg	ICC3	0.949	38.21	19	19	2.83E-11	0.893	0.976	v5.3	OASIS
LH_transversetemporal_surfavg	ICC2	0.832	11.11	19	19	1.26E-06	0.672	0.918	v5.3 vs. v6.0	OASIS
LH_transversetemporal_surfavg	ICC2	0.764	7.39	19	19	3.03E-05	0.553	0.883	v5.3 vs. v7.1	OASIS
LH_transversetemporal_surfavg	ICC3	0.945	35.63	19	19	5.33E-11	0.885	0.974	v6.0	OASIS
LH_transversetemporal_surfavg	ICC2	0.953	39.79	19	19	1.96E-11	0.901	0.978	v6.0 vs. v7.1	OASIS
LH_transversetemporal_surfavg	ICC3	0.943	34.00	19	19	8.14E-11	0.880	0.973	v7.1	OASIS
LH_transversetemporal_thickavg	ICC3	0.941	33.08	19	19	1.04E-10	0.877	0.973	v5.3	OASIS
LH_transversetemporal_thickavg	ICC2	0.780	31.58	19	19	1.58E-10	0.015	0.930	v5.3 vs. v6.0	OASIS
LH_transversetemporal_thickavg	ICC2	0.775	16.45	19	19	4.81E-08	0.211	0.917	v5.3 vs. v7.1	OASIS
LH_transversetemporal_thickavg	ICC3	0.882	16.00	19	19	6.09E-08	0.761	0.944	v6.0	OASIS
LH_transversetemporal_thickavg	ICC2	0.954	50.76	19	19	2.12E-12	0.891	0.979	v6.0 vs. v7.1	OASIS
LH_transversetemporal_thickavg	ICC3	0.931	28.13	19	19	4.44E-10	0.857	0.968	v7.1	OASIS
RH_Accumbens	ICC3	0.841	11.59	19	19	8.92E-07	0.685	0.923	v5.3	OASIS
RH_Accumbens	ICC2	0.574	8.41	19	19	1.13E-05	-0.013	0.823	v5.3 vs. v6.0	OASIS
RH_Accumbens	ICC2	0.668	11.22	19	19	1.16E-06	0.052	0.870	v5.3 vs. v7.1	OASIS
RH_Accumbens	ICC3	0.871	14.45	19	19	1.44E-07	0.739	0.938	v6.0	OASIS
RH_Accumbens	ICC2	0.915	24.12	19	19	1.74E-09	0.825	0.960	v6.0 vs. v7.1	OASIS
RH_Accumbens	ICC3	0.857	12.99	19	19	3.50E-07	0.714	0.931	v7.1	OASIS
RH_Amygdala	ICC3	0.798	8.91	19	19	7.25E-06	0.609	0.902	v5.3	OASIS
RH_Amygdala	ICC2	0.863	13.11	19	19	3.24E-07	0.725	0.934	v5.3 vs. v6.0	OASIS
RH_Amygdala	ICC2	0.871	13.89	19	19	2.00E-07	0.740	0.938	v5.3 vs. v7.1	OASIS
RH_Amygdala	ICC3	0.913	21.96	19	19	3.96E-09	0.820	0.959	v6.0	OASIS
RH_Amygdala	ICC2	0.942	34.68	19	19	6.80E-11	0.880	0.973	v6.0 vs. v7.1	OASIS
RH_Amygdala	ICC3	0.870	14.41	19	19	1.47E-07	0.738	0.938	v7.1	OASIS
RH_bankssts_surfavg	ICC3	0.963	52.96	19	19	1.44E-12	0.921	0.983	v5.3	OASIS

RH_bankssts_surfavg	ICC2	0.833	14.34	19	19	1.53E-07	0.606	0.925	v5.3 vs. v6.0	OASIS
RH_bankssts_surfavg	ICC2	0.835	13.40	19	19	2.70E-07	0.644	0.923	v5.3 vs. v7.1	OASIS
RH_bankssts_surfavg	ICC3	0.966	57.26	19	19	7.03E-13	0.927	0.984	v6.0	OASIS
RH_bankssts_surfavg	ICC2	0.968	60.77	19	19	4.06E-13	0.933	0.985	v6.0 vs. v7.1	OASIS
RH_bankssts_surfavg	ICC3	0.979	92.84	19	19	7.98E-15	0.954	0.990	v7.1	OASIS
RH_bankssts_thickavg	ICC3	0.800	9.01	19	19	6.67E-06	0.612	0.903	v5.3	OASIS
RH_bankssts_thickavg	ICC2	0.817	9.85	19	19	3.31E-06	0.645	0.911	v5.3 vs. v6.0	OASIS
RH_bankssts_thickavg	ICC2	0.768	7.36	19	19	3.12E-05	0.556	0.886	v5.3 vs. v7.1	OASIS
RH_bankssts_thickavg	ICC3	0.883	16.02	19	19	6.01E-08	0.762	0.944	v6.0	OASIS
RH_bankssts_thickavg	ICC2	0.903	19.09	19	19	1.34E-08	0.802	0.954	v6.0 vs. v7.1	OASIS
RH_bankssts_thickavg	ICC3	0.941	32.70	19	19	1.16E-10	0.876	0.972	v7.1	OASIS
RH_caudalanteriorcingulate_surfavg	ICC3	0.967	58.82	19	19	5.48E-13	0.929	0.984	v5.3	OASIS
RH_caudalanteriorcingulate_surfavg	ICC2	0.854	41.81	19	19	1.25E-11	0.133	0.955	v5.3 vs. v6.0	OASIS
RH_caudalanteriorcingulate_surfavg	ICC2	0.940	41.74	19	19	1.27E-11	0.843	0.974	v5.3 vs. v7.1	OASIS
RH_caudalanteriorcingulate_surfavg	ICC3	0.961	50.33	19	19	2.30E-12	0.917	0.982	v6.0	OASIS
RH_caudalanteriorcingulate_surfavg	ICC2	0.922	58.45	19	19	5.81E-13	0.499	0.975	v6.0 vs. v7.1	OASIS
RH_caudalanteriorcingulate_surfavg	ICC3	0.963	52.38	19	19	1.59E-12	0.921	0.983	v7.1	OASIS
RH_caudalanteriorcingulate_thickavg	ICC3	0.760	7.33	19	19	3.23E-05	0.543	0.882	v5.3	OASIS
RH_caudalanteriorcingulate_thickavg	ICC2	0.878	17.54	19	19	2.79E-08	0.741	0.943	v5.3 vs. v6.0	OASIS
RH_caudalanteriorcingulate_thickavg	ICC2	0.520	14.43	19	19	1.46E-07	-0.051	0.811	v5.3 vs. v7.1	OASIS
RH_caudalanteriorcingulate_thickavg	ICC3	0.813	9.67	19	19	3.82E-06	0.634	0.909	v6.0	OASIS
RH_caudalanteriorcingulate_thickavg	ICC2	0.538	8.69	19	19	8.79E-06	-0.045	0.808	v6.0 vs. v7.1	OASIS
RH_caudalanteriorcingulate_thickavg	ICC3	0.843	11.73	19	19	8.10E-07	0.688	0.924	v7.1	OASIS
RH_caudalmiddlefrontal_surfavg	ICC3	0.963	53.59	19	19	1.29E-12	0.922	0.983	v5.3	OASIS
RH_caudalmiddlefrontal_surfavg	ICC2	0.883	16.25	19	19	5.33E-08	0.766	0.944	v5.3 vs. v6.0	OASIS
RH_caudalmiddlefrontal_surfavg	ICC2	0.865	14.19	19	19	1.68E-07	0.731	0.935	v5.3 vs. v7.1	OASIS
RH_caudalmiddlefrontal_surfavg	ICC3	0.984	120.93	19	19	6.76E-16	0.965	0.992	v6.0	OASIS
RH_caudalmiddlefrontal_surfavg	ICC2	0.970	62.39	19	19	3.18E-13	0.936	0.986	v6.0 vs. v7.1	OASIS
RH_caudalmiddlefrontal_surfavg	ICC3	0.992	243.83	19	19	9.28E-19	0.982	0.996	v7.1	OASIS
RH_caudalmiddlefrontal_thickavg	ICC3	0.864	13.70	19	19	2.25E-07	0.727	0.935	v5.3	OASIS
RH_caudalmiddlefrontal_thickavg	ICC2	0.846	14.65	19	19	1.29E-07	0.662	0.929	v5.3 vs. v6.0	OASIS
RH_caudalmiddlefrontal_thickavg	ICC2	0.828	17.69	19	19	2.58E-08	0.453	0.932	v5.3 vs. v7.1	OASIS
RH_caudalmiddlefrontal_thickavg	ICC3	0.775	7.91	19	19	1.82E-05	0.570	0.890	v6.0	OASIS
RH_caudalmiddlefrontal_thickavg	ICC2	0.909	23.84	19	19	1.93E-09	0.803	0.958	v6.0 vs. v7.1	OASIS
RH_caudalmiddlefrontal_thickavg	ICC3	0.842	11.66	19	19	8.48E-07	0.686	0.924	v7.1	OASIS
RH_Caudate	ICC3	0.953	41.20	19	19	1.43E-11	0.900	0.978	v5.3	OASIS
RH_Caudate	ICC2	0.865	43.24	19	19	9.19E-12	0.168	0.958	v5.3 vs. v6.0	OASIS
RH_Caudate	ICC2	0.951	50.17	19	19	2.37E-12	0.878	0.979	v5.3 vs. v7.1	OASIS
RH_Caudate	ICC3	0.972	70.33	19	19	1.05E-13	0.940	0.987	v6.0	OASIS
RH_Caudate	ICC2	0.933	81.96	19	19	2.55E-14	0.426	0.979	v6.0 vs. v7.1	OASIS
RH_Caudate	ICC3	0.985	130.87	19	19	3.23E-16	0.967	0.993	v7.1	OASIS
RH_cuneus_surfavg	ICC3	0.870	14.41	19	19	1.47E-07	0.738	0.938	v5.3	OASIS
RH_cuneus_surfavg	ICC2	0.743	13.93	19	19	1.96E-07	0.171	0.902	v5.3 vs. v6.0	OASIS
RH_cuneus_surfavg	ICC2	0.606	13.28	19	19	2.91E-07	-0.042	0.852	v5.3 vs. v7.1	OASIS

RH_cuneus_surfavg	ICC3	0.990	208.78	19	19	4.01E-18	0.979	0.996	v6.0	OASIS
RH_cuneus_surfavg	ICC2	0.924	79.55	19	19	3.36E-14	0.332	0.977	v6.0 vs. v7.1	OASIS
RH_cuneus_surfavg	ICC3	0.992	248.95	19	19	7.63E-19	0.983	0.996	v7.1	OASIS
RH_cuneus_thickavg	ICC3	0.937	30.61	19	19	2.09E-10	0.868	0.970	v5.3	OASIS
RH_cuneus_thickavg	ICC2	0.886	45.00	19	19	6.39E-12	0.289	0.964	v5.3 vs. v6.0	OASIS
RH_cuneus_thickavg	ICC2	0.890	16.82	19	19	3.98E-08	0.778	0.948	v5.3 vs. v7.1	OASIS
RH_cuneus_thickavg	ICC3	0.931	27.99	19	19	4.65E-10	0.856	0.968	v6.0	OASIS
RH_cuneus_thickavg	ICC2	0.866	25.59	19	19	1.03E-09	0.480	0.950	v6.0 vs. v7.1	OASIS
RH_cuneus_thickavg	ICC3	0.905	19.96	19	19	9.12E-09	0.804	0.955	v7.1	OASIS
RH_entorhinal_surfavg	ICC3	0.871	14.47	19	19	1.43E-07	0.739	0.938	v5.3	OASIS
RH_entorhinal_surfavg	ICC2	0.549	8.46	19	19	1.08E-05	-0.036	0.812	v5.3 vs. v6.0	OASIS
RH_entorhinal_surfavg	ICC2	0.594	8.37	19	19	1.18E-05	0.014	0.831	v5.3 vs. v7.1	OASIS
RH_entorhinal_surfavg	ICC3	0.911	21.50	19	19	4.76E-09	0.817	0.958	v6.0	OASIS
RH_entorhinal_surfavg	ICC2	0.835	11.84	19	19	7.51E-07	0.676	0.920	v6.0 vs. v7.1	OASIS
RH_entorhinal_surfavg	ICC3	0.914	22.23	19	19	3.56E-09	0.822	0.959	v7.1	OASIS
RH_entorhinal_thickavg	ICC3	0.872	14.57	19	19	1.34E-07	0.741	0.939	v5.3	OASIS
RH_entorhinal_thickavg	ICC2	0.820	11.90	19	19	7.22E-07	0.625	0.915	v5.3 vs. v6.0	OASIS
RH_entorhinal_thickavg	ICC2	0.781	9.12	19	19	6.06E-06	0.573	0.894	v5.3 vs. v7.1	OASIS
RH_entorhinal_thickavg	ICC3	0.858	13.04	19	19	3.39E-07	0.715	0.932	v6.0	OASIS
RH_entorhinal_thickavg	ICC2	0.745	16.70	19	19	4.23E-08	0.094	0.908	v6.0 vs. v7.1	OASIS
RH_entorhinal_thickavg	ICC3	0.890	17.15	19	19	3.37E-08	0.776	0.948	v7.1	OASIS
RH_frontalpole_surfavg	ICC3	0.757	7.23	19	19	3.57E-05	0.538	0.880	v5.3	OASIS
RH_frontalpole_surfavg	ICC2	0.457	3.41	19	19	5.19E-03	0.097	0.707	v5.3 vs. v6.0	OASIS
RH_frontalpole_surfavg	ICC2	0.238	2.71	19	19	1.78E-02	-0.073	0.536	v5.3 vs. v7.1	OASIS
RH_frontalpole_surfavg	ICC3	0.918	23.30	19	19	2.35E-09	0.830	0.961	v6.0	OASIS
RH_frontalpole_surfavg	ICC2	0.618	12.05	19	19	6.48E-07	-0.028	0.854	v6.0 vs. v7.1	OASIS
RH_frontalpole_surfavg	ICC3	0.858	13.08	19	19	3.31E-07	0.716	0.932	v7.1	OASIS
RH_frontalpole_thickavg	ICC3	0.792	8.60	19	19	9.55E-06	0.597	0.898	v5.3	OASIS
RH_frontalpole_thickavg	ICC2	0.766	8.68	19	19	8.88E-06	0.539	0.886	v5.3 vs. v6.0	OASIS
RH_frontalpole_thickavg	ICC2	0.774	8.45	19	19	1.09E-05	0.570	0.889	v5.3 vs. v7.1	OASIS
RH_frontalpole_thickavg	ICC3	0.774	7.87	19	19	1.88E-05	0.568	0.889	v6.0	OASIS
RH_frontalpole_thickavg	ICC2	0.855	12.53	19	19	4.71E-07	0.712	0.930	v6.0 vs. v7.1	OASIS
RH_frontalpole_thickavg	ICC3	0.851	12.44	19	19	5.00E-07	0.703	0.929	v7.1	OASIS
RH_fusiform_surfavg	ICC3	0.979	92.93	19	19	7.91E-15	0.954	0.990	v5.3	OASIS
RH_fusiform_surfavg	ICC2	0.868	45.52	19	19	5.76E-12	0.167	0.959	v5.3 vs. v6.0	OASIS
RH_fusiform_surfavg	ICC2	0.889	40.58	19	19	1.64E-11	0.374	0.963	v5.3 vs. v7.1	OASIS
RH_fusiform_surfavg	ICC3	0.973	72.60	19	19	7.84E-14	0.942	0.987	v6.0	OASIS
RH_fusiform_surfavg	ICC2	0.981	116.30	19	19	9.74E-16	0.958	0.991	v6.0 vs. v7.1	OASIS
RH_fusiform_surfavg	ICC3	0.985	133.05	19	19	2.76E-16	0.968	0.993	v7.1	OASIS
RH_fusiform_thickavg	ICC3	0.596	3.95	19	19	2.19E-03	0.291	0.791	v5.3	OASIS
RH_fusiform_thickavg	ICC2	0.597	7.61	19	19	2.42E-05	0.050	0.827	v5.3 vs. v6.0	OASIS
RH_fusiform_thickavg	ICC2	0.581	4.73	19	19	6.97E-04	0.237	0.787	v5.3 vs. v7.1	OASIS
RH_fusiform_thickavg	ICC3	0.683	5.32	19	19	3.20E-04	0.421	0.840	v6.0	OASIS
RH_fusiform_thickavg	ICC2	0.843	13.18	19	19	3.09E-07	0.681	0.925	v6.0 vs. v7.1	OASIS

RH_fusiform_thickavg	ICC3	0.766	7.56	19	19	2.56E-05	0.554	0.885	v7.1	OASIS
RH_Hippocampus	ICC3	0.945	35.10	19	19	6.10E-11	0.884	0.974	v5.3	OASIS
RH_Hippocampus	ICC2	0.906	19.59	19	19	1.07E-08	0.807	0.955	v5.3 vs. v6.0	OASIS
RH_Hippocampus	ICC2	0.944	33.73	19	19	8.74E-11	0.883	0.973	v5.3 vs. v7.1	OASIS
RH_Hippocampus	ICC3	0.945	35.14	19	19	6.04E-11	0.884	0.974	v6.0	OASIS
RH_Hippocampus	ICC2	0.939	30.00	19	19	2.50E-10	0.871	0.971	v6.0 vs. v7.1	OASIS
RH_Hippocampus	ICC3	0.972	70.46	19	19	1.03E-13	0.940	0.987	v7.1	OASIS
RH_inferiorparietal_surfavg	ICC3	0.995	434.70	19	19	3.94E-21	0.990	0.998	v5.3	OASIS
RH_inferiorparietal_surfavg	ICC2	0.950	47.17	19	19	4.16E-12	0.882	0.978	v5.3 vs. v6.0	OASIS
RH_inferiorparietal_surfavg	ICC2	0.957	44.19	19	19	7.55E-12	0.909	0.980	v5.3 vs. v7.1	OASIS
RH_inferiorparietal_surfavg	ICC3	0.994	342.55	19	19	3.75E-20	0.987	0.997	v6.0	OASIS
RH_inferiorparietal_surfavg	ICC2	0.984	166.65	19	19	3.34E-17	0.953	0.993	v6.0 vs. v7.1	OASIS
RH_inferiorparietal_surfavg	ICC3	0.989	176.40	19	19	1.96E-17	0.976	0.995	v7.1	OASIS
RH_inferiorparietal_thickavg	ICC3	0.826	10.50	19	19	1.98E-06	0.658	0.916	v5.3	OASIS
RH_inferiorparietal_thickavg	ICC2	0.802	9.21	19	19	5.60E-06	0.620	0.903	v5.3 vs. v6.0	OASIS
RH_inferiorparietal_thickavg	ICC2	0.791	8.24	19	19	1.33E-05	0.595	0.898	v5.3 vs. v7.1	OASIS
RH_inferiorparietal_thickavg	ICC3	0.885	16.33	19	19	5.13E-08	0.766	0.945	v6.0	OASIS
RH_inferiorparietal_thickavg	ICC2	0.885	20.19	19	19	8.26E-09	0.737	0.948	v6.0 vs. v7.1	OASIS
RH_inferiorparietal_thickavg	ICC3	0.789	8.47	19	19	1.08E-05	0.592	0.897	v7.1	OASIS
RH_inferiortemporal_surfavg	ICC3	0.978	90.94	19	19	9.67E-15	0.953	0.990	v5.3	OASIS
RH_inferiortemporal_surfavg	ICC2	0.881	29.75	19	19	2.70E-10	0.505	0.957	v5.3 vs. v6.0	OASIS
RH_inferiortemporal_surfavg	ICC2	0.838	28.68	19	19	3.73E-10	0.212	0.946	v5.3 vs. v7.1	OASIS
RH_inferiortemporal_surfavg	ICC3	0.981	105.62	19	19	2.40E-15	0.960	0.991	v6.0	OASIS
RH_inferiortemporal_surfavg	ICC2	0.973	105.57	19	19	2.41E-15	0.908	0.989	v6.0 vs. v7.1	OASIS
RH_inferiortemporal_surfavg	ICC3	0.986	140.70	19	19	1.64E-16	0.970	0.993	v7.1	OASIS
RH_inferiortemporal_thickavg	ICC3	0.640	4.55	19	19	8.92E-04	0.355	0.816	v5.3	OASIS
RH_inferiortemporal_thickavg	ICC2	0.719	7.24	19	19	3.52E-05	0.454	0.863	v5.3 vs. v6.0	OASIS
RH_inferiortemporal_thickavg	ICC2	0.700	6.01	19	19	1.36E-04	0.453	0.849	v5.3 vs. v7.1	OASIS
RH_inferiortemporal_thickavg	ICC3	0.814	9.76	19	19	3.55E-06	0.636	0.910	v6.0	OASIS
RH_inferiortemporal_thickavg	ICC2	0.819	9.89	19	19	3.20E-06	0.648	0.912	v6.0 vs. v7.1	OASIS
RH_inferiortemporal_thickavg	ICC3	0.922	24.58	19	19	1.47E-09	0.838	0.963	v7.1	OASIS
RH_insula_surfavg	ICC3	0.912	21.84	19	19	4.16E-09	0.819	0.959	v5.3	OASIS
RH_insula_surfavg	ICC2	0.497	6.91	19	19	4.98E-05	-0.046	0.777	v5.3 vs. v6.0	OASIS
RH_insula_surfavg	ICC2	0.606	9.60	19	19	4.03E-06	-0.003	0.842	v5.3 vs. v7.1	OASIS
RH_insula_surfavg	ICC3	0.577	3.72	19	19	3.11E-03	0.264	0.780	v6.0	OASIS
RH_insula_surfavg	ICC2	0.697	5.44	19	19	2.73E-04	0.441	0.848	v6.0 vs. v7.1	OASIS
RH_insula_surfavg	ICC3	0.789	8.47	19	19	1.07E-05	0.592	0.897	v7.1	OASIS
RH_insula_thickavg	ICC3	0.723	6.22	19	19	1.07E-04	0.483	0.862	v5.3	OASIS
RH_insula_thickavg	ICC2	0.667	8.94	19	19	7.05E-06	0.149	0.860	v5.3 vs. v6.0	OASIS
RH_insula_thickavg	ICC2	0.812	12.32	19	19	5.43E-07	0.578	0.914	v5.3 vs. v7.1	OASIS
RH_insula_thickavg	ICC3	0.867	14.02	19	19	1.85E-07	0.732	0.936	v6.0	OASIS
RH_insula_thickavg	ICC2	0.588	28.16	19	19	4.41E-10	-0.032	0.855	v6.0 vs. v7.1	OASIS
RH_insula_thickavg	ICC3	0.840	11.48	19	19	9.66E-07	0.682	0.923	v7.1	OASIS
RH_isthmuscingulate_surfavg	ICC3	0.916	22.92	19	19	2.72E-09	0.827	0.961	v5.3	OASIS

RH_isthmuscingulate_surfavg	ICC2	0.929	26.66	19	19	7.17E-10	0.854	0.967	v5.3 vs. v6.0	OASIS
RH_isthmuscingulate_surfavg	ICC2	0.918	34.95	19	19	6.34E-11	0.743	0.967	v5.3 vs. v7.1	OASIS
RH_isthmuscingulate_surfavg	ICC3	0.964	55.32	19	19	9.65E-13	0.925	0.983	v6.0	OASIS
RH_isthmuscingulate_surfavg	ICC2	0.927	66.30	19	19	1.82E-13	0.475	0.977	v6.0 vs. v7.1	OASIS
RH_isthmuscingulate_surfavg	ICC3	0.978	90.76	19	19	9.85E-15	0.953	0.990	v7.1	OASIS
RH_isthmuscingulate_thickavg	ICC3	0.895	18.10	19	19	2.13E-08	0.786	0.950	v5.3	OASIS
RH_isthmuscingulate_thickavg	ICC2	0.898	19.03	19	19	1.38E-08	0.794	0.951	v5.3 vs. v6.0	OASIS
RH_isthmuscingulate_thickavg	ICC2	0.604	12.97	19	19	3.55E-07	-0.042	0.850	v5.3 vs. v7.1	OASIS
RH_isthmuscingulate_thickavg	ICC3	0.899	18.76	19	19	1.56E-08	0.793	0.952	v6.0	OASIS
RH_isthmuscingulate_thickavg	ICC2	0.669	17.22	19	19	3.26E-08	-0.025	0.883	v6.0 vs. v7.1	OASIS
RH_isthmuscingulate_thickavg	ICC3	0.921	24.20	19	19	1.69E-09	0.836	0.963	v7.1	OASIS
RH_lateraloccipital_surfavg	ICC3	0.953	41.45	19	19	1.35E-11	0.901	0.978	v5.3	OASIS
RH_lateraloccipital_surfavg	ICC2	0.678	16.24	19	19	5.36E-08	-0.014	0.885	v5.3 vs. v6.0	OASIS
RH_lateraloccipital_surfavg	ICC2	0.600	17.78	19	19	2.48E-08	-0.045	0.855	v5.3 vs. v7.1	OASIS
RH_lateraloccipital_surfavg	ICC3	0.990	197.14	19	19	6.87E-18	0.978	0.995	v6.0	OASIS
RH_lateraloccipital_surfavg	ICC2	0.977	325.41	19	19	6.09E-20	0.605	0.994	v6.0 vs. v7.1	OASIS
RH_lateraloccipital_surfavg	ICC3	0.992	253.88	19	19	6.34E-19	0.983	0.996	v7.1	OASIS
RH_lateraloccipital_thickavg	ICC3	0.880	15.64	19	19	7.38E-08	0.757	0.943	v5.3	OASIS
RH_lateraloccipital_thickavg	ICC2	0.888	17.31	19	19	3.11E-08	0.775	0.946	v5.3 vs. v6.0	OASIS
RH_lateraloccipital_thickavg	ICC2	0.866	15.03	19	19	1.04E-07	0.730	0.936	v5.3 vs. v7.1	OASIS
RH_lateraloccipital_thickavg	ICC3	0.916	22.94	19	19	2.70E-09	0.827	0.961	v6.0	OASIS
RH_lateraloccipital_thickavg	ICC2	0.884	26.30	19	19	8.09E-10	0.603	0.955	v6.0 vs. v7.1	OASIS
RH_lateraloccipital_thickavg	ICC3	0.981	104.38	19	19	2.68E-15	0.959	0.991	v7.1	OASIS
RH_lateralorbitofrontal_surfavg	ICC3	0.849	12.21	19	19	5.84E-07	0.698	0.927	v5.3	OASIS
RH_lateralorbitofrontal_surfavg	ICC2	0.813	12.56	19	19	4.61E-07	0.574	0.915	v5.3 vs. v6.0	OASIS
RH_lateralorbitofrontal_surfavg	ICC2	0.675	11.62	19	19	8.72E-07	0.055	0.874	v5.3 vs. v7.1	OASIS
RH_lateralorbitofrontal_surfavg	ICC3	0.908	20.85	19	19	6.22E-09	0.812	0.957	v6.0	OASIS
RH_lateralorbitofrontal_surfavg	ICC2	0.798	14.76	19	19	1.20E-07	0.394	0.919	v6.0 vs. v7.1	OASIS
RH_lateralorbitofrontal_surfavg	ICC3	0.803	9.17	19	19	5.79E-06	0.618	0.904	v7.1	OASIS
RH_lateralorbitofrontal_thickavg	ICC3	0.673	5.12	19	19	4.10E-04	0.405	0.835	v5.3	OASIS
RH_lateralorbitofrontal_thickavg	ICC2	0.708	6.46	19	19	8.11E-05	0.458	0.854	v5.3 vs. v6.0	OASIS
RH_lateralorbitofrontal_thickavg	ICC2	0.615	4.17	19	19	1.55E-03	0.324	0.801	v5.3 vs. v7.1	OASIS
RH_lateralorbitofrontal_thickavg	ICC3	0.765	7.52	19	19	2.66E-05	0.552	0.884	v6.0	OASIS
RH_lateralorbitofrontal_thickavg	ICC2	0.687	9.15	19	19	5.92E-06	0.203	0.867	v6.0 vs. v7.1	OASIS
RH_lateralorbitofrontal_thickavg	ICC3	0.585	3.82	19	19	2.69E-03	0.275	0.784	v7.1	OASIS
RH_LateralVentricle	ICC3	0.998	917.23	19	19	3.34E-24	0.995	0.999	v5.3	OASIS
RH_LateralVentricle	ICC2	0.994	1678.93	19	19	1.08E-26	0.781	0.998	v5.3 vs. v6.0	OASIS
RH_LateralVentricle	ICC2	0.993	1697.28	19	19	9.73E-27	0.740	0.998	v5.3 vs. v7.1	OASIS
RH_LateralVentricle	ICC3	0.999	1667.59	19	19	1.15E-26	0.997	0.999	v6.0	OASIS
RH_LateralVentricle	ICC2	1.000	4859.57	19	19	4.48E-31	0.999	1.000	v6.0 vs. v7.1	OASIS
RH_LateralVentricle	ICC3	0.999	1735.58	19	19	7.88E-27	0.998	0.999	v7.1	OASIS
RH_lingual_surfavg	ICC3	0.983	114.34	19	19	1.14E-15	0.963	0.992	v5.3	OASIS
RH_lingual_surfavg	ICC2	0.945	52.23	19	19	1.64E-12	0.823	0.978	v5.3 vs. v6.0	OASIS
RH_lingual_surfavg	ICC2	0.887	41.40	19	19	1.37E-11	0.341	0.963	v5.3 vs. v7.1	OASIS

RH_lingual_surfavg	ICC3	0.993	278.12	19	19	2.68E-19	0.985	0.997	v6.0	OASIS
RH_lingual_surfavg	ICC2	0.978	295.14	19	19	1.53E-19	0.667	0.994	v6.0 vs. v7.1	OASIS
RH_lingual_surfavg	ICC3	0.989	182.95	19	19	1.39E-17	0.977	0.995	v7.1	OASIS
RH_lingual_thickavg	ICC3	0.899	18.72	19	19	1.58E-08	0.792	0.952	v5.3	OASIS
RH_lingual_thickavg	ICC2	0.786	16.85	19	19	3.91E-08	0.245	0.920	v5.3 vs. v6.0	OASIS
RH_lingual_thickavg	ICC2	0.788	9.13	19	19	6.01E-06	0.591	0.896	v5.3 vs. v7.1	OASIS
RH_lingual_thickavg	ICC3	0.868	14.20	19	19	1.67E-07	0.735	0.937	v6.0	OASIS
RH_lingual_thickavg	ICC2	0.708	21.80	19	19	4.23E-09	-0.017	0.901	v6.0 vs. v7.1	OASIS
RH_lingual_thickavg	ICC3	0.888	16.90	19	19	3.83E-08	0.773	0.947	v7.1	OASIS
RH_medialorbitofrontal_surfavg	ICC3	0.807	9.35	19	19	4.98E-06	0.624	0.906	v5.3	OASIS
RH_medialorbitofrontal_surfavg	ICC2	0.613	8.62	19	19	9.38E-06	0.037	0.839	v5.3 vs. v6.0	OASIS
RH_medialorbitofrontal_surfavg	ICC2	0.291	8.33	19	19	1.22E-05	-0.045	0.630	v5.3 vs. v7.1	OASIS
RH_medialorbitofrontal_surfavg	ICC3	0.890	17.13	19	19	3.40E-08	0.775	0.948	v6.0	OASIS
RH_medialorbitofrontal_surfavg	ICC2	0.550	18.29	19	19	1.94E-08	-0.044	0.831	v6.0 vs. v7.1	OASIS
RH_medialorbitofrontal_surfavg	ICC3	0.905	19.98	19	19	9.02E-09	0.804	0.955	v7.1	OASIS
RH_medialorbitofrontal_thickavg	ICC3	0.733	6.50	19	19	7.80E-05	0.500	0.867	v5.3	OASIS
RH_medialorbitofrontal_thickavg	ICC2	0.771	9.08	19	19	6.24E-06	0.540	0.890	v5.3 vs. v6.0	OASIS
RH_medialorbitofrontal_thickavg	ICC2	0.743	6.55	19	19	7.39E-05	0.514	0.873	v5.3 vs. v7.1	OASIS
RH_medialorbitofrontal_thickavg	ICC3	0.801	9.03	19	19	6.54E-06	0.613	0.903	v6.0	OASIS
RH_medialorbitofrontal_thickavg	ICC2	0.872	22.07	19	19	3.79E-09	0.618	0.948	v6.0 vs. v7.1	OASIS
RH_medialorbitofrontal_thickavg	ICC3	0.775	7.89	19	19	1.84E-05	0.569	0.890	v7.1	OASIS
RH_middletemporal_surfavg	ICC3	0.983	118.59	19	19	8.11E-16	0.964	0.992	v5.3	OASIS
RH_middletemporal_surfavg	ICC2	0.931	90.07	19	19	1.06E-14	0.358	0.980	v5.3 vs. v6.0	OASIS
RH_middletemporal_surfavg	ICC2	0.855	42.75	19	19	1.02E-11	0.129	0.955	v5.3 vs. v7.1	OASIS
RH_middletemporal_surfavg	ICC3	0.990	198.19	19	19	6.54E-18	0.978	0.995	v6.0	OASIS
RH_middletemporal_surfavg	ICC2	0.971	113.77	19	19	1.20E-15	0.878	0.989	v6.0 vs. v7.1	OASIS
RH_middletemporal_surfavg	ICC3	0.986	137.24	19	19	2.07E-16	0.969	0.993	v7.1	OASIS
RH_middletemporal_thickavg	ICC3	0.771	7.75	19	19	2.12E-05	0.563	0.888	v5.3	OASIS
RH_middletemporal_thickavg	ICC2	0.784	8.10	19	19	1.51E-05	0.586	0.894	v5.3 vs. v6.0	OASIS
RH_middletemporal_thickavg	ICC2	0.618	4.15	19	19	1.62E-03	0.323	0.804	v5.3 vs. v7.1	OASIS
RH_middletemporal_thickavg	ICC3	0.868	14.10	19	19	1.76E-07	0.734	0.937	v6.0	OASIS
RH_middletemporal_thickavg	ICC2	0.764	8.11	19	19	1.50E-05	0.552	0.884	v6.0 vs. v7.1	OASIS
RH_middletemporal_thickavg	ICC3	0.850	12.31	19	19	5.47E-07	0.700	0.928	v7.1	OASIS
RH_Pallidum	ICC3	0.840	11.46	19	19	9.76E-07	0.682	0.923	v5.3	OASIS
RH_Pallidum	ICC2	0.874	15.21	19	19	9.37E-08	0.748	0.939	v5.3 vs. v6.0	OASIS
RH_Pallidum	ICC2	0.791	10.88	19	19	1.49E-06	0.540	0.903	v5.3 vs. v7.1	OASIS
RH_Pallidum	ICC3	0.948	37.82	19	19	3.11E-11	0.892	0.976	v6.0	OASIS
RH_Pallidum	ICC2	0.939	52.10	19	19	1.67E-12	0.766	0.977	v6.0 vs. v7.1	OASIS
RH_Pallidum	ICC3	0.938	31.39	19	19	1.67E-10	0.871	0.971	v7.1	OASIS
RH_paracentral_surfavg	ICC3	0.983	114.73	19	19	1.11E-15	0.963	0.992	v5.3	OASIS
RH_paracentral_surfavg	ICC2	0.942	31.81	19	19	1.48E-10	0.878	0.973	v5.3 vs. v6.0	OASIS
RH_paracentral_surfavg	ICC2	0.947	36.23	19	19	4.58E-11	0.889	0.975	v5.3 vs. v7.1	OASIS
RH_paracentral_surfavg	ICC3	0.984	127.25	19	19	4.19E-16	0.966	0.993	v6.0	OASIS
RH_paracentral_surfavg	ICC2	0.991	291.24	19	19	1.74E-19	0.977	0.996	v6.0 vs. v7.1	OASIS

RH_paracentral_surfavg	ICC3	0.993	280.71	19	19	2.46E-19	0.985	0.997	v7.1	OASIS
RH_paracentral_thickavg	ICC3	0.884	16.24	19	19	5.37E-08	0.764	0.945	v5.3	OASIS
RH_paracentral_thickavg	ICC2	0.701	21.16	19	19	5.47E-09	-0.020	0.898	v5.3 vs. v6.0	OASIS
RH_paracentral_thickavg	ICC2	0.723	16.61	19	19	4.43E-08	0.043	0.901	v5.3 vs. v7.1	OASIS
RH_paracentral_thickavg	ICC3	0.859	13.21	19	19	3.05E-07	0.718	0.933	v6.0	OASIS
RH_paracentral_thickavg	ICC2	0.946	37.69	19	19	3.20E-11	0.887	0.975	v6.0 vs. v7.1	OASIS
RH_paracentral_thickavg	ICC3	0.897	18.38	19	19	1.86E-08	0.789	0.951	v7.1	OASIS
RH_parahippocampal_surfavg	ICC3	0.911	21.59	19	19	4.60E-09	0.817	0.958	v5.3	OASIS
RH_parahippocampal_surfavg	ICC2	0.703	12.25	19	19	5.67E-07	0.104	0.885	v5.3 vs. v6.0	OASIS
RH_parahippocampal_surfavg	ICC2	0.538	6.77	19	19	5.75E-05	-0.006	0.796	v5.3 vs. v7.1	OASIS
RH_parahippocampal_surfavg	ICC3	0.914	22.23	19	19	3.56E-09	0.822	0.959	v6.0	OASIS
RH_parahippocampal_surfavg	ICC2	0.918	33.94	19	19	8.26E-11	0.751	0.967	v6.0 vs. v7.1	OASIS
RH_parahippocampal_surfavg	ICC3	0.949	38.41	19	19	2.70E-11	0.893	0.976	v7.1	OASIS
RH_parahippocampal_thickavg	ICC3	0.765	7.51	19	19	2.68E-05	0.552	0.884	v5.3	OASIS
RH_parahippocampal_thickavg	ICC2	0.818	19.16	19	19	1.30E-08	0.336	0.932	v5.3 vs. v6.0	OASIS
RH_parahippocampal_thickavg	ICC2	0.798	9.17	19	19	5.80E-06	0.613	0.901	v5.3 vs. v7.1	OASIS
RH_parahippocampal_thickavg	ICC3	0.762	7.39	19	19	3.02E-05	0.546	0.883	v6.0	OASIS
RH_parahippocampal_thickavg	ICC2	0.870	24.63	19	19	1.44E-09	0.535	0.950	v6.0 vs. v7.1	OASIS
RH_parahippocampal_thickavg	ICC3	0.850	12.32	19	19	5.42E-07	0.701	0.928	v7.1	OASIS
RH_parsopercularis_surfavg	ICC3	0.942	33.43	19	19	9.46E-11	0.878	0.973	v5.3	OASIS
RH_parsopercularis_surfavg	ICC2	0.906	24.26	19	19	1.65E-09	0.785	0.957	v5.3 vs. v6.0	OASIS
RH_parsopercularis_surfavg	ICC2	0.914	23.64	19	19	2.07E-09	0.822	0.959	v5.3 vs. v7.1	OASIS
RH_parsopercularis_surfavg	ICC3	0.968	61.00	19	19	3.92E-13	0.931	0.985	v6.0	OASIS
RH_parsopercularis_surfavg	ICC2	0.980	106.21	19	19	2.27E-15	0.956	0.991	v6.0 vs. v7.1	OASIS
RH_parsopercularis_surfavg	ICC3	0.988	164.10	19	19	3.86E-17	0.974	0.994	v7.1	OASIS
RH_parsopercularis_thickavg	ICC3	0.734	6.52	19	19	7.64E-05	0.501	0.868	v5.3	OASIS
RH_parsopercularis_thickavg	ICC2	0.872	17.76	19	19	2.50E-08	0.715	0.942	v5.3 vs. v6.0	OASIS
RH_parsopercularis_thickavg	ICC2	0.827	10.61	19	19	1.82E-06	0.663	0.916	v5.3 vs. v7.1	OASIS
RH_parsopercularis_thickavg	ICC3	0.865	13.86	19	19	2.05E-07	0.729	0.936	v6.0	OASIS
RH_parsopercularis_thickavg	ICC2	0.885	16.57	19	19	4.52E-08	0.769	0.945	v6.0 vs. v7.1	OASIS
RH_parsopercularis_thickavg	ICC3	0.859	13.18	19	19	3.11E-07	0.717	0.932	v7.1	OASIS
RH_parsorbitalis_surfavg	ICC3	0.924	25.26	19	19	1.16E-09	0.842	0.964	v5.3	OASIS
RH_parsorbitalis_surfavg	ICC2	0.568	10.56	19	19	1.89E-06	-0.046	0.828	v5.3 vs. v6.0	OASIS
RH_parsorbitalis_surfavg	ICC2	0.488	11.89	19	19	7.24E-07	-0.055	0.790	v5.3 vs. v7.1	OASIS
RH_parsorbitalis_surfavg	ICC3	0.946	36.28	19	19	4.53E-11	0.887	0.975	v6.0	OASIS
RH_parsorbitalis_surfavg	ICC2	0.948	106.50	19	19	2.22E-15	0.511	0.984	v6.0 vs. v7.1	OASIS
RH_parsorbitalis_surfavg	ICC3	0.973	73.90	19	19	6.65E-14	0.943	0.988	v7.1	OASIS
RH_parsorbitalis_thickavg	ICC3	0.721	6.18	19	19	1.12E-04	0.481	0.861	v5.3	OASIS
RH_parsorbitalis_thickavg	ICC2	0.842	11.31	19	19	1.09E-06	0.688	0.924	v5.3 vs. v6.0	OASIS
RH_parsorbitalis_thickavg	ICC2	0.847	12.08	19	19	6.34E-07	0.700	0.926	v5.3 vs. v7.1	OASIS
RH_parsorbitalis_thickavg	ICC3	0.816	9.88	19	19	3.22E-06	0.640	0.911	v6.0	OASIS
RH_parsorbitalis_thickavg	ICC2	0.923	24.40	19	19	1.57E-09	0.842	0.964	v6.0 vs. v7.1	OASIS
RH_parsorbitalis_thickavg	ICC3	0.800	9.02	19	19	6.61E-06	0.612	0.903	v7.1	OASIS
RH_parstriangularis_surfavg	ICC3	0.967	59.95	19	19	4.61E-13	0.930	0.985	v5.3	OASIS

RH_parstriangularis_surfavg	ICC2	0.839	14.42	19	19	1.47E-07	0.636	0.927	v5.3 vs. v6.0	OASIS
RH_parstriangularis_surfavg	ICC2	0.819	14.19	19	19	1.67E-07	0.539	0.922	v5.3 vs. v7.1	OASIS
RH_parstriangularis_surfavg	ICC3	0.982	112.78	19	19	1.30E-15	0.962	0.992	v6.0	OASIS
RH_parstriangularis_surfavg	ICC2	0.982	126.69	19	19	4.37E-16	0.960	0.992	v6.0 vs. v7.1	OASIS
RH_parstriangularis_surfavg	ICC3	0.989	188.77	19	19	1.03E-17	0.977	0.995	v7.1	OASIS
RH_parstriangularis_thickavg	ICC3	0.698	5.63	19	19	2.16E-04	0.444	0.849	v5.3	OASIS
RH_parstriangularis_thickavg	ICC2	0.821	13.81	19	19	2.10E-07	0.563	0.921	v5.3 vs. v6.0	OASIS
RH_parstriangularis_thickavg	ICC2	0.743	7.54	19	19	2.59E-05	0.513	0.873	v5.3 vs. v7.1	OASIS
RH_parstriangularis_thickavg	ICC3	0.902	19.45	19	19	1.14E-08	0.799	0.954	v6.0	OASIS
RH_parstriangularis_thickavg	ICC2	0.884	15.77	19	19	6.89E-08	0.766	0.945	v6.0 vs. v7.1	OASIS
RH_parstriangularis_thickavg	ICC3	0.802	9.11	19	19	6.09E-06	0.616	0.904	v7.1	OASIS
RH_pericalcarine_surfavg	ICC3	0.885	16.45	19	19	4.82E-08	0.767	0.945	v5.3	OASIS
RH_pericalcarine_surfavg	ICC2	0.943	64.04	19	19	2.50E-13	0.713	0.980	v5.3 vs. v6.0	OASIS
RH_pericalcarine_surfavg	ICC2	0.917	60.21	19	19	4.43E-13	0.416	0.974	v5.3 vs. v7.1	OASIS
RH_pericalcarine_surfavg	ICC3	0.993	303.47	19	19	1.18E-19	0.986	0.997	v6.0	OASIS
RH_pericalcarine_surfavg	ICC2	0.989	254.13	19	19	6.28E-19	0.964	0.996	v6.0 vs. v7.1	OASIS
RH_pericalcarine_surfavg	ICC3	0.989	177.91	19	19	1.81E-17	0.976	0.995	v7.1	OASIS
RH_pericalcarine_thickavg	ICC3	0.879	15.47	19	19	8.11E-08	0.754	0.942	v5.3	OASIS
RH_pericalcarine_thickavg	ICC2	0.766	14.12	19	19	1.75E-07	0.258	0.909	v5.3 vs. v6.0	OASIS
RH_pericalcarine_thickavg	ICC2	0.826	11.13	19	19	1.24E-06	0.660	0.916	v5.3 vs. v7.1	OASIS
RH_pericalcarine_thickavg	ICC3	0.734	6.53	19	19	7.53E-05	0.501	0.868	v6.0	OASIS
RH_pericalcarine_thickavg	ICC2	0.868	20.21	19	19	8.18E-09	0.634	0.944	v6.0 vs. v7.1	OASIS
RH_pericalcarine_thickavg	ICC3	0.907	20.40	19	19	7.54E-09	0.808	0.956	v7.1	OASIS
RH_postcentral_surfavg	ICC3	0.990	208.57	19	19	4.04E-18	0.979	0.996	v5.3	OASIS
RH_postcentral_surfavg	ICC2	0.919	23.16	19	19	2.49E-09	0.834	0.962	v5.3 vs. v6.0	OASIS
RH_postcentral_surfavg	ICC2	0.917	22.43	19	19	3.29E-09	0.830	0.961	v5.3 vs. v7.1	OASIS
RH_postcentral_surfavg	ICC3	0.990	206.99	19	19	4.34E-18	0.979	0.996	v6.0	OASIS
RH_postcentral_surfavg	ICC2	0.990	485.23	19	19	1.39E-21	0.886	0.997	v6.0 vs. v7.1	OASIS
RH_postcentral_surfavg	ICC3	0.991	229.71	19	19	1.63E-18	0.981	0.996	v7.1	OASIS
RH_postcentral_thickavg	ICC3	0.925	25.81	19	19	9.56E-10	0.845	0.965	v5.3	OASIS
RH_postcentral_thickavg	ICC2	0.803	20.17	19	19	8.33E-09	0.226	0.930	v5.3 vs. v6.0	OASIS
RH_postcentral_thickavg	ICC2	0.839	26.39	19	19	7.83E-10	0.265	0.944	v5.3 vs. v7.1	OASIS
RH_postcentral_thickavg	ICC3	0.958	46.53	19	19	4.71E-12	0.911	0.980	v6.0	OASIS
RH_postcentral_thickavg	ICC2	0.981	104.79	19	19	2.58E-15	0.960	0.991	v6.0 vs. v7.1	OASIS
RH_postcentral_thickavg	ICC3	0.984	120.91	19	19	6.77E-16	0.965	0.992	v7.1	OASIS
RH_posteriorcingulate_surfavg	ICC3	0.972	69.60	19	19	1.16E-13	0.940	0.987	v5.3	OASIS
RH_posteriorcingulate_surfavg	ICC2	0.963	50.24	19	19	2.34E-12	0.921	0.983	v5.3 vs. v6.0	OASIS
RH_posteriorcingulate_surfavg	ICC2	0.942	45.36	19	19	5.95E-12	0.839	0.976	v5.3 vs. v7.1	OASIS
RH_posteriorcingulate_surfavg	ICC3	0.981	101.89	19	19	3.35E-15	0.958	0.991	v6.0	OASIS
RH_posteriorcingulate_surfavg	ICC2	0.974	158.29	19	19	5.41E-17	0.817	0.991	v6.0 vs. v7.1	OASIS
RH_posteriorcingulate_surfavg	ICC3	0.972	69.58	19	19	1.16E-13	0.940	0.987	v7.1	OASIS
RH_posteriorcingulate_thickavg	ICC3	0.877	15.31	19	19	8.87E-08	0.752	0.941	v5.3	OASIS
RH_posteriorcingulate_thickavg	ICC2	0.904	32.65	19	19	1.17E-10	0.649	0.963	v5.3 vs. v6.0	OASIS
RH_posteriorcingulate_thickavg	ICC2	0.579	13.79	19	19	2.13E-07	-0.050	0.841	v5.3 vs. v7.1	OASIS

RH_posteriorcingulate_thickavg	ICC3	0.877	15.26	19	19	9.11E-08	0.751	0.941	v6.0	OASIS
RH_posteriorcingulate_thickavg	ICC2	0.730	25.48	19	19	1.07E-09	-0.013	0.911	v6.0 vs. v7.1	OASIS
RH_posteriorcingulate_thickavg	ICC3	0.915	22.66	19	19	3.01E-09	0.825	0.960	v7.1	OASIS
RH_precentral_surfavg	ICC3	0.982	109.77	19	19	1.67E-15	0.961	0.992	v5.3	OASIS
RH_precentral_surfavg	ICC2	0.804	9.41	19	19	4.72E-06	0.623	0.904	v5.3 vs. v6.0	OASIS
RH_precentral_surfavg	ICC2	0.780	7.96	19	19	1.73E-05	0.580	0.892	v5.3 vs. v7.1	OASIS
RH_precentral_surfavg	ICC3	0.993	284.18	19	19	2.19E-19	0.985	0.997	v6.0	OASIS
RH_precentral_surfavg	ICC2	0.985	140.73	19	19	1.63E-16	0.969	0.993	v6.0 vs. v7.1	OASIS
RH_precentral_surfavg	ICC3	0.989	176.62	19	19	1.93E-17	0.976	0.995	v7.1	OASIS
RH_precentral_thickavg	ICC3	0.955	43.73	19	19	8.30E-12	0.906	0.979	v5.3	OASIS
RH_precentral_thickavg	ICC2	0.724	15.85	19	19	6.61E-08	0.061	0.900	v5.3 vs. v6.0	OASIS
RH_precentral_thickavg	ICC2	0.714	12.19	19	19	5.89E-07	0.137	0.888	v5.3 vs. v7.1	OASIS
RH_precentral_thickavg	ICC3	0.948	37.47	19	19	3.38E-11	0.891	0.976	v6.0	OASIS
RH_precentral_thickavg	ICC2	0.961	48.92	19	19	2.98E-12	0.918	0.982	v6.0 vs. v7.1	OASIS
RH_precentral_thickavg	ICC3	0.968	62.39	19	19	3.19E-13	0.933	0.985	v7.1	OASIS
RH_precuneus_surfavg	ICC3	0.810	9.54	19	19	4.24E-06	0.630	0.908	v5.3	OASIS
RH_precuneus_surfavg	ICC2	0.973	69.40	19	19	1.19E-13	0.942	0.987	v5.3 vs. v6.0	OASIS
RH_precuneus_surfavg	ICC2	0.963	50.02	19	19	2.43E-12	0.921	0.983	v5.3 vs. v7.1	OASIS
RH_precuneus_surfavg	ICC3	0.989	183.51	19	19	1.35E-17	0.977	0.995	v6.0	OASIS
RH_precuneus_surfavg	ICC2	0.996	532.77	19	19	5.74E-22	0.992	0.998	v6.0 vs. v7.1	OASIS
RH_precuneus_surfavg	ICC3	0.988	169.47	19	19	2.85E-17	0.975	0.995	v7.1	OASIS
RH_precuneus_thickavg	ICC3	0.874	14.83	19	19	1.16E-07	0.745	0.940	v5.3	OASIS
RH_precuneus_thickavg	ICC2	0.916	24.63	19	19	1.44E-09	0.824	0.960	v5.3 vs. v6.0	OASIS
RH_precuneus_thickavg	ICC2	0.859	12.57	19	19	4.59E-07	0.716	0.932	v5.3 vs. v7.1	OASIS
RH_precuneus_thickavg	ICC3	0.934	29.35	19	19	3.04E-10	0.862	0.969	v6.0	OASIS
RH_precuneus_thickavg	ICC2	0.925	31.56	19	19	1.59E-10	0.824	0.967	v6.0 vs. v7.1	OASIS
RH_precuneus_thickavg	ICC3	0.907	20.43	19	19	7.45E-09	0.808	0.956	v7.1	OASIS
RH_Putamen	ICC3	0.973	73.78	19	19	6.75E-14	0.943	0.988	v5.3	OASIS
RH_Putamen	ICC2	0.406	51.25	19	19	1.95E-12	-0.009	0.747	v5.3 vs. v6.0	OASIS
RH_Putamen	ICC2	0.428	51.29	19	19	1.93E-12	-0.010	0.764	v5.3 vs. v7.1	OASIS
RH_Putamen	ICC3	0.981	105.61	19	19	2.40E-15	0.960	0.991	v6.0	OASIS
RH_Putamen	ICC2	0.987	212.51	19	19	3.39E-18	0.963	0.995	v6.0 vs. v7.1	OASIS
RH_Putamen	ICC3	0.978	89.57	19	19	1.11E-14	0.953	0.990	v7.1	OASIS
RH_rostralanteriorcingulate_surfavg	ICC3	0.895	18.11	19	19	2.11E-08	0.786	0.950	v5.3	OASIS
RH_rostralanteriorcingulate_surfavg	ICC2	0.785	19.15	19	19	1.30E-08	0.171	0.923	v5.3 vs. v6.0	OASIS
RH_rostralanteriorcingulate_surfavg	ICC2	0.825	11.89	19	19	7.27E-07	0.645	0.917	v5.3 vs. v7.1	OASIS
RH_rostralanteriorcingulate_surfavg	ICC3	0.928	26.91	19	19	6.60E-10	0.851	0.966	v6.0	OASIS
RH_rostralanteriorcingulate_surfavg	ICC2	0.922	50.08	19	19	2.41E-12	0.592	0.973	v6.0 vs. v7.1	OASIS
RH_rostralanteriorcingulate_surfavg	ICC3	0.892	17.48	19	19	2.86E-08	0.779	0.949	v7.1	OASIS
RH_rostralanteriorcingulate_thickavg	ICC3	0.542	3.36	19	19	5.60E-03	0.216	0.759	v5.3	OASIS
RH_rostralanteriorcingulate_thickavg	ICC2	0.530	4.32	19	19	1.25E-03	0.154	0.760	v5.3 vs. v6.0	OASIS
RH_rostralanteriorcingulate_thickavg	ICC2	0.420	2.44	19	19	2.93E-02	0.066	0.682	v5.3 vs. v7.1	OASIS
RH_rostralanteriorcingulate_thickavg	ICC3	0.335	2.01	19	19	6.90E-02	-0.039	0.626	v6.0	OASIS
RH_rostralanteriorcingulate_thickavg	ICC2	0.493	6.99	19	19	4.57E-05	-0.050	0.776	v6.0 vs. v7.1	OASIS

RH_rostralanteriorcingulate_thickavg	ICC3	0.471	2.78	19	19	1.57E-02	0.123	0.715	v7.1	OASIS
RH_rostralmiddlefrontal_surfavg	ICC3	0.966	58.31	19	19	5.95E-13	0.928	0.984	v5.3	OASIS
RH_rostralmiddlefrontal_surfavg	ICC2	0.928	28.24	19	19	4.29E-10	0.850	0.966	v5.3 vs. v6.0	OASIS
RH_rostralmiddlefrontal_surfavg	ICC2	0.833	18.19	19	19	2.04E-08	0.465	0.934	v5.3 vs. v7.1	OASIS
RH_rostralmiddlefrontal_surfavg	ICC3	0.987	149.52	19	19	9.25E-17	0.971	0.994	v6.0	OASIS
RH_rostralmiddlefrontal_surfavg	ICC2	0.939	77.39	19	19	4.34E-14	0.561	0.981	v6.0 vs. v7.1	OASIS
RH_rostralmiddlefrontal_surfavg	ICC3	0.983	115.24	19	19	1.06E-15	0.963	0.992	v7.1	OASIS
RH_rostralmiddlefrontal_thickavg	ICC3	0.840	11.54	19	19	9.25E-07	0.684	0.923	v5.3	OASIS
RH_rostralmiddlefrontal_thickavg	ICC2	0.845	12.23	19	19	5.77E-07	0.695	0.925	v5.3 vs. v6.0	OASIS
RH_rostralmiddlefrontal_thickavg	ICC2	0.674	5.21	19	19	3.65E-04	0.413	0.834	v5.3 vs. v7.1	OASIS
RH_rostralmiddlefrontal_thickavg	ICC3	0.866	13.94	19	19	1.95E-07	0.731	0.936	v6.0	OASIS
RH_rostralmiddlefrontal_thickavg	ICC2	0.898	18.01	19	19	2.21E-08	0.793	0.952	v6.0 vs. v7.1	OASIS
RH_rostralmiddlefrontal_thickavg	ICC3	0.909	21.07	19	19	5.70E-09	0.813	0.957	v7.1	OASIS
RH_superiorfrontal_surfavg	ICC3	0.989	183.45	19	19	1.35E-17	0.977	0.995	v5.3	OASIS
RH_superiorfrontal_surfavg	ICC2	0.948	72.58	19	19	7.86E-14	0.721	0.982	v5.3 vs. v6.0	OASIS
RH_superiorfrontal_surfavg	ICC2	0.909	55.68	19	19	9.09E-13	0.379	0.971	v5.3 vs. v7.1	OASIS
RH_superiorfrontal_surfavg	ICC3	0.990	198.14	19	19	6.56E-18	0.978	0.995	v6.0	OASIS
RH_superiorfrontal_surfavg	ICC2	0.985	237.08	19	19	1.21E-18	0.916	0.995	v6.0 vs. v7.1	OASIS
RH_superiorfrontal_surfavg	ICC3	0.993	270.71	19	19	3.46E-19	0.984	0.997	v7.1	OASIS
RH_superiorfrontal_thickavg	ICC3	0.840	11.48	19	19	9.66E-07	0.682	0.923	v5.3	OASIS
RH_superiorfrontal_thickavg	ICC2	0.867	13.48	19	19	2.57E-07	0.732	0.936	v5.3 vs. v6.0	OASIS
RH_superiorfrontal_thickavg	ICC2	0.846	12.07	19	19	6.43E-07	0.698	0.925	v5.3 vs. v7.1	OASIS
RH_superiorfrontal_thickavg	ICC3	0.903	19.72	19	19	1.01E-08	0.802	0.954	v6.0	OASIS
RH_superiorfrontal_thickavg	ICC2	0.926	26.17	19	19	8.46E-10	0.848	0.965	v6.0 vs. v7.1	OASIS
RH_superiorfrontal_thickavg	ICC3	0.889	16.94	19	19	3.74E-08	0.773	0.947	v7.1	OASIS
RH_superiorparietal_surfavg	ICC3	0.978	88.13	19	19	1.30E-14	0.952	0.990	v5.3	OASIS
RH_superiorparietal_surfavg	ICC2	0.908	20.00	19	19	8.97E-09	0.812	0.957	v5.3 vs. v6.0	OASIS
RH_superiorparietal_surfavg	ICC2	0.905	19.49	19	19	1.12E-08	0.806	0.955	v5.3 vs. v7.1	OASIS
RH_superiorparietal_surfavg	ICC3	0.993	297.12	19	19	1.44E-19	0.986	0.997	v6.0	OASIS
RH_superiorparietal_surfavg	ICC2	0.990	376.50	19	19	1.53E-20	0.932	0.996	v6.0 vs. v7.1	OASIS
RH_superiorparietal_surfavg	ICC3	0.992	256.19	19	19	5.82E-19	0.983	0.996	v7.1	OASIS
RH_superiorparietal_thickavg	ICC3	0.867	14.06	19	19	1.81E-07	0.733	0.936	v5.3	OASIS
RH_superiorparietal_thickavg	ICC2	0.842	22.03	19	19	3.86E-09	0.399	0.941	v5.3 vs. v6.0	OASIS
RH_superiorparietal_thickavg	ICC2	0.825	17.64	19	19	2.65E-08	0.435	0.931	v5.3 vs. v7.1	OASIS
RH_superiorparietal_thickavg	ICC3	0.927	26.52	19	19	7.51E-10	0.849	0.966	v6.0	OASIS
RH_superiorparietal_thickavg	ICC2	0.936	28.73	19	19	3.68E-10	0.866	0.970	v6.0 vs. v7.1	OASIS
RH_superiorparietal_thickavg	ICC3	0.936	30.12	19	19	2.41E-10	0.866	0.970	v7.1	OASIS
RH_superiortemporal_surfavg	ICC3	0.976	82.20	19	19	2.48E-14	0.949	0.989	v5.3	OASIS
RH_superiortemporal_surfavg	ICC2	0.933	60.31	19	19	4.35E-13	0.621	0.977	v5.3 vs. v6.0	OASIS
RH_superiortemporal_surfavg	ICC2	0.887	43.85	19	19	8.10E-12	0.310	0.964	v5.3 vs. v7.1	OASIS
RH_superiortemporal_surfavg	ICC3	0.993	277.31	19	19	2.76E-19	0.984	0.997	v6.0	OASIS
RH_superiortemporal_surfavg	ICC2	0.984	229.05	19	19	1.67E-18	0.905	0.994	v6.0 vs. v7.1	OASIS
RH_superiortemporal_surfavg	ICC3	0.992	236.82	19	19	1.22E-18	0.982	0.996	v7.1	OASIS
RH_superiortemporal_thickavg	ICC3	0.894	17.85	19	19	2.39E-08	0.783	0.950	v5.3	OASIS

RH_superiortemporal_thickavg	ICC2	0.852	18.64	19	19	1.65E-08	0.578	0.939	v5.3 vs. v6.0	OASIS
RH_superiortemporal_thickavg	ICC2	0.831	13.69	19	19	2.26E-07	0.616	0.923	v5.3 vs. v7.1	OASIS
RH_superiortemporal_thickavg	ICC3	0.960	48.60	19	19	3.17E-12	0.915	0.981	v6.0	OASIS
RH_superiortemporal_thickavg	ICC2	0.894	17.11	19	19	3.43E-08	0.783	0.950	v6.0 vs. v7.1	OASIS
RH_superiortemporal_thickavg	ICC3	0.894	17.95	19	19	2.28E-08	0.784	0.950	v7.1	OASIS
RH_supramarginal_surfavg	ICC3	0.983	117.66	19	19	8.73E-16	0.964	0.992	v5.3	OASIS
RH_supramarginal_surfavg	ICC2	0.903	20.38	19	19	7.59E-09	0.802	0.954	v5.3 vs. v6.0	OASIS
RH_supramarginal_surfavg	ICC2	0.895	17.22	19	19	3.25E-08	0.785	0.950	v5.3 vs. v7.1	OASIS
RH_supramarginal_surfavg	ICC3	0.993	292.35	19	19	1.67E-19	0.985	0.997	v6.0	OASIS
RH_supramarginal_surfavg	ICC2	0.988	510.77	19	19	8.56E-22	0.797	0.996	v6.0 vs. v7.1	OASIS
RH_supramarginal_surfavg	ICC3	0.991	228.65	19	19	1.70E-18	0.981	0.996	v7.1	OASIS
RH_supramarginal_thickavg	ICC3	0.914	22.27	19	19	3.51E-09	0.823	0.959	v5.3	OASIS
RH_supramarginal_thickavg	ICC2	0.843	12.23	19	19	5.76E-07	0.691	0.924	v5.3 vs. v6.0	OASIS
RH_supramarginal_thickavg	ICC2	0.841	11.06	19	19	1.31E-06	0.683	0.923	v5.3 vs. v7.1	OASIS
RH_supramarginal_thickavg	ICC3	0.924	25.27	19	19	1.15E-09	0.842	0.964	v6.0	OASIS
RH_supramarginal_thickavg	ICC2	0.903	21.44	19	19	4.88E-09	0.797	0.954	v6.0 vs. v7.1	OASIS
RH_supramarginal_thickavg	ICC3	0.909	20.93	19	19	6.02E-09	0.812	0.957	v7.1	OASIS
RH_SurfArea	ICC3	0.993	302.46	19	19	1.21E-19	0.986	0.997	v5.3	OASIS
RH_SurfArea	ICC2	0.981	387.13	19	19	1.18E-20	0.642	0.995	v5.3 vs. v6.0	OASIS
RH_SurfArea	ICC2	0.920	324.56	19	19	6.24E-20	0.056	0.980	v5.3 vs. v7.1	OASIS
RH_SurfArea	ICC3	0.995	393.90	19	19	1.00E-20	0.989	0.998	v6.0	OASIS
RH_SurfArea	ICC2	0.972	814.17	19	19	1.03E-23	0.211	0.993	v6.0 vs. v7.1	OASIS
RH_SurfArea	ICC3	0.995	413.63	19	19	6.30E-21	0.990	0.998	v7.1	OASIS
RH_temporalpole_surfavg	ICC3	0.701	5.68	19	19	2.01E-04	0.448	0.850	v5.3	OASIS
RH_temporalpole_surfavg	ICC2	0.603	10.32	19	19	2.27E-06	-0.020	0.843	v5.3 vs. v6.0	OASIS
RH_temporalpole_surfavg	ICC2	0.316	4.68	19	19	7.45E-04	-0.073	0.638	v5.3 vs. v7.1	OASIS
RH_temporalpole_surfavg	ICC3	0.793	8.65	19	19	9.11E-06	0.599	0.899	v6.0	OASIS
RH_temporalpole_surfavg	ICC2	0.716	9.34	19	19	5.02E-06	0.304	0.877	v6.0 vs. v7.1	OASIS
RH_temporalpole_surfavg	ICC3	0.879	15.59	19	19	7.61E-08	0.756	0.943	v7.1	OASIS
RH_temporalpole_thickavg	ICC3	0.294	1.83	19	19	9.75E-02	-0.083	0.598	v5.3	OASIS
RH_temporalpole_thickavg	ICC2	0.856	12.53	19	19	4.72E-07	0.713	0.931	v5.3 vs. v6.0	OASIS
RH_temporalpole_thickavg	ICC2	0.723	7.11	19	19	4.05E-05	0.472	0.863	v5.3 vs. v7.1	OASIS
RH_temporalpole_thickavg	ICC3	0.650	4.72	19	19	7.10E-04	0.370	0.822	v6.0	OASIS
RH_temporalpole_thickavg	ICC2	0.843	14.99	19	19	1.05E-07	0.638	0.929	v6.0 vs. v7.1	OASIS
RH_temporalpole_thickavg	ICC3	0.489	2.91	19	19	1.22E-02	0.147	0.727	v7.1	OASIS
RH_Thalamus	ICC3	0.951	40.18	19	19	1.79E-11	0.898	0.977	v5.3	OASIS
RH_Thalamus	ICC2	0.944	39.41	19	19	2.14E-11	0.876	0.974	v5.3 vs. v6.0	OASIS
RH_Thalamus	ICC2	0.936	29.60	19	19	2.82E-10	0.867	0.970	v5.3 vs. v7.1	OASIS
RH_Thalamus	ICC3	0.971	67.24	19	19	1.59E-13	0.938	0.986	v6.0	OASIS
RH_Thalamus	ICC2	0.959	48.92	19	19	2.98E-12	0.914	0.981	v6.0 vs. v7.1	OASIS
RH_Thalamus	ICC3	0.926	26.12	19	19	8.60E-10	0.847	0.965	v7.1	OASIS
RH_Thickness	ICC3	0.846	11.95	19	19	6.95E-07	0.693	0.926	v5.3	OASIS
RH_Thickness	ICC2	0.801	15.93	19	19	6.31E-08	0.360	0.922	v5.3 vs. v6.0	OASIS
RH_Thickness	ICC2	0.813	10.13	19	19	2.65E-06	0.638	0.909	v5.3 vs. v7.1	OASIS

RH_Thickness	ICC3	0.941	32.73	19	19	1.15E-10	0.876	0.972	v6.0	OASIS
RH_Thickness	ICC2	0.867	19.41	19	19	1.16E-08	0.649	0.943	v6.0 vs. v7.1	OASIS
RH_Thickness	ICC3	0.927	26.48	19	19	7.61E-10	0.849	0.966	v7.1	OASIS
RH_transversetemporal_surfavg	ICC3	0.905	20.00	19	19	8.95E-09	0.804	0.955	v5.3	OASIS
RH_transversetemporal_surfavg	ICC2	0.731	6.29	19	19	9.89E-05	0.497	0.866	v5.3 vs. v6.0	OASIS
RH_transversetemporal_surfavg	ICC2	0.705	5.56	19	19	2.34E-04	0.451	0.853	v5.3 vs. v7.1	OASIS
RH_transversetemporal_surfavg	ICC3	0.914	22.28	19	19	3.49E-09	0.823	0.959	v6.0	OASIS
RH_transversetemporal_surfavg	ICC2	0.933	33.38	19	19	9.60E-11	0.853	0.970	v6.0 vs. v7.1	OASIS
RH_transversetemporal_surfavg	ICC3	0.977	85.42	19	19	1.73E-14	0.950	0.989	v7.1	OASIS
RH_transversetemporal_thickavg	ICC3	0.934	29.25	19	19	3.14E-10	0.862	0.969	v5.3	OASIS
RH_transversetemporal_thickavg	ICC2	0.783	21.44	19	19	4.88E-09	0.114	0.925	v5.3 vs. v6.0	OASIS
RH_transversetemporal_thickavg	ICC2	0.854	26.96	19	19	6.48E-10	0.351	0.949	v5.3 vs. v7.1	OASIS
RH_transversetemporal_thickavg	ICC3	0.914	22.25	19	19	3.53E-09	0.822	0.959	v6.0	OASIS
RH_transversetemporal_thickavg	ICC2	0.924	27.99	19	19	4.65E-10	0.839	0.965	v6.0 vs. v7.1	OASIS
RH_transversetemporal_thickavg	ICC3	0.967	58.88	19	19	5.43E-13	0.929	0.984	v7.1	OASIS
SUM_bankssts_surfavg	ICC3	0.953	41.91	19	19	1.22E-11	0.902	0.978	v5.3	OASIS
SUM_bankssts_surfavg	ICC2	0.737	7.60	19	19	2.46E-05	0.493	0.871	v5.3 vs. v6.0	OASIS
SUM_bankssts_surfavg	ICC2	0.697	5.98	19	19	1.41E-04	0.448	0.847	v5.3 vs. v7.1	OASIS
SUM_bankssts_surfavg	ICC3	0.953	41.63	19	19	1.30E-11	0.901	0.978	v6.0	OASIS
SUM_bankssts_surfavg	ICC2	0.943	33.92	19	19	8.30E-11	0.883	0.973	v6.0 vs. v7.1	OASIS
SUM_bankssts_surfavg	ICC3	0.898	18.54	19	19	1.72E-08	0.791	0.951	v7.1	OASIS
SUM_caudalanteriorcingulate_surfavg	ICC3	0.987	151.00	19	19	8.43E-17	0.972	0.994	v5.3	OASIS
SUM_caudalanteriorcingulate_surfavg	ICC2	0.886	42.90	19	19	9.88E-12	0.316	0.963	v5.3 vs. v6.0	OASIS
SUM_caudalanteriorcingulate_surfavg	ICC2	0.937	29.70	19	19	2.74E-10	0.869	0.970	v5.3 vs. v7.1	OASIS
SUM_caudalanteriorcingulate_surfavg	ICC3	0.969	64.35	19	19	2.39E-13	0.935	0.986	v6.0	OASIS
SUM_caudalanteriorcingulate_surfavg	ICC2	0.909	82.38	19	19	2.43E-14	0.187	0.974	v6.0 vs. v7.1	OASIS
SUM_caudalanteriorcingulate_surfavg	ICC3	0.964	54.18	19	19	1.17E-12	0.923	0.983	v7.1	OASIS
SUM_caudalmiddlefrontal_surfavg	ICC3	0.977	87.74	19	19	1.35E-14	0.952	0.990	v5.3	OASIS
SUM_caudalmiddlefrontal_surfavg	ICC2	0.945	38.17	19	19	2.86E-11	0.883	0.974	v5.3 vs. v6.0	OASIS
SUM_caudalmiddlefrontal_surfavg	ICC2	0.947	40.32	19	19	1.74E-11	0.885	0.975	v5.3 vs. v7.1	OASIS
SUM_caudalmiddlefrontal_surfavg	ICC3	0.988	170.09	19	19	2.76E-17	0.975	0.995	v6.0	OASIS
SUM_caudalmiddlefrontal_surfavg	ICC2	0.987	145.41	19	19	1.20E-16	0.972	0.994	v6.0 vs. v7.1	OASIS
SUM_caudalmiddlefrontal_surfavg	ICC3	0.975	78.34	19	19	3.87E-14	0.946	0.988	v7.1	OASIS
SUM_cuneus_surfavg	ICC3	0.963	53.47	19	19	1.32E-12	0.922	0.983	v5.3	OASIS
SUM_cuneus_surfavg	ICC2	0.874	29.02	19	19	3.37E-10	0.459	0.955	v5.3 vs. v6.0	OASIS
SUM_cuneus_surfavg	ICC2	0.765	30.09	19	19	2.43E-10	0.003	0.925	v5.3 vs. v7.1	OASIS
SUM_cuneus_surfavg	ICC3	0.994	333.93	19	19	4.77E-20	0.987	0.997	v6.0	OASIS
SUM_cuneus_surfavg	ICC2	0.946	131.71	19	19	3.04E-16	0.369	0.985	v6.0 vs. v7.1	OASIS
SUM_cuneus_surfavg	ICC3	0.996	485.95	19	19	1.37E-21	0.991	0.998	v7.1	OASIS
SUM_entorhinal_surfavg	ICC3	0.780	8.10	19	19	1.51E-05	0.578	0.892	v5.3	OASIS
SUM_entorhinal_surfavg	ICC2	0.398	4.74	19	19	6.88E-04	-0.059	0.700	v5.3 vs. v6.0	OASIS
SUM_entorhinal_surfavg	ICC2	0.489	5.82	19	19	1.71E-04	-0.026	0.764	v5.3 vs. v7.1	OASIS
SUM_entorhinal_surfavg	ICC3	0.964	54.58	19	19	1.09E-12	0.924	0.983	v6.0	OASIS
SUM_entorhinal_surfavg	ICC2	0.773	9.51	19	19	4.34E-06	0.532	0.893	v6.0 vs. v7.1	OASIS

SUM_entorhinal_surfavg	ICC3	0.888	16.92	19	19	3.79E-08	0.773	0.947	v7.1	OASIS
SUM_frontalpole_surfavg	ICC3	0.822	10.26	19	19	2.39E-06	0.651	0.914	v5.3	OASIS
SUM_frontalpole_surfavg	ICC2	0.417	5.55	19	19	2.37E-04	-0.064	0.721	v5.3 vs. v6.0	OASIS
SUM_frontalpole_surfavg	ICC2	0.243	5.52	19	19	2.47E-04	-0.054	0.568	v5.3 vs. v7.1	OASIS
SUM_frontalpole_surfavg	ICC3	0.924	25.35	19	19	1.12E-09	0.842	0.964	v6.0	OASIS
SUM_frontalpole_surfavg	ICC2	0.693	37.19	19	19	3.62E-11	-0.027	0.902	v6.0 vs. v7.1	OASIS
SUM_frontalpole_surfavg	ICC3	0.895	17.97	19	19	2.26E-08	0.785	0.950	v7.1	OASIS
SUM_fusiform_surfavg	ICC3	0.978	88.08	19	19	1.30E-14	0.952	0.990	v5.3	OASIS
SUM_fusiform_surfavg	ICC2	0.860	46.19	19	19	5.04E-12	0.124	0.957	v5.3 vs. v6.0	OASIS
SUM_fusiform_surfavg	ICC2	0.876	32.67	19	19	1.16E-10	0.398	0.957	v5.3 vs. v7.1	OASIS
SUM_fusiform_surfavg	ICC3	0.986	146.18	19	19	1.14E-16	0.971	0.994	v6.0	OASIS
SUM_fusiform_surfavg	ICC2	0.983	150.98	19	19	8.44E-17	0.956	0.993	v6.0 vs. v7.1	OASIS
SUM_fusiform_surfavg	ICC3	0.991	226.86	19	19	1.83E-18	0.981	0.996	v7.1	OASIS
SUM_inferiorparietal_surfavg	ICC3	0.995	426.80	19	19	4.69E-21	0.990	0.998	v5.3	OASIS
SUM_inferiorparietal_surfavg	ICC2	0.918	36.99	19	19	3.79E-11	0.713	0.968	v5.3 vs. v6.0	OASIS
SUM_inferiorparietal_surfavg	ICC2	0.932	35.28	19	19	5.82E-11	0.836	0.970	v5.3 vs. v7.1	OASIS
SUM_inferiorparietal_surfavg	ICC3	0.991	229.19	19	19	1.66E-18	0.981	0.996	v6.0	OASIS
SUM_inferiorparietal_surfavg	ICC2	0.990	267.62	19	19	3.86E-19	0.968	0.996	v6.0 vs. v7.1	OASIS
SUM_inferiorparietal_surfavg	ICC3	0.979	94.39	19	19	6.84E-15	0.955	0.990	v7.1	OASIS
SUM_inferiortemporal_surfavg	ICC3	0.983	117.45	19	19	8.88E-16	0.964	0.992	v5.3	OASIS
SUM_inferiortemporal_surfavg	ICC2	0.909	45.20	19	19	6.14E-12	0.507	0.969	v5.3 vs. v6.0	OASIS
SUM_inferiortemporal_surfavg	ICC2	0.853	36.49	19	19	4.30E-11	0.182	0.953	v5.3 vs. v7.1	OASIS
SUM_inferiortemporal_surfavg	ICC3	0.989	175.25	19	19	2.08E-17	0.976	0.995	v6.0	OASIS
SUM_inferiortemporal_surfavg	ICC2	0.981	234.68	19	19	1.33E-18	0.851	0.994	v6.0 vs. v7.1	OASIS
SUM_inferiortemporal_surfavg	ICC3	0.990	206.66	19	19	4.41E-18	0.979	0.996	v7.1	OASIS
SUM_insula_surfavg	ICC3	0.963	53.72	19	19	1.26E-12	0.922	0.983	v5.3	OASIS
SUM_insula_surfavg	ICC2	0.537	32.91	19	19	1.09E-10	-0.024	0.830	v5.3 vs. v6.0	OASIS
SUM_insula_surfavg	ICC2	0.600	20.47	19	19	7.32E-09	-0.042	0.857	v5.3 vs. v7.1	OASIS
SUM_insula_surfavg	ICC3	0.820	10.14	19	19	2.62E-06	0.648	0.913	v6.0	OASIS
SUM_insula_surfavg	ICC2	0.873	15.07	19	19	1.01E-07	0.748	0.939	v6.0 vs. v7.1	OASIS
SUM_insula_surfavg	ICC3	0.869	14.27	19	19	1.60E-07	0.736	0.937	v7.1	OASIS
SUM_isthmuscingulate_surfavg	ICC3	0.962	51.47	19	19	1.87E-12	0.919	0.982	v5.3	OASIS
SUM_isthmuscingulate_surfavg	ICC2	0.947	52.46	19	19	1.57E-12	0.834	0.978	v5.3 vs. v6.0	OASIS
SUM_isthmuscingulate_surfavg	ICC2	0.908	42.82	19	19	1.01E-11	0.524	0.968	v5.3 vs. v7.1	OASIS
SUM_isthmuscingulate_surfavg	ICC3	0.990	195.44	19	19	7.46E-18	0.978	0.995	v6.0	OASIS
SUM_isthmuscingulate_surfavg	ICC2	0.985	282.76	19	19	2.29E-19	0.888	0.995	v6.0 vs. v7.1	OASIS
SUM_isthmuscingulate_surfavg	ICC3	0.975	78.21	19	19	3.93E-14	0.946	0.988	v7.1	OASIS
SUM_lateraloccipital_surfavg	ICC3	0.979	94.70	19	19	6.63E-15	0.955	0.990	v5.3	OASIS
SUM_lateraloccipital_surfavg	ICC2	0.690	21.06	19	19	5.70E-09	-0.026	0.894	v5.3 vs. v6.0	OASIS
SUM_lateraloccipital_surfavg	ICC2	0.597	18.58	19	19	1.69E-08	-0.045	0.854	v5.3 vs. v7.1	OASIS
SUM_lateraloccipital_surfavg	ICC3	0.992	239.57	19	19	1.10E-18	0.982	0.996	v6.0	OASIS
SUM_lateraloccipital_surfavg	ICC2	0.978	357.92	19	19	2.48E-20	0.582	0.994	v6.0 vs. v7.1	OASIS
SUM_lateraloccipital_surfavg	ICC3	0.992	253.02	19	19	6.55E-19	0.983	0.996	v7.1	OASIS
SUM_lateralorbitofrontal_surfavg	ICC3	0.896	18.17	19	19	2.06E-08	0.787	0.950	v5.3	OASIS

SUM_lateralorbitofrontal_surfavg	ICC2	0.893	36.80	19	19	3.98E-11	0.471	0.963	v5.3 vs. v6.0	OASIS
SUM_lateralorbitofrontal_surfavg	ICC2	0.729	31.15	19	19	1.79E-10	-0.021	0.913	v5.3 vs. v7.1	OASIS
SUM_lateralorbitofrontal_surfavg	ICC3	0.944	34.98	19	19	6.30E-11	0.883	0.974	v6.0	OASIS
SUM_lateralorbitofrontal_surfavg	ICC2	0.862	35.52	19	19	5.49E-11	0.245	0.955	v6.0 vs. v7.1	OASIS
SUM_lateralorbitofrontal_surfavg	ICC3	0.943	34.26	19	19	7.59E-11	0.881	0.973	v7.1	OASIS
SUM_lingual_surfavg	ICC3	0.987	150.51	19	19	8.69E-17	0.972	0.994	v5.3	OASIS
SUM_lingual_surfavg	ICC2	0.952	41.66	19	19	1.29E-11	0.900	0.978	v5.3 vs. v6.0	OASIS
SUM_lingual_surfavg	ICC2	0.919	38.41	19	19	2.70E-11	0.702	0.969	v5.3 vs. v7.1	OASIS
SUM_lingual_surfavg	ICC3	0.996	460.13	19	19	2.30E-21	0.991	0.998	v6.0	OASIS
SUM_lingual_surfavg	ICC2	0.981	395.33	19	19	9.67E-21	0.647	0.995	v6.0 vs. v7.1	OASIS
SUM_lingual_surfavg	ICC3	0.996	526.94	19	19	6.38E-22	0.992	0.998	v7.1	OASIS
SUM_medialorbitofrontal_surfavg	ICC3	0.860	13.25	19	19	2.97E-07	0.719	0.933	v5.3	OASIS
SUM_medialorbitofrontal_surfavg	ICC2	0.800	13.29	19	19	2.89E-07	0.474	0.915	v5.3 vs. v6.0	OASIS
SUM_medialorbitofrontal_surfavg	ICC2	0.382	16.17	19	19	5.57E-08	-0.032	0.722	v5.3 vs. v7.1	OASIS
SUM_medialorbitofrontal_surfavg	ICC3	0.922	24.52	19	19	1.50E-09	0.838	0.963	v6.0	OASIS
SUM_medialorbitofrontal_surfavg	ICC2	0.531	32.62	19	19	1.18E-10	-0.024	0.826	v6.0 vs. v7.1	OASIS
SUM_medialorbitofrontal_surfavg	ICC3	0.926	26.03	19	19	8.87E-10	0.846	0.965	v7.1	OASIS
SUM_middletemporal_surfavg	ICC3	0.987	157.67	19	19	5.62E-17	0.973	0.994	v5.3	OASIS
SUM_middletemporal_surfavg	ICC2	0.904	105.41	19	19	2.44E-15	0.103	0.973	v5.3 vs. v6.0	OASIS
SUM_middletemporal_surfavg	ICC2	0.836	106.09	19	19	2.30E-15	0.007	0.954	v5.3 vs. v7.1	OASIS
SUM_middletemporal_surfavg	ICC3	0.993	304.54	19	19	1.14E-19	0.986	0.997	v6.0	OASIS
SUM_middletemporal_surfavg	ICC2	0.979	268.30	19	19	3.76E-19	0.751	0.994	v6.0 vs. v7.1	OASIS
SUM_middletemporal_surfavg	ICC3	0.990	193.37	19	19	8.25E-18	0.978	0.995	v7.1	OASIS
SUM_paracentral_surfavg	ICC3	0.981	102.33	19	19	3.22E-15	0.959	0.991	v5.3	OASIS
SUM_paracentral_surfavg	ICC2	0.910	21.17	19	19	5.45E-09	0.817	0.957	v5.3 vs. v6.0	OASIS
SUM_paracentral_surfavg	ICC2	0.927	25.35	19	19	1.12E-09	0.849	0.966	v5.3 vs. v7.1	OASIS
SUM_paracentral_surfavg	ICC3	0.987	149.43	19	19	9.30E-17	0.971	0.994	v6.0	OASIS
SUM_paracentral_surfavg	ICC2	0.990	278.14	19	19	2.68E-19	0.969	0.996	v6.0 vs. v7.1	OASIS
SUM_paracentral_surfavg	ICC3	0.978	92.01	19	19	8.68E-15	0.954	0.990	v7.1	OASIS
SUM_parahippocampal_surfavg	ICC3	0.937	30.53	19	19	2.14E-10	0.867	0.970	v5.3	OASIS
SUM_parahippocampal_surfavg	ICC2	0.659	9.69	19	19	3.75E-06	0.088	0.861	v5.3 vs. v6.0	OASIS
SUM_parahippocampal_surfavg	ICC2	0.494	6.51	19	19	7.66E-05	-0.041	0.773	v5.3 vs. v7.1	OASIS
SUM_parahippocampal_surfavg	ICC3	0.944	34.46	19	19	7.21E-11	0.882	0.974	v6.0	OASIS
SUM_parahippocampal_surfavg	ICC2	0.866	21.68	19	19	4.43E-09	0.583	0.946	v6.0 vs. v7.1	OASIS
SUM_parahippocampal_surfavg	ICC3	0.957	45.14	19	19	6.21E-12	0.908	0.980	v7.1	OASIS
SUM_parsopercularis_surfavg	ICC3	0.975	79.42	19	19	3.41E-14	0.947	0.988	v5.3	OASIS
SUM_parsopercularis_surfavg	ICC2	0.937	42.04	19	19	1.19E-11	0.822	0.974	v5.3 vs. v6.0	OASIS
SUM_parsopercularis_surfavg	ICC2	0.950	44.80	19	19	6.65E-12	0.888	0.977	v5.3 vs. v7.1	OASIS
SUM_parsopercularis_surfavg	ICC3	0.983	115.22	19	19	1.06E-15	0.963	0.992	v6.0	OASIS
SUM_parsopercularis_surfavg	ICC2	0.990	275.92	19	19	2.89E-19	0.972	0.996	v6.0 vs. v7.1	OASIS
SUM_parsopercularis_surfavg	ICC3	0.988	170.48	19	19	2.70E-17	0.975	0.995	v7.1	OASIS
SUM_parsorbitalis_surfavg	ICC3	0.924	25.37	19	19	1.11E-09	0.843	0.964	v5.3	OASIS
SUM_parsorbitalis_surfavg	ICC2	0.509	21.79	19	19	4.25E-09	-0.035	0.811	v5.3 vs. v6.0	OASIS
SUM_parsorbitalis_surfavg	ICC2	0.402	20.57	19	19	7.02E-09	-0.027	0.740	v5.3 vs. v7.1	OASIS

SUM_parsorbitalis_surfavg	ICC3	0.960	48.53	19	19	3.21E-12	0.914	0.981	v6.0	OASIS
SUM_parsorbitalis_surfavg	ICC2	0.924	82.01	19	19	2.53E-14	0.325	0.977	v6.0 vs. v7.1	OASIS
SUM_parsorbitalis_surfavg	ICC3	0.974	77.07	19	19	4.50E-14	0.945	0.988	v7.1	OASIS
SUM_parstriangularis_surfavg	ICC3	0.974	74.58	19	19	6.11E-14	0.943	0.988	v5.3	OASIS
SUM_parstriangularis_surfavg	ICC2	0.923	36.63	19	19	4.15E-11	0.760	0.969	v5.3 vs. v6.0	OASIS
SUM_parstriangularis_surfavg	ICC2	0.908	42.89	19	19	9.90E-12	0.532	0.968	v5.3 vs. v7.1	OASIS
SUM_parstriangularis_surfavg	ICC3	0.989	188.48	19	19	1.05E-17	0.977	0.995	v6.0	OASIS
SUM_parstriangularis_surfavg	ICC2	0.988	245.60	19	19	8.67E-19	0.957	0.995	v6.0 vs. v7.1	OASIS
SUM_parstriangularis_surfavg	ICC3	0.993	281.61	19	19	2.38E-19	0.985	0.997	v7.1	OASIS
SUM_pericalcarine_surfavg	ICC3	0.960	48.54	19	19	3.20E-12	0.914	0.981	v5.3	OASIS
SUM_pericalcarine_surfavg	ICC2	0.949	76.10	19	19	5.07E-14	0.710	0.982	v5.3 vs. v6.0	OASIS
SUM_pericalcarine_surfavg	ICC2	0.911	52.03	19	19	1.69E-12	0.434	0.971	v5.3 vs. v7.1	OASIS
SUM_pericalcarine_surfavg	ICC3	0.993	288.97	19	19	1.87E-19	0.985	0.997	v6.0	OASIS
SUM_pericalcarine_surfavg	ICC2	0.987	246.96	19	19	8.23E-19	0.944	0.995	v6.0 vs. v7.1	OASIS
SUM_pericalcarine_surfavg	ICC3	0.990	193.95	19	19	8.02E-18	0.978	0.995	v7.1	OASIS
SUM_postcentral_surfavg	ICC3	0.984	127.63	19	19	4.08E-16	0.967	0.993	v5.3	OASIS
SUM_postcentral_surfavg	ICC2	0.920	27.17	19	19	6.05E-10	0.827	0.963	v5.3 vs. v6.0	OASIS
SUM_postcentral_surfavg	ICC2	0.926	24.91	19	19	1.31E-09	0.846	0.965	v5.3 vs. v7.1	OASIS
SUM_postcentral_surfavg	ICC3	0.990	202.26	19	19	5.40E-18	0.979	0.995	v6.0	OASIS
SUM_postcentral_surfavg	ICC2	0.985	399.91	19	19	8.67E-21	0.797	0.996	v6.0 vs. v7.1	OASIS
SUM_postcentral_surfavg	ICC3	0.988	169.65	19	19	2.82E-17	0.975	0.995	v7.1	OASIS
SUM_posteriorcingulate_surfavg	ICC3	0.975	79.94	19	19	3.21E-14	0.947	0.989	v5.3	OASIS
SUM_posteriorcingulate_surfavg	ICC2	0.978	89.54	19	19	1.12E-14	0.954	0.990	v5.3 vs. v6.0	OASIS
SUM_posteriorcingulate_surfavg	ICC2	0.961	109.08	19	19	1.77E-15	0.729	0.987	v5.3 vs. v7.1	OASIS
SUM_posteriorcingulate_surfavg	ICC3	0.986	137.47	19	19	2.03E-16	0.969	0.993	v6.0	OASIS
SUM_posteriorcingulate_surfavg	ICC2	0.980	421.79	19	19	5.24E-21	0.566	0.994	v6.0 vs. v7.1	OASIS
SUM_posteriorcingulate_surfavg	ICC3	0.984	121.22	19	19	6.61E-16	0.965	0.992	v7.1	OASIS
SUM_precentral_surfavg	ICC3	0.870	14.44	19	19	1.45E-07	0.739	0.938	v5.3	OASIS
SUM_precentral_surfavg	ICC2	0.912	20.74	19	19	6.52E-09	0.818	0.959	v5.3 vs. v6.0	OASIS
SUM_precentral_surfavg	ICC2	0.915	21.92	19	19	4.03E-09	0.826	0.960	v5.3 vs. v7.1	OASIS
SUM_precentral_surfavg	ICC3	0.992	244.30	19	19	9.11E-19	0.982	0.996	v6.0	OASIS
SUM_precentral_surfavg	ICC2	0.990	233.51	19	19	1.40E-18	0.978	0.996	v6.0 vs. v7.1	OASIS
SUM_precentral_surfavg	ICC3	0.972	70.65	19	19	1.01E-13	0.940	0.987	v7.1	OASIS
SUM_precuneus_surfavg	ICC3	0.922	24.64	19	19	1.44E-09	0.838	0.963	v5.3	OASIS
SUM_precuneus_surfavg	ICC2	0.985	147.97	19	19	1.02E-16	0.967	0.993	v5.3 vs. v6.0	OASIS
SUM_precuneus_surfavg	ICC2	0.980	114.87	19	19	1.09E-15	0.955	0.991	v5.3 vs. v7.1	OASIS
SUM_precuneus_surfavg	ICC3	0.991	222.09	19	19	2.24E-18	0.981	0.996	v6.0	OASIS
SUM_precuneus_surfavg	ICC2	0.997	648.10	19	19	8.98E-23	0.993	0.999	v6.0 vs. v7.1	OASIS
SUM_precuneus_surfavg	ICC3	0.948	37.36	19	19	3.47E-11	0.890	0.976	v7.1	OASIS
SUM_rostralanteriorcingulate_surfavg	ICC3	0.948	37.53	19	19	3.33E-11	0.891	0.976	v5.3	OASIS
SUM_rostralanteriorcingulate_surfavg	ICC2	0.894	38.69	19	19	2.53E-11	0.452	0.964	v5.3 vs. v6.0	OASIS
SUM_rostralanteriorcingulate_surfavg	ICC2	0.893	16.88	19	19	3.87E-08	0.781	0.949	v5.3 vs. v7.1	OASIS
SUM_rostralanteriorcingulate_surfavg	ICC3	0.957	45.69	19	19	5.57E-12	0.909	0.980	v6.0	OASIS
SUM_rostralanteriorcingulate_surfavg	ICC2	0.893	43.20	19	19	9.27E-12	0.371	0.965	v6.0 vs. v7.1	OASIS

SUM_rostralanteriorcingulate_surfavg	ICC3	0.884	16.23	19	19	5.39E-08	0.764	0.945	v7.1	OASIS
SUM_rostralmiddlefrontal_surfavg	ICC3	0.980	99.75	19	19	4.09E-15	0.957	0.991	v5.3	OASIS
SUM_rostralmiddlefrontal_surfavg	ICC2	0.962	52.41	19	19	1.58E-12	0.920	0.982	v5.3 vs. v6.0	OASIS
SUM_rostralmiddlefrontal_surfavg	ICC2	0.919	36.65	19	19	4.13E-11	0.729	0.968	v5.3 vs. v7.1	OASIS
SUM_rostralmiddlefrontal_surfavg	ICC3	0.986	146.13	19	19	1.15E-16	0.971	0.994	v6.0	OASIS
SUM_rostralmiddlefrontal_surfavg	ICC2	0.972	173.84	19	19	2.24E-17	0.743	0.991	v6.0 vs. v7.1	OASIS
SUM_rostralmiddlefrontal_surfavg	ICC3	0.970	65.04	19	19	2.17E-13	0.935	0.986	v7.1	OASIS
SUM_superiorfrontal_surfavg	ICC3	0.985	135.45	19	19	2.34E-16	0.968	0.993	v5.3	OASIS
SUM_superiorfrontal_surfavg	ICC2	0.933	83.68	19	19	2.10E-14	0.415	0.980	v5.3 vs. v6.0	OASIS
SUM_superiorfrontal_surfavg	ICC2	0.868	68.25	19	19	1.39E-13	0.063	0.962	v5.3 vs. v7.1	OASIS
SUM_superiorfrontal_surfavg	ICC3	0.988	172.16	19	19	2.46E-17	0.975	0.995	v6.0	OASIS
SUM_superiorfrontal_surfavg	ICC2	0.973	187.59	19	19	1.10E-17	0.731	0.992	v6.0 vs. v7.1	OASIS
SUM_superiorfrontal_surfavg	ICC3	0.963	53.18	19	19	1.39E-12	0.922	0.983	v7.1	OASIS
SUM_superiorparietal_surfavg	ICC3	0.936	30.43	19	19	2.20E-10	0.867	0.970	v5.3	OASIS
SUM_superiorparietal_surfavg	ICC2	0.956	42.96	19	19	9.76E-12	0.908	0.980	v5.3 vs. v6.0	OASIS
SUM_superiorparietal_surfavg	ICC2	0.955	44.75	19	19	6.73E-12	0.906	0.979	v5.3 vs. v7.1	OASIS
SUM_superiorparietal_surfavg	ICC3	0.996	509.35	19	19	8.79E-22	0.992	0.998	v6.0	OASIS
SUM_superiorparietal_surfavg	ICC2	0.991	506.40	19	19	9.29E-22	0.928	0.997	v6.0 vs. v7.1	OASIS
SUM_superiorparietal_surfavg	ICC3	0.897	18.46	19	19	1.79E-08	0.790	0.951	v7.1	OASIS
SUM_superiortemporal_surfavg	ICC3	0.991	229.36	19	19	1.65E-18	0.981	0.996	v5.3	OASIS
SUM_superiortemporal_surfavg	ICC2	0.925	96.22	19	19	5.72E-15	0.253	0.978	v5.3 vs. v6.0	OASIS
SUM_superiortemporal_surfavg	ICC2	0.871	58.60	19	19	5.68E-13	0.100	0.962	v5.3 vs. v7.1	OASIS
SUM_superiortemporal_surfavg	ICC3	0.995	417.37	19	19	5.79E-21	0.990	0.998	v6.0	OASIS
SUM_superiortemporal_surfavg	ICC2	0.988	401.17	19	19	8.42E-21	0.870	0.996	v6.0 vs. v7.1	OASIS
SUM_superiortemporal_surfavg	ICC3	0.992	241.25	19	19	1.03E-18	0.982	0.996	v7.1	OASIS
SUM_supramarginal_surfavg	ICC3	0.985	132.96	19	19	2.78E-16	0.968	0.993	v5.3	OASIS
SUM_supramarginal_surfavg	ICC2	0.909	25.68	19	19	1.00E-09	0.787	0.959	v5.3 vs. v6.0	OASIS
SUM_supramarginal_surfavg	ICC2	0.872	22.71	19	19	2.96E-09	0.603	0.949	v5.3 vs. v7.1	OASIS
SUM_supramarginal_surfavg	ICC3	0.994	333.34	19	19	4.85E-20	0.987	0.997	v6.0	OASIS
SUM_supramarginal_surfavg	ICC2	0.990	614.47	19	19	1.49E-22	0.850	0.997	v6.0 vs. v7.1	OASIS
SUM_supramarginal_surfavg	ICC3	0.984	120.25	19	19	7.12E-16	0.965	0.992	v7.1	OASIS
SUM_SurfArea	ICC3	0.994	359.49	19	19	2.37E-20	0.988	0.997	v5.3	OASIS
SUM_SurfArea	ICC2	0.979	713.56	19	19	3.61E-23	0.356	0.995	v5.3 vs. v6.0	OASIS
SUM_SurfArea	ICC2	0.919	462.33	19	19	2.20E-21	0.049	0.980	v5.3 vs. v7.1	OASIS
SUM_SurfArea	ICC3	0.996	499.98	19	19	1.05E-21	0.991	0.998	v6.0	OASIS
SUM_SurfArea	ICC2	0.976	904.07	19	19	3.83E-24	0.251	0.994	v6.0 vs. v7.1	OASIS
SUM_SurfArea	ICC3	0.995	374.70	19	19	1.61E-20	0.988	0.998	v7.1	OASIS
SUM_temporalpole_surfavg	ICC3	0.807	9.35	19	19	4.97E-06	0.624	0.906	v5.3	OASIS
SUM_temporalpole_surfavg	ICC2	0.701	7.78	19	19	2.06E-05	0.354	0.861	v5.3 vs. v6.0	OASIS
SUM_temporalpole_surfavg	ICC2	0.316	4.08	19	19	1.79E-03	-0.073	0.631	v5.3 vs. v7.1	OASIS
SUM_temporalpole_surfavg	ICC3	0.840	11.53	19	19	9.33E-07	0.683	0.923	v6.0	OASIS
SUM_temporalpole_surfavg	ICC2	0.617	10.61	19	19	1.82E-06	-0.010	0.850	v6.0 vs. v7.1	OASIS
SUM_temporalpole_surfavg	ICC3	0.866	13.96	19	19	1.93E-07	0.731	0.936	v7.1	OASIS
SUM_transversetemporal_surfavg	ICC3	0.952	40.94	19	19	1.51E-11	0.899	0.978	v5.3	OASIS

SUM_transversetemporal_surfavg	ICC2	0.789	8.52	19	19	1.03E-05	0.597	0.896	v5.3 vs. v6.0	OASIS
SUM_transversetemporal_surfavg	ICC2	0.749	6.72	19	19	6.12E-05	0.524	0.876	v5.3 vs. v7.1	OASIS
SUM_transversetemporal_surfavg	ICC3	0.957	45.19	19	19	6.16E-12	0.908	0.980	v6.0	OASIS
SUM_transversetemporal_surfavg	ICC2	0.966	62.44	19	19	3.16E-13	0.927	0.984	v6.0 vs. v7.1	OASIS
SUM_transversetemporal_surfavg	ICC3	0.976	83.84	19	19	2.06E-14	0.950	0.989	v7.1	OASIS

Table S6. HNU specific ICCs and associated statistics.

ROI	Type	ICC	F	df1	df2	p	lower bound	upper bound	comparison	dataset
AVG_Accumbens	ICC3	0.703	24.63	26	234	6.66E-53	0.5994	0.8054	v5.3	HNU
AVG_Accumbens	ICC2	0.547	7.63	26	26	9.03E-07	-0.0148	0.7971	v5.3 vs. v6.0	HNU
AVG_Accumbens	ICC2	0.643	9.49	26	26	9.20E-08	0.0715	0.8469	v5.3 vs. v7.1	HNU
AVG_Accumbens	ICC3	0.851	58.01	26	234	4.62E-87	0.7854	0.9081	v6.0	HNU
AVG_Accumbens	ICC2	0.905	21.08	26	26	1.05E-11	0.8247	0.9497	v6.0 vs. v7.1	HNU
AVG_Accumbens	ICC3	0.837	52.29	26	234	1.33E-82	0.7668	0.8990	v7.1	HNU
AVG_Amygdala	ICC3	0.792	39.08	26	234	1.60E-70	0.7087	0.8689	v5.3	HNU
AVG_Amygdala	ICC2	0.761	8.39	26	26	3.40E-07	0.5677	0.8712	v5.3 vs. v6.0	HNU
AVG_Amygdala	ICC2	0.752	7.89	26	26	6.43E-07	0.5600	0.8650	v5.3 vs. v7.1	HNU
AVG_Amygdala	ICC3	0.850	57.88	26	234	5.73E-87	0.7850	0.9079	v6.0	HNU
AVG_Amygdala	ICC2	0.894	17.43	26	26	9.88E-11	0.8061	0.9439	v6.0 vs. v7.1	HNU
AVG_Amygdala	ICC3	0.824	47.92	26	234	6.40E-79	0.7504	0.8907	v7.1	HNU
AVG_bankssts_thickavg	ICC3	0.695	23.82	26	234	1.06E-51	0.5908	0.8000	v5.3	HNU
AVG_bankssts_thickavg	ICC2	0.745	9.95	26	26	5.52E-08	0.4115	0.8789	v5.3 vs. v6.0	HNU
AVG_bankssts_thickavg	ICC2	0.776	8.06	26	26	5.14E-07	0.6117	0.8767	v5.3 vs. v7.1	HNU
AVG_bankssts_thickavg	ICC3	0.794	39.50	26	234	5.92E-71	0.7110	0.8701	v6.0	HNU
AVG_bankssts_thickavg	ICC2	0.870	22.29	26	26	5.38E-12	0.6184	0.9438	v6.0 vs. v7.1	HNU
AVG_bankssts_thickavg	ICC3	0.783	37.03	26	234	2.33E-68	0.6969	0.8625	v7.1	HNU
AVG_caudalanteriorcingulate_thickavg	ICC3	0.769	34.32	26	234	2.35E-65	0.6799	0.8531	v5.3	HNU
AVG_caudalanteriorcingulate_thickavg	ICC2	0.771	16.40	26	26	2.01E-10	0.2069	0.9104	v5.3 vs. v6.0	HNU
AVG_caudalanteriorcingulate_thickavg	ICC2	0.888	19.49	26	26	2.66E-11	0.7792	0.9425	v5.3 vs. v7.1	HNU
AVG_caudalanteriorcingulate_thickavg	ICC3	0.835	51.72	26	234	3.88E-82	0.7648	0.8980	v6.0	HNU
AVG_caudalanteriorcingulate_thickavg	ICC2	0.713	36.06	26	26	1.54E-14	-0.0245	0.9054	v6.0 vs. v7.1	HNU
AVG_caudalanteriorcingulate_thickavg	ICC3	0.861	62.78	26	234	1.66E-90	0.7987	0.9146	v7.1	HNU
AVG_caudalmiddlefrontal_thickavg	ICC3	0.859	61.98	26	234	6.01E-90	0.7966	0.9136	v5.3	HNU
AVG_caudalmiddlefrontal_thickavg	ICC2	0.657	32.28	26	26	6.03E-14	-0.0303	0.8822	v5.3 vs. v6.0	HNU
AVG_caudalmiddlefrontal_thickavg	ICC2	0.741	22.49	26	26	4.85E-12	0.0141	0.9090	v5.3 vs. v7.1	HNU
AVG_caudalmiddlefrontal_thickavg	ICC3	0.906	97.68	26	234	2.10E-110	0.8617	0.9435	v6.0	HNU
AVG_caudalmiddlefrontal_thickavg	ICC2	0.892	26.20	26	26	7.69E-13	0.6871	0.9527	v6.0 vs. v7.1	HNU
AVG_caudalmiddlefrontal_thickavg	ICC3	0.879	73.99	26	234	8.87E-98	0.8244	0.9266	v7.1	HNU
AVG_Caudate	ICC3	0.969	315.32	26	234	1.37E-166	0.9531	0.9819	v5.3	HNU
AVG_Caudate	ICC2	0.863	85.48	26	26	3.02E-19	0.0343	0.9600	v5.3 vs. v6.0	HNU
AVG_Caudate	ICC2	0.898	69.67	26	26	4.05E-18	0.1842	0.9688	v5.3 vs. v7.1	HNU
AVG_Caudate	ICC3	0.979	456.21	26	234	7.05E-185	0.9672	0.9874	v6.0	HNU
AVG_Caudate	ICC2	0.983	200.04	26	26	5.61E-24	0.9266	0.9934	v6.0 vs. v7.1	HNU
AVG_Caudate	ICC3	0.984	618.27	26	234	4.87E-200	0.9756	0.9907	v7.1	HNU
AVG_cuneus_thickavg	ICC3	0.801	41.13	26	234	1.36E-72	0.7195	0.8747	v5.3	HNU
AVG_cuneus_thickavg	ICC2	0.687	13.63	26	26	1.69E-09	0.0384	0.8780	v5.3 vs. v6.0	HNU
AVG_cuneus_thickavg	ICC2	0.789	13.20	26	26	2.43E-09	0.4456	0.9056	v5.3 vs. v7.1	HNU
AVG_cuneus_thickavg	ICC3	0.828	49.19	26	234	5.17E-80	0.7554	0.8932	v6.0	HNU
AVG_cuneus_thickavg	ICC2	0.875	20.04	26	26	1.91E-11	0.7039	0.9408	v6.0 vs. v7.1	HNU

AVG_cuneus_thickavg	ICC3	0.826	48.60	26	234	1.66E-79	0.7530	0.8921	v7.1	HNU
AVG_entorhinal_thickavg	ICC3	0.627	17.81	26	234	6.90E-42	0.5132	0.7478	v5.3	HNU
AVG_entorhinal_thickavg	ICC2	0.807	10.14	26	26	4.50E-08	0.6545	0.8957	v5.3 vs. v6.0	HNU
AVG_entorhinal_thickavg	ICC2	0.586	7.67	26	26	8.57E-07	0.0373	0.8134	v5.3 vs. v7.1	HNU
AVG_entorhinal_thickavg	ICC3	0.609	16.57	26	234	1.35E-39	0.4933	0.7334	v6.0	HNU
AVG_entorhinal_thickavg	ICC2	0.468	9.01	26	26	1.61E-07	-0.0612	0.7626	v6.0 vs. v7.1	HNU
AVG_entorhinal_thickavg	ICC3	0.629	17.92	26	234	4.38E-42	0.5149	0.7490	v7.1	HNU
AVG_frontalpole_thickavg	ICC3	0.781	36.66	26	234	5.79E-68	0.6947	0.8613	v5.3	HNU
AVG_frontalpole_thickavg	ICC2	0.696	5.46	26	26	2.39E-05	0.4855	0.8297	v5.3 vs. v6.0	HNU
AVG_frontalpole_thickavg	ICC2	0.732	6.50	26	26	4.54E-06	0.5440	0.8511	v5.3 vs. v7.1	HNU
AVG_frontalpole_thickavg	ICC3	0.830	49.91	26	234	1.25E-80	0.7581	0.8946	v6.0	HNU
AVG_frontalpole_thickavg	ICC2	0.882	15.54	26	26	3.75E-10	0.7842	0.9367	v6.0 vs. v7.1	HNU
AVG_frontalpole_thickavg	ICC3	0.839	53.08	26	234	3.00E-83	0.7696	0.9004	v7.1	HNU
AVG_fusiform_thickavg	ICC3	0.604	16.25	26	234	5.29E-39	0.4880	0.7295	v5.3	HNU
AVG_fusiform_thickavg	ICC2	0.672	8.63	26	26	2.52E-07	0.2045	0.8504	v5.3 vs. v6.0	HNU
AVG_fusiform_thickavg	ICC2	0.775	7.66	26	26	8.75E-07	0.6063	0.8771	v5.3 vs. v7.1	HNU
AVG_fusiform_thickavg	ICC3	0.726	27.56	26	234	5.47E-57	0.6277	0.8228	v6.0	HNU
AVG_fusiform_thickavg	ICC2	0.745	19.51	26	26	2.63E-11	0.0505	0.9074	v6.0 vs. v7.1	HNU
AVG_fusiform_thickavg	ICC3	0.750	31.02	26	234	1.91E-61	0.6564	0.8397	v7.1	HNU
AVG_Hippocampus	ICC3	0.767	33.88	26	234	7.50E-65	0.6770	0.8514	v5.3	HNU
AVG_Hippocampus	ICC2	0.814	13.01	26	26	2.86E-09	0.5877	0.9098	v5.3 vs. v6.0	HNU
AVG_Hippocampus	ICC2	0.748	18.40	26	26	5.24E-11	0.0754	0.9070	v5.3 vs. v7.1	HNU
AVG_Hippocampus	ICC3	0.899	90.13	26	234	1.04E-106	0.8516	0.9391	v6.0	HNU
AVG_Hippocampus	ICC2	0.896	34.85	26	26	2.35E-14	0.5589	0.9604	v6.0 vs. v7.1	HNU
AVG_Hippocampus	ICC3	0.920	116.33	26	234	1.61E-118	0.8815	0.9522	v7.1	HNU
AVG_inferiorparietal_thickavg	ICC3	0.812	44.13	26	234	1.73E-75	0.7340	0.8823	v5.3	HNU
AVG_inferiorparietal_thickavg	ICC2	0.676	16.30	26	26	2.16E-10	-0.0117	0.8792	v5.3 vs. v6.0	HNU
AVG_inferiorparietal_thickavg	ICC2	0.816	12.10	26	26	6.42E-09	0.6309	0.9063	v5.3 vs. v7.1	HNU
AVG_inferiorparietal_thickavg	ICC3	0.836	52.04	26	234	2.12E-82	0.7659	0.8985	v6.0	HNU
AVG_inferiorparietal_thickavg	ICC2	0.789	20.14	26	26	1.81E-11	0.1729	0.9216	v6.0 vs. v7.1	HNU
AVG_inferiorparietal_thickavg	ICC3	0.809	43.36	26	234	9.19E-75	0.7305	0.8804	v7.1	HNU
AVG_inferiortemporal_thickavg	ICC3	0.629	17.98	26	234	3.47E-42	0.5158	0.7496	v5.3	HNU
AVG_inferiortemporal_thickavg	ICC2	0.679	8.02	26	26	5.45E-07	0.2777	0.8470	v5.3 vs. v6.0	HNU
AVG_inferiortemporal_thickavg	ICC2	0.753	7.53	26	26	1.04E-06	0.5721	0.8635	v5.3 vs. v7.1	HNU
AVG_inferiortemporal_thickavg	ICC3	0.729	27.91	26	234	1.84E-57	0.6308	0.8247	v6.0	HNU
AVG_inferiortemporal_thickavg	ICC2	0.654	17.63	26	26	8.65E-11	-0.0325	0.8722	v6.0 vs. v7.1	HNU
AVG_inferiortemporal_thickavg	ICC3	0.698	24.06	26	234	4.58E-52	0.5934	0.8016	v7.1	HNU
AVG_insula_thickavg	ICC3	0.596	15.76	26	234	4.55E-38	0.4796	0.7232	v5.3	HNU
AVG_insula_thickavg	ICC2	0.662	4.79	26	26	7.94E-05	0.4344	0.8100	v5.3 vs. v6.0	HNU
AVG_insula_thickavg	ICC2	0.766	8.69	26	26	2.33E-07	0.5707	0.8746	v5.3 vs. v7.1	HNU
AVG_insula_thickavg	ICC3	0.556	13.50	26	234	1.68E-33	0.4366	0.6898	v6.0	HNU
AVG_insula_thickavg	ICC2	0.698	6.58	26	26	4.02E-06	0.4639	0.8358	v6.0 vs. v7.1	HNU
AVG_insula_thickavg	ICC3	0.703	24.63	26	234	6.81E-53	0.5994	0.8054	v7.1	HNU
AVG_isthmuscingulate_thickavg	ICC3	0.846	55.79	26	234	2.23E-85	0.7785	0.9048	v5.3	HNU

AVG_isthmuscingulate_thickavg	ICC2	0.733	7.72	26	26	8.08E-07	0.5098	0.8577	v5.3 vs. v6.0	HNU
AVG_isthmuscingulate_thickavg	ICC2	0.521	6.17	26	26	7.52E-06	0.0063	0.7717	v5.3 vs. v7.1	HNU
AVG_isthmuscingulate_thickavg	ICC3	0.900	90.81	26	234	4.69E-107	0.8526	0.9395	v6.0	HNU
AVG_isthmuscingulate_thickavg	ICC2	0.816	26.40	26	26	7.00E-13	0.1576	0.9352	v6.0 vs. v7.1	HNU
AVG_isthmuscingulate_thickavg	ICC3	0.894	85.19	26	234	3.84E-104	0.8443	0.9357	v7.1	HNU
AVG_lateraloccipital_thickavg	ICC3	0.790	38.54	26	234	5.76E-70	0.7057	0.8673	v5.3	HNU
AVG_lateraloccipital_thickavg	ICC2	0.559	10.18	26	26	4.34E-08	-0.0439	0.8157	v5.3 vs. v6.0	HNU
AVG_lateraloccipital_thickavg	ICC2	0.731	9.63	26	26	7.86E-08	0.3698	0.8734	v5.3 vs. v7.1	HNU
AVG_lateraloccipital_thickavg	ICC3	0.863	63.90	26	234	2.77E-91	0.8016	0.9160	v6.0	HNU
AVG_lateraloccipital_thickavg	ICC2	0.827	22.74	26	26	4.25E-12	0.2973	0.9349	v6.0 vs. v7.1	HNU
AVG_lateraloccipital_thickavg	ICC3	0.867	66.24	26	234	7.24E-93	0.8074	0.9187	v7.1	HNU
AVG_lateralorbitofrontal_thickavg	ICC3	0.584	15.02	26	234	1.28E-36	0.4663	0.7131	v5.3	HNU
AVG_lateralorbitofrontal_thickavg	ICC2	0.608	10.29	26	26	3.84E-08	-0.0096	0.8373	v5.3 vs. v6.0	HNU
AVG_lateralorbitofrontal_thickavg	ICC2	0.795	9.37	26	26	1.06E-07	0.6378	0.8885	v5.3 vs. v7.1	HNU
AVG_lateralorbitofrontal_thickavg	ICC3	0.689	23.11	26	234	1.24E-50	0.5829	0.7950	v6.0	HNU
AVG_lateralorbitofrontal_thickavg	ICC2	0.661	11.18	26	26	1.55E-08	0.0493	0.8611	v6.0 vs. v7.1	HNU
AVG_lateralorbitofrontal_thickavg	ICC3	0.562	13.84	26	234	3.34E-34	0.4434	0.6952	v7.1	HNU
AVG_LateralVentricle	ICC3	0.997	3854.51	26	234	2.37E-292	0.9960	0.9985	v5.3	HNU
AVG_LateralVentricle	ICC2	0.992	2566.73	26	26	2.45E-38	0.5259	0.9979	v5.3 vs. v6.0	HNU
AVG_LateralVentricle	ICC2	0.991	3348.68	26	26	7.75E-40	0.4264	0.9977	v5.3 vs. v7.1	HNU
AVG_LateralVentricle	ICC3	0.998	5114.53	26	234	1.08E-306	0.9970	0.9989	v6.0	HNU
AVG_LateralVentricle	ICC2	1.000	1.34E+04	26	26	1.21E-47	0.9995	0.9999	v6.0 vs. v7.1	HNU
AVG_LateralVentricle	ICC3	0.998	5209.19	26	234	1.27E-307	0.9970	0.9989	v7.1	HNU
AVG_lingual_thickavg	ICC3	0.789	38.46	26	234	7.12E-70	0.7052	0.8670	v5.3	HNU
AVG_lingual_thickavg	ICC2	0.697	12.47	26	26	4.61E-09	0.0894	0.8778	v5.3 vs. v6.0	HNU
AVG_lingual_thickavg	ICC2	0.864	13.22	26	26	2.38E-09	0.7524	0.9271	v5.3 vs. v7.1	HNU
AVG_lingual_thickavg	ICC3	0.824	47.96	26	234	5.93E-79	0.7505	0.8908	v6.0	HNU
AVG_lingual_thickavg	ICC2	0.740	19.32	26	26	2.94E-11	0.0437	0.9056	v6.0 vs. v7.1	HNU
AVG_lingual_thickavg	ICC3	0.851	58.12	26	234	3.79E-87	0.7857	0.9083	v7.1	HNU
AVG_medialorbitofrontal_thickavg	ICC3	0.549	13.19	26	234	7.83E-33	0.4300	0.6845	v5.3	HNU
AVG_medialorbitofrontal_thickavg	ICC2	0.552	7.45	26	26	1.16E-06	-0.0043	0.7976	v5.3 vs. v6.0	HNU
AVG_medialorbitofrontal_thickavg	ICC2	0.683	5.24	26	26	3.50E-05	0.4684	0.8220	v5.3 vs. v7.1	HNU
AVG_medialorbitofrontal_thickavg	ICC3	0.642	18.97	26	234	6.40E-44	0.5303	0.7599	v6.0	HNU
AVG_medialorbitofrontal_thickavg	ICC2	0.572	7.85	26	26	6.77E-07	0.0089	0.8094	v6.0 vs. v7.1	HNU
AVG_medialorbitofrontal_thickavg	ICC3	0.589	15.31	26	234	3.52E-37	0.4715	0.7171	v7.1	HNU
AVG_middletemporal_thickavg	ICC3	0.772	34.83	26	234	6.17E-66	0.6833	0.8550	v5.3	HNU
AVG_middletemporal_thickavg	ICC2	0.832	17.49	26	26	9.49E-11	0.5070	0.9274	v5.3 vs. v6.0	HNU
AVG_middletemporal_thickavg	ICC2	0.856	15.45	26	26	4.00E-10	0.7109	0.9267	v5.3 vs. v7.1	HNU
AVG_middletemporal_thickavg	ICC3	0.885	78.26	26	234	2.66E-100	0.8325	0.9304	v6.0	HNU
AVG_middletemporal_thickavg	ICC2	0.721	24.79	26	26	1.50E-12	-0.0147	0.9039	v6.0 vs. v7.1	HNU
AVG_middletemporal_thickavg	ICC3	0.859	61.68	26	234	9.89E-90	0.7958	0.9132	v7.1	HNU
AVG_Pallidum	ICC3	0.855	59.78	26	234	2.29E-88	0.7905	0.9106	v5.3	HNU
AVG_Pallidum	ICC2	0.603	4.95	26	26	5.86E-05	0.3115	0.7809	v5.3 vs. v6.0	HNU
AVG_Pallidum	ICC2	0.460	3.81	26	26	5.45E-04	0.0844	0.6988	v5.3 vs. v7.1	HNU

AVG_Pallidum	ICC3	0.903	94.41	26	234	7.74E-109	0.8575	0.9417	v6.0	HNU
AVG_Pallidum	ICC2	0.915	54.24	26	26	9.51E-17	0.4743	0.9710	v6.0 vs. v7.1	HNU
AVG_Pallidum	ICC3	0.889	81.01	26	234	7.35E-102	0.8374	0.9326	v7.1	HNU
AVG_paracentral_thickavg	ICC3	0.790	38.66	26	234	4.38E-70	0.7063	0.8676	v5.3	HNU
AVG_paracentral_thickavg	ICC2	0.522	21.81	26	26	6.97E-12	-0.0358	0.8122	v5.3 vs. v6.0	HNU
AVG_paracentral_thickavg	ICC2	0.534	17.45	26	26	9.73E-11	-0.0445	0.8158	v5.3 vs. v7.1	HNU
AVG_paracentral_thickavg	ICC3	0.797	40.18	26	234	1.20E-71	0.7146	0.8721	v6.0	HNU
AVG_paracentral_thickavg	ICC2	0.866	13.44	26	26	1.97E-09	0.7557	0.9280	v6.0 vs. v7.1	HNU
AVG_paracentral_thickavg	ICC3	0.793	39.35	26	234	8.44E-71	0.7102	0.8697	v7.1	HNU
AVG_parahippocampal_thickavg	ICC3	0.883	76.59	26	234	2.50E-99	0.8294	0.9290	v5.3	HNU
AVG_parahippocampal_thickavg	ICC2	0.876	50.75	26	26	2.19E-16	0.1711	0.9604	v5.3 vs. v6.0	HNU
AVG_parahippocampal_thickavg	ICC2	0.930	31.42	26	26	8.39E-14	0.8615	0.9643	v5.3 vs. v7.1	HNU
AVG_parahippocampal_thickavg	ICC3	0.906	96.95	26	234	4.67E-110	0.8608	0.9431	v6.0	HNU
AVG_parahippocampal_thickavg	ICC2	0.790	43.36	26	26	1.57E-15	0.0019	0.9338	v6.0 vs. v7.1	HNU
AVG_parahippocampal_thickavg	ICC3	0.906	97.27	26	234	3.29E-110	0.8612	0.9433	v7.1	HNU
AVG_parsopercularis_thickavg	ICC3	0.820	46.70	26	234	7.69E-78	0.7453	0.8881	v5.3	HNU
AVG_parsopercularis_thickavg	ICC2	0.643	18.26	26	26	5.73E-11	-0.0374	0.8684	v5.3 vs. v6.0	HNU
AVG_parsopercularis_thickavg	ICC2	0.739	23.85	26	26	2.39E-12	0.0041	0.9096	v5.3 vs. v7.1	HNU
AVG_parsopercularis_thickavg	ICC3	0.880	74.19	26	234	6.69E-98	0.8248	0.9268	v6.0	HNU
AVG_parsopercularis_thickavg	ICC2	0.887	19.80	26	26	2.20E-11	0.7686	0.9426	v6.0 vs. v7.1	HNU
AVG_parsopercularis_thickavg	ICC3	0.842	54.12	26	234	4.45E-84	0.7731	0.9021	v7.1	HNU
AVG_parsorbitalis_thickavg	ICC3	0.834	51.29	26	234	8.75E-82	0.7632	0.8972	v5.3	HNU
AVG_parsorbitalis_thickavg	ICC2	0.628	12.95	26	26	3.00E-09	-0.0251	0.8537	v5.3 vs. v6.0	HNU
AVG_parsorbitalis_thickavg	ICC2	0.626	6.20	26	26	7.10E-06	0.2528	0.8101	v5.3 vs. v7.1	HNU
AVG_parsorbitalis_thickavg	ICC3	0.809	43.38	26	234	8.84E-75	0.7306	0.8805	v6.0	HNU
AVG_parsorbitalis_thickavg	ICC2	0.780	10.87	26	26	2.11E-08	0.5193	0.8922	v6.0 vs. v7.1	HNU
AVG_parsorbitalis_thickavg	ICC3	0.784	37.24	26	234	1.39E-68	0.6982	0.8632	v7.1	HNU
AVG_parstriangularis_thickavg	ICC3	0.832	50.41	26	234	4.74E-81	0.7600	0.8956	v5.3	HNU
AVG_parstriangularis_thickavg	ICC2	0.522	13.12	26	26	2.60E-09	-0.0529	0.8039	v5.3 vs. v6.0	HNU
AVG_parstriangularis_thickavg	ICC2	0.661	14.58	26	26	7.83E-10	-0.0113	0.8705	v5.3 vs. v7.1	HNU
AVG_parstriangularis_thickavg	ICC3	0.859	61.80	26	234	8.03E-90	0.7961	0.9133	v6.0	HNU
AVG_parstriangularis_thickavg	ICC2	0.865	20.20	26	26	1.74E-11	0.6364	0.9393	v6.0 vs. v7.1	HNU
AVG_parstriangularis_thickavg	ICC3	0.829	49.33	26	234	3.90E-80	0.7559	0.8935	v7.1	HNU
AVG_pericalcarine_thickavg	ICC3	0.775	35.54	26	234	9.92E-67	0.6878	0.8575	v5.3	HNU
AVG_pericalcarine_thickavg	ICC2	0.693	13.05	26	26	2.77E-09	0.0641	0.8784	v5.3 vs. v6.0	HNU
AVG_pericalcarine_thickavg	ICC2	0.733	8.73	26	26	2.25E-07	0.4426	0.8667	v5.3 vs. v7.1	HNU
AVG_pericalcarine_thickavg	ICC3	0.826	48.44	26	234	2.28E-79	0.7524	0.8917	v6.0	HNU
AVG_pericalcarine_thickavg	ICC2	0.878	20.51	26	26	1.45E-11	0.7107	0.9422	v6.0 vs. v7.1	HNU
AVG_pericalcarine_thickavg	ICC3	0.872	69.33	26	234	7.01E-95	0.8146	0.9221	v7.1	HNU
AVG_postcentral_thickavg	ICC3	0.889	80.76	26	234	1.01E-101	0.8370	0.9324	v5.3	HNU
AVG_postcentral_thickavg	ICC2	0.508	27.17	26	26	4.95E-13	-0.0275	0.8059	v5.3 vs. v6.0	HNU
AVG_postcentral_thickavg	ICC2	0.510	40.86	26	26	3.27E-15	-0.0175	0.8100	v5.3 vs. v7.1	HNU
AVG_postcentral_thickavg	ICC3	0.898	89.03	26	234	3.78E-106	0.8501	0.9384	v6.0	HNU
AVG_postcentral_thickavg	ICC2	0.955	42.65	26	26	1.92E-15	0.9153	0.9763	v6.0 vs. v7.1	HNU

AVG_postcentral_thickavg	ICC3	0.862	63.54	26	234	4.93E-91	0.8007	0.9155	v7.1	HNU
AVG_posteriorcingulate_thickavg	ICC3	0.748	30.74	26	234	4.22E-61	0.6543	0.8385	v5.3	HNU
AVG_posteriorcingulate_thickavg	ICC2	0.791	9.64	26	26	7.80E-08	0.6212	0.8881	v5.3 vs. v6.0	HNU
AVG_posteriorcingulate_thickavg	ICC2	0.619	8.01	26	26	5.51E-07	0.0887	0.8284	v5.3 vs. v7.1	HNU
AVG_posteriorcingulate_thickavg	ICC3	0.816	45.39	26	234	1.20E-76	0.7397	0.8852	v6.0	HNU
AVG_posteriorcingulate_thickavg	ICC2	0.528	12.32	26	26	5.25E-09	-0.0542	0.8054	v6.0 vs. v7.1	HNU
AVG_posteriorcingulate_thickavg	ICC3	0.784	37.39	26	234	9.46E-69	0.6991	0.8637	v7.1	HNU
AVG_precentral_thickavg	ICC3	0.833	51.03	26	234	1.44E-81	0.7623	0.8967	v5.3	HNU
AVG_precentral_thickavg	ICC2	0.514	22.33	26	26	5.27E-12	-0.0342	0.8077	v5.3 vs. v6.0	HNU
AVG_precentral_thickavg	ICC2	0.455	16.35	26	26	2.09E-10	-0.0397	0.7675	v5.3 vs. v7.1	HNU
AVG_precentral_thickavg	ICC3	0.837	52.32	26	234	1.24E-82	0.7669	0.8990	v6.0	HNU
AVG_precentral_thickavg	ICC2	0.898	20.16	26	26	1.79E-11	0.8102	0.9464	v6.0 vs. v7.1	HNU
AVG_precentral_thickavg	ICC3	0.817	45.60	26	234	7.62E-77	0.7406	0.8857	v7.1	HNU
AVG_precuneus_thickavg	ICC3	0.817	45.51	26	234	9.21E-77	0.7402	0.8855	v5.3	HNU
AVG_precuneus_thickavg	ICC2	0.572	14.43	26	26	8.81E-10	-0.0492	0.8316	v5.3 vs. v6.0	HNU
AVG_precuneus_thickavg	ICC2	0.760	21.59	26	26	7.87E-12	0.0559	0.9145	v5.3 vs. v7.1	HNU
AVG_precuneus_thickavg	ICC3	0.856	60.27	26	234	1.01E-88	0.7919	0.9113	v6.0	HNU
AVG_precuneus_thickavg	ICC2	0.818	18.79	26	26	4.10E-11	0.3723	0.9272	v6.0 vs. v7.1	HNU
AVG_precuneus_thickavg	ICC3	0.850	57.65	26	234	8.52E-87	0.7843	0.9076	v7.1	HNU
AVG_Putamen	ICC3	0.937	149.90	26	234	1.71E-130	0.9058	0.9625	v5.3	HNU
AVG_Putamen	ICC2	0.329	28.26	26	26	3.06E-13	-0.0141	0.6699	v5.3 vs. v6.0	HNU
AVG_Putamen	ICC2	0.352	24.94	26	26	1.39E-12	-0.0179	0.6911	v5.3 vs. v7.1	HNU
AVG_Putamen	ICC3	0.944	170.04	26	234	1.59E-136	0.9161	0.9668	v6.0	HNU
AVG_Putamen	ICC2	0.979	156.40	26	26	1.33E-22	0.9187	0.9917	v6.0 vs. v7.1	HNU
AVG_Putamen	ICC3	0.979	462.60	26	234	1.43E-185	0.9676	0.9876	v7.1	HNU
AVG_rostralanteriorcingulate_thickavg	ICC3	0.527	12.13	26	234	1.59E-30	0.4068	0.6652	v5.3	HNU
AVG_rostralanteriorcingulate_thickavg	ICC2	0.698	9.36	26	26	1.07E-07	0.2490	0.8631	v5.3 vs. v6.0	HNU
AVG_rostralanteriorcingulate_thickavg	ICC2	0.734	7.42	26	26	1.20E-06	0.5253	0.8553	v5.3 vs. v7.1	HNU
AVG_rostralanteriorcingulate_thickavg	ICC3	0.647	19.33	26	234	1.53E-44	0.5354	0.7634	v6.0	HNU
AVG_rostralanteriorcingulate_thickavg	ICC2	0.679	63.00	26	26	1.44E-17	-0.0151	0.8955	v6.0 vs. v7.1	HNU
AVG_rostralanteriorcingulate_thickavg	ICC3	0.629	17.95	26	234	3.87E-42	0.5154	0.7494	v7.1	HNU
AVG_rostralmiddlefrontal_thickavg	ICC3	0.810	43.77	26	234	3.83E-75	0.7324	0.8814	v5.3	HNU
AVG_rostralmiddlefrontal_thickavg	ICC2	0.569	12.21	26	26	5.82E-09	-0.0487	0.8260	v5.3 vs. v6.0	HNU
AVG_rostralmiddlefrontal_thickavg	ICC2	0.606	8.67	26	26	2.39E-07	0.0314	0.8284	v5.3 vs. v7.1	HNU
AVG_rostralmiddlefrontal_thickavg	ICC3	0.832	50.41	26	234	4.70E-81	0.7600	0.8956	v6.0	HNU
AVG_rostralmiddlefrontal_thickavg	ICC2	0.827	13.09	26	26	2.66E-09	0.6429	0.9126	v6.0 vs. v7.1	HNU
AVG_rostralmiddlefrontal_thickavg	ICC3	0.793	39.26	26	234	1.04E-70	0.7097	0.8694	v7.1	HNU
AVG_superiorfrontal_thickavg	ICC3	0.866	65.38	26	234	2.73E-92	0.8053	0.9177	v5.3	HNU
AVG_superiorfrontal_thickavg	ICC2	0.673	16.68	26	26	1.65E-10	-0.0176	0.8783	v5.3 vs. v6.0	HNU
AVG_superiorfrontal_thickavg	ICC2	0.771	13.87	26	26	1.39E-09	0.3145	0.9038	v5.3 vs. v7.1	HNU
AVG_superiorfrontal_thickavg	ICC3	0.894	85.03	26	234	4.69E-104	0.8440	0.9356	v6.0	HNU
AVG_superiorfrontal_thickavg	ICC2	0.865	22.92	26	26	3.86E-12	0.5596	0.9436	v6.0 vs. v7.1	HNU
AVG_superiorfrontal_thickavg	ICC3	0.851	58.32	26	234	2.71E-87	0.7863	0.9086	v7.1	HNU
AVG_superiorparietal_thickavg	ICC3	0.862	63.24	26	234	7.99E-91	0.7999	0.9151	v5.3	HNU

AVG_superiorparietal_thickavg	ICC2	0.561	19.42	26	26	2.78E-11	-0.0424	0.8314	v5.3 vs. v6.0	HNU
AVG_superiorparietal_thickavg	ICC2	0.547	20.37	26	26	1.57E-11	-0.0400	0.8251	v5.3 vs. v7.1	HNU
AVG_superiorparietal_thickavg	ICC3	0.853	58.90	26	234	9.94E-88	0.7880	0.9094	v6.0	HNU
AVG_superiorparietal_thickavg	ICC2	0.906	19.68	26	26	2.37E-11	0.8269	0.9504	v6.0 vs. v7.1	HNU
AVG_superiorparietal_thickavg	ICC3	0.810	43.69	26	234	4.48E-75	0.7320	0.8812	v7.1	HNU
AVG_superiortemporal_thickavg	ICC3	0.813	44.38	26	234	1.02E-75	0.7352	0.8829	v5.3	HNU
AVG_superiortemporal_thickavg	ICC2	0.646	16.15	26	26	2.40E-10	-0.0320	0.8670	v5.3 vs. v6.0	HNU
AVG_superiortemporal_thickavg	ICC2	0.806	15.30	26	26	4.49E-10	0.4395	0.9164	v5.3 vs. v7.1	HNU
AVG_superiortemporal_thickavg	ICC3	0.852	58.59	26	234	1.68E-87	0.7871	0.9090	v6.0	HNU
AVG_superiortemporal_thickavg	ICC2	0.824	24.65	26	26	1.61E-12	0.2346	0.9362	v6.0 vs. v7.1	HNU
AVG_superiortemporal_thickavg	ICC3	0.818	45.80	26	234	5.02E-77	0.7415	0.8861	v7.1	HNU
AVG_supramarginal_thickavg	ICC3	0.795	39.69	26	234	3.78E-71	0.7120	0.8707	v5.3	HNU
AVG_supramarginal_thickavg	ICC2	0.676	16.42	26	26	1.98E-10	-0.0126	0.8794	v5.3 vs. v6.0	HNU
AVG_supramarginal_thickavg	ICC2	0.835	28.04	26	26	3.38E-13	0.2234	0.9417	v5.3 vs. v7.1	HNU
AVG_supramarginal_thickavg	ICC3	0.849	57.23	26	234	1.76E-86	0.7830	0.9070	v6.0	HNU
AVG_supramarginal_thickavg	ICC2	0.861	21.23	26	26	9.62E-12	0.5817	0.9405	v6.0 vs. v7.1	HNU
AVG_supramarginal_thickavg	ICC3	0.820	46.70	26	234	7.81E-78	0.7453	0.8881	v7.1	HNU
AVG_temporalpole_thickavg	ICC3	0.354	6.48	26	234	2.46E-16	0.2424	0.5037	v5.3	HNU
AVG_temporalpole_thickavg	ICC2	0.794	10.10	26	26	4.72E-08	0.6141	0.8910	v5.3 vs. v6.0	HNU
AVG_temporalpole_thickavg	ICC2	0.836	11.22	26	26	1.50E-08	0.7080	0.9111	v5.3 vs. v7.1	HNU
AVG_temporalpole_thickavg	ICC3	0.365	6.75	26	234	4.29E-17	0.2522	0.5149	v6.0	HNU
AVG_temporalpole_thickavg	ICC2	0.824	16.24	26	26	2.26E-10	0.5047	0.9226	v6.0 vs. v7.1	HNU
AVG_temporalpole_thickavg	ICC3	0.374	6.96	26	234	1.09E-17	0.2599	0.5234	v7.1	HNU
AVG_Thalamus	ICC3	0.869	67.61	26	234	9.00E-94	0.8107	0.9202	v5.3	HNU
AVG_Thalamus	ICC2	0.898	60.56	26	26	2.38E-17	0.2422	0.9678	v5.3 vs. v6.0	HNU
AVG_Thalamus	ICC2	0.733	25.05	26	26	1.32E-12	-0.0071	0.9081	v5.3 vs. v7.1	HNU
AVG_Thalamus	ICC3	0.938	151.60	26	234	4.94E-131	0.9068	0.9630	v6.0	HNU
AVG_Thalamus	ICC2	0.918	55.11	26	26	7.79E-17	0.4978	0.9718	v6.0 vs. v7.1	HNU
AVG_Thalamus	ICC3	0.922	119.40	26	234	9.71E-120	0.8842	0.9534	v7.1	HNU
AVG_Thickness	ICC3	0.816	45.28	26	234	1.48E-76	0.7392	0.8850	v5.3	HNU
AVG_Thickness	ICC2	0.494	12.63	26	26	3.99E-09	-0.0531	0.7873	v5.3 vs. v6.0	HNU
AVG_Thickness	ICC2	0.700	12.71	26	26	3.71E-09	0.0893	0.8795	v5.3 vs. v7.1	HNU
AVG_Thickness	ICC3	0.877	72.29	26	234	9.64E-97	0.8209	0.9250	v6.0	HNU
AVG_Thickness	ICC2	0.744	15.92	26	26	2.83E-10	0.1202	0.9015	v6.0 vs. v7.1	HNU
AVG_Thickness	ICC3	0.826	48.60	26	234	1.66E-79	0.7530	0.8921	v7.1	HNU
AVG_transversetemporal_thickavg	ICC3	0.868	66.72	26	234	3.47E-93	0.8086	0.9192	v5.3	HNU
AVG_transversetemporal_thickavg	ICC2	0.708	38.79	26	26	6.25E-15	-0.0243	0.9041	v5.3 vs. v6.0	HNU
AVG_transversetemporal_thickavg	ICC2	0.781	30.25	26	26	1.34E-13	0.0247	0.9268	v5.3 vs. v7.1	HNU
AVG_transversetemporal_thickavg	ICC3	0.878	72.70	26	234	5.35E-97	0.8218	0.9254	v6.0	HNU
AVG_transversetemporal_thickavg	ICC2	0.950	66.30	26	26	7.57E-18	0.8042	0.9803	v6.0 vs. v7.1	HNU
AVG_transversetemporal_thickavg	ICC3	0.866	65.82	26	234	1.39E-92	0.8064	0.9182	v7.1	HNU
ICV	ICC3	0.991	1059.85	26	234	4.34E-227	0.9856	0.9945	v5.3	HNU
ICV	ICC2	1.000	1.32E+07	26	26	1.39E-86	1.0000	1.0000	v5.3 vs. v6.0	HNU
ICV	ICC2	0.996	2739.14	26	26	1.05E-38	0.8083	0.9988	v5.3 vs. v7.1	HNU

ICV	ICC3	0.991	1061.10	26	234	3.78E-227	0.9856	0.9946	v6.0	HNU
ICV	ICC2	0.996	2737.88	26	26	1.06E-38	0.8074	0.9988	v6.0 vs. v7.1	HNU
ICV	ICC3	0.988	831.81	26	234	6.59E-215	0.9818	0.9931	v7.1	HNU
LH_Accumbens	ICC3	0.602	16.14	26	234	8.61E-39	0.4861	0.7281	v5.3	HNU
LH_Accumbens	ICC2	0.517	4.29	26	26	2.04E-04	0.1534	0.7355	v5.3 vs. v6.0	HNU
LH_Accumbens	ICC2	0.590	6.66	26	26	3.53E-06	0.1060	0.8049	v5.3 vs. v7.1	HNU
LH_Accumbens	ICC3	0.745	30.15	26	234	2.33E-60	0.6496	0.8358	v6.0	HNU
LH_Accumbens	ICC2	0.920	24.30	26	26	1.91E-12	0.8529	0.9577	v6.0 vs. v7.1	HNU
LH_Accumbens	ICC3	0.762	33.09	26	234	6.18E-64	0.6715	0.8484	v7.1	HNU
LH_Amygdala	ICC3	0.788	38.22	26	234	1.27E-69	0.7038	0.8663	v5.3	HNU
LH_Amygdala	ICC2	0.825	10.61	26	26	2.74E-08	0.6908	0.9051	v5.3 vs. v6.0	HNU
LH_Amygdala	ICC2	0.761	7.15	26	26	1.75E-06	0.5838	0.8686	v5.3 vs. v7.1	HNU
LH_Amygdala	ICC3	0.806	42.49	26	234	6.35E-74	0.7263	0.8782	v6.0	HNU
LH_Amygdala	ICC2	0.894	18.07	26	26	6.47E-11	0.8073	0.9436	v6.0 vs. v7.1	HNU
LH_Amygdala	ICC3	0.788	38.28	26	234	1.09E-69	0.7042	0.8665	v7.1	HNU
LH_bankssts_surfavg	ICC3	0.887	79.62	26	234	4.46E-101	0.8350	0.9315	v5.3	HNU
LH_bankssts_surfavg	ICC2	0.774	8.08	26	26	5.02E-07	0.6088	0.8757	v5.3 vs. v6.0	HNU
LH_bankssts_surfavg	ICC2	0.744	7.11	26	26	1.86E-06	0.5619	0.8583	v5.3 vs. v7.1	HNU
LH_bankssts_surfavg	ICC3	0.951	196.79	26	234	1.45E-143	0.9268	0.9712	v6.0	HNU
LH_bankssts_surfavg	ICC2	0.943	33.06	26	26	4.50E-14	0.8931	0.9700	v6.0 vs. v7.1	HNU
LH_bankssts_surfavg	ICC3	0.955	211.50	26	234	4.67E-147	0.9315	0.9732	v7.1	HNU
LH_bankssts_thickavg	ICC3	0.553	13.38	26	234	3.05E-33	0.4341	0.6877	v5.3	HNU
LH_bankssts_thickavg	ICC2	0.642	7.58	26	26	9.64E-07	0.1829	0.8322	v5.3 vs. v6.0	HNU
LH_bankssts_thickavg	ICC2	0.693	6.21	26	26	7.07E-06	0.4688	0.8301	v5.3 vs. v7.1	HNU
LH_bankssts_thickavg	ICC3	0.744	30.03	26	234	3.31E-60	0.6486	0.8352	v6.0	HNU
LH_bankssts_thickavg	ICC2	0.796	10.79	26	26	2.30E-08	0.5948	0.8951	v6.0 vs. v7.1	HNU
LH_bankssts_thickavg	ICC3	0.741	29.57	26	234	1.27E-59	0.6449	0.8330	v7.1	HNU
LH_caudalanteriorcingulate_surfavg	ICC3	0.976	414.70	26	234	3.87E-180	0.9640	0.9862	v5.3	HNU
LH_caudalanteriorcingulate_surfavg	ICC2	0.895	25.10	26	26	1.29E-12	0.7272	0.9519	v5.3 vs. v6.0	HNU
LH_caudalanteriorcingulate_surfavg	ICC2	0.922	25.21	26	26	1.23E-12	0.8548	0.9585	v5.3 vs. v7.1	HNU
LH_caudalanteriorcingulate_surfavg	ICC3	0.987	747.02	26	234	1.63E-209	0.9797	0.9923	v6.0	HNU
LH_caudalanteriorcingulate_surfavg	ICC2	0.971	109.94	26	26	1.22E-20	0.8949	0.9884	v6.0 vs. v7.1	HNU
LH_caudalanteriorcingulate_surfavg	ICC3	0.990	974.96	26	234	6.89E-223	0.9844	0.9941	v7.1	HNU
LH_caudalanteriorcingulate_thickavg	ICC3	0.612	16.76	26	234	5.96E-40	0.4964	0.7357	v5.3	HNU
LH_caudalanteriorcingulate_thickavg	ICC2	0.554	7.15	26	26	1.75E-06	0.0095	0.7961	v5.3 vs. v6.0	HNU
LH_caudalanteriorcingulate_thickavg	ICC2	0.799	9.01	26	26	1.60E-07	0.6475	0.8898	v5.3 vs. v7.1	HNU
LH_caudalanteriorcingulate_thickavg	ICC3	0.691	23.40	26	234	4.52E-51	0.5861	0.7970	v6.0	HNU
LH_caudalanteriorcingulate_thickavg	ICC2	0.570	10.84	26	26	2.17E-08	-0.0434	0.8228	v6.0 vs. v7.1	HNU
LH_caudalanteriorcingulate_thickavg	ICC3	0.746	30.30	26	234	1.49E-60	0.6508	0.8365	v7.1	HNU
LH_caudalmiddlefrontal_surfavg	ICC3	0.973	355.62	26	234	1.58E-172	0.9582	0.9839	v5.3	HNU
LH_caudalmiddlefrontal_surfavg	ICC2	0.730	7.50	26	26	1.07E-06	0.5108	0.8549	v5.3 vs. v6.0	HNU
LH_caudalmiddlefrontal_surfavg	ICC2	0.830	12.09	26	26	6.51E-09	0.6834	0.9098	v5.3 vs. v7.1	HNU
LH_caudalmiddlefrontal_surfavg	ICC3	0.971	336.56	26	234	8.32E-170	0.9560	0.9830	v6.0	HNU
LH_caudalmiddlefrontal_surfavg	ICC2	0.964	65.04	26	26	9.66E-18	0.9218	0.9822	v6.0 vs. v7.1	HNU

LH_caudalmiddlefrontal_surfavg	ICC3	0.984	624.22	26	234	1.62E-200	0.9758	0.9908	v7.1	HNU
LH_caudalmiddlefrontal_thickavg	ICC3	0.795	39.89	26	234	2.36E-71	0.7131	0.8712	v5.3	HNU
LH_caudalmiddlefrontal_thickavg	ICC2	0.639	22.08	26	26	6.03E-12	-0.0385	0.8697	v5.3 vs. v6.0	HNU
LH_caudalmiddlefrontal_thickavg	ICC2	0.743	16.39	26	26	2.02E-10	0.1040	0.9021	v5.3 vs. v7.1	HNU
LH_caudalmiddlefrontal_thickavg	ICC3	0.867	66.45	26	234	5.28E-93	0.8079	0.9189	v6.0	HNU
LH_caudalmiddlefrontal_thickavg	ICC2	0.888	23.53	26	26	2.82E-12	0.7151	0.9487	v6.0 vs. v7.1	HNU
LH_caudalmiddlefrontal_thickavg	ICC3	0.824	47.67	26	234	1.08E-78	0.7493	0.8902	v7.1	HNU
LH_Caudate	ICC3	0.967	292.06	26	234	8.04E-163	0.9496	0.9805	v5.3	HNU
LH_Caudate	ICC2	0.881	56.52	26	26	5.67E-17	0.1631	0.9629	v5.3 vs. v6.0	HNU
LH_Caudate	ICC2	0.889	38.49	26	26	6.87E-15	0.4181	0.9604	v5.3 vs. v7.1	HNU
LH_Caudate	ICC3	0.971	338.75	26	234	3.99E-170	0.9562	0.9831	v6.0	HNU
LH_Caudate	ICC2	0.976	95.05	26	26	7.82E-20	0.9519	0.9881	v6.0 vs. v7.1	HNU
LH_Caudate	ICC3	0.973	359.36	26	234	4.80E-173	0.9587	0.9841	v7.1	HNU
LH_cuneus_surfavg	ICC3	0.889	80.91	26	234	8.35E-102	0.8372	0.9325	v5.3	HNU
LH_cuneus_surfavg	ICC2	0.847	17.16	26	26	1.19E-10	0.6139	0.9295	v5.3 vs. v6.0	HNU
LH_cuneus_surfavg	ICC2	0.736	14.73	26	26	6.95E-10	0.1314	0.8966	v5.3 vs. v7.1	HNU
LH_cuneus_surfavg	ICC3	0.938	151.88	26	234	4.03E-131	0.9069	0.9630	v6.0	HNU
LH_cuneus_surfavg	ICC2	0.935	55.33	26	26	7.41E-17	0.7092	0.9754	v6.0 vs. v7.1	HNU
LH_cuneus_surfavg	ICC3	0.947	177.92	26	234	1.06E-138	0.9196	0.9683	v7.1	HNU
LH_cuneus_thickavg	ICC3	0.758	32.25	26	234	6.23E-63	0.6655	0.8450	v5.3	HNU
LH_cuneus_thickavg	ICC2	0.674	10.00	26	26	5.24E-08	0.1292	0.8602	v5.3 vs. v6.0	HNU
LH_cuneus_thickavg	ICC2	0.756	10.48	26	26	3.16E-08	0.4302	0.8848	v5.3 vs. v7.1	HNU
LH_cuneus_thickavg	ICC3	0.802	41.48	26	234	6.17E-73	0.7213	0.8756	v6.0	HNU
LH_cuneus_thickavg	ICC2	0.887	19.08	26	26	3.41E-11	0.7794	0.9416	v6.0 vs. v7.1	HNU
LH_cuneus_thickavg	ICC3	0.798	40.49	26	234	5.94E-72	0.7162	0.8729	v7.1	HNU
LH_entorhinal_surfavg	ICC3	0.647	19.35	26	234	1.38E-44	0.5358	0.7636	v5.3	HNU
LH_entorhinal_surfavg	ICC2	0.370	2.29	26	26	1.94E-02	0.0796	0.6084	v5.3 vs. v6.0	HNU
LH_entorhinal_surfavg	ICC2	0.485	2.85	26	26	4.81E-03	0.1980	0.6953	v5.3 vs. v7.1	HNU
LH_entorhinal_surfavg	ICC3	0.796	40.12	26	234	1.39E-71	0.7143	0.8719	v6.0	HNU
LH_entorhinal_surfavg	ICC2	0.840	13.28	26	26	2.26E-09	0.6936	0.9165	v6.0 vs. v7.1	HNU
LH_entorhinal_surfavg	ICC3	0.864	64.66	26	234	8.47E-92	0.8035	0.9169	v7.1	HNU
LH_entorhinal_thickavg	ICC3	0.515	11.63	26	234	2.11E-29	0.3952	0.6553	v5.3	HNU
LH_entorhinal_thickavg	ICC2	0.691	9.80	26	26	6.50E-08	0.1964	0.8643	v5.3 vs. v6.0	HNU
LH_entorhinal_thickavg	ICC2	0.670	6.55	26	26	4.17E-06	0.3703	0.8273	v5.3 vs. v7.1	HNU
LH_entorhinal_thickavg	ICC3	0.465	9.68	26	234	8.42E-25	0.3453	0.6103	v6.0	HNU
LH_entorhinal_thickavg	ICC2	0.408	6.83	26	26	2.76E-06	-0.0664	0.7137	v6.0 vs. v7.1	HNU
LH_entorhinal_thickavg	ICC3	0.429	8.52	26	234	7.12E-22	0.3115	0.5775	v7.1	HNU
LH_frontalpole_surfavg	ICC3	0.669	21.26	26	234	9.74E-48	0.5609	0.7806	v5.3	HNU
LH_frontalpole_surfavg	ICC2	0.272	4.26	26	26	2.19E-04	-0.0699	0.5786	v5.3 vs. v6.0	HNU
LH_frontalpole_surfavg	ICC2	0.187	5.02	26	26	5.14E-05	-0.0452	0.4787	v5.3 vs. v7.1	HNU
LH_frontalpole_surfavg	ICC3	0.746	30.34	26	234	1.34E-60	0.6511	0.8367	v6.0	HNU
LH_frontalpole_surfavg	ICC2	0.541	6.64	26	26	3.66E-06	0.0121	0.7855	v6.0 vs. v7.1	HNU
LH_frontalpole_surfavg	ICC3	0.781	36.69	26	234	5.46E-68	0.6949	0.8614	v7.1	HNU
LH_frontalpole_thickavg	ICC3	0.682	22.44	26	234	1.32E-49	0.5752	0.7900	v5.3	HNU

LH_frontalpole_thickavg	ICC2	0.601	4.00	26	26	3.67E-04	0.3534	0.7702	v5.3 vs. v6.0	HNU
LH_frontalpole_thickavg	ICC2	0.685	5.61	26	26	1.86E-05	0.4731	0.8224	v5.3 vs. v7.1	HNU
LH_frontalpole_thickavg	ICC3	0.754	31.61	26	234	3.63E-62	0.6608	0.8423	v6.0	HNU
LH_frontalpole_thickavg	ICC2	0.843	11.48	26	26	1.15E-08	0.7188	0.9156	v6.0 vs. v7.1	HNU
LH_frontalpole_thickavg	ICC3	0.743	29.85	26	234	5.58E-60	0.6472	0.8344	v7.1	HNU
LH_fusiform_surfavg	ICC3	0.943	167.19	26	234	1.03E-135	0.9148	0.9663	v5.3	HNU
LH_fusiform_surfavg	ICC2	0.779	11.82	26	26	8.33E-09	0.4659	0.8973	v5.3 vs. v6.0	HNU
LH_fusiform_surfavg	ICC2	0.781	10.38	26	26	3.49E-08	0.5512	0.8895	v5.3 vs. v7.1	HNU
LH_fusiform_surfavg	ICC3	0.978	452.61	26	234	1.74E-184	0.9669	0.9873	v6.0	HNU
LH_fusiform_surfavg	ICC2	0.979	102.53	26	26	2.97E-20	0.9603	0.9894	v6.0 vs. v7.1	HNU
LH_fusiform_surfavg	ICC3	0.975	392.30	26	234	2.19E-177	0.9620	0.9854	v7.1	HNU
LH_fusiform_thickavg	ICC3	0.585	15.08	26	234	9.98E-37	0.4673	0.7139	v5.3	HNU
LH_fusiform_thickavg	ICC2	0.700	6.69	26	26	3.40E-06	0.4610	0.8375	v5.3 vs. v6.0	HNU
LH_fusiform_thickavg	ICC2	0.799	9.12	26	26	1.41E-07	0.6480	0.8900	v5.3 vs. v7.1	HNU
LH_fusiform_thickavg	ICC3	0.623	17.56	26	234	1.99E-41	0.5093	0.7450	v6.0	HNU
LH_fusiform_thickavg	ICC2	0.709	9.30	26	26	1.15E-07	0.2994	0.8656	v6.0 vs. v7.1	HNU
LH_fusiform_thickavg	ICC3	0.695	23.81	26	234	1.10E-51	0.5907	0.7999	v7.1	HNU
LH_Hippocampus	ICC3	0.660	20.43	26	234	2.17E-46	0.5503	0.7735	v5.3	HNU
LH_Hippocampus	ICC2	0.769	10.80	26	26	2.27E-08	0.4720	0.8895	v5.3 vs. v6.0	HNU
LH_Hippocampus	ICC2	0.729	18.30	26	26	5.58E-11	0.0369	0.9008	v5.3 vs. v7.1	HNU
LH_Hippocampus	ICC3	0.864	64.69	26	234	7.99E-92	0.8036	0.9169	v6.0	HNU
LH_Hippocampus	ICC2	0.885	26.94	26	26	5.49E-13	0.6195	0.9523	v6.0 vs. v7.1	HNU
LH_Hippocampus	ICC3	0.901	91.71	26	234	1.67E-107	0.8539	0.9401	v7.1	HNU
LH_inferiorparietal_surfavg	ICC3	0.980	497.53	26	234	3.41E-189	0.9698	0.9884	v5.3	HNU
LH_inferiorparietal_surfavg	ICC2	0.909	21.65	26	26	7.61E-12	0.8329	0.9518	v5.3 vs. v6.0	HNU
LH_inferiorparietal_surfavg	ICC2	0.896	17.68	26	26	8.38E-11	0.8089	0.9448	v5.3 vs. v7.1	HNU
LH_inferiorparietal_surfavg	ICC3	0.987	755.48	26	234	4.45E-210	0.9799	0.9924	v6.0	HNU
LH_inferiorparietal_surfavg	ICC2	0.983	145.17	26	26	3.47E-22	0.9611	0.9919	v6.0 vs. v7.1	HNU
LH_inferiorparietal_surfavg	ICC3	0.983	577.70	26	234	1.20E-196	0.9739	0.9900	v7.1	HNU
LH_inferiorparietal_thickavg	ICC3	0.802	41.41	26	234	7.20E-73	0.7209	0.8754	v5.3	HNU
LH_inferiorparietal_thickavg	ICC2	0.779	21.45	26	26	8.54E-12	0.1082	0.9202	v5.3 vs. v6.0	HNU
LH_inferiorparietal_thickavg	ICC2	0.806	10.02	26	26	5.12E-08	0.6538	0.8949	v5.3 vs. v7.1	HNU
LH_inferiorparietal_thickavg	ICC3	0.810	43.63	26	234	5.18E-75	0.7317	0.8811	v6.0	HNU
LH_inferiorparietal_thickavg	ICC2	0.812	15.32	26	26	4.42E-10	0.4742	0.9177	v6.0 vs. v7.1	HNU
LH_inferiorparietal_thickavg	ICC3	0.772	34.92	26	234	4.90E-66	0.6838	0.8553	v7.1	HNU
LH_inferiortemporal_surfavg	ICC3	0.937	148.91	26	234	3.53E-130	0.9052	0.9623	v5.3	HNU
LH_inferiortemporal_surfavg	ICC2	0.887	36.85	26	26	1.18E-14	0.4343	0.9594	v5.3 vs. v6.0	HNU
LH_inferiortemporal_surfavg	ICC2	0.839	20.76	26	26	1.26E-11	0.4418	0.9353	v5.3 vs. v7.1	HNU
LH_inferiortemporal_surfavg	ICC3	0.926	126.03	26	234	2.79E-122	0.8897	0.9557	v6.0	HNU
LH_inferiortemporal_surfavg	ICC2	0.965	54.02	26	26	1.00E-16	0.9331	0.9815	v6.0 vs. v7.1	HNU
LH_inferiortemporal_surfavg	ICC3	0.957	224.61	26	234	5.59E-150	0.9353	0.9747	v7.1	HNU
LH_inferiortemporal_thickavg	ICC3	0.538	12.64	26	234	1.18E-31	0.4183	0.6748	v5.3	HNU
LH_inferiortemporal_thickavg	ICC2	0.575	4.77	26	26	8.20E-05	0.2519	0.7677	v5.3 vs. v6.0	HNU
LH_inferiortemporal_thickavg	ICC2	0.627	4.69	26	26	9.46E-05	0.3882	0.7867	v5.3 vs. v7.1	HNU

LH_inferiortemporal_thickavg	ICC3	0.626	17.75	26	234	8.91E-42	0.5123	0.7472	v6.0	HNU
LH_inferiortemporal_thickavg	ICC2	0.583	11.69	26	26	9.45E-09	-0.0421	0.8314	v6.0 vs. v7.1	HNU
LH_inferiortemporal_thickavg	ICC3	0.622	17.47	26	234	2.93E-41	0.5079	0.7440	v7.1	HNU
LH_insula_surfavg	ICC3	0.846	56.12	26	234	1.23E-85	0.7796	0.9053	v5.3	HNU
LH_insula_surfavg	ICC2	0.626	29.26	26	26	2.01E-13	-0.0325	0.8677	v5.3 vs. v6.0	HNU
LH_insula_surfavg	ICC2	0.647	19.08	26	26	3.42E-11	-0.0370	0.8709	v5.3 vs. v7.1	HNU
LH_insula_surfavg	ICC3	0.851	58.12	26	234	3.77E-87	0.7857	0.9083	v6.0	HNU
LH_insula_surfavg	ICC2	0.858	13.98	26	26	1.26E-09	0.7424	0.9242	v6.0 vs. v7.1	HNU
LH_insula_surfavg	ICC3	0.887	79.62	26	234	4.48E-101	0.8350	0.9315	v7.1	HNU
LH_insula_thickavg	ICC3	0.590	15.41	26	234	2.24E-37	0.4733	0.7185	v5.3	HNU
LH_insula_thickavg	ICC2	0.584	4.21	26	26	2.43E-04	0.3269	0.7603	v5.3 vs. v6.0	HNU
LH_insula_thickavg	ICC2	0.700	7.58	26	26	9.66E-07	0.3936	0.8476	v5.3 vs. v7.1	HNU
LH_insula_thickavg	ICC3	0.516	11.64	26	234	1.96E-29	0.3955	0.6556	v6.0	HNU
LH_insula_thickavg	ICC2	0.704	5.65	26	26	1.73E-05	0.4986	0.8349	v6.0 vs. v7.1	HNU
LH_insula_thickavg	ICC3	0.661	20.46	26	234	1.95E-46	0.5507	0.7738	v7.1	HNU
LH_isthmuscingulate_surfavg	ICC3	0.764	33.32	26	234	3.35E-64	0.6731	0.8493	v5.3	HNU
LH_isthmuscingulate_surfavg	ICC2	0.746	7.96	26	26	5.84E-07	0.5385	0.8635	v5.3 vs. v6.0	HNU
LH_isthmuscingulate_surfavg	ICC2	0.776	9.46	26	26	9.50E-08	0.5731	0.8824	v5.3 vs. v7.1	HNU
LH_isthmuscingulate_surfavg	ICC3	0.969	313.55	26	234	2.58E-166	0.9529	0.9818	v6.0	HNU
LH_isthmuscingulate_surfavg	ICC2	0.963	51.17	26	26	1.98E-16	0.9298	0.9807	v6.0 vs. v7.1	HNU
LH_isthmuscingulate_surfavg	ICC3	0.966	288.34	26	234	3.43E-162	0.9489	0.9802	v7.1	HNU
LH_isthmuscingulate_thickavg	ICC3	0.780	36.55	26	234	7.63E-68	0.6941	0.8609	v5.3	HNU
LH_isthmuscingulate_thickavg	ICC2	0.733	7.58	26	26	9.75E-07	0.5165	0.8564	v5.3 vs. v6.0	HNU
LH_isthmuscingulate_thickavg	ICC2	0.593	6.44	26	26	4.94E-06	0.1318	0.8032	v5.3 vs. v7.1	HNU
LH_isthmuscingulate_thickavg	ICC3	0.899	89.74	26	234	1.64E-106	0.8511	0.9388	v6.0	HNU
LH_isthmuscingulate_thickavg	ICC2	0.864	20.07	26	26	1.88E-11	0.6335	0.9389	v6.0 vs. v7.1	HNU
LH_isthmuscingulate_thickavg	ICC3	0.894	85.03	26	234	4.69E-104	0.8440	0.9356	v7.1	HNU
LH_lateraloccipital_surfavg	ICC3	0.963	261.49	26	234	2.13E-157	0.9440	0.9782	v5.3	HNU
LH_lateraloccipital_surfavg	ICC2	0.834	21.93	26	26	6.54E-12	0.3641	0.9356	v5.3 vs. v6.0	HNU
LH_lateraloccipital_surfavg	ICC2	0.726	20.62	26	26	1.37E-11	0.0092	0.9025	v5.3 vs. v7.1	HNU
LH_lateraloccipital_surfavg	ICC3	0.983	590.85	26	234	9.02E-198	0.9745	0.9903	v6.0	HNU
LH_lateraloccipital_surfavg	ICC2	0.951	117.56	26	26	5.18E-21	0.5254	0.9847	v6.0 vs. v7.1	HNU
LH_lateraloccipital_surfavg	ICC3	0.978	446.73	26	234	7.80E-184	0.9665	0.9871	v7.1	HNU
LH_lateraloccipital_thickavg	ICC3	0.733	28.44	26	234	3.63E-58	0.6355	0.8274	v5.3	HNU
LH_lateraloccipital_thickavg	ICC2	0.523	7.62	26	26	9.15E-07	-0.0362	0.7856	v5.3 vs. v6.0	HNU
LH_lateraloccipital_thickavg	ICC2	0.743	10.32	26	26	3.71E-08	0.3779	0.8809	v5.3 vs. v7.1	HNU
LH_lateraloccipital_thickavg	ICC3	0.813	44.60	26	234	6.36E-76	0.7362	0.8834	v6.0	HNU
LH_lateraloccipital_thickavg	ICC2	0.791	16.91	26	26	1.41E-10	0.2823	0.9167	v6.0 vs. v7.1	HNU
LH_lateraloccipital_thickavg	ICC3	0.824	47.71	26	234	9.84E-79	0.7495	0.8902	v7.1	HNU
LH_lateralorbitofrontal_surfavg	ICC3	0.822	47.20	26	234	2.76E-78	0.7474	0.8892	v5.3	HNU
LH_lateralorbitofrontal_surfavg	ICC2	0.897	70.00	26	26	3.81E-18	0.1737	0.9684	v5.3 vs. v6.0	HNU
LH_lateralorbitofrontal_surfavg	ICC2	0.784	44.29	26	26	1.20E-15	-0.0031	0.9320	v5.3 vs. v7.1	HNU
LH_lateralorbitofrontal_surfavg	ICC3	0.854	59.69	26	234	2.65E-88	0.7903	0.9105	v6.0	HNU
LH_lateralorbitofrontal_surfavg	ICC2	0.949	93.77	26	26	9.29E-20	0.6111	0.9829	v6.0 vs. v7.1	HNU

LH_lateralorbitofrontal_surfavg	ICC3	0.858	61.28	26	234	1.90E-89	0.7947	0.9126	v7.1	HNU
LH_lateralorbitofrontal_thickavg	ICC3	0.562	13.81	26	234	3.72E-34	0.4430	0.6949	v5.3	HNU
LH_lateralorbitofrontal_thickavg	ICC2	0.555	6.45	26	26	4.84E-06	0.0464	0.7896	v5.3 vs. v6.0	HNU
LH_lateralorbitofrontal_thickavg	ICC2	0.659	4.94	26	26	6.02E-05	0.4368	0.8064	v5.3 vs. v7.1	HNU
LH_lateralorbitofrontal_thickavg	ICC3	0.646	19.25	26	234	2.05E-44	0.5344	0.7627	v6.0	HNU
LH_lateralorbitofrontal_thickavg	ICC2	0.646	8.30	26	26	3.82E-07	0.1441	0.8399	v6.0 vs. v7.1	HNU
LH_lateralorbitofrontal_thickavg	ICC3	0.590	15.38	26	234	2.58E-37	0.4727	0.7180	v7.1	HNU
LH_LateralVentricle	ICC3	0.998	4411.36	26	234	3.43E-299	0.9965	0.9987	v5.3	HNU
LH_LateralVentricle	ICC2	0.994	3267.33	26	26	1.07E-39	0.6589	0.9986	v5.3 vs. v6.0	HNU
LH_LateralVentricle	ICC2	0.992	2297.69	26	26	1.03E-37	0.5745	0.9980	v5.3 vs. v7.1	HNU
LH_LateralVentricle	ICC3	0.998	5723.74	26	234	2.12573047	0.9973	0.9990	v6.0	HNU
LH_LateralVentricle	ICC2	1.000	6984.10	26	26	5.51E-44	0.9983	0.9998	v6.0 vs. v7.1	HNU
LH_LateralVentricle	ICC3	0.998	5720.17	26	234	2.28660433	0.9973	0.9990	v7.1	HNU
LH_lingual_surfavg	ICC3	0.930	134.31	26	234	2.78E-125	0.8959	0.9584	v5.3	HNU
LH_lingual_surfavg	ICC2	0.864	13.50	26	26	1.88E-09	0.7546	0.9271	v5.3 vs. v6.0	HNU
LH_lingual_surfavg	ICC2	0.850	13.71	26	26	1.58E-09	0.7216	0.9207	v5.3 vs. v7.1	HNU
LH_lingual_surfavg	ICC3	0.966	283.82	26	234	2.05E-161	0.9482	0.9799	v6.0	HNU
LH_lingual_surfavg	ICC2	0.943	84.52	26	26	3.48E-19	0.5744	0.9810	v6.0 vs. v7.1	HNU
LH_lingual_surfavg	ICC3	0.973	368.18	26	234	3.05E-174	0.9596	0.9844	v7.1	HNU
LH_lingual_thickavg	ICC3	0.723	27.07	26	234	2.53E-56	0.6232	0.8201	v5.3	HNU
LH_lingual_thickavg	ICC2	0.527	5.91	26	26	1.13E-05	0.0291	0.7712	v5.3 vs. v6.0	HNU
LH_lingual_thickavg	ICC2	0.745	7.26	26	26	1.50E-06	0.5601	0.8588	v5.3 vs. v7.1	HNU
LH_lingual_thickavg	ICC3	0.759	32.48	26	234	3.30E-63	0.6672	0.8459	v6.0	HNU
LH_lingual_thickavg	ICC2	0.676	10.29	26	26	3.83E-08	0.1211	0.8624	v6.0 vs. v7.1	HNU
LH_lingual_thickavg	ICC3	0.779	36.15	26	234	2.08E-67	0.6916	0.8596	v7.1	HNU
LH_medialorbitofrontal_surfavg	ICC3	0.679	22.15	26	234	3.73E-49	0.5718	0.7878	v5.3	HNU
LH_medialorbitofrontal_surfavg	ICC2	0.808	9.20	26	26	1.28E-07	0.6600	0.8952	v5.3 vs. v6.0	HNU
LH_medialorbitofrontal_surfavg	ICC2	0.634	9.56	26	26	8.55E-08	0.0499	0.8440	v5.3 vs. v7.1	HNU
LH_medialorbitofrontal_surfavg	ICC3	0.769	34.21	26	234	3.12E-65	0.6792	0.8527	v6.0	HNU
LH_medialorbitofrontal_surfavg	ICC2	0.709	15.80	26	26	3.10E-10	0.0373	0.8900	v6.0 vs. v7.1	HNU
LH_medialorbitofrontal_surfavg	ICC3	0.823	47.37	26	234	1.98E-78	0.7481	0.8895	v7.1	HNU
LH_medialorbitofrontal_thickavg	ICC3	0.527	12.16	26	234	1.36E-30	0.4075	0.6658	v5.3	HNU
LH_medialorbitofrontal_thickavg	ICC2	0.559	5.50	26	26	2.25E-05	0.1287	0.7772	v5.3 vs. v6.0	HNU
LH_medialorbitofrontal_thickavg	ICC2	0.580	3.66	26	26	7.51E-04	0.3182	0.7589	v5.3 vs. v7.1	HNU
LH_medialorbitofrontal_thickavg	ICC3	0.599	15.96	26	234	1.94E-38	0.4830	0.7257	v6.0	HNU
LH_medialorbitofrontal_thickavg	ICC2	0.515	4.37	26	26	1.76E-04	0.1412	0.7370	v6.0 vs. v7.1	HNU
LH_medialorbitofrontal_thickavg	ICC3	0.549	13.16	26	234	8.95E-33	0.4295	0.6840	v7.1	HNU
LH_middletemporal_surfavg	ICC3	0.932	138.71	26	234	8.28E-127	0.8989	0.9596	v5.3	HNU
LH_middletemporal_surfavg	ICC2	0.793	17.05	26	26	1.28E-10	0.2893	0.9176	v5.3 vs. v6.0	HNU
LH_middletemporal_surfavg	ICC2	0.739	19.42	26	26	2.77E-11	0.0408	0.9055	v5.3 vs. v7.1	HNU
LH_middletemporal_surfavg	ICC3	0.946	174.79	26	234	7.59E-138	0.9182	0.9677	v6.0	HNU
LH_middletemporal_surfavg	ICC2	0.954	57.79	26	26	4.28E-17	0.8817	0.9792	v6.0 vs. v7.1	HNU
LH_middletemporal_surfavg	ICC3	0.962	255.04	26	234	3.54E-156	0.9426	0.9777	v7.1	HNU
LH_middletemporal_thickavg	ICC3	0.745	30.14	26	234	2.40E-60	0.6495	0.8357	v5.3	HNU

LH_middletemporal_thickavg	ICC2	0.831	13.16	26	26	2.51E-09	0.6612	0.9139	v5.3 vs. v6.0	HNU
LH_middletemporal_thickavg	ICC2	0.857	15.08	26	26	5.32E-10	0.7200	0.9258	v5.3 vs. v7.1	HNU
LH_middletemporal_thickavg	ICC3	0.848	56.72	26	234	4.26E-86	0.7815	0.9062	v6.0	HNU
LH_middletemporal_thickavg	ICC2	0.785	23.16	26	26	3.40E-12	0.0977	0.9236	v6.0 vs. v7.1	HNU
LH_middletemporal_thickavg	ICC3	0.844	54.93	26	234	1.03E-84	0.7758	0.9034	v7.1	HNU
LH_Pallidum	ICC3	0.759	32.42	26	234	3.90E-63	0.6667	0.8457	v5.3	HNU
LH_Pallidum	ICC2	0.524	4.37	26	26	1.76E-04	0.1639	0.7404	v5.3 vs. v6.0	HNU
LH_Pallidum	ICC2	0.459	3.98	26	26	3.87E-04	0.0656	0.7035	v5.3 vs. v7.1	HNU
LH_Pallidum	ICC3	0.854	59.51	26	234	3.58E-88	0.7898	0.9102	v6.0	HNU
LH_Pallidum	ICC2	0.937	44.18	26	26	1.24E-15	0.8168	0.9722	v6.0 vs. v7.1	HNU
LH_Pallidum	ICC3	0.839	53.00	26	234	3.49E-83	0.7693	0.9002	v7.1	HNU
LH_paracentral_surfavg	ICC3	0.946	174.96	26	234	6.80E-138	0.9183	0.9678	v5.3	HNU
LH_paracentral_surfavg	ICC2	0.849	13.61	26	26	1.71E-09	0.7201	0.9202	v5.3 vs. v6.0	HNU
LH_paracentral_surfavg	ICC2	0.839	12.82	26	26	3.37E-09	0.6985	0.9147	v5.3 vs. v7.1	HNU
LH_paracentral_surfavg	ICC3	0.949	187.43	26	234	3.28E-141	0.9234	0.9698	v6.0	HNU
LH_paracentral_surfavg	ICC2	0.977	82.31	26	26	4.88E-19	0.9556	0.9878	v6.0 vs. v7.1	HNU
LH_paracentral_surfavg	ICC3	0.954	210.24	26	234	9.12E-147	0.9312	0.9730	v7.1	HNU
LH_paracentral_thickavg	ICC3	0.726	27.44	26	234	7.79E-57	0.6267	0.8222	v5.3	HNU
LH_paracentral_thickavg	ICC2	0.535	17.74	26	26	8.06E-11	-0.0440	0.8167	v5.3 vs. v6.0	HNU
LH_paracentral_thickavg	ICC2	0.521	12.84	26	26	3.31E-09	-0.0534	0.8029	v5.3 vs. v7.1	HNU
LH_paracentral_thickavg	ICC3	0.739	29.36	26	234	2.36E-59	0.6432	0.8320	v6.0	HNU
LH_paracentral_thickavg	ICC2	0.869	13.76	26	26	1.52E-09	0.7608	0.9297	v6.0 vs. v7.1	HNU
LH_paracentral_thickavg	ICC3	0.732	28.25	26	234	6.63E-58	0.6338	0.8264	v7.1	HNU
LH_parahippocampal_surfavg	ICC3	0.504	11.17	26	234	2.39E-28	0.3841	0.6456	v5.3	HNU
LH_parahippocampal_surfavg	ICC2	0.481	7.31	26	26	1.40E-06	-0.0547	0.7616	v5.3 vs. v6.0	HNU
LH_parahippocampal_surfavg	ICC2	0.372	5.09	26	26	4.58E-05	-0.0673	0.6740	v5.3 vs. v7.1	HNU
LH_parahippocampal_surfavg	ICC3	0.945	174.26	26	234	1.06E-137	0.9180	0.9676	v6.0	HNU
LH_parahippocampal_surfavg	ICC2	0.871	15.38	26	26	4.24E-10	0.7643	0.9311	v6.0 vs. v7.1	HNU
LH_parahippocampal_surfavg	ICC3	0.914	107.22	26	234	1.02E-114	0.8726	0.9483	v7.1	HNU
LH_parahippocampal_thickavg	ICC3	0.864	64.77	26	234	7.05E-92	0.8038	0.9170	v5.3	HNU
LH_parahippocampal_thickavg	ICC2	0.847	32.77	26	26	5.00E-14	0.2065	0.9475	v5.3 vs. v6.0	HNU
LH_parahippocampal_thickavg	ICC2	0.916	22.63	26	26	4.50E-12	0.8447	0.9552	v5.3 vs. v7.1	HNU
LH_parahippocampal_thickavg	ICC3	0.896	87.06	26	234	3.96E-105	0.8471	0.9370	v6.0	HNU
LH_parahippocampal_thickavg	ICC2	0.788	24.07	26	26	2.14E-12	0.0944	0.9253	v6.0 vs. v7.1	HNU
LH_parahippocampal_thickavg	ICC3	0.899	89.88	26	234	1.38E-106	0.8513	0.9389	v7.1	HNU
LH_parsopercularis_surfavg	ICC3	0.984	599.10	26	234	1.83E-198	0.9748	0.9904	v5.3	HNU
LH_parsopercularis_surfavg	ICC2	0.915	29.90	26	26	1.54E-13	0.7939	0.9602	v5.3 vs. v6.0	HNU
LH_parsopercularis_surfavg	ICC2	0.923	31.76	26	26	7.37E-14	0.8253	0.9631	v5.3 vs. v7.1	HNU
LH_parsopercularis_surfavg	ICC3	0.992	1297.23	26	234	2.84E-237	0.9882	0.9955	v6.0	HNU
LH_parsopercularis_surfavg	ICC2	0.989	184.84	26	26	1.55E-23	0.9796	0.9944	v6.0 vs. v7.1	HNU
LH_parsopercularis_surfavg	ICC3	0.991	1052.30	26	234	9.93E-227	0.9855	0.9945	v7.1	HNU
LH_parsopercularis_thickavg	ICC3	0.773	35.02	26	234	3.76E-66	0.6845	0.8556	v5.3	HNU
LH_parsopercularis_thickavg	ICC2	0.727	17.65	26	26	8.52E-11	0.0416	0.8991	v5.3 vs. v6.0	HNU
LH_parsopercularis_thickavg	ICC2	0.854	28.44	26	26	2.84E-13	0.3298	0.9466	v5.3 vs. v7.1	HNU

LH_parsopercularis_thickavg	ICC3	0.853	58.80	26	234	1.19E-87	0.7877	0.9093	v6.0	HNU
LH_parsopercularis_thickavg	ICC2	0.900	24.20	26	26	2.01E-12	0.7751	0.9516	v6.0 vs. v7.1	HNU
LH_parsopercularis_thickavg	ICC3	0.828	49.18	26	234	5.26E-80	0.7553	0.8932	v7.1	HNU
LH_parsorbitalis_surfavg	ICC3	0.895	86.64	26	234	6.57E-105	0.8465	0.9368	v5.3	HNU
LH_parsorbitalis_surfavg	ICC2	0.744	22.33	26	26	5.27E-12	0.0195	0.9099	v5.3 vs. v6.0	HNU
LH_parsorbitalis_surfavg	ICC2	0.562	9.92	26	26	5.72E-08	-0.0405	0.8160	v5.3 vs. v7.1	HNU
LH_parsorbitalis_surfavg	ICC3	0.940	157.88	26	234	5.70E-133	0.9102	0.9644	v6.0	HNU
LH_parsorbitalis_surfavg	ICC2	0.899	31.77	26	26	7.32E-14	0.6419	0.9590	v6.0 vs. v7.1	HNU
LH_parsorbitalis_surfavg	ICC3	0.950	189.38	26	234	1.04E-141	0.9241	0.9701	v7.1	HNU
LH_parsorbitalis_thickavg	ICC3	0.771	34.58	26	234	1.20E-65	0.6816	0.8540	v5.3	HNU
LH_parsorbitalis_thickavg	ICC2	0.711	11.09	26	26	1.69E-08	0.1957	0.8769	v5.3 vs. v6.0	HNU
LH_parsorbitalis_thickavg	ICC2	0.766	8.46	26	26	3.09E-07	0.5795	0.8732	v5.3 vs. v7.1	HNU
LH_parsorbitalis_thickavg	ICC3	0.788	38.19	26	234	1.35E-69	0.7037	0.8662	v6.0	HNU
LH_parsorbitalis_thickavg	ICC2	0.769	9.47	26	26	9.45E-08	0.5477	0.8808	v6.0 vs. v7.1	HNU
LH_parsorbitalis_thickavg	ICC3	0.761	32.76	26	234	1.52E-63	0.6692	0.8471	v7.1	HNU
LH_parstriangularis_surfavg	ICC3	0.969	318.00	26	234	5.22E-167	0.9535	0.9820	v5.3	HNU
LH_parstriangularis_surfavg	ICC2	0.852	13.77	26	26	1.50E-09	0.7256	0.9214	v5.3 vs. v6.0	HNU
LH_parstriangularis_surfavg	ICC2	0.814	11.91	26	26	7.72E-09	0.6305	0.9050	v5.3 vs. v7.1	HNU
LH_parstriangularis_surfavg	ICC3	0.982	559.83	26	234	4.45E-195	0.9731	0.9897	v6.0	HNU
LH_parstriangularis_surfavg	ICC2	0.982	137.77	26	26	6.78E-22	0.9561	0.9914	v6.0 vs. v7.1	HNU
LH_parstriangularis_surfavg	ICC3	0.989	883.69	26	234	6.02E-218	0.9828	0.9935	v7.1	HNU
LH_parstriangularis_thickavg	ICC3	0.791	38.76	26	234	3.45E-70	0.7069	0.8679	v5.3	HNU
LH_parstriangularis_thickavg	ICC2	0.579	13.61	26	26	1.71E-09	-0.0482	0.8336	v5.3 vs. v6.0	HNU
LH_parstriangularis_thickavg	ICC2	0.743	14.58	26	26	7.79E-10	0.1584	0.8981	v5.3 vs. v7.1	HNU
LH_parstriangularis_thickavg	ICC3	0.835	51.42	26	234	6.75E-82	0.7637	0.8974	v6.0	HNU
LH_parstriangularis_thickavg	ICC2	0.840	17.28	26	26	1.10E-10	0.5682	0.9286	v6.0 vs. v7.1	HNU
LH_parstriangularis_thickavg	ICC3	0.791	38.82	26	234	2.98E-70	0.7072	0.8681	v7.1	HNU
LH_pericalcarine_surfavg	ICC3	0.891	82.74	26	234	8.20E-103	0.8403	0.9339	v5.3	HNU
LH_pericalcarine_surfavg	ICC2	0.883	33.61	26	26	3.67E-14	0.4570	0.9568	v5.3 vs. v6.0	HNU
LH_pericalcarine_surfavg	ICC2	0.851	24.39	26	26	1.83E-12	0.4106	0.9424	v5.3 vs. v7.1	HNU
LH_pericalcarine_surfavg	ICC3	0.935	145.88	26	234	3.34E-129	0.9034	0.9615	v6.0	HNU
LH_pericalcarine_surfavg	ICC2	0.960	48.78	26	26	3.60E-16	0.9245	0.9789	v6.0 vs. v7.1	HNU
LH_pericalcarine_surfavg	ICC3	0.940	156.74	26	234	1.27E-132	0.9096	0.9641	v7.1	HNU
LH_pericalcarine_thickavg	ICC3	0.747	30.58	26	234	6.82E-61	0.6530	0.8377	v5.3	HNU
LH_pericalcarine_thickavg	ICC2	0.704	12.15	26	26	6.17E-09	0.1230	0.8792	v5.3 vs. v6.0	HNU
LH_pericalcarine_thickavg	ICC2	0.695	6.70	26	26	3.35E-06	0.4444	0.8361	v5.3 vs. v7.1	HNU
LH_pericalcarine_thickavg	ICC3	0.768	34.17	26	234	3.48E-65	0.6789	0.8525	v6.0	HNU
LH_pericalcarine_thickavg	ICC2	0.862	17.80	26	26	7.75E-11	0.6841	0.9338	v6.0 vs. v7.1	HNU
LH_pericalcarine_thickavg	ICC3	0.838	52.78	26	234	5.28E-83	0.7685	0.8998	v7.1	HNU
LH_postcentral_surfavg	ICC3	0.958	231.55	26	234	1.85E-151	0.9371	0.9755	v5.3	HNU
LH_postcentral_surfavg	ICC2	0.937	33.51	26	26	3.80E-14	0.8792	0.9673	v5.3 vs. v6.0	HNU
LH_postcentral_surfavg	ICC2	0.930	26.61	26	26	6.38E-13	0.8691	0.9631	v5.3 vs. v7.1	HNU
LH_postcentral_surfavg	ICC3	0.963	264.86	26	234	5.03E-158	0.9446	0.9785	v6.0	HNU
LH_postcentral_surfavg	ICC2	0.977	125.47	26	26	2.25E-21	0.9258	0.9901	v6.0 vs. v7.1	HNU

LH_postcentral_surfavg	ICC3	0.958	227.37	26	234	1.42E-150	0.9360	0.9750	v7.1	HNU
LH_postcentral_thickavg	ICC3	0.873	70.03	26	234	2.50E-95	0.8161	0.9228	v5.3	HNU
LH_postcentral_thickavg	ICC2	0.552	21.45	26	26	8.52E-12	-0.0386	0.8283	v5.3 vs. v6.0	HNU
LH_postcentral_thickavg	ICC2	0.594	28.29	26	26	3.03E-13	-0.0323	0.8522	v5.3 vs. v7.1	HNU
LH_postcentral_thickavg	ICC3	0.872	69.13	26	234	9.38E-95	0.8141	0.9219	v6.0	HNU
LH_postcentral_thickavg	ICC2	0.928	26.08	26	26	8.12E-13	0.8659	0.9619	v6.0 vs. v7.1	HNU
LH_postcentral_thickavg	ICC3	0.880	74.08	26	234	7.74E-98	0.8246	0.9267	v7.1	HNU
LH_posteriorcingulate_surfavg	ICC3	0.951	194.89	26	234	4.28E-143	0.9261	0.9710	v5.3	HNU
LH_posteriorcingulate_surfavg	ICC2	0.973	70.76	26	26	3.33E-18	0.9488	0.9860	v5.3 vs. v6.0	HNU
LH_posteriorcingulate_surfavg	ICC2	0.948	42.25	26	26	2.17E-15	0.8948	0.9733	v5.3 vs. v7.1	HNU
LH_posteriorcingulate_surfavg	ICC3	0.974	377.62	26	234	1.70E-175	0.9606	0.9848	v6.0	HNU
LH_posteriorcingulate_surfavg	ICC2	0.966	70.90	26	26	3.24E-18	0.9226	0.9835	v6.0 vs. v7.1	HNU
LH_posteriorcingulate_surfavg	ICC3	0.977	420.62	26	234	7.64E-181	0.9645	0.9864	v7.1	HNU
LH_posteriorcingulate_thickavg	ICC3	0.650	19.55	26	234	6.34E-45	0.5385	0.7655	v5.3	HNU
LH_posteriorcingulate_thickavg	ICC2	0.765	7.62	26	26	9.24E-07	0.5946	0.8703	v5.3 vs. v6.0	HNU
LH_posteriorcingulate_thickavg	ICC2	0.628	7.36	26	26	1.31E-06	0.1572	0.8255	v5.3 vs. v7.1	HNU
LH_posteriorcingulate_thickavg	ICC3	0.655	19.96	26	234	1.31E-45	0.5441	0.7693	v6.0	HNU
LH_posteriorcingulate_thickavg	ICC2	0.622	20.85	26	26	1.20E-11	-0.0406	0.8614	v6.0 vs. v7.1	HNU
LH_posteriorcingulate_thickavg	ICC3	0.700	24.34	26	234	1.82E-52	0.5963	0.8035	v7.1	HNU
LH_precentral_surfavg	ICC3	0.974	371.60	26	234	1.06E-174	0.9600	0.9846	v5.3	HNU
LH_precentral_surfavg	ICC2	0.933	40.16	26	26	4.06E-15	0.8197	0.9698	v5.3 vs. v6.0	HNU
LH_precentral_surfavg	ICC2	0.929	30.62	26	26	1.15E-13	0.8600	0.9635	v5.3 vs. v7.1	HNU
LH_precentral_surfavg	ICC3	0.972	348.30	26	234	1.69E-171	0.9574	0.9836	v6.0	HNU
LH_precentral_surfavg	ICC2	0.986	153.12	26	26	1.75E-22	0.9717	0.9927	v6.0 vs. v7.1	HNU
LH_precentral_surfavg	ICC3	0.973	358.99	26	234	5.41E-173	0.9586	0.9840	v7.1	HNU
LH_precentral_thickavg	ICC3	0.816	45.41	26	234	1.15E-76	0.7398	0.8852	v5.3	HNU
LH_precentral_thickavg	ICC2	0.583	20.22	26	26	1.72E-11	-0.0420	0.8430	v5.3 vs. v6.0	HNU
LH_precentral_thickavg	ICC2	0.554	13.06	26	26	2.74E-09	-0.0517	0.8207	v5.3 vs. v7.1	HNU
LH_precentral_thickavg	ICC3	0.792	39.10	26	234	1.53E-70	0.7088	0.8689	v6.0	HNU
LH_precentral_thickavg	ICC2	0.890	16.93	26	26	1.39E-10	0.7990	0.9412	v6.0 vs. v7.1	HNU
LH_precentral_thickavg	ICC3	0.806	42.53	26	234	5.84E-74	0.7265	0.8783	v7.1	HNU
LH_precuneus_surfavg	ICC3	0.977	423.77	26	234	3.27E-181	0.9647	0.9865	v5.3	HNU
LH_precuneus_surfavg	ICC2	0.967	68.54	26	26	4.98E-18	0.9314	0.9834	v5.3 vs. v6.0	HNU
LH_precuneus_surfavg	ICC2	0.967	66.98	26	26	6.65E-18	0.9322	0.9831	v5.3 vs. v7.1	HNU
LH_precuneus_surfavg	ICC3	0.983	571.26	26	234	4.36E-196	0.9736	0.9899	v6.0	HNU
LH_precuneus_surfavg	ICC2	0.992	250.92	26	26	3.02E-25	0.9853	0.9960	v6.0 vs. v7.1	HNU
LH_precuneus_surfavg	ICC3	0.988	843.41	26	234	1.33E-215	0.9820	0.9932	v7.1	HNU
LH_precuneus_thickavg	ICC3	0.749	30.81	26	234	3.52E-61	0.6547	0.8388	v5.3	HNU
LH_precuneus_thickavg	ICC2	0.556	11.44	26	26	1.20E-08	-0.0504	0.8178	v5.3 vs. v6.0	HNU
LH_precuneus_thickavg	ICC2	0.772	14.69	26	26	7.19E-10	0.2776	0.9064	v5.3 vs. v7.1	HNU
LH_precuneus_thickavg	ICC3	0.793	39.23	26	234	1.12E-70	0.7095	0.8693	v6.0	HNU
LH_precuneus_thickavg	ICC2	0.716	9.64	26	26	7.75E-08	0.3019	0.8698	v6.0 vs. v7.1	HNU
LH_precuneus_thickavg	ICC3	0.778	36.02	26	234	2.92E-67	0.6908	0.8591	v7.1	HNU
LH_Putamen	ICC3	0.879	73.88	26	234	1.02E-97	0.8242	0.9266	v5.3	HNU

LH_Putamen	ICC2	0.284	21.14	26	26	1.01E-11	-0.0161	0.6219	v5.3 vs. v6.0	HNU
LH_Putamen	ICC2	0.298	18.22	26	26	5.87E-11	-0.0202	0.6361	v5.3 vs. v7.1	HNU
LH_Putamen	ICC3	0.842	54.12	26	234	4.47E-84	0.7731	0.9021	v6.0	HNU
LH_Putamen	ICC2	0.977	89.70	26	26	1.63E-19	0.9556	0.9880	v6.0 vs. v7.1	HNU
LH_Putamen	ICC3	0.960	240.73	26	234	2.34E-153	0.9394	0.9764	v7.1	HNU
LH_rostralanteriorcingulate_surfavg	ICC3	0.920	115.89	26	234	2.42E-118	0.8811	0.9520	v5.3	HNU
LH_rostralanteriorcingulate_surfavg	ICC2	0.908	20.08	26	26	1.87E-11	0.8301	0.9514	v5.3 vs. v6.0	HNU
LH_rostralanteriorcingulate_surfavg	ICC2	0.885	15.96	26	26	2.76E-10	0.7895	0.9385	v5.3 vs. v7.1	HNU
LH_rostralanteriorcingulate_surfavg	ICC3	0.920	116.21	26	234	1.79E-118	0.8814	0.9522	v6.0	HNU
LH_rostralanteriorcingulate_surfavg	ICC2	0.951	39.39	26	26	5.17E-15	0.9083	0.9743	v6.0 vs. v7.1	HNU
LH_rostralanteriorcingulate_surfavg	ICC3	0.952	199.52	26	234	3.13E-144	0.9277	0.9716	v7.1	HNU
LH_rostralanteriorcingulate_thickavg	ICC3	0.567	14.07	26	234	1.09E-34	0.4481	0.6990	v5.3	HNU
LH_rostralanteriorcingulate_thickavg	ICC2	0.637	5.06	26	26	4.77E-05	0.3916	0.7953	v5.3 vs. v6.0	HNU
LH_rostralanteriorcingulate_thickavg	ICC2	0.648	5.41	26	26	2.62E-05	0.3963	0.8040	v5.3 vs. v7.1	HNU
LH_rostralanteriorcingulate_thickavg	ICC3	0.621	17.37	26	234	4.44E-41	0.5063	0.7429	v6.0	HNU
LH_rostralanteriorcingulate_thickavg	ICC2	0.668	15.42	26	26	4.11E-10	-0.0122	0.8747	v6.0 vs. v7.1	HNU
LH_rostralanteriorcingulate_thickavg	ICC3	0.672	21.51	26	234	3.79E-48	0.5641	0.7827	v7.1	HNU
LH_rostralmiddlefrontal_surfavg	ICC3	0.980	484.86	26	234	6.56E-188	0.9691	0.9881	v5.3	HNU
LH_rostralmiddlefrontal_surfavg	ICC2	0.889	20.92	26	26	1.15E-11	0.7624	0.9448	v5.3 vs. v6.0	HNU
LH_rostralmiddlefrontal_surfavg	ICC2	0.903	29.89	26	26	1.54E-13	0.7037	0.9581	v5.3 vs. v7.1	HNU
LH_rostralmiddlefrontal_surfavg	ICC3	0.991	1096.17	26	234	8.72E-229	0.9861	0.9947	v6.0	HNU
LH_rostralmiddlefrontal_surfavg	ICC2	0.992	270.01	26	26	1.17E-25	0.9846	0.9959	v6.0 vs. v7.1	HNU
LH_rostralmiddlefrontal_surfavg	ICC3	0.992	1265.38	26	234	5.10E-236	0.9879	0.9954	v7.1	HNU
LH_rostralmiddlefrontal_thickavg	ICC3	0.712	25.72	26	234	1.83E-54	0.6104	0.8123	v5.3	HNU
LH_rostralmiddlefrontal_thickavg	ICC2	0.546	8.04	26	26	5.29E-07	-0.0263	0.7990	v5.3 vs. v6.0	HNU
LH_rostralmiddlefrontal_thickavg	ICC2	0.667	8.70	26	26	2.32E-07	0.1837	0.8496	v5.3 vs. v7.1	HNU
LH_rostralmiddlefrontal_thickavg	ICC3	0.765	33.53	26	234	1.90E-64	0.6746	0.8501	v6.0	HNU
LH_rostralmiddlefrontal_thickavg	ICC2	0.760	8.60	26	26	2.62E-07	0.5561	0.8723	v6.0 vs. v7.1	HNU
LH_rostralmiddlefrontal_thickavg	ICC3	0.699	24.25	26	234	2.46E-52	0.5954	0.8029	v7.1	HNU
LH_superiorfrontal_surfavg	ICC3	0.986	706.55	26	234	1.01E-206	0.9786	0.9918	v5.3	HNU
LH_superiorfrontal_surfavg	ICC2	0.970	151.35	26	26	2.03E-22	0.7795	0.9900	v5.3 vs. v6.0	HNU
LH_superiorfrontal_surfavg	ICC2	0.939	101.59	26	26	3.35E-20	0.4053	0.9811	v5.3 vs. v7.1	HNU
LH_superiorfrontal_surfavg	ICC3	0.991	1152.79	26	234	2.53E-231	0.9868	0.9950	v6.0	HNU
LH_superiorfrontal_surfavg	ICC2	0.989	380.20	26	26	1.41E-27	0.9154	0.9961	v6.0 vs. v7.1	HNU
LH_superiorfrontal_surfavg	ICC3	0.991	1101.93	26	234	4.75E-229	0.9862	0.9948	v7.1	HNU
LH_superiorfrontal_thickavg	ICC3	0.852	58.47	26	234	2.08E-87	0.7867	0.9088	v5.3	HNU
LH_superiorfrontal_thickavg	ICC2	0.672	12.87	26	26	3.24E-09	0.0268	0.8704	v5.3 vs. v6.0	HNU
LH_superiorfrontal_thickavg	ICC2	0.818	14.47	26	26	8.52E-10	0.5443	0.9161	v5.3 vs. v7.1	HNU
LH_superiorfrontal_thickavg	ICC3	0.867	66.12	26	234	8.70E-93	0.8071	0.9186	v6.0	HNU
LH_superiorfrontal_thickavg	ICC2	0.837	17.93	26	26	7.12E-11	0.5240	0.9295	v6.0 vs. v7.1	HNU
LH_superiorfrontal_thickavg	ICC3	0.827	48.82	26	234	1.06E-79	0.7539	0.8925	v7.1	HNU
LH_superiorparietal_surfavg	ICC3	0.975	385.47	26	234	1.63E-176	0.9614	0.9851	v5.3	HNU
LH_superiorparietal_surfavg	ICC2	0.940	45.08	26	26	9.63E-16	0.8351	0.9730	v5.3 vs. v6.0	HNU
LH_superiorparietal_surfavg	ICC2	0.921	44.97	26	26	9.94E-16	0.6522	0.9697	v5.3 vs. v7.1	HNU

LH_superiorparietal_surfavg	ICC3	0.989	890.92	26	234	2.35E-218	0.9829	0.9935	v6.0	HNU
LH_superiorparietal_surfavg	ICC2	0.991	385.20	26	26	1.19E-27	0.9608	0.9966	v6.0 vs. v7.1	HNU
LH_superiorparietal_surfavg	ICC3	0.986	721.95	26	234	8.41E-208	0.9790	0.9920	v7.1	HNU
LH_superiorparietal_thickavg	ICC3	0.846	55.93	26	234	1.72E-85	0.7790	0.9050	v5.3	HNU
LH_superiorparietal_thickavg	ICC2	0.598	19.67	26	26	2.39E-11	-0.0427	0.8499	v5.3 vs. v6.0	HNU
LH_superiorparietal_thickavg	ICC2	0.611	17.95	26	26	6.99E-11	-0.0434	0.8543	v5.3 vs. v7.1	HNU
LH_superiorparietal_thickavg	ICC3	0.845	55.57	26	234	3.29E-85	0.7778	0.9044	v6.0	HNU
LH_superiorparietal_thickavg	ICC2	0.855	12.49	26	26	4.51E-09	0.7385	0.9220	v6.0 vs. v7.1	HNU
LH_superiorparietal_thickavg	ICC3	0.777	35.87	26	234	4.28E-67	0.6899	0.8586	v7.1	HNU
LH_superiortemporal_surfavg	ICC3	0.965	272.81	26	234	1.78E-159	0.9462	0.9791	v5.3	HNU
LH_superiortemporal_surfavg	ICC2	0.876	45.77	26	26	7.98E-16	0.2190	0.9595	v5.3 vs. v6.0	HNU
LH_superiortemporal_surfavg	ICC2	0.810	62.36	26	26	1.64E-17	0.0020	0.9427	v5.3 vs. v7.1	HNU
LH_superiortemporal_surfavg	ICC3	0.971	336.33	26	234	9.01E-170	0.9559	0.9830	v6.0	HNU
LH_superiortemporal_surfavg	ICC2	0.974	255.25	26	26	2.42E-25	0.6280	0.9922	v6.0 vs. v7.1	HNU
LH_superiortemporal_surfavg	ICC3	0.984	630.38	26	234	5.21E-201	0.9760	0.9909	v7.1	HNU
LH_superiortemporal_thickavg	ICC3	0.798	40.40	26	234	7.27E-72	0.7157	0.8727	v5.3	HNU
LH_superiortemporal_thickavg	ICC2	0.726	17.07	26	26	1.27E-10	0.0490	0.8979	v5.3 vs. v6.0	HNU
LH_superiortemporal_thickavg	ICC2	0.846	12.91	26	26	3.11E-09	0.7208	0.9177	v5.3 vs. v7.1	HNU
LH_superiortemporal_thickavg	ICC3	0.837	52.32	26	234	1.26E-82	0.7669	0.8990	v6.0	HNU
LH_superiortemporal_thickavg	ICC2	0.752	12.41	26	26	4.85E-09	0.2946	0.8936	v6.0 vs. v7.1	HNU
LH_superiortemporal_thickavg	ICC3	0.782	36.79	26	234	4.23E-68	0.6955	0.8617	v7.1	HNU
LH_supramarginal_surfavg	ICC3	0.976	415.58	26	234	3.03E-180	0.9641	0.9862	v5.3	HNU
LH_supramarginal_surfavg	ICC2	0.820	27.21	26	26	4.87E-13	0.1640	0.9371	v5.3 vs. v6.0	HNU
LH_supramarginal_surfavg	ICC2	0.787	24.81	26	26	1.48E-12	0.0828	0.9257	v5.3 vs. v7.1	HNU
LH_supramarginal_surfavg	ICC3	0.992	1194.70	26	234	4.02E-233	0.9872	0.9952	v6.0	HNU
LH_supramarginal_surfavg	ICC2	0.993	380.12	26	26	1.41E-27	0.9784	0.9968	v6.0 vs. v7.1	HNU
LH_supramarginal_surfavg	ICC3	0.992	1189.35	26	234	6.77E-233	0.9872	0.9951	v7.1	HNU
LH_supramarginal_thickavg	ICC3	0.788	38.24	26	234	1.19E-69	0.7040	0.8663	v5.3	HNU
LH_supramarginal_thickavg	ICC2	0.588	7.23	26	26	1.57E-06	0.0650	0.8104	v5.3 vs. v6.0	HNU
LH_supramarginal_thickavg	ICC2	0.817	12.80	26	26	3.44E-09	0.6073	0.9093	v5.3 vs. v7.1	HNU
LH_supramarginal_thickavg	ICC3	0.806	42.53	26	234	5.78E-74	0.7265	0.8783	v6.0	HNU
LH_supramarginal_thickavg	ICC2	0.721	8.72	26	26	2.27E-07	0.3925	0.8639	v6.0 vs. v7.1	HNU
LH_supramarginal_thickavg	ICC3	0.759	32.49	26	234	3.22E-63	0.6673	0.8459	v7.1	HNU
LH_SurfArea	ICC3	0.990	996.24	26	234	5.65E-224	0.9847	0.9942	v5.3	HNU
LH_SurfArea	ICC2	0.974	678.39	26	26	7.79E-31	0.2670	0.9931	v5.3 vs. v6.0	HNU
LH_SurfArea	ICC2	0.928	405.64	26	26	6.09E-28	0.0618	0.9811	v5.3 vs. v7.1	HNU
LH_SurfArea	ICC3	0.994	1558.86	26	234	1.52E-246	0.9902	0.9963	v6.0	HNU
LH_SurfArea	ICC2	0.985	1076.30	26	26	1.96E-33	0.4504	0.9962	v6.0 vs. v7.1	HNU
LH_SurfArea	ICC3	0.994	1646.95	26	234	2.55E-249	0.9907	0.9965	v7.1	HNU
LH_temporalpole_surfavg	ICC3	0.670	21.27	26	234	9.36E-48	0.5611	0.7807	v5.3	HNU
LH_temporalpole_surfavg	ICC2	0.536	3.28	26	26	1.78E-03	0.2644	0.7294	v5.3 vs. v6.0	HNU
LH_temporalpole_surfavg	ICC2	0.437	3.08	26	26	2.81E-03	0.1301	0.6644	v5.3 vs. v7.1	HNU
LH_temporalpole_surfavg	ICC3	0.815	45.09	26	234	2.25E-76	0.7383	0.8845	v6.0	HNU
LH_temporalpole_surfavg	ICC2	0.611	6.73	26	26	3.23E-06	0.1572	0.8127	v6.0 vs. v7.1	HNU

LH_temporalpole_surfavg	ICC3	0.846	55.87	26	234	1.91E-85	0.7788	0.9049	v7.1	HNU
LH_temporalpole_thickavg	ICC3	0.407	7.87	26	234	3.84E-20	0.2906	0.5563	v5.3	HNU
LH_temporalpole_thickavg	ICC2	0.756	8.06	26	26	5.14E-07	0.5669	0.8677	v5.3 vs. v6.0	HNU
LH_temporalpole_thickavg	ICC2	0.826	10.34	26	26	3.66E-08	0.6905	0.9055	v5.3 vs. v7.1	HNU
LH_temporalpole_thickavg	ICC3	0.405	7.79	26	234	5.98E-20	0.2883	0.5538	v6.0	HNU
LH_temporalpole_thickavg	ICC2	0.841	17.49	26	26	9.48E-11	0.5672	0.9293	v6.0 vs. v7.1	HNU
LH_temporalpole_thickavg	ICC3	0.391	7.42	26	234	6.05E-19	0.2758	0.5407	v7.1	HNU
LH_Thalamus	ICC3	0.857	60.69	26	234	5.03E-89	0.7931	0.9119	v5.3	HNU
LH_Thalamus	ICC2	0.844	25.06	26	26	1.32E-12	0.3440	0.9415	v5.3 vs. v6.0	HNU
LH_Thalamus	ICC2	0.700	16.02	26	26	2.63E-10	0.0197	0.8875	v5.3 vs. v7.1	HNU
LH_Thalamus	ICC3	0.918	113.51	26	234	2.26E-117	0.8789	0.9511	v6.0	HNU
LH_Thalamus	ICC2	0.915	47.37	26	26	5.20E-16	0.5555	0.9692	v6.0 vs. v7.1	HNU
LH_Thalamus	ICC3	0.899	89.75	26	234	1.63E-106	0.8511	0.9388	v7.1	HNU
LH_Thickness	ICC3	0.777	35.75	26	234	5.85E-67	0.6891	0.8582	v5.3	HNU
LH_Thickness	ICC2	0.467	8.60	26	26	2.62E-07	-0.0616	0.7607	v5.3 vs. v6.0	HNU
LH_Thickness	ICC2	0.721	10.12	26	26	4.60E-08	0.2909	0.8742	v5.3 vs. v7.1	HNU
LH_Thickness	ICC3	0.822	47.24	26	234	2.57E-78	0.7476	0.8893	v6.0	HNU
LH_Thickness	ICC2	0.657	8.35	26	26	3.58E-07	0.1754	0.8438	v6.0 vs. v7.1	HNU
LH_Thickness	ICC3	0.759	32.42	26	234	3.83E-63	0.6668	0.8457	v7.1	HNU
LH_transversetemporal_surfavg	ICC3	0.948	183.16	26	234	4.24E-140	0.9217	0.9692	v5.3	HNU
LH_transversetemporal_surfavg	ICC2	0.930	29.31	26	26	1.97E-13	0.8669	0.9631	v5.3 vs. v6.0	HNU
LH_transversetemporal_surfavg	ICC2	0.923	24.21	26	26	2.00E-12	0.8568	0.9594	v5.3 vs. v7.1	HNU
LH_transversetemporal_surfavg	ICC3	0.952	198.46	26	234	5.68E-144	0.9273	0.9715	v6.0	HNU
LH_transversetemporal_surfavg	ICC2	0.967	84.00	26	26	3.77E-19	0.9021	0.9854	v6.0 vs. v7.1	HNU
LH_transversetemporal_surfavg	ICC3	0.970	319.57	26	234	2.98E-167	0.9537	0.9821	v7.1	HNU
LH_transversetemporal_thickavg	ICC3	0.840	53.54	26	234	1.28E-83	0.7712	0.9011	v5.3	HNU
LH_transversetemporal_thickavg	ICC2	0.771	26.44	26	26	6.90E-13	0.0325	0.9219	v5.3 vs. v6.0	HNU
LH_transversetemporal_thickavg	ICC2	0.855	24.93	26	26	1.40E-12	0.4298	0.9440	v5.3 vs. v7.1	HNU
LH_transversetemporal_thickavg	ICC3	0.837	52.40	26	234	1.07E-82	0.7672	0.8992	v6.0	HNU
LH_transversetemporal_thickavg	ICC2	0.928	38.93	26	26	5.97E-15	0.7955	0.9685	v6.0 vs. v7.1	HNU
LH_transversetemporal_thickavg	ICC3	0.827	48.68	26	234	1.40E-79	0.7534	0.8922	v7.1	HNU
RH_Accumbens	ICC3	0.608	16.51	26	234	1.73E-39	0.4924	0.7327	v5.3	HNU
RH_Accumbens	ICC2	0.485	6.67	26	26	3.48E-06	-0.0447	0.7590	v5.3 vs. v6.0	HNU
RH_Accumbens	ICC2	0.581	5.28	26	26	3.24E-05	0.2111	0.7805	v5.3 vs. v7.1	HNU
RH_Accumbens	ICC3	0.718	26.42	26	234	1.95E-55	0.6172	0.8164	v6.0	HNU
RH_Accumbens	ICC2	0.729	7.70	26	26	8.21E-07	0.4943	0.8563	v6.0 vs. v7.1	HNU
RH_Accumbens	ICC3	0.750	30.97	26	234	2.20E-61	0.6560	0.8395	v7.1	HNU
RH_Amygdala	ICC3	0.634	18.32	26	234	8.63E-43	0.5209	0.7533	v5.3	HNU
RH_Amygdala	ICC2	0.655	5.74	26	26	1.48E-05	0.3929	0.8115	v5.3 vs. v6.0	HNU
RH_Amygdala	ICC2	0.648	5.99	26	26	9.96E-06	0.3491	0.8131	v5.3 vs. v7.1	HNU
RH_Amygdala	ICC3	0.778	36.11	26	234	2.29E-67	0.6914	0.8595	v6.0	HNU
RH_Amygdala	ICC2	0.832	10.57	26	26	2.88E-08	0.6990	0.9094	v6.0 vs. v7.1	HNU
RH_Amygdala	ICC3	0.699	24.18	26	234	3.04E-52	0.5947	0.8025	v7.1	HNU
RH_bankssts_surfavg	ICC3	0.883	76.73	26	234	2.06E-99	0.8297	0.9291	v5.3	HNU

RH_bankssts_surfavg	ICC2	0.621	8.07	26	26	5.08E-07	0.0908	0.8297	v5.3 vs. v6.0	HNU
RH_bankssts_surfavg	ICC2	0.578	5.22	26	26	3.61E-05	0.2082	0.7782	v5.3 vs. v7.1	HNU
RH_bankssts_surfavg	ICC3	0.890	82.17	26	234	1.67E-102	0.8394	0.9335	v6.0	HNU
RH_bankssts_surfavg	ICC2	0.921	24.69	26	26	1.58E-12	0.8543	0.9582	v6.0 vs. v7.1	HNU
RH_bankssts_surfavg	ICC3	0.894	85.21	26	234	3.75E-104	0.8443	0.9358	v7.1	HNU
RH_bankssts_thickavg	ICC3	0.750	30.94	26	234	2.41E-61	0.6558	0.8394	v5.3	HNU
RH_bankssts_thickavg	ICC2	0.810	10.46	26	26	3.22E-08	0.6575	0.8979	v5.3 vs. v6.0	HNU
RH_bankssts_thickavg	ICC2	0.782	7.90	26	26	6.32E-07	0.6165	0.8809	v5.3 vs. v7.1	HNU
RH_bankssts_thickavg	ICC3	0.802	41.51	26	234	5.75E-73	0.7214	0.8757	v6.0	HNU
RH_bankssts_thickavg	ICC2	0.883	21.15	26	26	1.01E-11	0.7280	0.9442	v6.0 vs. v7.1	HNU
RH_bankssts_thickavg	ICC3	0.772	34.89	26	234	5.31E-66	0.6836	0.8552	v7.1	HNU
RH_caudalanteriorcingulate_surfavg	ICC3	0.958	227.53	26	234	1.32E-150	0.9361	0.9750	v5.3	HNU
RH_caudalanteriorcingulate_surfavg	ICC2	0.899	51.78	26	26	1.71E-16	0.3286	0.9665	v5.3 vs. v6.0	HNU
RH_caudalanteriorcingulate_surfavg	ICC2	0.941	57.01	26	26	5.09E-17	0.7579	0.9768	v5.3 vs. v7.1	HNU
RH_caudalanteriorcingulate_surfavg	ICC3	0.966	282.91	26	234	2.94E-161	0.9480	0.9798	v6.0	HNU
RH_caudalanteriorcingulate_surfavg	ICC2	0.965	73.63	26	26	2.01E-18	0.9091	0.9837	v6.0 vs. v7.1	HNU
RH_caudalanteriorcingulate_surfavg	ICC3	0.970	327.18	26	234	2.06E-168	0.9548	0.9825	v7.1	HNU
RH_caudalanteriorcingulate_thickavg	ICC3	0.788	38.19	26	234	1.34E-69	0.7037	0.8662	v5.3	HNU
RH_caudalanteriorcingulate_thickavg	ICC2	0.870	18.28	26	26	5.67E-11	0.7142	0.9363	v5.3 vs. v6.0	HNU
RH_caudalanteriorcingulate_thickavg	ICC2	0.836	12.03	26	26	6.88E-09	0.7042	0.9120	v5.3 vs. v7.1	HNU
RH_caudalanteriorcingulate_thickavg	ICC3	0.844	55.05	26	234	8.38E-85	0.7761	0.9036	v6.0	HNU
RH_caudalanteriorcingulate_thickavg	ICC2	0.814	25.38	26	26	1.13E-12	0.1657	0.9338	v6.0 vs. v7.1	HNU
RH_caudalanteriorcingulate_thickavg	ICC3	0.855	59.93	26	234	1.77E-88	0.7910	0.9108	v7.1	HNU
RH_caudalmiddlefrontal_surfavg	ICC3	0.966	280.92	26	234	6.54E-161	0.9477	0.9797	v5.3	HNU
RH_caudalmiddlefrontal_surfavg	ICC2	0.963	51.20	26	26	1.96E-16	0.9298	0.9806	v5.3 vs. v6.0	HNU
RH_caudalmiddlefrontal_surfavg	ICC2	0.961	49.05	26	26	3.36E-16	0.9269	0.9798	v5.3 vs. v7.1	HNU
RH_caudalmiddlefrontal_surfavg	ICC3	0.984	602.19	26	234	1.01E-198	0.9750	0.9904	v6.0	HNU
RH_caudalmiddlefrontal_surfavg	ICC2	0.985	127.77	26	26	1.78E-21	0.9712	0.9922	v6.0 vs. v7.1	HNU
RH_caudalmiddlefrontal_surfavg	ICC3	0.978	445.57	26	234	1.05E-183	0.9664	0.9871	v7.1	HNU
RH_caudalmiddlefrontal_thickavg	ICC3	0.834	51.32	26	234	8.20E-82	0.7634	0.8973	v5.3	HNU
RH_caudalmiddlefrontal_thickavg	ICC2	0.721	24.74	26	26	1.54E-12	-0.0147	0.9038	v5.3 vs. v6.0	HNU
RH_caudalmiddlefrontal_thickavg	ICC2	0.737	12.88	26	26	3.21E-09	0.2068	0.8915	v5.3 vs. v7.1	HNU
RH_caudalmiddlefrontal_thickavg	ICC3	0.868	66.84	26	234	2.91E-93	0.8089	0.9194	v6.0	HNU
RH_caudalmiddlefrontal_thickavg	ICC2	0.856	15.05	26	26	5.41E-10	0.7196	0.9257	v6.0 vs. v7.1	HNU
RH_caudalmiddlefrontal_thickavg	ICC3	0.845	55.38	26	234	4.62E-85	0.7772	0.9041	v7.1	HNU
RH_Caudate	ICC3	0.936	147.10	26	234	1.34E-129	0.9042	0.9619	v5.3	HNU
RH_Caudate	ICC2	0.828	51.96	26	26	1.63E-16	0.0270	0.9469	v5.3 vs. v6.0	HNU
RH_Caudate	ICC2	0.888	59.32	26	26	3.09E-17	0.1845	0.9651	v5.3 vs. v7.1	HNU
RH_Caudate	ICC3	0.965	273.51	26	234	1.33E-159	0.9463	0.9792	v6.0	HNU
RH_Caudate	ICC2	0.976	144.00	26	26	3.85E-22	0.8854	0.9907	v6.0 vs. v7.1	HNU
RH_Caudate	ICC3	0.968	301.50	26	234	2.19E-164	0.9511	0.9811	v7.1	HNU
RH_cuneus_surfavg	ICC3	0.935	145.94	26	234	3.21E-129	0.9035	0.9616	v5.3	HNU
RH_cuneus_surfavg	ICC2	0.884	25.61	26	26	1.01E-12	0.6430	0.9507	v5.3 vs. v6.0	HNU
RH_cuneus_surfavg	ICC2	0.761	24.13	26	26	2.08E-12	0.0328	0.9171	v5.3 vs. v7.1	HNU

RH_cuneus_surfavg	ICC3	0.964	269.90	26	234	5.98E-159	0.9456	0.9789	v6.0	HNU
RH_cuneus_surfavg	ICC2	0.923	73.93	26	26	1.91E-18	0.3746	0.9754	v6.0 vs. v7.1	HNU
RH_cuneus_surfavg	ICC3	0.949	186.73	26	234	4.98E-141	0.9231	0.9697	v7.1	HNU
RH_cuneus_thickavg	ICC3	0.750	31.08	26	234	1.63E-61	0.6568	0.8400	v5.3	HNU
RH_cuneus_thickavg	ICC2	0.696	11.96	26	26	7.34E-09	0.1067	0.8759	v5.3 vs. v6.0	HNU
RH_cuneus_thickavg	ICC2	0.747	8.43	26	26	3.24E-07	0.5170	0.8674	v5.3 vs. v7.1	HNU
RH_cuneus_thickavg	ICC3	0.754	31.67	26	234	3.04E-62	0.6613	0.8426	v6.0	HNU
RH_cuneus_thickavg	ICC2	0.793	10.54	26	26	2.97E-08	0.5925	0.8930	v6.0 vs. v7.1	HNU
RH_cuneus_thickavg	ICC3	0.773	34.96	26	234	4.44E-66	0.6841	0.8554	v7.1	HNU
RH_entorhinal_surfavg	ICC3	0.704	24.74	26	234	4.64E-53	0.6006	0.8061	v5.3	HNU
RH_entorhinal_surfavg	ICC2	0.408	4.08	26	26	3.10E-04	-0.0187	0.6808	v5.3 vs. v6.0	HNU
RH_entorhinal_surfavg	ICC2	0.459	4.08	26	26	3.12E-04	0.0544	0.7063	v5.3 vs. v7.1	HNU
RH_entorhinal_surfavg	ICC3	0.758	32.32	26	234	5.11E-63	0.6660	0.8452	v6.0	HNU
RH_entorhinal_surfavg	ICC2	0.888	20.35	26	26	1.59E-11	0.7682	0.9438	v6.0 vs. v7.1	HNU
RH_entorhinal_surfavg	ICC3	0.756	32.06	26	234	1.03E-62	0.6642	0.8442	v7.1	HNU
RH_entorhinal_thickavg	ICC3	0.546	13.03	26	234	1.71E-32	0.4267	0.6817	v5.3	HNU
RH_entorhinal_thickavg	ICC2	0.708	5.93	26	26	1.10E-05	0.5074	0.8361	v5.3 vs. v6.0	HNU
RH_entorhinal_thickavg	ICC2	0.423	3.86	26	26	4.98E-04	0.0169	0.6828	v5.3 vs. v7.1	HNU
RH_entorhinal_thickavg	ICC3	0.542	12.83	26	234	4.53E-32	0.4225	0.6783	v6.0	HNU
RH_entorhinal_thickavg	ICC2	0.469	4.37	26	26	1.76E-04	0.0448	0.7181	v6.0 vs. v7.1	HNU
RH_entorhinal_thickavg	ICC3	0.584	15.06	26	234	1.10E-36	0.4669	0.7136	v7.1	HNU
RH_frontalpole_surfavg	ICC3	0.647	19.30	26	234	1.72E-44	0.5350	0.7631	v5.3	HNU
RH_frontalpole_surfavg	ICC2	0.320	2.58	26	26	9.32E-03	0.0005	0.5783	v5.3 vs. v6.0	HNU
RH_frontalpole_surfavg	ICC2	0.325	6.71	26	26	3.31E-06	-0.0602	0.6470	v5.3 vs. v7.1	HNU
RH_frontalpole_surfavg	ICC3	0.704	24.78	26	234	4.09E-53	0.6009	0.8064	v6.0	HNU
RH_frontalpole_surfavg	ICC2	0.589	7.83	26	26	6.90E-07	0.0366	0.8161	v6.0 vs. v7.1	HNU
RH_frontalpole_surfavg	ICC3	0.775	35.35	26	234	1.60E-66	0.6866	0.8568	v7.1	HNU
RH_frontalpole_thickavg	ICC3	0.728	27.77	26	234	2.84E-57	0.6296	0.8239	v5.3	HNU
RH_frontalpole_thickavg	ICC2	0.733	6.28	26	26	6.31E-06	0.5394	0.8523	v5.3 vs. v6.0	HNU
RH_frontalpole_thickavg	ICC2	0.698	5.46	26	26	2.38E-05	0.4872	0.8317	v5.3 vs. v7.1	HNU
RH_frontalpole_thickavg	ICC3	0.754	31.68	26	234	3.02E-62	0.6613	0.8426	v6.0	HNU
RH_frontalpole_thickavg	ICC2	0.862	13.11	26	26	2.61E-09	0.7499	0.9260	v6.0 vs. v7.1	HNU
RH_frontalpole_thickavg	ICC3	0.807	42.76	26	234	3.50E-74	0.7276	0.8789	v7.1	HNU
RH_fusiform_surfavg	ICC3	0.937	148.51	26	234	4.73E-130	0.9050	0.9622	v5.3	HNU
RH_fusiform_surfavg	ICC2	0.771	21.61	26	26	7.79E-12	0.0829	0.9180	v5.3 vs. v6.0	HNU
RH_fusiform_surfavg	ICC2	0.762	16.10	26	26	2.49E-10	0.1801	0.9073	v5.3 vs. v7.1	HNU
RH_fusiform_surfavg	ICC3	0.969	309.93	26	234	9.62E-166	0.9523	0.9816	v6.0	HNU
RH_fusiform_surfavg	ICC2	0.979	91.23	26	26	1.32E-19	0.9599	0.9890	v6.0 vs. v7.1	HNU
RH_fusiform_surfavg	ICC3	0.964	266.11	26	234	2.96E-158	0.9449	0.9786	v7.1	HNU
RH_fusiform_thickavg	ICC3	0.601	16.04	26	234	1.37E-38	0.4843	0.7268	v5.3	HNU
RH_fusiform_thickavg	ICC2	0.715	11.25	26	26	1.45E-08	0.1999	0.8786	v5.3 vs. v6.0	HNU
RH_fusiform_thickavg	ICC2	0.677	5.28	26	26	3.25E-05	0.4634	0.8178	v5.3 vs. v7.1	HNU
RH_fusiform_thickavg	ICC3	0.740	29.46	26	234	1.74E-59	0.6440	0.8325	v6.0	HNU
RH_fusiform_thickavg	ICC2	0.737	10.12	26	26	4.59E-08	0.3659	0.8783	v6.0 vs. v7.1	HNU

RH_fusiform_thickavg	ICC3	0.718	26.41	26	234	2.00E-55	0.6171	0.8164	v7.1	HNU
RH_Hippocampus	ICC3	0.735	28.74	26	234	1.51E-58	0.6380	0.8289	v5.3	HNU
RH_Hippocampus	ICC2	0.785	8.88	26	26	1.87E-07	0.6213	0.8826	v5.3 vs. v6.0	HNU
RH_Hippocampus	ICC2	0.746	11.32	26	26	1.35E-08	0.3307	0.8873	v5.3 vs. v7.1	HNU
RH_Hippocampus	ICC3	0.864	64.63	26	234	8.80E-92	0.8035	0.9168	v6.0	HNU
RH_Hippocampus	ICC2	0.897	31.05	26	26	9.69E-14	0.6402	0.9582	v6.0 vs. v7.1	HNU
RH_Hippocampus	ICC3	0.869	67.63	26	234	8.85E-94	0.8107	0.9202	v7.1	HNU
RH_inferiorparietal_surfavg	ICC3	0.969	313.51	26	234	2.62E-166	0.9529	0.9818	v5.3	HNU
RH_inferiorparietal_surfavg	ICC2	0.920	23.72	26	26	2.55E-12	0.8528	0.9578	v5.3 vs. v6.0	HNU
RH_inferiorparietal_surfavg	ICC2	0.916	24.38	26	26	1.83E-12	0.8411	0.9556	v5.3 vs. v7.1	HNU
RH_inferiorparietal_surfavg	ICC3	0.955	211.55	26	234	4.56E-147	0.9316	0.9732	v6.0	HNU
RH_inferiorparietal_surfavg	ICC2	0.968	131.26	26	26	1.26E-21	0.7824	0.9887	v6.0 vs. v7.1	HNU
RH_inferiorparietal_surfavg	ICC3	0.965	274.62	26	234	8.46E-160	0.9465	0.9792	v7.1	HNU
RH_inferiorparietal_thickavg	ICC3	0.734	28.56	26	234	2.53E-58	0.6365	0.8281	v5.3	HNU
RH_inferiorparietal_thickavg	ICC2	0.605	9.24	26	26	1.22E-07	0.0121	0.8315	v5.3 vs. v6.0	HNU
RH_inferiorparietal_thickavg	ICC2	0.762	8.78	26	26	2.11E-07	0.5535	0.8741	v5.3 vs. v7.1	HNU
RH_inferiorparietal_thickavg	ICC3	0.725	27.37	26	234	9.73E-57	0.6260	0.8218	v6.0	HNU
RH_inferiorparietal_thickavg	ICC2	0.737	11.73	26	26	9.12E-09	0.2658	0.8870	v6.0 vs. v7.1	HNU
RH_inferiorparietal_thickavg	ICC3	0.713	25.88	26	234	1.11E-54	0.6120	0.8132	v7.1	HNU
RH_inferiortemporal_surfavg	ICC3	0.933	140.06	26	234	2.88E-127	0.8998	0.9600	v5.3	HNU
RH_inferiortemporal_surfavg	ICC2	0.883	28.15	26	26	3.22E-13	0.5780	0.9531	v5.3 vs. v6.0	HNU
RH_inferiortemporal_surfavg	ICC2	0.887	30.50	26	26	1.21E-13	0.5538	0.9556	v5.3 vs. v7.1	HNU
RH_inferiortemporal_surfavg	ICC3	0.939	155.62	26	234	2.80E-132	0.9090	0.9639	v6.0	HNU
RH_inferiortemporal_surfavg	ICC2	0.984	120.44	26	26	3.80E-21	0.9696	0.9917	v6.0 vs. v7.1	HNU
RH_inferiortemporal_surfavg	ICC3	0.963	260.67	26	234	3.03E-157	0.9438	0.9781	v7.1	HNU
RH_inferiortemporal_thickavg	ICC3	0.590	15.40	26	234	2.37E-37	0.4731	0.7183	v5.3	HNU
RH_inferiortemporal_thickavg	ICC2	0.732	8.62	26	26	2.55E-07	0.4448	0.8655	v5.3 vs. v6.0	HNU
RH_inferiortemporal_thickavg	ICC2	0.754	7.00	26	26	2.16E-06	0.5745	0.8640	v5.3 vs. v7.1	HNU
RH_inferiortemporal_thickavg	ICC3	0.699	24.18	26	234	3.11E-52	0.5946	0.8024	v6.0	HNU
RH_inferiortemporal_thickavg	ICC2	0.737	13.46	26	26	1.94E-09	0.1793	0.8932	v6.0 vs. v7.1	HNU
RH_inferiortemporal_thickavg	ICC3	0.670	21.33	26	234	7.53E-48	0.5618	0.7812	v7.1	HNU
RH_insula_surfavg	ICC3	0.770	34.42	26	234	1.78E-65	0.6806	0.8535	v5.3	HNU
RH_insula_surfavg	ICC2	0.604	7.96	26	26	5.82E-07	0.0579	0.8226	v5.3 vs. v6.0	HNU
RH_insula_surfavg	ICC2	0.546	5.62	26	26	1.84E-05	0.0867	0.7743	v5.3 vs. v7.1	HNU
RH_insula_surfavg	ICC3	0.764	33.41	26	234	2.62E-64	0.6738	0.8496	v6.0	HNU
RH_insula_surfavg	ICC2	0.786	8.08	26	26	5.05E-07	0.6230	0.8830	v6.0 vs. v7.1	HNU
RH_insula_surfavg	ICC3	0.812	44.06	26	234	2.03E-75	0.7337	0.8821	v7.1	HNU
RH_insula_thickavg	ICC3	0.495	10.82	26	234	1.57E-27	0.3754	0.6379	v5.3	HNU
RH_insula_thickavg	ICC2	0.407	2.60	26	26	8.89E-03	0.1197	0.6354	v5.3 vs. v6.0	HNU
RH_insula_thickavg	ICC2	0.608	3.99	26	26	3.75E-04	0.3569	0.7765	v5.3 vs. v7.1	HNU
RH_insula_thickavg	ICC3	0.506	11.24	26	234	1.60E-28	0.3860	0.6472	v6.0	HNU
RH_insula_thickavg	ICC2	0.620	5.66	26	26	1.70E-05	0.2881	0.7994	v6.0 vs. v7.1	HNU
RH_insula_thickavg	ICC3	0.649	19.52	26	234	7.09E-45	0.5382	0.7653	v7.1	HNU
RH_isthmuscingulate_surfavg	ICC3	0.907	99.04	26	234	4.82E-111	0.8634	0.9443	v5.3	HNU

RH_isthmuscingulate_surfavg	ICC2	0.837	13.70	26	26	1.60E-09	0.6705	0.9171	v5.3 vs. v6.0	HNU
RH_isthmuscingulate_surfavg	ICC2	0.855	13.23	26	26	2.37E-09	0.7403	0.9221	v5.3 vs. v7.1	HNU
RH_isthmuscingulate_surfavg	ICC3	0.962	252.83	26	234	9.42E-156	0.9421	0.9775	v6.0	HNU
RH_isthmuscingulate_surfavg	ICC2	0.965	80.44	26	26	6.53E-19	0.8946	0.9847	v6.0 vs. v7.1	HNU
RH_isthmuscingulate_surfavg	ICC3	0.955	214.90	26	234	7.87E-148	0.9326	0.9736	v7.1	HNU
RH_isthmuscingulate_thickavg	ICC3	0.850	57.65	26	234	8.48E-87	0.7843	0.9076	v5.3	HNU
RH_isthmuscingulate_thickavg	ICC2	0.759	7.81	26	26	7.10E-07	0.5801	0.8674	v5.3 vs. v6.0	HNU
RH_isthmuscingulate_thickavg	ICC2	0.571	6.23	26	26	6.84E-06	0.0940	0.7927	v5.3 vs. v7.1	HNU
RH_isthmuscingulate_thickavg	ICC3	0.837	52.42	26	234	1.04E-82	0.7673	0.8992	v6.0	HNU
RH_isthmuscingulate_thickavg	ICC2	0.755	14.95	26	26	5.85E-10	0.1913	0.9026	v6.0 vs. v7.1	HNU
RH_isthmuscingulate_thickavg	ICC3	0.862	63.36	26	234	6.57E-91	0.8002	0.9153	v7.1	HNU
RH_lateraloccipital_surfavg	ICC3	0.946	176.37	26	234	2.79E-138	0.9189	0.9680	v5.3	HNU
RH_lateraloccipital_surfavg	ICC2	0.738	29.11	26	26	2.14E-13	-0.0134	0.9120	v5.3 vs. v6.0	HNU
RH_lateraloccipital_surfavg	ICC2	0.609	32.83	26	26	4.91E-14	-0.0286	0.8606	v5.3 vs. v7.1	HNU
RH_lateraloccipital_surfavg	ICC3	0.963	261.33	26	234	2.28E-157	0.9439	0.9782	v6.0	HNU
RH_lateraloccipital_surfavg	ICC2	0.946	129.63	26	26	1.48E-21	0.3772	0.9838	v6.0 vs. v7.1	HNU
RH_lateraloccipital_surfavg	ICC3	0.951	194.77	26	234	4.60E-143	0.9260	0.9709	v7.1	HNU
RH_lateraloccipital_thickavg	ICC3	0.792	39.12	26	234	1.46E-70	0.7089	0.8690	v5.3	HNU
RH_lateraloccipital_thickavg	ICC2	0.656	11.47	26	26	1.17E-08	0.0327	0.8605	v5.3 vs. v6.0	HNU
RH_lateraloccipital_thickavg	ICC2	0.699	7.07	26	26	1.96E-06	0.4324	0.8417	v5.3 vs. v7.1	HNU
RH_lateraloccipital_thickavg	ICC3	0.850	57.61	26	234	9.09E-87	0.7842	0.9075	v6.0	HNU
RH_lateraloccipital_thickavg	ICC2	0.817	14.02	26	26	1.22E-09	0.5616	0.9144	v6.0 vs. v7.1	HNU
RH_lateraloccipital_thickavg	ICC3	0.870	68.01	26	234	5.00E-94	0.8116	0.9207	v7.1	HNU
RH_lateralorbitofrontal_surfavg	ICC3	0.790	38.56	26	234	5.53E-70	0.7058	0.8673	v5.3	HNU
RH_lateralorbitofrontal_surfavg	ICC2	0.896	20.44	26	26	1.52E-11	0.8012	0.9460	v5.3 vs. v6.0	HNU
RH_lateralorbitofrontal_surfavg	ICC2	0.649	8.49	26	26	2.98E-07	0.1413	0.8425	v5.3 vs. v7.1	HNU
RH_lateralorbitofrontal_surfavg	ICC3	0.814	44.67	26	234	5.51E-76	0.7365	0.8835	v6.0	HNU
RH_lateralorbitofrontal_surfavg	ICC2	0.775	14.96	26	26	5.80E-10	0.2804	0.9079	v6.0 vs. v7.1	HNU
RH_lateralorbitofrontal_surfavg	ICC3	0.762	32.93	26	234	9.60E-64	0.6704	0.8477	v7.1	HNU
RH_lateralorbitofrontal_thickavg	ICC3	0.588	15.24	26	234	4.71E-37	0.4703	0.7162	v5.3	HNU
RH_lateralorbitofrontal_thickavg	ICC2	0.698	11.57	26	26	1.07E-08	0.1286	0.8751	v5.3 vs. v6.0	HNU
RH_lateralorbitofrontal_thickavg	ICC2	0.728	6.41	26	26	5.14E-06	0.5378	0.8485	v5.3 vs. v7.1	HNU
RH_lateralorbitofrontal_thickavg	ICC3	0.622	17.47	26	234	2.94E-41	0.5078	0.7440	v6.0	HNU
RH_lateralorbitofrontal_thickavg	ICC2	0.655	7.09	26	26	1.90E-06	0.2678	0.8304	v6.0 vs. v7.1	HNU
RH_lateralorbitofrontal_thickavg	ICC3	0.513	11.54	26	234	3.34E-29	0.3931	0.6535	v7.1	HNU
RH_LateralVentricle	ICC3	0.995	2144.57	26	234	1.16E-262	0.9929	0.9973	v5.3	HNU
RH_LateralVentricle	ICC2	0.987	881.39	26	26	2.61E-32	0.5902	0.9965	v5.3 vs. v6.0	HNU
RH_LateralVentricle	ICC2	0.988	1314.01	26	26	1.47E-34	0.5209	0.9970	v5.3 vs. v7.1	HNU
RH_LateralVentricle	ICC3	0.997	3242.50	26	234	1.37E-283	0.9953	0.9982	v6.0	HNU
RH_LateralVentricle	ICC2	0.999	1941.83	26	26	9.20E-37	0.9981	0.9995	v6.0 vs. v7.1	HNU
RH_LateralVentricle	ICC3	0.997	3610.70	26	234	4.86E-289	0.9957	0.9984	v7.1	HNU
RH_lingual_surfavg	ICC3	0.878	73.13	26	234	2.94E-97	0.8227	0.9258	v5.3	HNU
RH_lingual_surfavg	ICC2	0.857	16.48	26	26	1.90E-10	0.6903	0.9296	v5.3 vs. v6.0	HNU
RH_lingual_surfavg	ICC2	0.697	10.34	26	26	3.63E-08	0.1829	0.8688	v5.3 vs. v7.1	HNU

RH_lingual_surfavg	ICC3	0.931	136.38	26	234	5.25E-126	0.8973	0.9590	v6.0	HNU
RH_lingual_surfavg	ICC2	0.885	32.10	26	26	6.46E-14	0.5059	0.9564	v6.0 vs. v7.1	HNU
RH_lingual_surfavg	ICC3	0.940	158.50	26	234	3.71E-133	0.9105	0.9645	v7.1	HNU
RH_lingual_thickavg	ICC3	0.701	24.42	26	234	1.37E-52	0.5972	0.8040	v5.3	HNU
RH_lingual_thickavg	ICC2	0.859	22.16	26	26	5.78E-12	0.5428	0.9416	v5.3 vs. v6.0	HNU
RH_lingual_thickavg	ICC2	0.877	16.08	26	26	2.53E-10	0.7740	0.9341	v5.3 vs. v7.1	HNU
RH_lingual_thickavg	ICC3	0.737	28.99	26	234	7.02E-59	0.6401	0.8302	v6.0	HNU
RH_lingual_thickavg	ICC2	0.802	21.08	26	26	1.05E-11	0.2079	0.9264	v6.0 vs. v7.1	HNU
RH_lingual_thickavg	ICC3	0.779	36.21	26	234	1.78E-67	0.6920	0.8598	v7.1	HNU
RH_medialorbitofrontal_surfavg	ICC3	0.791	38.84	26	234	2.84E-70	0.7073	0.8681	v5.3	HNU
RH_medialorbitofrontal_surfavg	ICC2	0.751	11.58	26	26	1.05E-08	0.3374	0.8897	v5.3 vs. v6.0	HNU
RH_medialorbitofrontal_surfavg	ICC2	0.345	7.38	26	26	1.27E-06	-0.0591	0.6678	v5.3 vs. v7.1	HNU
RH_medialorbitofrontal_surfavg	ICC3	0.805	42.24	26	234	1.10E-73	0.7251	0.8776	v6.0	HNU
RH_medialorbitofrontal_surfavg	ICC2	0.609	11.97	26	26	7.28E-09	-0.0301	0.8433	v6.0 vs. v7.1	HNU
RH_medialorbitofrontal_surfavg	ICC3	0.783	37.05	26	234	2.22E-68	0.6971	0.8626	v7.1	HNU
RH_medialorbitofrontal_thickavg	ICC3	0.525	12.06	26	234	2.20E-30	0.4054	0.6640	v5.3	HNU
RH_medialorbitofrontal_thickavg	ICC2	0.640	9.70	26	26	7.32E-08	0.0577	0.8471	v5.3 vs. v6.0	HNU
RH_medialorbitofrontal_thickavg	ICC2	0.775	7.95	26	26	5.92E-07	0.6096	0.8760	v5.3 vs. v7.1	HNU
RH_medialorbitofrontal_thickavg	ICC3	0.641	18.88	26	234	9.00E-44	0.5291	0.7590	v6.0	HNU
RH_medialorbitofrontal_thickavg	ICC2	0.635	8.39	26	26	3.40E-07	0.1089	0.8371	v6.0 vs. v7.1	HNU
RH_medialorbitofrontal_thickavg	ICC3	0.577	14.65	26	234	7.28E-36	0.4592	0.7077	v7.1	HNU
RH_middletemporal_surfavg	ICC3	0.966	285.51	26	234	1.04E-161	0.9485	0.9800	v5.3	HNU
RH_middletemporal_surfavg	ICC2	0.894	31.15	26	26	9.35E-14	0.6115	0.9577	v5.3 vs. v6.0	HNU
RH_middletemporal_surfavg	ICC2	0.807	21.38	26	26	8.87E-12	0.2261	0.9282	v5.3 vs. v7.1	HNU
RH_middletemporal_surfavg	ICC3	0.980	488.48	26	234	2.80E-188	0.9693	0.9882	v6.0	HNU
RH_middletemporal_surfavg	ICC2	0.966	140.07	26	26	5.48E-22	0.7306	0.9888	v6.0 vs. v7.1	HNU
RH_middletemporal_surfavg	ICC3	0.984	615.39	26	234	8.33E-200	0.9755	0.9906	v7.1	HNU
RH_middletemporal_thickavg	ICC3	0.652	19.75	26	234	2.91E-45	0.5413	0.7674	v5.3	HNU
RH_middletemporal_thickavg	ICC2	0.815	19.25	26	26	3.07E-11	0.3312	0.9271	v5.3 vs. v6.0	HNU
RH_middletemporal_thickavg	ICC2	0.762	7.82	26	26	7.07E-07	0.5873	0.8689	v5.3 vs. v7.1	HNU
RH_middletemporal_thickavg	ICC3	0.762	33.03	26	234	7.27E-64	0.6711	0.8481	v6.0	HNU
RH_middletemporal_thickavg	ICC2	0.634	11.44	26	26	1.21E-08	0.0006	0.8521	v6.0 vs. v7.1	HNU
RH_middletemporal_thickavg	ICC3	0.696	23.86	26	234	9.09E-52	0.5912	0.8003	v7.1	HNU
RH_Pallidum	ICC3	0.706	25.03	26	234	1.79E-53	0.6035	0.8080	v5.3	HNU
RH_Pallidum	ICC2	0.642	4.57	26	26	1.20E-04	0.4110	0.7967	v5.3 vs. v6.0	HNU
RH_Pallidum	ICC2	0.475	3.35	26	26	1.51E-03	0.1738	0.6911	v5.3 vs. v7.1	HNU
RH_Pallidum	ICC3	0.848	56.74	26	234	4.18E-86	0.7815	0.9062	v6.0	HNU
RH_Pallidum	ICC2	0.842	23.44	26	26	2.94E-12	0.3738	0.9393	v6.0 vs. v7.1	HNU
RH_Pallidum	ICC3	0.841	53.74	26	234	8.95E-84	0.7718	0.9015	v7.1	HNU
RH_paracentral_surfavg	ICC3	0.950	189.06	26	234	1.25E-141	0.9240	0.9701	v5.3	HNU
RH_paracentral_surfavg	ICC2	0.915	22.28	26	26	5.40E-12	0.8433	0.9549	v5.3 vs. v6.0	HNU
RH_paracentral_surfavg	ICC2	0.931	27.06	26	26	5.19E-13	0.8708	0.9635	v5.3 vs. v7.1	HNU
RH_paracentral_surfavg	ICC3	0.974	379.24	26	234	1.04E-175	0.9607	0.9849	v6.0	HNU
RH_paracentral_surfavg	ICC2	0.975	78.21	26	26	9.34E-19	0.9527	0.9869	v6.0 vs. v7.1	HNU

RH_paracentral_surfavg	ICC3	0.972	350.72	26	234	7.67E-172	0.9577	0.9837	v7.1	HNU
RH_paracentral_thickavg	ICC3	0.778	36.13	26	234	2.21E-67	0.6915	0.8595	v5.3	HNU
RH_paracentral_thickavg	ICC2	0.601	21.40	26	26	8.74E-12	-0.0406	0.8523	v5.3 vs. v6.0	HNU
RH_paracentral_thickavg	ICC2	0.592	15.62	26	26	3.53E-10	-0.0465	0.8429	v5.3 vs. v7.1	HNU
RH_paracentral_thickavg	ICC3	0.751	31.11	26	234	1.47E-61	0.6571	0.8401	v6.0	HNU
RH_paracentral_thickavg	ICC2	0.840	11.13	26	26	1.63E-08	0.7120	0.9137	v6.0 vs. v7.1	HNU
RH_paracentral_thickavg	ICC3	0.759	32.43	26	234	3.75E-63	0.6668	0.8457	v7.1	HNU
RH_parahippocampal_surfavg	ICC3	0.865	64.95	26	234	5.32E-92	0.8043	0.9172	v5.3	HNU
RH_parahippocampal_surfavg	ICC2	0.568	6.12	26	26	8.15E-06	0.0971	0.7903	v5.3 vs. v6.0	HNU
RH_parahippocampal_surfavg	ICC2	0.678	12.68	26	26	3.80E-09	0.0411	0.8720	v5.3 vs. v7.1	HNU
RH_parahippocampal_surfavg	ICC3	0.895	86.24	26	234	1.07E-104	0.8459	0.9365	v6.0	HNU
RH_parahippocampal_surfavg	ICC2	0.909	20.21	26	26	1.73E-11	0.8311	0.9517	v6.0 vs. v7.1	HNU
RH_parahippocampal_surfavg	ICC3	0.915	109.30	26	234	1.30E-115	0.8747	0.9493	v7.1	HNU
RH_parahippocampal_thickavg	ICC3	0.806	42.61	26	234	4.85E-74	0.7269	0.8785	v5.3	HNU
RH_parahippocampal_thickavg	ICC2	0.888	28.15	26	26	3.22E-13	0.6180	0.9540	v5.3 vs. v6.0	HNU
RH_parahippocampal_thickavg	ICC2	0.870	16.55	26	26	1.81E-10	0.7458	0.9325	v5.3 vs. v7.1	HNU
RH_parahippocampal_thickavg	ICC3	0.838	52.84	26	234	4.73E-83	0.7687	0.8999	v6.0	HNU
RH_parahippocampal_thickavg	ICC2	0.814	44.53	26	26	1.12E-15	0.0237	0.9415	v6.0 vs. v7.1	HNU
RH_parahippocampal_thickavg	ICC3	0.814	44.73	26	234	4.79E-76	0.7368	0.8837	v7.1	HNU
RH_parsopercularis_surfavg	ICC3	0.967	298.11	26	234	7.89E-164	0.9505	0.9808	v5.3	HNU
RH_parsopercularis_surfavg	ICC2	0.854	12.76	26	26	3.55E-09	0.7389	0.9215	v5.3 vs. v6.0	HNU
RH_parsopercularis_surfavg	ICC2	0.813	9.58	26	26	8.33E-08	0.6701	0.8985	v5.3 vs. v7.1	HNU
RH_parsopercularis_surfavg	ICC3	0.982	534.84	26	234	8.46E-193	0.9719	0.9892	v6.0	HNU
RH_parsopercularis_surfavg	ICC2	0.979	89.96	26	26	1.58E-19	0.9594	0.9889	v6.0 vs. v7.1	HNU
RH_parsopercularis_surfavg	ICC3	0.984	610.11	26	234	2.25E-199	0.9753	0.9906	v7.1	HNU
RH_parsopercularis_thickavg	ICC3	0.751	31.16	26	234	1.28E-61	0.6575	0.8403	v5.3	HNU
RH_parsopercularis_thickavg	ICC2	0.648	13.29	26	26	2.25E-09	-0.0112	0.8625	v5.3 vs. v6.0	HNU
RH_parsopercularis_thickavg	ICC2	0.636	10.64	26	26	2.66E-08	0.0207	0.8500	v5.3 vs. v7.1	HNU
RH_parsopercularis_thickavg	ICC3	0.828	49.04	26	234	6.88E-80	0.7548	0.8929	v6.0	HNU
RH_parsopercularis_thickavg	ICC2	0.850	12.26	26	26	5.58E-09	0.7308	0.9189	v6.0 vs. v7.1	HNU
RH_parsopercularis_thickavg	ICC3	0.773	34.96	26	234	4.43E-66	0.6841	0.8554	v7.1	HNU
RH_parsorbitalis_surfavg	ICC3	0.933	140.71	26	234	1.74E-127	0.9002	0.9602	v5.3	HNU
RH_parsorbitalis_surfavg	ICC2	0.819	23.98	26	26	2.24E-12	0.2227	0.9342	v5.3 vs. v6.0	HNU
RH_parsorbitalis_surfavg	ICC2	0.733	24.89	26	26	1.43E-12	-0.0065	0.9080	v5.3 vs. v7.1	HNU
RH_parsorbitalis_surfavg	ICC3	0.967	294.71	26	234	2.89E-163	0.9500	0.9806	v6.0	HNU
RH_parsorbitalis_surfavg	ICC2	0.951	86.91	26	26	2.45E-19	0.6904	0.9829	v6.0 vs. v7.1	HNU
RH_parsorbitalis_surfavg	ICC3	0.976	413.19	26	234	5.86E-180	0.9639	0.9861	v7.1	HNU
RH_parsorbitalis_thickavg	ICC3	0.717	26.38	26	234	2.24E-55	0.6168	0.8162	v5.3	HNU
RH_parsorbitalis_thickavg	ICC2	0.571	10.05	26	26	4.97E-08	-0.0365	0.8207	v5.3 vs. v6.0	HNU
RH_parsorbitalis_thickavg	ICC2	0.516	4.97	26	26	5.68E-05	0.0786	0.7509	v5.3 vs. v7.1	HNU
RH_parsorbitalis_thickavg	ICC3	0.697	24.05	26	234	4.86E-52	0.5932	0.8015	v6.0	HNU
RH_parsorbitalis_thickavg	ICC2	0.727	6.90	26	26	2.51E-06	0.5295	0.8493	v6.0 vs. v7.1	HNU
RH_parsorbitalis_thickavg	ICC3	0.643	19.02	26	234	5.07E-44	0.5312	0.7604	v7.1	HNU
RH_parstriangularis_surfavg	ICC3	0.968	303.26	26	234	1.14E-164	0.9513	0.9812	v5.3	HNU

RH_parstriangularis_surfavg	ICC2	0.882	24.07	26	26	2.14E-12	0.6579	0.9484	v5.3 vs. v6.0	HNU
RH_parstriangularis_surfavg	ICC2	0.824	24.48	26	26	1.74E-12	0.2356	0.9359	v5.3 vs. v7.1	HNU
RH_parstriangularis_surfavg	ICC3	0.980	492.95	26	234	9.84E-189	0.9696	0.9883	v6.0	HNU
RH_parstriangularis_surfavg	ICC2	0.968	141.31	26	26	4.90E-22	0.7519	0.9891	v6.0 vs. v7.1	HNU
RH_parstriangularis_surfavg	ICC3	0.983	567.50	26	234	9.31E-196	0.9735	0.9899	v7.1	HNU
RH_parstriangularis_thickavg	ICC3	0.780	36.37	26	234	1.21E-67	0.6930	0.8603	v5.3	HNU
RH_parstriangularis_thickavg	ICC2	0.551	10.01	26	26	5.21E-08	-0.0466	0.8110	v5.3 vs. v6.0	HNU
RH_parstriangularis_thickavg	ICC2	0.583	6.94	26	26	2.36E-06	0.0711	0.8055	v5.3 vs. v7.1	HNU
RH_parstriangularis_thickavg	ICC3	0.805	42.28	26	234	1.01E-73	0.7253	0.8777	v6.0	HNU
RH_parstriangularis_thickavg	ICC2	0.840	12.13	26	26	6.29E-09	0.7130	0.9138	v6.0 vs. v7.1	HNU
RH_parstriangularis_thickavg	ICC3	0.791	38.95	26	234	2.20E-70	0.7079	0.8685	v7.1	HNU
RH_pericalcarine_surfavg	ICC3	0.875	70.93	26	234	6.72E-96	0.8181	0.9237	v5.3	HNU
RH_pericalcarine_surfavg	ICC2	0.852	21.60	26	26	7.83E-12	0.5060	0.9393	v5.3 vs. v6.0	HNU
RH_pericalcarine_surfavg	ICC2	0.824	18.86	26	26	3.91E-11	0.4036	0.9286	v5.3 vs. v7.1	HNU
RH_pericalcarine_surfavg	ICC3	0.940	158.01	26	234	5.20E-133	0.9102	0.9644	v6.0	HNU
RH_pericalcarine_surfavg	ICC2	0.979	96.34	26	26	6.58E-20	0.9599	0.9890	v6.0 vs. v7.1	HNU
RH_pericalcarine_surfavg	ICC3	0.948	183.18	26	234	4.20E-140	0.9217	0.9692	v7.1	HNU
RH_pericalcarine_thickavg	ICC3	0.725	27.37	26	234	9.67E-57	0.6260	0.8218	v5.3	HNU
RH_pericalcarine_thickavg	ICC2	0.726	12.61	26	26	4.07E-09	0.1763	0.8874	v5.3 vs. v6.0	HNU
RH_pericalcarine_thickavg	ICC2	0.770	10.21	26	26	4.17E-08	0.5095	0.8860	v5.3 vs. v7.1	HNU
RH_pericalcarine_thickavg	ICC3	0.771	34.75	26	234	7.65E-66	0.6827	0.8547	v6.0	HNU
RH_pericalcarine_thickavg	ICC2	0.865	15.74	26	26	3.25E-10	0.7412	0.9296	v6.0 vs. v7.1	HNU
RH_pericalcarine_thickavg	ICC3	0.794	39.63	26	234	4.39E-71	0.7117	0.8705	v7.1	HNU
RH_postcentral_surfavg	ICC3	0.957	226.19	26	234	2.55E-150	0.9357	0.9749	v5.3	HNU
RH_postcentral_surfavg	ICC2	0.949	36.92	26	26	1.15E-14	0.9040	0.9733	v5.3 vs. v6.0	HNU
RH_postcentral_surfavg	ICC2	0.959	56.25	26	26	6.03E-17	0.9122	0.9795	v5.3 vs. v7.1	HNU
RH_postcentral_surfavg	ICC3	0.974	370.58	26	234	1.45E-174	0.9599	0.9845	v6.0	HNU
RH_postcentral_surfavg	ICC2	0.979	144.39	26	26	3.71E-22	0.9243	0.9912	v6.0 vs. v7.1	HNU
RH_postcentral_surfavg	ICC3	0.954	209.12	26	234	1.65E-146	0.9308	0.9729	v7.1	HNU
RH_postcentral_thickavg	ICC3	0.855	60.14	26	234	1.25E-88	0.7915	0.9111	v5.3	HNU
RH_postcentral_thickavg	ICC2	0.534	26.76	26	26	5.96E-13	-0.0300	0.8206	v5.3 vs. v6.0	HNU
RH_postcentral_thickavg	ICC2	0.497	27.20	26	26	4.88E-13	-0.0267	0.7996	v5.3 vs. v7.1	HNU
RH_postcentral_thickavg	ICC3	0.868	66.73	26	234	3.44E-93	0.8086	0.9193	v6.0	HNU
RH_postcentral_thickavg	ICC2	0.950	43.34	26	26	1.58E-15	0.9015	0.9743	v6.0 vs. v7.1	HNU
RH_postcentral_thickavg	ICC3	0.793	39.30	26	234	9.59E-71	0.7099	0.8695	v7.1	HNU
RH_posteriorcingulate_surfavg	ICC3	0.962	256.30	26	234	2.03E-156	0.9429	0.9778	v5.3	HNU
RH_posteriorcingulate_surfavg	ICC2	0.950	42.35	26	26	2.10E-15	0.9042	0.9741	v5.3 vs. v6.0	HNU
RH_posteriorcingulate_surfavg	ICC2	0.934	33.63	26	26	3.64E-14	0.8650	0.9663	v5.3 vs. v7.1	HNU
RH_posteriorcingulate_surfavg	ICC3	0.973	361.37	26	234	2.55E-173	0.9589	0.9842	v6.0	HNU
RH_posteriorcingulate_surfavg	ICC2	0.979	95.62	26	26	7.24E-20	0.9600	0.9890	v6.0 vs. v7.1	HNU
RH_posteriorcingulate_surfavg	ICC3	0.978	455.22	26	234	9.03E-185	0.9671	0.9874	v7.1	HNU
RH_posteriorcingulate_thickavg	ICC3	0.711	25.61	26	234	2.61E-54	0.6094	0.8116	v5.3	HNU
RH_posteriorcingulate_thickavg	ICC2	0.743	7.79	26	26	7.36E-07	0.5375	0.8612	v5.3 vs. v6.0	HNU
RH_posteriorcingulate_thickavg	ICC2	0.618	6.36	26	26	5.60E-06	0.2129	0.8101	v5.3 vs. v7.1	HNU

RH_posteriorcingulate_thickavg	ICC3	0.817	45.65	26	234	6.81E-77	0.7408	0.8858	v6.0	HNU
RH_posteriorcingulate_thickavg	ICC2	0.514	7.29	26	26	1.43E-06	-0.0364	0.7790	v6.0 vs. v7.1	HNU
RH_posteriorcingulate_thickavg	ICC3	0.755	31.79	26	234	2.20E-62	0.6622	0.8430	v7.1	HNU
RH_precentral_surfavg	ICC3	0.969	310.89	26	234	6.78E-166	0.9525	0.9816	v5.3	HNU
RH_precentral_surfavg	ICC2	0.961	52.85	26	26	1.32E-16	0.9255	0.9796	v5.3 vs. v6.0	HNU
RH_precentral_surfavg	ICC2	0.959	46.55	26	26	6.46E-16	0.9224	0.9784	v5.3 vs. v7.1	HNU
RH_precentral_surfavg	ICC3	0.969	318.34	26	234	4.62E-167	0.9536	0.9820	v6.0	HNU
RH_precentral_surfavg	ICC2	0.982	171.98	26	26	3.93E-23	0.9356	0.9926	v6.0 vs. v7.1	HNU
RH_precentral_surfavg	ICC3	0.958	229.84	26	234	4.23E-151	0.9367	0.9753	v7.1	HNU
RH_precentral_thickavg	ICC3	0.739	29.32	26	234	2.65E-59	0.6429	0.8318	v5.3	HNU
RH_precentral_thickavg	ICC2	0.483	11.70	26	26	9.40E-09	-0.0550	0.7789	v5.3 vs. v6.0	HNU
RH_precentral_thickavg	ICC2	0.405	8.97	26	26	1.68E-07	-0.0585	0.7208	v5.3 vs. v7.1	HNU
RH_precentral_thickavg	ICC3	0.775	35.50	26	234	1.10E-66	0.6876	0.8573	v6.0	HNU
RH_precentral_thickavg	ICC2	0.820	10.98	26	26	1.88E-08	0.6755	0.9032	v6.0 vs. v7.1	HNU
RH_precentral_thickavg	ICC3	0.709	25.42	26	234	4.92E-54	0.6074	0.8104	v7.1	HNU
RH_precuneus_surfavg	ICC3	0.977	435.03	26	234	1.63E-182	0.9656	0.9868	v5.3	HNU
RH_precuneus_surfavg	ICC2	0.967	66.87	26	26	6.79E-18	0.9326	0.9831	v5.3 vs. v6.0	HNU
RH_precuneus_surfavg	ICC2	0.964	66.94	26	26	6.71E-18	0.9202	0.9826	v5.3 vs. v7.1	HNU
RH_precuneus_surfavg	ICC3	0.988	791.23	26	234	2.13E-212	0.9808	0.9927	v6.0	HNU
RH_precuneus_surfavg	ICC2	0.993	301.86	26	26	2.78E-26	0.9874	0.9965	v6.0 vs. v7.1	HNU
RH_precuneus_surfavg	ICC3	0.985	665.16	26	234	1.07E-203	0.9773	0.9913	v7.1	HNU
RH_precuneus_thickavg	ICC3	0.777	35.88	26	234	4.20E-67	0.6899	0.8586	v5.3	HNU
RH_precuneus_thickavg	ICC2	0.651	16.58	26	26	1.77E-10	-0.0306	0.8698	v5.3 vs. v6.0	HNU
RH_precuneus_thickavg	ICC2	0.728	13.81	26	26	1.45E-09	0.1355	0.8917	v5.3 vs. v7.1	HNU
RH_precuneus_thickavg	ICC3	0.813	44.58	26	234	6.57E-76	0.7361	0.8834	v6.0	HNU
RH_precuneus_thickavg	ICC2	0.872	19.82	26	26	2.18E-11	0.6910	0.9398	v6.0 vs. v7.1	HNU
RH_precuneus_thickavg	ICC3	0.825	48.14	26	234	4.11E-79	0.7512	0.8911	v7.1	HNU
RH_Putamen	ICC3	0.918	112.52	26	234	5.81E-117	0.8779	0.9507	v5.3	HNU
RH_Putamen	ICC2	0.401	22.79	26	26	4.12E-12	-0.0237	0.7314	v5.3 vs. v6.0	HNU
RH_Putamen	ICC2	0.439	20.18	26	26	1.76E-11	-0.0307	0.7591	v5.3 vs. v7.1	HNU
RH_Putamen	ICC3	0.960	243.08	26	234	7.87E-154	0.9400	0.9766	v6.0	HNU
RH_Putamen	ICC2	0.961	96.30	26	26	6.62E-20	0.7926	0.9856	v6.0 vs. v7.1	HNU
RH_Putamen	ICC3	0.971	341.01	26	234	1.87E-170	0.9565	0.9832	v7.1	HNU
RH_rostralanteriorcingulate_surfavg	ICC3	0.912	104.85	26	234	1.12E-113	0.8700	0.9472	v5.3	HNU
RH_rostralanteriorcingulate_surfavg	ICC2	0.683	11.03	26	26	1.80E-08	0.1076	0.8680	v5.3 vs. v6.0	HNU
RH_rostralanteriorcingulate_surfavg	ICC2	0.798	12.81	26	26	3.40E-09	0.5121	0.9058	v5.3 vs. v7.1	HNU
RH_rostralanteriorcingulate_surfavg	ICC3	0.945	173.37	26	234	1.87E-137	0.9176	0.9675	v6.0	HNU
RH_rostralanteriorcingulate_surfavg	ICC2	0.920	46.24	26	26	7.02E-16	0.6348	0.9701	v6.0 vs. v7.1	HNU
RH_rostralanteriorcingulate_surfavg	ICC3	0.946	177.08	26	234	1.79E-138	0.9192	0.9681	v7.1	HNU
RH_rostralanteriorcingulate_thickavg	ICC3	0.559	13.66	26	234	7.83E-34	0.4398	0.6924	v5.3	HNU
RH_rostralanteriorcingulate_thickavg	ICC2	0.790	16.70	26	26	1.63E-10	0.2890	0.9162	v5.3 vs. v6.0	HNU
RH_rostralanteriorcingulate_thickavg	ICC2	0.852	12.86	26	26	3.24E-09	0.7341	0.9201	v5.3 vs. v7.1	HNU
RH_rostralanteriorcingulate_thickavg	ICC3	0.700	24.31	26	234	1.99E-52	0.5960	0.8033	v6.0	HNU
RH_rostralanteriorcingulate_thickavg	ICC2	0.754	33.25	26	26	4.19E-14	-0.0100	0.9191	v6.0 vs. v7.1	HNU

RH_rostralanteriorcingulate_thickavg	ICC3	0.676	21.82	26	234	1.21E-48	0.5679	0.7852	v7.1	HNU
RH_rostralmiddlefrontal_surfavg	ICC3	0.979	460.42	26	234	2.46E-185	0.9675	0.9875	v5.3	HNU
RH_rostralmiddlefrontal_surfavg	ICC2	0.971	90.31	26	26	1.50E-19	0.9280	0.9868	v5.3 vs. v6.0	HNU
RH_rostralmiddlefrontal_surfavg	ICC2	0.947	73.84	26	26	1.94E-18	0.7094	0.9807	v5.3 vs. v7.1	HNU
RH_rostralmiddlefrontal_surfavg	ICC3	0.990	1027.49	26	234	1.58E-225	0.9852	0.9944	v6.0	HNU
RH_rostralmiddlefrontal_surfavg	ICC2	0.983	171.93	26	26	3.94E-23	0.9422	0.9927	v6.0 vs. v7.1	HNU
RH_rostralmiddlefrontal_surfavg	ICC3	0.986	681.79	26	234	6.21E-205	0.9778	0.9915	v7.1	HNU
RH_rostralmiddlefrontal_thickavg	ICC3	0.787	37.84	26	234	3.13E-69	0.7017	0.8651	v5.3	HNU
RH_rostralmiddlefrontal_thickavg	ICC2	0.644	15.91	26	26	2.86E-10	-0.0319	0.8659	v5.3 vs. v6.0	HNU
RH_rostralmiddlefrontal_thickavg	ICC2	0.580	6.61	26	26	3.82E-06	0.0859	0.8007	v5.3 vs. v7.1	HNU
RH_rostralmiddlefrontal_thickavg	ICC3	0.803	41.88	26	234	2.47E-73	0.7233	0.8767	v6.0	HNU
RH_rostralmiddlefrontal_thickavg	ICC2	0.817	10.54	26	26	2.96E-08	0.6746	0.9010	v6.0 vs. v7.1	HNU
RH_rostralmiddlefrontal_thickavg	ICC3	0.741	29.68	26	234	9.16E-60	0.6458	0.8336	v7.1	HNU
RH_superiorfrontal_surfavg	ICC3	0.986	680.93	26	234	7.18E-205	0.9778	0.9915	v5.3	HNU
RH_superiorfrontal_surfavg	ICC2	0.980	111.22	26	26	1.05E-20	0.9614	0.9900	v5.3 vs. v6.0	HNU
RH_superiorfrontal_surfavg	ICC2	0.972	115.97	26	26	6.16E-21	0.8909	0.9889	v5.3 vs. v7.1	HNU
RH_superiorfrontal_surfavg	ICC3	0.993	1412.39	26	234	1.46E-241	0.9892	0.9959	v6.0	HNU
RH_superiorfrontal_surfavg	ICC2	0.992	392.47	26	26	9.33E-28	0.9638	0.9967	v6.0 vs. v7.1	HNU
RH_superiorfrontal_surfavg	ICC3	0.984	633.30	26	234	3.06E-201	0.9762	0.9909	v7.1	HNU
RH_superiorfrontal_thickavg	ICC3	0.800	41.00	26	234	1.84E-72	0.7188	0.8743	v5.3	HNU
RH_superiorfrontal_thickavg	ICC2	0.686	18.48	26	26	4.97E-11	-0.0171	0.8857	v5.3 vs. v6.0	HNU
RH_superiorfrontal_thickavg	ICC2	0.669	8.03	26	26	5.37E-07	0.2394	0.8444	v5.3 vs. v7.1	HNU
RH_superiorfrontal_thickavg	ICC3	0.831	50.13	26	234	8.09E-81	0.7590	0.8950	v6.0	HNU
RH_superiorfrontal_thickavg	ICC2	0.817	11.81	26	26	8.43E-09	0.6436	0.9052	v6.0 vs. v7.1	HNU
RH_superiorfrontal_thickavg	ICC3	0.769	34.29	26	234	2.52E-65	0.6797	0.8530	v7.1	HNU
RH_superiorparietal_surfavg	ICC3	0.971	341.28	26	234	1.71E-170	0.9566	0.9832	v5.3	HNU
RH_superiorparietal_surfavg	ICC2	0.911	21.15	26	26	1.01E-11	0.8362	0.9528	v5.3 vs. v6.0	HNU
RH_superiorparietal_surfavg	ICC2	0.910	23.08	26	26	3.54E-12	0.8297	0.9528	v5.3 vs. v7.1	HNU
RH_superiorparietal_surfavg	ICC3	0.983	573.03	26	234	3.06E-196	0.9737	0.9900	v6.0	HNU
RH_superiorparietal_surfavg	ICC2	0.990	306.16	26	26	2.32E-26	0.9681	0.9959	v6.0 vs. v7.1	HNU
RH_superiorparietal_surfavg	ICC3	0.947	180.61	26	234	2.01E-139	0.9207	0.9687	v7.1	HNU
RH_superiorparietal_thickavg	ICC3	0.811	43.83	26	234	3.33E-75	0.7327	0.8816	v5.3	HNU
RH_superiorparietal_thickavg	ICC2	0.545	14.62	26	26	7.60E-10	-0.0503	0.8184	v5.3 vs. v6.0	HNU
RH_superiorparietal_thickavg	ICC2	0.481	11.12	26	26	1.64E-08	-0.0565	0.7768	v5.3 vs. v7.1	HNU
RH_superiorparietal_thickavg	ICC3	0.785	37.47	26	234	7.82E-69	0.6995	0.8639	v6.0	HNU
RH_superiorparietal_thickavg	ICC2	0.872	14.65	26	26	7.39E-10	0.7693	0.9315	v6.0 vs. v7.1	HNU
RH_superiorparietal_thickavg	ICC3	0.748	30.71	26	234	4.69E-61	0.6540	0.8383	v7.1	HNU
RH_superiortemporal_surfavg	ICC3	0.959	232.76	26	234	1.03E-151	0.9374	0.9756	v5.3	HNU
RH_superiortemporal_surfavg	ICC2	0.944	65.84	26	26	8.27E-18	0.7301	0.9791	v5.3 vs. v6.0	HNU
RH_superiortemporal_surfavg	ICC2	0.890	54.12	26	26	9.78E-17	0.2353	0.9648	v5.3 vs. v7.1	HNU
RH_superiortemporal_surfavg	ICC3	0.945	171.26	26	234	7.23E-137	0.9167	0.9671	v6.0	HNU
RH_superiortemporal_surfavg	ICC2	0.963	93.00	26	26	1.03E-19	0.8347	0.9857	v6.0 vs. v7.1	HNU
RH_superiortemporal_surfavg	ICC3	0.951	196.15	26	234	2.09E-143	0.9265	0.9711	v7.1	HNU
RH_superiortemporal_thickavg	ICC3	0.725	27.41	26	234	8.59E-57	0.6264	0.8220	v5.3	HNU

RH_superiortemporal_thickavg	ICC2	0.640	14.93	26	26	5.97E-10	-0.0296	0.8629	v5.3 vs. v6.0	HNU
RH_superiortemporal_thickavg	ICC2	0.715	11.01	26	26	1.83E-08	0.2152	0.8777	v5.3 vs. v7.1	HNU
RH_superiortemporal_thickavg	ICC3	0.773	35.04	26	234	3.60E-66	0.6846	0.8557	v6.0	HNU
RH_superiortemporal_thickavg	ICC2	0.827	15.90	26	26	2.87E-10	0.5393	0.9224	v6.0 vs. v7.1	HNU
RH_superiortemporal_thickavg	ICC3	0.744	30.01	26	234	3.53E-60	0.6485	0.8351	v7.1	HNU
RH_supramarginal_surfavg	ICC3	0.954	206.90	26	234	5.44E-146	0.9301	0.9726	v5.3	HNU
RH_supramarginal_surfavg	ICC2	0.900	18.37	26	26	5.33E-11	0.8156	0.9470	v5.3 vs. v6.0	HNU
RH_supramarginal_surfavg	ICC2	0.885	15.81	26	26	3.08E-10	0.7887	0.9386	v5.3 vs. v7.1	HNU
RH_supramarginal_surfavg	ICC3	0.947	180.31	26	234	2.42E-139	0.9205	0.9687	v6.0	HNU
RH_supramarginal_surfavg	ICC2	0.986	145.92	26	26	3.24E-22	0.9735	0.9928	v6.0 vs. v7.1	HNU
RH_supramarginal_surfavg	ICC3	0.922	119.70	26	234	7.38E-120	0.8845	0.9535	v7.1	HNU
RH_supramarginal_thickavg	ICC3	0.722	27.01	26	234	2.99E-56	0.6227	0.8198	v5.3	HNU
RH_supramarginal_thickavg	ICC2	0.758	28.45	26	26	2.82E-13	0.0044	0.9186	v5.3 vs. v6.0	HNU
RH_supramarginal_thickavg	ICC2	0.806	21.93	26	26	6.53E-12	0.2061	0.9286	v5.3 vs. v7.1	HNU
RH_supramarginal_thickavg	ICC3	0.801	41.23	26	234	1.07E-72	0.7201	0.8749	v6.0	HNU
RH_supramarginal_thickavg	ICC2	0.898	21.57	26	26	7.98E-12	0.7977	0.9479	v6.0 vs. v7.1	HNU
RH_supramarginal_thickavg	ICC3	0.769	34.34	26	234	2.25E-65	0.6800	0.8531	v7.1	HNU
RH_SurfArea	ICC3	0.988	853.25	26	234	3.47E-216	0.9822	0.9932	v5.3	HNU
RH_SurfArea	ICC2	0.984	807.70	26	26	8.11E-32	0.4806	0.9957	v5.3 vs. v6.0	HNU
RH_SurfArea	ICC2	0.935	291.10	26	26	4.44E-26	0.0942	0.9828	v5.3 vs. v7.1	HNU
RH_SurfArea	ICC3	0.991	1116.33	26	234	1.05E-229	0.9863	0.9948	v6.0	HNU
RH_SurfArea	ICC2	0.979	550.45	26	26	1.17E-29	0.4461	0.9943	v6.0 vs. v7.1	HNU
RH_SurfArea	ICC3	0.991	1064.54	26	234	2.60E-227	0.9857	0.9946	v7.1	HNU
RH_temporalpole_surfavg	ICC3	0.630	18.01	26	234	3.11E-42	0.5162	0.7499	v5.3	HNU
RH_temporalpole_surfavg	ICC2	0.701	7.34	26	26	1.34E-06	0.4179	0.8452	v5.3 vs. v6.0	HNU
RH_temporalpole_surfavg	ICC2	0.347	4.17	26	26	2.61E-04	-0.0636	0.6425	v5.3 vs. v7.1	HNU
RH_temporalpole_surfavg	ICC3	0.742	29.79	26	234	6.58E-60	0.6467	0.8341	v6.0	HNU
RH_temporalpole_surfavg	ICC2	0.544	7.53	26	26	1.03E-06	-0.0151	0.7950	v6.0 vs. v7.1	HNU
RH_temporalpole_surfavg	ICC3	0.833	50.77	26	234	2.38E-81	0.7613	0.8962	v7.1	HNU
RH_temporalpole_thickavg	ICC3	0.362	6.66	26	234	7.41E-17	0.2492	0.5115	v5.3	HNU
RH_temporalpole_thickavg	ICC2	0.756	7.29	26	26	1.44E-06	0.5806	0.8650	v5.3 vs. v6.0	HNU
RH_temporalpole_thickavg	ICC2	0.781	8.01	26	26	5.50E-07	0.6179	0.8800	v5.3 vs. v7.1	HNU
RH_temporalpole_thickavg	ICC3	0.388	7.35	26	234	9.53E-19	0.2733	0.5380	v6.0	HNU
RH_temporalpole_thickavg	ICC2	0.762	8.18	26	26	4.41E-07	0.5792	0.8705	v6.0 vs. v7.1	HNU
RH_temporalpole_thickavg	ICC3	0.418	8.18	26	234	5.69E-21	0.3007	0.5666	v7.1	HNU
RH_Thalamus	ICC3	0.723	27.09	26	234	2.32E-56	0.6235	0.8202	v5.3	HNU
RH_Thalamus	ICC2	0.875	22.86	26	26	3.97E-12	0.6354	0.9454	v5.3 vs. v6.0	HNU
RH_Thalamus	ICC2	0.720	15.97	26	26	2.74E-10	0.0572	0.8942	v5.3 vs. v7.1	HNU
RH_Thalamus	ICC3	0.876	71.34	26	234	3.74E-96	0.8189	0.9241	v6.0	HNU
RH_Thalamus	ICC2	0.895	30.88	26	26	1.04E-13	0.6186	0.9575	v6.0 vs. v7.1	HNU
RH_Thalamus	ICC3	0.870	67.75	26	234	7.29E-94	0.8110	0.9204	v7.1	HNU
RH_Thickness	ICC3	0.770	34.56	26	234	1.26E-65	0.6815	0.8540	v5.3	HNU
RH_Thickness	ICC2	0.515	13.41	26	26	2.03E-09	-0.0521	0.8002	v5.3 vs. v6.0	HNU
RH_Thickness	ICC2	0.592	7.11	26	26	1.86E-06	0.0809	0.8106	v5.3 vs. v7.1	HNU

RH_Thickness	ICC3	0.830	49.71	26	234	1.86E-80	0.7574	0.8942	v6.0	HNU
RH_Thickness	ICC2	0.712	9.20	26	26	1.28E-07	0.3196	0.8658	v6.0 vs. v7.1	HNU
RH_Thickness	ICC3	0.770	34.47	26	234	1.56E-65	0.6809	0.8537	v7.1	HNU
RH_transversetemporal_surfavg	ICC3	0.883	76.45	26	234	3.00E-99	0.8292	0.9289	v5.3	HNU
RH_transversetemporal_surfavg	ICC2	0.841	11.61	26	26	1.02E-08	0.7160	0.9137	v5.3 vs. v6.0	HNU
RH_transversetemporal_surfavg	ICC2	0.801	8.83	26	26	1.99E-07	0.6488	0.8917	v5.3 vs. v7.1	HNU
RH_transversetemporal_surfavg	ICC3	0.937	148.67	26	234	4.21E-130	0.9051	0.9622	v6.0	HNU
RH_transversetemporal_surfavg	ICC2	0.925	25.15	26	26	1.26E-12	0.8605	0.9602	v6.0 vs. v7.1	HNU
RH_transversetemporal_surfavg	ICC3	0.938	153.19	26	234	1.57E-131	0.9076	0.9633	v7.1	HNU
RH_transversetemporal_thickavg	ICC3	0.809	43.36	26	234	9.21E-75	0.7305	0.8804	v5.3	HNU
RH_transversetemporal_thickavg	ICC2	0.670	19.97	26	26	1.99E-11	-0.0306	0.8811	v5.3 vs. v6.0	HNU
RH_transversetemporal_thickavg	ICC2	0.686	16.78	26	26	1.54E-10	-0.0067	0.8834	v5.3 vs. v7.1	HNU
RH_transversetemporal_thickavg	ICC3	0.808	43.00	26	234	2.05E-74	0.7287	0.8795	v6.0	HNU
RH_transversetemporal_thickavg	ICC2	0.885	17.24	26	26	1.12E-10	0.7881	0.9385	v6.0 vs. v7.1	HNU
RH_transversetemporal_thickavg	ICC3	0.820	46.62	26	234	9.18E-78	0.7450	0.8879	v7.1	HNU
SUM_bankssts_surfavg	ICC3	0.917	110.84	26	234	2.92E-116	0.8763	0.9499	v5.3	HNU
SUM_bankssts_surfavg	ICC2	0.749	10.20	26	26	4.24E-08	0.4147	0.8814	v5.3 vs. v6.0	HNU
SUM_bankssts_surfavg	ICC2	0.706	7.54	26	26	1.02E-06	0.4203	0.8487	v5.3 vs. v7.1	HNU
SUM_bankssts_surfavg	ICC3	0.956	216.90	26	234	2.80E-148	0.9331	0.9738	v6.0	HNU
SUM_bankssts_surfavg	ICC2	0.963	51.92	26	26	1.65E-16	0.9306	0.9808	v6.0 vs. v7.1	HNU
SUM_bankssts_surfavg	ICC3	0.956	218.53	26	234	1.21E-148	0.9336	0.9740	v7.1	HNU
SUM_caudalanteriorcingulate_surfavg	ICC3	0.980	483.74	26	234	8.57E-188	0.9690	0.9881	v5.3	HNU
SUM_caudalanteriorcingulate_surfavg	ICC2	0.895	45.24	26	26	9.22E-16	0.3767	0.9642	v5.3 vs. v6.0	HNU
SUM_caudalanteriorcingulate_surfavg	ICC2	0.945	51.53	26	26	1.81E-16	0.8398	0.9761	v5.3 vs. v7.1	HNU
SUM_caudalanteriorcingulate_surfavg	ICC3	0.986	712.90	26	234	3.61E-207	0.9788	0.9919	v6.0	HNU
SUM_caudalanteriorcingulate_surfavg	ICC2	0.974	163.83	26	26	7.33E-23	0.8267	0.9911	v6.0 vs. v7.1	HNU
SUM_caudalanteriorcingulate_surfavg	ICC3	0.988	836.64	26	234	3.37E-215	0.9819	0.9931	v7.1	HNU
SUM_caudalmiddlefrontal_surfavg	ICC3	0.981	507.98	26	234	3.14E-190	0.9704	0.9887	v5.3	HNU
SUM_caudalmiddlefrontal_surfavg	ICC2	0.907	22.93	26	26	3.83E-12	0.8214	0.9518	v5.3 vs. v6.0	HNU
SUM_caudalmiddlefrontal_surfavg	ICC2	0.944	37.62	26	26	9.15E-15	0.8917	0.9708	v5.3 vs. v7.1	HNU
SUM_caudalmiddlefrontal_surfavg	ICC3	0.986	684.85	26	234	3.70E-205	0.9779	0.9916	v6.0	HNU
SUM_caudalmiddlefrontal_surfavg	ICC2	0.984	130.07	26	26	1.42E-21	0.9695	0.9917	v6.0 vs. v7.1	HNU
SUM_caudalmiddlefrontal_surfavg	ICC3	0.988	820.99	26	234	2.99E-214	0.9815	0.9930	v7.1	HNU
SUM_cuneus_surfavg	ICC3	0.940	157.77	26	234	6.15E-133	0.9101	0.9644	v5.3	HNU
SUM_cuneus_surfavg	ICC2	0.897	37.11	26	26	1.08E-14	0.5213	0.9616	v5.3 vs. v6.0	HNU
SUM_cuneus_surfavg	ICC2	0.765	33.09	26	26	4.45E-14	-0.0021	0.9228	v5.3 vs. v7.1	HNU
SUM_cuneus_surfavg	ICC3	0.972	345.35	26	234	4.45E-171	0.9570	0.9834	v6.0	HNU
SUM_cuneus_surfavg	ICC2	0.936	102.53	26	26	2.97E-20	0.3557	0.9804	v6.0 vs. v7.1	HNU
SUM_cuneus_surfavg	ICC3	0.964	268.58	26	234	1.04E-158	0.9454	0.9788	v7.1	HNU
SUM_entorhinal_surfavg	ICC3	0.774	35.26	26	234	2.01E-66	0.6861	0.8565	v5.3	HNU
SUM_entorhinal_surfavg	ICC2	0.469	4.15	26	26	2.69E-04	0.0656	0.7127	v5.3 vs. v6.0	HNU
SUM_entorhinal_surfavg	ICC2	0.577	4.71	26	26	9.21E-05	0.2643	0.7670	v5.3 vs. v7.1	HNU
SUM_entorhinal_surfavg	ICC3	0.847	56.31	26	234	8.85E-86	0.7802	0.9056	v6.0	HNU
SUM_entorhinal_surfavg	ICC2	0.902	26.72	26	26	6.06E-13	0.7505	0.9550	v6.0 vs. v7.1	HNU

SUM_entorhinal_surfavg	ICC3	0.876	71.64	26	234	2.45E-96	0.8196	0.9244	v7.1	HNU
SUM_frontalpole_surfavg	ICC3	0.667	21.07	26	234	1.95E-47	0.5586	0.7791	v5.3	HNU
SUM_frontalpole_surfavg	ICC2	0.279	4.57	26	26	1.20E-04	-0.0688	0.5894	v5.3 vs. v6.0	HNU
SUM_frontalpole_surfavg	ICC2	0.194	8.98	26	26	1.66E-07	-0.0257	0.4963	v5.3 vs. v7.1	HNU
SUM_frontalpole_surfavg	ICC3	0.806	42.66	26	234	4.30E-74	0.7271	0.8787	v6.0	HNU
SUM_frontalpole_surfavg	ICC2	0.537	9.70	26	26	7.29E-08	-0.0502	0.8030	v6.0 vs. v7.1	HNU
SUM_frontalpole_surfavg	ICC3	0.832	50.51	26	234	3.86E-81	0.7604	0.8958	v7.1	HNU
SUM_fusiform_surfavg	ICC3	0.956	220.80	26	234	3.79E-149	0.9343	0.9743	v5.3	HNU
SUM_fusiform_surfavg	ICC2	0.771	17.60	26	26	8.82E-11	0.1703	0.9128	v5.3 vs. v6.0	HNU
SUM_fusiform_surfavg	ICC2	0.767	13.42	26	26	2.01E-09	0.3130	0.9012	v5.3 vs. v7.1	HNU
SUM_fusiform_surfavg	ICC3	0.981	523.38	26	234	1.02E-191	0.9713	0.9890	v6.0	HNU
SUM_fusiform_surfavg	ICC2	0.986	143.24	26	26	4.12E-22	0.9724	0.9925	v6.0 vs. v7.1	HNU
SUM_fusiform_surfavg	ICC3	0.977	433.19	26	234	2.64E-182	0.9655	0.9867	v7.1	HNU
SUM_inferiorparietal_surfavg	ICC3	0.982	537.49	26	234	4.79E-193	0.9720	0.9893	v5.3	HNU
SUM_inferiorparietal_surfavg	ICC2	0.941	33.80	26	26	3.43E-14	0.8887	0.9687	v5.3 vs. v6.0	HNU
SUM_inferiorparietal_surfavg	ICC2	0.941	32.78	26	26	4.98E-14	0.8896	0.9687	v5.3 vs. v7.1	HNU
SUM_inferiorparietal_surfavg	ICC3	0.982	540.27	26	234	2.65E-193	0.9722	0.9893	v6.0	HNU
SUM_inferiorparietal_surfavg	ICC2	0.977	184.70	26	26	1.57E-23	0.8436	0.9921	v6.0 vs. v7.1	HNU
SUM_inferiorparietal_surfavg	ICC3	0.981	523.61	26	234	9.69E-192	0.9713	0.9890	v7.1	HNU
SUM_inferiortemporal_surfavg	ICC3	0.949	188.41	26	234	1.84E-141	0.9237	0.9700	v5.3	HNU
SUM_inferiortemporal_surfavg	ICC2	0.878	38.23	26	26	7.48E-15	0.3332	0.9579	v5.3 vs. v6.0	HNU
SUM_inferiortemporal_surfavg	ICC2	0.859	30.80	26	26	1.07E-13	0.3177	0.9495	v5.3 vs. v7.1	HNU
SUM_inferiortemporal_surfavg	ICC3	0.945	171.81	26	234	5.09E-137	0.9169	0.9672	v6.0	HNU
SUM_inferiortemporal_surfavg	ICC2	0.984	123.79	26	26	2.67E-21	0.9703	0.9919	v6.0 vs. v7.1	HNU
SUM_inferiortemporal_surfavg	ICC3	0.965	274.47	26	234	8.99E-160	0.9465	0.9792	v7.1	HNU
SUM_insula_surfavg	ICC3	0.887	79.79	26	234	3.58E-101	0.8353	0.9317	v5.3	HNU
SUM_insula_surfavg	ICC2	0.642	21.93	26	26	6.53E-12	-0.0382	0.8710	v5.3 vs. v6.0	HNU
SUM_insula_surfavg	ICC2	0.649	16.77	26	26	1.55E-10	-0.0324	0.8691	v5.3 vs. v7.1	HNU
SUM_insula_surfavg	ICC3	0.890	82.01	26	234	2.05E-102	0.8391	0.9334	v6.0	HNU
SUM_insula_surfavg	ICC2	0.889	16.63	26	26	1.71E-10	0.7967	0.9406	v6.0 vs. v7.1	HNU
SUM_insula_surfavg	ICC3	0.900	90.74	26	234	5.12E-107	0.8525	0.9395	v7.1	HNU
SUM_isthmuscingulate_surfavg	ICC3	0.895	86.60	26	234	6.90E-105	0.8464	0.9367	v5.3	HNU
SUM_isthmuscingulate_surfavg	ICC2	0.826	13.95	26	26	1.30E-09	0.6089	0.9157	v5.3 vs. v6.0	HNU
SUM_isthmuscingulate_surfavg	ICC2	0.844	14.32	26	26	9.60E-10	0.6856	0.9208	v5.3 vs. v7.1	HNU
SUM_isthmuscingulate_surfavg	ICC3	0.983	562.47	26	234	2.59E-195	0.9732	0.9898	v6.0	HNU
SUM_isthmuscingulate_surfavg	ICC2	0.976	91.04	26	26	1.35E-19	0.9529	0.9878	v6.0 vs. v7.1	HNU
SUM_isthmuscingulate_surfavg	ICC3	0.982	554.58	26	234	1.32E-194	0.9729	0.9896	v7.1	HNU
SUM_lateraloccipital_surfavg	ICC3	0.970	327.40	26	234	1.92E-168	0.9548	0.9825	v5.3	HNU
SUM_lateraloccipital_surfavg	ICC2	0.792	32.65	26	26	5.24E-14	0.0314	0.9315	v5.3 vs. v6.0	HNU
SUM_lateraloccipital_surfavg	ICC2	0.662	35.46	26	26	1.90E-14	-0.0280	0.8848	v5.3 vs. v7.1	HNU
SUM_lateraloccipital_surfavg	ICC3	0.984	632.59	26	234	3.48E-201	0.9761	0.9909	v6.0	HNU
SUM_lateraloccipital_surfavg	ICC2	0.944	140.71	26	26	5.17E-22	0.3116	0.9836	v6.0 vs. v7.1	HNU
SUM_lateraloccipital_surfavg	ICC3	0.979	474.40	26	234	7.99E-187	0.9684	0.9879	v7.1	HNU
SUM_lateralorbitofrontal_surfavg	ICC3	0.829	49.60	26	234	2.28E-80	0.7570	0.8940	v5.3	HNU

SUM_lateralorbitofrontal_surfavg	ICC2	0.924	58.72	26	26	3.51E-17	0.5259	0.9737	v5.3 vs. v6.0	HNU
SUM_lateralorbitofrontal_surfavg	ICC2	0.736	21.35	26	26	9.02E-12	0.0161	0.9064	v5.3 vs. v7.1	HNU
SUM_lateralorbitofrontal_surfavg	ICC3	0.857	60.75	26	234	4.49E-89	0.7933	0.9119	v6.0	HNU
SUM_lateralorbitofrontal_surfavg	ICC2	0.883	40.95	26	26	3.19E-15	0.3295	0.9599	v6.0 vs. v7.1	HNU
SUM_lateralorbitofrontal_surfavg	ICC3	0.831	50.33	26	234	5.56E-81	0.7597	0.8954	v7.1	HNU
SUM_lingual_surfavg	ICC3	0.929	132.44	26	234	1.28E-124	0.8945	0.9578	v5.3	HNU
SUM_lingual_surfavg	ICC2	0.865	13.83	26	26	1.43E-09	0.7573	0.9276	v5.3 vs. v6.0	HNU
SUM_lingual_surfavg	ICC2	0.748	10.22	26	26	4.14E-08	0.4062	0.8812	v5.3 vs. v7.1	HNU
SUM_lingual_surfavg	ICC3	0.972	348.03	26	234	1.84E-171	0.9574	0.9836	v6.0	HNU
SUM_lingual_surfavg	ICC2	0.911	60.82	26	26	2.25E-17	0.3561	0.9710	v6.0 vs. v7.1	HNU
SUM_lingual_surfavg	ICC3	0.973	356.03	26	234	1.39E-172	0.9583	0.9839	v7.1	HNU
SUM_medialorbitofrontal_surfavg	ICC3	0.773	34.97	26	234	4.24E-66	0.6842	0.8555	v5.3	HNU
SUM_medialorbitofrontal_surfavg	ICC2	0.884	22.91	26	26	3.88E-12	0.7004	0.9472	v5.3 vs. v6.0	HNU
SUM_medialorbitofrontal_surfavg	ICC2	0.503	12.76	26	26	3.56E-09	-0.0532	0.7927	v5.3 vs. v7.1	HNU
SUM_medialorbitofrontal_surfavg	ICC3	0.822	47.19	26	234	2.83E-78	0.7474	0.8892	v6.0	HNU
SUM_medialorbitofrontal_surfavg	ICC2	0.671	20.87	26	26	1.18E-11	-0.0317	0.8821	v6.0 vs. v7.1	HNU
SUM_medialorbitofrontal_surfavg	ICC3	0.845	55.55	26	234	3.41E-85	0.7778	0.9044	v7.1	HNU
SUM_middletemporal_surfavg	ICC3	0.966	285.35	26	234	1.11E-161	0.9484	0.9800	v5.3	HNU
SUM_middletemporal_surfavg	ICC2	0.867	35.92	26	26	1.62E-14	0.2825	0.9541	v5.3 vs. v6.0	HNU
SUM_middletemporal_surfavg	ICC2	0.781	27.75	26	26	3.82E-13	0.0410	0.9258	v5.3 vs. v7.1	HNU
SUM_middletemporal_surfavg	ICC3	0.979	470.39	26	234	2.11E-186	0.9681	0.9878	v6.0	HNU
SUM_middletemporal_surfavg	ICC2	0.967	134.09	26	26	9.60E-22	0.7659	0.9888	v6.0 vs. v7.1	HNU
SUM_middletemporal_surfavg	ICC3	0.985	638.23	26	234	1.25E-201	0.9763	0.9910	v7.1	HNU
SUM_paracentral_surfavg	ICC3	0.963	257.69	26	234	1.11E-156	0.9432	0.9779	v5.3	HNU
SUM_paracentral_surfavg	ICC2	0.900	20.11	26	26	1.83E-11	0.8136	0.9468	v5.3 vs. v6.0	HNU
SUM_paracentral_surfavg	ICC2	0.905	20.90	26	26	1.16E-11	0.8242	0.9495	v5.3 vs. v7.1	HNU
SUM_paracentral_surfavg	ICC3	0.969	318.84	26	234	3.87E-167	0.9536	0.9821	v6.0	HNU
SUM_paracentral_surfavg	ICC2	0.982	106.99	26	26	1.73E-20	0.9657	0.9906	v6.0 vs. v7.1	HNU
SUM_paracentral_surfavg	ICC3	0.971	336.61	26	234	8.19E-170	0.9560	0.9830	v7.1	HNU
SUM_parahippocampal_surfavg	ICC3	0.745	30.15	26	234	2.35E-60	0.6496	0.8358	v5.3	HNU
SUM_parahippocampal_surfavg	ICC2	0.553	11.00	26	26	1.86E-08	-0.0502	0.8151	v5.3 vs. v6.0	HNU
SUM_parahippocampal_surfavg	ICC2	0.524	11.30	26	26	1.37E-08	-0.0555	0.8012	v5.3 vs. v7.1	HNU
SUM_parahippocampal_surfavg	ICC3	0.954	209.01	26	234	1.75E-146	0.9308	0.9729	v6.0	HNU
SUM_parahippocampal_surfavg	ICC2	0.925	26.34	26	26	7.21E-13	0.8619	0.9605	v6.0 vs. v7.1	HNU
SUM_parahippocampal_surfavg	ICC3	0.945	173.40	26	234	1.83E-137	0.9176	0.9675	v7.1	HNU
SUM_parsopercularis_surfavg	ICC3	0.985	679.33	26	234	9.41E-205	0.9777	0.9915	v5.3	HNU
SUM_parsopercularis_surfavg	ICC2	0.916	28.59	26	26	2.66E-13	0.8112	0.9591	v5.3 vs. v6.0	HNU
SUM_parsopercularis_surfavg	ICC2	0.911	24.84	26	26	1.46E-12	0.8218	0.9546	v5.3 vs. v7.1	HNU
SUM_parsopercularis_surfavg	ICC3	0.993	1337.73	26	234	8.01E-239	0.9886	0.9957	v6.0	HNU
SUM_parsopercularis_surfavg	ICC2	0.985	126.50	26	26	2.02E-21	0.9707	0.9920	v6.0 vs. v7.1	HNU
SUM_parsopercularis_surfavg	ICC3	0.992	1251.65	26	234	1.81E-235	0.9878	0.9954	v7.1	HNU
SUM_parsorbitalis_surfavg	ICC3	0.941	160.52	26	234	9.18E-134	0.9115	0.9649	v5.3	HNU
SUM_parsorbitalis_surfavg	ICC2	0.770	26.72	26	26	6.06E-13	0.0287	0.9217	v5.3 vs. v6.0	HNU
SUM_parsorbitalis_surfavg	ICC2	0.638	21.26	26	26	9.50E-12	-0.0388	0.8691	v5.3 vs. v7.1	HNU

SUM_parsorbitalis_surfavg	ICC3	0.970	326.84	26	234	2.33E-168	0.9547	0.9825	v6.0	HNU
SUM_parsorbitalis_surfavg	ICC2	0.940	103.76	26	26	2.55E-20	0.4089	0.9815	v6.0 vs. v7.1	HNU
SUM_parsorbitalis_surfavg	ICC3	0.977	429.73	26	234	6.61E-182	0.9652	0.9866	v7.1	HNU
SUM_parstriangularis_surfavg	ICC3	0.980	485.04	26	234	6.29E-188	0.9691	0.9881	v5.3	HNU
SUM_parstriangularis_surfavg	ICC2	0.886	23.60	26	26	2.71E-12	0.6990	0.9485	v5.3 vs. v6.0	HNU
SUM_parstriangularis_surfavg	ICC2	0.841	23.66	26	26	2.64E-12	0.3631	0.9394	v5.3 vs. v7.1	HNU
SUM_parstriangularis_surfavg	ICC3	0.988	806.65	26	234	2.29E-213	0.9812	0.9928	v6.0	HNU
SUM_parstriangularis_surfavg	ICC2	0.979	211.81	26	26	2.69E-24	0.8394	0.9929	v6.0 vs. v7.1	HNU
SUM_parstriangularis_surfavg	ICC3	0.992	1169.68	26	234	4.69E-232	0.9870	0.9951	v7.1	HNU
SUM_pericalcarine_surfavg	ICC3	0.916	109.35	26	234	1.24E-115	0.8748	0.9493	v5.3	HNU
SUM_pericalcarine_surfavg	ICC2	0.884	41.04	26	26	3.10E-15	0.3386	0.9603	v5.3 vs. v6.0	HNU
SUM_pericalcarine_surfavg	ICC2	0.852	29.39	26	26	1.90E-13	0.2961	0.9468	v5.3 vs. v7.1	HNU
SUM_pericalcarine_surfavg	ICC3	0.959	232.73	26	234	1.04E-151	0.9374	0.9756	v6.0	HNU
SUM_pericalcarine_surfavg	ICC2	0.977	87.00	26	26	2.41E-19	0.9554	0.9878	v6.0 vs. v7.1	HNU
SUM_pericalcarine_surfavg	ICC3	0.967	297.62	26	234	9.49E-164	0.9505	0.9808	v7.1	HNU
SUM_postcentral_surfavg	ICC3	0.970	325.87	26	234	3.26E-168	0.9546	0.9825	v5.3	HNU
SUM_postcentral_surfavg	ICC2	0.964	55.17	26	26	7.69E-17	0.9328	0.9813	v5.3 vs. v6.0	HNU
SUM_postcentral_surfavg	ICC2	0.960	51.76	26	26	1.71E-16	0.9236	0.9791	v5.3 vs. v7.1	HNU
SUM_postcentral_surfavg	ICC3	0.977	432.14	26	234	3.49E-182	0.9654	0.9867	v6.0	HNU
SUM_postcentral_surfavg	ICC2	0.979	171.01	26	26	4.22E-23	0.8977	0.9921	v6.0 vs. v7.1	HNU
SUM_postcentral_surfavg	ICC3	0.965	280.69	26	234	7.14E-161	0.9476	0.9797	v7.1	HNU
SUM_posteriorcingulate_surfavg	ICC3	0.970	321.00	26	234	1.80E-167	0.9539	0.9822	v5.3	HNU
SUM_posteriorcingulate_surfavg	ICC2	0.975	81.36	26	26	5.66E-19	0.9516	0.9868	v5.3 vs. v6.0	HNU
SUM_posteriorcingulate_surfavg	ICC2	0.956	58.88	26	26	3.39E-17	0.8903	0.9797	v5.3 vs. v7.1	HNU
SUM_posteriorcingulate_surfavg	ICC3	0.981	523.00	26	234	1.11E-191	0.9713	0.9890	v6.0	HNU
SUM_posteriorcingulate_surfavg	ICC2	0.981	131.63	26	26	1.22E-21	0.9560	0.9910	v6.0 vs. v7.1	HNU
SUM_posteriorcingulate_surfavg	ICC3	0.986	683.48	26	234	4.67E-205	0.9779	0.9916	v7.1	HNU
SUM_precentral_surfavg	ICC3	0.981	515.73	26	234	5.53E-191	0.9709	0.9888	v5.3	HNU
SUM_precentral_surfavg	ICC2	0.965	77.44	26	26	1.06E-18	0.9006	0.9843	v5.3 vs. v6.0	HNU
SUM_precentral_surfavg	ICC2	0.966	57.63	26	26	4.44E-17	0.9360	0.9822	v5.3 vs. v7.1	HNU
SUM_precentral_surfavg	ICC3	0.976	407.17	26	234	3.13E-179	0.9634	0.9859	v6.0	HNU
SUM_precentral_surfavg	ICC2	0.988	278.95	26	26	7.70E-26	0.9488	0.9953	v6.0 vs. v7.1	HNU
SUM_precentral_surfavg	ICC3	0.969	316.03	26	234	1.06E-166	0.9532	0.9819	v7.1	HNU
SUM_precuneus_surfavg	ICC3	0.985	642.13	26	234	6.21E-202	0.9765	0.9910	v5.3	HNU
SUM_precuneus_surfavg	ICC2	0.974	91.83	26	26	1.21E-19	0.9410	0.9873	v5.3 vs. v6.0	HNU
SUM_precuneus_surfavg	ICC2	0.973	95.30	26	26	7.56E-20	0.9345	0.9875	v5.3 vs. v7.1	HNU
SUM_precuneus_surfavg	ICC3	0.990	1016.87	26	234	5.26E-225	0.9850	0.9943	v6.0	HNU
SUM_precuneus_surfavg	ICC2	0.996	454.07	26	26	1.41E-28	0.9918	0.9978	v6.0 vs. v7.1	HNU
SUM_precuneus_surfavg	ICC3	0.991	1165.80	26	234	6.90E-232	0.9869	0.9950	v7.1	HNU
SUM_rostralanteriorcingulate_surfavg	ICC3	0.931	135.74	26	234	8.81E-126	0.8969	0.9588	v5.3	HNU
SUM_rostralanteriorcingulate_surfavg	ICC2	0.897	35.74	26	26	1.72E-14	0.5493	0.9610	v5.3 vs. v6.0	HNU
SUM_rostralanteriorcingulate_surfavg	ICC2	0.913	24.76	26	26	1.53E-12	0.8294	0.9550	v5.3 vs. v7.1	HNU
SUM_rostralanteriorcingulate_surfavg	ICC3	0.943	165.10	26	234	4.13E-135	0.9138	0.9659	v6.0	HNU
SUM_rostralanteriorcingulate_surfavg	ICC2	0.956	60.93	26	26	2.20E-17	0.8794	0.9801	v6.0 vs. v7.1	HNU

SUM_rostralanteriorcingulate_surfavg	ICC3	0.959	232.63	26	234	1.09E-151	0.9374	0.9756	v7.1	HNU
SUM_rostralmiddlefrontal_surfavg	ICC3	0.985	636.90	26	234	1.59E-201	0.9763	0.9910	v5.3	HNU
SUM_rostralmiddlefrontal_surfavg	ICC2	0.947	50.31	26	26	2.44E-16	0.8559	0.9759	v5.3 vs. v6.0	HNU
SUM_rostralmiddlefrontal_surfavg	ICC2	0.939	70.87	26	26	3.26E-18	0.6140	0.9787	v5.3 vs. v7.1	HNU
SUM_rostralmiddlefrontal_surfavg	ICC3	0.993	1478.85	26	234	6.97E-244	0.9897	0.9961	v6.0	HNU
SUM_rostralmiddlefrontal_surfavg	ICC2	0.991	306.65	26	26	2.27E-26	0.9714	0.9960	v6.0 vs. v7.1	HNU
SUM_rostralmiddlefrontal_surfavg	ICC3	0.993	1374.24	26	234	3.51E-240	0.9889	0.9958	v7.1	HNU
SUM_superiorfrontal_surfavg	ICC3	0.990	995.73	26	234	6.00E-224	0.9847	0.9942	v5.3	HNU
SUM_superiorfrontal_surfavg	ICC2	0.982	198.40	26	26	6.24E-24	0.9167	0.9933	v5.3 vs. v6.0	HNU
SUM_superiorfrontal_surfavg	ICC2	0.962	147.44	26	26	2.84E-22	0.6162	0.9881	v5.3 vs. v7.1	HNU
SUM_superiorfrontal_surfavg	ICC3	0.994	1787.44	26	234	1.87E-253	0.9914	0.9968	v6.0	HNU
SUM_superiorfrontal_surfavg	ICC2	0.992	599.84	26	26	3.84E-30	0.9182	0.9973	v6.0 vs. v7.1	HNU
SUM_superiorfrontal_surfavg	ICC3	0.992	1299.79	26	234	2.26E-237	0.9883	0.9955	v7.1	HNU
SUM_superiorparietal_surfavg	ICC3	0.980	501.28	26	234	1.44E-189	0.9700	0.9885	v5.3	HNU
SUM_superiorparietal_surfavg	ICC2	0.939	35.46	26	26	1.90E-14	0.8791	0.9685	v5.3 vs. v6.0	HNU
SUM_superiorparietal_surfavg	ICC2	0.924	36.11	26	26	1.51E-14	0.7924	0.9662	v5.3 vs. v7.1	HNU
SUM_superiorparietal_surfavg	ICC3	0.989	930.38	26	234	1.56E-220	0.9837	0.9938	v6.0	HNU
SUM_superiorparietal_surfavg	ICC2	0.993	604.53	26	26	3.47E-30	0.9425	0.9975	v6.0 vs. v7.1	HNU
SUM_superiorparietal_surfavg	ICC3	0.976	403.99	26	234	7.67E-179	0.9631	0.9858	v7.1	HNU
SUM_superiortemporal_surfavg	ICC3	0.977	425.59	26	234	2.00E-181	0.9649	0.9865	v5.3	HNU
SUM_superiortemporal_surfavg	ICC2	0.917	81.80	26	26	5.28E-19	0.2617	0.9746	v5.3 vs. v6.0	HNU
SUM_superiortemporal_surfavg	ICC2	0.849	90.30	26	26	1.50E-19	0.0175	0.9562	v5.3 vs. v7.1	HNU
SUM_superiortemporal_surfavg	ICC3	0.975	394.42	26	234	1.18E-177	0.9622	0.9855	v6.0	HNU
SUM_superiortemporal_surfavg	ICC2	0.969	180.76	26	26	2.07E-23	0.6523	0.9902	v6.0 vs. v7.1	HNU
SUM_superiortemporal_surfavg	ICC3	0.981	507.91	26	234	3.19E-190	0.9704	0.9887	v7.1	HNU
SUM_supramarginal_surfavg	ICC3	0.974	373.29	26	234	6.32E-175	0.9601	0.9846	v5.3	HNU
SUM_supramarginal_surfavg	ICC2	0.914	40.80	26	26	3.34E-15	0.6338	0.9667	v5.3 vs. v6.0	HNU
SUM_supramarginal_surfavg	ICC2	0.889	34.80	26	26	2.40E-14	0.4852	0.9587	v5.3 vs. v7.1	HNU
SUM_supramarginal_surfavg	ICC3	0.984	635.88	26	234	1.92E-201	0.9763	0.9909	v6.0	HNU
SUM_supramarginal_surfavg	ICC2	0.993	340.62	26	26	5.83E-27	0.9815	0.9965	v6.0 vs. v7.1	HNU
SUM_supramarginal_surfavg	ICC3	0.977	429.72	26	234	6.62E-182	0.9652	0.9866	v7.1	HNU
SUM_SurfArea	ICC3	0.991	1101.75	26	234	4.84E-229	0.9862	0.9948	v5.3	HNU
SUM_SurfArea	ICC2	0.980	918.96	26	26	1.52E-32	0.3200	0.9947	v5.3 vs. v6.0	HNU
SUM_SurfArea	ICC2	0.932	381.11	26	26	1.36E-27	0.0716	0.9822	v5.3 vs. v7.1	HNU
SUM_SurfArea	ICC3	0.994	1653.78	26	234	1.58E-249	0.9907	0.9965	v6.0	HNU
SUM_SurfArea	ICC2	0.983	887.52	26	26	2.39E-32	0.4093	0.9955	v6.0 vs. v7.1	HNU
SUM_SurfArea	ICC3	0.994	1578.65	26	234	3.52E-247	0.9903	0.9963	v7.1	HNU
SUM_temporalpole_surfavg	ICC3	0.752	31.31	26	234	8.35E-62	0.6586	0.8410	v5.3	HNU
SUM_temporalpole_surfavg	ICC2	0.714	6.04	26	26	9.17E-06	0.5166	0.8401	v5.3 vs. v6.0	HNU
SUM_temporalpole_surfavg	ICC2	0.399	4.10	26	26	3.03E-04	-0.0293	0.6753	v5.3 vs. v7.1	HNU
SUM_temporalpole_surfavg	ICC3	0.877	72.06	26	234	1.34E-96	0.8205	0.9248	v6.0	HNU
SUM_temporalpole_surfavg	ICC2	0.633	13.73	26	26	1.55E-09	-0.0274	0.8575	v6.0 vs. v7.1	HNU
SUM_temporalpole_surfavg	ICC3	0.895	86.63	26	234	6.67E-105	0.8465	0.9367	v7.1	HNU
SUM_transversetemporal_surfavg	ICC3	0.952	199.86	26	234	2.59E-144	0.9278	0.9717	v5.3	HNU

SUM_transversetemporal_surfavg	ICC2	0.924	27.03	26	26	5.28E-13	0.8556	0.9599	v5.3 vs. v6.0	HNU
SUM_transversetemporal_surfavg	ICC2	0.910	20.39	26	26	1.56E-11	0.8324	0.9521	v5.3 vs. v7.1	HNU
SUM_transversetemporal_surfavg	ICC3	0.969	308.81	26	234	1.45E-165	0.9522	0.9815	v6.0	HNU
SUM_transversetemporal_surfavg	ICC2	0.963	62.26	26	26	1.68E-17	0.9202	0.9815	v6.0 vs. v7.1	HNU
SUM_transversetemporal_surfavg	ICC3	0.974	378.49	26	234	1.30E-175	0.9607	0.9849	v7.1	HNU
